# Catalytic Asymmetric Fluorination of Copper Carbene Complexes: Preparative Advances and a Mechanistic Rationale

**DOI:** 10.1002/chem.202000081

**Published:** 2020-02-18

**Authors:** Michael Buchsteiner, Luis Martinez‐Rodriguez, Paul Jerabek, Iago Pozo, Michael Patzer, Nils Nöthling, Christian W. Lehmann, Alois Fürstner

**Affiliations:** ^1^ Max-Planck-Institut für Kohlenforschung 45470 Mülheim/Ruhr Germany; ^2^ Present Address: Nanotechnology Department Helmholtz-Zentrum Geesthacht 21502 Geesthacht Germany

**Keywords:** asymmetric catalysis, bisoxazoline ligands, carbene complexes, copper, organofluorine compounds

## Abstract

The Cu‐catalyzed reaction of substituted α‐diazoesters with fluoride gives α‐fluoroesters with *ee* values of up to 95 %, provided that chiral indane‐derived bis(oxazoline) ligands are used that carry bulky benzyl substituents at the bridge and moderately bulky isopropyl groups on their core. The apparently homogeneous solution of CsF in C_6_F_6_/hexafluoroisopropanol (HFIP) is the best reaction medium, but CsF in the biphasic mixture CH_2_Cl_2_/HFIP also provides good results. DFT studies suggest that fluoride initially attacks the Cu‐ rather than the C‐atom of the transient donor/acceptor carbene intermediate. This unusual step is followed by 1,2‐fluoride shift; for this migratory insertion to occur, the carbene must rotate about the Cu−C bond to ensure orbital overlap. The directionality of this rotatory movement within the *C*_2_‐symmetric binding site determines the sense of induction. This model is in excellent accord with the absolute configuration of the resulting product as determined by X‐ray diffraction using single crystals of this a priori wax‐like material grown by capillary crystallization.

## Graphical abstract

**Let's work together**: Even though bisoxazolines are privileged ligands in asymmetric catalysis, standard representatives give poor *ee* values in the copper catalyzed asymmetric fluorination of α‐diazoesters. The outcome was greatly improved, however, upon *gem*‐benzylation of the bridge, proper substitution of the core, and optimization of the medium. The reaction follows an unusual course in that the incoming fluoride first hits the metal rather than the C‐atom of the transient copper carbene complex.

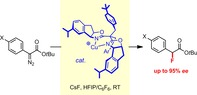

## Introduction

The incorporation of fluorine into active pharmaceutical ingredients (API′s) or agrochemicals often entails substantial advantages in chemical and/or biological terms.^1^ (Chiral) α‐fluoro carbonyl compounds represent a privileged motif and are therefore prominently featured in API′s;^1^, ^2^ at the same time, they constitute valuable building blocks for further elaboration. Most methods for their synthesis invoke the carbonyl compound as (masked) enolate or enamine; the price to pay is the need for an electrophilic fluorine source as the reaction partner.^3^, ^4^, ^5^ The alternative approach of using an “umpoled” substrate in combination with an ordinary fluoride salt is currently less developed, despite potential chemical virtues and practical advantages.^6^, ^7^, ^8^

**Scheme 1:**
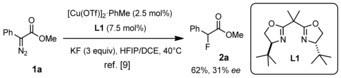
Lead finding of a copper catalyzed formation of α‐fluoroesters.

A recent report on the fluorination of highly electrophilic copper carbenes generated in situ provides an interesting foray in this direction (Scheme 1).^9^, ^10^ Specifically, it was shown that α‐diazoesters such as **1 a**, on treatment with catalytic amounts of [Cu(MeCN)_4_]PF_6_ (or [Cu(OTf)]_2_⋅PhMe) and **L1** as the preferred ligand, react with excess KF in a biphasic mixture comprised of hexafluoroisopropanol (HFIP) and 1,2‐dichloroethane (DCE) at 40 °C to give the corresponding α‐fluoroester **2 a** in good yield. Despite the excellent track record of chiral bis(oxazolines) (BOX) and related ligands in asymmetric catalysis,^11^, ^12^ however, only poor enantioselectivity (≤31 % *ee*) was reached. Use of a monodentate chiral phosphoramidite in lieu of **L1** entailed higher optical purity (86 % *ee*) at the expense of the chemical yield, which dropped to only 12 %.^9^

## Results and Discussion

### Reaction optimization and scope

As part of our studies into structure, bonding and reactivity of organotransition metal carbene complexes,^13^, ^14^, ^15^, ^16^, ^17^, ^18^ we sought to improve on these lead findings. We were fully apprehensive that the small size of the fluoride ion constitutes a formidable and inherent challenge for asymmetric catalysis; moreover, any uncatalyzed and hence racemic background reaction had to be prevented.^19^ To this end, it was deemed imperative to find milder reaction conditions and to carry out a broad ligand screening. The first goal was readily achieved in that the use of CsF instead of KF allowed the reaction to proceed at ambient temperature in the biphasic mixture CH_2_Cl_2_/HFIP as a ligand‐accelerated process.^20^ Of arguably higher relevance—even beyond the present context—is the observation that mixtures of HFIP and hexafluorobenzene form an apparently homogeneous phase capable of dissolving CsF, in which the α‐fluorination proceeds particularly cleanly.

Not unexpectedly, the search for effective chiral ligands proved challenging, not least because clear‐cut structure/selectivity relationships were difficult to deduce from the acquired data (for the full list, see the Supporting Information). As one consistent trend, however, it was noticed that standard BOX ligands led to clean reactions. The poor chiral induction notwithstanding (cf. Scheme 1 and the Supporting Information),^9^ the ligand optimization exercise remained largely focused on this privileged scaffold.^11^, ^12^ Our efforts were guided by the notion that efficient steering of the small incoming fluoride anion likely mandates a tight chiral binding site. We conjectured that this goal might be reached by adjustment of the following three parameters: (i) implementation of substituents at the methylene bridge to exert remote control,^21^, ^22^ (ii) extension of the lateral “walls” of the *C*_2_‐symmetric ligand scaffold beyond the *tert*‐butyl substituents in **L1**, and (iii) increase of the size of the electrophilic partner by using bulkier α‐diazoesters.

**Figure 1:**
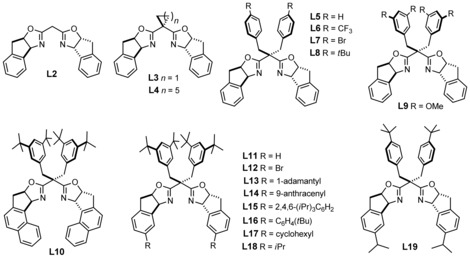
Subset of the tested BOX‐ligands; for the full list, see the Supporting Information.

Variation of any of these factors alone did not grant success; gratifyingly though, they were found to synergize. While the commercially available indane‐based bis(oxazoline) **L2** gave methyl ester **2 a** with only 31 % *ee* (Figure 1) and the corresponding *tert*‐butyl ester **2 b** with equally disappointing 35 % *ee* (Table 1, entries 1, 2), *gem*‐disubstitution of the methylene bridge entailed some improvement. This effect has ample precedent in the literature and is usually ascribed to a buttressing effect, the fine‐tuning of the ligand's bite‐angle, and suppression of enolization at this site.^11^, ^12^ Although these factors are likely relevant in the present context too, additional aspects seem to play an important role. Specifically, introduction of a cycloheptyl or cyclopropyl group improved the *ee* to 45 % and 54 % for product **2 b**, respectively (entries 3, 4). Equally good or even better results were obtained with ligands such as **L5**–**L9** carrying different benzyl substituents at this position, which furnished **2 b** with *ee* values of up to 75 % (entries 5–9);^23^ moreover, the size of the ester group in the diazo compound had a marked effect (cf. entries 11 and 12).

**Table 1:** Ligand screening and optimization of the reaction conditions

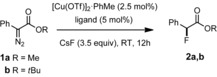				
Entry	R	Medium^[a]^	Ligand	*ee* [%]^[b]^ (yield)
1	Me	**A**	**L2**	31
2	*t*Bu	**A**	**L2**	35
3	*t*Bu	**A**	**L3**	54
4	*t*Bu	**A**	**L4**	45
5	*t*Bu	**A**	**L5**	66
6	*t*Bu	**A**	**L6**	61
7	*t*Bu	**A**	**L7**	59
8	*t*Bu	**A**	**L8**	75
9	*t*Bu	**A**	**L9**	71
10	*t*Bu	**A**	**L10**	16
11	Me	**A**	**L11**	52
12	*t*Bu	**A**	**L11**	73
13	*t*Bu	**A**	**L12**	76
14	*t*Bu	**A**	**L13**	66
15	*t*Bu	**A**	**L14**	71
16	*t*Bu	**A**	**L15**	52
17	*t*Bu	**A**	**L16**	72
18	*t*Bu	**A**	**L17**	70
19	*t*Bu	**A**	**L18**	81
20	*t*Bu	**A**	**L19**	85 (72)^[c]^
21	*t*Bu	**A**	**L19**	87 (65)^[c,d]^
22	*t*Bu	**B**	**L19**	89 (87)^[c]^

[a] **A**: HFIP (10 equiv), CH_2_Cl_2_; **B**: HFIP (10 equiv), C_6_F_6_. [b] *ee* of the isolated pure compound; for details, see the Supporting Information. [c] Isolated yield of analytically pure product. [d] Using [Cu(O*t*Bu)]_4_ instead of [Cu(OTf)]_2_⋅PhMe

**Figure 2:**
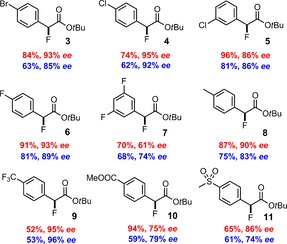
Chiral α‐fluoroester derivatives prepared by copper‐catalyzed diazoester decomposition. Reaction conditions: [Cu(OTf)]_2_⋅PhMe (2.5 mol %), **L19** (5 mol %), CsF (3.5 equiv), RT, 12 h, HFIP (10 equiv)/CH_2_Cl_2_ (blue) or HFIP (10 equiv)/C_6_F_6_ (red).

Next, we considered placement of substituents at strategic positions of the indane core or an extension of the scaffold by annulation of additional aryl rings. Such modifications are rare,^24^, ^25^ but it was hoped that they might entail a narrower trajectory for the incoming fluoride anion. Screening of a number of variants showed that bis(oxazoline) **L19** carrying bulky *p‐tert*‐butyl substituted benzyl moieties at the bridge and an only moderately big isopropyl substituent on the indane nucleus gave the best result (for the full data set, see the Supporting Information). This particular ligand furnished α‐fluorophenylacetate **2 b** in 87 % yield with 89 % *ee* when the reaction was carried out in HFIP/C_6_F_6_ as the optimal medium (entry 22). More electron‐deficient α‐diazocarbonyl derivatives performed even better, especially those carrying the electron‐withdrawing group at the *para*‐position of the aryl ring (Figure 2); the corresponding products were isolated in high yields with optical purities of up to 95 % *ee*. Comparison of the results obtained for products **4** and **5** illustrates the influence of the substitution pattern on the outcome; in this context it is noteworthy that attempted formation of the corresponding *ortho*‐chlorinated analogue failed due to decomposition of the diazo ester precursor.

### Configurational assignment via capillary crystallization

Since none of the resulting α‐fluorinated esters shown in Figure 2 had previously been prepared in the literature in optically active form, we faced the need to rigorously establish the absolute configuration prior to any mechanistic discussion.^26^ To this end, a sample of **2 b** (89 % *ee*) as the parent compound of this series was subjected to preparative HPLC on a chiral stationary phase to remove the remaining minor enantiomer. Low temperature differential scanning calorimetry showed that **2 b** (*ee* ≥99 %) features a broad melting range close to ambient temperature (ca. +3 to +22 °C) and a remarkably strong hysteresis upon cooling (see the Supporting Information). Therefore crystallization was expected to be non‐trivial.

**Figure 3:**
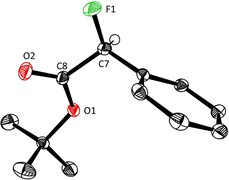
Structure of compound **2 b** in the solid state which allowed the absolute configuration of the α‐fluorinated ester derivative to be determined; all hydrogen atoms—except the one on the (*S*)‐configured chiral center C7—are omitted for clarity.

The challenge was met by capillary crystallization: the neat “liquid” sample was filled into a capillary, which was tightly fused before being chilled in a stream of cold air; the resulting polycrystalline material was then locally warmed to slightly above melting temperature. This cooling/warming cycle led to single crystals suitable for X‐ray diffraction. The capillary was transferred quickly to the diffractometer; the collected data set afforded a statistically significant absolute structure parameter and allowed the absolute configuration to be determined;^27^ to the best of our knowledge is this the first example of its kind^28^ and bodes well for configurational assignments of other chiral liquid samples in the future. As shown in Figure 3, the major isomer of the α‐fluoroester **2 b** (${\left[\alpha \right]}_{D}^{20}$ =+65.9) formed in the copper catalyzed reaction with ligand **L19** is (*S*)‐configured. All other compounds shown in Figure 2 were assigned by analogy, based on the fact that they are invariably dextrorotatory.

### Mechanistic discussion

Despite numerous attempts, we failed to grow single crystals of the copper precatalyst carrying the optimal ligand **L19**, most likely because of the greasy lateral substituents. Therefore, we resorted to solving the structure of the related complex derived from **L3**, which leads to the same sense of induction in the fluorination reaction but to a lower *ee* of 54 % (Table 1, entry 3). In contrast to the copious information on BOX complexes of Cu^II^ and other (Lewis‐acidic) transition metals,^29^ surprisingly few crystal structures of (chiral) [BOX⋅Cu^I^]X complexes are known in the literature;^30^, ^31^, ^32^, ^33^ actually, some of them are intricate dimeric or oligomeric arrays, which likely have to disassemble before any catalytic reaction can take place.

**Figure 4:**
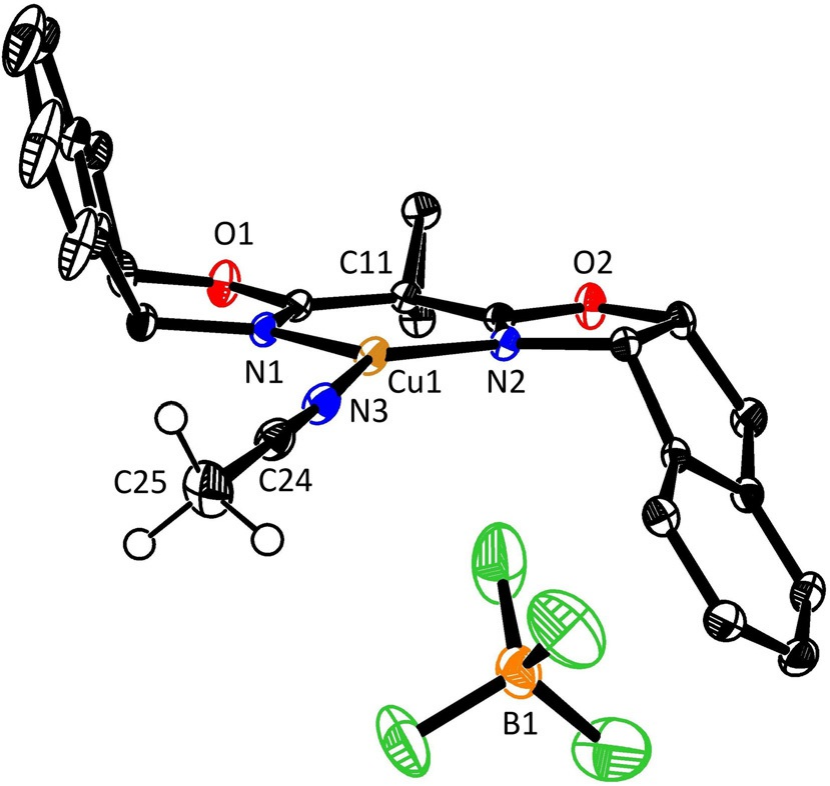
Structure of complex [**L3**⋅Cu(MeCN)]BF_4_ in the solid state; hydrogen atoms on the ligand backbone are omitted for clarity.

In contrast, complex [**L3**⋅Cu(MeCN)]BF_4_ is a monomeric entity (Figure 4); the coordination geometry about the Cu‐center is distorted trigonal. The N1‐Cu1‐N2 bite‐angle of 95.9(1)° is wider than that of the few comparable cases in the literature,^31^ which likely reflects the influence of the *gem*‐disubstitution at the bridge as well as the steric demand of the indane core. Although the crystal structure provides only a static picture, it is noteworthy that the complex is not strictly *C*_2_‐symmetric in the solid state; specifically, the N3‐Cu1‐N1 (121.8(1)°) and the N3‐Cu1‐N2 (141.5°) angles are notably uneven, as are the distances Cu1−N1 (2.004(2) Å) and Cu1−N2 (1.952(2) Å).^31^, ^34^

**Figure 5:**
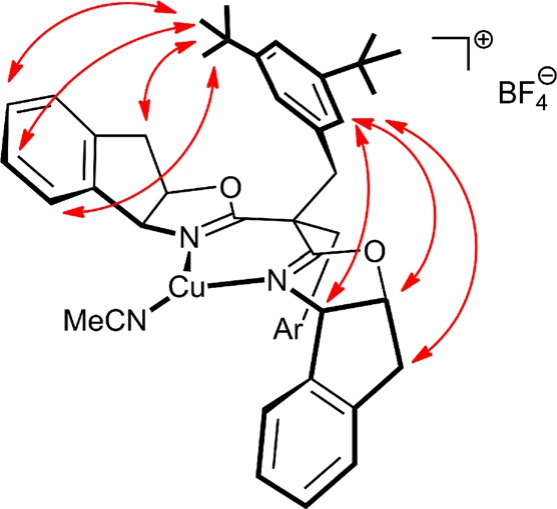
Graphical representation of relevant NOE contacts in [**L11**⋅Cu(MeCN)]BF_4_, which indicate that the substituted benzyl groups are, on average, “forward” oriented on the NMR time scale.

NMR investigations provided some insights into the role of the substituents at the bridge between the oxazoline rings.^34^ NOESY spectra of complex [**L11**⋅Cu(MeCN)]BF_4_ in CDCl_3_ show numerous cross peaks between the protons of the substituted benzyl groups and those of the indane ring (see Figure 5). The benzyl substituents are hence “forward” oriented in at least one averaged conformer on the NMR timescale; as such, they (temporarily) reside in vicinity of the incipient carbene, above and below the catalytically active Cu‐center. The crystal structure of a related BOX⋅Cu complex with pendant *p*‐(*tert*‐butyl)benzyl substituents corroborates the notion that these seemingly remote substituents actually shield the upper and lower face of the coordination plane.^31^, ^35^

**Figure 6:**
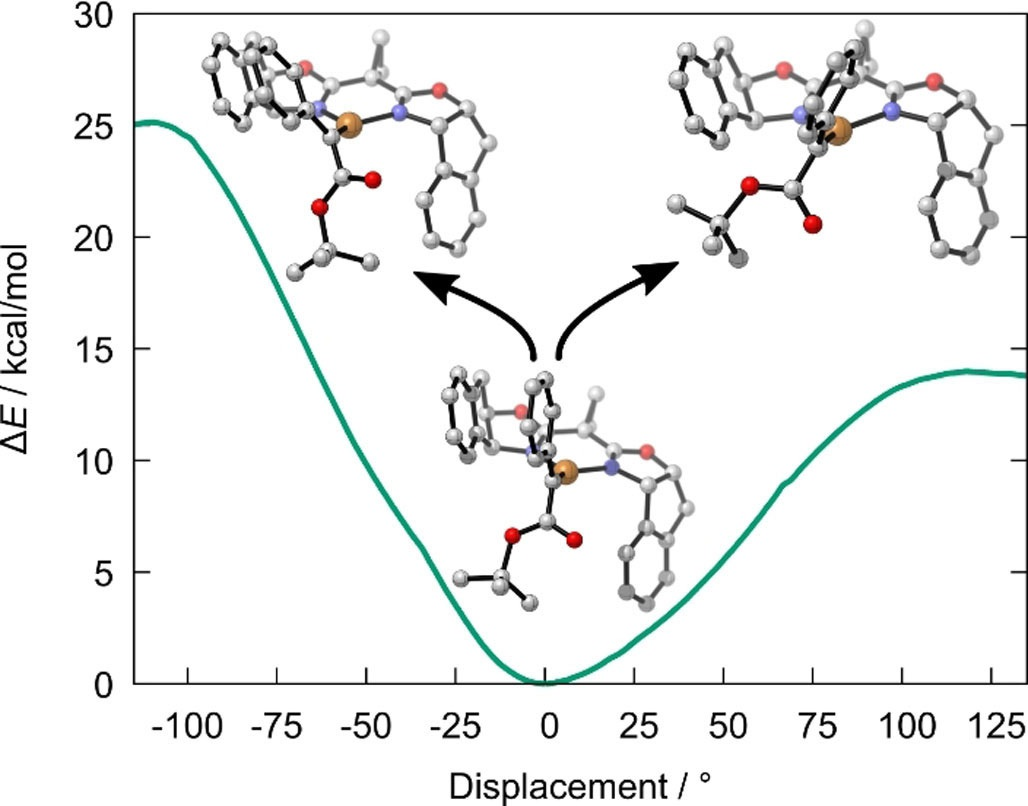
Computed ground state structure of the donor/acceptor copper carbene complex [**L3**⋅Cu=CPh(COO*t*Bu)]BF_4_ formed from [**L3**⋅Cu(MeCN)]BF_4_ and the diazoester **1 b**; illustration of the uneven barriers for clockwise and counterclockwise rotation about the Cu−C bond.

If one takes the ligated acetonitrile as a dummy for the carbene to be formed upon reaction of the Cu‐center with the α‐diazoester, attack onto the *Re* face of the electrophilic C‐atom seems most plausible at first sight, which would entail formation of (*R*)‐**2 b** (compare Figures 5 and 6). Since (*S*)‐**2 b**, however, has been shown to be the major product, a more involved scenario must be operative. Therefore, a computational study was performed to gain a better understanding. Density functionals based on the generalized gradient approximation (GGA) were chosen for the good balance between accuracy and performance. The BP86 functional was found to reproduce the experimental structure of the copper complex [**L3**⋅Cu(MeCN)]BF_4_ particularly well and was therefore used throughout (for the computational methods, see the Supporting Information).

The plane defined by the trigonal donor/acceptor carbene center in [**L3**⋅Cu=CPh(COO*t*Bu)]BF_4_ is orthogonal to the {N_2_Cu} plane of the chiral catalyst (Figure 6);^36^ this arrangement is geometrically favorable and, at the same time, arguably maximizes back‐bonding from the filled metal d‐orbital that is most destabilized by the *N*,*N*‐donor ligand into the empty carbene *p*‐orbital. As one might expect for a *C*_2_‐symmetric ligand environment,^34^ the barriers for clockwise and counter‐clockwise rotation about the Cu−C bond are notably different. The appreciable barrier heights of ≈14 and ≈25 kcal mol^−1^, respectively, imply that the carbene moiety does not freely rotate at ambient temperature at which the reaction with fluoride does occur, but rather oscillates about the conformation of lowest energy. Importantly, this swinging motion is directional and the amplitudes are uneven, in that clockwise movement will prevail by large margins as it avoids a clash between the carbene's substituents and the rigid indane scaffold.^37^

**Figure 7:**
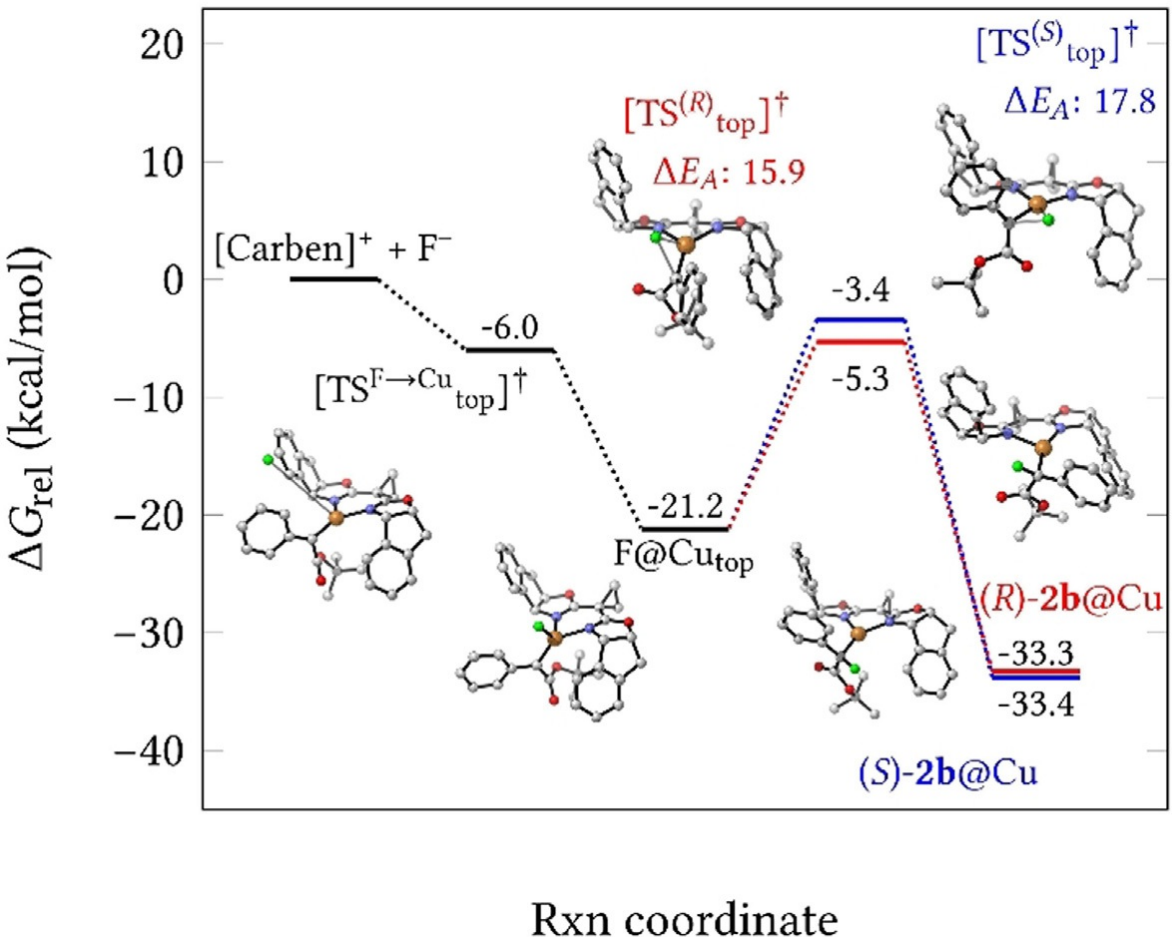
Reaction profile for nucleophilic attack of fluoride from the top‐face of the Cu‐carbene complex [**L3**⋅Cu=CPh(COO*t*Bu)]BF_4_, followed by isomerization of **F@Cu_top_** into (*S*)‐ or (*R*)‐**2 b**; for the free energy profile of the bottom‐face approach, see the Supporting Information.

In exploring possible trajectories for the attack of fluoride onto this complex, we were surprised to find that the Cu‐center rather than the electrophilic carbene C‐atom constitutes the prime site of attack; no transition state was found for direct C−F bond formation, despite considerable search efforts.^38^ The incoming nucleophile approaches the metal center from above or below the coordination plane to form the copper fluoride complexes **F@Cu_top_** and **F@Cu_bot_**, respectively. It is striking that top‐side attack is barrierless with a submerged transition state **TS^F→Cu^**_**top**_,^39^ provided that a continuum solvent environment was used in the computations (Figure 7). In stark contrast, attack from the bottom face shows an activation barrier of no less than 12.3 kcal mol^−1^ (see the Supporting Information). Qualitatively, this differential is rather intuitive and very well in line with the conclusions previously drawn from the solid‐state structure of electrophilic donor/acceptor dirhodium carbenes:^13^ Any incoming nucleophile, especially when charged, will avoid dipole repulsion with the lone‐pairs of the ester O‐atoms^40^ and hence preferentially follow a Bürgi–Dunitz trajectory alongside the aryl substituent of the donor/acceptor carbene.^13^, ^41^

Since the top–face approach wins out by far, only the fate of the resulting complex **F@Cu_top_** needs to be considered at this point. Orbital alignment is necessary before the fluoride ligand can migrate from copper to carbon,^42^, ^43^ which, in turn, mandates that the carbene unit rotates about the Cu−C bond. The transition state for clockwise rotation is 1.9 kcal mol^−1^ lower in energy (Figure 7); it leads to complex (*R*)‐**2 b**@Cu, which ultimately delivers (*S*)‐**2 b** upon protonation (note that the “inversion” is due to the formalism of the CIP rules). Although this computational result overestimates the level of induction, it correctly predicts the sense of induction of the copper catalyzed α‐fluorination reaction.

## Conclusions

The first highly enantioselective metal catalyzed addition of fluoride to α‐diazoesters is described, featuring *ee* values of up to 95 %. To properly assess this result, one has to consider the inherent challenges for asymmetric catalysis posed by this particular nucleophile, which had basically thwarted an earlier attempt at rendering this transformation asymmetric.^9^ Interestingly, the reaction most likely proceeds by initial attack of fluoride onto the Cu‐ rather than the C‐atom of the electrophilic metal carbene intermediate; a subsequent 1,2‐fluoride shift then leads to product formation. For this migration to happen, the carbene must rotate about the Cu−C bond to ensure the necessary orbital overlap; the directionality of this rotatory motion, enacted by the *C*_2_‐symmetric ligand environment, is the major enantiodetermining factor. Further work on the structure and reactivity of transition metal carbene complexes and the stereoselective preparation of organofluorine derivatives^44^ is underway in our laboratory and will be reported in due course.

## Experimental Section

All experimental details can be found in the Supporting Information. The material includes compound characterization data, additional crystallographic information, the full list of ligands investigated, HPLC traces, supporting computational data, and copies of spectra of new compounds.

## Conflict of interest

The authors declare no conflict of interest.

## Supporting information

As a service to our authors and readers, this journal provides supporting information supplied by the authors. Such materials are peer reviewed and may be re‐organized for online delivery, but are not copy‐edited or typeset. Technical support issues arising from supporting information (other than missing files) should be addressed to the authors.

Supplementary
