# Supplementary material for: Catalytic Asymmetric Fluorination of Copper Carbene Complexes: Preparative Advances and a Mechanistic Rationale
Source: Chemistry. 2020 Feb 18;26(11):2509–15. doi: 10.1002/chem.202000081 (PMC7065061; doi:10.1002/chem.202000081)
Supplement: Supplementary file 1 — Supplementary [file CHEM-26-2509-s001.pdf]

CHEMISTRY
A European Journal

Supporting Information

Catalytic Asymmetric Fluorination of Copper Carbene Complexes: Preparative Advances and a Mechanistic Rationale

Michael Buchsteiner,[a] Luis Martinez-Rodriguez,[a] Paul Jerabek,[a, b] Iago Pozo,[a]
Michael Patzer,[a] Nils Nöthling,[a] Christian W. Lehmann,[a] and Alois Fürstner*[a]

chem_202000081_sm_miscellaneous_information.pdf

TABLE OF CONTENTS

GENERAL 2
SUPPORTING CRYSTALLOGRAPHIC INFORMATION 3
NOESY NMR STUDIES OF A CATIONIC COPPER(+1) BOX COMPLEX 9
INITIAL LIGAND SCREENING 11
SCREENING OF FLUORIDE SOURCES 14
OPTIMIZATION OF THE REACTION CONDITIONS 15
REAGENTS AND BUILDING BLOCKS 16
LIGAND SYNTHESES 20
SUBSTRATES 32
DIAZO COMPOUNDS 35
ENANTIOSELECTIVE FLUORINATION 38
SUPPORTING COMPUTATIONAL DATA 45
CARTESIAN COORDINATES 53
NMR SPECTRA 74

GENERAL

Unless stated otherwise, all reactions were carried out under argon atmosphere in flame dried Schlenk glassware. The solvents were purified by distillation over the indicated drying agents under argon: THF, Et_2O (Mg/anthracene), pentane (Na/K), CH_2Cl_2, *tert*-BuOH, C_6F_6 (CaH_2), HFIP (4 Å molecular sieves). MeCN, 1,4-dioxane, pyridine and Et_3N were dried by an absorption solvent purification system based on molecular sieves. Flash chromatography: Merck Geduran silica gel 60 (40 – 63µm).

NMR spectra were recorded on Bruker DPX 300, AV 400, AV 500 or AV III 600 spectrometers in the solvents indicated; chemical shifts are given in ppm relative to TMS, coupling constants (*J*) in Hz. The solvent signals were used as references and the chemical shifts converted to the TMS scale ($CDCl_3$: δ_C = 77.2 ppm; residual $CHCl_3$: δ_H = 7.26 ppm; CD_2Cl_2: δ_C = 54.0 ppm; residual $CHDCl_2$: δ_H = 5.32 ppm; C_6D_6: δ_C = 128.1 ppm; residual C_6HD_5: δ_H = 7.16 ppm). Signal assignments were established using HSQC, HMBC and NOESY experiments.

IR: Alpha Platinum ATR (Bruker), wavenumbers ($\tilde{\nu}$) in cm^{-1}. Signal intensities were assigned according to their relative intensities as strong (s) or medium (m). Most medium and weak (w) resonances were omitted.

MS (EI): Finnigan MAT 8200 (70 eV), ESI-MS: ESQ 3000 (Bruker) or Thermo Scientific LTQ-FT or Thermo Scientific Exactive Spectrometer. HRMS: Bruker APEX III FT-MS (7 T magnet), MAT 95 (Finnigan), Thermo Scientific LTQ-FT or Thermo Scientific Exactive Spectrometer. GC-MS spectra were measured on a Shimadzu GCMS-QP2010 Ultra instrument.

LC analyses were conducted on a Shimadzu LC 2020 instrument equipped with a Shimadzu SPD-M20A UV/VIS detector.

Unless stated otherwise, all commercially available compounds (abcr, Acros, TCI, Aldrich, Alfa Aesar, Fluoro Chem) were used as received. CsF was dried under vacuum at 100°C and stored under argon atmosphere in a Schlenk tube.

SUPPORTING CRYSTALLOGRAPHIC INFORMATION

Complex [**L3**·Cu(MeCN)]BF_4**.** A flame-dried Schlenk tube was charged with [Cu(MeCN)$_4$]BF_4 (157 mg, 0.512 mmol,) and (3a*S*,3a'*S*,8a*R*,8a'*R*)-2,2'-(cyclopropane-1,1-diyl)bis-(3a,8a-dihydro-8*H*-indeno[1,2-*d*]oxazole) **L3** (179 mg, 0.556 mmol). Degassed CH_2Cl_2 (7 mL) was added and the mixture was stirred for 30 min at rt before it was passed through a cannula filter. The solvent was removed in vacuo to obtain the title compound as a colorless solid (271 mg, 99 % yield). ^{1}H NMR (400 MHz, $CDCl_3$): δ = 7.60 (d, *J* = 7.4 Hz, 2H), 7.37 (dt, *J* = 23.8, 7.3 Hz, 4H), 7.27 (d, *J* = 5.5 Hz, 2H), 5.66 (d, *J* = 7.8 Hz, 2H), 5.47 – 5.38 (m, 2H), 3.46 (dd, *J* = 18.3, 6.8 Hz, 2H), 3.18 (d, *J* = 18.2 Hz, 2H), 2.52 (s, 3H), 1.87 – 1.68 (m, 4H); ^{13}C NMR (101 MHz, $CDCl_3$): δ = 168.1, 139.6, 139.2, 129.8, 128.4, 125.6, 84.4, 76.6, 39.3, 20.9, 18.6, 2.9; IR (ATR): ṽ = 1651 (s), 1619 (m), 1427 (s), 1109 (s), 1041 (s), 988 (s), 761 (s), 518 (m) cm^{-1}; HRMS (ESI^+) for $C_{25}H_{23}N_3O_2Cu$ [M – BF_4^-]$^+$: calcd: 460.10807, found: 460.10787. Single crystals suitable for X-ray diffraction analysis were obtained by diffusing pentane into a saturated solution of the complex in CH_2Cl_2 at rt.

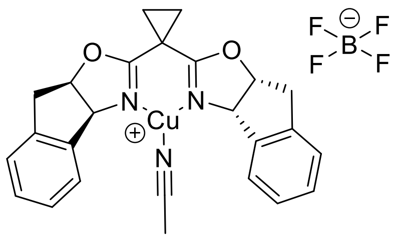

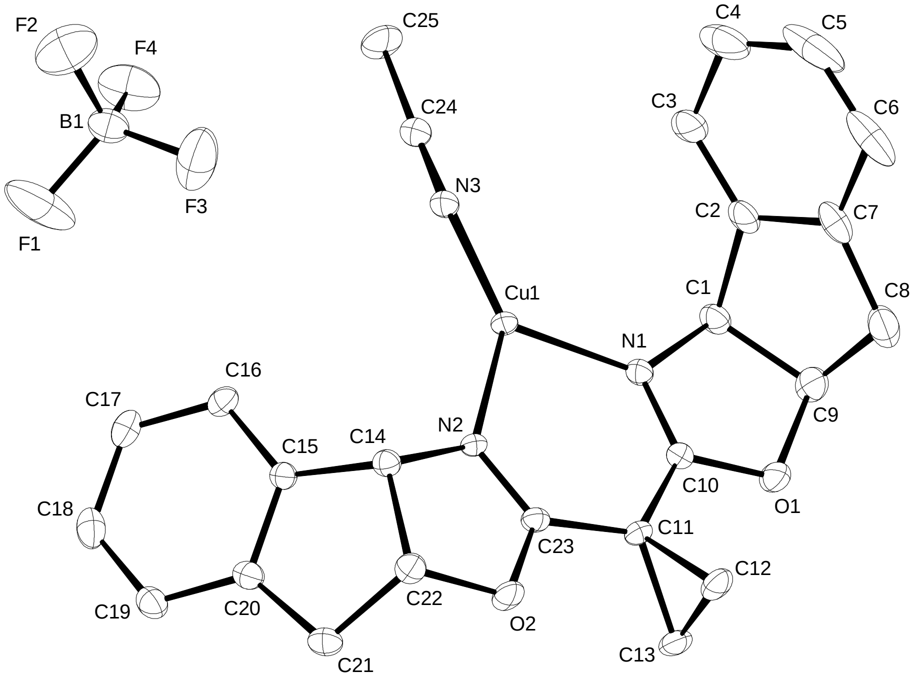

Figure S1. Structure of complex [L3·Cu(MeCN)]BF_4 in the solid state. H atoms have been removed for clarity.

X-ray Crystal Structure Analysis of Complex [L3·Cu(MeCN)]BF$_4$: $C_{25}H_{23}BCuF_4N_3O_2$, M_r = 547.81 g mol^{-1}, colourless plate, crystal size 0.080 x 0.050 x 0.021 mm^3, orthorhombic, space group $P2_12_12_1$ [No. 19], a = 7.4066(9) Å, b = 11.5168(13) Å, c = 27.002(3) Å, V = 2303.3(5) Å^3, T = 100(2) K, Z = 4, D_{calc} = 1.580 g cm^{-3}, λ = 0.71073 Å, $\mu(Mo\text{-}K_\alpha)$ = 1.010 mm^{-1}, analytical absorption correction (T_{min} = 0.94058, T_{max} = 0.98521), Bruker-AXS Kappa Mach3 diffractometer with APEX-II detector and IµS micro focus X-ray source, 2.3244 < θ < 32.7540 °, 68847 measured reflections, 8690 independent reflections, 7733 reflections with $I > 2\sigma(I)$, R_{int} = 0.0536. The structure was solved by dual space methods (*SHELXT*) and refined by full-matrix least-squares (*SHELXL*) against F^2 to R_1 = 0.0349 [$I > 2\sigma(I)$], wR_2 = 0.0781, 326 parameters, absolute structure parameter using Flack's method = 0.010(3).

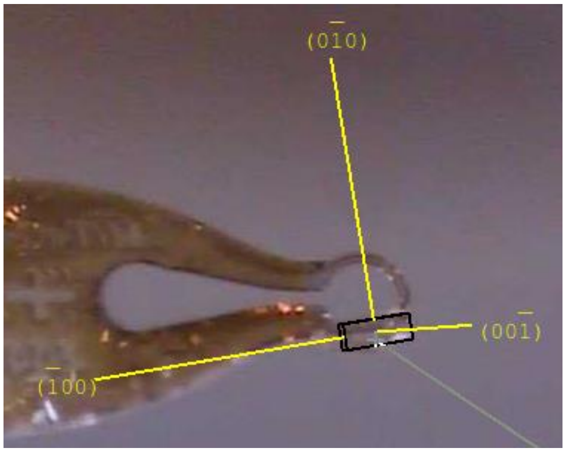

H	K	L	Distance [mm]
1	0	0	0.040
-1	0	0	0.040
0	0	1	0.005
0	0	-1	0.010
0	-1	0	0.025
0	1	0	0.005

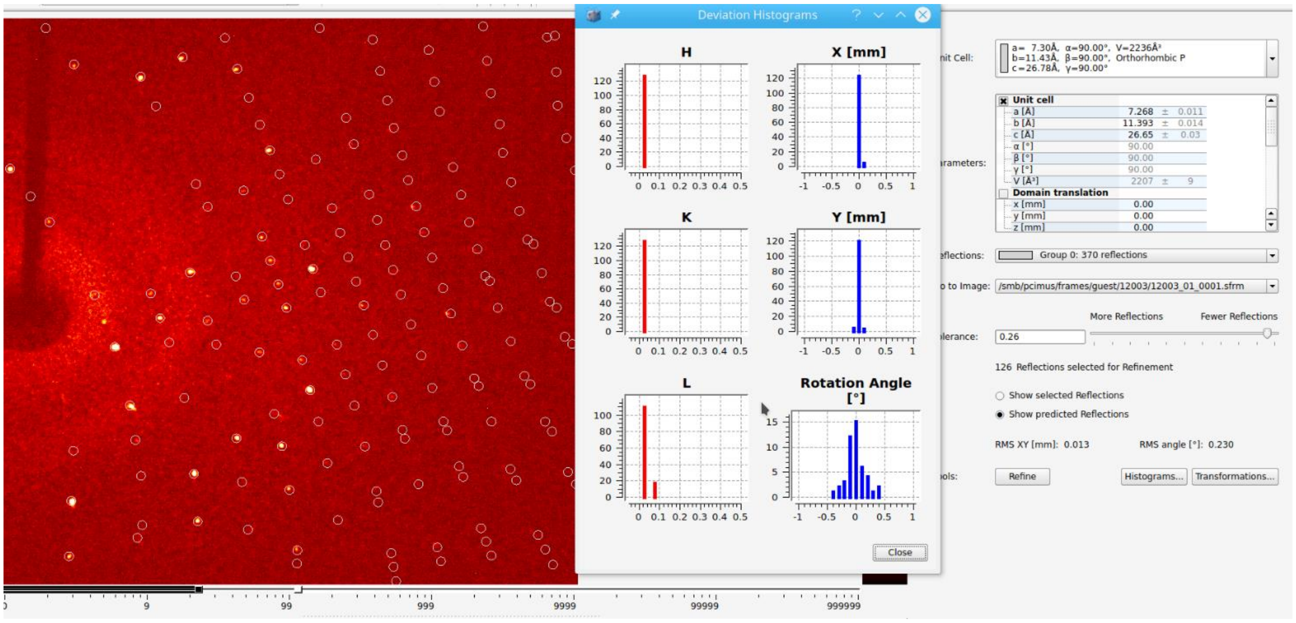

INTENSITY STATISTICS FOR DATASET

Resolution	#Data	#Theory	%Complete	Redundancy	Mean I	Mean I/s	Rmerge	Rsigma
Inf - 2.67	132	135	97.8	10.33	58.13	92.99	0.0169	0.0087
2.67 - 1.78	305	305	100.0	11.41	25.68	82.42	0.0209	0.0097
1.78 - 1.41	445	446	99.8	11.42	19.95	71.21	0.0257	0.0112
1.41 - 1.22	457	457	100.0	11.12	11.59	54.34	0.0367	0.0145
1.22 - 1.12	404	404	100.0	10.43	9.69	45.04	0.0441	0.0176
1.12 - 1.03	486	486	100.0	9.74	7.81	36.91	0.0537	0.0218
1.03 - 0.97	427	427	100.0	9.17	6.29	29.74	0.0642	0.0270
0.97 - 0.92	464	464	100.0	8.54	5.29	24.40	0.0765	0.0331
0.92 - 0.88	452	452	100.0	8.41	4.40	20.76	0.0901	0.0393
0.88 - 0.85	387	387	100.0	7.88	3.92	18.41	0.1027	0.0464
0.85 - 0.82	421	421	100.0	7.60	3.73	16.41	0.1043	0.0502
0.82 - 0.79	549	549	100.0	7.30	2.62	12.25	0.1400	0.0686
0.79 - 0.77	382	382	100.0	7.19	2.43	11.17	0.1536	0.0761
0.77 - 0.75	431	431	100.0	6.72	2.59	10.88	0.1521	0.0794
0.75 - 0.73	482	482	100.0	6.63	2.19	9.39	0.1787	0.0927
0.73 - 0.71	559	559	100.0	6.42	1.96	7.93	0.1972	0.1067
0.71 - 0.70	286	286	100.0	6.04	1.96	7.66	0.1962	0.1142
0.70 - 0.68	623	623	100.0	6.02	1.57	6.23	0.2397	0.1416
0.68 - 0.67	338	338	100.0	5.82	1.60	5.99	0.2528	0.1473
0.67 - 0.66	399	399	100.0	5.77	1.29	5.00	0.2912	0.1812
0.66 - 0.65	296	349	84.8	2.65	1.57	4.20	0.2409	0.2272
0.75 - 0.65	2983	3036	98.3	5.75	1.75	6.80	0.2179	0.1335
Inf - 0.65	8725	8782	99.4	7.86	6.45	24.47	0.0527	0.0321

Complete data of the compound are available under the CCDC number **CCDC- 1966678**.

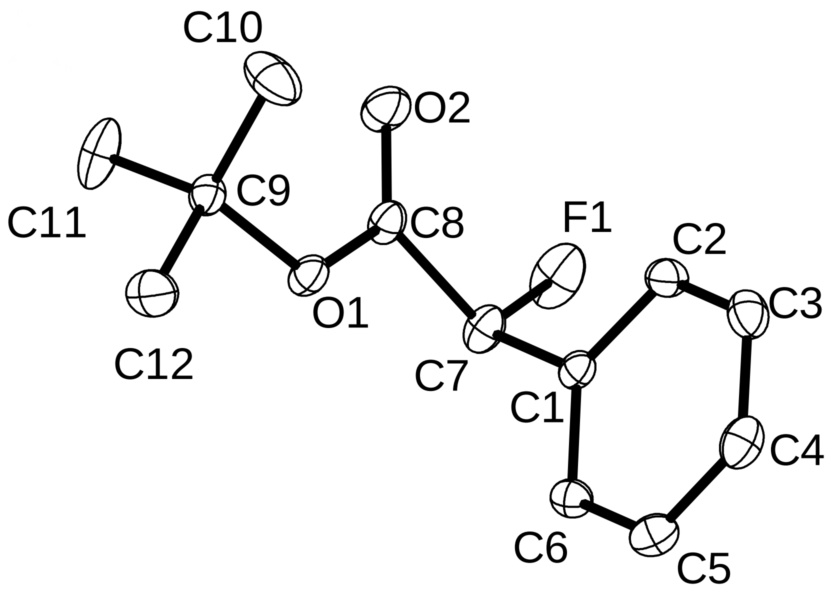

Figure S2. Structure of Ester (*S*)-2b in the solid state. H atoms have been removed for clarity.

X-ray Crystal Structure Analysis of Compound (*S*)-2b: C_{12} H_{15} F O_2, M_r = 210.24 g mol^{-1}, colourless cylinder/capillary, estimated crystal size 1.078 x 0.392 x 0.387 mm^3, orthorhombic, space group $P2_12_12_1$ [No. 19], a = 5.9437(3) Å, b = 8.9653(4) Å, c = 20.9960(10) Å, V = 1118.82(9) Å^3, T = 100(2) K, Z = 4, D_{calc} = 1.248 g · cm^{-3}, λ = 0.71073 Å, μ(Mo-K_α) = 0.094 mm^{-1}, analytical absorption correction (T_{min} = 0.93924, T_{max} = 0.97525), Bruker-AXS Kappa Mach3 diffractometer with APEX-II detector and IµS micro focus X-ray source, 3.563 < θ < 41.876 °, 74385 measured reflections, 7611 independent reflections, 6582 reflections with $I > 2\sigma(I)$, R_{int} = 0.0354. The structure was solved by dual space methods (*SHELXT*) and refined by full-matrix least-squares (*SHELXL*) against F^2 to R_1 = 0.0375 [$I > 2\sigma(I)$], wR_2 = 0.0989, 139 parameters, absolute structure parameter using Flack´s-Method = -0.08(10).

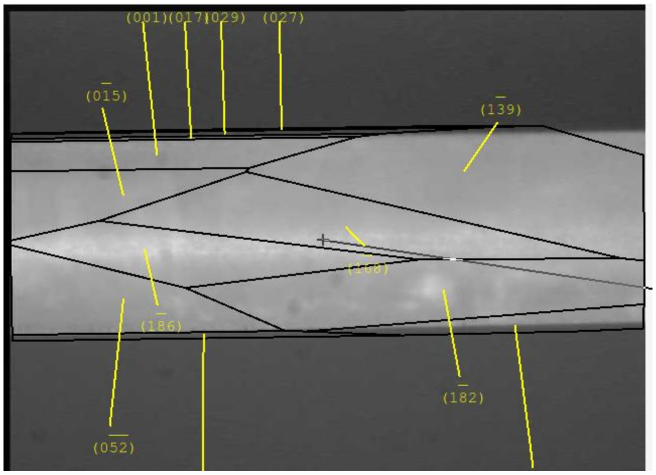

H	K	L	Distance [mm]
0	9	4	0.174
0	-5	-2	0.189
0	-1	-7	0.169
0	1	7	0.207
0	-2	-9	0.163
0	2	9	0.195
0	-2	-7	0.162
0	2	7	0.190
0	-3	-7	0.170
1	-9	-7	0.182
-1	9	7	0.213
6	1	-2	0.528
-8	-1	2	0.515
1	-8	2	0.162
-1	9	-2	0.197
1	-8	6	0.165
-1	8	-6	0.195
1	-6	8	0.169
-1	6	-8	0.196
-1	4	-9	0.211
1	-3	9	0.193
-1	3	-9	0.227
1	6	9	0.236
0	5	8	0.190
0	-1	5	0.240
0	1	-6	0.209
0	0	1	0.221
0	0	-1	0.192

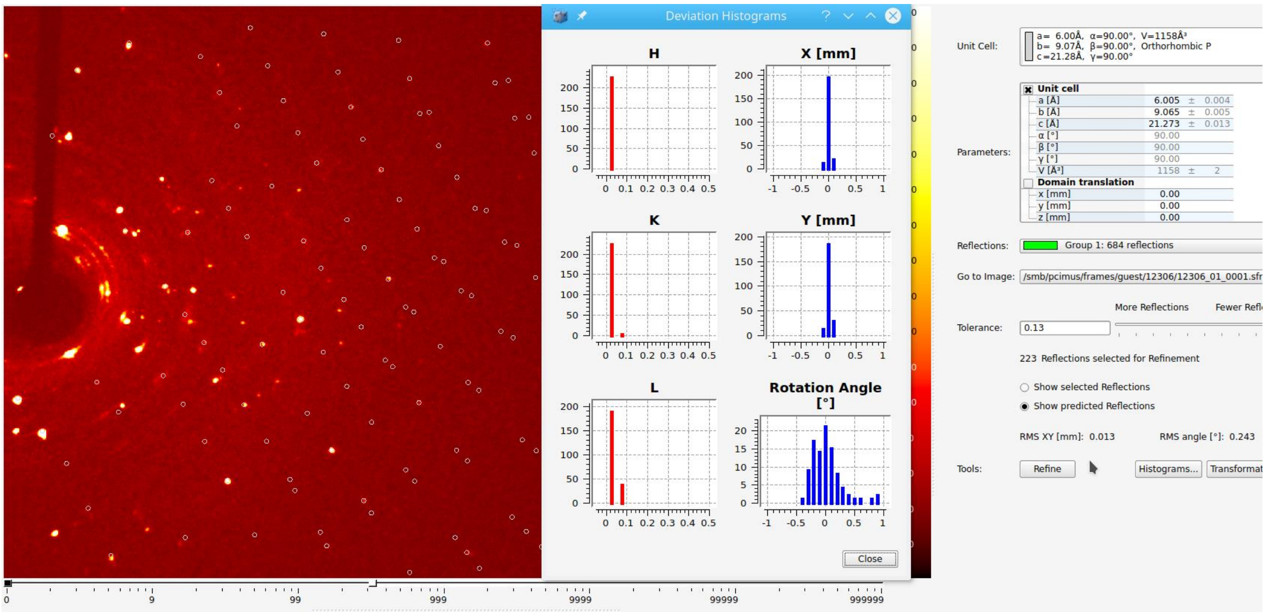

Resolution	#Data	#Theory	%Complete	Redundancy	Mean I	Mean I/s	Rmerge	Rsigma
Inf - 2.19	117	119	98.3	20.97	91.81	125.75	0.0283	0.0073
2.19 - 1.46	268	268	100.0	23.79	28.08	121.00	0.0245	0.0071
1.46 - 1.15	397	397	100.0	23.52	13.31	99.18	0.0262	0.0081
1.15 - 1.01	367	367	100.0	19.02	11.40	85.50	0.0290	0.0096
1.01 - 0.92	383	383	100.0	14.31	5.57	55.26	0.0414	0.0143
0.92 - 0.85	394	394	100.0	12.40	3.52	39.79	0.0549	0.0194
0.85 - 0.80	398	398	100.0	11.36	2.79	32.48	0.0679	0.0244
0.80 - 0.76	370	370	100.0	10.93	2.61	29.35	0.0747	0.0272
0.76 - 0.72	475	475	100.0	10.04	2.42	25.74	0.0818	0.0311
0.72 - 0.70	279	279	100.0	9.85	2.47	25.27	0.0903	0.0321
0.70 - 0.67	463	463	100.0	9.25	1.80	19.32	0.1015	0.0420
0.67 - 0.65	386	386	100.0	8.62	1.60	17.01	0.1172	0.0492
0.65 - 0.63	419	419	100.0	8.36	1.21	13.57	0.1431	0.0626
0.63 - 0.61	476	476	100.0	8.17	1.24	13.24	0.1465	0.0645
0.61 - 0.60	240	240	100.0	7.66	0.96	10.56	0.1827	0.0845
0.60 - 0.58	611	618	98.9	3.77	0.91	7.27	0.1736	0.1246
0.58 - 0.57	312	312	100.0	2.56	0.69	5.25	0.1692	0.1763
0.57 - 0.56	348	348	100.0	2.59	0.72	5.52	0.1647	0.1710
0.56 - 0.55	361	361	100.0	2.48	0.65	4.69	0.1867	0.2021
0.55 - 0.54	386	388	99.5	2.40	0.54	3.96	0.2292	0.2452
0.54 - 0.53	195	329	59.3	0.94	0.34	1.89	0.2815	0.5720
0.63 - 0.53	2929	3072	95.3	3.87	0.80	6.97	0.1663	0.1461
Inf - 0.53	7645	7790	98.1	9.58	5.16	31.32	0.0352	0.0208

The low-angle reflection [0 0 2] was shadowed by the beamstop and has been removed from the data set before the final refinement cycles. The crystal was grown from the liquid in a long glass capillary. The maximum crystal size was estimated based on the diffraction geometry. For data acquisition, a fixed chi angle of 54.7 degree was chosen to ensure that the exposed crystalline volume was as small as possible consistent with a high redundancy. Complete data of the compound are available under the CCDC number **CCDC- 1966679**.

***tert*-Butyl (*S*)-2-fluoro-2-phenylacetate ((*S*)-2b).** For the preparation and the analytical data, see below.

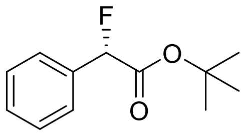

A sample (89% *ee*) was enriched in major isomer (≥ 99% *ee*) by preparative HPLC using the following conditions: YMC-Chiralart SB 5 µm, hexane/*iso*-propanol = 80/20, 20 mL/min, 100 mg sample in *iso*-propanol (c = 50 mg/mL), 39 runs, 2.5 mg sample per run. Single crystals suitable for X-ray diffraction analysis were obtained by capillary crystallization.

Results of ambient to low temperature Differential scanning calorimetry of tert-Butyl (*S*)-2-fluoro-2-phenylacetate, to determine the phase transition temperatures and possible polymorphism. Experiments where performed on a METTLER TOLEDO DSC 820 measuring module for thermal analysis. Two cycles with different cooling and heating rates where used for the probe.

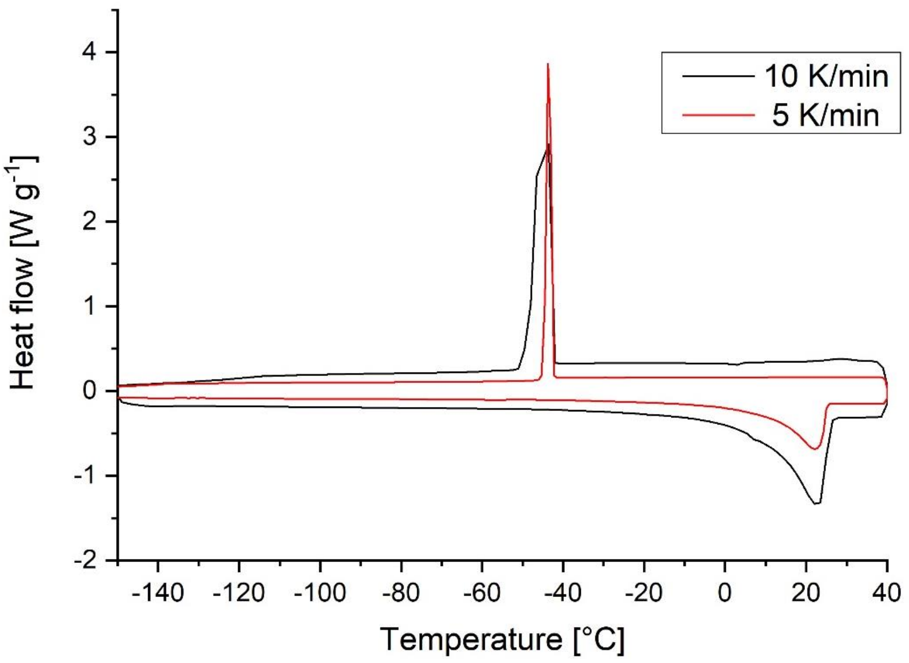

Figure S3. Recorded DSC curve of ester (*S*)-**2b**

NOESY NMR STUDIES OF A CATIONIC COPPER(+1) BOX COMPLEX

Complex [L11·Cu(MeCN)]BF_4. A flame-dried Schlenk tube was charged with $[Cu(MeCN)_4]BF_4$ (98.9 mg, 0.311 mmol) and **L11** (238 mg, 0.320 mmol). CH_2Cl_2 (5 mL) was added and the mixture was stirred at rt for 30 min. The mixture was filtered by cannula filtration and the solvent was evaporated in vacuo to obtain the title compound as a colorless solid (290 mg, 99 % yield). ^{1}H NMR (400 MHz, $CDCl_3$): δ = 7.51 (d, *J* = 7.7 Hz, 2H), 7.40 (t, *J* = 7.4 Hz, 2H), 7.32 (t, *J* = 7.4 Hz, 2H), 7.23 – 7.17 (m, 4H), 6.66 (d, *J* = 1.6 Hz, 4H), 5.36 – 5.29 (m, 2H), 5.15 (d, *J* = 8.8 Hz, 2H), 3.60 – 3.45 (m, 4H), 3.35 (d, *J* = 14.3 Hz, 2H), 3.15 (d, *J* = 19.7 Hz, 2H), 2.49 (s, 3H), 1.08 (s, 36H); ^{13}C NMR (101 MHz, $CDCl_3$): δ = 169.6, 151.3, 138.9, 138.1, 133.8, 130.4, 128.8, 126.3, 125.7, 123.0, 121.6, 83.4, 76.8, 50.8, 45.5, 39.6, 34.7, 31.5, 3.0; IR (ATR) ṽ 2953 (m), 1645 (m), 1362 (m), 1199 (m), 1041 (s), 755 (m), 711 (m), 519 (w) cm^{-1}; HRMS (ESI$^+$) for $C_{53}H_{65}CuN_3O_2$ $[M - BF_4^-]^+$: calcd: 838.4367, found: 838.43659.

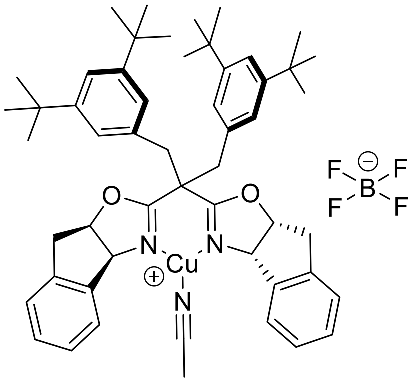

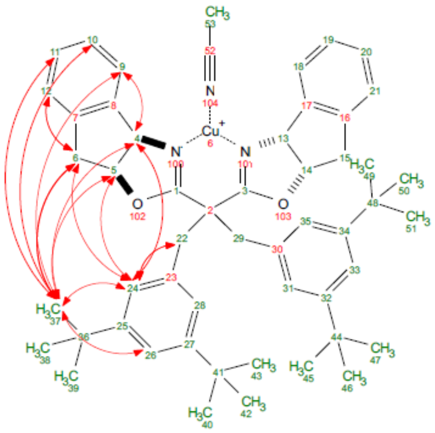

Figure S4. Observed NOE correlations of complex [**L11**·Cu(MeCN)]BF_4

NOEs between the *tert*-butyl groups and the indane backbone indicate the presence of a "closed" conformation, where the 3,5-di-*tert*-butyl groups shield one half space each. It has to be kept in mind, however, that NMR averages over all the conformations that are equilibrating faster than NMR timescale. The recorded number of signals shows that the structure is C_2 symmetric in solution. The rotation about the C22-C23 is faster than the NMR timescale because H24/H28 and H31/H35 are magnetically equivalent.

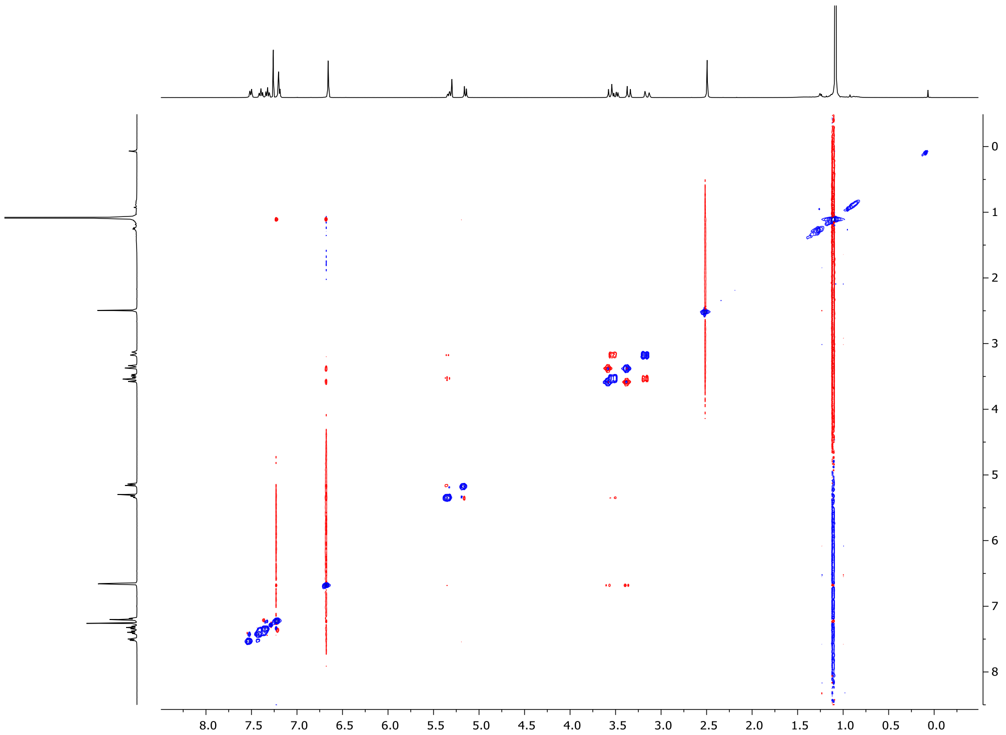

Figure S5. NOESY NMR spectrum of complex [**L11**·Cu(MeCN)]BF_4

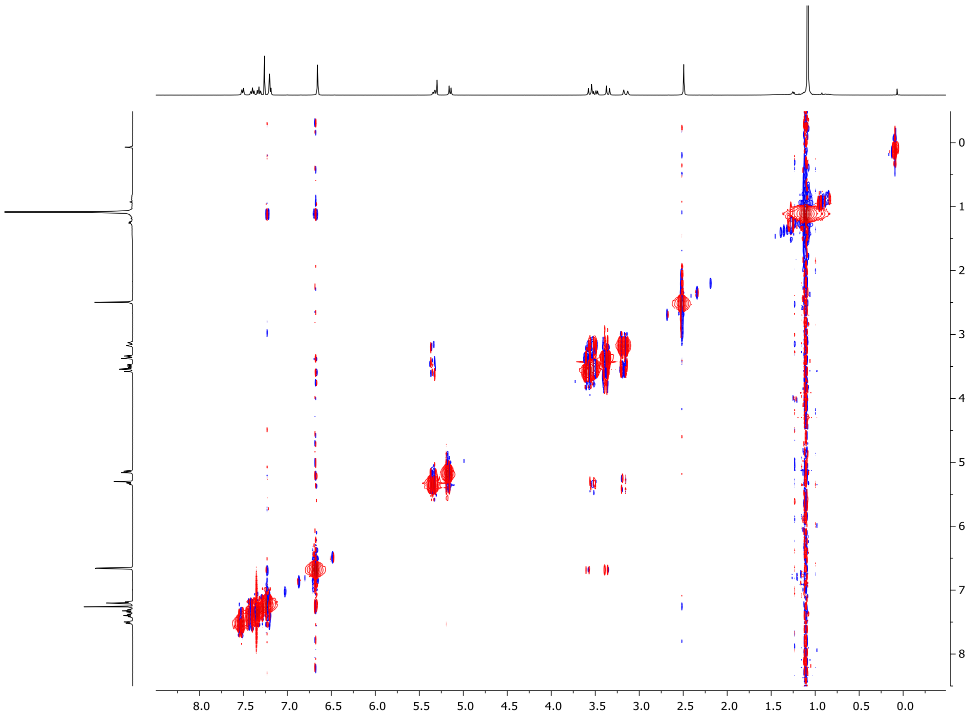

Figure S6. ROESY NMR spectrum of complex [**L11**·Cu(MeCN)]BF_4

INITIAL LIGAND SCREENING

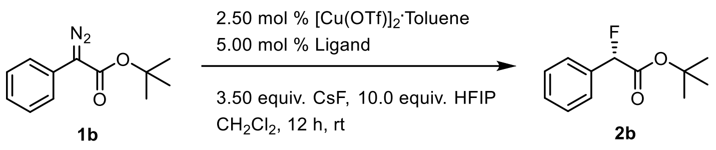

The use of $[Rh_2(S\text{-PTTL})_4]$ (instead of Cu(+1)/L) as the catalyst furnished only trace amounts of product; the ee was not determined

Figure S7. Ligand screening: the tested ligands are roughly custered according to the induced enantioselectivity (note, however, the greatly varying yields); all reactions were performed on a 0.150 mmol scale according to the representative procedure (see below) using CH_2Cl_2 (instead of C_6F_6); NMR yields are given relative to mesitylene serving as internal standard; data marked * refer to the methyl ester **2a** instead of *tert*-butyl ester **2b**

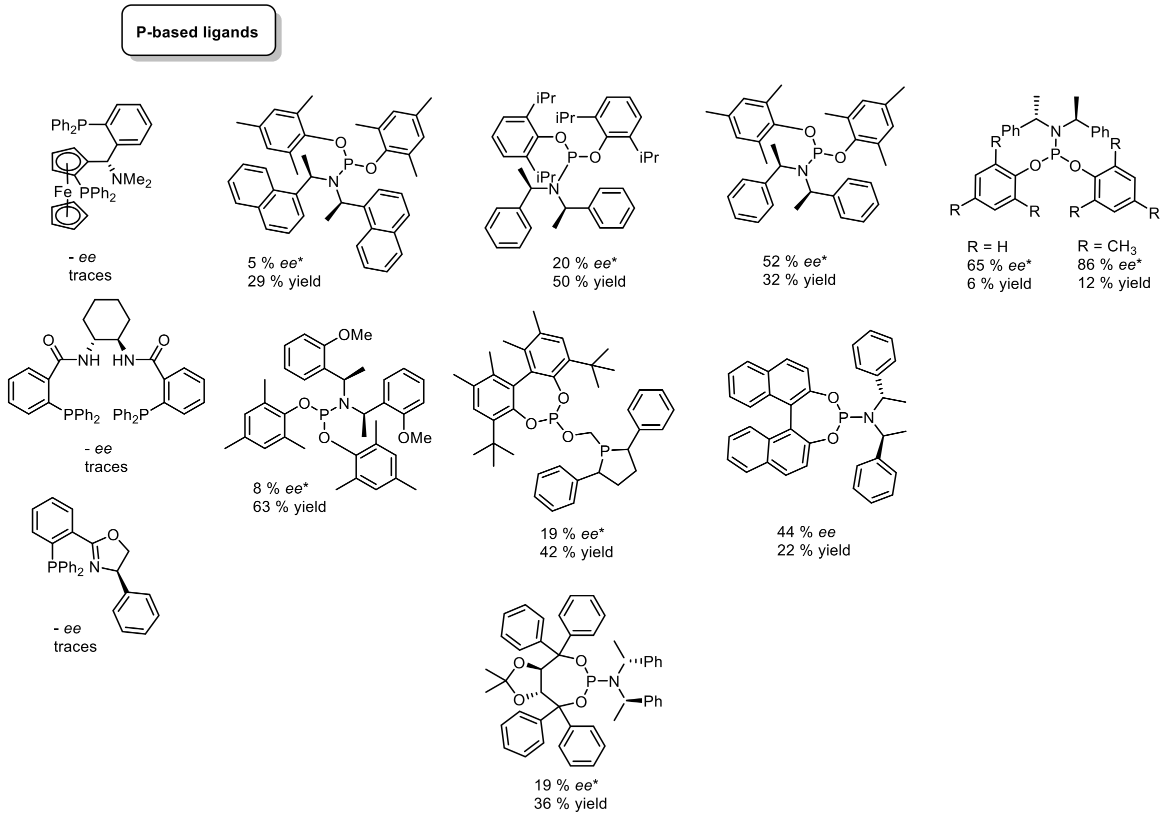

Oxazoline-based Ligands

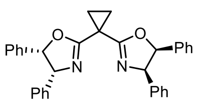

-1,9 % *ee*
60 % yield

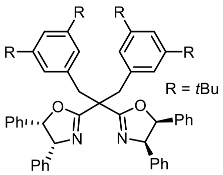

-26,9 % *ee*
46 % yield

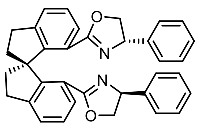

44 % *ee**
42 % yield

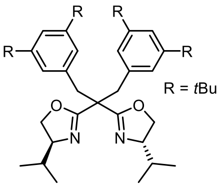

50 % *ee*
87 % yield

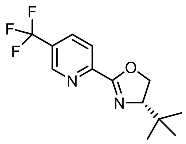

75 % *ee**
3 % yield

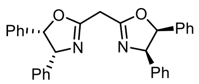

-5,08 % *ee*
61 % yield

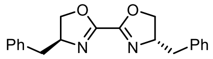

7 % *ee**
25 % yield

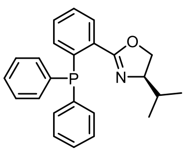

21 % *ee**
56 % yield

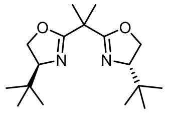

31 % *ee*
62 % yield

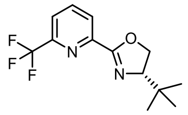

50 % *ee**
3 % yield

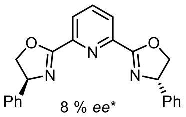

8 % *ee**
24 % yield

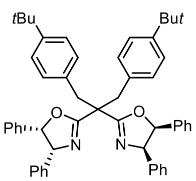

-14,1 % *ee*
49 % yield

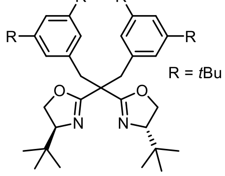

30 % *ee*
81 % yield

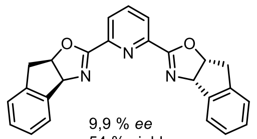

9,9 % *ee*
54 % yield

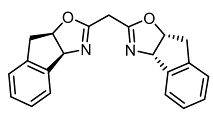

L2
35 % *ee*
71 % yield

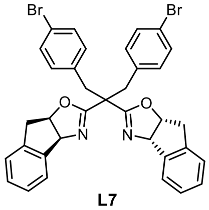

L7
59 % *ee*
61 % yield

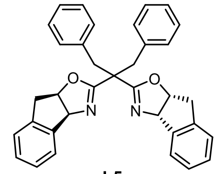

L5
66 % *ee*
90 % yield

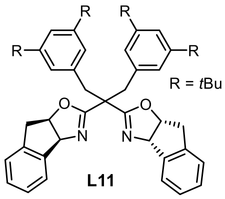

L11
73 % *ee*
84 % yield

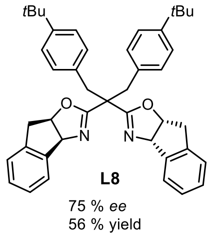

L8
75 % *ee*
56 % yield

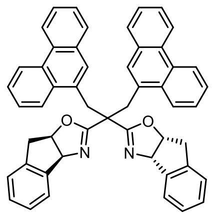

40 % *ee**
80 % yield

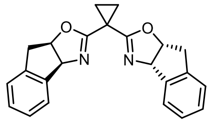

L3
54 % *ee*
88 % yield

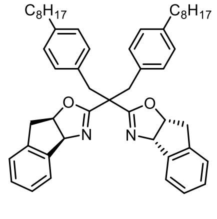

66 % *ee*
72 % yield

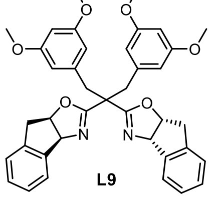

L9
71 % *ee*
87 % yield

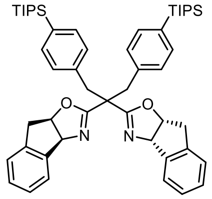

76 % *ee*
81 % yield

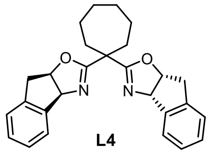

L4
45 % *ee*
54 % yield

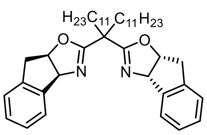

51 % *ee*
66 % yield

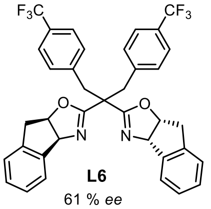

L6
61 % *ee*
91 % yield

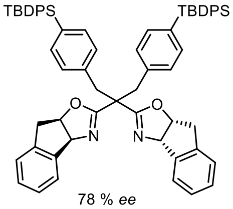

78 % *ee*
75 % yield

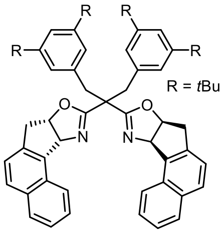

ent-**L10** -16 % *ee* traces

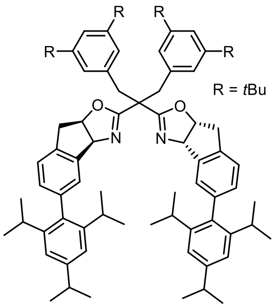

L15 52 % *ee* 54 % yield

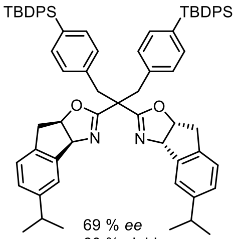

69 % *ee*
60 % yield

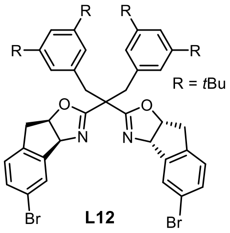

L12
76 % *ee*
75 % yield

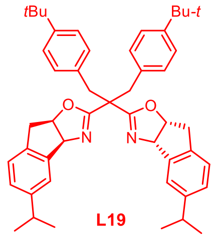

L19
85 % *ee* (89% ee in C_6F_6)
90 % yield

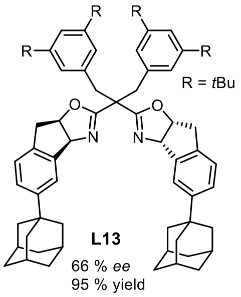

L13
66 % *ee*
95 % yield

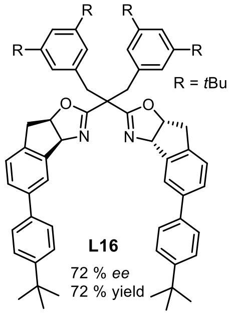

L16
72 % *ee*
72 % yield

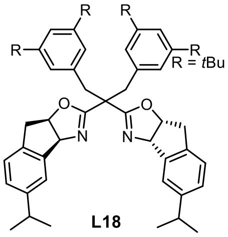

L18
81 % *ee*
66 % yield

L14 71 % *ee* 61 % yield

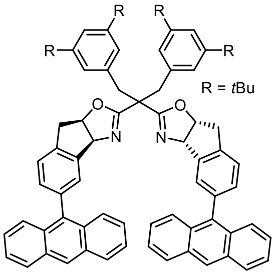

L17 70 % *ee* 55 % yield

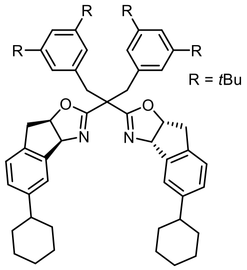

SCREENING OF FLUORIDE SOURCES

Table S1. Screening of Different Fluoride Sources.[a]

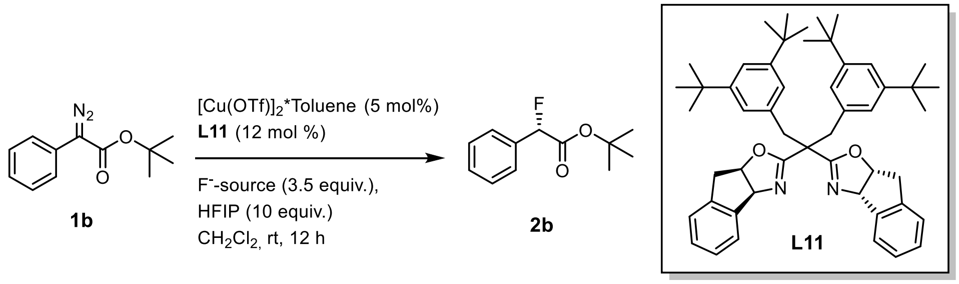

Entry	Fluoride source	*ee* [%]	Yield (NMR) [%][b]
1	SnF_2	-	-
2	CsF	70	79
3	NaF	-	-
4	FeF_3	-	-
5	$LiBF_4$	-	-
6	TiF_4	-	-
7	CoF_3	-	-
8	KHF_2	65	36
9	AgF	n.d.	traces
10	AgF_2	n.d.	traces
11	DMPU/HF	n.d.	traces
12	CaF_2	-	-
13	Phenofluor	-	-
14	DAST	-	-
15	CsF/Pyfluor	n.d.	traces
16	TASF	51	43
17	Pyridine/HF	-	-
18	TBAF	10	46

[a] All reactions were performed according to representative procedure for fluorination reactions using CH_2Cl_2 instead of C_6F_6 with 0.150 mmol of the diazo compound **1b**. [b] NMR yields relative to mesitylene are given.

OPTIMIZATION OF THE REACTION CONDITIONS

Table S2. Optimization of the Reaction Conditions.[a]

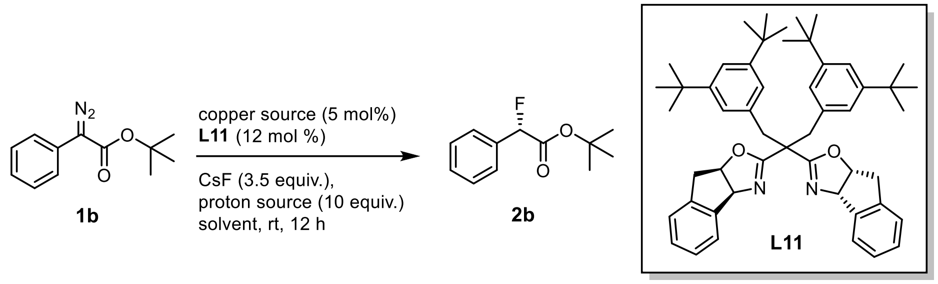

Entry	Cu source	Solvent	Proton source	Yield (NMR)[b] [%]	*ee* [%]
1	$[Cu(OTf)]_2$·Tol	CH_2Cl_2	HFIP	63	64
2	$[Cu(OTf)]_2$·Tol	C_6F_6	HFIP	53	68
3	$[Cu(OTf)]_2$·Tol	CH_2Cl_2	HFIP	83[c]	70
4	$[Cu(OTf)]_2$·Tol	CH_2Cl_2	HFIP	81[c,d]	71
5	$[Cu(OTf)]_2$·Tol	CH_2Cl_2	HFIP	20[c]	40
6	$[Cu(OTf)]_2$·Tol	CH_2Cl_2	*tert*-BuOH	- [c]	-
7	$[Cu(OTf)]_2$·Tol	CH_2Cl_2	*tert*-BuOH	Traces[c,e]	n.d.
8	$[Cu(OTf)]_2$·Tol	CH_2Cl_2	MeOH	Traces[c]	n.d.
9	$[Cu(OTf)]_2$·Tol	CH_2Cl_2/HFIP	HFIP	80[c]	58
10	$[Cu(OTf)]_2$·Tol	CH_2Cl_2	HFIP	25[c,f]	66
11	$[Cu(OTf)]_2$·Tol	CH_2Cl_2	HFIP	Traces[c,g,k]	n.d.
12	$[Cu(OTf)]_2$·Tol	MeCN	HFIP	31[c]	40
13	CuCl/NaBARF	CH_2Cl_2	HFIP	64[c]	69
14	CuBr/$AgSbF_6$	CH_2Cl_2	HFIP	71[c]	68
15	$CuPF_6(MeCN)_4$	CH_2Cl_2	HFIP	75[c]	46
16	$[Cu(OTf)]_2$·Tol	CH_2Cl_2	HFIP	82[c,h]	70
17	$[Cu(OTf)]_2$·Tol	CH_2Cl_2	HFIP	84[c,i]	70
18	$[Cu(OTf)]_2$·Tol	CH_2Cl_2	HFIP	Traces[c,k]	n.d.
19	$[Cu(OTf)]_2$·Tol	THF	HFIP	- [c]	-
20	$[Cu(OTf)]_2$·Tol	THF	HFIP	- [c,k]	-
21	$[Cu(OTf)]_2$·Tol	CH_2Cl_2	CF_3CH_2OH	69[c,l]	20
22	$[Cu(OTf)]_2$·Tol	CH_2Cl_2	HFIP	Traces[m]	n.d.
23	[**L11**·Cu(MeCN)]BF_4 [n]	CH_2Cl_2	HFIP	82[n]	71

[a] All reactions were performed according to representative procedure for fluorination reactions (see below) but using CH_2Cl_2 instead of C_6F_6; [b] NMR yields relative to mesitylene are given; [c] 5 mL of the solvent instead of 1.5 mL; [d] fresh batch of copper source; [e] additional 18-crown-6 ether (1 equiv.) was added; [f] 1.5 equiv. of HFIP instead of 10 equiv.; [g] additional perfluoro-*tert*-BuOH (10 equiv.) was added; [h] 5 h instead of 12 h reaction time; [i] 1 h instead of 12 h reaction time; [j] performed at 10 °C instead of rt; [k] at 0 °C instead of rt; [l] at –10 °C instead of rt; [m] no ligand; [n] using the preformed complex

REAGENTS AND BUILDING BLOCKS

[Cu(O*tBu*)]$_4$. The compound was prepared according to a literature procedure under rigorously inert conditions.[1] Copper (I) chloride (933 mg, 9.43 mmol) and sodium *tert*-butanolate (955 mg, 9.94 mmol) were suspended in freshly distilled THF (30 mL). The resulting brownish suspension was stirred at rt for 3 h. The solvent was evaporated in vacuo and the residue was purified by sublimation (170 °C, 10^{-3} mbar) to obtain the title compound as a yellow solid (556 mg, 43 % yield). ^{1}H NMR (400 MHz, C_6D_6): δ = 1.30 (s, 9H); ^{13}C NMR (101 MHz, C_6D_6): δ = 72.68, 35.83; HRMS (EI) for $C_{16}H_{36}O_4Cu_4$ [M]$^+$: calcd: 543.97918, found: 543.97958.

4-Acetamidobenzenesulfonyl azide. The compound was prepared according to a literature procedure.[2] ^{1}H NMR (400 MHz, $CDCl_3$): δ = 7.92 – 7.87 (m, 2H), 7.79 – 7.75 (m, 2H), 7.68 (s, 1H), 2.24 (s, 3H); ^{13}C NMR (101 MHz, $CDCl_3$): δ = 168.9, 144.0, 132.8, 129.2, 119.7, 25.0.

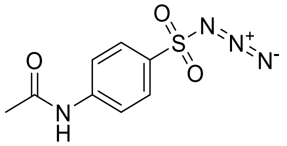

2-Nitrobenzenesulfonyl azide. The compound was prepared according to a literature procedure.[3] ^{1}H NMR (400 MHz, $CDCl_3$): δ = 8.23 – 8.17 (m, 1H), 7.95 – 7.80 (m, 3H). ^{13}C NMR (101 MHz, $CDCl_3$): δ = 147.9, 135.9, 133.2, 132.8, 131.9, 125.5.

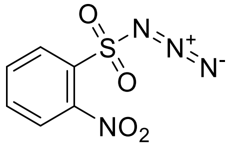

(4-(*tert*-Butyldiphenylsilyl)phenyl)methanol. Methyllithium (1.6 M in hexanes, 1.70 mL, 2.72 mmol) was added dropwise to a solution of (4-bromophenyl)methanol (516 mg, 2.81 mmol) in Et_2O (20 mL) at –78 °C. After stirring for 15 min, *tert*-butyllithium (1.7 M in hexanes, 3.20 mL, 5.44 mmol) was added dropwise. The mixture was diluted with THF (10 mL). After stirring for 20 min at this temperature, TBDPSCl (0.70 mL, 2.69 mmol) was added via syringe and the resulting mixture was warmed to rt and stirred for 18 h. Water (20 mL) was added and the mixture was extracted with CH_2Cl_2 (3 x, 20 mL), the combined organic layers were dried over $MgSO_4$ and concentrated to yield a greyish oil, which was purified by flash chromatography (silica, hexanes/EtOAc = 6/1) to yield the title compound as a colorless oil (621 mg, 65 % yield). ^{1}H NMR (400 MHz, $CDCl_3$): δ = 7.63 – 7.55 (m, 6H), 7.45 – 7.33 (m, 8H), 4.72 (s, 2H), 1.78 (s, 1H), 1.20 (s, 9H); ^{13}C NMR (101 MHz, $CDCl_3$): δ = 141.9, 136.9, 136.6, 134.9, 134.3, 129.3, 127.8, 126.3, 65.4, 28.9, 18.9; ^{29}Si NMR (99 MHz, $CDCl_3$): δ = –6.2; IR (ATR): ṽ = 3292 (m), 2929 (m), 1426 (m), 1103 (s), 1009 (m), 818 (m), 683 (s), 653 (m), 507 (s) cm^{-1}; HRMS (ESI$^+$) for $C_{23}H_{26}OSiNa$ [M + Na$^+$]$^+$: calcd: 369.16451, found: 369.16436.

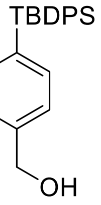

---

1 T. Tsuda, T. Hashimoto, T. Saegusa, *J. Am. Chem. Soc.* **1972**, *94*, 658.
2 H. M. L. Davies, W. R. Cantrell, K. R. Romines, J. S. Baum, *Org. Synth.* **1992**, *70*, 93.
3 B. H. Brodsky, J. Du Bois, *Org. Lett.* **2004**, *6*, 2619.

(4-(Triisopropylsilyl)phenyl)methanol. Prepared analogously as colorless oil (666 mg, 58 % yield). ^{1}H NMR

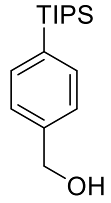

(400 MHz, $CDCl_3$): δ = 7.53 – 7.48 (m, 2H), 7.39 – 7.32 (m, 2H), 4.70 (d, *J* = 5.0 Hz, 2H), 1.73 – 1.67 (m, 1H), 1.41 (hept, *J* = 7.5 Hz, 2H), 1.07 (d, *J* = 7.4 Hz, 18H); ^{13}C NMR (101 MHz, $CDCl_3$): δ = 141.2, 135.7, 134.4, 126.2, 65.6, 18.7, 10.9; ^{29}Si NMR (99 MHz, $CDCl_3$): δ = 1.7. IR (ATR): ṽ = 3274 (m), 2941 (m), 2863 (m), 1460 (m), 1100 (m), 1014 (s), 882 (s), 656 (s), 634 (s), 570 (m), 511 (m) cm^{-1}; HRMS (ESI$^+$) for $C_{16}H_{29}OSi$ [M + H$^+$]$^+$: calcd: 265.19822, found: 265.19826.

(4-(Bromomethyl)phenyl)(*tert*-butyl)diphenylsilane. A flame-dried Schlenk tube was charged with (4-

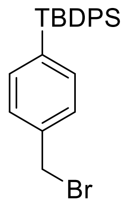

(*tert*-butyldiphenylsilyl)phenyl)methanol (286 mg, 0.830 mmol), CBr_4 (286 mg,, 0.863 mmol) and triphenylphosphine (221 mg, 0.843 mmol). The mixture was cooled to 0 °C and dry CH_2Cl_2 (7 mL) was added. After stirring for 2.5 h, the mixture was concentrated and the remaining colorless oil was triturated with EtOAc (5 mL). The resulting suspension was filtered through a plug of Celite which was washed with EtOAc. The combined filtrates were concentrated and the residue purified by flash chromatography (silica, hexanes/EtOAc = 20/1) to give the title compound as a colorless solid (239 mg, 71 % yield). ^{1}H NMR (400 MHz, $CDCl_3$): δ = 7.60 – 7.54 (m, 6H), 7.44 – 7.33 (m, 8H), 4.51 (s, 2H), 1.18 (s, 9H); ^{13}C NMR (101 MHz, $CDCl_3$): δ = 138.6, 137.1, 136.6, 135.7, 134.6, 129.4, 128.3, 127.9, 33.6, 28.9, 18.9; ^{29}Si NMR (99 MHz, $CDCl_3$): δ = –6.2; IR (ATR): ṽ = 2929 (m), 1426 (m), 1105 (s), 820 (m), 738 (m), 697 (s), 640 (s), 610 (s), 512 (s), 491 (s), 468 (m) cm^{-1}.

(4-(Bromomethyl)phenyl)triisopropylsilane. Prepared analogously as colorless solid (746 mg, 99 %). ^{1}H

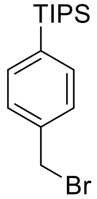

NMR (400 MHz, $CDCl_3$): δ = 7.50 – 7.44 (m, 2H), 7.40 – 7.34 (m, 2H), 4.50 (s, 2H), 1.40 (hept, *J* = 7.5 Hz, 3H), 1.07 (d, *J* = 7.5 Hz, 18H); ^{13}C NMR (101 MHz, $CDCl_3$): δ = 137.9, 135.8, 135.8, 128.2, 33.9, 18.7, 10.9; ^{29}Si NMR (79 MHz, $CDCl_3$): δ = 1.8. IR (ATR): ṽ = 2943 (m), 2862 (m), 1458 (m), 1392 (m), 1097 (m), 1012 (m), 989 (m), 880 (s), 681 (s), 656 (s), 624 (s), 608 (s), 513 (s), 476 (s) cm^{-1}; HRMS (EI) for $C_{16}H_{27}BrSi$ [M]$^+$: calcd: 326.10655, found: 326.10686.

(3a*S*,8a*R*)-5-Bromo-3,3a,8,8a-tetrahydro-2*H*-indeno[1,2-d]oxazol-2-one (S1).[4] (3a*S*-cis)-(−)-3,3a,8,8a-

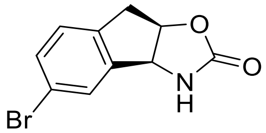

Tetrahydro-2*H*-indeno[1,2-d]oxazol-2-one (5.49 g, 31.3 mmol) was added in one portion to a mixture of CF_3COOH (22 mL) and H_2SO_4 (7 mL) at 0 °C. *N*-Bromosuccinimide (5.95 g, 33.4 mmol) was added in portions and the resulting yellow suspension was stirred at 0 °C for 2 h. Water (20 mL) was slowly introduced and the mixture was neutralized by carefully adding solid NaOH at 0 °C. The mixture was extracted with EtOAc (3 x, 50 mL), the combined organic phases were dried over $MgSO_4$ and concentrated. The crude product was either used directly for the next step (in case of Suzuki cross couplings) or purified by preparative HPLC, using the following conditions: Kromasil 100-5 C18 (150 x 30 mm), MeOH/water = 50/50, Sample dissolved in MeCN (c = 29.1 mg/mL), 8.7 mg sample per run. After preparative HPLC the compound was obtained as a colorless solid (2.95 g, 37 % yield). $[\alpha]_D^{20}$ = –89.5 (c = 0.50, $CHCl_3$); ^{1}H NMR (400 MHz, $CDCl_3$): δ = 7.44 – 7.38 (m, 2H), 7.14 (d, *J* = 8.2 Hz, 1H), 6.79 (s, 1H), 5.44 – 5.38 (m, 1H), 5.13 (d, *J* = 7.1 Hz, 1H), 3.40 – 3.23 (m, 2H); ^{13}C NMR (101 MHz, $CDCl_3$): δ = 159.6, 142.6, 138.8, 132.7, 128.1, 127.3, 121.6, 80.9, 61.0, 38.5; IR (ATR): ṽ = 3217 (w), 1774 (s), 1699 (s), 1474 (m), 1315 (m), 1238 (m), 1044 (s), 813 (s), 698 (m), 435 (m) cm^{-1}; HRMS (ESI$^+$) for $C_{10}H_9NO_2Br^{79}$ [M + H$^+$]$^+$: calcd: 253.98113, found: 253.98119.

[4] C. Liu, J.-C. Yi, Z.-B. Zheng, Y. Tang, L.-X. Dai, S.-L. You, *Angew. Chem. Int. Ed*. **2016**, *55*, 751-754.

7-Bromo-3,3a*S*,8,8a*R*-tetrahydro-2*H*-indeno[1,2-d]oxazol-2-one was identified as the major side product (approx. 17 %), which shows the following characteristic data: ^{1}H NMR (400 MHz, $CHCl_3$): δ = 7.50 (d, *J* = 7.8 Hz, 1H), 7.22 (d, *J* = 7.4 Hz, 1H), 7.17 (t, *J* = 7.6 Hz, 1H), 5.72 (bs, 1H), 5.42 (dt, *J* = 7.2, 4.0 Hz, 1H), 5.25 (dq, *J* = 7.3, 0.7 Hz, 1H), 3.39 (d, *J* = 4.1 Hz, 2H); ^{13}C NMR (101 MHz, $CHCl_3$): δ = 158.6, 141.8, 140.7, 132.6, 129.8, 123.4, 120.8, 79.3, 61.8, 40.5.

(3a*S*,8a*R*)-5-(Prop-1-en-2-yl)-3,3a,8,8a-tetrahydro-2*H*-indeno[1,2-d]oxazol-2-one (**S2**).[4] A flame-dried 2-necked round bottom flask equipped with a condensor was charged with bromide **S1** (1.04 g, 4.11 mmol), potassium isopropenyltrifluoroborate (720 mg, 4.87 mmol), caesium carbonate (2.76 g, 8.46 mmol) and [1,1'-bis(diphenylphosphino)-ferrocene]dichloropalladium(II) (88.7 mg, 0.121 mmol). Degassed THF (20 mL) and degassed water (4 mL) were added, and the resulting suspension was stirred at 80 °C for 12 h. Water (20 mL) was added and the mixture was extracted with EtOAc (3 x, 70 mL). The combined organic phases were dried over $MgSO_4$ and concentrated. The crude product was purified by flash chromatography (silica, hexanes/EtOAc = 5/1) to obtain the title compound as a colorless powder (754 mg, 85 % yield). $[\alpha]_D^{20}$ = –111.4 (c = 0.55, $CHCl_3$); ^{1}H NMR (400 MHz, $CDCl_3$): δ = 7.41 (dd, *J* = 8.0, 1.7 Hz, 1H), 7.35 (s, 1H), 7.21 (d, *J* = 7.9 Hz, 1H), 6.49 (s, 1H), 5.42 (ddd, *J* = 7.3, 6.2, 2.1 Hz, 1H), 5.34 – 5.31 (m, 1H), 5.16 (d, *J* = 7.2 Hz, 1H), 5.08 – 5.06 (m, 1H), 3.43 – 3.28 (m, 2H), 2.12 (dd, *J* = 1.5, 0.8 Hz, 3H); ^{13}C NMR (101 MHz, $CDCl_3$): δ = 159.6, 142.8, 141.6, 140.5, 139.0, 127.0, 125.4, 121.8, 113.0, 81.0, 61.3, 38.7, 22.1; IR (ATR): ṽ = 3376 (w), 3275 (w), 1759 (s), 1747 (s), 1726 (s), 1210 (m), 1031 (m), 825 (m), 423 (m) cm^{-1}; HRMS (ESI$^+$) for $C_{13}H_{13}NO_2Na$ [M + Na$^+$]$^+$: calcd: 238.08385, found: 238.08377.

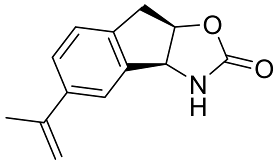

(3a*S*,8a*R*)-5-(4-(*tert*-butyl)phenyl)-3,3a,8,8a-tetrahydro-2*H*-indeno[1,2-d]oxazol-2-one (**S3**). Prepared analogously from bromide **S1** (1.01 g, 3.98 mmol) and ((4-*tert*-butyl)phenyl)boronic acid (796 mg, 4.47 mmol) as a colorless solid (650 mg, 56 % yield). $[\alpha]_D^{20}$ = - 116.8 (c = 0.578, $CHCl_3$); ^{1}H NMR (400 MHz, $CDCl_3$): δ = 7.53 – 7.41 (m, 6H), 7.30 – 7.24 (m, 1H), 6.74 (s, 1H), 5.43 (ddd, *J* = 7.4, 6.3, 1.9 Hz, 1H), 5.20 (d, *J* = 7.3 Hz, 1H), 3.45 – 3.27 (m, 2H), 1.36 (s, 9H); ^{13}C NMR (101 MHz, $CDCl_3$): δ = 159.7, 150.6, 141.3, 141.0, 138.6, 137.7, 128.5, 126.9, 125.9, 125.9, 123.3, 81.0, 61.3, 38.7, 34.7, 31.5; IR (ATR): ṽ = 3230 (w), 2956 (m), 1650 (s), 1488 (m), 1204 (m), 1041 (m), 960 (m), 831 (s), 769 (m), 706 (m), 530 (m) cm^{-1}; HRMS (ESI$^+$) for $C_{20}H_{21}N_1O_2Na_1$ [M + Na$^+$]$^+$: calcd: 330.146448, found: 330.146410.

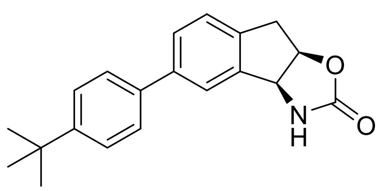

(1*S*,2*R*)-1-Amino-6-isopropyl-2,3-dihydro-1*H*-inden-2-ol (**S4**). A 2-necked 50 mL round bottom flask was charged with compound **S2** (750 mg, 3.54 mmol) and Pd/C (10 % *w/w*, 209 mg, 0.196 mmol) under Ar. EtOH (20 mL) was added and the flask was purged with H_2. The mixture was stirred under H_2 atmosphere (balloon) for 2.25 h, after which the suspension was filtered through a plug of Celite, eluting with EtOH (3x10 mL). Water (30 mL) and KOH (703 mg, 12.5 mmol) were added to the combined filtrates and the resulting solution was stirred at reflux temperature overnight. After cooling to rt, the solution was extracted with CH_2Cl_2 (3 x, 50 mL), the combined organic phases were dried over $MgSO_4$, filtered and concentrated to yield the product as a colorless solid (559 mg, 84 % yield), which was used in the next step without further purification. $[\alpha]_D^{20}$ = –123.7 (c = 0.49, $CHCl_3$); ^{1}H NMR (400 MHz, $CDCl_3$): δ = 7.11 – 7.06

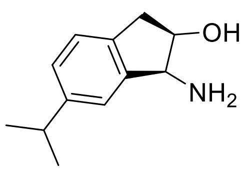

(m, 2H), 7.03 (dd, J = 7.7, 1.7 Hz, 1H), 4.30 (td, J = 5.6, 3.0 Hz, 1H), 4.21 (d, J = 5.4 Hz, 1H), 2.98 (dd, J = 16.2, 5.7, 1H), 2.88 – 2.79 (m, 2H), 2.62 – 1.35 (bs, 3H) 1.18 (d, J = 6.9 Hz, 6H); ^{13}C NMR (101 MHz, $CDCl_3$): δ = 148.1, 144.2, 138.4, 126.5, 125.4, 121.9, 73.2, 58.7, 39.2, 34.3, 24.4, 24.3. IR (ATR): ṽ = 3308 (m), 2954 (s), 2866 (m), 1331 (m), 1021 (s), 987 (s), 735 (s), 711 (s), 698 (m) cm^{-1}. HRMS (ESI$^+$) for $C_{12}H_{18}NO$ [M + H$^+$]$^+$: calcd: 192.13829, found: 192.13824.

(3a*S*,8a*R*)-5-((3*S*,5*S*,7*S*)-adamantan-1-yl)-3,3a,8,8a-tetrahydro-2*H*-indeno[1,2-d]oxazol-2-one (**S5**). A round bottom flask was charged with (3a*S*,8a*R*)-3,3a,8,8a-tetrahydro-2*H*-indeno[1,2-d]oxazol-2-one (1.032 g, 5.89 mmol), trifluoroacetic acid (5 mL) and conc. sulfuric acid (2 mL). The mixture was cooled to 0 °C in an ice bath before 1-adamantol (911 mg, 5.98 mmol) was added in portions. After stirring for 30 min at this temperature, water (30 mL) was added and the solution was neutralized by careful addition of solid Na_2CO_3. The resulting suspension was extracted with EtOAc (50 mL) and CH_2Cl_2 (50 mL), the combined organic layers were dried over $MgSO_4$ and concentrated, and the residue was purified by flash chromatography (silica, hexanes/EtOAc = 5/1) to give the title compound as a colorless solid (408 mg, 22 % yield). $[\alpha]_D^{20}$ = –112.7 (c = 0.51, $CHCl_3$); ^{1}H NMR (400 MHz, $CDCl_3$): δ = 7.33 – 7.24 (m, 2H), 7.16 (d, J = 7.9 Hz, 1H), 5.37 (ddd, J = 7.8, 6.4, 1.8 Hz, 1H), 5.12 (d, J = 7.2 Hz, 1H), 3.37 – 3.20 (m, 2H), 2.07 – 2.00 (m, 2H), 1.82 – 1.65 (m, 13H); ^{13}C NMR (101 MHz, $CDCl_3$): δ = 160.1, 151.7, 140.4, 136.7, 126.2, 125.0, 121.3, 81.1, 61.5, 43.2, 38.5, 36.7, 36.2, 28.9; IR (ATR): ṽ = 3344 (w), 2897 (m), 2845 (m), 1759 (m), 1721 (s), 1201 (m), 1031 (m), 602 (m), 528 (m) cm^{-1}; HRMS (ESI$^+$) for $C_{20}H_{23}NO_2Na$ [M + Na$^+$]$^+$: calcd: 332.16210, found: 332.16200.

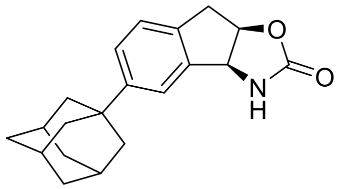

(1*S*,2*R*)-1-Amino-6-bromo-2,3-dihydro-1*H*-inden-2-ol (**S6**).[4] A 100 mL round bottom flask equipped with a condenser was charged with compound **S1** (782 mg, 3.17 mmol), KOH (657 mg, 11.7 mmol), EtOH (15 mL) and water (15 mL). The mixture was stirred at 80 °C for 5 h. After cooling, the mixture was extracted with EtOAc (3 x, 30 mL) and the organic phase was extracted with aqueous HCl (2 M, 30 mL). The aqueous phase was brought to pH 14 by adding solid NaOH and was extracted again with EtOAc (3 x, 30 mL). The combined EtOAc layers were dried over $MgSO_4$ and concentrated to obtain the title compound as a colorless solid (624 mg, 89 % yield). $[\alpha]_D^{20}$ = –93.1 (c = 0.53, $CHCl_3$); ^{1}H NMR (400 MHz, $CDCl_3$): δ = 7.43 (t, J = 1.3 Hz, 1H), 7.35 (dd, J = 8.0, 1.2 Hz, 1H), 7.10 (d, J = 7.9 Hz, 1H), 4.40 – 4.28 (m, 2H), 3.02 (dd, J = 16.6, 5.4 Hz, 1H), 2.88 (dd, J = 16.6, 2.7 Hz, 1H), 2.22 (s, 3H); ^{13}C NMR (101 MHz, $CDCl_3$): δ = 146.6, 140.0, 131.1, 127.4, 127.2, 120.6, 73.2, 58.7, 39.1; IR (ATR): ṽ = 3354 (m), 2874 (s), 1577 (m), 1465 (s), 1156 (s), 1039 (s), 994 (s), 968 (s), 886 (s), 773 (s), 542 (s), 484 (s) cm^{-1}; HRMS (ESI$^+$) for $C_9H_{11}NOBr^{79}$ [M + H$^+$]$^+$: calcd: 228.00186, found: 228.00169.

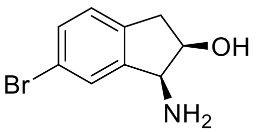

(1*S*,2*R*)-1-Amino-6-isopropyl-2,3-dihydro-1*H*-inden-2-ol (**S7**). Prepared according to the literature as a colorless solid (235 mg, 46 % yield).[5] $[\alpha]_D^{20}$ = –73.5 (c = 0.50, $CHCl_3$); ^{1}H NMR (400 MHz, $CDCl_3$): δ = 7.15 (dd, J = 4.6, 3.1 Hz, 2H), 7.08 (dd, J = 7.8, 1.5 Hz, 1H), 4.46 – 4.21 (m, 2H), 3.06 (dd, J = 16.3, 5.5 Hz, 1H), 2.89 (dd, J = 16.3, 2.9 Hz, 1H), 2.54 – 2.21 (m, 4H), 1.86 (m, 4H), 1.80 – 1.70 (m, 1H), 1.51 – 1.31 (m, 4H);

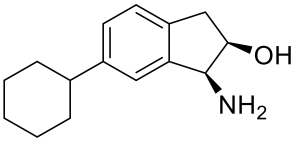

[5] S. Chen, L. Wu, Q. Shao, G. Yang, W. Zhang, *Chem. Commun.* **2018**, *54*, 2522.

^{13}C NMR (101 MHz, $CDCl_3$): δ = 147.3, 144.1, 138.4, 126.9, 125.3, 122.3, 73.1, 58.6, 44.8, 39.2, 34.9, 34.8, 27.1, 26.3; IR (ATR): ṽ = 2977 (w), 2939 (s), 2850 (s), 1714 (w), 1448 (m), 1366 (m), 1246 (m), 1144 (m), 1018 (s), 850 (m), 832 (s), 731 (s) cm^{-1}; HRMS (ESI$^+$) for $C_{15}H_{22}NO$ [M + H$^+$]$^+$: calcd: 232.16959, found: 232.16938.

(1*S*,2*R*)-1-Amino-6-(4-(*tert*-butyl)phenyl)-2,3-dihydro-1*H*-inden-2-ol (**S8**). Prepared analogously as a colorless solid (410 mg, 69 % yield). $[\alpha]_D^{20}$ = –83.4 (c = 0.46, $CHCl_3$); ^1H NMR (400 MHz, $CDCl_3$): δ = 7.56 – 7.42 (m, 7H), 4.44 (d, *J* = 43.4 Hz, 2H), 3.97 (s, 2H), 3.19 – 2.91 (m, 2H), 1.93 (s, 1H), 1.35 (s, 9H); ^{13}C NMR (101 MHz, $CDCl_3$): δ = 150.3, 140.5, 139.9, 138.4, 127.5, 126.9, 125.9, 125.8, 122.9, 72.8, 39.2, 34.7, 31.5; IR (ATR): ṽ = 2958 (m), 1486 (m), 1392 (m), 1099 (m), 851 (s), 724 (s), 563 (m) cm^{-1}; HRMS (ESI$^+$) for $C_{19}H_{24}NO$ [M + H$^+$]$^+$: calcd: 282.18524, found: 282.18479.

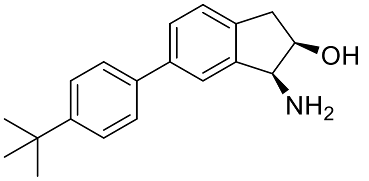

(1*S*,2*R*)-6-((1*S*,3*R*)-Adamantan-1-yl)-1-amino-2,3-dihydro-1*H*-inden-2-ol (**S9**). This compound was prepared analogously as a colorless solid (357 mg, 95 % yield). $[\alpha]_D^{20}$ = –98.3 (c = 0.48, $CHCl_3$); ^1H NMR (400 MHz, $CDCl_3$): δ = 7.33 – 7.30 (m, 2H), 7.28 – 7.24 (m, 2H), 7.18 (d, *J* = 7.9 Hz, 2H), 4.41 – 4.35 (m, 2H), 4.30 (d, *J* = 5.4 Hz, 2H), 3.06 (ddd, *J* = 16.3, 5.7, 1.0 Hz, 2H), 2.90 (dd, *J* = 16.3, 3.1 Hz, 2H), 2.37 (s, 3H), 2.13 – 2.08 (m, 6H), 1.94 – 1.88 (m, 14H), 1.83 – 1.71 (m, 10H); ^{13}C NMR (101 MHz, $CDCl_3$): δ = 150.8, 143.8, 138.1, 125.1, 125.0, 120.5, 73.1, 58.7, 43.6, 39.1, 36.9, 29.1; IR (ATR): ṽ = 3325 (w), 2899 (s), 2846 (s), 1449 (w), 906 (m), 804 (m), 738 (s) cm^{-1}; HRMS (ESI$^+$) for $C_{19}H_{26}NO$ [M + Na$^+$]$^+$: calcd: 284.20089, found: 284.20035.

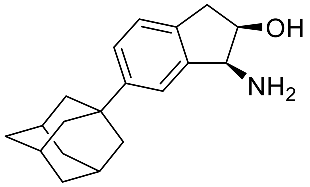

LIGAND SYNTHESES

Preparation of Bis((3a*S*,8a*R*)-5-isopropyl-3a,8a-dihydro-8*H*-indeno[1,2-d]oxazol-2-yl)methane (S10). The compound was prepared according to a literature procedure.[6] A flame-dried 2-necked round bottom flask was charged with compound **S4** (629 mg, 3.30 mmol) and diethyl malonimidate dihydrochloride (323 mg, 1.40 mmol). THF (15 mL) was added and the mixture was stirred at 50 °C for 20 h. After reaching rt, the mixture was filtered through a plug of Celite®, eluting with CH_2Cl_2. The crude product was purified by flash chromatography (silica, EtOAc/EtOH = 1/1) to give the title compound as a colorless solid (238 mg, 40 %). $[\alpha]_D^{20}$ = –214.7 (c = 0.34, $CHCl_3$); ^1H NMR (400 MHz, $CDCl_3$): δ = 7.34 (s, 2H), 7.19 – 7.10 (m, 4H), 5.54 (d, *J* = 8.0 Hz, 2H), 5.39 – 5.29 (m, 2H), 3.35 (dd, *J* = 17.9, 7.1 Hz, 2H), 3.27 (s, 2H), 3.14 (dd, *J* = 17.8, 1.8 Hz, 2H), 2.91 (hept, *J* = 6.9 Hz, 2H), 1.24 (d, *J* = 6.9 Hz, 12H); ^{13}C NMR (101 MHz, $CDCl_3$): δ = 162.1, 148.6, 141.9, 137.2, 127.2, 125.2, 123.4, 84.1, 76.7 (from 2D), 39.5, 34.2, 29.0, 24.4, 24.2;

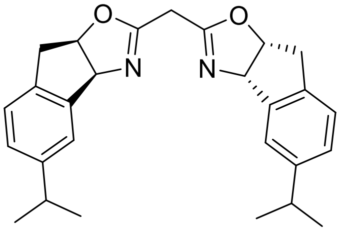

6 C. Liu, J.-C. Yi, Z.-B. Zheng, Y. Tang, L.-X. Dai, S.-L. You, *Angew. Chem. Int. Ed.* **2016**, *55*, 751.

IR (ATR): ṽ = 2955 (s), 2867 (w), 1657 (s), 1194 (s), 993 (s), 818 (s), 710 (m), 479 (m) cm^{-1}; HRMS (ESI^+) for $C_{27}H_{31}N_2O_2$ $[M + H^+]^+$: calcd: 415.23800, found: 415.23812.

Bis((3a*S*,8a*R*)-5-bromo-3a,8a-dihydro-8*H*-indeno[1,2-d]oxazol-2-yl)methane (**S11**). Prepared analogously from compound **S6** as a colorless solid (251 mg, 42 % yield). α_D^{20}= –427.0 (c = 0.58, $CHCl_3$); ^1H NMR (400 MHz, $CDCl_3$): δ = 7.57 (s, 2H), 7.37 (dd, *J* = 8.1, 1.9 Hz, 2H), 7.10 (d, *J* = 8.1 Hz, 2H), 5.52 (d, *J* = 7.9 Hz, 2H), 5.33 (ddd, *J* = 8.2, 7.0, 1.7 Hz, 2H), 3.32 (dd, *J* = 18.1, 7.0 Hz, 2H), 3.27 (s, 2H), 3.09 (d, *J* = 18.1 Hz, 2H); ^{13}C NMR (101 MHz, $CDCl_3$): δ = 162.4, 144.0, 138.7, 131.8, 128.8, 126.9, 121.2, 83.9, 76.5, 39.4, 28.7. IR (ATR): ṽ = 2924 (w), 1654 (s), 1470 (m), 1199 (s), 1158 (m), 1000 (s), 956 (m), 915 (s), 837 (s), 791 (s), 471 (m) cm^{-1}; HRMS (ESI^+) for $C_{21}H_{17}(Br^{79})_2N_2O_2$ $[M + H^+]^+$: calcd: 486.96515, found: 486.96529.

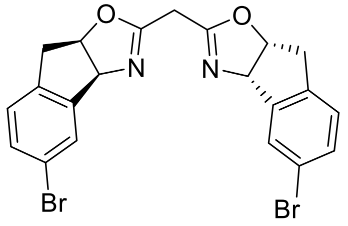

Bis((3a*S*,8a*R*)-5-cyclohexyl-3a,8a-dihydro-8*H*-indeno[1,2-d]oxazol-2-yl)methane (**S12**). Prepared analogously from compound **S7** (221 mg, 0.957 mmol) as a colorless solid (179 mg, 81 % yield). $[\alpha]_D^{20}$ = –298.4 (c = 0.36, $CHCl_3$); ^1H NMR (400 MHz, $CDCl_3$): δ = 7.32 (s, 2H), 7.17 – 7.09 (m, 4H), 5.53 (d, *J* = 8.0 Hz, 2H), 5.37 – 5.30 (m, 2H), 3.35 (dd, *J* = 17.9, 7.0 Hz, 2H), 3.27 (s, 2H), 3.14 (dd, *J* = 17.8, 1.8 Hz, 2H), 2.56 – 2.43 (m, 2H), 1.90 – 1.79 (m, 9H), 1.78 – 1.70 (m, 2H), 1.48 – 1.31 (m, 9H); ^{13}C NMR (101 MHz, $CDCl_3$): δ = 162.0, 147.8, 141.9, 137.2, 127.6, 125.1, 123.8, 84.0, 76.8, 44.6, 39.5, 34.9, 34.6, 29.0, 27.1, 27.1, 26.3; IR (ATR): ṽ = 2941 (s), 2851 (s), 1657 (s), 1445 (m), 1351 (m), 1202 (s), 989 (s), 920 (s), 817 (s), 718 (m) cm^{-1}; HRMS (ESI^+) for $C_{33}H_{39}N_2O_2$ $[M + H^+]^+$: calcd: 495.30060, found: 495.30037.

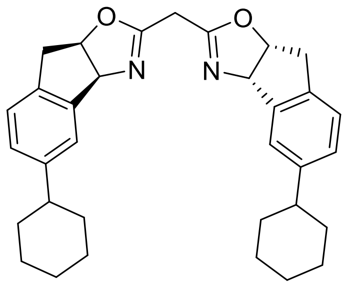

Bis((3a*S*,8a*R*)-5-(4-(*tert*-butyl)phenyl)-3a,8a-dihydro-8*H*-indeno[1,2-d]oxazol-2-yl)methane (**S13**). Prepared analogously from compound **S8** (473 mg, 1.68 mmol) as a colorless solid (136 mg, 41 % yield). $[\alpha]_D^{20}$ = –251.9 (c = 0.35, $CHCl_3$); ^1H NMR (400 MHz, $CDCl_3$): δ = 7.71 – 7.69 (m, 2H), 7.56 – 7.42 (m, 10H), 7.28 (d, *J* = 7.9 Hz, 2H), 5.62 (d, *J* = 8.0 Hz, 2H), 5.39 (ddd, *J* = 7.9, 7.0, 1.8 Hz, 2H), 3.48 – 3.38 (m, 2H), 3.32 (s, 2H), 3.21 (d, *J* = 18.0 Hz, 2H), 1.36 (s, 18H); ^{13}C NMR (101 MHz, $CDCl_3$): δ = 162.2, 150.3, 142.4, 140.7, 138.6, 138.1, 127.7, 126.9, 125.8, 125.6, 124.1, 84.1, 76.7, 53.9, 39.5, 34.7, 31.5, 29.4, 28.9; IR (ATR): ṽ = 3230 (w), 2958 (w), 1774 (s), 1488 (m), 1204 (m), 1041 (m), 823 (s) cm^{-1}; HRMS (ESI^+) for $C_{41}H_{43}N_2O_2$ $[M + H^+]^+$: calcd: 595.33190, found: 595.33187.

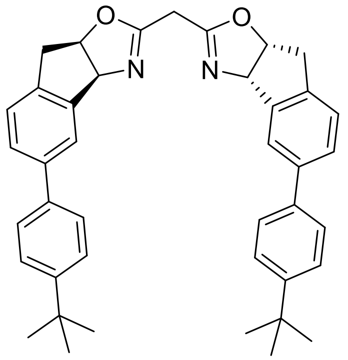

Bis((3a*S*,8a*R*)-5-((3*S*,5*S*,7*S*)-adamantan-1-yl)-3a,8a-dihydro-8*H*-indeno[1,2-d]oxazol-2-yl)methane (**S14**).

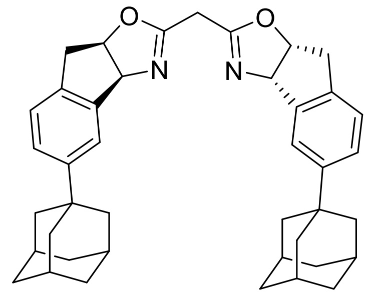

Prepared analogously from compound **S9** (347 mg, 1.22 mmol) as a colorless solid (229 mg, 88 % yield). $[\alpha]_D^{20}$ = –214.5 (c = 0.50, $CHCl_3$); ^{1}H NMR (400 MHz, $CDCl_3$): δ = 7.48 (s, 2H), 7.29 (dd, *J* = 8.0, 1.9 Hz, 2H), 7.18 (d, *J* = 8.0 Hz, 2H), 5.55 (d, *J* = 8.0 Hz, 2H), 5.38 – 5.31 (m, 2H), 3.36 (dd, *J* = 17.9, 7.0 Hz, 2H), 3.27 (s, 2H), 3.16 (d, *J* = 17.9, 1.8 Hz, 2H), 2.12 – 2.06 (m, 6H), 1.91 (d, *J* = 2.9 Hz, 11H), 1.82 – 1.70 (m, 13H); ^{13}C NMR (101 MHz, $CDCl_3$): δ = 162.0, 151.2, 141.7, 137.0, 125.6, 124.9, 122.0, 84.0, 77.0, 43.5, 39.5, 36.9, 36.4, 29.1; IR (ATR): ṽ = 2901 (s), 2846 (s), 1656 (s), 1154 (m), 1005 (s), 907 (m), 804 (s), 674 (s) cm^{-1}; HRMS (ESI$^+$) for $C_{41}H_{47}N_2O_2$ [M + H$^+$]$^+$: calcd: 599.36320, found: 599.36339.

Representative Procedure for *gem*-Dialkylation. Preparation of (3a*S*,3a'*S*,8a*R*,8a'*R*)-2,2'-(1,3-bis(4-(*tert*-butyl)phenyl)propane-2,2-diyl)bis(3a,8a-dihydro-8*H*-indeno[1,2-d]oxazole) (L19). The compound was prepared in analogy to a literature procedure.[7] Bis((3a*S*,8a*R*)-5-isopropyl-3a,8a-dihydro-8*H*-indeno[1,2-d]oxazol-2-yl)methane (**S10**) (314 mg, 0.325 mmol) was dissolved in dry THF (10 mL) in a flame-dried Schlenk tube. The solution was cooled to 0 °C and NaH (55.8 mg, 2.33 mmol) was added in portions. A solution of *p-tert*-butyl benzyl bromide (0.300 mL, 1.59 mmol) in THF (5 mL) was added dropwise and the resulting mixture was stirred for 6 h at rt. Sat. NH_4Cl solution (10 mL) and water (30 mL) were added and the mixture was repeatedly extracted with CH_2Cl_2 (30 mL). The combined organic phases were dried over $MgSO_4$ and concentrated in the presence of Celite®. The loaded Celite® was added on top of flash column filled with silica, eluting the product with hexanes/EtOAc (5/1). Evaporation of the product-containing fractions gave the title compound as a colorless solid material (130 mg, 58 % yield). $[\alpha]_D^{20}$ = –231.5 (c = 0.45, $CHCl_3$); ^{1}H NMR (400 MHz, $CDCl_3$): δ = 7.39 (s, 2H), 7.25 – 7.17 (m, 4H), 6.89 – 6.83 (m, 4H), 6.73 – 6.69 (m, 4H), 5.59 (d, *J* = 8.0 Hz, 2H), 5.31 (ddd, *J* = 8.2, 6.9, 1.6 Hz, 2H), 3.36 – 3.19 (m, 4H), 3.08 – 2.91 (m, 6H), 1.29 (dd, *J* = 7.0, 5.8 Hz, 12H), 1.21 (s, 18H); ^{13}C NMR (101 MHz, $CDCl_3$): δ = 167.6, 149.0, 148.5, 141.7, 137.4, 133.2, 130.1, 127.0, 125.0, 124.6, 123.8, 83.6, 76.8, 47.7, 39.3, 38.0, 34.4, 34.1, 31.5, 24.5, 24.3; IR (ATR): ṽ = 2957 (s), 2868 (m), 1644 (s), 1162 (m), 1014 (s), 835 (s), 568 (m) cm^{-1}; HRMS (ESI$^+$) for $C_{49}H_{59}N_2O_2$ [M + H$^+$]$^+$: calcd: 707.45710, found: 707.45729.

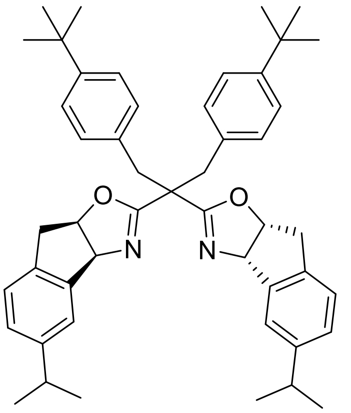

(3a*S*,3a'*S*,8a*R*,8a'*R*)-2,2'-(Cyclopropane-1,1-diyl)bis(3a,8a-dihydro-8*H*-indeno[1,2-d]oxazole) (L3).

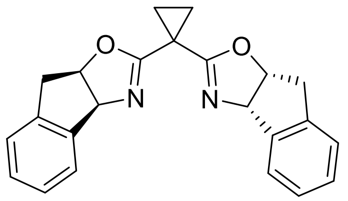

Prepared analogously as a colorless solid (300 mg, 79 % yield). $[\alpha]_D^{20}$ = –335.7 (c = 0.51, $CHCl_3$); ^{1}H NMR (400 MHz, $CDCl_3$): δ = 7.47 – 7.42 (m, 2H), 7.23 (d, *J* = 3.5 Hz 6H), 5.52 (dd, *J* = 8.0, 0.8 Hz, 2H), 5.32 (ddd, *J* = 7.9, 7.0, 2.0 Hz, 2H), 3.43 – 3.34 (m, 2H), 3.19 (dd, *J* = 17.9, 1.9 Hz, 2H), 1.38 – 1.23 (m, 4H); ^{13}C NMR (101 MHz, $CDCl_3$): δ = 166.0, 141.9, 139.9, 128.5, 127.5, 125.8, 125.3, 83.5, 76.5, 39.8, 18.5, 15.9; IR (ATR): ṽ = 2921 (w), 1656 (s),

7 H. Xiong, H. Xu, S. Liao, Z. Xie, Y. Tang, *J. Am. Chem. Soc.* **2013**, *135*, 7851.

1364 (m), 1159 (s), 1118 (s), 995 (s), 741 (s), 730 (s), 606 (m), 438 (s) cm^{-1}; HRMS (ESI$^+$) for $C_{23}H_{20}N_2O_2Na$ [M + Na$^+$]$^+$: calcd: 379.14170, found: 379.14186.

(3a*S*,3a'*S*,8a*R*,8a'*R*)-2,2'-(Cycloheptane-1,1-diyl)bis(3a,8a-dihydro-8*H*-indeno[1,2-d]oxazole) (L4).

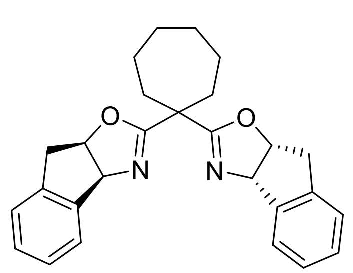

Prepared analogously as a colorless solid (45.0 mg, 45 % yield). $[\alpha]_D^{20}$ = –183.2 (c = 0.65, CHCl$_3$); ^{1}H NMR (400 MHz, CDCl$_3$) δ = 7.54 – 7.47 (m, 2H), 7.29 – 7.20 (m, 6H), 5.52 (d, *J* = 7.8 Hz, 2H), 5.24 – 5.17 (m, 2H), 3.27 (dd, *J* = 17.9, 6.9 Hz, 2H), 2.90 (d, *J* = 17.8 Hz, 2H), 2.19 – 1.99 (m, 4H), 1.51 – 1.34 (m, 8H); ^{13}C NMR (101 MHz, CDCl$_3$): δ = 169.2, 142.2, 139.8, 128.4, 127.4, 125.7, 125.1, 83.0, 76.5, 46.2, 39.7, 34.4, 29.6, 23.2; IR (ATR): ṽ = 2917 (w), 1637 (s), 1167 (m), 999 (s), 847 (m), 728 (s), 721 (s), 712 (s), 595 (m) cm^{-1}; HRMS (ESI$^+$) for $C_{27}H_{28}N_2O_2Na$ [M + Na$^+$]$^+$: calcd: 435.20430, found: 435.20440.

(3a*S*,3a'*S*,8a*R*,8a'*R*)-2,2'-(1,3-diphenylpropane-2,2-diyl)bis(3a,8a-dihydro-8*H*-indeno[1,2-d]oxazole)

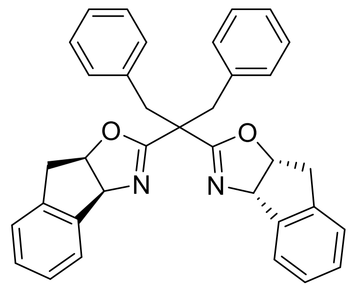

(L5). Prepared analogously as a colorless solid (113 mg, 75 % yield). $[\alpha]_D^{20}$ = –183.5 (c = 0.50, CHCl$_3$); ^{1}H NMR (400 MHz, CDCl$_3$): δ = 7.49 – 7.44 (m, 2H), 7.37 – 7.27 (m, 6H), 7.02 – 6.97 (m, 2H), 6.81 (dd, *J* = 8.6, 6.9 Hz, 4H), 6.74 (dd, *J* = 8.2, 1.5 Hz, 4H), 5.60 (d, *J* = 7.8 Hz, 2H), 5.30 (ddd, *J* = 7.9, 6.8, 1.3 Hz, 2H), 3.34 (dd, *J* = 18.1, 6.7 Hz, 2H), 3.25 (d, *J* = 14.3 Hz, 2H), 3.10 – 3.01 (m, 4H); ^{13}C NMR (101 MHz, CDCl$_3$): δ = 167.5, 141.6, 140.0, 136.2, 130.3, 128.5, 127.8, 127.6, 126.4, 125.9, 125.3, 83.6, 76.6, 47.6, 39.5, 38.8; IR (ATR) ṽ = 2934 (w), 1644 (s), 1455 (m), 1160 (m), 1018 (m), 845 (m), 760 (s), 731 (s), 696 (s), 539 (s) cm^{-1}. HRMS (ESI$^+$) for $C_{35}H_{31}N_2O_2$ [M + H$^+$]$^+$: calcd: 511.23800, found: 511.23847.

(3a*S*,3a'*S*,8a*R*,8a'*R*)-2,2'-(1,3-bis(4-(trifluoromethyl)phenyl)propane-2,2-diyl)bis(3a,8a-dihydro-8*H*-indeno[1,2-d]oxazole) (L6).

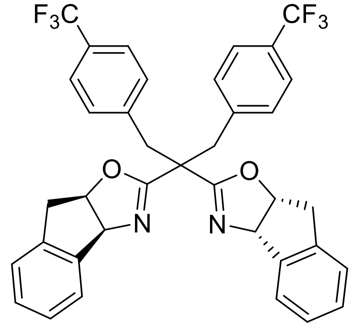

Prepared analogously as a colorless solid (197 mg, 99 % yield). $[\alpha]_D^{20}$ = –179.9 (c = 0.31, CHCl$_3$); ^{1}H NMR (400 MHz, CDCl$_3$): δ = 7.43 – 7.35 (m, 4H), 7.34 – 7.27 (m, 4H), 6.98 (d, *J* = 8.0 Hz, 4H), 6.82 (d, *J* = 8.0 Hz, 4H), 5.62 (d, *J* = 7.8 Hz, 2H), 5.32 (ddd, *J* = 7.7, 6.7, 1.0 Hz, 2H), 3.41 – 3.29 (m, 4H), 3.04 (d, *J* = 1.9 Hz, 2H), 3.00 (d, *J* = 5.7 Hz, 2H); ^{13}C NMR (101 MHz, CDCl$_3$): δ = 166.8, 141.5, 140.0, 139.8, 130.5, 128.8, 128.7 (q, *J* = 32.3 Hz), 127.8, 125.9, 125.4, 124.7 (q, *J* = 3.7 Hz), 124.3 (q, *J* = 272.0 Hz), 84.1, 76.5, 47.3, 39.7, 39.3; ^{19}F NMR (282 MHz, CDCl$_3$): δ = –62.5; IR (ATR): ṽ = 2937 (w), 1643 (m), 1324 (s), 1161 (s), 1115 (s), 1068 (s), 1020 (s), 859 (m), 755 (s), 605 (m) cm^{-1}; HRMS (ESI$^+$) for $C_{37}H_{29}N_2O_2F_6$ [M + H$^+$]$^+$: calcd: 647.21277, found: 647.21364.

(3a*S*,3a'*S*,8a*R*,8a'*R*)-2,2'-(1,3-Bis(4-bromophenyl)propane-2,2-diyl)bis(3a,8a-dihydro-8*H*-indeno[1,2-d]oxazole) (L7).

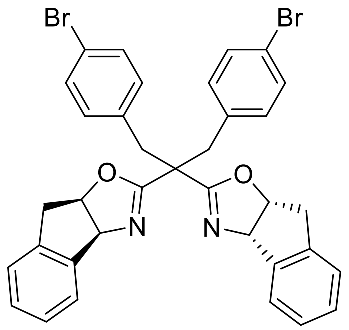

Prepared analogously as a colorless solid (304 mg, 87 %). $[\alpha]_D^{20}$ = –190.3 (c = 0.58, $CHCl_3$); ^1H NMR (400 MHz, $CDCl_3$): δ = 7.45 – 7.28 (m, 8H), 6.88 – 6.82 (m, 4H), 6.59 – 6.54 (m, 4H), 5.60 (d, *J* = 7.7 Hz, 2H), 5.30 (ddd, *J* = 7.8, 6.7, 1.2 Hz, 2H), 3.35 (dd, *J* = 18.1, 6.6 Hz, 2H), 3.19 (d, *J* = 14.2 Hz, 2H), 3.04 (d, *J* = 18.1 Hz, 2H), 2.93 (d, *J* = 14.3 Hz, 2H); ^{13}C NMR (101 MHz, $CDCl_3$): δ = 167.0, 141.5, 139.8, 134.9, 131.9, 130.8, 128.8, 127.8, 125.9, 125.3, 120.5, 83.9, 76.5, 47.3, 39.3, 39.0; IR (ATR): ṽ = 2945 (w), 1646 (s), 1482 (m), 1163 (m), 1010 (s), 843 (m), 746 (s), 711 (m), 612 (m), 546 (s), 454 (m) cm^{-1}; HRMS (ESI^+) for $C_{35}H_{29}N_2O_2(Br^{79})_2$ $[M + H^+]^+$: calcd: 667.05905, found: 667.06023.

(3a*S*,3a'*S*,8a*R*,8a'*R*)-2,2'-(1,3-bis(4-(*tert*-butyl)phenyl)propane-2,2-diyl)bis(3a,8a-dihydro-8*H*-indeno[1,2-d]oxazole) (L8).

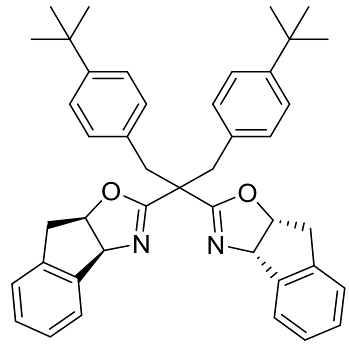

Prepared analogously as a colorless solid (0.153 g, 81 % yield). $[\alpha]_D^{20}$ = –170.3 (c = 0.78, $CHCl_3$); ^1H NMR (400 MHz, $CDCl_3$): δ = 7.49 – 7.44 (m, 2H), 7.39 – 7.27 (m, 6H), 6.84 – 6.79 (m, 4H), 6.73 – 6.67 (m, 4H), 5.61 (d, *J* = 7.9 Hz, 2H), 5.32 (ddd, *J* = 8.0, 6.8, 1.4 Hz, 2H), 3.35 (dd, *J* = 18.0, 6.8 Hz, 2H), 3.21 (d, *J* = 14.2 Hz, 2H), 3.08 (d, *J* = 18.0 Hz, 2H), 3.00 (d, *J* = 14.2 Hz, 2H), 1.21 (s, 18H); ^{13}C NMR (101 MHz, $CDCl_3$): δ = 167.7, 148.9, 141.7, 140.0, 133.1, 130.0, 128.5, 127.6, 126.0, 125.3, 124.6, 83.5, 76.7, 47.8, 39.5, 37.9, 34.4, 31.5; IR (ATR): ṽ = 2956 (w), 1644 (s), 1160 (m), 1013 (s), 856 (m), 817 (m), 747 (s), 608 (m), 569 (m) cm^{-1}; HRMS (ESI^+) for $C_{43}H_{47}N_2O_2$ $[M + H^+]^+$: calcd: 623.36320, found: 623.36324. The spectral data is consistent with previously reported values.[7]

(3a*S*,3a'*S*,8a*R*,8a'*R*)-2,2'-(1,3-Bis(3,5-dimethoxyphenyl)propane-2,2-diyl)bis(3a,8a-dihydro-8*H*-indeno[1,2-d]oxazole) (L9).

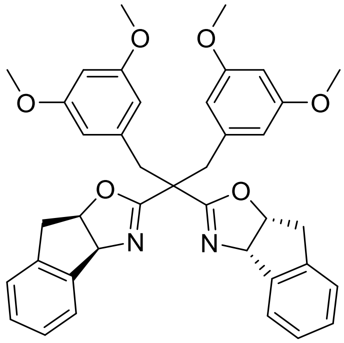

Prepared analogously as a colorless solid (111 mg, 59 % yield). $[\alpha]_D^{20}$ = –249.4 (c = 0.49, $CHCl_3$); ^1H NMR (400 MHz, $CDCl_3$): δ = 7.52 – 7.46 (m, 2H), 7.26 (tdd, *J* = 9.3, 7.6, 4.3 Hz, 6H), 6.23 (s, 6H), 5.51 (d, *J* = 8.0 Hz, 2H), 5.18 (ddd, *J* = 8.1, 7.1, 1.9 Hz, 2H), 3.58 (s, 12H), 3.38 (d, *J* = 14.3 Hz, 2H), 3.28 – 3.19 (m, 2H), 3.11 (d, *J* = 14.4 Hz, 2H), 2.82 – 2.73 (m, 2H); ^{13}C NMR (101 MHz, $CDCl_3$): δ = 167.2, 160.3, 141.7, 139.9, 138.9, 128.5, 127.5, 125.8, 125.2, 108.3, 99.0, 83.0, 76.7, 55.1, 47.7, 39.6, 39.1; IR (ATR): ṽ = 2937 (w), 1642 (m), 1591 (s), 1459 (m), 1430 (m), 1205 (m), 1152 (s), 1072 (m), 830 (m), 746 (m), 696 (m) cm^{-1}; HRMS (ESI^+) for $C_{39}H_{39}N_2O_6$ $[M + H^+]^+$: calcd: 631.28026, found: 631.28093.

(7a*S*,7a'*S*,10a*R*,10a'*R*)-9,9'-(1,3-Bis(3,5-di-*tert*-butylphenyl)propane-2,2-diyl)bis(7a,10a-dihydro-7*H*-benzo[6,7]indeno[1,2-d]oxazole) (*ent*-L10). Prepared analogously from bis((7a*S*,10a*R*)-7a,10a-dihydro-7*H*-benzo[6,7]indeno[1,2-d]oxazol-9-yl)methane (60.0 mg, 0.139 mmol)[8] as a colorless solid (45.0 mg, 39 % yield). $[\alpha]_D^{20}$ = 217.3 (c = 0.47, $CHCl_3$); ^1H NMR (400 MHz, $CDCl_3$): δ = 8.30 (d, *J* = 8.3 Hz, 2H), 7.87 (d, *J* = 8.1 Hz, 2H), 7.80 (d, *J* = 8.3 Hz, 2H), 7.55 (ddd, *J* = 8.3, 6.8, 1.4 Hz, 2H), 7.48 (ddd, *J* = 8.1, 6.9, 1.3 Hz, 2H), 7.35 (d, *J* = 8.4 Hz, 2H), 7.17 (t, *J* = 1.8 Hz, 2H), 7.05 (d, *J* = 1.9 Hz, 4H), 5.80 (d, *J* = 8.0 Hz, 2H), 5.09 (ddd, *J* = 8.1, 7.0, 1.8 Hz, 2H), 3.49 (d, *J* = 14.3 Hz, 2H), 3.29 (dd, *J* = 17.8, 7.1 Hz, 2H), 3.18 (d, *J* = 14.3 Hz, 2H), 2.85 (d, *J* = 18.0 Hz, 2H), 1.16 (s, 36H); ^{13}C NMR (101 MHz, $CDCl_3$): δ = 167.2, 150.0, 137.5, 137.0, 136.4, 133.3, 130.6, 129.3, 128.2, 126.6, 125.5, 125.4, 124.7, 123.1, 120.1, 82.4, 76.4, 48.5, 40.6, 39.6, 34.7, 31.6. IR (ATR): ṽ = 2951 (s), 2903 (m), 2864 (w), 16464 (s), 1361 (m), 1249 (m), 1020 (s), 790 (s), 771 (m), 714 (m) cm^{-1}; HRMS (ESI^+) for $C_{59}H_{67}N_2O_2$ $[M + H^+]^+$: calcd: 835.51970, found: 835.52085.

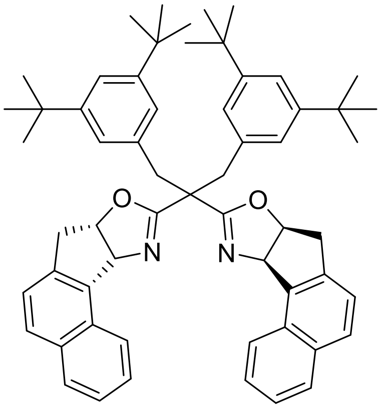

(3a*S*,3a'*S*,8a*R*,8a'*R*)-2,2'-(1,3-Bis(3,5-di-*tert*-butylphenyl)propane-2,2-diyl)bis(5-bromo-3a,8a-dihydro-8*H*-indeno[1,2-d]oxazole) (L12). Prepared analogously as a colorless solid (421 mg, 96 % yield). $[\alpha]_D^{20}$ = –334.9 (c = 0.47, $CHCl_3$); ^1H NMR (400 MHz, $CDCl_3$): δ = 7.64 (d, *J* = 1.8 Hz, 2H), 7.39 (dd, *J* = 8.1, 1.9 Hz, 2H), 7.24 (t, *J* = 1.8 Hz, 2H), 7.07 (q, *J* = 3.2 Hz, 6H), 5.35 (d, *J* = 8.0 Hz, 2H), 5.06 (ddd, *J* = 8.0, 7.1, 1.9 Hz, 2H), 3.47 (d, *J* = 14.3 Hz, 2H), 3.21 – 3.09 (m, 4H), 2.60 (d, *J* = 17.8 Hz, 2H), 1.26 (s, 36H); ^{13}C NMR (101 MHz, $CDCl_3$): δ = 167.5, 150.2, 144.2, 138.9, 135.9, 131.6, 129.1, 126.5, 124.5, 121.2, 120.5, 82.7, 76.4, 48.2, 39.2, 39.0, 34.8, 31.7; IR (ATR): ṽ = 2952 (s), 2865 (m), 1645 (s), 1597 (s), 1473 (s), 1361 (s), 1248 (s), 1175 (s), 1018 (s), 806 (s), 726 (s) cm^{-1}; HRMS (ESI^+) for $C_{51}H_{61}N_2O_2(Br^{79})_2$ [M + $H^+]^+$: calcd: 891.30945, found: 891.30943.

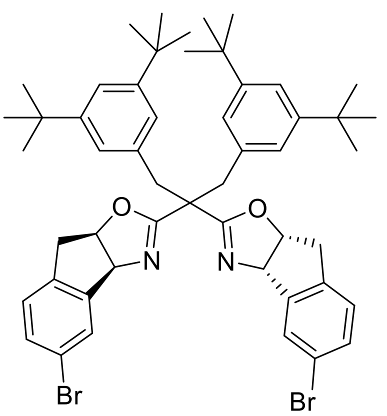

(3a*S*,3a'*S*,8a*R*,8a'*R*)-2,2'-(1,3-Bis(3,5-di-*tert*-butylphenyl)propane-2,2-diyl)bis(5-((3*S*,5*S*,7*S*)-adamantan-1-yl)-3a,8a-dihydro-8H-indeno[1,2-d]oxazole) (**L13**). Prepared analogously as a colorless solid (143 mg, 42 % yield). $[\alpha]_D^{20}$ = –221.8 (c = 0.65, $CHCl_3$); ^1H NMR (400 MHz, $CDCl_3$): δ = 7.53 (d, *J* = 1.8 Hz, 2H), 7.29 (dd, *J* = 8.1, 1.9 Hz, 2H), 7.22 (t, *J* = 1.8 Hz, 2H), 7.15 (d, *J* = 8.2 Hz, 2H), 7.07 (d, *J* = 1.8 Hz, 4H), 5.37 (d, *J* = 8.2 Hz, 2H), 5.11 – 5.05 (m, 2H), 3.51 (d, *J* = 14.3 Hz, 2H), 3.23 – 3.12 (m, 4H), 2.76 – 2.68 (m, 2H), 2.09 (p, *J* = 3.1 Hz, 6H), 1.93 (d, *J* = 2.9 Hz, 11H), 1.83 – 1.71 (m, 13H), 1.24 (s, 36H); ^{13}C NMR (101 MHz, $CDCl_3$): δ = 166.9, 150.9, 150.0, 141.5, 137.0, 136.0, 125.3, 124.6, 124.3, 122.2, 120.3, 82.9, 76.8, 60.4, 48.1, 43.4, 39.3, 39.2, 36.8, 36.3, 34.7, 31.6, 29.0; IR (ATR): ṽ = 2950 (s), 2900

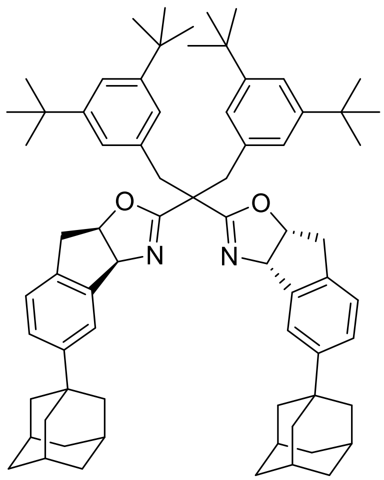

[8] V. L. Rendina, S. A. Goetz, A. E. Neitzel, H. Z. Kaplan, J. S. Kingsbury, *Tetrahedron Lett.* **2012**, *53*, 15.

(s), 2848 (s), 1646 cm^{-1}; HRMS (ESI$^+$) for $C_{71}H_{91}N_2O_2$ [M + H$^+$]$^+$: calcd: 1003.70750, found: 1003.70764.

(3a*S*,3a'*S*,8a*R*,8a'*R*)-2,2'-(1,3-Bbis(3,5-di-*tert*-butylphenyl)propane-2,2-diyl)bis(5-(4-(*tert*-butyl)phenyl)-3a,8a-dihydro-8*H*-indeno[1,2-d]oxazole) (**L16**). Prepared analogously as a colorless solid (115 mg, 51 % yield). $[\alpha]_D^{20}$ = –213.6 (c = 0.49, CHCl$_3$); ^{1}H NMR (400 MHz, CDCl$_3$): δ = 7.79 (s, 2H), 7.63 – 7.54 (m, 6H), 7.51 – 7.45 (m, 4H), 7.30 (d, *J* = 7.9 Hz, 2H), 7.24 (q, *J* = 1.7 Hz, 2H), 7.12 (d, *J* = 1.8 Hz, 4H), 5.44 (d, *J* = 7.9 Hz, 2H), 5.12 (ddd, *J* = 8.0, 7.1, 2.0 Hz, 2H), 3.52 (d, *J* = 14.3 Hz, 2H), 3.26 – 3.16 (m, 4H), 2.71 (d, *J* = 17.9 Hz, 2H), 1.38 (s, 18H), 1.26 (s, 36H); ^{13}C NMR (101 MHz, CDCl$_3$): δ = 167.3, 150.4, 150.2, 142.6, 140.4, 138.9, 138.1, 136.1, 127.3, 126.8, 125.9, 125.3, 124.5, 124.3, 120.5, 83.0, 76.6, 48.2, 41.5, 39.3, 38.6, 34.9, 34.7, 31.7, 31.5; IR (ATR): ṽ = 2959 (s), 2866 (s), 1650 (s), 1598 (m), 1479 (m), 1361 (s), 1248 (s), 1159 (s), 1020 (s), 812 (s), 696 (s), 708 (s), 565 (m) cm^{-1}; HRMS (ESI$^+$) for $C_{71}H_{87}N_2O_2$ [M + H$^+$]$^+$: calcd: 999.67620, found: 999.67621.

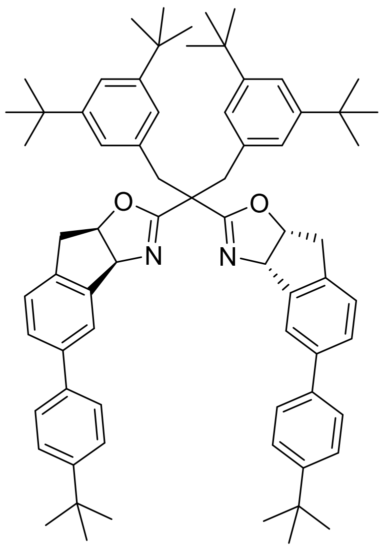

(3a*S*,3a'*S*,8a*R*,8a'*R*)-2,2'-(1,3-Bis(3,5-di-*tert*-butylphenyl)propane-2,2-diyl)bis(5-cyclohexyl-3a,8a-dihydro-8*H*-indeno[1,2-d]oxazole) (**L17**). Prepared analogously as a colorless solid (161 mg, 54 % yield). $[\alpha]_D^{20}$ = –257.1 (c = 0.60, CHCl$_3$); ^{1}H NMR (400 MHz, CDCl$_3$): δ = 7.39 – 7.37 (m, 2H), 7.23 (t, *J* = 1.8 Hz, 2H), 7.15 – 7.10 (m, 4H), 7.07 (d, *J* = 1.8 Hz, 4H), 5.36 (d, *J* = 8.1, 0.7 Hz, 2H), 5.11 – 5.05 (m, 2H), 3.50 (d, *J* = 14.3 Hz, 2H), 3.25 – 3.09 (m, 4H), 2.69 (dd, *J* = 17.6, 2.4 Hz, 2H), 2.57 – 2.46 (m, 2H), 1.95 – 1.70 (m, 10H), 1.50 – 1.31 (m, 10H), 1.25 (s, 36H); ^{13}C NMR (101 MHz, CDCl$_3$): δ = 166.9, 150.0, 147.4, 141.7, 137.3, 136.1, 127.2, 124.8, 124.3, 124.0, 120.3, 82.9, 76.6, 48.0, 44.4, 39.2, 39.0, 34.8, 34.7, 34.5, 31.6, 27.0, 26.2; IR (ATR): ṽ = 2951 (s), 2939 (s), 2852 (s), 1646 (s), 1598 (s), 1448 (s), 1361 (s), 1249 (s), 1170 (s), 1019 (s), 874 (m), 814 (s), 723 (s) cm^{-1}; HRMS (ESI$^+$) for $C_{63}H_{83}N_2O_2$ [M + H$^+$]$^+$: calcd: 899.64490, found: 899.64455.

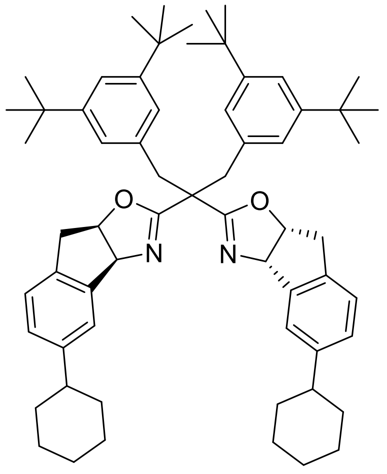

(3a*S*,3a'*S*,8a*R*,8a'*R*)-2,2'-(1,3-Bis(3,5-di-*tert*-butylphenyl)propane-2,2-diyl)bis(5-isopropyl-3a,8a-dihydro-8*H*-indeno[1,2-d]oxazole) (L18). Prepared analogously as a colorless solid (264 mg, 58 % yield).$[\alpha]_D^{20}$ = –223.6 (c = 0.57, CHCl$_3$); ^{1}H NMR (400 MHz, CHCl$_3$): δ = 7.40 (s, 2H), 7.23 (t, *J* = 1.8 Hz, 2H), 7.18 – 7.11 (m, 4H), 7.07 (d, *J* = 1.8 Hz, 4H), 5.37 (d, *J* = 8.1 Hz, 2H), 5.08 (td, *J* = 7.7, 2.4 Hz, 2H), 3.51 (d, *J* = 14.3 Hz, 2H), 3.24 – 3.11 (m, 4H), 2.93 (hept, *J* = 6.9 Hz, 2H), 2.72 (dd, *J* = 17.6, 2.4 Hz, 2H), 1.28 (d, *J* = 2.5 Hz, 6H), 1.26 (d, *J* = 2.5 Hz, 6H), 1.24 (s, 36H); ^{13}C NMR (101 MHz, CHCl$_3$): δ = 167.1, 150.1, 148.3, 141.8, 137.4, 136.2, 127.0, 125.0, 124.4, 123.8, 120.4, 83.0, 76.7, 48.2, 39.3, 34.8, 34.1, 31.7, 24.5, 24.1; IR (ATR): ṽ = 2959 (s), 2867 (m), 1651 (s), 1362 (s), 1249 (s), 1165 (s), 1020 (s), 820 (s), 689 (s) cm^{-1}; HRMS (ESI$^+$) for $C_{57}H_{75}N_2O_2$ [M + H$^+$]$^+$: calcd: 819.58230, found: 819.58270.

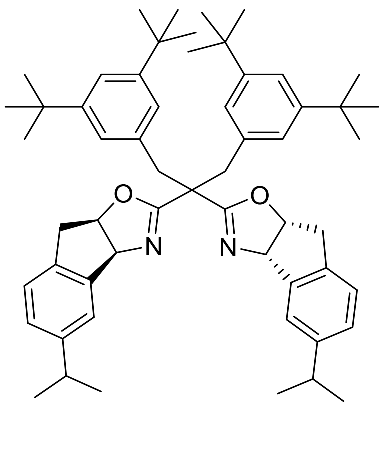

(3a*S*,3a'*S*,8a*R*,8a'*R*)-2,2'-(1,3-Bis(3,5-di-*tert*-butylphenyl)propane-2,2-diyl)bis(5-(2,4,6-triisopropylphenyl)-3a,8a-dihydro-8*H*-indeno[1,2-d]oxazole) (L15). A flame-dried 25 mL 2-necked round bottom flask equipped with a condensor was charged with bromide **L12** (44.1 mg, 0.049 mmol), 2,4,6-triisopropylphenyl boronic acid (37.6 mg, 0.152 mmol), caesium carbonate (76.0 mg, 0.233 mmol) and Pd(dppf)Cl_2 (7.59 mg, 0.010 mmol). A mixture of degassed THF and water (6:1, 5 mL) was added and the resulting suspension was stirred at reflux temperature overnight. Water (5 mL) was introduced and the mixture was extracted with CH_2Cl_2 (3 x, 5 mL). The combined organic layers were dried over $MgSO_4$ and concentrated, and the residue was purified by flash chromatography (silica, hexanes/EtOAc = 30/1) to give the title compound as a colorless solid (54.2 mg, 96 % yield). $[\alpha]_D^{20}$ = –275.0 (c = 0.54, $CHCl_3$); ^1H NMR (400 MHz, $CDCl_3$): δ = 7.37 (d, *J* = 7.7 Hz, 2H), 7.32 – 7.29 (m, 4H), 7.23 – 7.16 (m, 6H), 7.09 (s, 4H), 5.41 (d, *J* = 7.1 Hz, 2H), 5.15 (t, *J* = 6.6 Hz, 2H), 3.48 (d, *J* = 14.2 Hz, 2H), 3.20 – 3.07 (m, 4H), 2.97 (hept, *J* = 6.9 Hz, 2H), 2.84 – 2.60 (m, 4H), 2.50 (d, *J* = 17.7 Hz, 2H), 1.35 (s, 36H), 1.34-1.32 (m, 12H), 1.31-1.25 (m, 6H), 1.23 – 1.15 (m, 12H), 1.08 (d, *J* = 6.8 Hz, 6H); ^{13}C NMR (101 MHz, $CDCl_3$): δ = 167.5, 150.3, 147.9, 146.9, 146.3, 142.4, 140.1, 137.9, 137.1, 136.3, 129.8, 127.4, 124.8, 124.4, 120.71, 120.68, 120.6, 83.4, 76.3, 48.2, 38.5, 36.5, 34.9, 34.4, 31.8, 30.6, 30.4, 24.9, 24.5, 24.23, 24.20, 24.17; IR (ATR): ṽ = 2967 (s), 2867 (m), 1650 (m), 1460 (m), 1361 (m), 1170 (m), 1016 (m), 875 (m), 714 (m) cm^{-1}; HRMS (ESI^+) for $C_{81}H_{107}N_2O_2$ $[M + H^+]^+$: calcd: 1139.83270, found: 1139.83197.

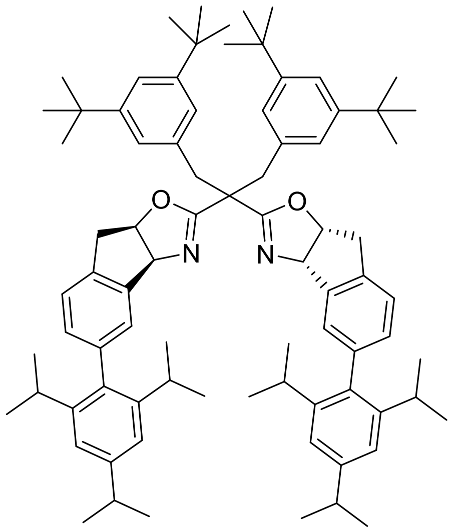

(3a*S*,3a'*S*,8a*R*,8a'*R*)-2,2'-(1,3-Bis(3,5-di-*tert*-butylphenyl)propane-2,2-diyl)bis(5-(anthracen-9-yl)-3a,8a-dihydro-8*H*-indeno[1,2-d]oxazole) (L14). Prepared analogously using 9-anthracenylboronic acid as the reagent; colorless solid (45.1 mg, 84 %). $[\alpha]_D^{20}$ = –264.8 (c = 0.39, $CHCl_3$); ^1H NMR (400 MHz, $CDCl_3$): δ = 8.53 (s, 2H), 8.08 (dd, *J* = 7.7, 1.7 Hz, 4H), 7.90 – 7.86 (m, 2H), 7.80 – 7.76 (m, 2H), 7.68 (s, 2H), 7.55 – 7.42 (m, 10H), 7.41 – 7.35 (m, 2H), 7.31 (q, *J* = 1.4 Hz, 2H), 7.20 (t, *J* = 1.3 Hz, 4H), 5.58 (d, *J* = 7.5 Hz, 2H), 5.42 – 5.35 (m, 2H), 3.67 (d, *J* = 14.6 Hz, 2H), 3.40 (dd, *J* = 17.8, 6.7 Hz, 2H), 3.30 (d, *J* = 14.6 Hz, 2H), 2.93 (d, *J* = 18.2 Hz, 2H), 1.34 (d, *J* = 1.0 Hz, 36H); ^{13}C NMR (101 MHz, $CDCl_3$): δ = 167.7, 150.3, 142.4, 139.3, 137.9, 137.1, 136.2, 131.7, 131.6, 131.5, 130.5, 130.3, 129.1, 128.6, 128.5, 127.2, 126.9, 126.8, 125.42, 125.38, 125.2 (2x), 125.0, 124.4, 120.6, 83.4, 76.7, 48.0, 39.4, 37.6, 34.9, 31.8; IR (ATR): ṽ = 2951 (m), 1646 (m), 1360 (m), 1248 (m), 1014 (m), 843 (m), 718 (s), 714 (s), 606 (m) cm^{-1}; HRMS (ESI^+) for $C_{79}H_{79}N_2O_2$ $[M + H^+]^+$: calcd: 1087.61360, found: 1087.61313.

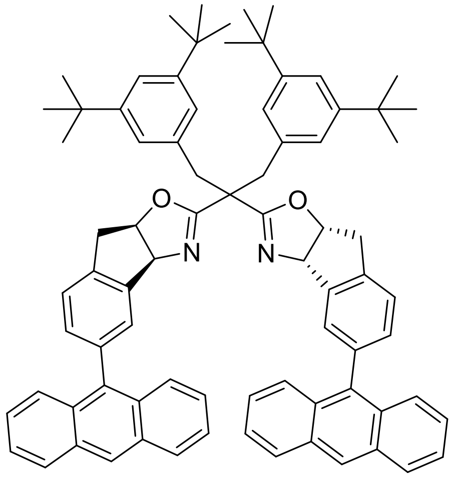

Additional New Ligands for Screening Purposes (cf. Figure S7)

(3a*S*,3a'*S*,8a*R*,8a'*R*)-2,2'-(1,3-Bis(4-(*tert*-butyldiphenylsilyl)phenyl)propane-2,2-diyl)bis(5-isopropyl-3a,8a-dihydro-8*H*-indeno[1,2-d]oxazole). Prepared analogously as a colorless solid (48.0 mg, 68 % yield).

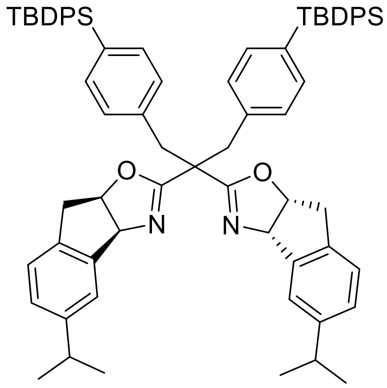

$[\alpha]_D^{20}$ = –228.7 (c = 0.48, $CHCl_3$); ^{1}H NMR (400 MHz, $CDCl_3$): δ = 7.56 – 7.47 (m, 6H), 7.42 – 7.29 (m, 16H), 7.18 – 7.09 (m, 4H), 7.06 – 6.95 (m, 4H), 6.77 (d, *J* = 8.0 Hz, 4H), 5.54 (d, *J* = 7.9 Hz, 2H), 5.24 (ddd, *J* = 8.2, 6.9, 1.6 Hz, 2H), 3.32 – 3.16 (m, 6H), 2.90 (d, *J* = 17.1 Hz, 2H), 2.78 (hept, *J* = 6.9 Hz, 2H), 1.14 (dd, *J* = 6.9, 2.6 Hz, 12H), 1.11 (s, 18H); ^{13}C NMR (101 MHz, $CDCl_3$): δ = 167.5, 148.5, 141.6, 137.6, 137.2, 136.6, 136.1, 135.1, 132.3, 129.7, 129.2, 127.7, 127.1, 125.0, 123.4, 83.7, 76.7, 47.4, 39.3, 39.0, 33.9, 28.9, 24.3, 24.2, 18.8; ^{29}Si NMR (79 MHz, $CDCl_3$): δ = –6.38; IR (ATR): ṽ = 2958 (w), 1643 (m), 1427 (m), 1103 (s), 818 (m), 723 (s), 601 (m), 517 (s) cm^{-1}; HRMS (ESI$^+$) for $C_{73}H_{79}N_2O_2Si_2$ [M + H$^+$]$^+$: calcd: 1071.56746, found: 1071.56836.

(3a*S*,3a'*S*,8a*R*,8a'*R*)-2,2'-(1-(4-(2l5,8l3-Octan-2-yl)phenyl)-3-(4-octylphenyl)propane-2,2-diyl)bis(3a,8a-dihydro-8*H*-indeno[1,2-d]oxazole). Prepared analogously as a colorless solid (181 mg, 77 % yield).

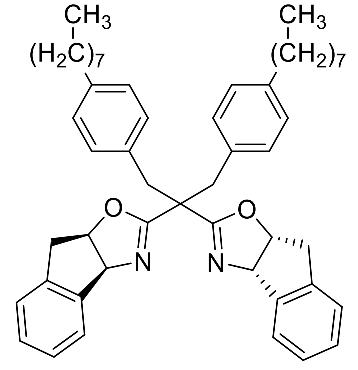

$[\alpha]_D^{20}$ = –197.8 (c = 0.60, $CHCl_3$); ^{1}H NMR (400 MHz, $CDCl_3$): δ = 7.45 (d, *J* = 7.1 Hz, 2H), 7.37 – 7.31 (m, 2H), 7.31 – 7.25 (m, 4H), 6.69 (d, *J* = 8.1 Hz, 4H), 6.63 (d, *J* = 8.1 Hz, 4H), 5.58 (d, *J* = 7.8 Hz, 2H), 5.28 (ddd, *J* = 8.0, 6.8, 1.4 Hz, 2H), 3.32 (dd, *J* = 18.0, 6.8 Hz, 2H), 3.20 (d, *J* = 14.3 Hz, 2H), 3.08 – 2.98 (m, 4H), 2.42 (m, 4H), 1.56 – 1.44 (m, 4H), 1.33 – 1.24 (m, 20H), 0.94 – 0.85 (m, 6H); ^{13}C NMR (101 MHz, $CDCl_3$): δ = 167.6, 141.7, 140.8, 140.0, 133.4, 130.2, 128.5, 127.8, 127.6, 125.9, 125.2, 83.4, 76.6, 47.8, 39.5, 38.2, 35.6, 32.1, 31.5, 29.7, 29.6, 29.4, 22.8, 14.3; IR (ATR): ṽ = 2923 (s), 2853 (s), 1645 (s), 1459 (m), 1159 (s), 1013 (s), 855 (s), 727 (s), 612 (m) cm^{-1}; HRMS (ESI$^+$) for $C_{51}H_{63}N_2O_2$ [M + H$^+$]$^+$: calcd: 735.48840, found: 735.48901.

(3a*S*,3a'*S*,8a*R*,8a'*R*)-2,2'-(Tricosane-12,12-diyl)bis(3a,8a-dihydro-8*H*-indeno[1,2-d]oxazole). Prepared analogously as a colorless solid (184 mg, 99 % yield).

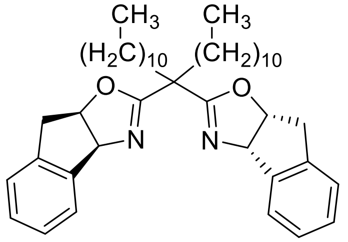

$[\alpha]_D^{20}$ = –197.8 (c = 0.59, $CHCl_3$); ^{1}H NMR (400 MHz, $CDCl_3$): δ = 7.52 – 7.46 (m, 2H), 7.28 – 7.17 (m, 6H), 5.53 (d, *J* = 7.6 Hz, 2H), 5.21 (ddd, *J* = 7.9, 6.8, 1.5 Hz, 2H), 3.28 (dd, *J* = 17.9, 6.7 Hz, 2H), 2.95 (d, *J* = 17.8 Hz, 2H), 1.91 – 1.75 (m, 4H), 1.36 – 0.97 (m, 34H), 0.88 (t, *J* = 7.0 Hz, 6H), 0.77 – 0.63 (m, 2H); ^{13}C NMR (101 MHz, $CDCl_3$): δ = 168.3, 142.2, 139.6, 128.4, 127.5, 125.7, 125.1, 82.9, 76.4, 45.7, 39.7, 32.1, 31.7, 29.8, 29.7, 29.7, 29.5, 29.5, 29.4, 23.3, 22.8, 14.3; IR (ATR): ṽ = 2953 (m), 2945 (s), 2849 (s), 1642 (s), 1004 (s), 848 (m), 728 (s), 715 (m), 623 (m) cm^{-1}; HRMS (ESI$^+$) for $C_{43}H_{63}N_2O_2$ [M + H$^+$]$^+$: calcd: 639.48840, found: 639.48938.

(3a*S*,3a'*S*,8a*R*,8a'*R*)-2,2'-(1,3-Bis(4-(triisopropylsilyl)phenyl)propane-2,2-diyl)bis(3a,8a-dihydro-8*H*-indeno[1,2-d]oxazole).

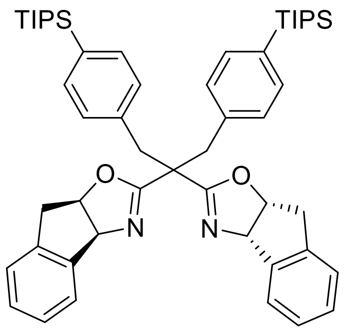

Prepared analogously as a colorless solid (111 mg, 74 % yield). $[\alpha]_D^{20}$ = –201.7 (c = 0.59, $CHCl_3$); ^1H NMR (400 MHz, $CDCl_3$): δ = 7.56 – 7.49 (m, 2H), 7.37 – 7.27 (m, 6H), 6.93 (d, *J* = 8.1 Hz, 4H), 6.60 (d, *J* = 8.0 Hz, 4H), 5.64 (d, *J* = 7.8 Hz, 2H), 5.31 (ddd, *J* = 7.9, 6.7, 1.3 Hz, 2H), 3.35 (dd, *J* = 18.1, 6.7 Hz, 2H), 3.24 (d, *J* = 14.4 Hz, 2H), 3.10 (d, *J* = 18.0 Hz, 2H), 3.00 (d, *J* = 14.4 Hz, 2H), 1.29 (m, 6H), 1.03 (dd, *J* = 7.5, 3.5 Hz, 36H); ^{13}C NMR (101 MHz, $CDCl_3$): δ = 167.8, 141.6, 140.0, 136.4, 134.7, 132.3, 129.6, 128.5, 127.7, 126.0, 125.3, 83.6, 76.7, 47.4, 39.5, 38.3, 18.8, 18.8, 10.9; ^{29}Si NMR (79 MHz, $CDCl_3$): δ = 1.2; IR (ATR) ṽ 2941 (m), 2863 (m), 1645 (m), 1460 (m), 1160 (m), 1101 (m), 1013 (s), 881 (s), 726 (s), 661 (s), 510 (s) cm^{-1}; HRMS (ESI^+) for $C_{53}H_{71}N_2O_2Si_2$ $[M + H^+]^+$: calcd: 823.50486, found: 823.50383.

(3a*S*,3a'*S*,8a*R*,8a'*R*)-2,2'-(1,3-Bis(4-(*tert*-butyldiphenylsilyl)phenyl)propane-2,2-diyl)bis(3a,8a-dihydro-8*H*-indeno[1,2-d]oxazole).

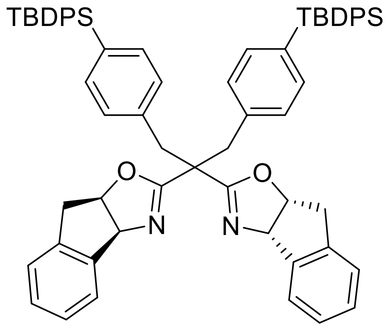

Prepared analogously as a colorless solid (155 mg, 86 % yield). $[\alpha]_D^{20}$ = –198.5 (c = 0.64, $CHCl_3$); ^1H NMR (400 MHz, $CDCl_3$): δ = 7.55 – 7.48 (m, 8H), 7.45 – 7.32 (m, 14H), 7.12 (dd, *J* = 6.3, 2.4 Hz, 2H), 7.04 – 6.94 (m, 8H), 6.66 – 6.58 (m, 4H), 5.62 (d, *J* = 7.7 Hz, 2H), 5.31 (ddd, *J* = 7.7, 6.6, 1.2 Hz, 2H), 3.37 – 3.24 (m, 4H), 3.09 (d, *J* = 17.9 Hz, 2H), 3.02 (d, *J* = 14.3 Hz, 2H), 1.11 (s, 18H); ^{13}C NMR (101 MHz, $CDCl_3$): δ = 167.6, 141.4, 139.8, 137.1, 136.7, 136.0, 135.2, 135.1, 132.2, 129.7, 129.2, 128.6, 127.7, 127.7, 125.8, 125.2, 83.8, 76.6, 47.3, 39.4, 38.7, 29.0, 18.8; ^{29}Si NMR (99 MHz, $CDCl_3$): δ = –6.3; IR (ATR) ṽ 2929 (w), 1644 (m), 1427 (m), 1103 (s), 1013 (m), 742 (m), 683 (s), 601 (m), 475 (s) cm^{-1}; HRMS (ESI^+) for $C_{67}H_{67}N_2O_2Si_2$ $[M + H^+]^+$: calcd: 987.47356, found: 987.47444.

(4*R*,4'*R*,5*S*,5'*S*)-2,2'-(Cyclopropane-1,1-diyl)bis(4,5-diphenyl-4,5-dihydrooxazole). Prepared analogously

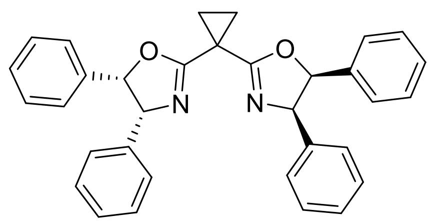

from 2,2′-methylenebis[(4*R*,5*S*)-4,5-diphenyl-2-oxazoline] (104 mg, 0.226 mmol) as a colorless solid (105 mg, 99 % yield). $[\alpha]_D^{20}$ = +163.9 (c = 0.53, $CHCl_3$); ^1H NMR (400 MHz, $CDCl_3$): δ = 7.04 – 6.94 (m, 20H), 5.96 (d, *J* = 10.2 Hz, 2H), 5.60 (d, *J* = 10.2 Hz, 2H), 1.85 – 1.73 (m, 4H). ^{13}C NMR (101 MHz, $CDCl_3$) δ 167.2, 137.8, 136.5, 128.0, 127.8, 127.7, 127.5, 127.0, 126.7, 86.2, 74.0, 19.0, 16.0; IR (ATR): ṽ = 3030 (w), 1662 (s), 1453 (m), 1165 (m), 1103 (m), 718 (s), 583 (m) cm^{-1}; HRMS (ESI^+) for $C_{33}H_{29}N_2O_2$ $[M + H^+]^+$: calcd: 485.22235, found: 485.22276.

(4*R*,4'*R*,5*S*,5'*S*)-2,2'-(1,3-Bis(4-(*tert*-butyl)phenyl)propane-2,2-diyl)bis(4,5-diphenyl-4,5-dihydrooxazole).

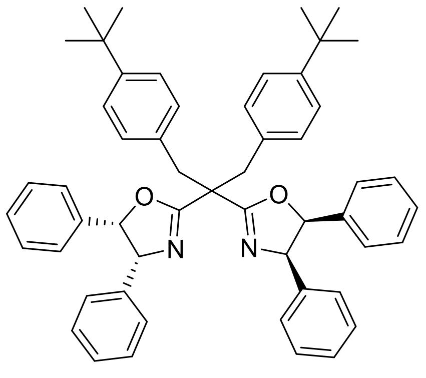

Prepared analogously as a colorless solid (145 mg, 87 % yield). $[\alpha]_D^{20}$ = +310.2 (c = 0.52, $CHCl_3$); ^1H NMR (400 MHz, $CDCl_3$): δ = 7.49 – 7.40 (m, 8H), 7.07 – 6.85 (m, 20H), 5.79 (d, *J* = 10.2 Hz, 2H), 5.45 (d, *J* = 10.2 Hz, 2H), 3.86 (d, *J* = 14.1 Hz, 2H), 3.54 (d, *J* = 14.1 Hz, 2H), 1.39 (s, 18H); ^{13}C NMR (101 MHz, $CDCl_3$): δ = 168.2, 149.8, 137.5, 135.9, 133.9, 130.6, 128.1, 127.6 (2x), 127.5, 127.1, 127.0, 125.2, 86.3, 73.7, 49.5, 39.9, 34.6, 31.6; IR (ATR): ṽ = 2960 (w), 1649 (m), 1453 (m), 1168 (m), 714 (s), 600 (m) cm^{-1}; HRMS (ESI^+) for $C_{53}H_{55}N_2O_2$ $[M + H^+]^+$: calcd: 751.42580, found: 751.42675. The spectral data is consistent with previously reported values.[9]

(4*R*,4'*R*,5*S*,5'*S*)-2,2'-(1,3-Bis(3,5-di-*tert*-butylphenyl)propane-2,2-diyl)bis(4,5-diphenyl-4,5-dihydrooxazole).

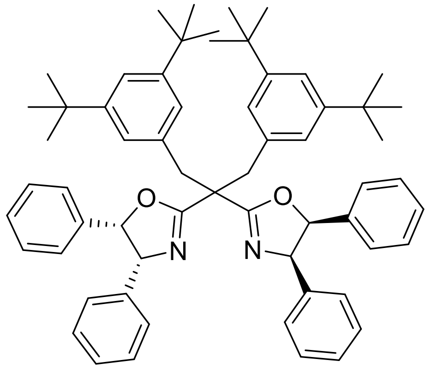

Prepared analogously as a colorless solid (125 mg, 67 % yield). $[\alpha]_D^{20}$ = +290.2 (c = 0.55, $CHCl_3$); ^1H NMR (400 MHz, $CDCl_3$): δ = 7.39 (s, 6H), 7.06 – 6.83 (m, 20H), 5.72 (d, *J* = 10.0 Hz, 2H), 5.39 (d, *J* = 10.0 Hz, 2H), 3.93 (d, *J* = 14.3 Hz, 2H), 3.66 (d, *J* = 14.3 Hz, 2H), 1.37 (s, 36H); ^{13}C NMR (101 MHz, $CDCl_3$): δ = 168.5, 150.5, 137.3, 136.3, 135.8, 128.1, 127.8, 127.7, 127.5, 127.0, 126.9, 124.9, 121.0, 86.4, 73.8, 49.2, 40.7, 35.0, 31.8; IR (ATR) : ṽ = 2960 (m), 1650 (w), 1454 (w), 1770 (w), 715 (m), 701 (s), 590 (w) cm^{-1}; HRMS (ESI^+) for $C_{61}H_{71}N_2O_2$ $[M + H^+]^+$: calcd: 863.55238, found: 863.55100. The spectral data is consistent with previously reported values.[9]

(4*S*,4'*S*)-2,2'-(1,3-Bis(3,5-di-*tert*-butylphenyl)propane-2,2-diyl)bis(4-*iso*-propyl-4,5-dihydrooxazole).

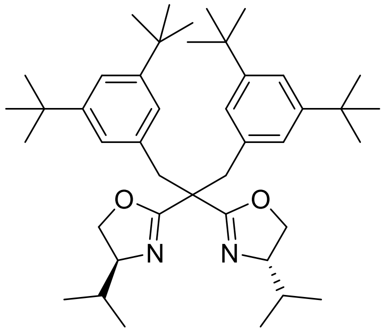

Prepared analogously from bis-[(*S*)-4-*iso*-propyl-4,5-dihydrooxazol-2-yl]-methane as a colorless solid (177 mg, 83 % yield). $[\alpha]_D^{20}$ = –73.1 (c = 0.50, $CHCl_3$); ^1H NMR (400 MHz, $CDCl_3$): δ = 7.26 (t, *J* = 1.8 Hz, 2H), 7.17 (d, *J* = 1.9 Hz, 4H), 4.17 – 4.08 (m, 2H), 3.92 – 3.78 (m, 4H), 3.52 (d, *J* = 14.3 Hz, 2H), 3.30 (d, *J* = 14.3 Hz, 2H), 1.60 (h, *J* = 6.7 Hz, 2H), 1.30 (s, 36H), 0.86 (d, *J* = 6.8 Hz, 6H), 0.77 (d, *J* = 6.8 Hz, 6H); ^{13}C NMR (101 MHz, $CDCl_3$): δ = 166.7, 150.1, 136.4, 124.7, 120.6, 72.1, 69.8, 48.2, 39.5, 34.9, 32.6, 31.7, 19.2, 17.8; IR (ATR): ṽ = 2954 (s), 2901 (m), 2870 (m), 1667 (s), 1597 (s), 1468 (s), 1362 (s), 1176 (s), 1023 (m), 694 (m), 913 (m), 711 (s), 699 (s) cm^{-1}; HRMS (ESI^+) for $C_{43}H_{67}N_2O_2$ $[M + H^+]^+$: calcd: 643.51970, found: 643.52036. The spectral data is consistent with previously reported values.[10]

---

9 X.-Q. Mou, F.-M. Rong, H. Zhang, G. Chen, G. He, *Org. Lett.* **2019**, *21*, 4657.

10 T. Sawada, M. Nakada, *Tetrahedron: Asymmetry* **2012**, *23*, 350.

(4*S*,4'*S*)-2,2'-(1,3-Bis(3,5-di-*tert*-butylphenyl)propane-2,2-diyl)bis(4-(*tert*-butyl)-4,5-dihydrooxazole).

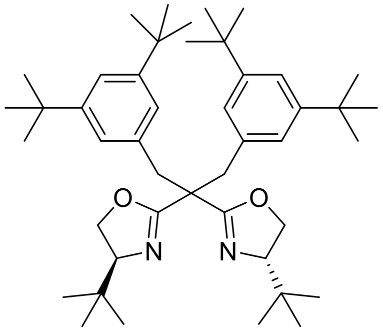

Prepared analogously from bis-[(*S*)-4-*iso*-propyl-4,5-dihydrooxazol-2-yl]-methane as a colorless solid (177 mg, 91 % yield). $[\alpha]_D^{20}$ = –81.4 (c = 0.55, $CHCl_3$); ^{1}H NMR (400 MHz, $CDCl_3$): δ = 7.25 (t, *J* = 1.8 Hz, 2H), 7.14 (d, *J* = 1.8 Hz, 4H), 4.07 (dd, *J* = 10.2, 8.6 Hz, 2H), 4.01 – 3.92 (m, 2H), 3.78 (dd, *J* = 10.1, 7.5 Hz, 2H), 3.54 (d, *J* = 14.4 Hz, 2H), 3.26 (d, *J* = 14.5 Hz, 2H), 1.29 (s, 36H), 0.83 (s, 18H); ^{13}C NMR (101 MHz, $CDCl_3$): δ = 166.8, 150.1, 136.6, 124.7, 120.5, 75.9, 68.6, 48.1, 39.2, 34.9, 34.1, 31.8, 26.0; IR (ATR): ṽ = 2934 (s), 2902 (s), 2867 (s), 1658 (s), 1598 (s), 1477 (s), 1362 (s), 1248 (m), 1175 (s), 1015 (s), 969 (m), 912 (m), 874 (m), 714 (s) cm^{-1}; HRMS (ESI$^+$) for $C_{45}H_{71}N_2O_2$ [M + H$^+$]$^+$: calcd: 671.55100, found: 671.55043.

SUBSTRATES

***tert*-Butyl 2-phenylacetate (1b).** The compound was prepared by adaptation of a literature procedure.[11] Dry

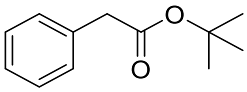

tert-BuOH (2 mL, 20.9 mmol) was mixed with THF (40 mL) in a flame-dried Schlenk flask. The solution was cooled to 0 °C before *n*-butyllithium (1.6 M in hexanes, 12.6 mL, 20.2 mmol) was added dropwise. The mixture was stirred for 10 min before a solution of methyl phenylacetate (3.62 g, 24.1 mmol) in THF (10 mL) was added dropwise. The cooling bath was removed and the mixture stirred at ambient temperature overnight. Water (30 mL) was added and the mixture extracted with CH_2Cl_2 (3 x, 30 mL). The combined organic layers were dried over $MgSO_4$ and concentrated, and the residue was purified by flash chromatography (silica, pentane/*tert*-butyl methyl ether = 30/1) to give the title compound as a colorless oil (3.87 g, 93 % yield). ^{1}H NMR (400 MHz, $CDCl_3$): δ = 7.35 – 7.23 (m, 5H), 3.53 (s, 2H), 1.44 (s, 9H); ^{13}C NMR (101 MHz, $CDCl_3$): δ = 171.1, 134.9, 129.3, 128.6, 127.0, 80.9, 42.8, 28.2. IR (ATR) ṽ 2983 (w), 1728 (s), 1371 (m), 1135 (s), 744 (s), 691 (s), 532 (w) cm^{-1}; HRMS (GC-CI, Isobutane) for $C_{12}H_{17}O_2$ $[M + H^+]^+$: calcd: 193.12231, found: 193.12207. The spectral data is consistent with previously reported values.[11]

***tert*-Butyl 2-(4-bromophenyl)acetate (S15).** Prepared analogously from methyl 2-(4-bromophenyl)acetate

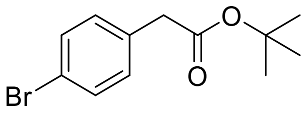

(2.36 g, 10.3 mmol) as a colorless oil (2.09 g, 75 % yield). ^{1}H NMR (400 MHz, $CDCl_3$): δ = 7.46 – 7.41 (m, 2H), 7.17 – 7.12 (m, 2H), 3.47 (s, 2H), 1.43 (s, 9H); ^{13}C NMR (101 MHz, $CDCl_3$): δ = 170.5, 133.8, 131.7, 131.1, 121.0, 81.2, 42.2, 28.2; IR (ATR): ṽ = 2978 (w), 1729 (s), 1488 (m), 1367 (m), 1255 (m), 1136 (s), 1012 (m), 803 (m), 493 (m) cm^{-1}; HRMS (GC-CI, Isobutane) for $C_{12}H_{16}O_2Br$ $[M + H^+]^+$: calcd: 271.032830 found: 271.03276. The spectral data is consistent with previously reported values.[12]

***tert*-Butyl 2-(4-chlorophenyl)acetate (S16).** Prepared analogously from from methyl 2-(4-

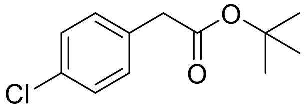

chlorophenyl)acetate (1.09 g, 5.91 mmol) as a colorless oil (832 mg, 62 % yield). ^{1}H NMR (400 MHz, $CDCl_3$): δ = 7.31 – 7.26 (m, 2H), 7.23 – 7.17 (m, 2H), 3.49 (s, 2H), 1.43 (s, 9H); ^{13}C NMR (101 MHz, $CDCl_3$): δ = 170.5, 133.2, 132.8, 130.6, 128.6, 81.1, 42.0, 28.0. IR (ATR): ṽ = 2981 (w), 1730 (s), 1492 (m), 1139 (s), 1090 (s), 1020 (m), 808 (m), 504 (m) cm^{-1}; HRMS (ESI^+) for $C_{12}H_{15}O_2ClNa$ $[M + Na^+]^+$: calcd: 249.06528, found: 249.06483. The spectral data is consistent with previously reported values.[13]

***tert*-Butyl 2-(4-fluorophenyl)acetate (S17).** Prepared analogously from methyl 2-(4-fluorophenyl)acetate

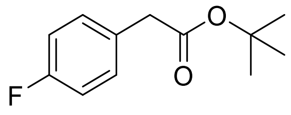

(3.20 g, 19.1 mmol) as a colorless oil (3.43 g, 86 % yield). ^{1}H NMR (400 MHz, $CDCl_3$): δ = 7.26 – 7.20 (m, 2H), 7.04 – 6.96 (m, 2H), 3.49 (s, 2H), 1.43 (s, 9H); ^{13}C NMR (101 MHz, $CDCl_3$): δ = 170.9, 162.0 (d, *J* = 244.9 Hz), 130.9 (d, *J* = 8.1 Hz), 130.5 (d, *J* = 3.5 Hz), 115.4 (d, *J* = 21.1 Hz), 81.1, 41.9, 28.2; ^{19}F NMR (282 MHz, $CDCl_3$): δ = –116.3. IR (ATR): ṽ = 2981 (w), 1732 (s), 1507 (s), 1366 (m), 1212 (s), 1137 (s), 956 (w), 835 (w), 797 (w), 519 (w) cm^{-1}; HRMS

---

11 B. M. Bhawal, S. P. Khanapure, E. R. Biehl, *Synthesis* **1991**, *1991*, 112.

12 A. J. Boddy, D. P. Affron, C. J. Cordier, E. L. Rivers, A. C. Spivey, J. A. Bull, *Angew. Chem. Int. Ed.* **2019**, *58*, 1458.

13 I. Abdiaj, L. Huck, J. M. Mateo, A. de La Hoz, M. V. Gomez, A. Díaz-Ortiz, J. Alcázar, *Angew. Chem. Int. Ed.* **2018**, *57*, 13231.

(GC-EI) for $C_{12}H_{15}O_2F$ $[M^+]^+$: calcd: 210.10506, found: 210.10508. The spectral data is consistent with previously reported values.[14]

***tert*-Butyl 2-(3,5-difluorophenyl)acetate (S18).** Prepared analogously from methyl 2-(3,5-difluorophenyl)acetate (1.16 g, 6.23 mmol) as a colorless oil (936 mg, 66 % yield).

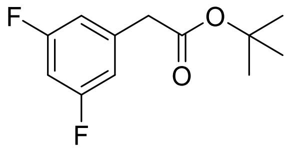

^{1}H NMR (400 MHz, $CDCl_3$): δ = 6.84 – 6.77 (m, 2H), 6.71 (tt, *J* = 9.0, 2.3 Hz, 1H), 3.50 (s, 2H), 1.45 (s, 9H). ^{13}C NMR (101 MHz, $CDCl_3$): δ = 169.6, 162.9 (dd, *J* = 248.0, 12.8 Hz), 138.2 (t, *J* = 9.7 Hz), 112.5 – 112.0 (m), 102.5 (t, *J* = 25.2 Hz), 81.5, 42.2 (t, *J* = 2.0 Hz), 28.0; ^{19}F NMR (282 MHz, $CDCl_3$): δ = –110.3. IR (ATR): ṽ = 2983 (w), 1730 (s), 1626 (s), 1598 (s), 1461 (m), 1371 (m), 1315 (s), 1126 (s), 993 (s), 843 (m), 671 (m), 508 (m) cm^{-1}; HRMS (GC-CI, Isobutane) for $C_{12}H_{15}O_2F_2$ $[M + H^+]^+$: calcd: 229.10346, found: 229.10334.

***tert*-Butyl 2-(p-tolyl)acetate (S19).** Prepared analogously from methyl 2-(p-tolyl)acetate (977 mg, 5.95 mmol) as a colorless oil (1.03 g, 84 % yield).

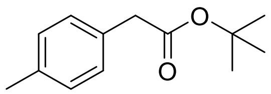

^{1}H NMR (400 MHz, $CDCl_3$): δ = 7.18 – 7.10 (m, 4H), 3.48 (s, 2H), 2.33 (s, 3H), 1.44 (s, 9H); ^{13}C NMR (101 MHz, $CDCl_3$): δ = 171.3, 136.5, 131.3, 129.3, 129.2, 80.8, 42.4, 28.2, 21.2; IR (ATR): ṽ = 2981 (w), 1728 (s), 1518 (m), 1366 (m), 1135 (s), 956 (m), 784 (m), 515 (m), 486 (m) cm^{-1}; HRMS (ESI$^+$) for $C_{13}H_{18}O_2Na$ $[M + Na^+]^+$: calcd: 229.11990, found: 229.11972. The spectral data is consistent with previously reported values.[13]

***tert*-Butyl 2-(2-chlorophenyl)acetate (S20).** Prepared analogously from methyl 2-(2-chlorophenyl)acetate (1.75 g, 9.47 mmol) as a colorless oil (1.65 g, 77 % yield).

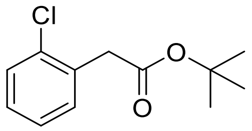

^{1}H NMR (400 MHz, $CDCl_3$): δ = 7.40 – 7.34 (m, 1H), 7.30 – 7.26 (m, 1H), 7.25 – 7.18 (m, 2H), 3.68 (s, 2H), 1.45 (s, 9H); ^{13}C NMR (101 MHz, $CDCl_3$): δ = 170.0, 134.7, 133.3, 131.5, 129.5, 128.6, 126.9, 81.3, 40.6, 28.1; IR (ATR): ṽ = 2980 (w), 1732 (s), 1368 (m), 1227 (m), 1145 (s), 1057 (m), 746 (s), 680 (m), 444 (w) cm^{-1}; HRMS (GC-CI, Isobutane) for $C_{12}H_{16}O_2Cl$ $[M + H^+]^+$: calcd: 227.08333, found: 227.08333. The spectral data is consistent with previously reported values.[15]

***tert*-Butyl 2-(4-(trifluoromethyl)phenyl)acetate (S21).** This compound was prepared by adaptation of a literature procedure.[16]

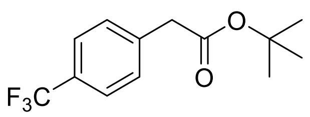

2-(4-(Trifluoromethyl)phenyl)acetic acid (1.30 g, 6.42 mmol) and dry *tert*-BuOH (6.00 mL, 62.7 mmol) were dissolved in CH_2Cl_2 (50 mL) in a flame-dried Schlenk flask. After the addition of pyridine (2.60 mL, 32.1 mmol), phosphoryl trichloride (0.80 mL, 8.58 mmol) was added dropwise. The mixture was stirred for 4 h before aqueous HCl (2 M, 30 mL) was introduced. The mixture was extracted with CH_2Cl_2 (3 x, 40 mL), the combined organic layers were dried over $MgSO_4$ and concentrated, and the crude product was purified by flash chromatography (silica, hexanes/EtOAc = 50/1) to yield the title compound as a colorless oil (1.58 g, 95 % yield). ^{1}H NMR (400 MHz, $CDCl_3$): δ = 7.58 (d, *J* = 8.0 Hz, 2H), 7.39 (d, *J* = 8.0 Hz, 2H), 3.59 (s, 2H), 1.44 (s, 9H); ^{13}C NMR (101 MHz, $CDCl_3$): δ = 170.2, 138.8 (q, *J* = 1.6 Hz), 129.8, 129.5 (q, *J* = 32.5 Hz), 125.5 (q, *J* = 3.8 Hz), 125.2 (q, *J* = 272.0 Hz), 81.5, 42.5, 28.2; ^{19}F NMR (282 MHz, $CDCl_3$): δ = –62.5; IR (ATR): ṽ = 2983 (w), 1730 (s), 1324 (s), 1124 (s), 1068 (s), 1022 (m), 826 (m), 596

---

14 M. Jørgensen, S. Lee, X. Liu, J. P. Wolkowski, J. F. Hartwig, *J. Am. Chem. Soc.* **2002**, *124*, 12557.
15 J. A. Greenberg, T. Sammakia, *J. Org. Chem.* **2017**, *82*, 3245.
16 M. Hu, L. Li, H. Wu, Y. Su, P.-Y. Yang, M. Uttamchandani, Q.-H. Xu, S. Q. Yao, *J. Am. Chem. Soc.* **2011**, *133*, 12009.

(m) cm^{-1}; HRMS (GC-CI, Isobutane) for $C_{13}H_{16}O_2F_3$ [M + H$^+$]$^+$: calcd: 261.10969, found: 261.10982. The spectral data is consistent with previously reported values.[14]

(2-(*tert*-Butoxy)-2-oxoethyl)zinc bromide. This compound was prepared similar to a literature procedure.[17] A 100 mL 2-necked round bottom flask equipped with a condensor was flame-dried and then charged with zinc powder (1.45 g, 22.2 mmol). THF (5 mL) and TMSCl (0.10 mL, 0.79 mmol) were added and the mixture was warmed to 50 °C. A solution of *tert*-butyl bromo acetate (1.50 mL, 10.3 mmol) in THF was added dropwise at this temperature. Once the addition was complete, the mixture was cooled to ambient temperature and stirring was continued for 1.5 h. Excess zinc was allowed to settle and the supernatant was separated via cannula. The resulting yellow solution was directly used in the next step.

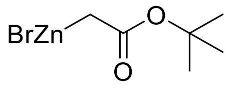

Methyl 4-(2-(*tert*-butoxy)-2-oxoethyl)benzoate (S22). This compound was prepared in analogy to a literature procedure.[18] In a flame-dried Schlenk flask, a solution of methyl 4-bromo benzoate (511 mg, 2.91 mmol), (2-(*tert*-butoxy)-2-oxoethyl)zinc(II) bromide (approx. 2.62 mmol), 1,2,3,4,5-pentaphenyl-1-(di-t-butylphosphino)ferrocene (18.8 mg, 0.027 mmol) and bis-(dibenzylidenaceton)-palladium (18.7 mg, 0.023 mmol) in THF (10 mL) was prepared. The mixture was stirred for 1 h before it was concentrated in the presence of Celite. The loaded Celite was added on top of a flash column filled with silica, eluting the product with hexanes/EtOAc (50/1 – 25/1 gradient) to yield the title compound as a colorless solid (513 mg, 86 % yield). ^{1}H NMR (400 MHz, $CDCl_3$): δ = 8.01 – 7.96 (m, 2H), 7.36 – 7.32 (m, 2H), 3.90 (s, 3H), 3.58 (s, 2H), 1.43 (s, 9H); ^{13}C NMR (101 MHz, $CDCl_3$): δ = 170.3 (s), 167.1 (s), 140.1 (s), 129.9 (s), 129.4 (s), 129.0 (s), 81.4 (s), 52.2 (s), 42.8 (s), 28.1 (s); IR (ATR): ṽ = 1717 (s), 1435 (m), 1393 (m), 1275 (s), 1140 (s), 1105 (s), 1021 (m), 779 (m), 753 (m) cm^{-1}; HRMS (ESI$^+$) for $C_{14}H_{18}O_4Na$ [M + Na$^+$]$^+$: calcd: 273.10973, found: 273.10931.

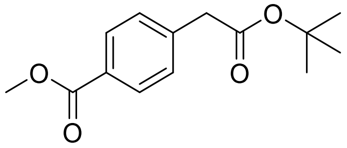

***tert*-Butyl 2-(4-(methylsulfonyl)phenyl)acetate (S23).** In a flame-dried Schlenk flask, 2-(4-(methylsulfonyl)phenyl)acetic acid (1.01g, 4.69 mmol) was dissolved in CH_2Cl_2 (40 mL). Dry *tert*-BuOH (4.50 mL), pyridine (2.30 mL) and phosphoryl chloride (0.60 mL, 6.44 mmol) were added subsequently. The mixture was stirred for 1.5 h at rt. Aqueous HCl (2 M, 40 mL) was added and the mixture was extracted with CH_2Cl_2 (3 x, 30 mL). The combined organic layers were dried over $MgSO_4$ and concentrated, and the crude material was purified by flash chromatography (silica, hexanes/EtOAc = 7/2) to yield the title compound as a colorless solid (1.20 g, 94 % yield). ^{1}H NMR (400 MHz, $CDCl_3$): δ = 7.92 – 7.87 (m, 2H), 7.50 – 7.45 (m, 2H), 3.62 (s, 2H), 3.04 (s, 3H), 1.44 (s, 9H); ^{13}C NMR (101 MHz, $CDCl_3$): δ = 169.8, 141.1, 139.3, 130.4, 127.7, 81.8, 44.7, 42.5, 28.1; IR (ATR): ṽ = 1988 (w), 2922 (w), 1715 (s), 1299 (s), 1237 (m), 1139 (s), 1088 (s), 957 (m), 875 (m), 532 (s) cm^{-1}; HRMS (GC-CI, Isobutane) for $C_{13}H_{19}O_4S$ [M + H$^+$]$^+$: calcd: 271.09982, found: 271.09982.

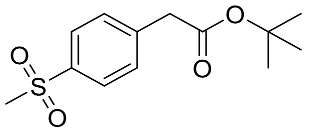

[17] M. Mineno, Y. Sawai, K. Kanno, N. Sawada, H. Mizufune, *J. Org. Chem.* **2013**, *78*, 5843.
[18] T. Hama, S. Ge, J. F. Hartwig, *J. Org. Chem.* **2013**, *78*, 8250.

***tert*-Butyl 2-(3-chlorophenyl)acetate (S24).** Prepared analogously from 2-(3-chlorophenyl)acetic acid as a

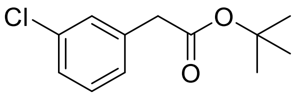

colorless oil (743 mg, 93 % yield). ^{1}H NMR (400 MHz, $CDCl_3$): δ = 7.30 – 7.23 (m, 3H), 7.20 – 7.15 (m, 1H), 3.51 (s, 2H), 1.46 (s, 9H); ^{13}C NMR (101 MHz, $CDCl_3$): δ = 170.4, 136.7, 134.3, 129.8, 129.6, 127.6, 127.2, 81.4, 42.3, 28.2; IR (ATR): ṽ = 2979 (w), 1729 (s), 1574 (w), 1477 (m), 1368 (m), 1229 (m), 1135 (s), 839 (m), 770 (s), 682 (s), 438 (w) cm^{-1}; HRMS (ESI$^+$) for $C_{12}H_{15}O_2ClNa$ [M + Na$^+$]$^+$: calcd: 249.06528, found: 249.06524.

DIAZO COMPOUNDS

Representative Procedure. Preparation of *tert*-Butyl 2-(4-chlorophenyl)-2-diazoacetate (S25). *tert*-Butyl

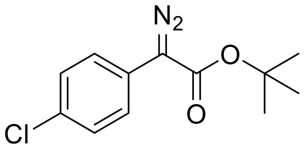

2-(4-chlorophenyl)acetate (**S16**) (629 mg, 2.78 mmol) was dissolved in dry MeCN (20 mL) in a flame-dried Schlenk flask. The solution was cooled to 0 °C in an ice bath before 4-acetamidobenzenesulfonyl azide (1.11 g, 4.61 mmol) was added in portions. The flask was protected from light with aluminum foil and 1,8-diazabicyclo(5.4.0)undec-7-ene (0.80 mL, 5.35 mmol) was added dropwise via syringe. After the addition was complete, the solution was stirred at ambient temperature overnight. Water (20 mL) was added and the mixture was extracted with CH_2Cl_2 (3 x, 40 mL). The combined organic layers were dried over $MgSO_4$, filtered and concentrated to yield an orange solid, which was purified by flash chromatography (silica, pentane/Et_2O = 95/5) to give the title compound as a yellow solid (562 mg, 80 % yield). ^{1}H NMR (400 MHz, $CDCl_3$): δ = 7.43 – 7.38 (m, 2H), 7.35 – 7.31 (m, 2H), 1.55 (s, 9H); ^{13}C NMR (101 MHz, $CDCl_3$): δ = 164.4, 131.3, 129.1, 125.2, 124.9, 82.5, 28.5 (C-N_2 was not observed); IR (ATR): ṽ = 2976 (w), 2083 (s), 1688 (s), 1492 (s), 1344 (s), 1243 (s), 1146 (s), 1000 (s), 810 (s), 744 (m), 499 (s) cm^{-1}; HRMS (ESI$^+$) for $C_{12}H_{13}ClN_2O_2Na$ [M + Na$^+$]$^+$: calcd: 275.05577, found: 275.05536. The spectral data is consistent with previously reported values.[19]

***tert*-Butyl 2-diazo-2-phenylacetate (1b).** Prepared analogously as a red oil (1.58 g, 74 % yield). ^{1}H NMR

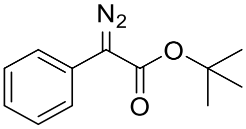

(400 MHz, $CDCl_3$): δ = 7.50 – 7.45 (m, 2H), 7.40 – 7.34 (m, 2H), 7.20 – 7.13 (m, 1H), 1.56 (s, 9H); ^{13}C NMR (101 MHz, $CDCl_3$): δ = 164.7, 129.0, 126.3, 125.7, 124.1, 82.2, 28.6 (C-N_2 was not observed); IR (ATR): ṽ = 2979 (w), 2076 (s), 1690 (s), 1499 (m), 1351 (m), 1245 (s), 1139 (s), 1009 (s), 850 (m), 751 (s), 689 (s), 490 (m) cm^{-1}; HRMS (ESI$^+$) for $C_{12}H_{14}N_2O_2Na$ [M + Na$^+$]$^+$: calcd: 241.09475, found: 241.09469. The spectral data is consistent with previously reported values.[20]

***tert*-Butyl 2-(4-bromophenyl)-2-diazoacetate (S26).** Prepared analogously as a yellow solid (742 mg, 86 %

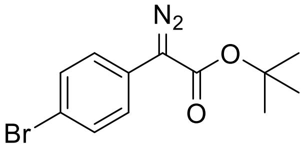

yield). ^{1}H NMR (400 MHz, $CDCl_3$): δ = 7.50 – 7.45 (m, 2H), 7.37 – 7.32 (m, 2H), 1.54 (s, 9H); ^{13}C NMR (101 MHz, $CDCl_3$): δ = 164.3, 132.0, 125.5, 125.5, 119.1, 82.5, 28.5 (C-N_2 was not observed); IR (ATR): ṽ = 2976 (w), 2079 (s), 1686 (s), 1490 (m), 1344 (m), 1243 (s), 1141 (s), 1004 (s), 806 (s), 740 (m), 517 (m), 497

[19] T. Hashimoto, Y. Naganawa, T. Kano, K. Maruoka, *Chem. Commun.* **2007**, 5143.
[20] I. D. Jurberg, H. M. L. Davies, *Org. Lett.* **2017**, *19*, 5158.

(s) cm^{-1}; HRMS (ESI$^+$) for $C_{12}H_{13}N_2O_2BrNa$ [M + Na$^+$]$^+$: calcd: 319.00527, found: 319.00477. The spectral data is consistent with previously reported values.[19]

***tert*-Butyl 2-(3-chlorophenyl)-2-diazoacetate (S27).** Prepared analogously as a yellow solid (310 mg, 84 %).

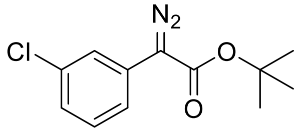

^{1}H NMR (400 MHz, $CDCl_3$): δ 7.57 – 7.54 (m, 1H), 7.32 – 7.25 (m, 2H), 7.14 – 7.09 (m, 1H), 1.55 (s, 9H); ^{13}C NMR (101 MHz, $CDCl_3$): δ = 164.1, 135.1, 130.15, 128.5, 125.6, 123.9, 121.7, 82.6, 28.5 (C-N_2 was not observed); IR (ATR): ṽ = 2980 (w), 2091 (s), 1691 (s), 1593 (m), 1561 (m), 1477 (m), 1356 (s), 1244 (s), 1121 (s), 1121 (s), 1022 (s), 828 (m), 767 (s), 674 (s), 503 (m), 440 (m) cm^{-1}; HRMS (ESI$^+$) for $C_{12}H_{13}N_2O_2ClNa$ [M + Na$^+$]$^+$: calcd: 275.05578, found: 275.05554.

***tert*-Butyl 2-diazo-2-(4-fluorophenyl)acetate (S28).** Prepared analogously as a yellow solid (1.36 g, 50 % yield).

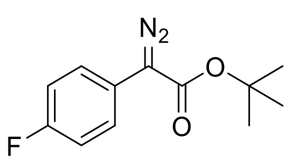

^{1}H NMR (400 MHz, $CDCl_3$): δ = 7.46 – 7.39 (m, 2H), 7.12 – 7.04 (m, 2H), 1.54 (s, 9H); ^{13}C NMR (101 MHz, $CDCl_3$): δ = 162.3 (d, *J* = 494.9 Hz), 162.3, 126.0 (d, *J* = 7.8 Hz), 122.0 (d, *J* = 3.1 Hz), 116.0 (d, *J* = 21.9 Hz), 82.3, 28.5 (C-N_2 was not observed); ^{19}F NMR (282 MHz, $CDCl_3$): δ = –116.8; IR (ATR): ṽ = 2980 (w), 2079 (s), 1693 (s), 1512 (s), 1366 (m), 1344 (m), 1232 (s), 1139 (s), 1011 (s), 828 (s), 610 (m), 508 (m) cm^{-1}. HRMS (ESI$^+$) for $C_{12}H_{13}N_2O_2FNa$ [M + Na$^+$]$^+$: calcd: 259.08533, found: 259.08522. The spectral data is consistent with previously reported values.[19]

***tert*-Butyl 2-diazo-2-(3,5-difluorophenyl)acetate (S29).** Prepared analogously as an orange oil (664 mg, 93 % yield).

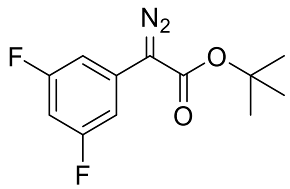

^{1}H NMR (400 MHz, $CDCl_3$): δ = 7.08 – 7.01 (m, 2H), 6.58 (tt, *J* = 8.8, 2.3 Hz, 1H), 1.55 (s, 9H); ^{13}C NMR (101 MHz, $CDCl_3$): δ = 163.5, 163.5 (dd, *J* = 247.2, 13.7 Hz), 130.4 (t, *J* = 11.4 Hz), 106.7 – 106.1 (m), 100.7 (t, *J* = 25.6 Hz), 83.0, 28.5 (C-N_2 was not observed); ^{19}F NMR (282 MHz, $CDCl_3$): δ = –109.0; IR (ATR): ṽ = 2980 (w), 2082 (s), 1698 (s), 1623 (s), 1588 (s), 1479 (m), 1450 (m), 1370 (m), 1256 (s), 1137 (s), 1068 (m), 988 (m), 837 (s), 669 (m) cm^{-1}; HRMS (ESI$^+$) for $C_{12}H_{12}F_2N_2O_2Na$ [M + Na$^+$]$^+$: calcd: 277.07590, found: 277.07577.

***tert*-Butyl 2-diazo-2-(4-(trifluoromethyl)phenyl)acetate (S30).** Prepared analogously as a yellow solid (1.07 g, 91 % yield).

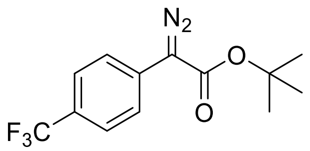

^{1}H NMR (400 MHz, $CDCl_3$): δ = 7.59 (s, 4H), 1.56 (s, 9H); ^{13}C NMR (151 MHz, $CDCl_3$): δ = 163.9, 130.9 (q, *J* = 1.5 Hz), 127.3 (q, *J* = 32.7 Hz), 125.8 (q, *J* = 3.8 Hz), 124.2 (q, *J* = 271.7 Hz), 123.5, 82.8, 28.5 (C-N_2 was not observed); ^{19}F NMR (282 MHz,$CDCl_3$): δ = –62.4. IR (ATR): ṽ = 3001 (w), 2096 (s), 1697 (s), 1320 (s), 1245 (s), 1115 (s), 1068 (s), 1004 (s), 843 (s), 821 (s), 749 (m), 521 (m) cm^{-1}; HRMS (ESI$^+$) for $C_{13}H_{13}F_3N_2O_2Na$ [M + Na$^+$]$^+$: calcd: 309.08213, found: 309.08194. The spectral data is consistent with previously reported values.[21]

---

[21] E. C. Lee, G. C. Fu, *J. Am. Chem. Soc.* **2007**, *129*, 12066.

Methyl 4-(2-(*tert*-butoxy)-1-diazo-2-oxoethyl)benzoate (S31). Prepared analogously as a yellow solid

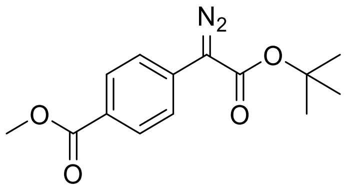

(508 mg, 85 % yield). ^{1}H NMR (400 MHz, $CDCl_3$): δ = 8.04 – 7.98 (m, 2H), 7.57 – 7.52 (m, 2H), 3.91 (s, 3H), 1.56 (s, 9H); ^{13}C NMR (101 MHz, $CDCl_3$): δ = 166.9, 163.8, 131.9, 130.2, 126.9, 123.1, 82.7, 52.2, 28.5 (C-N_2 not detected); IR (ATR): ṽ = 2975 (w), 2090 (s), 1716 (s), 1690 (s), 1319 (m), 1240 (m), 1109 (s), 1003 (s), 767 (s), 534 (m), 499 (m) cm^{-1}; HRMS (ESI^+) for $C_{14}H_{16}N_2O_4Na$ $[M + Na^+]^+$: calcd: 299.10023, found: 299.09988.

***tert*-Butyl 2-(4-(methylsulfonyl)phenyl)acetate (S32).** Prepared analogously as a yellow solid (314 mg,

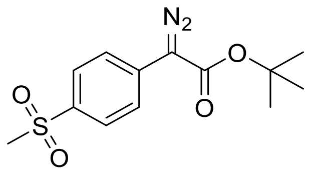

93 % yield). ^{1}H NMR (400 MHz, $CDCl_3$): δ = 7.93 – 7.87 (m, 2H), 7.71 – 7.65 (m, 2H), 3.04 (s, 3H), 1.56 (s, 9H); ^{13}C NMR (101 MHz, $CDCl_3$): δ = 163.4, 136.8, 133.5, 128.1, 123.7, 83.2, 44.8, 28.5 (C-N_2 not detected); IR (ATR): ṽ = 2978 (w), 2087 (s), 1693 (s), 1592 (m), 1289 (s), 1102 (s), 1003 (s), 770 (s), 524 (m) cm^{-1}; HRMS (ESI^+) for $C_{13}H_{16}N_2O_4SNa$ $[M + Na^+]^+$: calcd: 319.07230, found: 319.07187.

***tert*-Butyl 2-(2-chlorophenyl)-2-diazoacetate (S33).** Prepared analogously as a yellow solid (1.10 g, 82 %

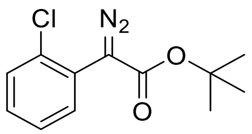

yield). ^{1}H NMR (400 MHz, $CDCl_3$) δ 7.57 – 7.53 (m, 1H), 7.43 – 7.39 (m, 1H), 7.34 – 7.28 (m, 1H), 7.27 – 7.21 (m, 1H), 1.53 (s, 9H). ^{13}C NMR (101 MHz, $CDCl_3$) δ 164.9, 133.7, 132.3, 130.2, 129.4, 127.1, 124.6, 82.3, 28.5 (C-N_2 was not observed). IR (ATR) ṽ 2978 (w), 2089 (s), 1693 (s), 1479 (m), 1375 (m), 1287 (m), 1245 (m), 1143 (s), 1071 (m), 1003 (m), 750 (s), 649 (m), 462 (w) cm^{-1}. HRMS (ESI^+) for $C_{12}H_{13}N_2O_2ClNa$ $[M + Na^+]^+$: calcd: 275.05577, found: 275.05542.

***tert*-Butyl 2-diazo-2-(4-methylphenyl)acetate (S34).** In a flame-dried Schlenk flask, *tert*-butyl 2-(p-

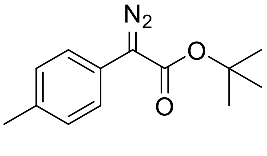

tolyl)acetate **S19** (1.33 g, 6.41 mmol) was dissolved in MeCN (20 mL) and the solution cooled to 0 °C. The flask was wrapped with aluminum foil before 2-nitrobenzenesulfonyl azide (2.22 g, 9.72 mmol) was added in portions. 1,8-Diazabicyclo[5.4.0]undec-7-ene (2.0 mL, 13.4 mmol) was added dropwise via syringe. After the addition was completed, the solution was stirred at room temperature overnight. Sat. NH_4Cl solution (40 mL) was added and the mixture was extracted with *tert*-butyl methyl ether (3 x, 40 mL). The combined organic layers were dried over $MgSO_4$ and concentrated to yield a red oil, which was purified by flash chromatography (silica, pentane/Et_2O = 98/2). The obtained orange product was dissolved in the minimum amount of pentane and the resulting solution cooled to –78 °C with dry ice to give orange crystals, which were collected by filtration (607 mg, 41 % yield). ^{1}H NMR (400 MHz, $CDCl_3$): δ = 7.37 – 7.33 (m, 2H), 7.20 – 7.16 (m, 2H), 2.33 (s, 3H), 1.55 (s, 9H); ^{13}C NMR (101 MHz, $CDCl_3$): δ = 165.0, 135.5, 129.7, 124.3, 122.9, 82.0, 28.6, 21.1 (C-N_2 was not observed); IR (ATR): ṽ = 2981 (w), 2076 (s), 1697 (s), 1516 (m), 1344 (m), 1243 (m), 1139 (s), 1009 (s), 808 (s), 499 (m) cm^{-1}; HRMS (ESI^+) for $C_{13}H_{16}N_2O_2Na$ $[M + Na^+]^+$: calcd: 255.11040, found: 255.11016. The spectral data is consistent with previously reported values.[19]

ENANTIOSELECTIVE FLUORINATION

Representative Procedure. Preparation of *tert*-Butyl (*S*)-2-fluoro-2-phenylacetate (2b).

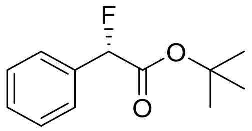

[Cu(OTf)]$_2$·toluene (1.25 mg, 0.0024 mmol) and ligand **L19** (5.00 mg, 0.0071 mmol) were dissolved in dry C_6F_6 (5 mL) in a flame dried Schlenk flask under Ar. The solution was stirred for 30 min before it was added to a solution of caesium fluoride (78.0 mg, 0.514 mmol) in dry hexafluoroisopropanol (0.160 mL) in a second flame-dried Schlenk flask. A solution of diazo compound **1b** (33.8 mg, 0.155 mmol) in dry C_6F_6 (1 mL) was added within 6 h via syringe pump. Once the addition was complete, the solution was stirred for additional 6 h before it was passed through a plug of silica, eluting with CH_2Cl_2. The filtrate was concentrated and the residue was purified by flash chromatography (silica, hexanes/EtOAc = 50/1) to yield the title compound as a colorless oil (28.2 mg, 87 % yield; 89 % *ee*). $[\alpha]_D^{20}$ = +65.9 (c = 1.12, $CHCl_3$); ^{1}H NMR (400 MHz, $CDCl_3$): δ = 7.49 – 7.36 (m, 5H), 5.65 (d, *J* = 48.2 Hz, 1H), 1.44 (s, 9H); ^{13}C NMR (101 MHz, $CDCl_3$): δ = 167.7 (d, *J* = 27.3 Hz), 134.9 (d, *J* = 20.5 Hz), 129.5 (d, *J* = 2.4 Hz), 128.8, 126.7 (d, *J* = 6.3 Hz), 89.5 (d, *J* = 185.3 Hz), 83.1, 28.0; ^{19}F NMR (282 MHz, $CDCl_3$): δ = –178.7; IR (ATR): ṽ = 2980 (w), 1753 (s), 1732 (s), 1369 (m), 1226 (m), 1156 (s), 1053 (s), 957 (m), 840 (m), 742 (s), 695 (s), 597 (m) cm^{-1}; HRMS (ESI$^+$) for $C_{12}H_{15}O_2FNa$ [M+Na$^+$]$^+$: calcd: 233.09483, found: 233.09490.

The *ee* of **2b** was determined by HPLC analysis: Daicel 150 mm Chiralpak IC-3, 4.6 mm i.D., n-heptane/i-propanol = 98/2, v = 1.0 mL/min, λ = 220 nm, t(major) = 4.07 min, t(minor) = 4.75 min.

All racemates, as necessary for *ee* determination, were prepared analogously using bis(4,4-dimethyl-4,5-dihydrooxazol-2-yl)methane as the ligand.[22]

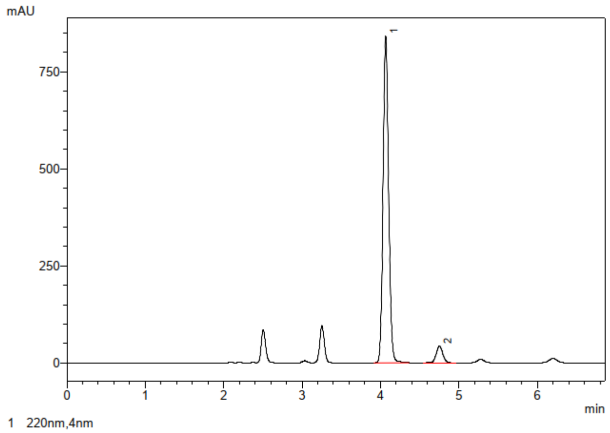

1 220nm,4nm

PDA Ch1 220nm

Peak #	Ret. Time	Area %	Name
1	4,07	94,27	1. Enantiomer, ee = 89 %
2	4,75	5,73	2. Enantiomer
Total		100,00	

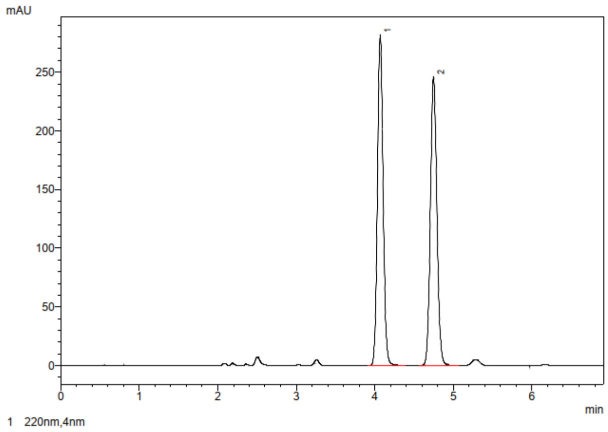

1 220nm,4nm

PDA Ch1 220nm

Peak #	Ret. Time	Area %	Name
1	4,07	49,96	1. Enantiomer
2	4,75	50,04	2. Enantiomer
Total		100,00	

Figure S8. HPLC traces of *tert*-butyl (*S*)-2-fluoro-2-phenylacetate (**2b**) (left) and the corresponding racemate (right).

22 A. Walli, S. Dechert, M. Bauer, S. Demeshko, F. Meyer, *Eur. J. Inorg. Chem.* **2014**, *2014*, 4660.

***tert*-Butyl (*S*)-2-(4-bromophenyl)-2-fluoroacetate (3).** Prepared analogously as a colorless oil (25.1 mg, 84 % yield; 93 % *ee*). [The *ee* was determined by HPLC analysis: Daicel 250 mm Chiralpak IA, 4.6 mm i.D., n-heptane/i-propanol = 98/2, v = 1.0 mL/min, λ = 220 nm, t(major) = 5.59 min, t(minor) = 5.03 min.] $[\alpha]_D^{20}$ = 45.1 (c = 0.46, $CHCl_3$); ^1H NMR (300 MHz, $CDCl_3$): δ = 7.53 (d, *J* = 8.6 Hz, 2H), 7.33 (d, *J* = 8.3 Hz, 2H), 5.60 (d, *J* = 48.0 Hz, 1H), 1.43 (s, 9H); ^{13}C NMR (75 MHz, $CDCl_3$): δ = 167.2 (d, *J* = 27.2 Hz), 134.0 (d, *J* = 20.8 Hz), 132.0, 128.2 (d, *J* = 6.2 Hz), 123.7 (d, *J* = 2.6 Hz), 88.9 (d, *J* = 186.3 Hz), 83.4, 28.0; ^{19}F NMR (282 MHz, $CDCl_3$): δ = –180.1. IR (ATR) ṽ 2983 (w), 1754 (s), 1492 (w), 1370 (m), 1150 (s), 1071 (s), 1015 (s), 963 (m), 830 (m), 782 (s), 493 (w) cm^{-1}; HRMS (ESI^+) for $C_{12}H_{14}BrFO_2Na$ $[M+Na^+]^+$: calcd: 311.00535, found: 311.00494.

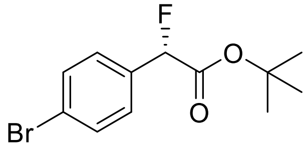

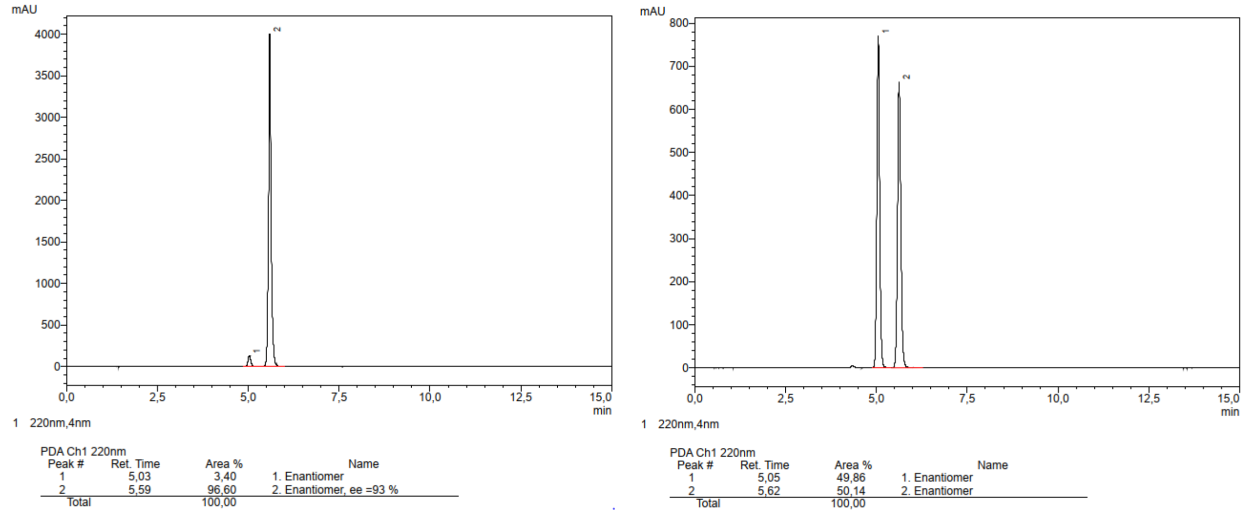

Figure S9. HPLC traces of *tert*-butyl (*S*)-2-(4-bromophenyl)-2-fluoroacetate (**3**) (left) and the corresponding racemate (right).

***tert*-Butyl (*S*)-2-(4-chlorophenyl)-2-fluoroacetate (4).** Prepared analogously as a colorless oil (25.0 mg, 74 % yield; 95 % *ee*). [The *ee* was determined by HPLC analysis: Daicel 250 mm Chiralpak IA, 4.6 mm i.D., n-heptane/i-propanol = 98/2, v = 1.0 mL/min, λ = 220 nm, t(major) = 5.66 min, t(minor) = 5.08 min.] $[\alpha]_D^{20}$ = 70.9 (c = 0.54, $CHCl_3$); ^1H NMR (400 MHz, $CDCl_3$): δ = 7.43 – 7.35 (m, 4H), 5.62 (d, *J* = 47.9 Hz, 1H), 1.44 (s, 9H); ^{13}C NMR (101 MHz, $CDCl_3$): δ = 167.3 (d, *J* = 27.5 Hz), 135.5 (d, *J* = 2.5 Hz), 133.4 (d, *J* = 21.0 Hz), 129.1, 128.0 (d, *J* = 6.4 Hz), 88.8 (d, *J* = 186.5 Hz), 83.4, 28.0; ^{19}F NMR (282 MHz, $CDCl_3$): δ = –179.7; IR (ATR): ṽ = 2985 (w), 1750 (s), 1491 (m), 1368 (m), 1154 (s), 1055 (m), 839 (m), 782 (m), 497 (w) cm^{-1}; HRMS (GC-CI, Isobutane) for $C_{12}H_{15}O_2FCl$ $[M+H^+]^+$: calcd: 245.07391, found: 245.07425.

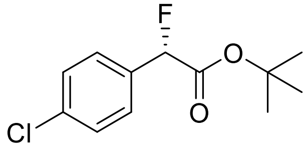

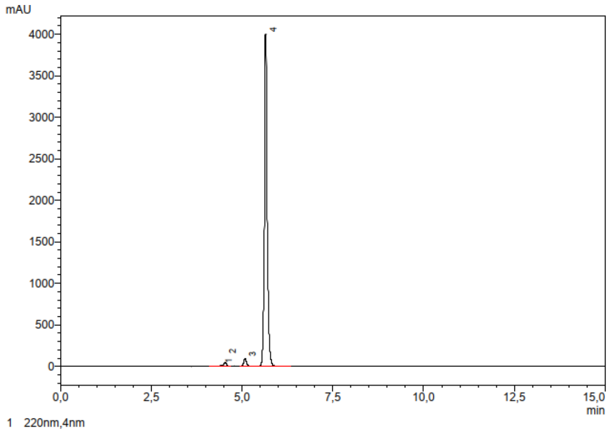

PDA Ch1 220nm

Peak #	Ret. Time	Area %	Name
1	4,43	0,27	
2	4,54	1,20	
3	5,08	2,37	1. Enantiomer
4	5,66	96,16	2. Enantiomer, ee=95 %
Total		100,00	

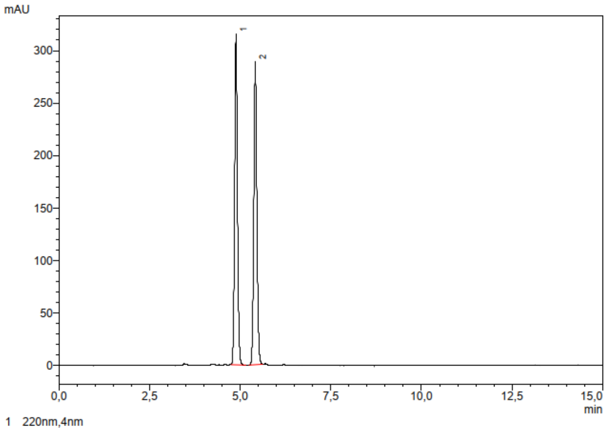

PDA Ch1 220nm

Peak #	Ret. Time	Area %	Name
1	4,89	49,89	1. Enantiomer
2	5,42	50,11	2. Enantiomer
Total		100,00	

Figure S10. HPLC traces of *tert*-butyl (*S*)-2-(4-chlorophenyl)-2-fluoroacetate (**4**) (left) and the corresponding racemate (right).

***tert*-Butyl (*S*)-2-(3-chlorophenyl)-2-fluoroacetate (5).** Prepared analogously as a colorless oil (28.5 mg, 96 % yield; 86 % *ee*). [The ee was determined by HPLC analysis: Daicel 150 mm Chiralpak IC-3, 3 µm, 4.6 mm i. D., n-heptane/i-propanol = 99/1, v = 1.0 mL/min, λ = 220 nm, t(major) = 4.18 min, t(minor) = 4.52 min.] $[\alpha]_D^{20}$ = + 49.3 (c = 0.46, $CHCl_3$); ^{1}H NMR (400 MHz, $CDCl_3$): δ = 7.47 – 7.44 (m, 1H), 7.39 – 7.29 (m, 3H), 5.61 (d, *J* = 48.0, 0.6 Hz, 1H), 1.45 (s, 9H); ^{13}C NMR (101 MHz, $CDCl_3$): δ = 167.1 (d, *J* = 27.1 Hz), 136.8 (d, *J* = 20.7 Hz), 134.8, 130.1, 129.6 (d, *J* = 1.6 Hz), 126.7 (d, *J* = 6.9 Hz), 124.6 (d, *J* = 6.4 Hz), 88.7 (d, *J* = 186.9 Hz), 83.6, 28.0; ^{19}F NMR (470 MHz, $CDCl_3$): δ = –181.0. IR (ATR): ṽ = 2981 (w), 1752 (s), 1477 (m), 1370 (m), 1166 (s), 1060 (s), 961 (m), 841 (m), 774 (s), 683 (m), 442 (w) cm^{-1}; HRMS (ESI$^+$) for $C_{12}H_{14}O_2FClNa$ $[M+Na^+]^+$: calcd: 267.05586, found: 267.05573.

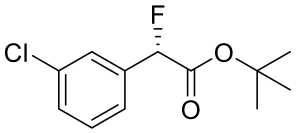

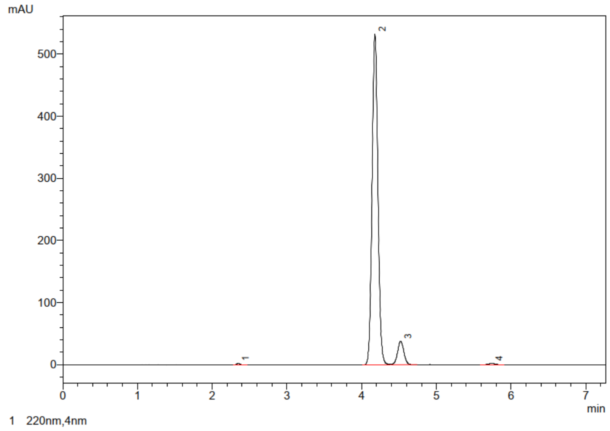

PDA Ch1 220nm

Peak #	Ret. Time	Area %	Name
1	2,35	0,26	
2	4,18	92,16	1. Enantiomer 85.7 % ee
3	4,52	7,08	2. Enantiomer
4	5,74	0,51	
Total		100,00	

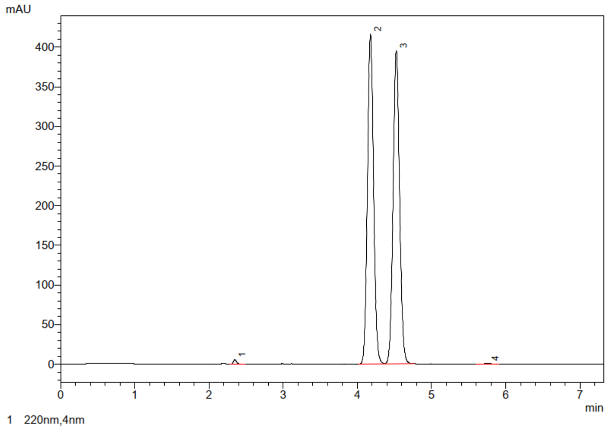

PDA Ch1 220nm

Peak #	Ret. Time	Area %	Name
1	2,35	0,35	
2	4,18	49,77	1. Enantiomer
3	4,53	49,77	2. Enantiomer
4	5,76	0,11	
Total		100,00	

Figure S11. HPLC traces of *tert*-butyl (*S*)-2-(3-chlorophenyl)-2-fluoroacetate (**5**) (left) and the corresponding racemate (right).

***tert*-Butyl (*S*)-2-fluoro-2-(4-fluorophenyl)acetate (6).** Prepared analogously as a colorless oil (28.4 mg, 91 % yield; 93 % *ee*). [The *ee* was determined by HPLC analysis: Daicel 250 mm Chiralpak IA, 4.6 mm i.D., n-heptane/i-propanol = 98/2, v = 1.0 mL/min, λ = 220 nm, t(major) = 5.44 min, t(minor) = 4.90 min.] $[\alpha]_D^{20}$ = +39.6 (c = 0.37, $CHCl_3$); ^{1}H NMR (400 MHz, $CDCl_3$): δ = 7.48 – 7.41 (m, 2H), 7.13 – 7.05 (m, 2H), 5.62 (d, *J* = 48.0 Hz, 1H), 1.44 (s, 9H); ^{13}C NMR (101 MHz, $CDCl_3$): δ = 167.5 (d, *J* = 27.5 Hz), 163.4 (dd, *J* = 248.4, 2.4 Hz), 130.9 (dd, *J* = 21.0, 3.3 Hz), 128.7 (dd, *J* = 8.5, 6.0 Hz), 115.9 (d, *J* = 22.0 Hz), 88.9 (d, *J* = 185.6 Hz), 83.3, 28.0; ^{19}F NMR (282 MHz, $CDCl_3$): δ = –111.9 (d, *J* = 3.8 Hz), –177.3 (d, *J* = 3.8 Hz); IR (ATR): ṽ = 2988 (w), 1748 (s), 1512 (s), 1225 (s), 1148 (s), 1053 (m), 843 (s), 568 (m), 515 (m) cm^{-1}; HRMS (GC-CI, Isobutane) for $C_{12}H_{15}O_2F_2$ $[M+H^+]^+$: calcd: 229.10346, found: 229.10352.

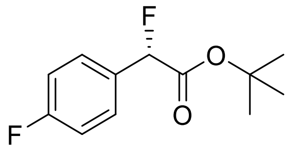

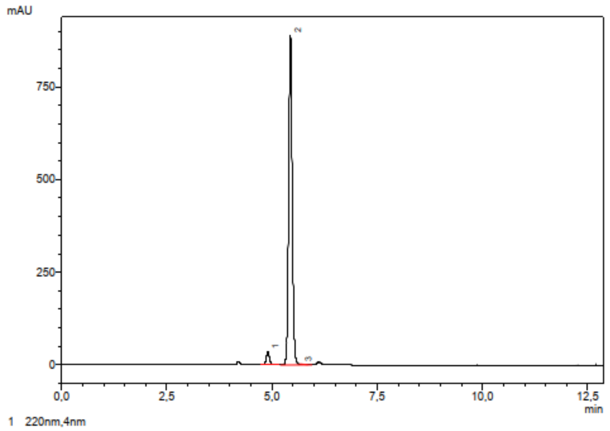

PDA Ch1 220nm

Peak #	Ret. Time	Area %	Name
1	4,90	3,61	1. Enantiomer
2	5,44	96,35	2. Enantiomer, ee = 93 %
3	5,68	0,04	
Total		100,00	

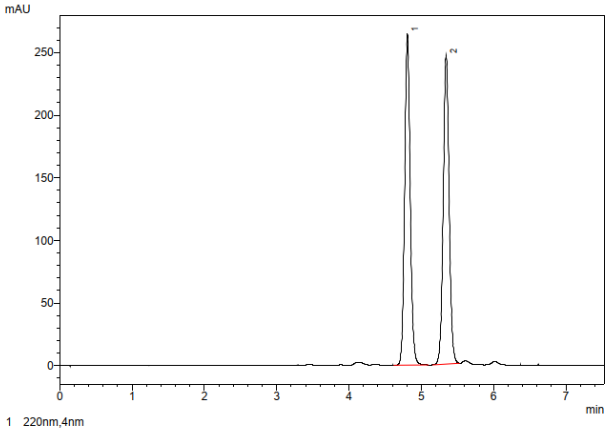

PDA Ch1 220nm

Peak #	Ret. Time	Area %	Name
1	4,81	50,19	1. Enantiomer
2	5,34	49,81	2. Enantiomer
Total		100,00	

Figure S12. HPLC traces of *tert*-butyl (*S*)-2-fluoro-2-(4-fluorophenyl)acetate (**6**) (left) and the corresponding racemate (right).

***tert*-Butyl (*S*)-2-(3,5-difluorophenyl)-2-fluoroacetate (7).** Prepared according to the representative procedure, but using CH_2Cl_2 instead of C_6F_6; colorless oil (23.0 mg, 68 % yield; 74 % *ee*). [The *ee* was determined by HPLC analysis: Daicel 150 mm Chiralcel OJ-3R, 4.6 mm i.D., MeCN/water = 40/60, v = 0.5 mL/min, λ = 220 nm, t(major) = 28.08 min, t(minor) = 29.93 min.] $[\alpha]_D^{20}$ = +39.0 (c = 0.25, $CHCl_3$); ^{1}H NMR (400 MHz, $CDCl_3$): δ = 7.00 (m, 2H), 6.83 (tt, *J* = 8.8, 2.3 Hz, 1H), 5.62 (d, *J* = 47.4 Hz, 1H), 1.46 (s, 9H); ^{13}C NMR (101 MHz, $CDCl_3$): δ = 164.4 (d, *J* = 12.5 Hz), 166.9 – 161.6 (m), 138.5 (m), 109.7 – 109.2 (m), 104.8 (td, *J* = 25.2, 1.5 Hz), 88.2 (dt, *J* = 188.8, 2.3 Hz), 83.9, 28.0; ^{19}F NMR (282 MHz, $CDCl_3$): δ = –108.4, –182.9. IR (ATR): ṽ = 2983 (w), 1754 (s), 1602 (s), 1461 (m), 1306 (m), 1159 (s), 1119 (s), 982 (s), 843 (s), 751 (m), 515 (m) cm^{-1}; HRMS (ESI^+) for $C_{12}H_{13}O_2F_3Na$ $[M+Na^+]^+$: calcd: 269.07598, found: 269.07550.

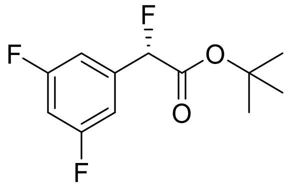

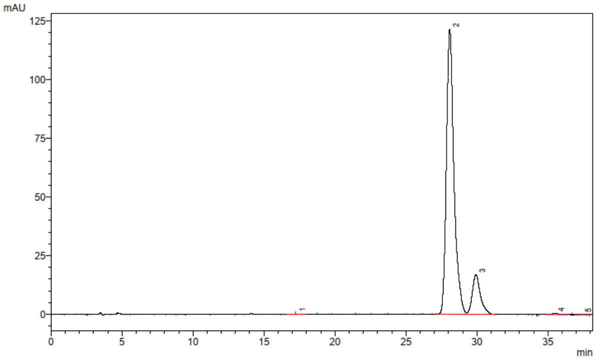

PDA Ch1 220nm

Peak #	Ret. Time	Area %	Name
1	17,26	0,14	
2	28,08	86,46	1. Enantiomer
3	29,93	12,91	2. Enantiomer 74.0 % ee
4	35,49	0,37	
5	37,42	0,13	
Total		100,00	

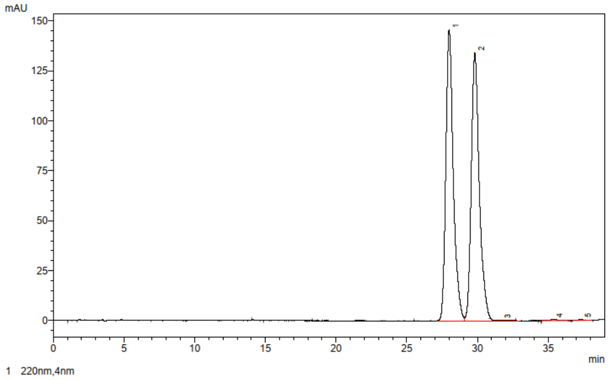

PDA Ch1 220nm

Peak #	Ret. Time	Area %	Name
1	27,99	49,92	1. Enantiomer
2	29,81	49,57	2. Enantiomer
3	31,67	0,13	
4	35,35	0,25	
5	37,36	0,12	
Total		100,00	

Figure S13. HPLC traces of *tert*-butyl (*S*)-2-(3,5-difluorophenyl)-2-fluoroacetate (**7**) (left) and the corresponding racemate (right).

***tert*-Butyl (*S*)-2-fluoro-2-(p-tolyl)acetate (8).** Prepared analogously as a colorless oil (28.6 mg, 87 % yield; 90 % *ee*). [The *ee* was determined by HPLC analysis: Daicel 150 mm Chiralpak IC-3, 4.6 mm i.D., n-heptane/i-propanol = 98/2, v = 1.0 mL/min, λ = 220 nm, t(major) = 4.83 min, t(minor) = 5.19 min.] $[\alpha]_D^{20}$ = +53.1 (c = 0.65, $CHCl_3$); ^1H NMR (400 MHz, $CDCl_3$): δ = 7.33 (d, *J* = 6.6 Hz, 2H), 7.20 (d, *J* = 8.6 Hz, 2H), 5.60 (d, *J* = 48.4 Hz, 1H), 2.37 (s, 3H), 1.44 (s, 9H); ^{13}C NMR (101 MHz, $CDCl_3$): δ = 167.9 (d, *J* = 27.9 Hz), 139.5 (d, *J* = 2.4 Hz), 132.0 (d, *J* = 20.5 Hz), 129.5, 126.8 (d, *J* = 5.7 Hz), 89.5 (d, *J* = 184.4 Hz), 82.9, 28.0, 21.4; ^{19}F NMR (282 MHz, $CDCl_3$): δ = –176.6. IR (ATR): ṽ = 2980 (w), 1752 (s), 1369 (m), 1153 (s), 1048 (m), 764 (m), 440 (m) cm^{-1}; HRMS (GC-CI, Isobutane) for $C_{13}H_{18}O_2F$ $[M+H^+]^+$: calcd: 225,12853, found: 225,12864.

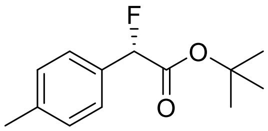

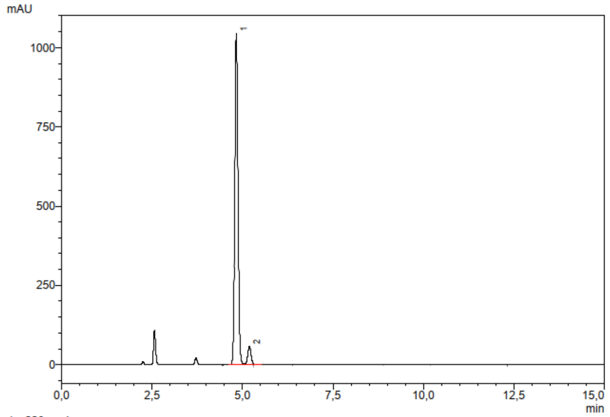

PDA Ch1 220nm

Peak #	Ret. Time	Area %	Name
1	4,83	94,45	1. Enantiomer, ee = 90 %
2	5,19	5,55	2. Enantiomer
Total		100,00	

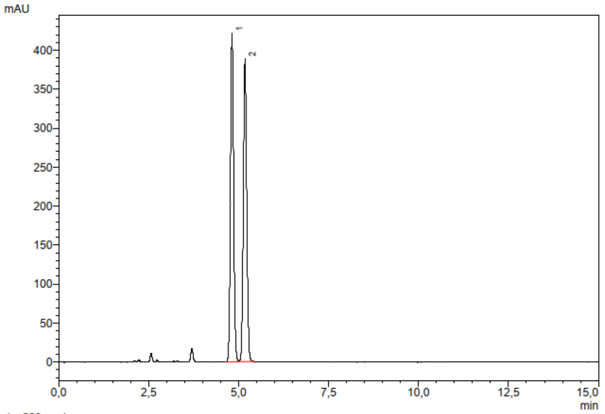

PDA Ch1 220nm

Peak #	Ret. Time	Area %	Name
1	4,82	50,09	1. Enantiomer
2	5,18	49,91	2. Enantiomer
Total		100,00	

Figure S14. HPLC traces of *tert*-butyl (*S*)-2-fluoro-2-(p-tolyl)acetate (**8**) (left) and the corresponding racemate (right).

***tert*-Butyl (*S*)-2-fluoro-2-(4-(trifluoromethyl)phenyl)acetate (9).** Prepared analogously as a colorless oil (16.9 mg, 52 % yield; 95 % *ee*). [The *ee* was determined by HPLC analysis: Daicel 250 mm Chiralpak IA, 4.6 mm i.D., n-heptane/i-propanol = 98/2, v = 1.0 mL/min, λ = 220 nm, t(major) = 4.97 min, t(minor) = 4.54 min.] $[\alpha]_D^{20}$ = +39.6 (c = 0.500, $CHCl_3$); ^{1}H NMR (400 MHz, $CDCl_3$): δ = 7.67 (d, *J* = 8.8 Hz, 2H), 7.59 (d, *J* = 8.1 Hz, 2H), 5.71 (d, *J* = 47.9 Hz, 1H), 1.44 (s, 9H); ^{13}C NMR (101 MHz, $CDCl_3$): δ = 166.9 (d, *J* = 26.6 Hz), 138.7 (d, *J* = 20.5 Hz), 131.4 (qd, *J* = 32.8, 1.8 Hz), 126.7 (d, *J* = 6.8 Hz), 125.8 (q, *J* = 3.8 Hz), 124.2 (q, *J* = 272.3 Hz), 88.8 (d, *J* = 187.2 Hz), 83.8, 28.0; ^{19}F NMR (282 MHz, $CDCl_3$): δ = –62.8 (d, *J* = 1.0 Hz), –182.9 (d, *J* = 1.6 Hz); IR (ATR): ṽ = 2985 (w), 1754 (s), 1322 (s), 1157 (s), 1126 (s), 1066 (s), 1020 (m), 837 (m), 795 (m), 598 (w) cm^{-1}; HRMS (ESI$^+$) for $C_{13}H_{14}F_4O_2Na$ [M+Na$^+$]$^+$: calcd: 301.08221, found: 301.08193.

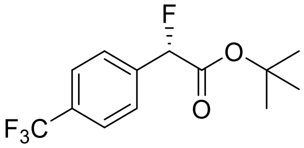

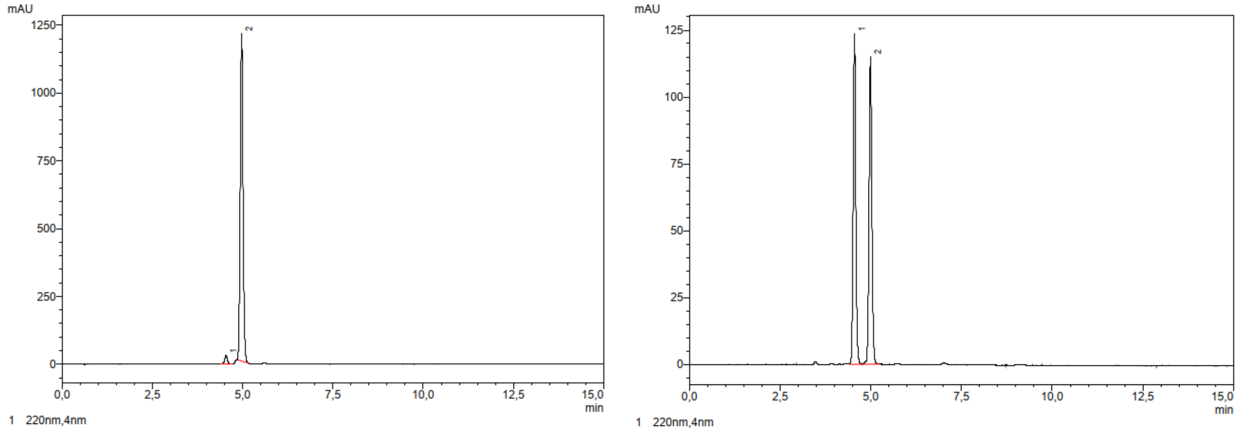

PDA Ch1 220nm

Peak #	Ret. Time	Area %	Name
1	4,54	2,39	1. Enantiomer
2	4,97	97,61	2. Enantiomer, ee =95 %
Total		100,00	

PDA Ch1 220nm

Peak #	Ret. Time	Area %	Name
1	4,55	49,91	1. Enantiomer
2	4,99	50,09	2. Enantiomer
Total		100,00	

Figure S15. HPLC traces of *tert*-butyl (*S*)-2-fluoro-2-(4-(trifluoromethyl)phenyl)acetate (**9**) (left) and the corresponding racemate (right).

Methyl (*S*)-4-(2-(*tert*-butoxy)-1-fluoro-2-oxoethyl)benzoate (10). Prepared analogously as a colorless oil (29.2 mg, 94 % yield; 75 % *ee*). [The *ee* was determined by HPLC analysis: Daicel 150 mm Chiralpak IA, 4.6 mm i.D., n-heptane/i-propanol = 90/10, v = 1.0 mL/min, λ = 220 nm, t(major) = 5.52 min, t(minor) = 5.06 min.] $[\alpha]_D^{20}$ = +55.0 (c = 0.55, $CHCl_3$). ^{1}H NMR (400 MHz, $CDCl_3$): δ = 8.07 (d, *J* = 7.8 Hz, 2H), 7.53 (d, *J* = 8.8 Hz, 2H), 5.70 (d, *J* = 48.0 Hz, 1H), 3.92 (s, 3H), 1.42 (s, 9H); ^{13}C NMR (101 MHz, $CDCl_3$): δ = 167.0 (d, *J* = 26.9 Hz), 166.7, 139.6 (d, *J* = 20.3 Hz), 131.1 (d, *J* = 2.0 Hz), 130.0, 126.3 (d, *J* = 6.8 Hz), 89.0 (d, *J* = 187.0 Hz), 83.6, 52.4, 28.0; ^{19}F NMR (282 MHz, $CDCl_3$): δ = –182.8; IR (ATR): ṽ = 2989 (w), 1749 (s), 1718 (s), 1367 (m), 1273 (s), 1228 (s), 1152 (s), 1109 (s), 1045 (s), 956 (m), 823 (s), 758 (s) cm^{-1}; HRMS (GC-CI, Isobutane) for $C_{14}H_{18}O_4F$ [M + H$^+$]$^+$: calcd: 269.11836, found: 269.11826.

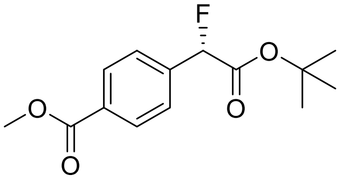

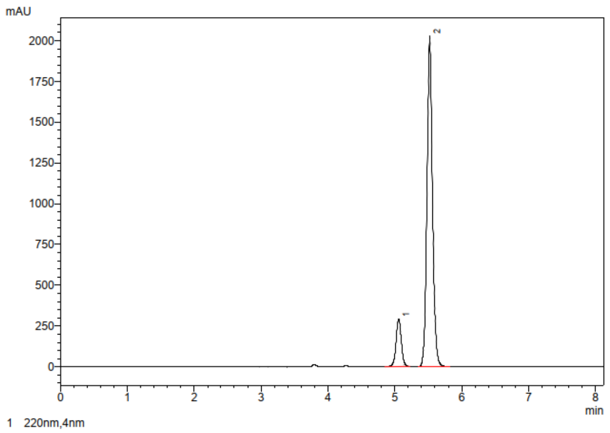

PDA Ch1 220nm

Peak #	Ret. Time	Area %	Name
1	5,06	12,31	1. Enantiomer
2	5,52	87,69	2. Enantiomer, ee =75 %
Total		100,00	

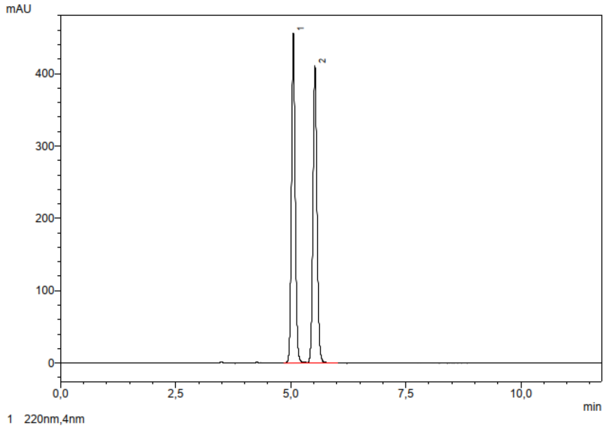

PDA Ch1 220nm

Peak #	Ret. Time	Area %	Name
1	5,05	50,07	1. Enantiomer
2	5,52	49,93	2. Enantiomer
Total		100,00	

Figure S16. HPLC traces of fluoride **10** (left) and its racemate (right).

***tert*-Butyl (*S*)-2-fluoro-2-(4-(methylsulfonyl)phenyl)acetate (11).** Prepared analogously as a colorless oil (19.7 mg, 65 % yield; 86 % *ee*). [The *ee* was determined by HPLC analysis: Daicel 250 mm Chiralpak IA, 4.6 mm i.D., n-heptane/i-propanol = 70/30, v = 1.0 mL/min, λ = 220 nm, t(major) = 6.61 min, t(minor) = 5.78 min.] $[\alpha]_D^{20}$ = +36.3 (c = 0.54, $CHCl_3$); ^{1}H NMR (400 MHz, $CDCl_3$): δ = 7.93 (d, *J* = 7.8 Hz, 2H), 7.61 (d, *J* = 8.8 Hz, 2H), 5.68 (d, *J* = 47.8 Hz, 1H), 3.01 (s, 3H), 1.38 (s, 9H); ^{13}C NMR (101 MHz, $CDCl_3$): δ = 166.5 (d, *J* = 26.2 Hz), 141.4 (d, *J* = 1.6 Hz), 140.8 (d, *J* = 20.6 Hz), 127.9, 127.2 (d, *J* = 7.0 Hz), 88.5 (d, *J* = 188.3 Hz), 84.1, 44.6, 28.0; ^{19}F NMR (282 MHz, $CDCl_3$): δ = –184.0. IR (ATR): ṽ = 2932 (w), 1737 (s), 1305 (s), 1151 (s), 1069 (s), 963 (m), 834 (m), 754 (s), 646 (m), 539 (s), 498 (m) cm^{-1}. HRMS (ESI$^+$) for $C_{13}H_{17}O_4SFNa$ [M + Na$^+$]$^+$: calcd: 311.07238, found: 311.07183.

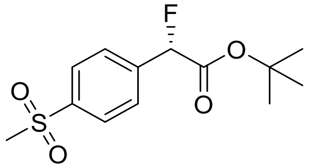

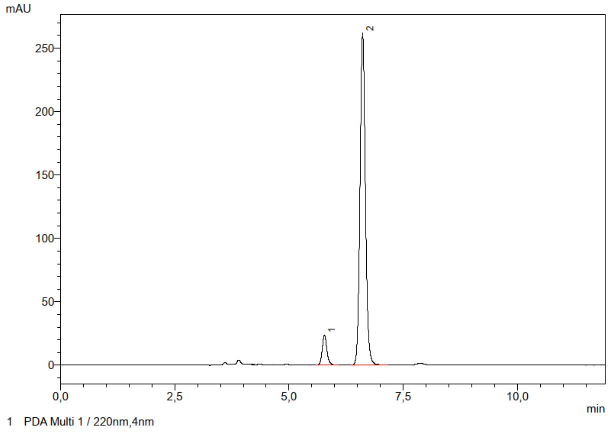

PDA Ch1 220nm

Peak #	Ret. Time	Name
1	5,78	1. Enantiomer
2	6,61	2. Enantiomer, ee=86 %
Total		

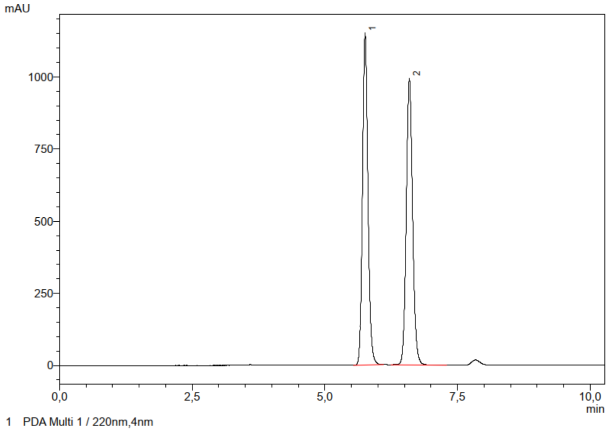

PDA Ch1 220nm

Peak #	Ret. Time	Name
1	5,76	1. Enantiomer
2	6,59	2. Enantiomer
Total		

Figure S17. HPLC traces of compound **11** (left) and the corresponding racemate (right).

SUPPORTING COMPUTATIONAL DATA

The structures were optimized with the ORCA 4.1.2 program package[23] employing the BP86 functional[24,25] together with the def2-SVP basis set[26] and D3 dispersion correction including Becke-Johnson damping (D3-BJ).[27] The resolution-of-identity (RI) approximation was utilized to speed up the calculations.[28,29] Inclusion of implicit solvent effects was achieved by employing the Conductor-like Polarizable Continuum Model (CPCM)[30,31] by using the ORCA default values for CH_2Cl_2. Stationary points were characterized by the analytical calculation of the Hessian. This level of theory is denoted as BP86-D3(BJ)-CPCM/def2-SVP. Transition states were optimized with the same methodology. They were identified by possessing one imaginary frequency corresponding to the respective reaction path and characterized via calculations of the intrinsic reaction coordinate (IRC) to connect the two minima in question. Thermodynamic corrections at ambient conditions (298.15 K, 1 atm) from the frequency calculations were used to obtain Gibbs free energies ΔG for the reaction mechanism. The BP86-D3(BJ)-CPCM/def2-SVP optimized structures were employed in single point calculations with the larger def2-TZVPP basis set[26] to obtain more accurate energies, denoted as BP86-D3(BJ)-CPCM/def2-TZVPP//BP86-D3(BJ)-CPCM/def2-SVP.

In order to estimate the rotational barriers, constrained optimizations at BP86-D3(BJ)-CPCM/def2-TZVPP level of theory were carried out in which the respective dihedral angles were changed in small steps (2° for the ester rotation, 4° for the rotation around the copper carbene bond) and kept fixed while the rest of the structure was allowed to relax.

Calculation of the electrostatic potential (ESP) of structure **2** was carried out by generation of a wave function file at BP86-D3(BJ)-CPCM/def2-SVP level using the program MultiWFN 3.6.[32]

---

23 a) F. Neese, *Wiley Interdiscip. Rev. Comput. Mol. Sci.* **2012**, *2*, 73–78; b) F. Neese, *Wiley Interdiscip. Rev. Comput. Mol. Sci.* **2017**, *8*, e1327.

24 A. D. Becke, *Phys. Rev. A* **1988**, *38*, 3098–3100.

25 J. P. Perdew, *Phys. Rev. B* **1986**, *33*, 8822–8824.

26 a) F. Weigend, R. Ahlrichs, *Phys. Chem. Chem. Phys.* **2005**, *7*, 3297–3305; b) F. Weigend, *Phys. Chem. Chem. Phys.* **2006**, *8*, 1057.

27 a) S. Grimme, J. Antony, S. Ehrlich, H. Krieg, *J. Chem. Phys.* **2010**, *132*, 154104–154104; b) S. Grimme, S. Ehrlich, L. Goerigk, *J. Comp. Chem.* **2011**, *32*, 1456–1465.

28 O. Vahtras, J. Almlöf, M. W. Feyereisen, *Chem. Phys. Lett.* **1993**, *213*, 514–518.

29 a) K. Eichkorn, O. Treutler, H. Öhm, M. Häser, R. Ahlrichs, *Chem. Phys. Lett.* **1995**, *240*, 283–290; b) K. Eichkorn, O. Treutler, H. Öhm, M. Häser, R. Ahlrichs, *Chem. Phys. Lett.* **1995**, *242*, 652–660; c) R. Ahlrichs, *Phys. Chem. Chem. Phys.* **2004**, *6*, 5119.

30 a) A. Klamt, G. Schüürmann, *J. Chem. Soc. Perkin Trans. 2* **1993**, 799–805; b) J. Andzelm, C. Kölmel, A. Klamt, *J. Chem. Phys.* **1995**, *103*, 9312–9320.

31 a) M. Cossi, N. Rega, G. Scalmani, V. Barone, *J. Comput. Chem.* **2003**, *24*, 669–681; b) V. Barone, M. Cossi, *J. Phys. Chem. A* **1998**, *102*, 1995–2001; c) M. Cossi, N. Rega, G. Scalmani, V. Barone, *J. Comput. Chem.* **2003**, *24*, 669–681.

32 T. Lu, F. Chen, *J. Comput. Chem.* **2012**, *33*, 580–592.

Figure S18. Optimized structures of minima and transition states calculated at BP86-D3(BJ)-CPCM/def2-SVP level of theory; bond lengths in Å, angles in °. Experimental values are shown in parentheses. Hydrogen atoms omitted for clarity.

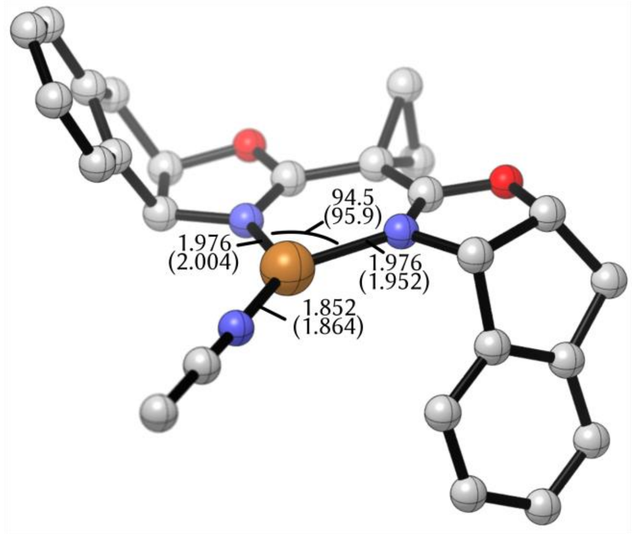

[**L3**·Cu(MeCN)]BF_4

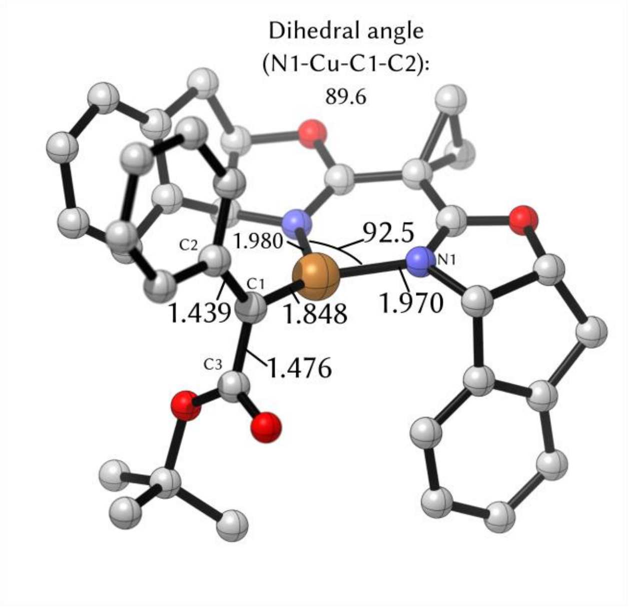

[**L3**·Cu=CPh(COO*t*Bu)]BF_4

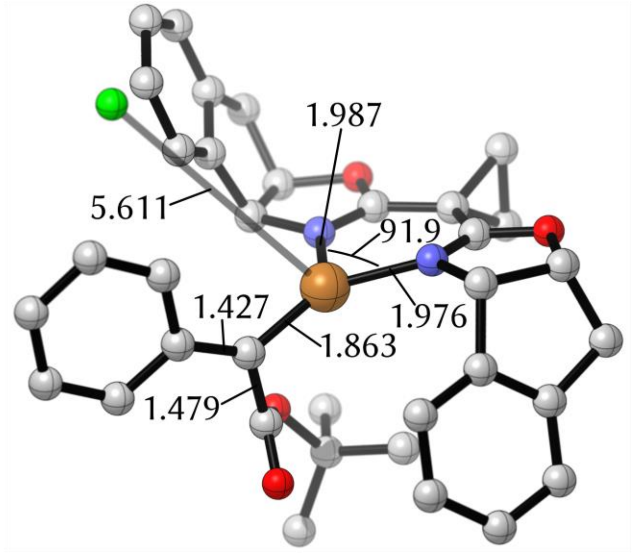

$TS^{F\rightarrow Cu}_{top}$

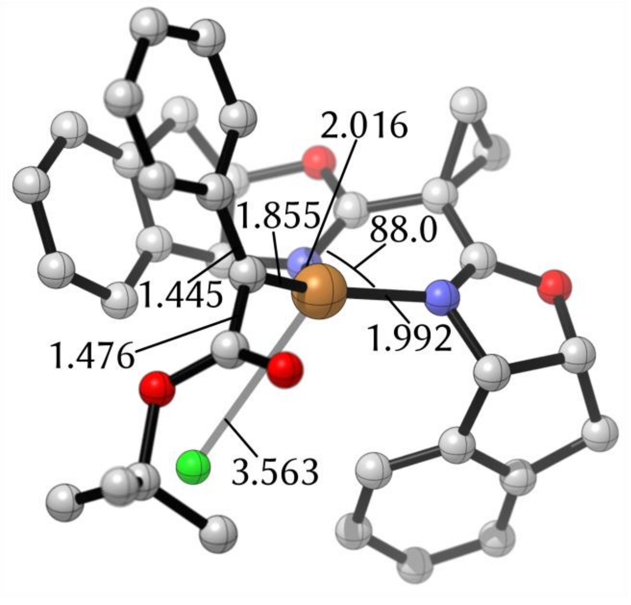

$TS^{F\rightarrow Cu}_{bottom}$

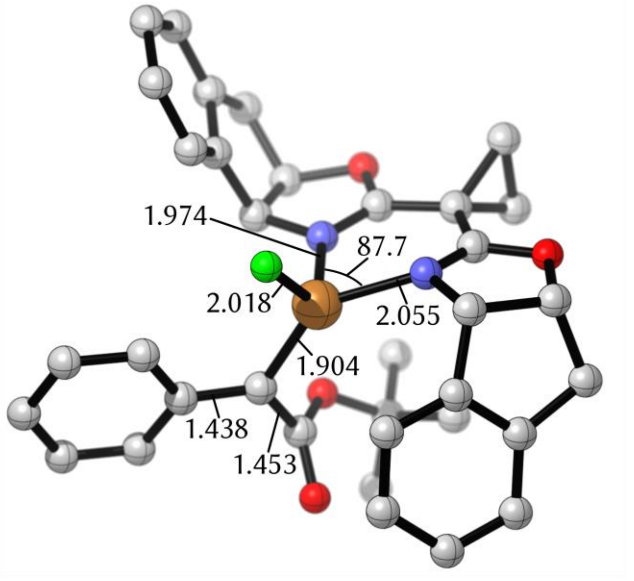

F@Cu$_{top}$

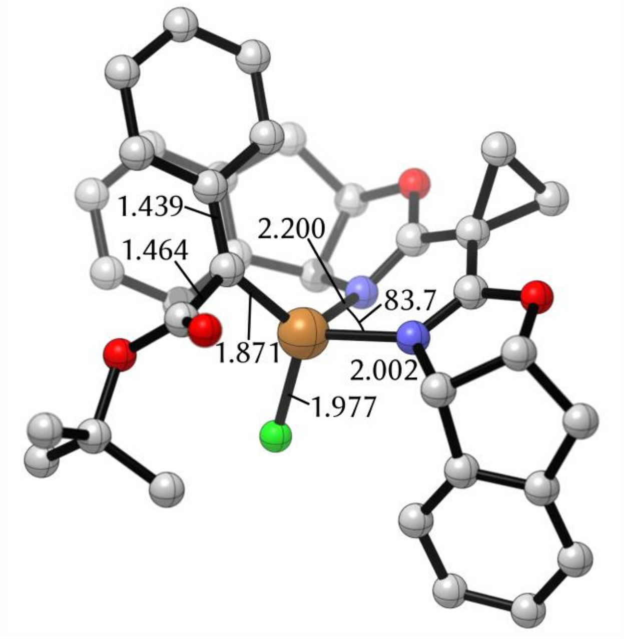

F@Cu$_{bottom}$

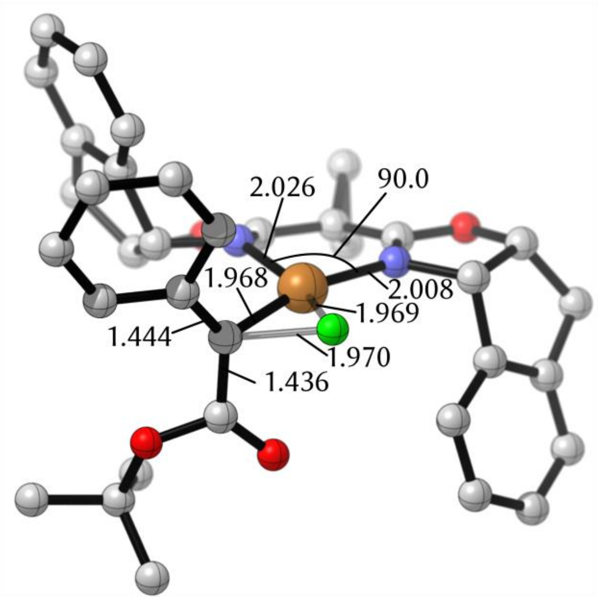

TS-F@Cu$_{top}$/(*S*)-**2b**@Cu

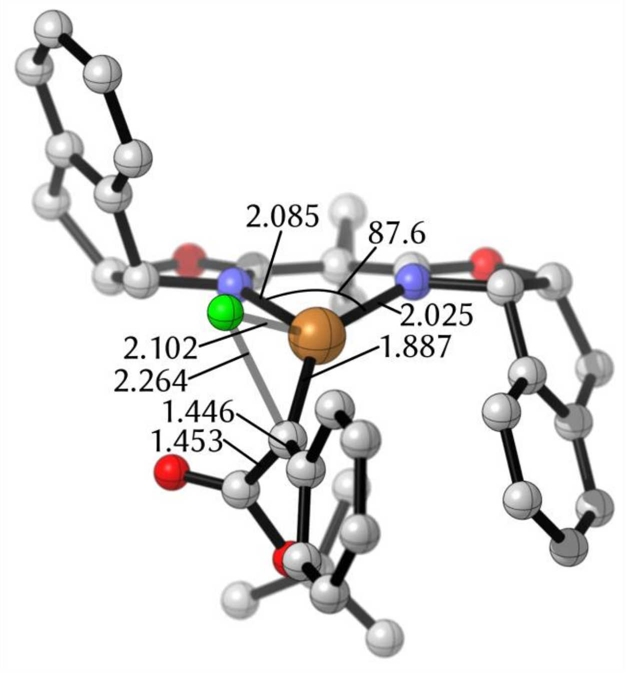

TS-F@Cu$_{top}$/(*R*)-**2b**@Cu

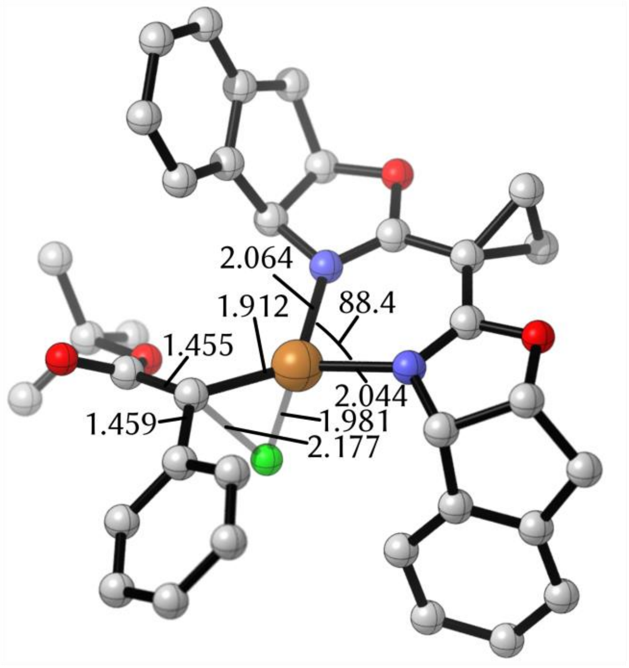

TS-F@Cu$_{\mathbf{bottom}}$/(*S*)-**2b**@Cu

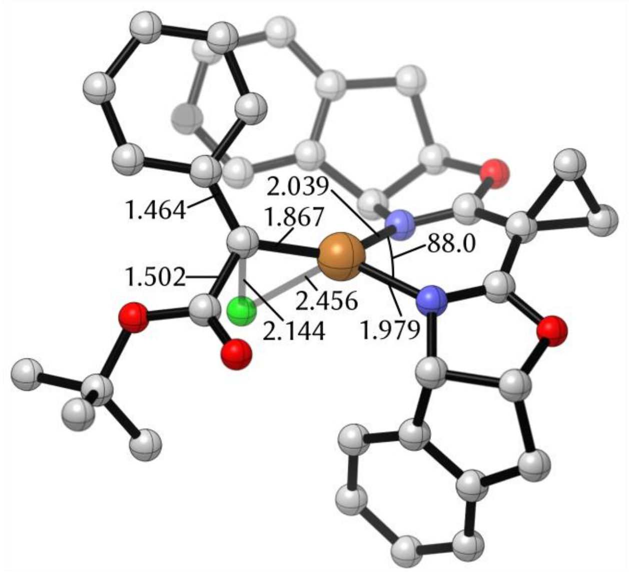

TS-F@Cu$_{\mathbf{bottom}}$/(*R*)-**2b**@Cu

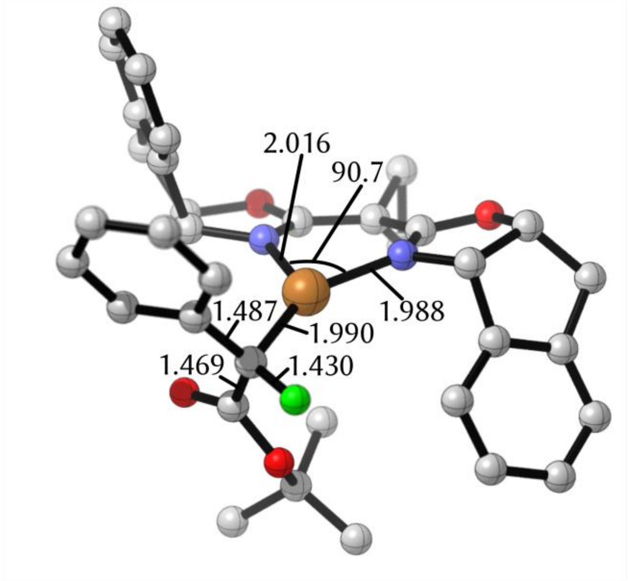

(*S*)-**2b**@Cu

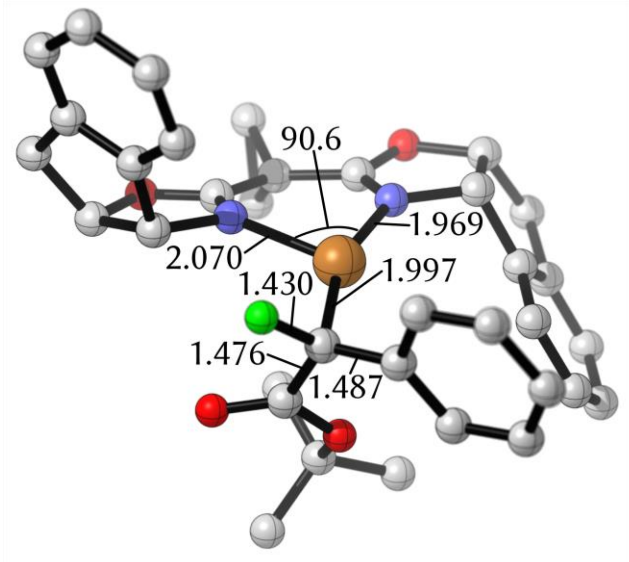

(*R*)-**2b**@Cu

Figure S19. Rotational barrier for the copper-carbene (Cu–C1) bond in [**L3**·Cu=CPh(COO*t*Bu)]BF_4. Shown is the clockwise and counter-clockwise displacement from equilibrium of the carbene moiety against the copper complex in °. Energies are given in kcal/mol and were calculated at BP86-D3(BJ)-CPCM/def2-TZVPP level of theory by constrained optimization.

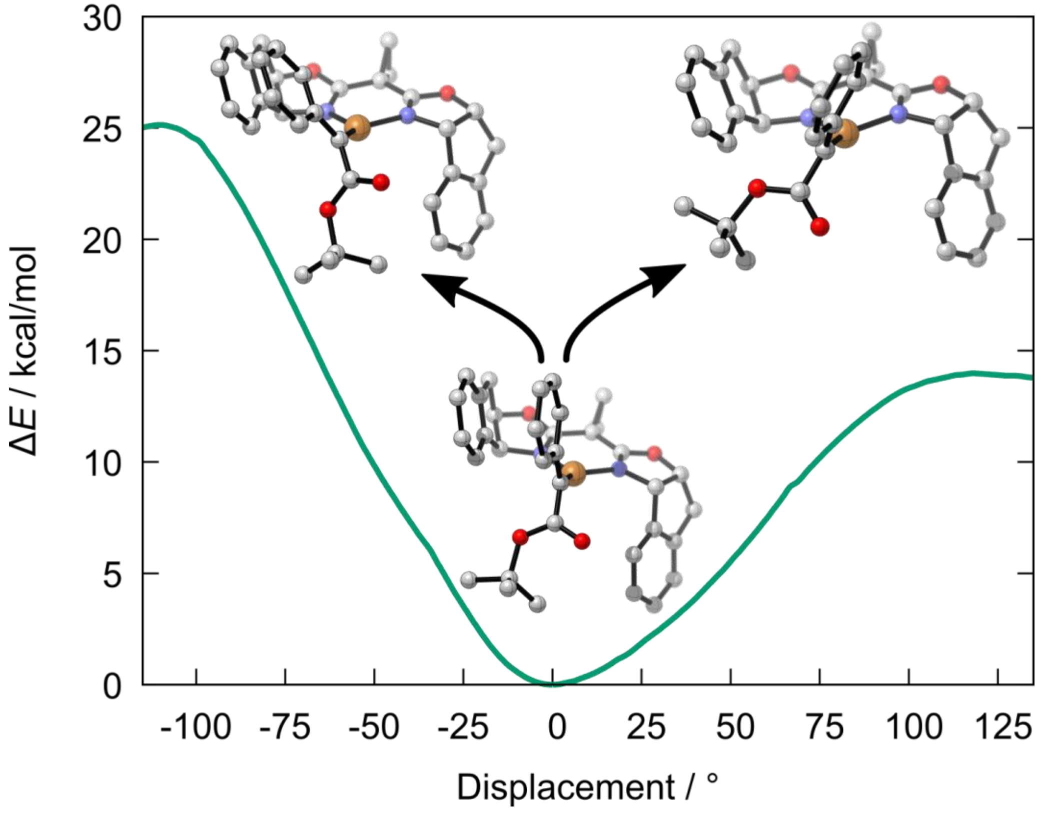

Figure S20. Barrier for the rotation of the ester group (C1–C3 bond) in [**L3**·Cu=C̲–(C̲OO*t*Bu)(Ph)]BF_4. Shown is the clockwise and counter-clockwise displacement from equilibrium in °. Energies are given in kcal/mol and were calculated at BP86-D3(BJ)-CPCM/def2-TZVPP level of theory by constrained optimization.

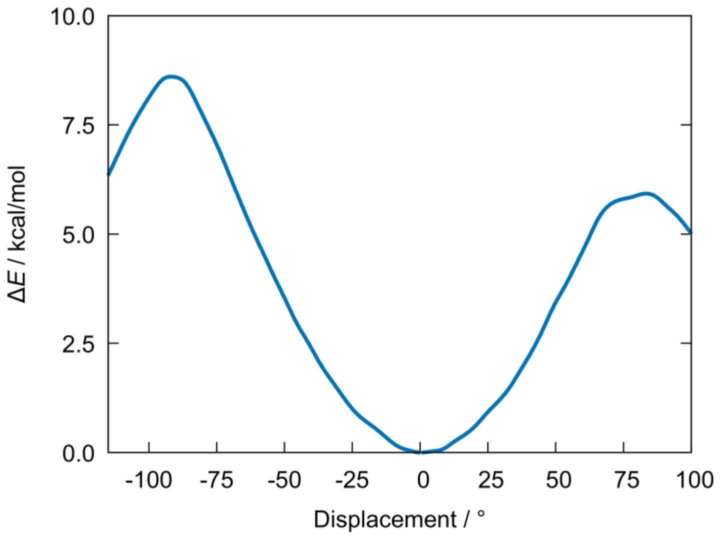

Figure S21. Electrostatic potential (ESP) of [**L3**·Cu=CPh(COO*t*Bu)]BF_4 mapped onto the electron density isovalue surface of 0.05 *e*/Bohr^3 at the BP86-D3(BJ)-CPCM/def2-SVP level of theory. Deep blue and light-green areas correspond to ESP values of +0.5 and –0.5 a.u., respectively.

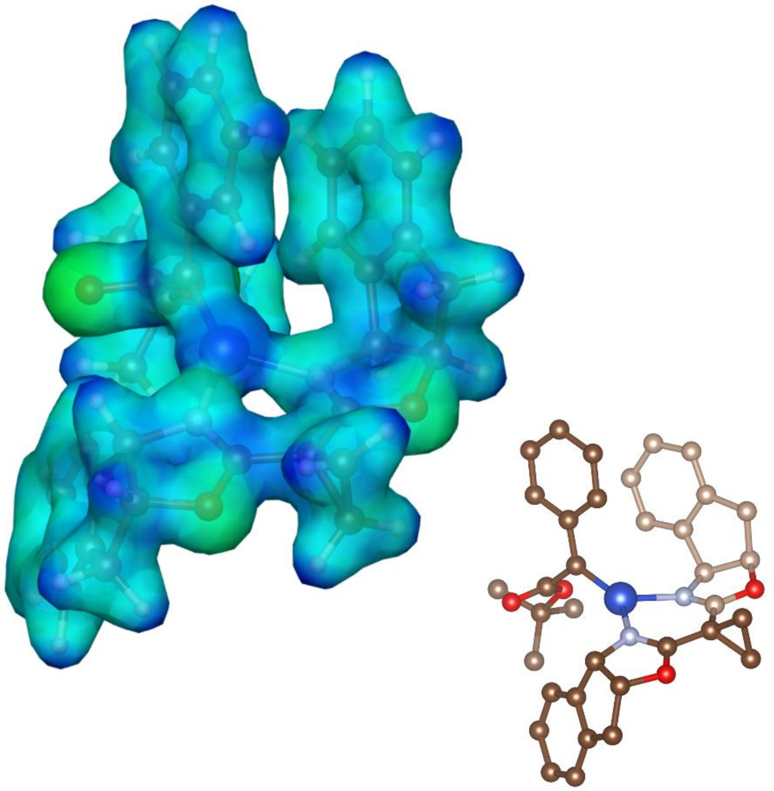

Figure S22. Reaction profile for nucleophilic attack of fluoride from the top face onto [**L3**·Cu=CPh(COO*t*Bu)]BF_4 and subsequent isomerization of the intermediate **F@Cu$_{top}$** initially formed into (*S*)-**2b**@Cu or (*R*)-**2b**@Cu as calculated at BP86-D3(BJ)-CPCM/def2-TZVPP//BP86-D3(BJ)-CPCM/def2-SVP level of theory. Shown are the relative Gibbs free energies in kcal/mol.

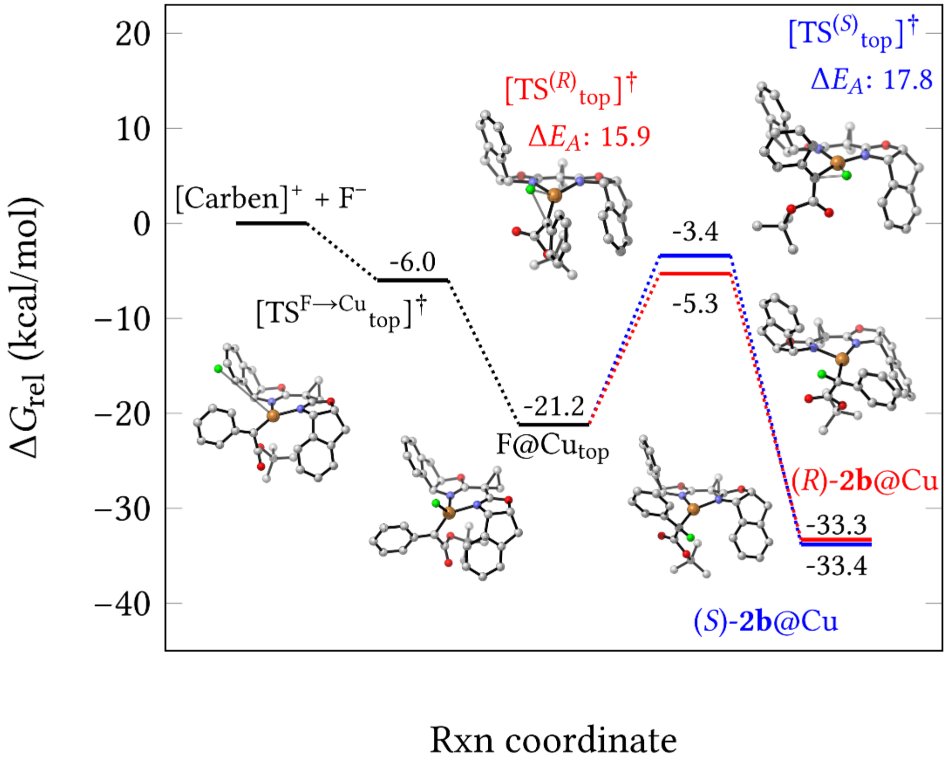

Figure S23. Reaction profile for nucleophilic attack of fluoride from the bottom face onto [**L3**·Cu=CPh(COO*t*Bu)]BF_4 and subsequent isomerization of the intermediate **F@Cu$_{bottom}$** initially formed into (*S*)-**2b**@Cu or (*R*)-**2b**@Cu as calculated at BP86-D3(BJ)-CPCM/def2-TZVPP//BP86-D3(BJ)-CPCM/def2-SVP level of theory. Shown are the relative Gibbs free energies in kcal/mol.

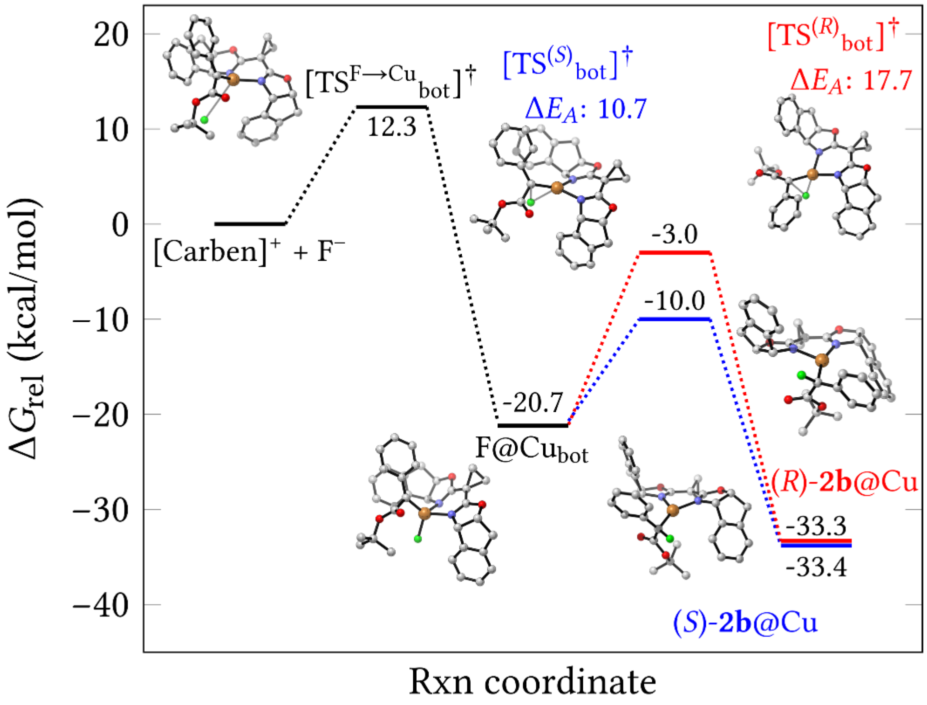

CARTESIAN COORDINATES

Cartesian coordinates for all optimized structures obtained at BP86-D3(BJ)-CPCM/def2-SVP level of theory. All values are in Å.

[**L3**·Cu(MeCN)]BF_4:

Cu	4.82535338615923	4.51066859009863	11.55154956184408
O	3.58002067549384	2.34236833214124	8.24708171813488
O	5.46456474060694	0.47084322253509	12.22218298982296
N	4.02392602958601	3.83738707768450	9.87613514076208
N	5.37964228131696	2.72409853736686	12.18696393903979
N	5.00172161062120	6.20392035301343	12.28187596368283
C	3.53771869231425	4.65796479339405	8.74238627463759
H	2.70988994480165	5.30904665839227	9.08795454897028
C	4.66835459670389	5.42879982084689	8.09019405933354
C	5.47525357330161	6.42121789035396	8.66618196701551
H	5.29507267944569	6.75701681998094	9.69800438937067
C	6.52045827986079	6.96980948855627	7.90185321383417
H	7.16600997500652	7.74880807159562	8.33545599437488
C	6.74547618028177	6.52682096822318	6.58291584559369
H	7.56738484219325	6.96327404398478	5.99466423540416
C	5.93002027723643	5.53378273626358	6.00884951695189
H	6.10977076169695	5.19378623236731	4.97720089774447
C	4.88754521670447	4.98198898912658	6.77153515129154
C	3.87302761826162	3.93320621346889	6.36648506612686
H	4.33177766217779	3.01989416264342	5.93624863562368
H	3.17128718196981	4.32828545081743	5.60194863209645
C	3.11330233055193	3.61001405530780	7.66493879650482
H	2.02157893496953	3.50461272296003	7.52791997953401
C	4.04884115956628	2.59902506318240	9.48293765403960
C	4.52486091352737	1.41604414178197	10.23756745409864
C	5.07863304077796	0.23546624246710	9.39767811353059
H	5.15144599831740	0.42882147845532	8.31801485148969
H	5.92578256499315	-0.29619290177618	9.85428486150033
C	3.73665778295918	0.09940192027250	10.01259165253489
H	3.62247872593045	-0.53087090001631	10.90598252347178
H	2.84881633427305	0.19860431005479	9.37168072893792
C	5.90569038273594	2.39298471262013	13.53154885969070
H	6.84241791805956	2.95769796327686	13.71093091867884
C	4.86024429068924	2.61685296243061	14.60665273584337
C	4.24099716205427	3.82347781275344	14.96480522533118
H	4.52797487617326	4.76538165090039	14.47447514642205
C	3.24229497045794	3.80230030174410	15.95468032747794
H	2.74330168274042	4.73810178255293	16.24944498814756
C	2.87685547412178	2.58851228586592	16.57077461761964
H	2.09302075362668	2.58456554010965	17.34385869265027
C	3.50375791740760	1.38145786827039	16.20958973675816
H	3.21466314303471	0.43682954034403	16.69569298740996

C	4.49964878263451	1.39969465906615	15.21923596244347
C	5.32637838098054	0.24739272773860	14.68821026177734
H	4.71796301754302	-0.62473223730362	14.37389146201046
H	6.03965213399341	-0.12067006255642	15.45566085900465
C	6.09626964356417	0.84355894323103	13.49758930038749
H	7.14752885312585	0.50801276260206	13.43262702502015
C	5.12987024717820	1.60429896054336	11.57676860591413
C	5.10021360641787	7.26906043618541	12.76369922505255
C	5.22717203025894	8.58454462402510	13.35697858291861
H	4.33933820063372	8.79617426124673	13.98638782861076
H	5.29654015655409	9.35535249189879	12.56322809630995
H	6.13620702541127	8.63003984988403	13.98990163364143

[**L3**·Cu=CPh(COOtBu)]BF_4:

Cu	-1.03535670564234	-2.55304708080998	-5.00893452001306
O	1.47266235568856	-1.34858195779618	-1.93376354732976
O	-2.74647298359389	-3.53792640157435	-1.40072486809887
N	0.40030395065046	-1.88273803053869	-3.83907471936457
N	-2.27066499609163	-2.82230082528056	-3.48485647334344
C	1.61091196061758	-1.16268546267932	-4.30020102164547
H	2.17074532132766	-1.80937671808940	-5.00534590852700
C	1.26932966936806	0.19477211175385	-4.87747129082161
C	0.54553567257986	0.46986698792660	-6.04697590805566
H	0.20805544378929	-0.34327113794155	-6.70767429191766
C	0.27958956324428	1.81259587237212	-6.37083654336936
H	-0.28381794384391	2.05381591469485	-7.28461385848430
C	0.73602716374004	2.85217854692572	-5.53590659576551
H	0.51954125134352	3.89838695805113	-5.80155189039728
C	1.47360715048713	2.56829710152913	-4.37160235815652
H	1.83722089696244	3.38544712754219	-3.72954771087913
C	1.73966853880041	1.22872411955055	-4.04342883628561
C	2.53984170465144	0.68462660997411	-2.87863305115432
H	2.18723740028977	1.05437947877906	-1.89421743968643
H	3.61019851920530	0.96721043798992	-2.96035619013416
C	2.38128917297789	-0.84447585751582	-2.97697781025215
H	3.32853279765841	-1.39904216287698	-2.85265259664203
C	0.40946823889839	-1.89709722792539	-2.54014271798679
C	-0.60930725248282	-2.49385295663233	-1.65067589362087
C	-0.68473254267816	-1.95998138695694	-0.19841279404604
H	-0.02932065211283	-1.10279945481098	0.01149860230461
H	-1.69883835190631	-1.91042546284744	0.22335623145518
C	-0.07317646116449	-3.28988206583400	-0.43180844525177
H	-0.64868622543820	-4.19199666308687	-0.17903575013728
H	1.02156493697519	-3.38488167500126	-0.38897899799228
C	-3.66625408291146	-3.34628852097172	-3.58002284651125
H	-4.34654789995675	-2.48184293359984	-3.72457244801095
C	-3.84843398302258	-4.42700259993748	-4.61538898472331

C	-4.05966896956111	-4.28102666663476	-5.99232930544848
H	-4.10825725251547	-3.28399263521720	-6.45156063237691
C	-4.17955342465186	-5.43864466767490	-6.78154522955661
H	-4.33366576281028	-5.34650281554359	-7.86684044191499
C	-4.08649269438741	-6.71290911970794	-6.19269971598969
H	-4.16811843310827	-7.61114670862093	-6.82340725321571
C	-3.89915795447947	-6.85422761742914	-4.80362078949486
H	-3.84749456746827	-7.85527011312083	-4.34853900743847
C	-3.78716900517710	-5.70114580130020	-4.01246131889836
C	-3.62683558827235	-5.59346654196920	-2.50957112233630
H	-2.61184576374680	-5.90623316853567	-2.18247740136709
H	-4.34995375248876	-6.22543016575577	-1.95708502095051
C	-3.83039071254488	-4.08431412021168	-2.22082526945975
H	-4.77264519127828	-3.86051248953062	-1.69164418824154
C	-1.88316314039768	-2.94851886697017	-2.24834967589872
C	-1.22198242465884	-3.12394366585436	-6.75699135244801
C	-1.05114063723781	-4.48683253435307	-7.18725748725136
C	-0.73658641236869	-5.48447405860053	-6.21521355609923
H	-0.59120755200646	-5.16962652149649	-5.16977056643516
C	-0.65538953582130	-6.83001663324195	-6.57084054190960
H	-0.43445067899799	-7.59143490947631	-5.80882702198902
C	-0.86455200756752	-7.21097818852990	-7.91207427349305
H	-0.80085583013756	-8.27296583656960	-8.19434093585705
C	-1.15924713270069	-6.24380669672029	-8.89479305780456
H	-1.32081383718577	-6.55217246368476	-9.93833386250691
C	-1.25611078025608	-4.89719391249167	-8.53949451500212
H	-1.49287267464262	-4.14400583975821	-9.30575357683884
C	-1.57325714914592	-2.11193333008279	-7.77194499948137
O	-0.68425718341872	-1.58614081759632	-8.44682580566947
O	-2.87582156115755	-1.81492934442739	-7.79619691470988
C	-3.44497306955516	-0.74559108628494	-8.67377186642965
C	-4.92568739663970	-0.75705935312013	-8.29285647561892
C	-3.24004071540046	-1.14643199529172	-10.13708663805338
C	-2.79089005403356	0.59399160085632	-8.32517437420482
H	-5.37399111184678	-1.75082443472783	-8.49060706202760
H	-5.47202837001452	-0.00157855550866	-8.89071708553583
H	-5.05534209864898	-0.51571079667465	-7.21923819397253
H	-2.85652992779612	0.78598336662050	-7.23556304235303
H	-3.32443732940402	1.40956925796006	-8.85241328766127
H	-1.72891932657165	0.61168395090181	-8.62942947453659
H	-3.67072928549591	-2.14987463942473	-10.32706787704308
H	-2.16680727973255	-1.15761877646228	-10.40269117985473
H	-3.75814980072141	-0.41886211490167	-10.79303730293534

$TS^{F\rightarrow Cu}{}_{top}$:

Cu	-0.96819547631531	-2.61854946615561	-5.35605295914508
O	1.21426457990167	-1.68979497744284	-1.95799905376759
O	-3.17779078499784	-3.57210490070482	-1.95425342601258

N	0.40657108976424	-2.24920375020916	-3.98550068991803
N	-2.25531136642031	-3.26044700958309	-3.98474418150591
C	1.75637851401427	-1.69700970392506	-4.27489769836338
H	2.36187618196723	-2.47913231868867	-4.77538269543963
C	1.67934893274604	-0.40586148293033	-5.05936600907849
C	1.29623217297315	-0.23558357848378	-6.39669444679081
H	1.05011310693185	-1.10448791561654	-7.02401043130866
C	1.21486750387913	1.06894665094606	-6.91410690733354
H	0.89558703986779	1.22034321942977	-7.95488967209217
C	1.51820721960459	2.17704431431558	-6.09978360284206
H	1.44397765861178	3.19415559405122	-6.51470952807717
C	1.92196364747301	1.99934957301839	-4.76292221028940
H	2.17170257248906	2.86936521133063	-4.13600989425205
C	2.00299186297991	0.69753062021938	-4.24383522351937
C	2.45204677246467	0.26153611041128	-2.86512366787422
H	1.84870409156232	0.71349589546207	-2.05083102615229
H	3.50736147776045	0.54608207087502	-2.67398180290941
C	2.28985696858574	-1.27366260596349	-2.86735957996123
H	3.19546864102311	-1.81510998663380	-2.54171438914208
C	0.20789139437882	-2.17635088750733	-2.70265144773387
C	-1.01088482456747	-2.55297315067508	-1.95215568308513
C	-1.38236731774993	-1.65460225331444	-0.73990867062357
H	-0.73848822254887	-0.77427157551524	-0.60074102475642
H	-2.46110128593929	-1.49367437197298	-0.59870062939029
C	-0.82025643154757	-3.00357236355677	-0.48664629885114
H	-1.49646480274469	-3.80566403497363	-0.15860643920622
H	0.22636101776021	-3.08625811755996	-0.16101368210092
C	-3.53142838111800	-3.95789789440066	-4.26831446624962
H	-4.09857329486332	-3.37468784386886	-5.02107607041259
C	-3.28023339538212	-5.39750662385034	-4.66091028702333
C	-2.55036373887464	-5.85946253707751	-5.76087169680706
H	-2.15074246456294	-5.14905080795759	-6.49439371524380
C	-2.29677412038988	-7.23418809006389	-5.91538036124553
H	-1.64899343622164	-7.54568098275807	-6.78160219559335
C	-2.81684961440718	-8.12600728578190	-4.95140971439570
H	-2.62820000577367	-9.20757533389656	-5.05342245831743
C	-3.56633573354661	-7.66733084254922	-3.84753593210936
H	-3.95892822887133	-8.38127085992865	-3.10586567683601
C	-3.78910495364037	-6.28771170889591	-3.69600954770032
C	-4.54014864007051	-5.54455966334270	-2.60899528901144
H	-4.24053480342478	-5.83587557554221	-1.58168552020069
H	-5.63550522404079	-5.71426418774029	-2.68168317589935
C	-4.22510729764177	-4.05981256130001	-2.87240438388091
H	-5.09203443122078	-3.38823916987967	-2.73236096643027

C	-2.14340178579660	-3.14230199681089	-2.69780624583552
C	-1.53208460130819	-2.26411845743968	-7.09541558565444
C	-1.67448120681898	-3.05885671947061	-8.27244043635782
C	-1.06596080089249	-4.35226509028919	-8.32162979386500
H	-0.40730973669663	-4.67934133037274	-7.50182961064940
C	-1.32659138535045	-5.25956339913723	-9.35222789871973
H	-0.91531227944526	-6.29894953875078	-9.18125213637868
C	-2.16337613366906	-4.84291474772248	-10.40807638267749
H	-2.38318731089850	-5.53468347142429	-11.23808011678169
C	-2.74002351631399	-3.54946576636392	-10.42608413578062
H	-3.38544744105464	-3.24716438960722	-11.26523110994041
C	-2.51122328798354	-2.66793948773669	-9.37091569564543
H	-2.97715824111972	-1.67128253985871	-9.36581334814898
C	-2.10417717020369	-0.90148436064661	-7.02894744730240
O	-1.66844257470186	0.03772786144572	-7.69224894146988
O	-3.04295311038023	-0.81653004406198	-6.05599457351600
C	-3.27349278440533	0.43575032411711	-5.28649620855586
C	-4.25339623725656	-0.01286906726751	-4.20075188984168
C	-3.88980499984721	1.50881152828156	-6.18839289342645
C	-1.93603064444655	0.86816658503801	-4.67450132629414
H	-5.18738597891315	-0.40266053970673	-4.65154614912373
H	-4.50885557951669	0.84079975104466	-3.54300061176758
H	-3.80176919494291	-0.81154784640733	-3.57967823198393
H	-1.49666893340891	0.03317169106954	-4.09176501046391
H	-2.09517687561958	1.72418345763398	-3.98975514900323
H	-1.20759061735147	1.16871454264123	-5.45048207471565
H	-4.81095382907219	1.12430822857512	-6.67030337186370
H	-3.17725611947423	1.81684240410687	-6.97447361043789
H	-4.16156370548167	2.39423677373554	-5.57900334004119
F	-0.45571694460618	-7.48950405874747	-8.09440049064510

$TS^{F\rightarrow Cu}{}_{bottom}$:

Cu	-0.14750687262159	-3.41477189507538	-5.12230262662088
O	1.53888626362745	-0.82066734814794	-2.37561621416369
O	-2.51877044217254	-3.14518806653539	-1.86740663344623
N	1.05769374612654	-2.23775881429561	-4.05978931120920
N	-1.66376203395694	-2.81794396526261	-3.93477886757437
C	1.88099050153177	-1.18478636765939	-4.68182643306060
H	2.65312804492653	-1.62454200505752	-5.34365788377402
C	0.95208765773273	-0.18944574253715	-5.36233176936586
C	-0.09721888047402	-0.46945681807810	-6.24926650311776
H	-0.37925516381201	-1.46960365319681	-6.63390770562206
C	-0.95203959721643	0.57888672396952	-6.63685797416205
H	-1.78774653230743	0.28540960030346	-7.29544162168729

C	-0.74383507123113	1.88370655732051	-6.15020117409728
H	-1.41617864258020	2.69996287890338	-6.45871220456119
C	0.29413574412819	2.15244322659498	-5.23349337133358
H	0.42509170608167	3.16548949904785	-4.82155054639675
C	1.13053363049302	1.10239546800959	-4.82299424296594
C	2.23025567221486	1.10485274883524	-3.77727317454723
H	1.97455790096942	1.67561756486521	-2.86179904394860
H	3.17917532194850	1.53288676638613	-4.16932647927052
C	2.44038360109784	-0.38435455012543	-3.46959265275081
H	3.46796647863409	-0.65019127845956	-3.15459950950973
C	0.83550966473325	-1.87006242378933	-2.83734825948006
C	-0.19641240419183	-2.47889467880484	-1.95721644636954
C	-0.26638186516295	-2.04703489029368	-0.49193488378782
H	0.44287386345818	-1.25907108833155	-0.20180124389326
H	-1.27741896483816	-1.96507237420755	-0.06936845060175
C	0.26271326767821	-3.40493943049786	-0.80581532850669
H	-0.37560283234772	-4.28197318295189	-0.62184481255065
H	1.34659954117193	-3.58567370304674	-0.74662412847606
C	-3.08859302169615	-3.15275126473836	-4.18164754214604
H	-3.58339667461610	-2.25559527912048	-4.60650972146765
C	-3.23120739485997	-4.37720640534375	-5.04762964335744
C	-2.98918541101312	-4.45462805606278	-6.42832864384120
H	-2.69637760418504	-3.53548275466157	-7.00174626268753
C	-3.10218036901936	-5.72527527320741	-7.02491665252929
H	-2.90247758064197	-5.83031405597447	-8.10054915510267
C	-3.42259579763358	-6.86308590695031	-6.26049924998182
H	-3.48806842611569	-7.84799124530121	-6.74840603799798
C	-3.64316414879773	-6.76478465954276	-4.87414058555359
H	-3.87592339355716	-7.66301899991914	-4.28117607834587
C	-3.54671009020730	-5.50283344627287	-4.26439517535046
C	-3.71929608749420	-5.13802069407023	-2.80173169914783
H	-2.92917971937186	-5.59403184000077	-2.16575977917852
H	-4.69106463618361	-5.47773806476946	-2.38787695584000
C	-3.59133328568957	-3.59234986327277	-2.77120378944234
H	-4.51054696833693	-3.08344382047774	-2.43121371380923
C	-1.47103413771256	-2.81438528692223	-2.65004759530534
C	0.01296096919047	-4.89143185049310	-6.23316494159130
C	-0.09272535890634	-6.26284223858789	-5.78937178821900
C	-0.35528389990806	-6.53376822967843	-4.41545574314557
H	-0.44217904619659	-5.67803076013517	-3.72839249782262
C	-0.49313924954974	-7.84443556062380	-3.95105413767333
H	-0.69467191762796	-8.03609517435748	-2.88632738657300
C	-0.37918949008545	-8.91875403514302	-4.85276871189905
H	-0.48937313533611	-9.95279695826285	-4.49061693387118

C	-0.12851570329547	-8.67847881492029	-6.21891565597555
H	-0.04611524827535	-9.52404316164292	-6.91845899401952
C	0.01218420611202	-7.36876819953879	-6.68368728834108
H	0.19659040805063	-7.17912688044031	-7.75134726480195
C	0.41637258358482	-4.62336790642946	-7.62711455027002
O	1.61410226960959	-4.42439077085359	-7.85022282614543
O	-0.58205024997595	-4.59213297077250	-8.49954498690112
C	-0.47561654157890	-3.89492353524022	-9.82181455996760
C	-1.94233105170975	-3.77193649938683	-10.23780122056363
C	0.34817336287773	-4.78067782603878	-10.75900509921540
C	0.10394030193786	-2.49758180638130	-9.59600939812856
H	-2.42659904982250	-4.76838288615780	-10.29570318145026
H	-2.00930897579720	-3.29353676344431	-11.23534267223384
H	-2.43955661695609	-3.13331121872269	-9.47507134593704
H	-0.55020753076929	-2.02677159306221	-8.82411387068043
H	0.04188573003366	-1.92473186411063	-10.54313799238826
H	1.16098653966103	-2.53463152235282	-9.27184675977592
H	-0.10137959931773	-5.79167791243009	-10.84371681380579
H	1.38786100054600	-4.88069719600468	-10.39142625037407
H	0.37225436055423	-4.32914851783621	-11.77139239349896
F	-2.12440562355890	-1.96327118822505	-7.70729695679420

F@Cu$_{top}$:

Cu	-0.62567917250391	-2.90534016349440	-5.52807893459879
O	0.90813310034436	-1.32739523900327	-1.95317287103313
O	-3.35957828677304	-3.50644853859024	-2.42235132795710
N	0.52929757230456	-2.40603681043856	-3.90309844360302
N	-2.06360635149704	-3.37806912042135	-4.26095849668306
C	1.86945294474929	-1.80265901305349	-4.07559507324751
H	2.60723964159439	-2.61191941684771	-4.25190418680743
C	1.83731385279207	-0.76492060278249	-5.17001894142478
C	1.62803415950566	-0.99415749066491	-6.53799306820889
H	1.53622477051478	-2.02510820199975	-6.91334581080759
C	1.49625971074032	0.11859724994675	-7.38564198566238
H	1.30621194712218	-0.03084088490317	-8.45857208460626
C	1.57950960035619	1.42433507765436	-6.86343801640474
H	1.46639782735919	2.28663316009180	-7.53883832510317
C	1.79080651306439	1.64386697677984	-5.48907404856632
H	1.84586056069155	2.66902494209848	-5.09068843615834
C	1.91232617339721	0.53434067793774	-4.63563316164304
C	2.11676595683184	0.50794892008212	-3.13362346233411
H	1.32059720754107	1.05509886311382	-2.58700912623903
H	3.08067575147245	0.97322645395973	-2.83966298206224

C	2.09183782485351	-0.99299109378389	-2.75736810817261
H	2.96841943276086	-1.31111945180373	-2.16553262720810
C	0.09298300717180	-2.07813480882024	-2.72940138978491
C	-1.21095630343200	-2.44844269154648	-2.12417454542312
C	-1.76542636122047	-1.53885998641584	-1.00647376519098
H	-1.18057986306391	-0.62887340883953	-0.81130978519342
H	-2.85863611082596	-1.42334898085359	-0.99514783895455
C	-1.18955591443182	-2.85663858614311	-0.63144454331065
H	-1.87136204725089	-3.67598010688748	-0.36126732551673
H	-0.19079424814657	-2.88691898875697	-0.17219077564436
C	-3.23862454401776	-4.15145266646017	-4.68553248159463
H	-3.62411617544952	-3.75130724295661	-5.64453550865653
C	-2.91779805041106	-5.63394225891512	-4.73022827858166
C	-1.85875268471435	-6.25758222482844	-5.40851317521290
H	-1.09888432703101	-5.65729399865888	-5.94475395815069
C	-1.72624336126522	-7.65313002725334	-5.27900117592518
H	-0.90282486269840	-8.17162229572642	-5.79482078343301
C	-2.62117094174966	-8.39166964752021	-4.47802101805217
H	-2.50059676499629	-9.48273808257659	-4.38931967309320
C	-3.64634847129508	-7.74544550272183	-3.76114843720463
H	-4.31631302663401	-8.32196071019565	-3.10414670401917
C	-3.78492966625869	-6.35319497320091	-3.88266626206614
C	-4.76027013242762	-5.42721954358685	-3.18218099408353
H	-4.82459338962302	-5.60146350277593	-2.08894647680897
H	-5.79046034607419	-5.53420636326973	-3.58467879906374
C	-4.23378708555638	-4.01945508851687	-3.49826079776156
H	-5.02914166503928	-3.26360222087243	-3.63927188966859
C	-2.19412311133277	-3.12538518594905	-2.99889575856828
C	-1.50841917018253	-2.11893030421076	-7.02012166381670
C	-1.87390095850570	-2.81030523800464	-8.22718088925008
C	-1.25244901407860	-4.06631819428779	-8.51615787765274
H	-0.48661165512797	-4.41288311258479	-7.78898634295944
C	-1.60806341899366	-4.78205732783684	-9.66390117513307
H	-1.13227227879027	-5.75224437468096	-9.87689163098074
C	-2.56704693640397	-4.26235106180206	-10.55682847709499
H	-2.84124612151968	-4.82754626470374	-11.46137308959181
C	-3.17850283808787	-3.01562163436522	-10.29836746607151
H	-3.92415605669872	-2.61390148477957	-11.00173026513603
C	-2.84728699865026	-2.30110813485649	-9.14556933242328
H	-3.32447497365558	-1.33044975975023	-8.94121314706647
C	-2.08302085377257	-0.82081421314785	-6.70805316975096
O	-1.69710218553052	0.18871286213076	-7.31337528472812
O	-2.96225459244561	-0.82028336703907	-5.67455612436732
C	-3.14381638593626	0.38310811772324	-4.83140748979507

C	-4.10717866619260	-0.09564531684636	-3.74325990302620
C	-3.77473890363121	1.52423141200952	-5.63962734325702
C	-1.77961296289428	0.76005901113339	-4.24300195620182
H	-5.06435211865251	-0.43004703853476	-4.19076070829876
H	-4.32038072576521	0.72693446814171	-3.03271377466710
H	-3.67314806709872	-0.94000199015108	-3.17649408439958
H	-1.35207565913486	-0.10837452312223	-3.70625241495666
H	-1.88761547189361	1.59819306251596	-3.52660387393257
H	-1.06716480063827	1.05648390734484	-5.03578063647880
H	-4.70909682754801	1.17781403987869	-6.12589078705271
H	-3.08190670326947	1.88475974265734	-6.42018190252435
H	-4.02973092778857	2.36266667488328	-4.96002065392937
F	0.53089298343937	-4.38762715734368	-6.25987487596180

F@Cu$_{bottom}$:

Cu	-0.96627201396848	-2.78580556560480	-5.25800176691380
O	2.07956744815443	-1.49051587855335	-2.69247510836419
O	-1.81728183982257	-3.98458121286764	-1.33497839939563
N	0.51848155317398	-1.86863154190243	-4.27695536841275
N	-1.92840536523671	-2.81698823555508	-3.27932675827330
C	1.48738561127331	-0.96557812837233	-4.91584896498764
H	1.76402342226290	-1.36479536797913	-5.91292909393420
C	0.96177116399278	0.45606156399918	-4.96425164365652
C	-0.25241112005546	0.89175755069845	-5.51707950395954
H	-0.96556514133856	0.15316887245921	-5.93499681167008
C	-0.56422805952079	2.26094826467114	-5.41672819453938
H	-1.51098754318482	2.63744332720436	-5.83545131315990
C	0.31270536680071	3.15516360246150	-4.76819653001095
H	0.05033539789957	4.22263266818017	-4.69936833053792
C	1.50700007586356	2.69475661570253	-4.18145861467262
H	2.17083162652339	3.39255425130912	-3.64723787553425
C	1.82621045215937	1.32995981350040	-4.27498362336250
C	3.01748827671782	0.58689634815358	-3.70227068548100
H	3.20435870058421	0.81451106809197	-2.63302737510719
H	3.95372029722680	0.83022021660152	-4.24893630922331
C	2.67241528484139	-0.89718851617443	-3.90688877396161
H	3.54376260805088	-1.52694282408247	-4.16842544598448
C	0.90058316846154	-2.05290974571134	-3.05409842446007
C	0.20691286979398	-2.86367110024915	-2.02181532519911
C	0.64710407563450	-2.69397898523681	-0.55707485552915
H	1.39157496001406	-1.90573073700513	-0.37664221630015
H	-0.15921949802530	-2.77740837501536	0.18560547662802
C	1.06810948352379	-3.92099531256563	-1.28673744374372

H	0.56008186668094	-4.87042161845648	-1.06313487335136
H	2.10889138450524	-4.00110277497588	-1.63349803418173
C	-3.27829621407385	-3.39360683469344	-3.11707736734667
H	-4.01884782232914	-2.56778271714511	-3.08032126628397
C	-3.58532838241785	-4.40464016564930	-4.19809888354703
C	-3.69911489645824	-4.16031352534369	-5.57667907203613
H	-3.56403213672026	-3.13745522999251	-5.96774583549737
C	-3.90051536658162	-5.25860768599262	-6.43087610647583
H	-3.96316781230218	-5.09569695188684	-7.51599490421009
C	-3.96614272497359	-6.56619596014291	-5.91058383003763
H	-4.10552903003112	-7.41767886804471	-6.59432071140555
C	-3.82821035360068	-6.80205724126003	-4.53100252729557
H	-3.85057739719754	-7.82937367036965	-4.13641368778077
C	-3.63016948930226	-5.70779417786293	-3.67085510019018
C	-3.42317933547221	-5.71280756477177	-2.16976062045619
H	-2.55066476693453	-6.33033333716557	-1.86979044252330
H	-4.30168384391298	-6.12459817855645	-1.63089691019720
C	-3.19095359941248	-4.23031664421100	-1.79631970165722
H	-3.85618526021198	-3.87714871858234	-0.98777652176821
C	-1.22022237066359	-3.19612228439478	-2.26844861665209
C	-0.66358428873883	-4.40882138172956	-6.13791616448046
C	-0.61378759024367	-5.77067956153955	-5.67484873205778
C	-0.50574952420467	-6.03106717306334	-4.27913160623031
H	-0.44750524928459	-5.16842117026776	-3.60316792995274
C	-0.44089982697621	-7.33867011379543	-3.79172947334262
H	-0.33565398340481	-7.52386152327605	-2.71211521790431
C	-0.52250151321614	-8.42131948473751	-4.68866676126649
H	-0.48536062611250	-9.45311270783898	-4.30655538949596
C	-0.64887274382851	-8.19163248903148	-6.07400464631025
H	-0.71634653731133	-9.04312978946148	-6.76791117017838
C	-0.68095031425413	-6.88384039618580	-6.56474996681060
H	-0.78185034232404	-6.69842463131885	-7.64490222486622
C	-0.37650435433937	-4.07866138323482	-7.53456509513258
O	0.77304840981658	-3.78924848029150	-7.89192480996234
O	-1.47462234696872	-4.03466865073986	-8.31155719304749
C	-1.56966336915066	-3.07679893932572	-9.45026237801732
C	-3.06738968604048	-3.07890912418580	-9.76315471772997
C	-0.74937896722506	-3.61428396577707	-10.62675984923196
C	-1.11432175267262	-1.69403648949849	-8.97027148708463
H	-3.41442332035320	-4.10199782504068	-10.01196619005727
H	-3.27539228624682	-2.41888527926549	-10.62856237196093
H	-3.64210462494268	-2.71084249789038	-8.89057827477507
H	-1.55739975868285	-1.49053084975316	-7.96836397369927
H	-1.44605602838280	-0.92321066810462	-9.69489580161239

H	-0.01266163206282	-1.64245148201596	-8.88310571671704
H	-1.09015017997526	-4.63072638116387	-10.91102588963636
H	0.32306386087188	-3.65811656300291	-10.36086338413917
H	-0.87406465368423	-2.94912206433889	-11.50550782354829
F	-2.17271948045184	-1.42766042078497	-6.03716146811077

TS-F@Cu$_{top}$/(*S*)-2b@Cu:

Cu	-0.41171935502757	-2.37410119215397	-5.50508285828541
O	2.15214299561042	-2.11107367259341	-2.19079417862979
O	-2.49844306065469	-3.26797423494565	-2.05502772777308
N	1.07571436292025	-2.16821164721483	-4.17162270653997
N	-1.54065335593367	-3.30425640438059	-4.10313975708731
C	2.38742004822423	-1.56044537863399	-4.47867898783712
H	2.84551764490765	-2.07487647698405	-5.34726972820267
C	2.25916961382337	-0.06062086015878	-4.67126760892224
C	1.46867300343105	0.61160662321976	-5.61514891585361
H	0.86361417759661	0.04716739956276	-6.34140273033918
C	1.43654160668769	2.01724978161610	-5.57307664242050
H	0.81201102635961	2.56631292770414	-6.29440402488174
C	2.18283611056502	2.72427686146815	-4.60881090547128
H	2.14503473292691	3.82472130571533	-4.58876895712515
C	2.96827047632391	2.03895779238080	-3.66196494270032
H	3.54146763106507	2.59629859226475	-2.90458723319302
C	2.99872241633583	0.63520011481708	-3.69321495975513
C	3.75820091773393	-0.31970260046831	-2.79498843463844
H	3.63882923443722	-0.10367122893118	-1.71393536135465
H	4.84831714029415	-0.29347646749500	-3.00800676578686
C	3.19409909439793	-1.70449424718087	-3.15113160425496
H	3.95062090230008	-2.51126191756690	-3.14938756253293
C	1.02915290729766	-2.37042194711190	-2.89225272460712
C	-0.14167884427094	-2.81298788396419	-2.09942752107958
C	-0.27276046318796	-2.20548243254774	-0.68448062447042
H	0.49188163277103	-1.45940736496194	-0.42539388180190
H	-1.30111027838350	-1.97663049836907	-0.37152848814850
C	0.13571216297549	-3.62830015882579	-0.81245652654697
H	-0.60233039275422	-4.41327086996246	-0.59108346138173
H	1.18945992606015	-3.89652794883960	-0.64833236723876
C	-2.94583524731440	-3.69818582979523	-4.33696628133029
H	-3.34949325344454	-3.08392566808431	-5.16966264762000
C	-3.09202675114849	-5.18738817724287	-4.58230160212969
C	-2.52896560084416	-5.94973168575244	-5.61560991914397
H	-1.91282555878830	-5.47312239912860	-6.39072333774016
C	-2.77327185205386	-7.33414019292267	-5.64172709254391
H	-2.34299751889388	-7.94639391654094	-6.44914339180117

C	-3.56636702869921	-7.93978111334618	-4.64653864691248
H	-3.74963033490055	-9.02510332058200	-4.67898995682796
C	-4.12884266502674	-7.16927892608679	-3.61114529588629
H	-4.74901015385894	-7.64555124061109	-2.83570061166501
C	-3.88653464470616	-5.78629818102229	-3.58262687296890
C	-4.40693371051687	-4.75540914805346	-2.60312384517004
H	-4.25130086233191	-5.03174506090216	-1.54055968627156
H	-5.49779484000105	-4.59219389711697	-2.73475158482558
C	-3.64262169187202	-3.47241952027028	-2.96064957696038
H	-4.25667590823459	-2.55424928068969	-2.89187885781944
C	-1.39590706547175	-3.13038468739595	-2.82408094778503
C	-1.71018414337892	-2.13278125305105	-6.96416284137725
C	-2.01176425892777	-3.24229572760102	-7.83780629036402
C	-0.99310039475391	-4.15968894474419	-8.24174124838612
H	0.04548409200316	-3.93108555153236	-7.97124511917060
C	-1.30117249824055	-5.30889309401187	-8.98133045896247
H	-0.48544292657684	-5.98691268460607	-9.27975132534583
C	-2.63066980828789	-5.60978957993625	-9.32992299607965
H	-2.87215688449609	-6.52400144304796	-9.89284904201348
C	-3.64593916126929	-4.71356479220300	-8.94382054221228
H	-4.69512007156808	-4.92539083560678	-9.20615778097957
C	-3.35355256481134	-3.54629018428931	-8.22761223651769
H	-4.15667074502497	-2.86148335447891	-7.93020365890181
C	-2.23528055299818	-0.79877590821857	-6.88511447504320
O	-1.63831722812289	0.19398944683021	-6.43457946740342
O	-3.55848942252360	-0.76860139229016	-7.27775025014782
C	-4.50029909103820	0.18217664876063	-6.65803456730139
C	-5.86701807907695	-0.31934604389739	-7.13485411806283
C	-4.22558165116243	1.59945676971002	-7.17890740999181
C	-4.39333812633215	0.09539560273060	-5.12978135358339
H	-5.91311002839178	-0.32482830333973	-8.24219895522543
H	-6.67101808758892	0.34120483818431	-6.75353595561008
H	-6.05511976852505	-1.34906549714828	-6.77010227450012
H	-4.57778466781336	-0.94423756153425	-4.79128244762499
H	-5.15562996302002	0.74930390162249	-4.66155878917771
H	-3.39258541445076	0.41215679286150	-4.78236351738557
H	-4.25465887733813	1.61101072464045	-8.28730195137368
H	-3.23019157937677	1.94475684937984	-6.84687937486500
H	-5.00225834050295	2.29716627310717	-6.80436206903469
F	0.20579091686934	-1.80623941620477	-7.28604113909587

TS-F@Cu$_{top}$/(*R*)-2b@Cu:

O	1.27873427825707	-1.70394909874523	-2.14302994050765

O	-3.34912652408371	-2.80767147046495	-2.70438615554873
C	2.29774096974819	-2.78887836080509	-3.98964244299873
H	2.81264095601406	-3.76755566797609	-3.90373588906271
C	2.68728590776205	-2.01532403375467	-5.22637255370621
C	2.66414908822212	-2.46842776120103	-6.55041973607428
H	2.41833308944573	-3.51421584553452	-6.77982703020156
C	2.94808208569235	-1.55916139381176	-7.58326552859104
H	2.91421089554056	-1.89671888809325	-8.62989221052937
C	3.26023252072358	-0.21980134808033	-7.27890347796802
H	3.47854347255139	0.48671224573102	-8.09464008083232
C	3.30093116544130	0.22715678734349	-5.94364758373859
H	3.55287715343563	1.27431627962702	-5.71512312595085
C	3.00796377858816	-0.68052568658820	-4.91242331297987
C	3.00020691792025	-0.44653905975033	-3.41627491616530
H	2.28815505359333	0.34888571833394	-3.11220617767220
H	3.99911440213894	-0.13701912513129	-3.04529287041064
C	2.57561353934057	-1.80528730866353	-2.80787663599986
H	3.28718271726244	-2.18769565376837	-2.05448852422296
C	0.37261103869765	-2.36342036597715	-2.90310287512879
C	-1.03042540530248	-2.21545380970801	-2.44318827016132
C	-1.41155752249631	-0.85452054432708	-1.80999806550525
H	-0.61780304160691	-0.09411468845620	-1.81810438301809
H	-2.42468006295987	-0.50100548813279	-2.05033156185751
C	-1.25770170896351	-2.03961969011910	-0.92572703152031
H	-2.16098050678755	-2.52615646872350	-0.53090089382781
H	-0.35360011354744	-2.11880085638110	-0.30544974238325
C	-3.24997766891719	-4.15346110526656	-4.65182921952562
H	-3.43759783459090	-3.96152642915222	-5.72542642425764
C	-3.33067574618363	-5.61748743116047	-4.26013237207198
C	-2.51989187107629	-6.66729712713928	-4.71779861835643
H	-1.75075630234265	-6.43735501981835	-5.47650182703181
C	-2.73068789551178	-7.95302806886215	-4.18660497402189
H	-2.10756063015305	-8.79554783873176	-4.52631708264050
C	-3.73303954707695	-8.17492100156673	-3.21958812261420
H	-3.88399826434710	-9.18674277861119	-2.81150238649249
C	-4.54343780104027	-7.11538186728619	-2.76908417238487
H	-5.32528559102583	-7.29404786053324	-2.01405593379084
C	-4.33621819931862	-5.82922302650280	-3.29479638357588
C	-5.08642972194243	-4.54755102266059	-2.99522416682974
H	-5.20952699571606	-4.34499641446647	-1.91183992678261
H	-6.10683940227774	-4.56890612858342	-3.43467830022699
C	-4.24994139896370	-3.45417906678661	-3.67705257543926
H	-4.85194973723544	-2.64444214631460	-4.13146456713655
C	-2.09407934730132	-2.90580360067427	-3.20673192854402

Cu	-0.28379205029615	-3.47523441067786	-5.56425955028199
N	0.82579086269358	-3.01491056325645	-3.93426820242396
N	-1.94122519287797	-3.56955004842885	-4.30255724491484
C	-0.51074306725739	-2.98280086321709	-7.37146992447157
C	0.19530753655949	-3.40874091748915	-8.55962386487400
C	1.02850636160307	-4.56590666178903	-8.54531040919799
H	0.99946632660630	-5.19252257446623	-7.64389773041546
C	1.79825682980404	-4.90929847496402	-9.65886864210918
H	2.42496228327434	-5.81424590433223	-9.63097444991339
C	1.78534606271438	-4.09694647310688	-10.81249193414603
H	2.40841854223397	-4.35891131230198	-11.68145527023189
C	0.96207745308057	-2.95635663421967	-10.84956138147887
H	0.93748722044600	-2.32306401889131	-11.75002684814940
C	0.15436761697219	-2.62953587013903	-9.75229113785626
H	-0.48085202261014	-1.73349286781945	-9.78539072153144
C	-1.60455709737337	-2.02578562162973	-7.37667359147650
O	-2.78146496650395	-2.20365316381178	-7.05670686851236
O	-1.08202811185033	-0.79211262379999	-7.71296734108050
C	-1.23566897136258	0.35845201687365	-6.78968590711248
C	-0.26334754844561	1.39245856770527	-7.35750755303778
C	-0.81011343595005	-0.07858548871528	-5.38489424923677
C	-2.68303447956752	0.86238110628794	-6.82351631726268
H	0.76546027426091	0.98280598383761	-7.35700285848294
H	-0.28155448081725	2.31321445346340	-6.74104344529214
H	-0.54162967972221	1.65962064202193	-8.39659163235541
H	-2.98252944056793	1.10357752035767	-7.86321404122868
H	-2.77484970810834	1.78307742408579	-6.21166309293063
H	-3.37170422893644	0.09274727739009	-6.42959166284750
H	0.22191778255240	-0.48080202787698	-5.39910000166006
H	-1.48710856180401	-0.86079610982683	-4.99075064217867
H	-0.84591615640253	0.78695247400485	-4.69457751661512
F	-1.02201282502112	-5.08322373626839	-6.69874097693304

TS-F@Cu$_{bottom}$/(*S*)-2b@Cu:

Cu	-0.28106514818385	-3.36297948683385	-5.56137369467248
O	0.97942310355687	-1.81528535813397	-1.81396582647198
O	-3.60731673153750	-2.37906734012103	-3.08146896399043
N	0.65887900930344	-2.70364106410553	-3.87020601581984
N	-2.08542251080227	-3.09132745071856	-4.59574806150956
C	2.12347069112953	-2.53993402330880	-3.73736278335257
H	2.63525270994483	-3.47801849729112	-4.02975130426804
C	2.64160378023351	-1.33506403300777	-4.50224359446884
C	2.50614835794260	-1.06335726978150	-5.87238768810557
H	1.92449627517025	-1.73703844864154	-6.52438398635520

C	3.05377374547583	0.13507179921980	-6.36726084822952
H	2.95646399430999	0.37595325038651	-7.43755340260972
C	3.71250173659072	1.03689844622124	-5.50723114387793
H	4.13358234507264	1.97022657760063	-5.91276019336991
C	3.82267078015503	0.76458485802611	-4.13064834957086
H	4.32161855226751	1.48004388807465	-3.45828026950276
C	3.27807581788577	-0.42870794942673	-3.62878364269041
C	3.27085634953261	-0.93849641731540	-2.20238920483160
H	2.91819601896318	-0.18816349473359	-1.46574702478870
H	4.28645974400019	-1.25147969588713	-1.87849767049808
C	2.34130111650191	-2.16043853041870	-2.24380039133907
H	2.67232658776910	-2.99538447680029	-1.59724805597019
C	0.12966019913363	-2.22519249122559	-2.78667650938766
C	-1.30890577824979	-2.04635974738861	-2.47378388003505
C	-1.68330218951863	-0.78866524232075	-1.65115272107038
H	-0.84851656175093	-0.10621139899171	-1.43598085802740
H	-2.63531129647247	-0.31618660139638	-1.93332459997884
C	-1.71365152314202	-2.11534027570375	-0.98254154854708
H	-2.68771025446475	-2.58506110491696	-0.78512939220548
H	-0.89930426778882	-2.37600780389096	-0.29173180368806
C	-3.39039765043309	-3.53052614173345	-5.12248594307932
H	-3.45179872270220	-3.26647192817293	-6.19513961493178
C	-3.63410625810727	-5.00769284946464	-4.86987001455571
C	-2.84547937749187	-6.09537176643618	-5.27399927194542
H	-1.92112337906833	-5.92736940628061	-5.84582010318356
C	-3.24631040660280	-7.39159460997469	-4.90448744734862
H	-2.64127870289449	-8.25812169685671	-5.21289322357442
C	-4.41328374897332	-7.58859334384518	-4.13910645881207
H	-4.71499823595454	-8.60925756561641	-3.85673512247376
C	-5.19221437448492	-6.49228118377220	-3.72377787392531
H	-6.09643557945247	-6.64979889797340	-3.11525990743689
C	-4.79406457123307	-5.19615711082752	-4.09009810285785
C	-5.46237335615015	-3.87435545696435	-3.77248891871962
H	-5.73001171849688	-3.75754102047235	-2.70280493489712
H	-6.40113810425369	-3.74794607895975	-4.35337484371136
C	-4.43677310122851	-2.82065932930319	-4.21403207955650
H	-4.88948820678229	-1.90864541174794	-4.64826637696418
C	-2.31079813704204	-2.53059194656488	-3.45172499491702
C	-0.08460331941203	-4.48540828533395	-7.09615105048959
C	1.28317102717077	-4.71436725758763	-7.54786503112307
C	2.31147284247244	-4.83741074607085	-6.57513938463669
H	2.00950652850434	-4.85766452399117	-5.51656886869358
C	3.65857509362882	-4.94291188985435	-6.94332161168899
H	4.43859189529500	-5.02638931308160	-6.17094455583548

C	4.00793574494596	-4.95802129585566	-8.30622403775171
H	5.06419350918823	-5.04655150551039	-8.60436420058153
C	3.00290093517307	-4.86913615803438	-9.29236466496512
H	3.27905711243028	-4.88192119599090	-10.35825685821239
C	1.65975590173227	-4.74706233028159	-8.92046390286353
H	0.88137263799216	-4.66045012481742	-9.69268617198320
C	-1.16934958558540	-4.76268794604458	-8.02546956279314
O	-1.23599526391066	-5.87955698626663	-8.56748161190424
O	-2.11947801117935	-3.80332555358575	-8.13309135952821
C	-3.26258430640720	-3.95988227214411	-9.05913520104618
C	-3.99045234446676	-2.61749556271710	-8.93415147659609
C	-4.16162748413247	-5.11650169019938	-8.60344578796122
C	-2.74716393619348	-4.15400433329336	-10.49184157445003
H	-4.35732116870405	-2.45841794563572	-7.90024282626755
H	-4.86318807854311	-2.59324625142838	-9.61595367451465
H	-3.31125166139604	-1.78217207527775	-9.19537869619938
H	-3.59936071632368	-4.12345100167270	-11.20003923500999
H	-2.22780094686071	-5.12310087579452	-10.60074527120760
H	-2.04471914433082	-3.33887030189327	-10.75899401434670
H	-4.49512976614530	-4.96811806776350	-7.55755233627440
H	-3.62263843979900	-6.07865666589270	-8.66417933293969
H	-5.06155408672244	-5.16330715598936	-9.24943328126697
F	0.11560776957801	-2.31997518035659	-7.19863501842131

TS-F@Cu$_{bottom}$/(*R*)-2b@Cu:

Cu	0.45843002409488	-2.56175124875076	-6.20048368775989
O	1.81581362184101	-0.51349475355130	-2.83012648352355
O	-1.61269619525276	-3.75352721272244	-2.75669761592431
N	1.33616334815247	-1.43375498056575	-4.83238576037017
N	-0.94161632207820	-3.04658517385230	-4.79944441867799
C	1.94362457741455	-0.13489796125630	-5.15765661363959
H	2.69305290303170	-0.25304960075105	-5.96467218192837
C	0.86462867035200	0.88027358191167	-5.49823403521438
C	-0.15012286443334	0.76500589397911	-6.46216425173809
H	-0.26127874646474	-0.13952664476532	-7.08575748136575
C	-1.07961652691140	1.81631194116332	-6.57104735750523
H	-1.88617687943246	1.75023907756300	-7.31788886194577
C	-0.99868432613502	2.94289964045845	-5.72762170784124
H	-1.73619953092659	3.75459340036268	-5.82811296016942
C	0.00083402136491	3.02930117279074	-4.73993979253750
H	0.04245831605543	3.89667711278057	-4.06255868407572
C	0.93132712903911	1.98367603977216	-4.62276338175057
C	2.06514121313621	1.82716571550029	-3.62812268011385

H	1.76923332769415	2.04517212856293	-2.58201865125857
H	2.91504931418298	2.50189151636356	-3.86828786230555
C	2.50995647290109	0.36696493888216	-3.79668200676277
H	3.59425150536815	0.20381029291582	-3.64816917091114
C	1.22513357738011	-1.49820796943490	-3.54520937558868
C	0.47643155778967	-2.54778960853729	-2.80949014737317
C	0.39035352428009	-2.43380969111249	-1.28365546345634
H	0.85487587039373	-1.53514463024337	-0.85427115417437
H	-0.56620486864788	-2.74185685566163	-0.83971361118382
C	1.27358587537885	-3.48259217590197	-1.86523255823043
H	0.94008084370738	-4.53114139113276	-1.84814410327307
H	2.36346343535166	-3.33012327204087	-1.85919679135115
C	-2.29230804924790	-3.60818186156580	-5.03043536852340
H	-2.96212108351730	-2.76301848876514	-5.29385142787337
C	-2.37671128744489	-4.72621882319662	-6.04204587767056
C	-2.37697944762299	-4.61766748716830	-7.44023796378212
H	-2.19366597234681	-3.63203601085121	-7.90327118738381
C	-2.53603245476682	-5.79186636869891	-8.19623700958107
H	-2.52950010117204	-5.73818516539662	-9.29553909989032
C	-2.66584666617731	-7.04164149918885	-7.56119066372066
H	-2.77717655824220	-7.95301464642334	-8.16915896370740
C	-2.64498026901987	-7.14312839930233	-6.15687232428719
H	-2.74625196024812	-8.12446438713904	-5.66718906655457
C	-2.50410035090697	-5.97223027274301	-5.39462319091495
C	-2.48106302828850	-5.81832048943768	-3.88605606767004
H	-1.51606370167279	-6.16797544631951	-3.45851182661372
H	-3.28292216523879	-6.39219079908150	-3.38008721111769
C	-2.62422782308735	-4.29238975424339	-3.66784479841887
H	-3.60620419317314	-4.00967664448283	-3.24817602820636
C	-0.69515581299996	-3.12068931740934	-3.52926305930681
C	0.69685987507167	-3.09852478416679	-7.97321610285341
C	0.65373443794146	-4.49168905906282	-8.42118971219444
C	0.68119912865979	-5.48658522274036	-7.40008087158041
H	0.69429771560015	-5.13587339668409	-6.35565825879630
C	0.68493142270570	-6.85001800970928	-7.70032155820121
H	0.70424923682589	-7.59406894518348	-6.89017698393419
C	0.64136445754557	-7.26523843434517	-9.04497569960978
H	0.63190600337049	-8.33861589556971	-9.29093829760440
C	0.60052740327536	-6.30692245261703	-10.07488499789323
H	0.56061524202217	-6.63382670718320	-11.12577501918907
C	0.61038555171022	-4.93857362120397	-9.77242752302186
H	0.57065460608710	-4.19110095108853	-10.57418269044211
C	1.02859561345682	-1.97689005845915	-8.91482724824853
O	1.76924336830342	-1.05077686855268	-8.57661549531327

O	0.49301428421334	-2.08142459754130	-10.15285896503745
C	0.33974568883420	-0.90092401934212	-11.02913729485231
C	-0.54740326163696	-1.43563915108863	-12.15765317511328
C	1.70651485444143	-0.46170074250417	-11.56703575593901
C	-0.38120424527089	0.20780614500793	-10.25037541397552
H	-0.05811344255741	-2.29005466013331	-12.66736310498175
H	-0.73339800194510	-0.64133970963176	-12.90768204187120
H	-1.52118607587310	-1.77772982986423	-11.75490700286196
H	-1.20973195894943	-0.24645139967971	-9.67220933401233
H	-0.77485786122494	0.96905134215519	-10.95313265034753
H	0.29744132239327	0.70056276181916	-9.53027757355526
H	2.20315943785765	-1.29950097471804	-12.09757990725845
H	2.35687513859455	-0.12586903273130	-10.73933958577866
H	1.57533628387979	0.37299703653575	-12.28565256962591
F	-1.12333028421019	-1.96589649088176	-7.98296027997370

(*S*)-**2b**@Cu:

O	1.53086939047331	-1.96328093225550	-2.12461746905958
O	-3.07092325533151	-3.20027993134381	-2.60984403158092
C	2.31181784192498	-2.03523686920685	-4.35097058358812
H	2.83127115630312	-2.81377259124300	-4.94494883863882
C	2.53943522290394	-0.63874967194365	-4.89739984408670
C	2.13879545012504	-0.12873529173812	-6.14046548989446
H	1.55174324735144	-0.74143106783945	-6.84094914151083
C	2.46768836748851	1.20346755374923	-6.45134840147711
H	2.17442294113151	1.62117348895078	-7.42628650359911
C	3.15931814505532	2.00863916140965	-5.52450380122485
H	3.40500660947005	3.05045297284534	-5.78284071030642
C	3.53005466421092	1.49695081817486	-4.26688616326271
H	4.05876123727627	2.13282260157638	-3.53967022145557
C	3.21725406249097	0.16344644527757	-3.95631005395881
C	3.53566835489512	-0.61174704366466	-2.69465234290946
H	3.21231363344352	-0.09575119841801	-1.76754991542217
H	4.62706781505809	-0.79217109017137	-2.59523496382447
C	2.79730446133073	-1.94778530963567	-2.87175318589639
H	3.37400734128345	-2.82470682274521	-2.52235770868216
C	0.54257661514338	-2.26311886507023	-2.99601774331165
C	-0.81035952333486	-2.39167434304788	-2.39771274625679
C	-1.21779193228564	-1.28941520411896	-1.38442514126815
H	-0.48278324884338	-0.48218770642938	-1.25000741034161
H	-2.27120387783451	-0.97737928927430	-1.43634524926636
C	-0.89230517283783	-2.64988420549573	-0.88094664369424
H	-1.71675975653252	-3.30397032376814	-0.56406325584967

H	0.07336868505681	-2.80774227565531	-0.38083927646475
C	-3.09123665188387	-3.89650180427541	-4.87052929762501
H	-3.45532455968686	-3.43331171066982	-5.80941443008839
C	-2.92539805038118	-5.40153945207101	-4.94248994017201
C	-2.02930139243336	-6.12896743124607	-5.73932268247681
H	-1.36231182363000	-5.61255051635884	-6.44412551351021
C	-1.99936442452934	-7.52918397373915	-5.61208515424551
H	-1.30759128129183	-8.11729813930879	-6.23445375400740
C	-2.84684013390823	-8.18112092836477	-4.69374237615012
H	-2.81261677704434	-9.27807480455039	-4.60351476158782
C	-3.72985021188185	-7.44331982456635	-3.88214055411895
H	-4.37939922164824	-7.95679092017567	-3.15597817758104
C	-3.76159509961947	-6.04459875038386	-4.00679609138231
C	-4.61172876064567	-5.04202872655044	-3.25204564924465
H	-4.60387502669396	-5.19290136397385	-2.15346842951827
H	-5.67408115211824	-5.09107138157960	-3.57395631221714
C	-4.01853765758363	-3.67702636220777	-3.63592954764391
H	-4.77438488833341	-2.87822150467260	-3.75670518738003
C	-1.89252879569632	-2.96172352023153	-3.23400580237893
Cu	-0.39435293522171	-2.60663302659525	-5.76070230042734
N	0.87473608476482	-2.37185850070249	-4.24830752611820
N	-1.82244522860077	-3.25098058720544	-4.49249915264952
C	-0.71819382486442	-2.24524326763839	-7.69029495196525
C	-1.01780817170569	-3.58356625472376	-8.26544267831569
C	0.07028881158659	-4.42516331989291	-8.62678586736995
H	1.09586614090601	-4.05019558064357	-8.49251293973763
C	-0.14466192008921	-5.71366649762064	-9.13512573238414
H	0.72111141491656	-6.34440552438112	-9.39339009421446
C	-1.45243359118103	-6.20439442883856	-9.31502124106508
H	-1.62070055583959	-7.21952402708736	-9.70584291968862
C	-2.53804724088063	-5.37486804006000	-8.98282746402945
H	-3.56913588348981	-5.73733935192332	-9.12197107394602
C	-2.33083822457638	-4.08738295325595	-8.46508721356574
H	-3.18143001120627	-3.44695169911801	-8.19914112142502
C	-1.77877370459860	-1.27208685552805	-7.39829979597273
O	-2.95718931965558	-1.56184995673882	-7.11378869882338
O	-1.31801477185474	0.01913726755093	-7.39913070917734
C	-1.92322879859539	1.00373628649797	-6.48136260488413
C	-1.00362635655316	2.22076403360031	-6.61155554398403
C	-1.87291251619124	0.44408193642296	-5.05157402927309
C	-3.34953448089190	1.36498942509778	-6.92015237387490
H	0.02290864456265	1.96920614719199	-6.28486026127904
H	-1.38217909960079	3.05295207516470	-5.98520195349375
H	-0.96332550733688	2.56501346532730	-7.66466896678977

H	-3.34945201275735	1.69994439275728	-7.97744440294369
H	-3.73296697177327	2.19767002813220	-6.29542597117336
H	-4.02074717917624	0.49395430390758	-6.82564814472040
H	-0.83709482946329	0.13128982547399	-4.80566806038756
H	-2.53863742204738	-0.43276181403022	-4.94397688386439
H	-2.18878991111061	1.21998110902048	-4.32606932410934
F	0.41331654426586	-1.67063081677724	-8.34921854885448

(*R*)-2b@Cu:

O	1.54790375779000	-1.81305036527674	-2.27124408223808
O	-3.11853755444574	-2.82187796060950	-2.61205688274735
C	2.37709802511647	-2.43987793614795	-4.40472334376386
H	2.85295601443252	-3.41711254812608	-4.62546330721126
C	2.72088852552311	-1.38028431142010	-5.42486203410779
C	2.57234045878749	-1.45676623946599	-6.81578221031501
H	2.16817632454879	-2.36205911783190	-7.29073605326315
C	2.94097722208471	-0.34475394311006	-7.59184767164111
H	2.83090456361396	-0.38679115377319	-8.68513650704986
C	3.43803490378387	0.82124016710876	-6.97746720058160
H	3.71860349960022	1.68639885005730	-7.59791049317435
C	3.57567950913624	0.89468681734942	-5.57867817469988
H	3.96508267115316	1.80869885542862	-5.10389739958567
C	3.21456286994933	-0.21772229691052	-4.80029228983482
C	3.31132569152182	-0.38784629682056	-3.29832761501732
H	2.69320942243224	0.35042211509559	-2.74586635676731
H	4.35305608212473	-0.26374939109779	-2.93731997427068
C	2.79866316312841	-1.82126467166242	-3.03327709576021
H	3.50966225815944	-2.43876511609101	-2.45578040439977
C	0.56438783409656	-2.26104197747559	-3.08123259319495
C	-0.79670541683736	-2.21333432283427	-2.49261419411611
C	-1.15045182588976	-0.92014934965290	-1.71087066549176
H	-0.37422282917337	-0.14121952370920	-1.71933146305044
H	-2.18566764318016	-0.57178709960495	-1.83696166063318
C	-0.89927481102268	-2.17449774603579	-0.95434114463700
H	-1.75901698416585	-2.71570344545545	-0.53454987690941
H	0.05300966503223	-2.28365790345386	-0.41687632367147
C	-3.18395022333780	-3.98423871273791	-4.68237989443548
H	-3.48092310021596	-3.61907709934790	-5.68651656497616
C	-3.21162276643896	-5.49132677281119	-4.54751307142357
C	-2.48835393070466	-6.42788603739203	-5.30076267647526
H	-1.85063842325660	-6.08410150625036	-6.12823282309332
C	-2.60832599358237	-7.79096200739464	-4.97483828212547
H	-2.05024515605575	-8.54422111788007	-5.55233533223582
C	-3.44004639011750	-8.19936008451796	-3.91210712624936

H	-3.52500162384801	-9.26944746642732	-3.66655761319893
C	-4.16389270984334	-7.25369264997788	-3.16133588227293
H	-4.81226813808053	-7.57962800869854	-2.33287321211139
C	-4.04337739424667	-5.89124940046552	-3.48237720923321
C	-4.72964339820896	-4.70203343426366	-2.84095083803018
H	-4.60226724492708	-4.66568703099299	-1.73950186300851
H	-5.82297980314066	-4.71587392130757	-3.03535374514563
C	-4.08431641667678	-3.47302586044724	-3.51051504053908
H	-4.81437536211951	-2.69390638047546	-3.79831009166594
C	-1.90881790100405	-2.86658039047292	-3.22279252326636
Cu	-0.33001104463655	-3.07881262063505	-5.73874777385117
N	0.91287824862496	-2.64338593380727	-4.27489655700592
N	-1.84264256634923	-3.44793816520180	-4.37448033010074
C	-1.08147553698216	-3.19016876839997	-7.58528665095078
C	-0.00755058250154	-3.35215609484459	-8.60035786436106
C	0.45232422979691	-4.66647874324469	-8.89157520223814
H	-0.00253031522964	-5.51690011895001	-8.36323597276003
C	1.47409386682494	-4.89392170919216	-9.82350972596004
H	1.80922375915145	-5.92595382807292	-10.01614014717642
C	2.07046556107829	-3.81949513655677	-10.51127610029938
H	2.87325571570075	-3.99698824019573	-11.24333252390189
C	1.61119867310614	-2.51588317566172	-10.25006096986071
H	2.04969128595794	-1.66114759134576	-10.79056437517332
C	0.59148334085243	-2.28069425003726	-9.31516129957171
H	0.25445287264215	-1.25649421277854	-9.11478401948383
C	-1.89201955916408	-1.96869797904414	-7.41356873881907
O	-3.10095891434669	-1.95708669817424	-7.14460381419083
O	-1.13031954291956	-0.82915218466112	-7.48865516752795
C	-1.49900804785081	0.38363550180201	-6.73809638667766
C	-0.23704846525827	1.24413748798454	-6.83745504906484
C	-1.77031446896911	0.02240934030499	-5.27172416573419
C	-2.69818273706397	1.06885988274685	-7.40418038073309
H	0.62207218400643	0.71993023312873	-6.37655097913798
H	-0.39074194676190	2.21047663429666	-6.31709672199426
H	0.01446083523331	1.45048080115896	-7.89677130969122
H	-2.47518090779812	1.27848511639434	-8.46995834852773
H	-2.91421288805997	2.03248787223927	-6.89921103044428
H	-3.59180692436782	0.42120675333653	-7.34896373830194
H	-0.90330625351437	-0.53473920491977	-4.86388700628869
H	-2.67347746990548	-0.60660894393964	-5.17091972275102
H	-1.90633953159982	0.94573024637470	-4.67471600148718
F	-1.96532212417283	-4.31296504661523	-7.64527606480808

NMR SPECTRA

[Cu(O*t*Bu)]$_4$

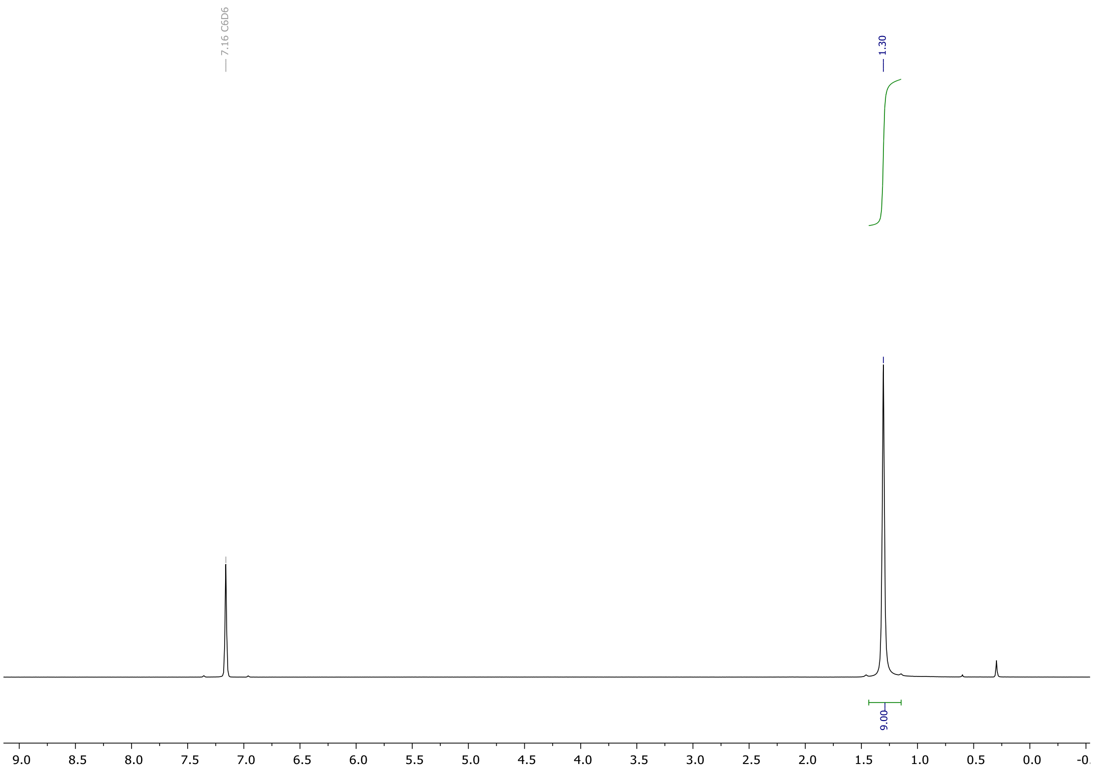

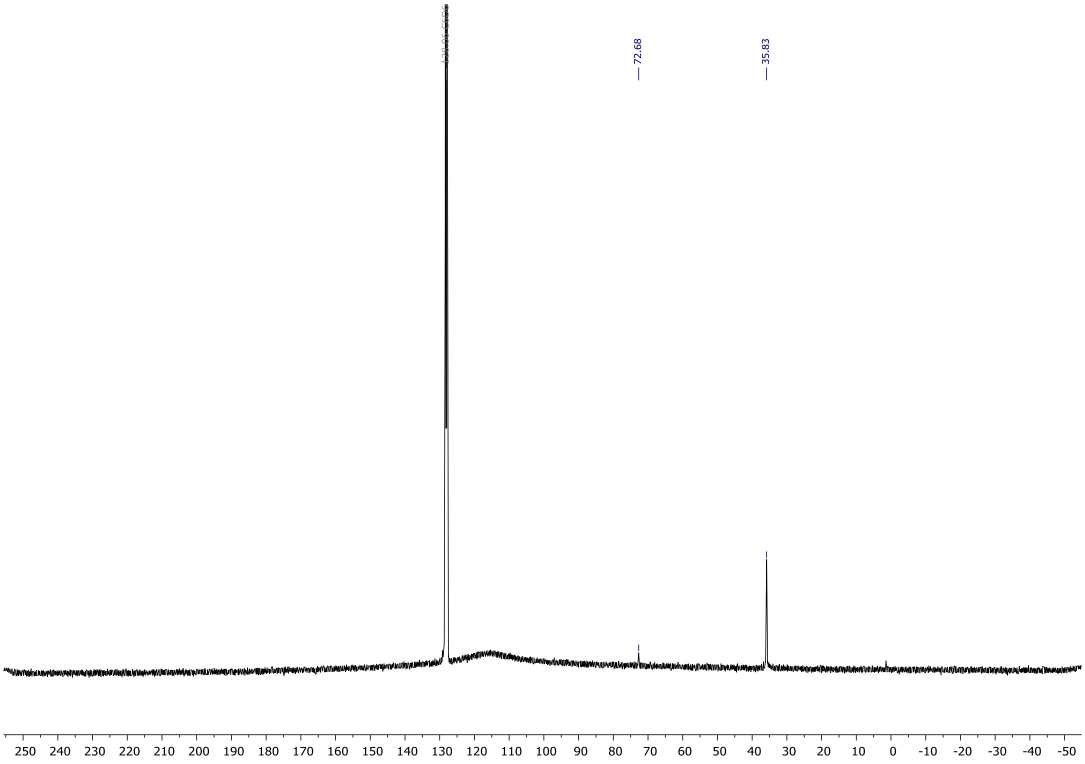

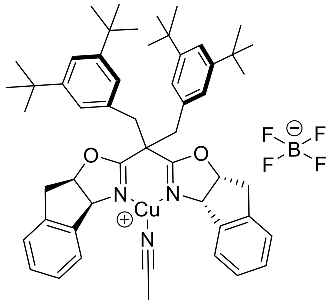

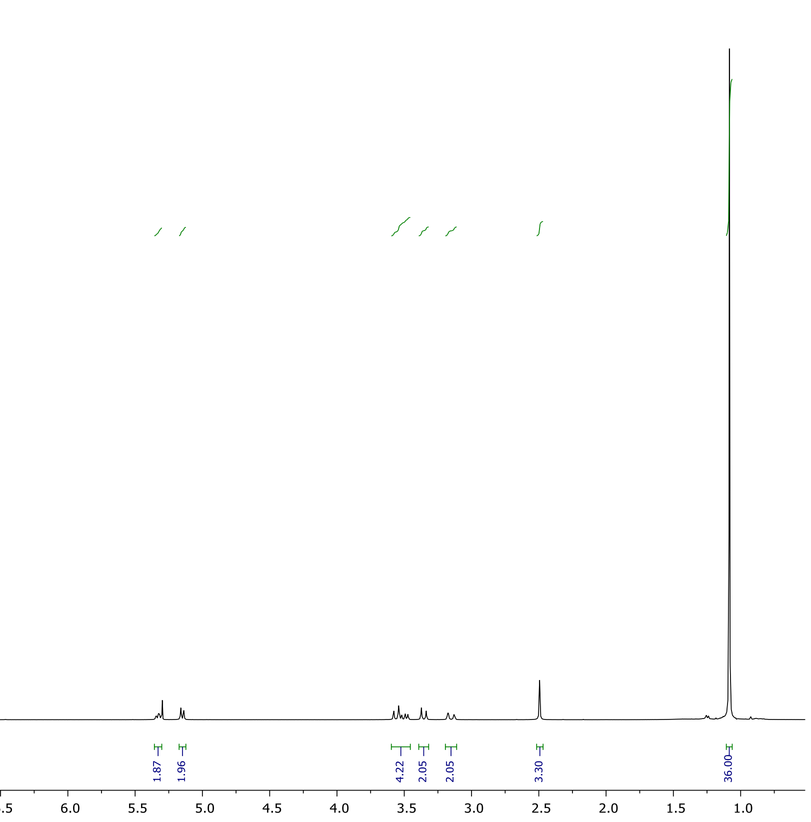

CDCl3
2.05
2.13
2.22
4.11
4.09
1.87
1.96
4.22
2.05
2.05
3.30
36.00
9.0
8.5
8.0
7.5
7.0
6.5
6.0
5.5
5.0
4.5
4.0
3.5
3.0
2.5
2.0
1.5
1.0

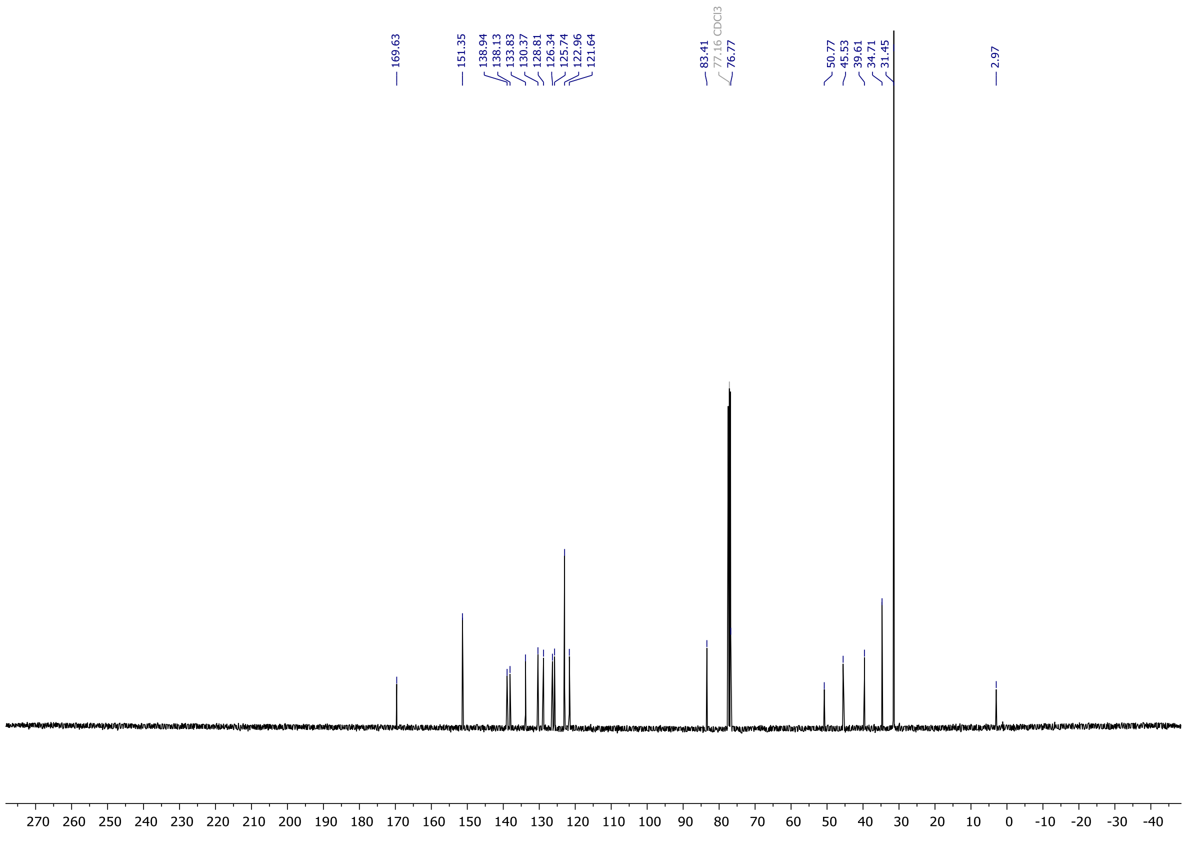

169.63
151.35
138.94
138.13
133.83
130.37
128.81
126.34
125.74
122.96
121.64
83.41
77.16 CDCl3
76.77
50.77
45.53
39.61
34.71
31.45
2.97
270 260 250 240 230 220 210 200 190 180 170 160 150 140 130 120 110 100 90 80 70 60 50 40 30 20 10 0 -10 -20 -30 -40

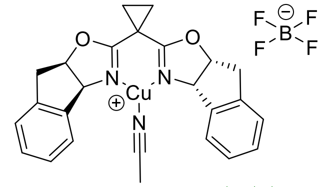

O
N
Cu
N
O
F
B
F
F
F

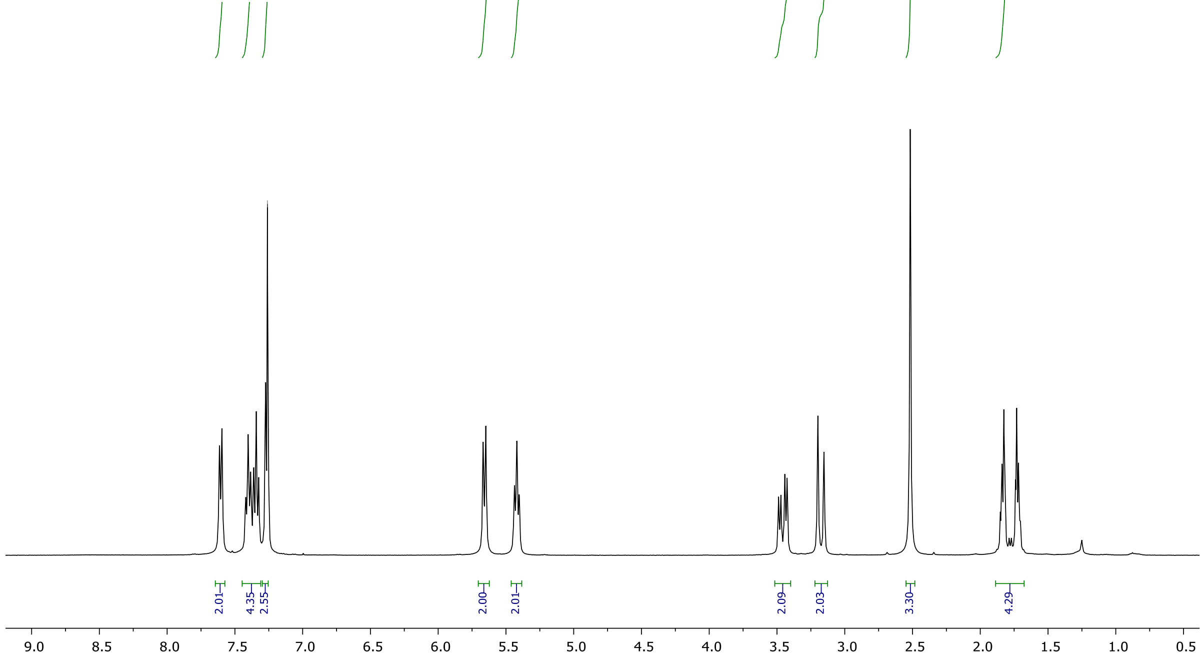

2.01
4.35
2.55
2.00
2.01
2.09
2.03
3.30
4.29
9.0 8.5 8.0 7.5 7.0 6.5 6.0 5.5 5.0 4.5 4.0 3.5 3.0 2.5 2.0 1.5 1.0 0.5

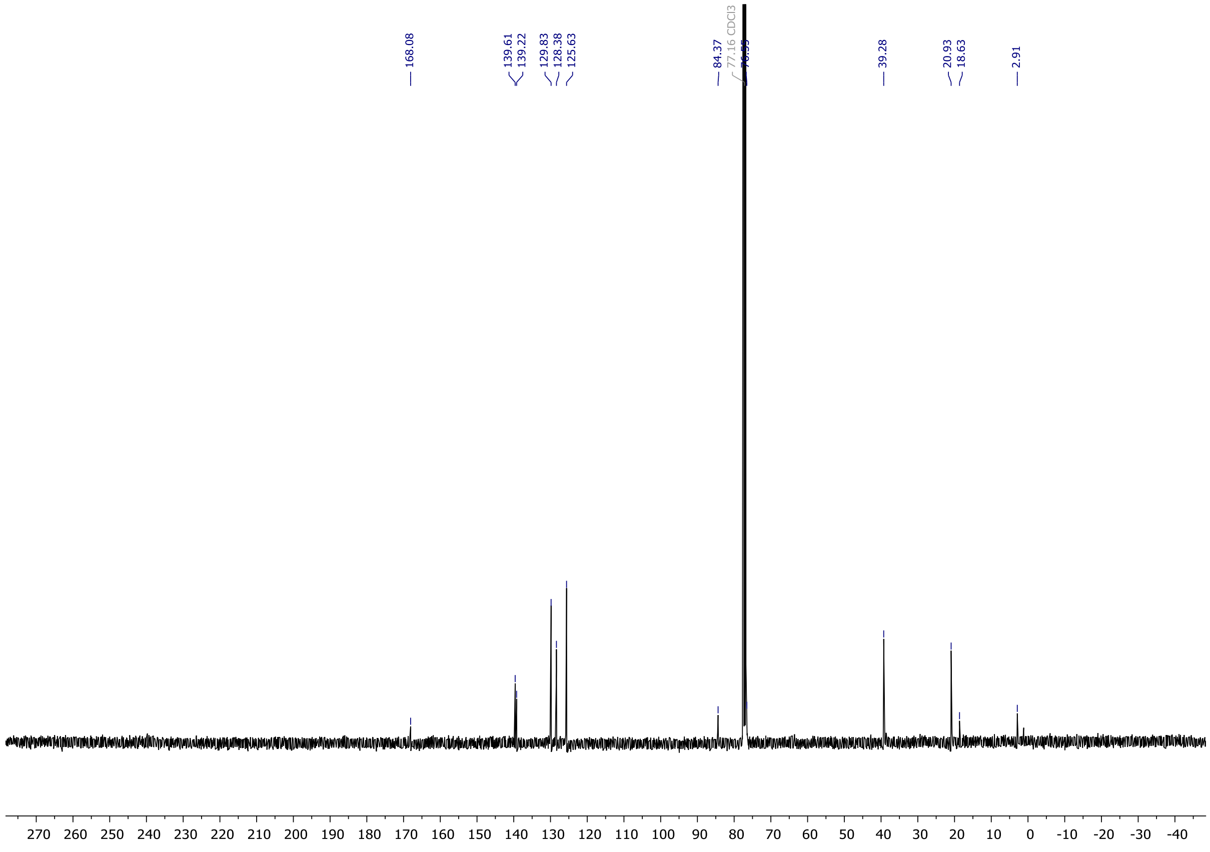

168.08
139.61
139.22
129.83
128.38
125.63
84.37
77.16 CDCl3
39.28
20.93
18.63
2.91
270 260 250 240 230 220 210 200 190 180 170 160 150 140 130 120 110 100 90 80 70 60 50 40 30 20 10 0 -10 -20 -30 -40

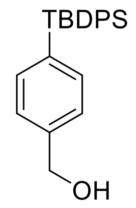
TBDPS
OH

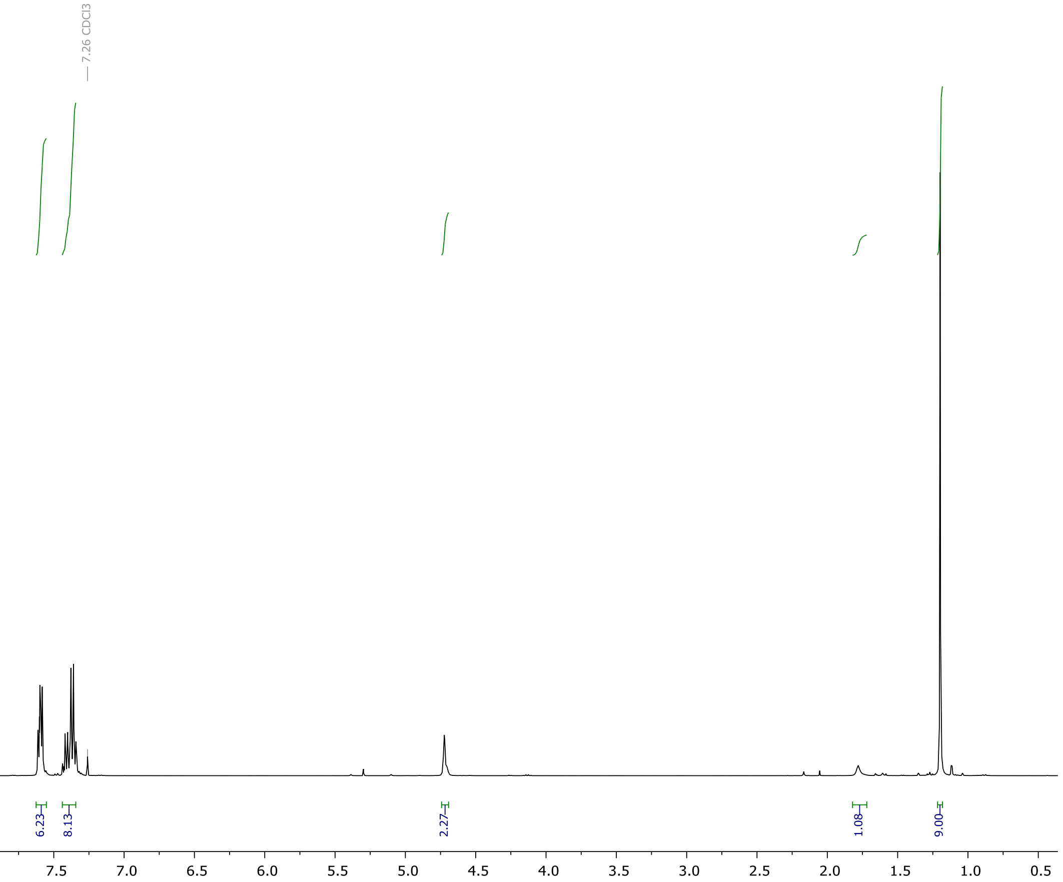
7.26 CDCl3
6.23
8.13
2.27
1.08
9.00
9.0
8.5
8.0
7.5
7.0
6.5
6.0
5.5
5.0
4.5
4.0
3.5
3.0
2.5
2.0
1.5
1.0
0.5

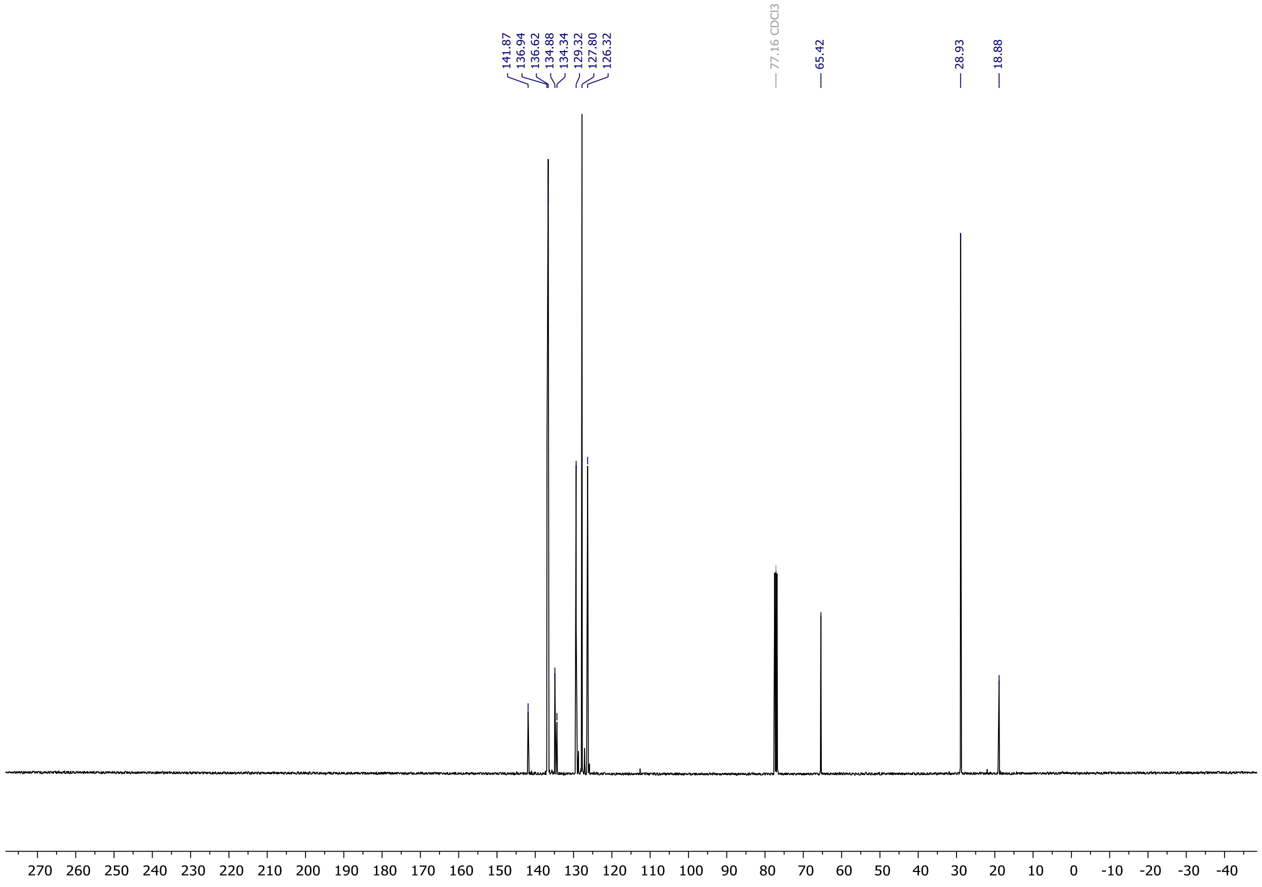
141.87
136.94
136.62
134.88
134.34
129.32
127.80
126.32
77.16 CDCl3
65.42
28.93
18.88
270 260 250 240 230 220 210 200 190 180 170 160 150 140 130 120 110 100 90 80 70 60 50 40 30 20 10 0 -10 -20 -30 -40

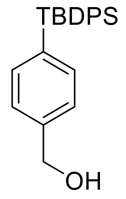
TBDPS
OH

^{29}Si NMR

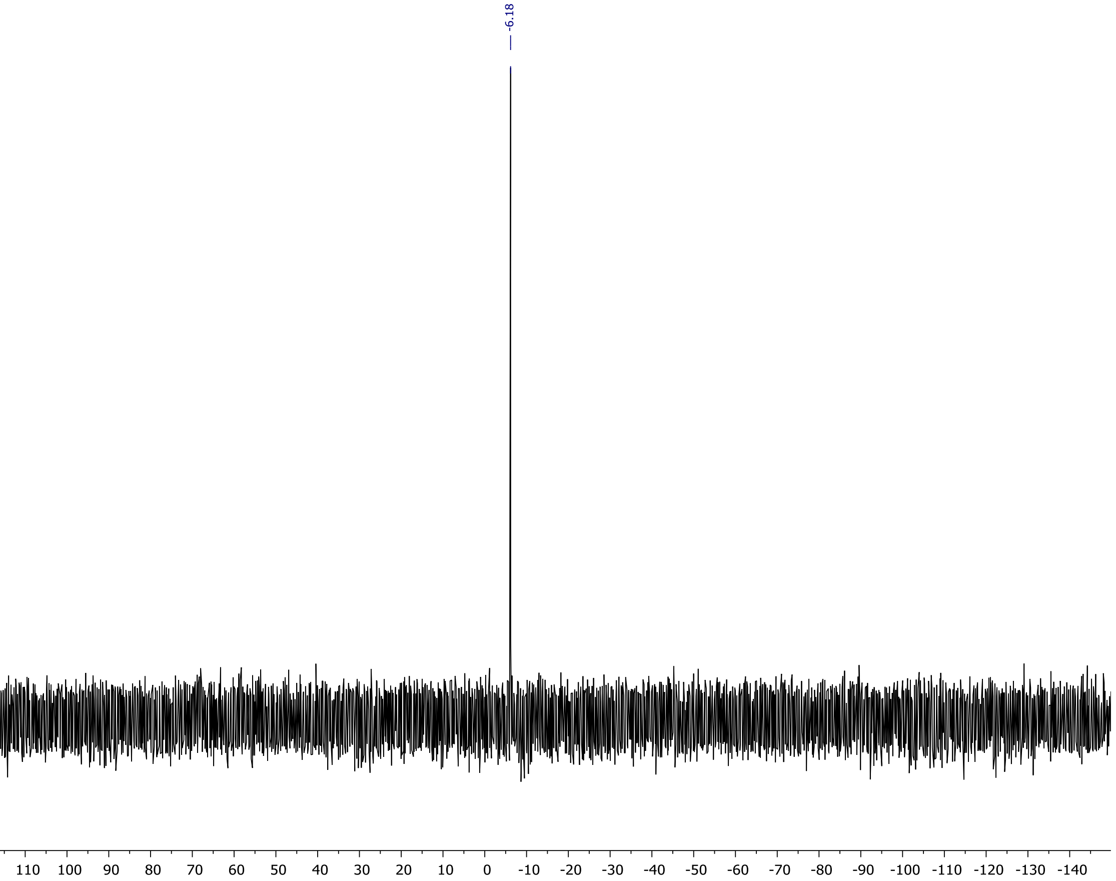
-6.18
140 130 120 110 100 90 80 70 60 50 40 30 20 10 0 -10 -20 -30 -40 -50 -60 -70 -80 -90 -100 -110 -120 -130 -140

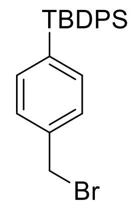
TBDPS
Br

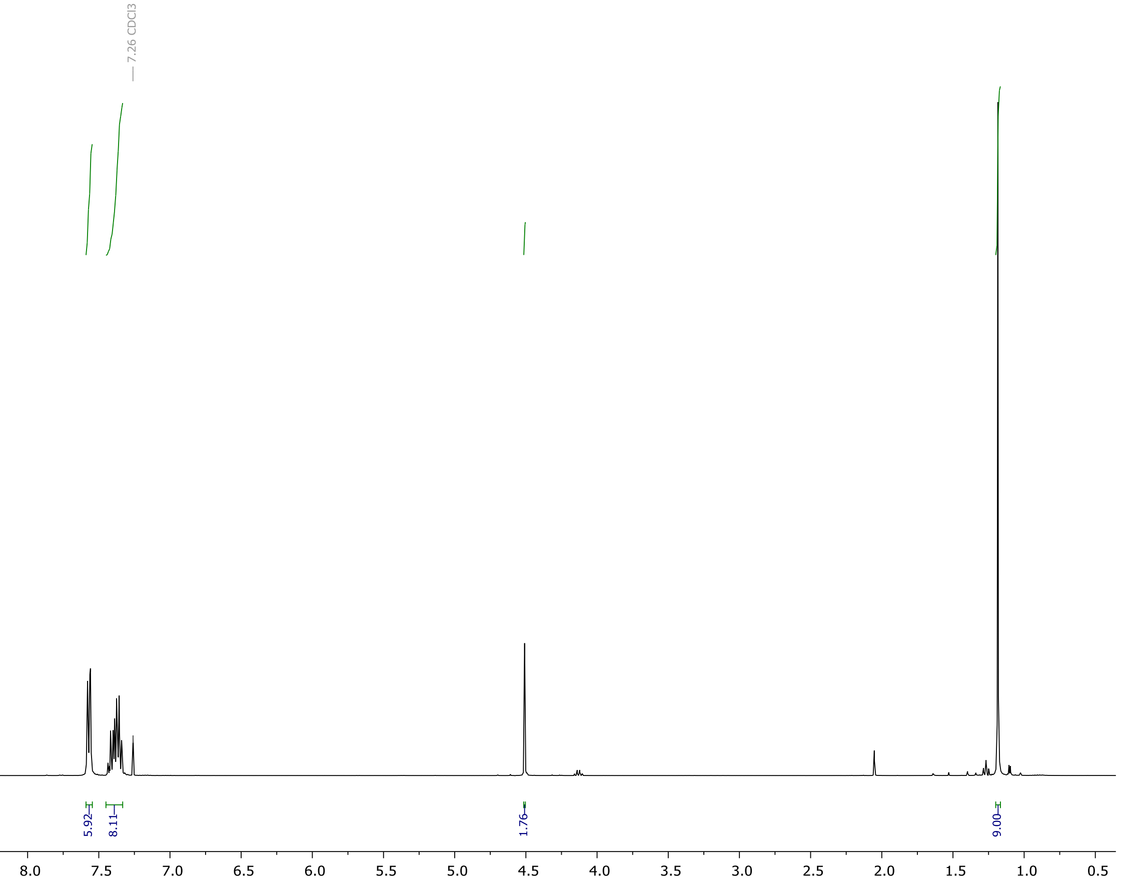
7.26 CDCl3
5.92
8.11
1.76
9.00
9.0 8.5 8.0 7.5 7.0 6.5 6.0 5.5 5.0 4.5 4.0 3.5 3.0 2.5 2.0 1.5 1.0 0.5

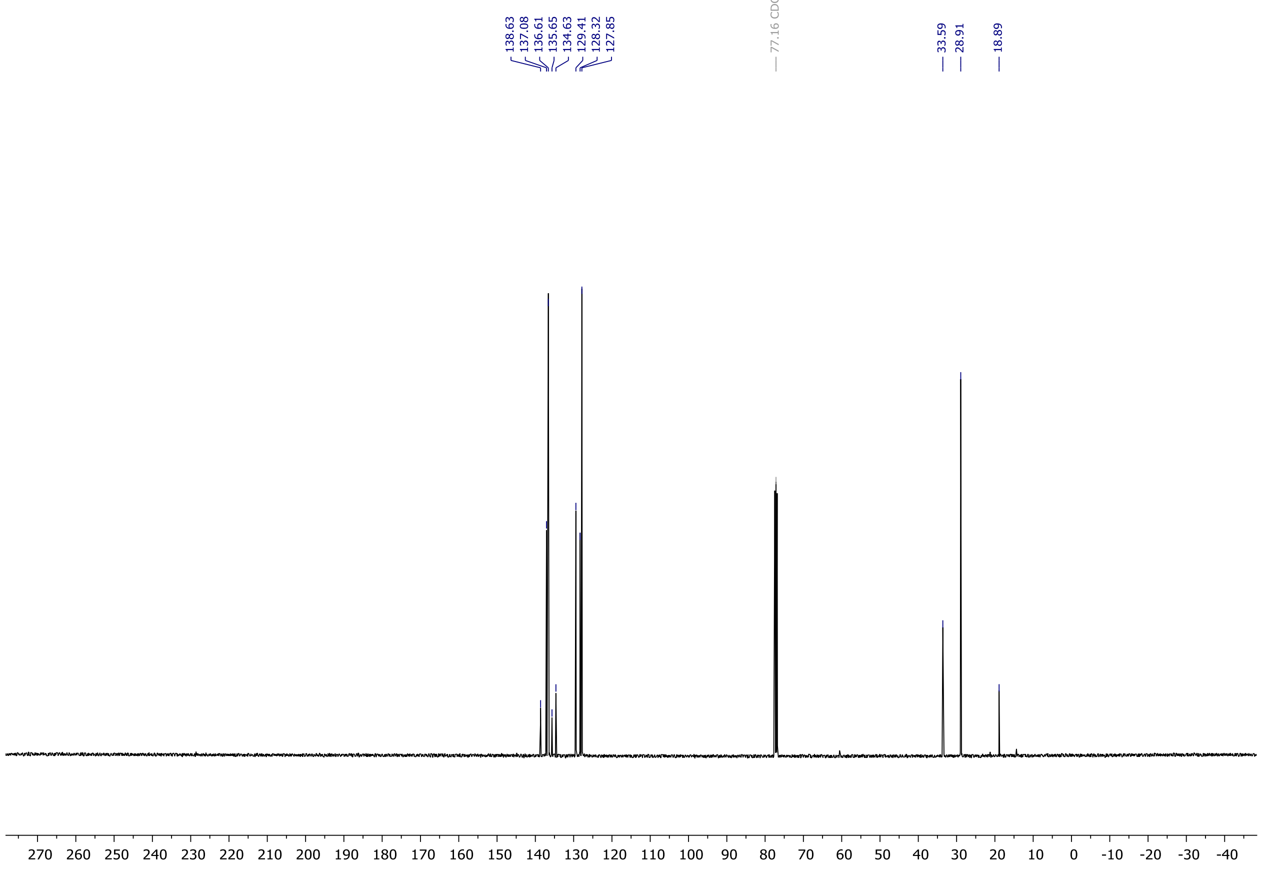
138.63
137.08
136.61
135.65
134.63
129.41
128.32
127.85
77.16 CDCl3
33.59
28.91
18.89
270 260 250 240 230 220 210 200 190 180 170 160 150 140 130 120 110 100 90 80 70 60 50 40 30 20 10 0 -10 -20 -30 -40

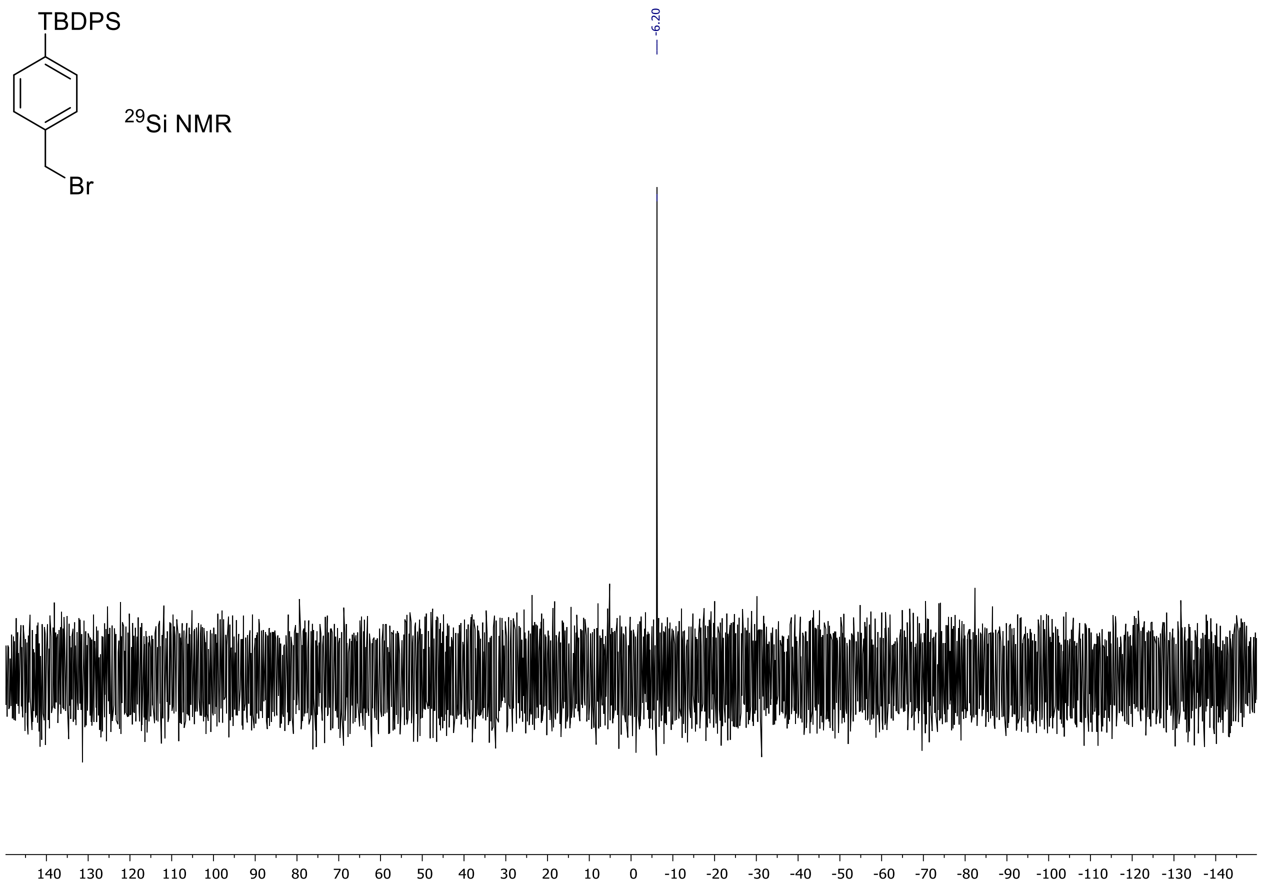
TBDPS
Br
29Si NMR
-6.20
140 130 120 110 100 90 80 70 60 50 40 30 20 10 0 -10 -20 -30 -40 -50 -60 -70 -80 -90 -100 -110 -120 -130 -140

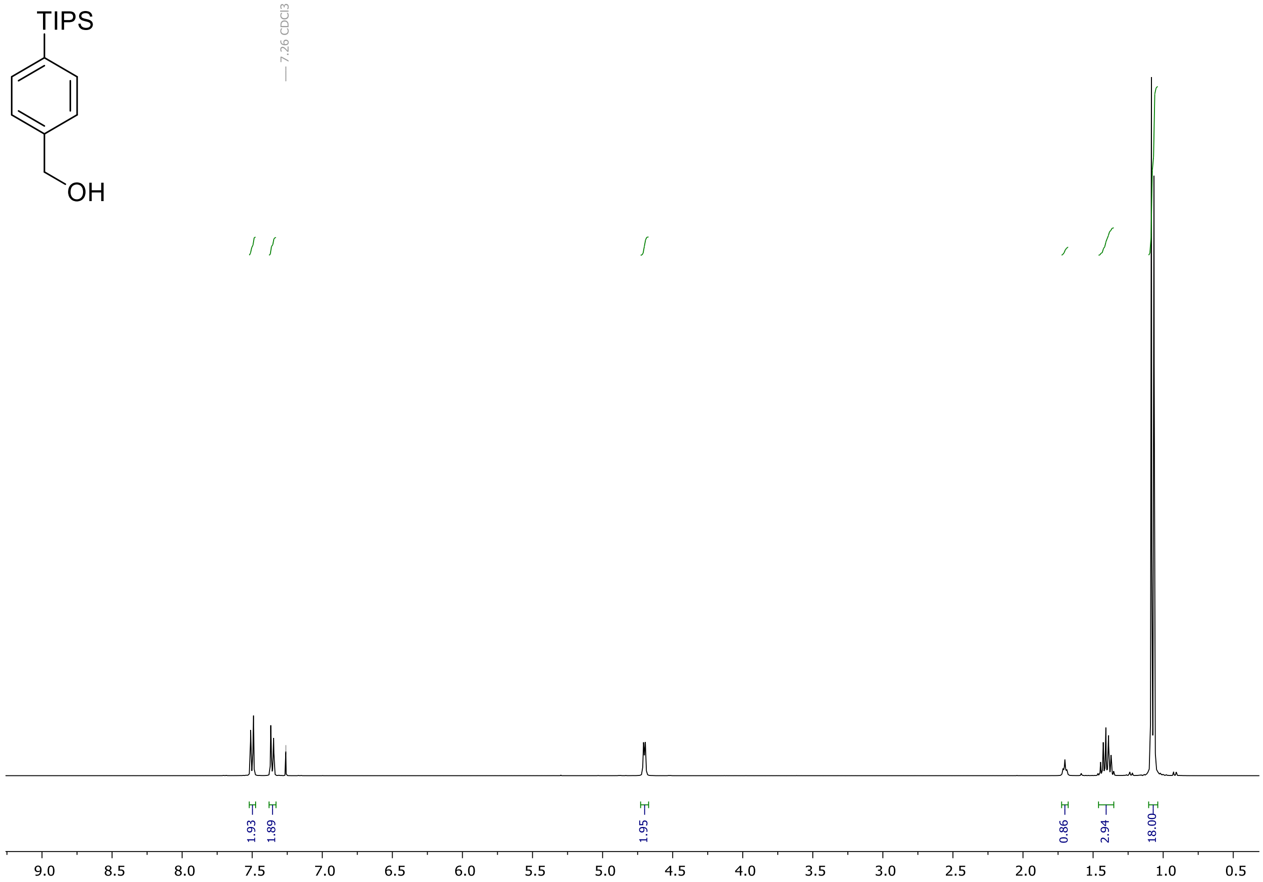
TIPS
OH
7.26 CDCl3
1.93
1.89
1.95
0.86
2.94
18.00
9.0 8.5 8.0 7.5 7.0 6.5 6.0 5.5 5.0 4.5 4.0 3.5 3.0 2.5 2.0 1.5 1.0 0.5

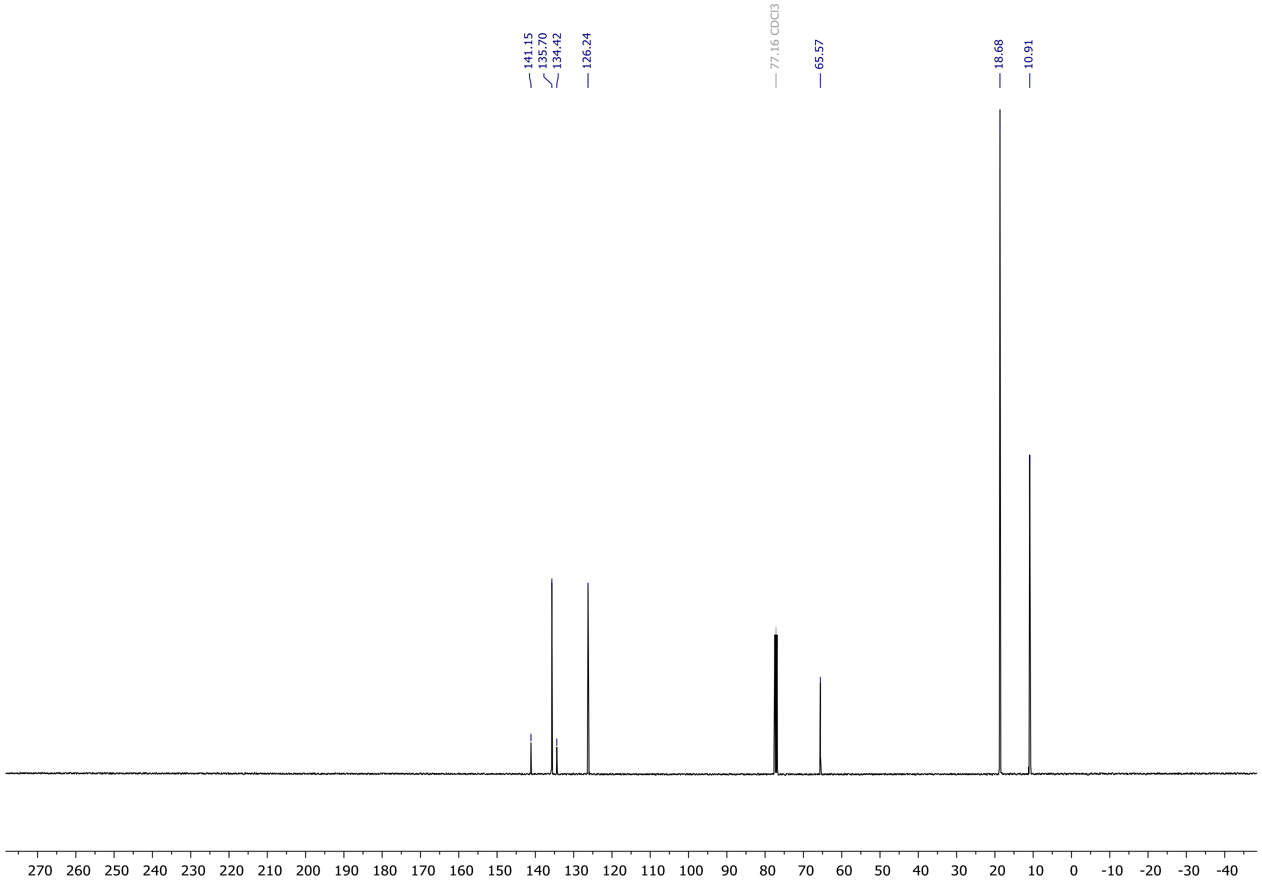
141.15
135.70
134.42
126.24
77.16 CDCl3
65.57
18.68
10.91
270 260 250 240 230 220 210 200 190 180 170 160 150 140 130 120 110 100 90 80 70 60 50 40 30 20 10 0 -10 -20 -30 -40

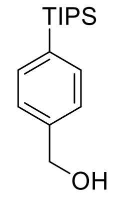

^{29}Si NMR

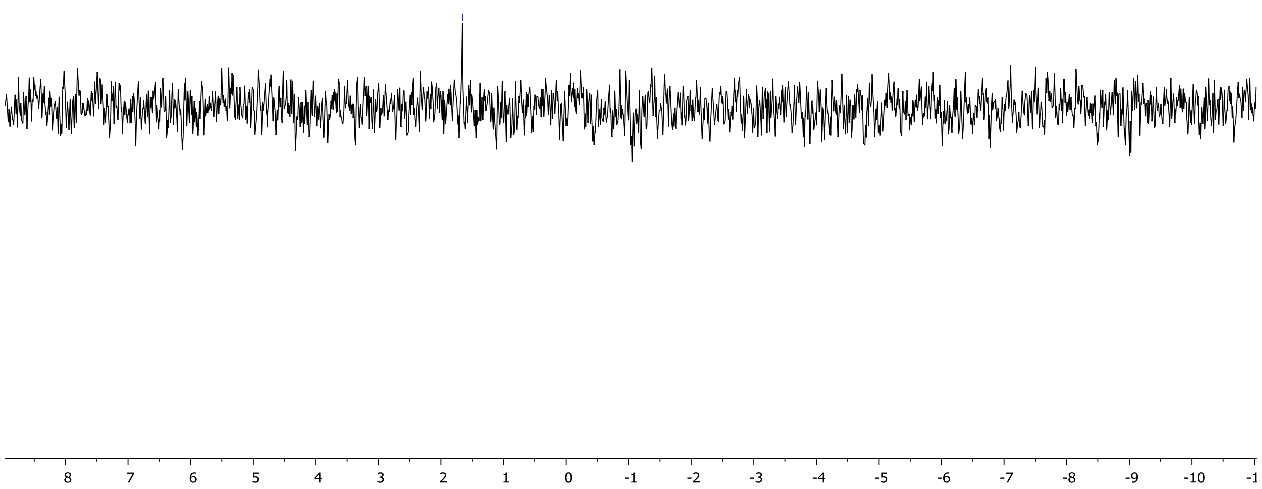

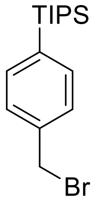
TIPS
Br

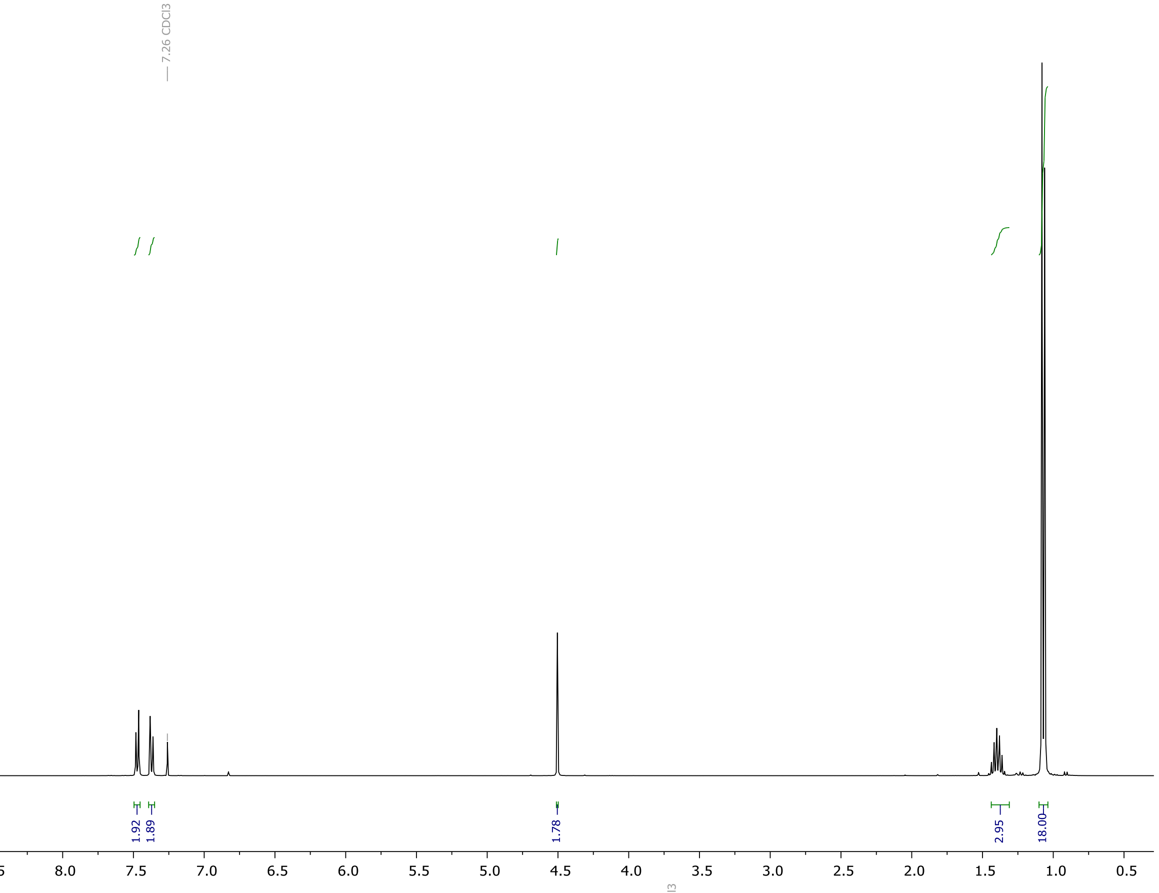
7.26 CDCl3
1.92
1.89
1.78
2.95
18.00
9.0
8.5
8.0
7.5
7.0
6.5
6.0
5.5
5.0
4.5
4.0
3.5
3.0
2.5
2.0
1.5
1.0
0.5

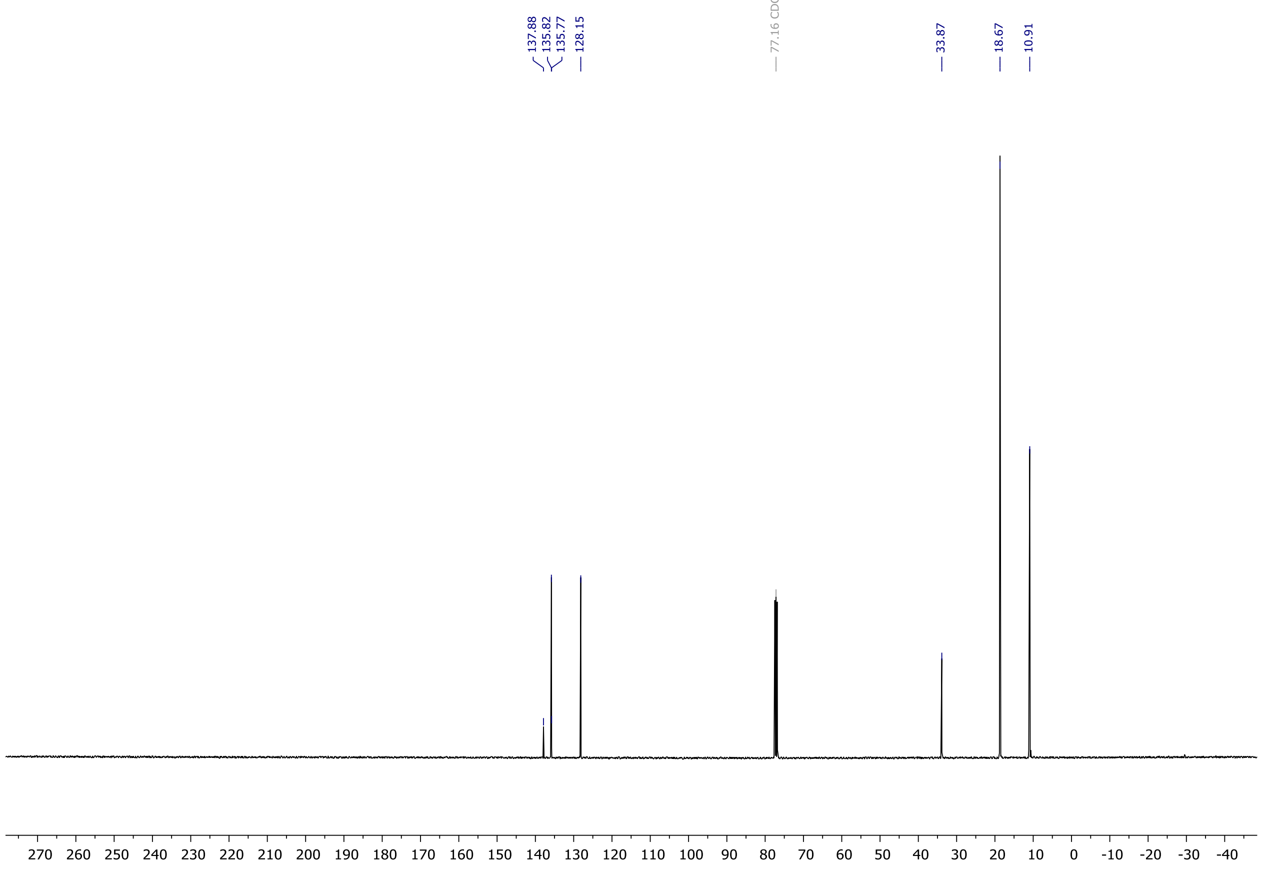
137.88
135.82
135.77
128.15
77.16 CDCl3
33.87
18.67
10.91
270
260
250
240
230
220
210
200
190
180
170
160
150
140
130
120
110
100
90
80
70
60
50
40
30
20
10
0
-10
-20
-30
-40

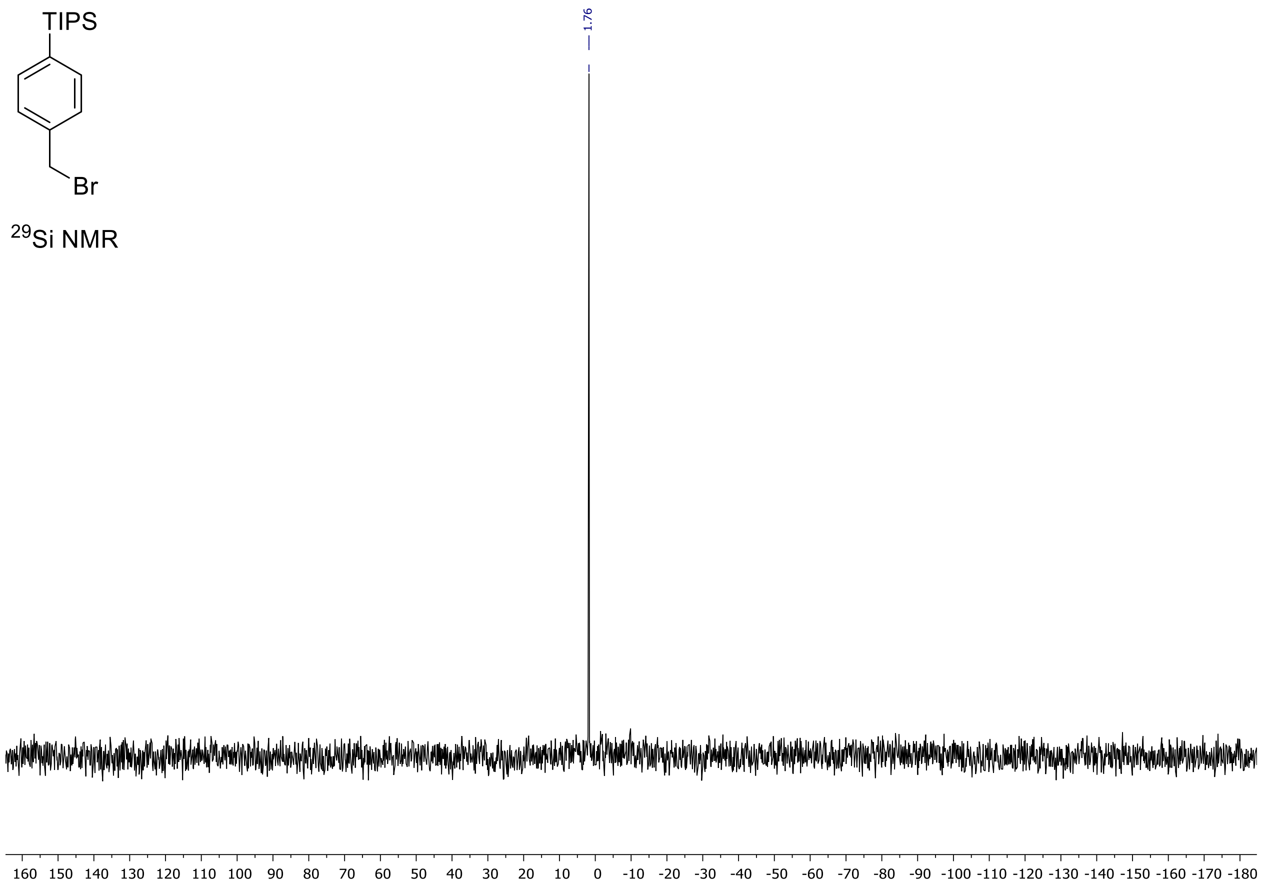
TIPS
Br
^{29}Si NMR
1.76
160 150 140 130 120 110 100 90 80 70 60 50 40 30 20 10 0 -10 -20 -30 -40 -50 -60 -70 -80 -90 -100 -110 -120 -130 -140 -150 -160 -170 -180

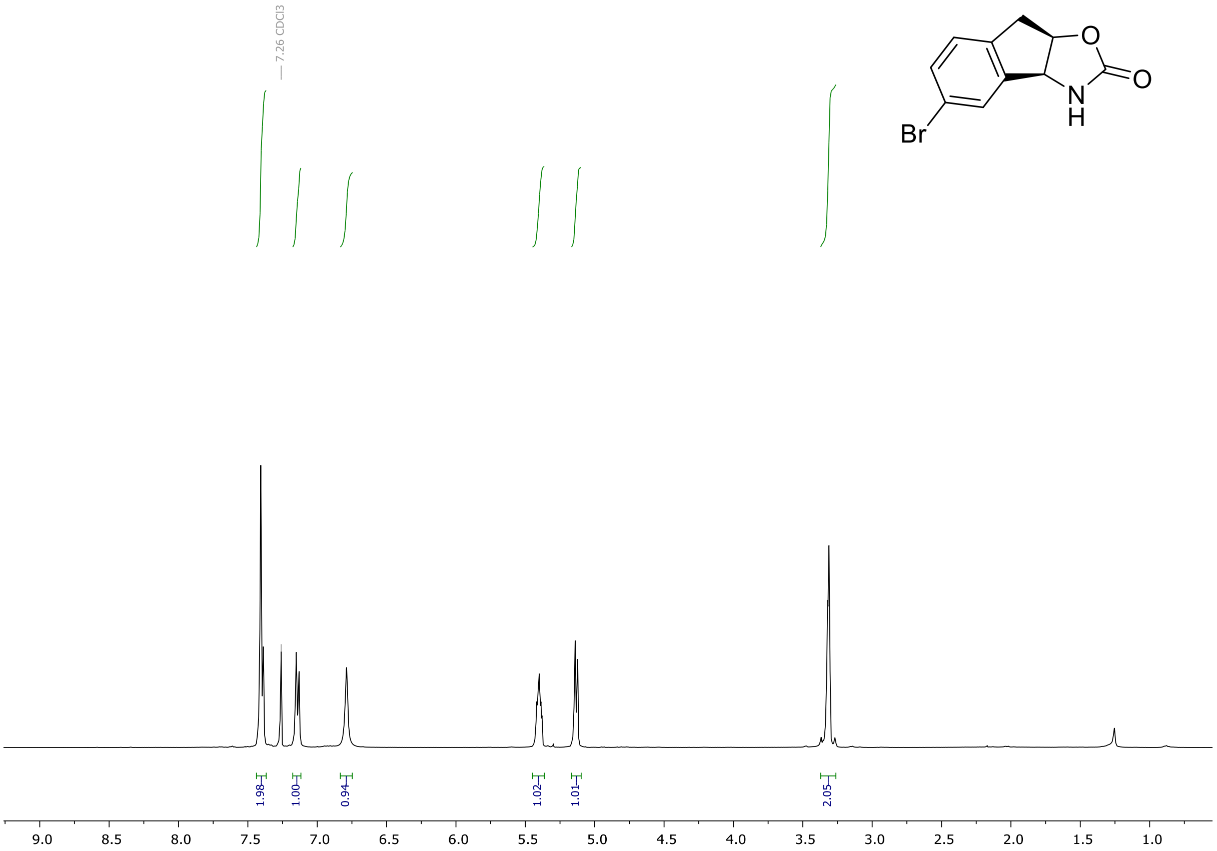
Br
O
O
N
H
7.26 CDCl3
1.98
1.00
0.94
1.02
1.01
2.05
9.0 8.5 8.0 7.5 7.0 6.5 6.0 5.5 5.0 4.5 4.0 3.5 3.0 2.5 2.0 1.5 1.0

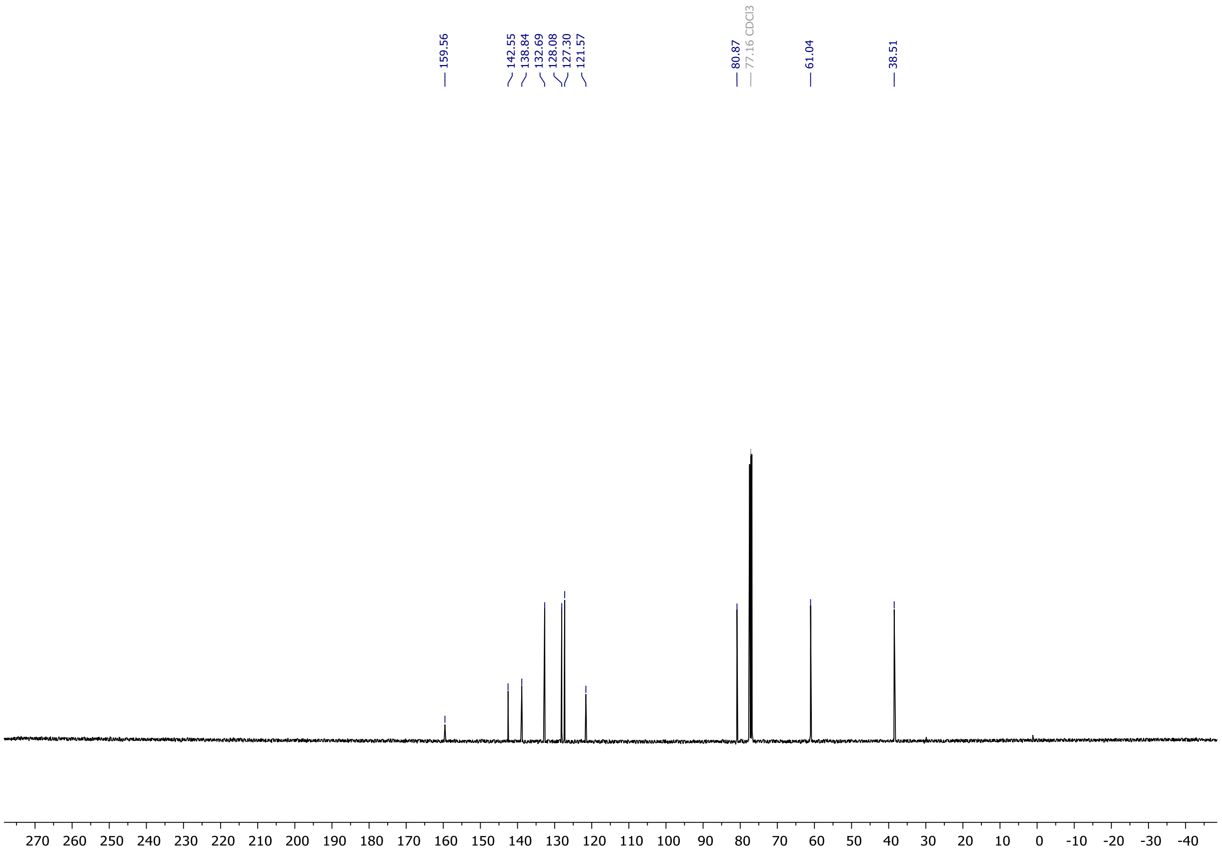
159.56
142.55
138.84
132.69
128.08
127.30
121.57
80.87
77.16 CDCl3
61.04
38.51
270 260 250 240 230 220 210 200 190 180 170 160 150 140 130 120 110 100 90 80 70 60 50 40 30 20 10 0 -10 -20 -30 -40

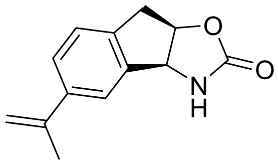

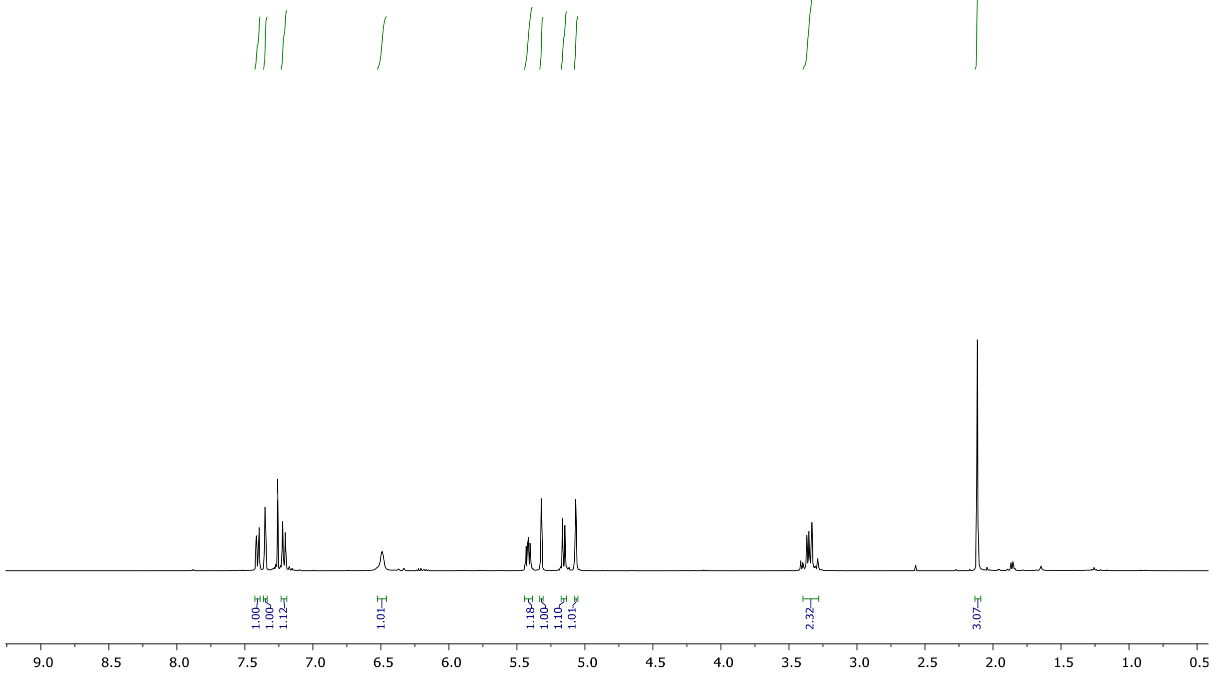
7.26 CDCl3
1.00
1.00
1.12
1.01
1.18
1.00
1.10
1.01
2.32
3.07
9.0 8.5 8.0 7.5 7.0 6.5 6.0 5.5 5.0 4.5 4.0 3.5 3.0 2.5 2.0 1.5 1.0 0.5

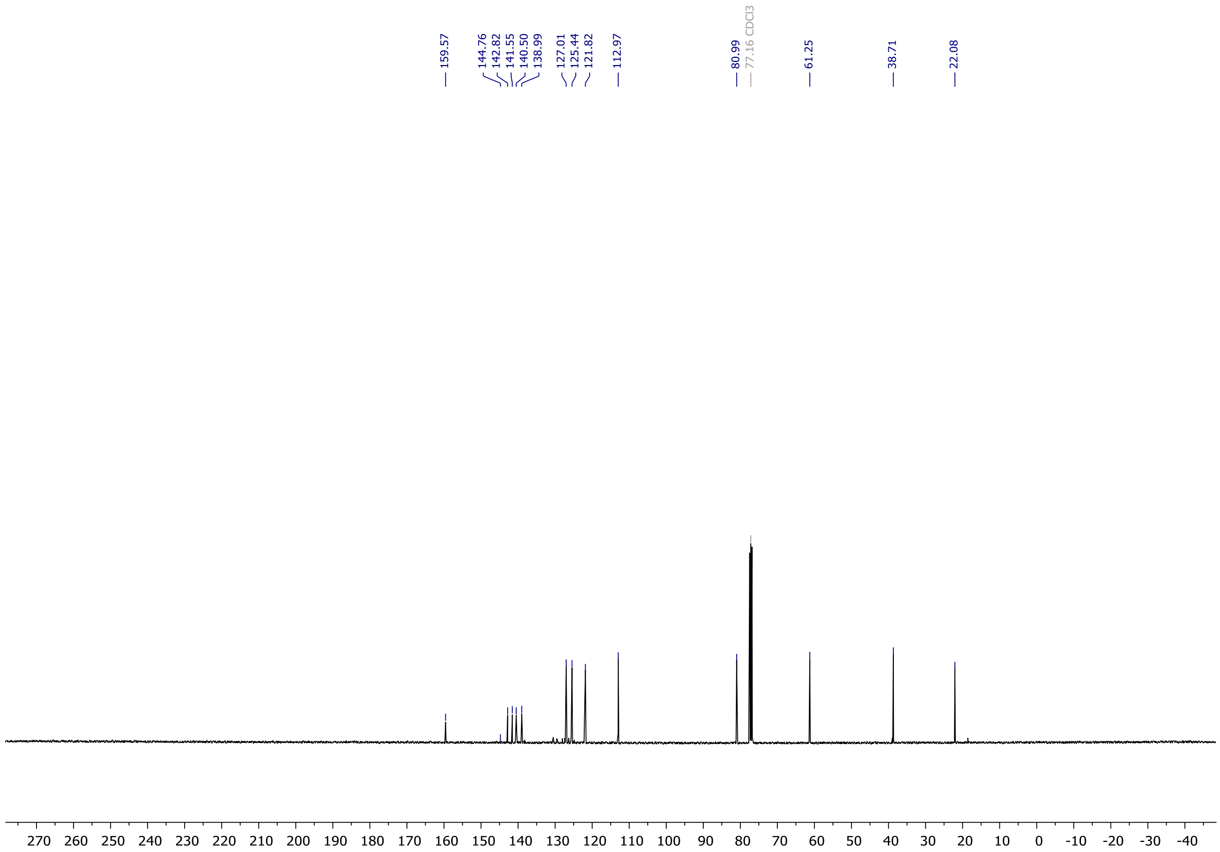
159.57
144.76
142.82
141.55
140.50
138.99
127.01
125.44
121.82
112.97
80.99
77.16 CDCl3
61.25
38.71
22.08
270 260 250 240 230 220 210 200 190 180 170 160 150 140 130 120 110 100 90 80 70 60 50 40 30 20 10 0 -10 -20 -30 -40

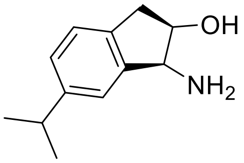

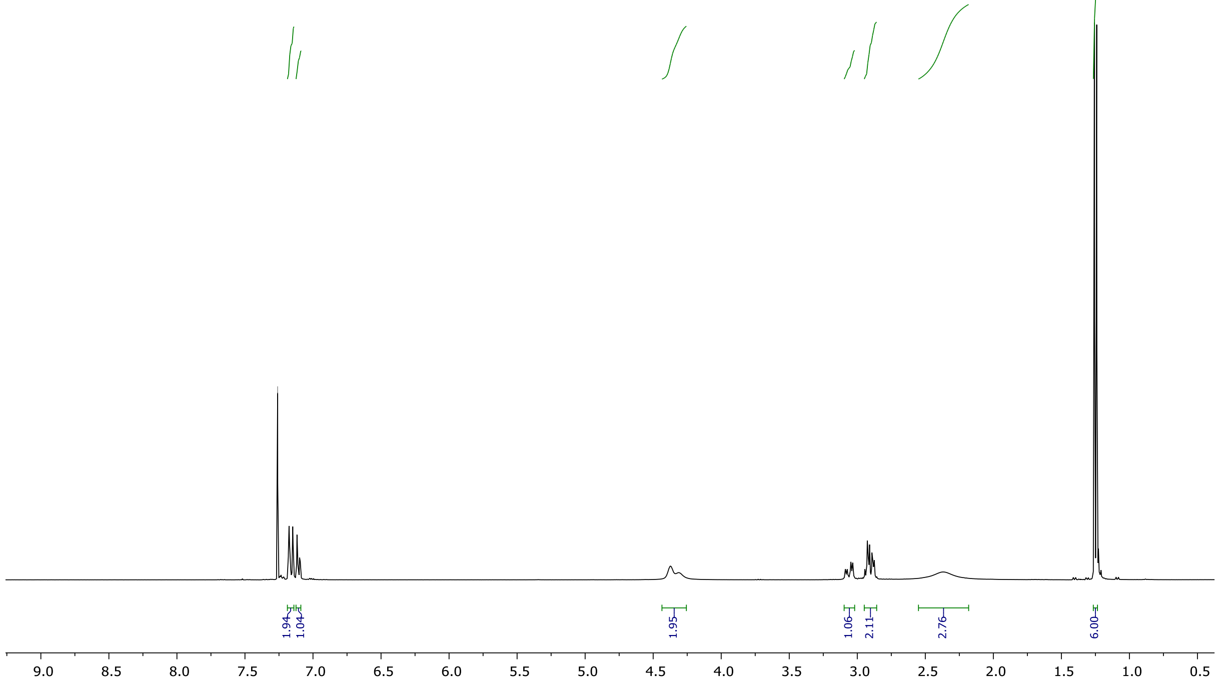

7.26 CDCl3
1.94
1.04
1.95
1.06
2.11
2.76
6.00
9.0
8.5
8.0
7.5
7.0
6.5
6.0
5.5
5.0
4.5
4.0
3.5
3.0
2.5
2.0
1.5
1.0
0.5

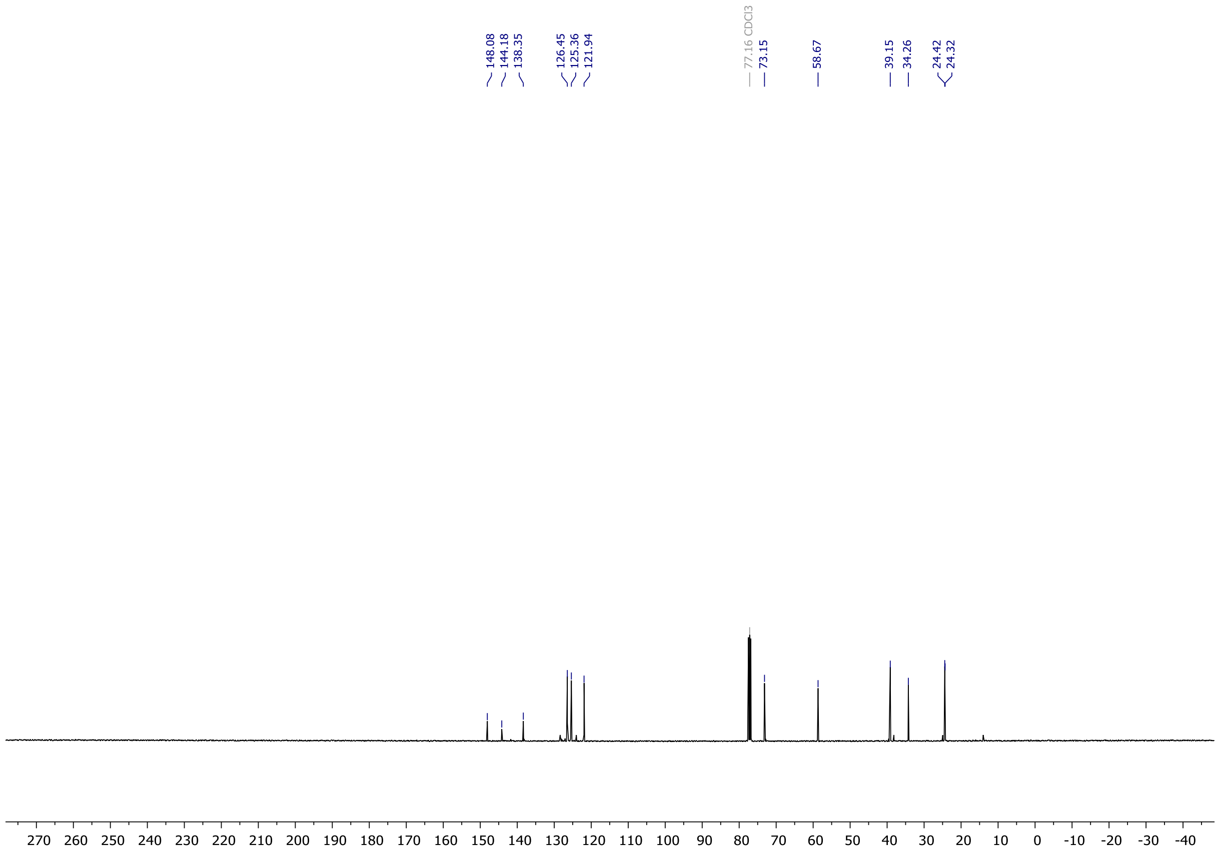

148.08
144.18
138.35
126.45
125.36
121.94
77.16 CDCl3
73.15
58.67
39.15
34.26
24.42
24.32
270 260 250 240 230 220 210 200 190 180 170 160 150 140 130 120 110 100 90 80 70 60 50 40 30 20 10 0 -10 -20 -30 -40

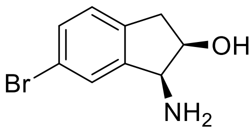

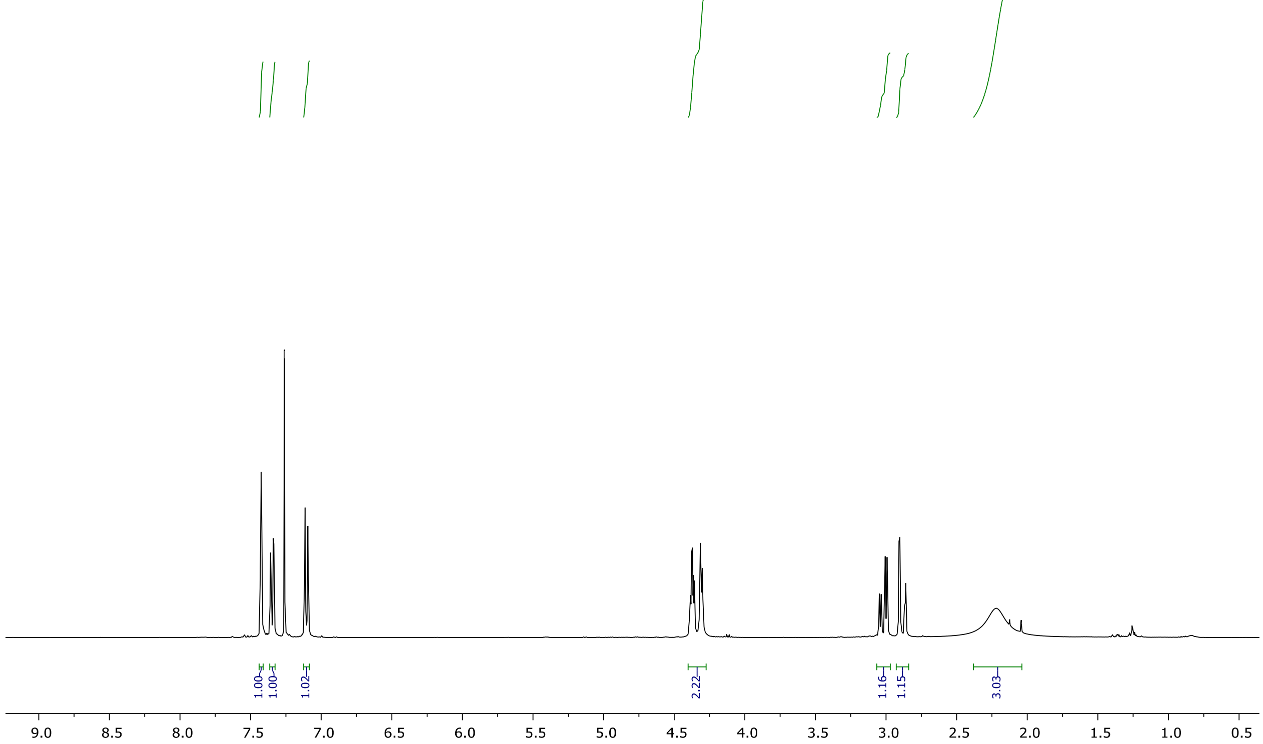

7.26 CDCl3
1.00
1.00
1.02
2.22
1.16
1.15
3.03
9.0
8.5
8.0
7.5
7.0
6.5
6.0
5.5
5.0
4.5
4.0
3.5
3.0
2.5
2.0
1.5
1.0
0.5

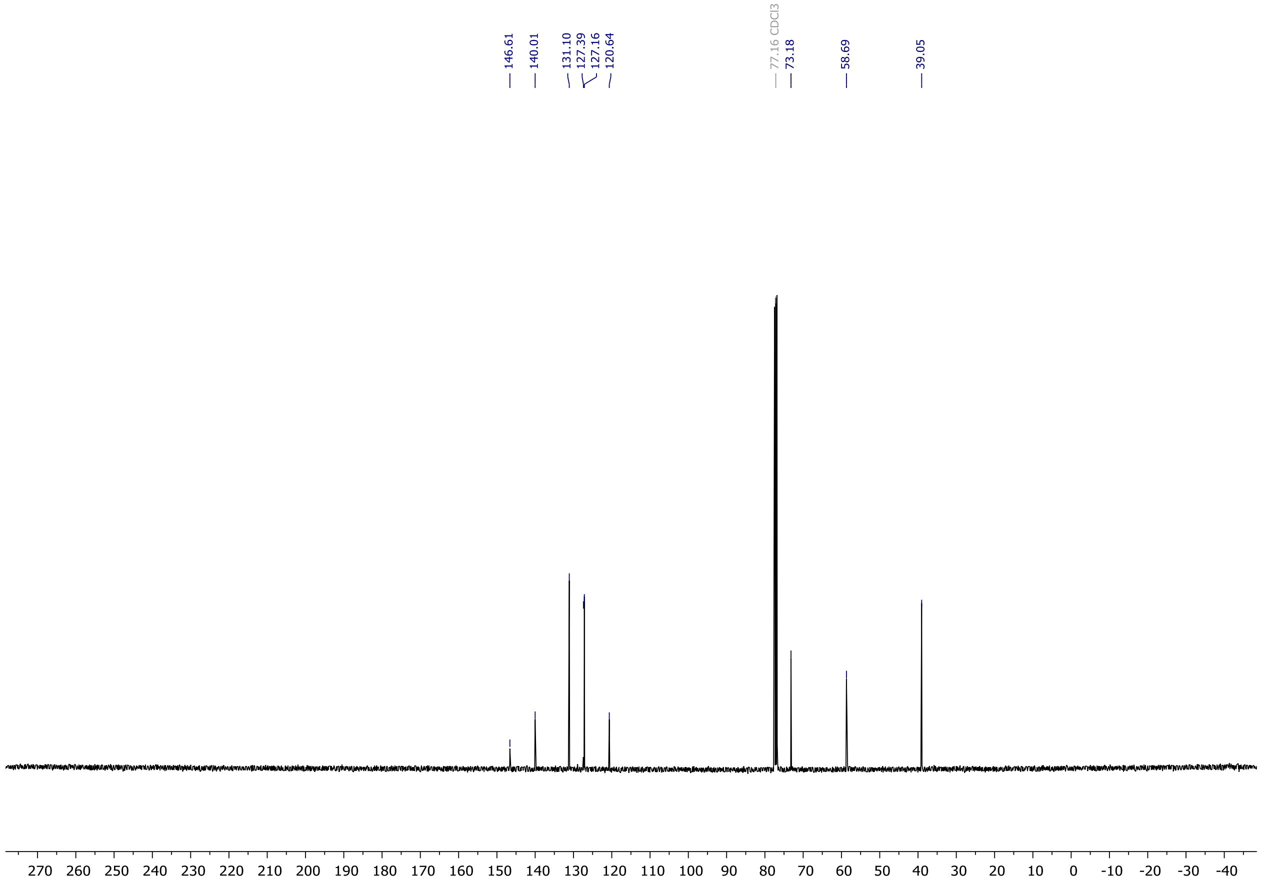

146.61
140.01
131.10
127.39
127.16
120.64
77.16 CDCl3
73.18
58.69
39.05
270 260 250 240 230 220 210 200 190 180 170 160 150 140 130 120 110 100 90 80 70 60 50 40 30 20 10 0 -10 -20 -30 -40

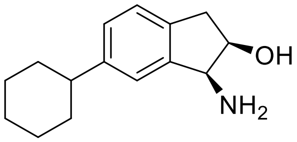

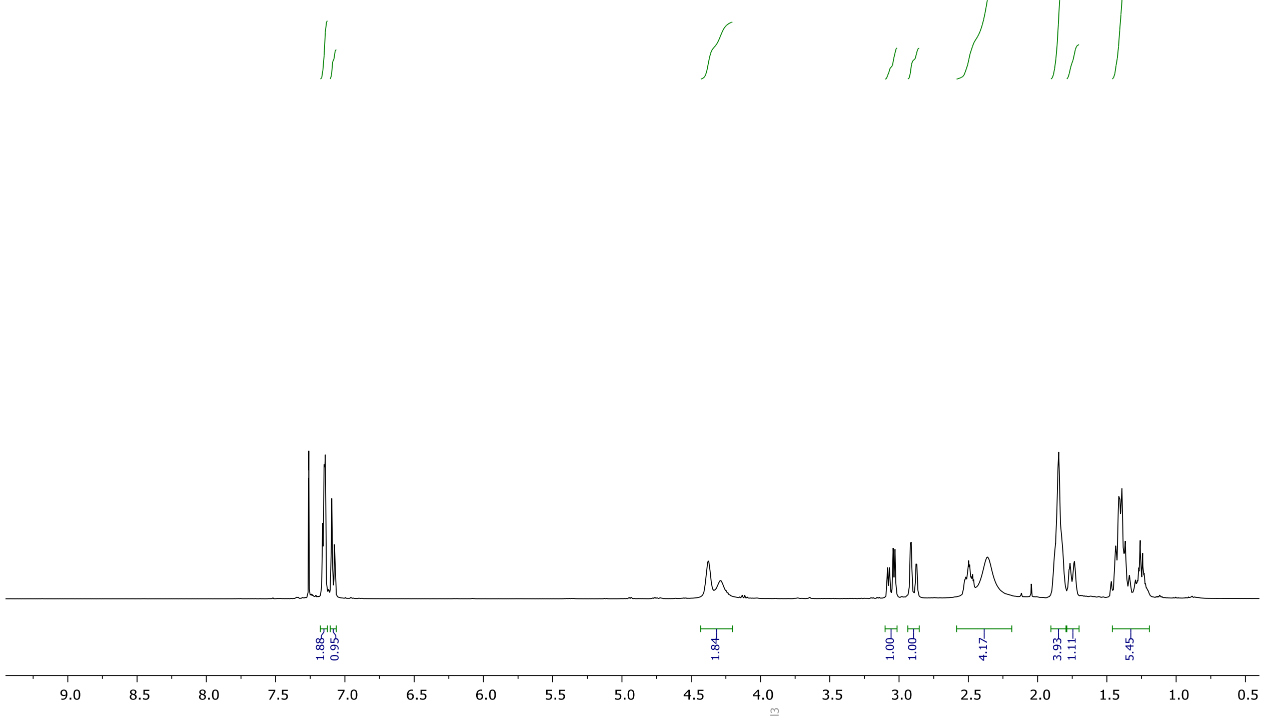
7.26 CDCl3
1.88
0.95
1.84
1.00
1.00
4.17
3.93
1.11
5.45
9.0 8.5 8.0 7.5 7.0 6.5 6.0 5.5 5.0 4.5 4.0 3.5 3.0 2.5 2.0 1.5 1.0 0.5

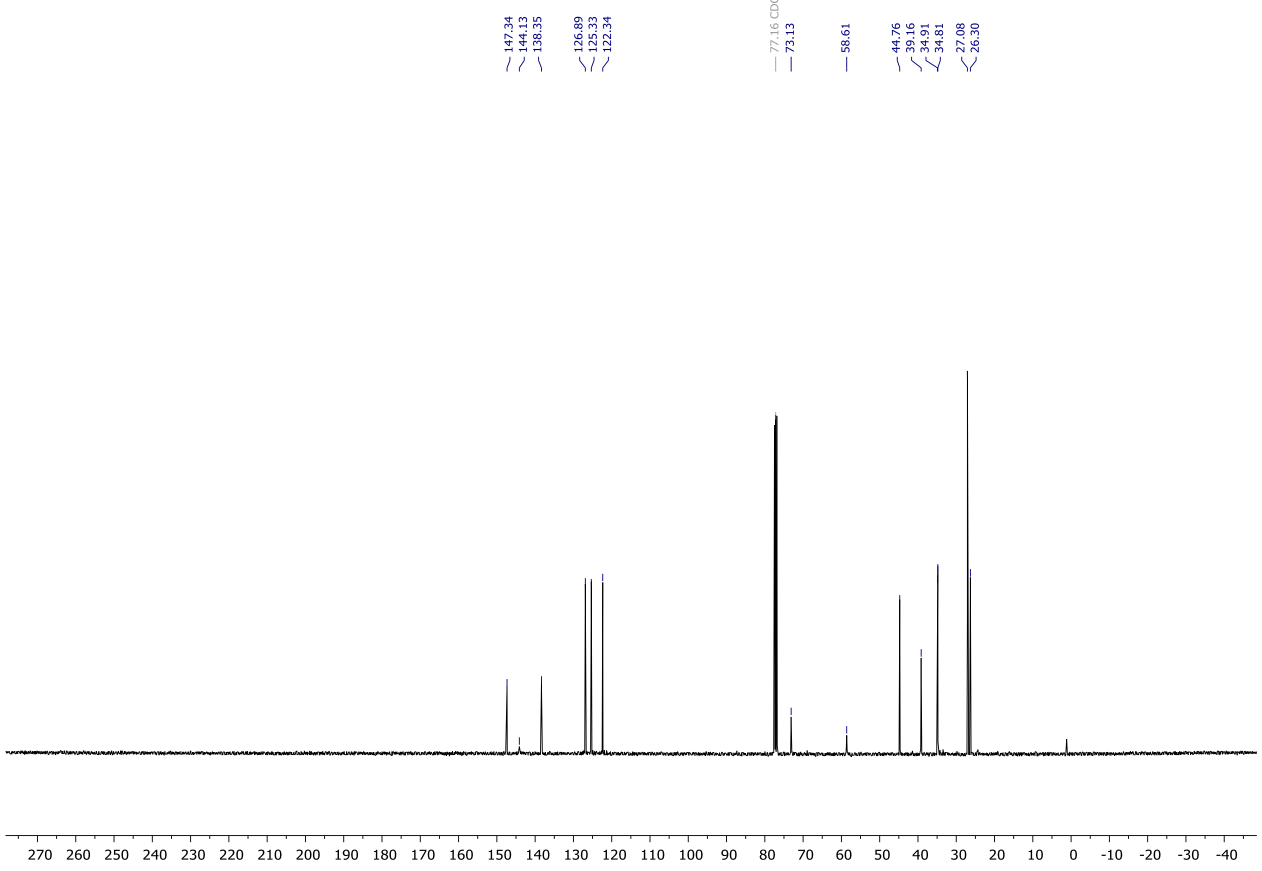
147.34
144.13
138.35
126.89
125.33
122.34
77.16 CDCl3
73.13
58.61
44.76
39.16
34.91
34.81
27.08
26.30
270 260 250 240 230 220 210 200 190 180 170 160 150 140 130 120 110 100 90 80 70 60 50 40 30 20 10 0 -10 -20 -30 -40

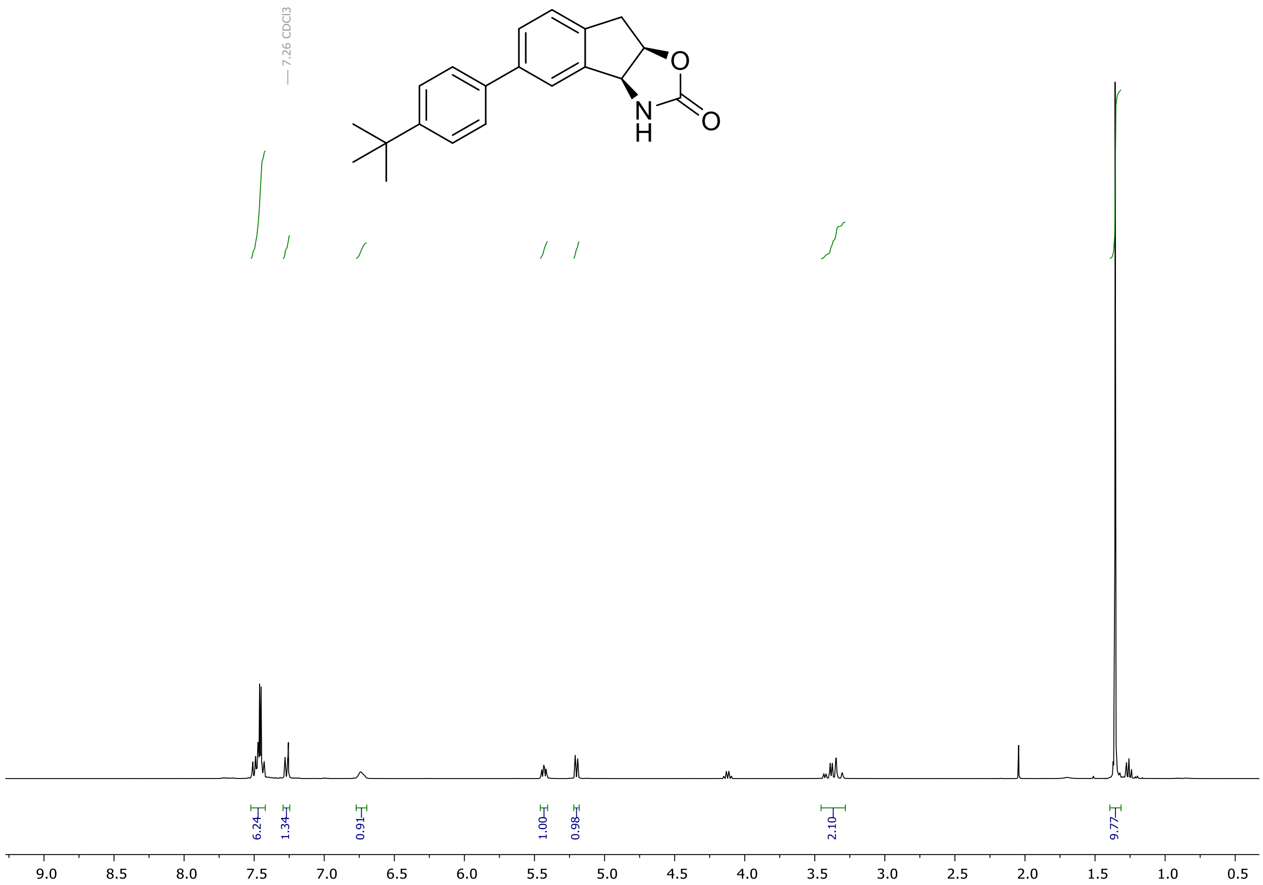

7.26 CDCl3
O
N
H
O
6.24
1.34
0.91
1.00
0.98
2.10
9.77
9.0
8.5
8.0
7.5
7.0
6.5
6.0
5.5
5.0
4.5
4.0
3.5
3.0
2.5
2.0
1.5
1.0
0.5

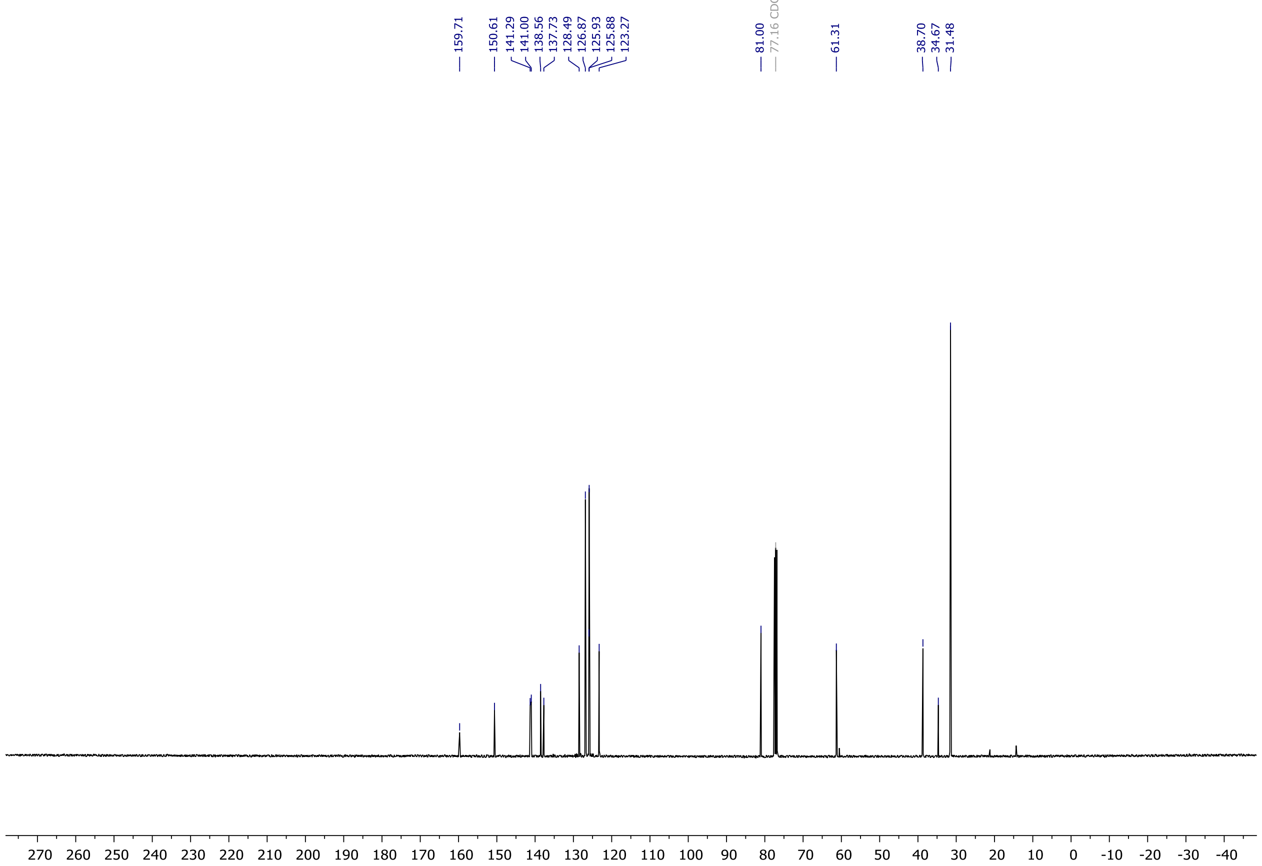

159.71
150.61
141.29
141.00
138.56
137.73
128.49
126.87
125.93
125.88
123.27
81.00
77.16 CDCl3
61.31
38.70
34.67
31.48
270 260 250 240 230 220 210 200 190 180 170 160 150 140 130 120 110 100 90 80 70 60 50 40 30 20 10 0 -10 -20 -30 -40

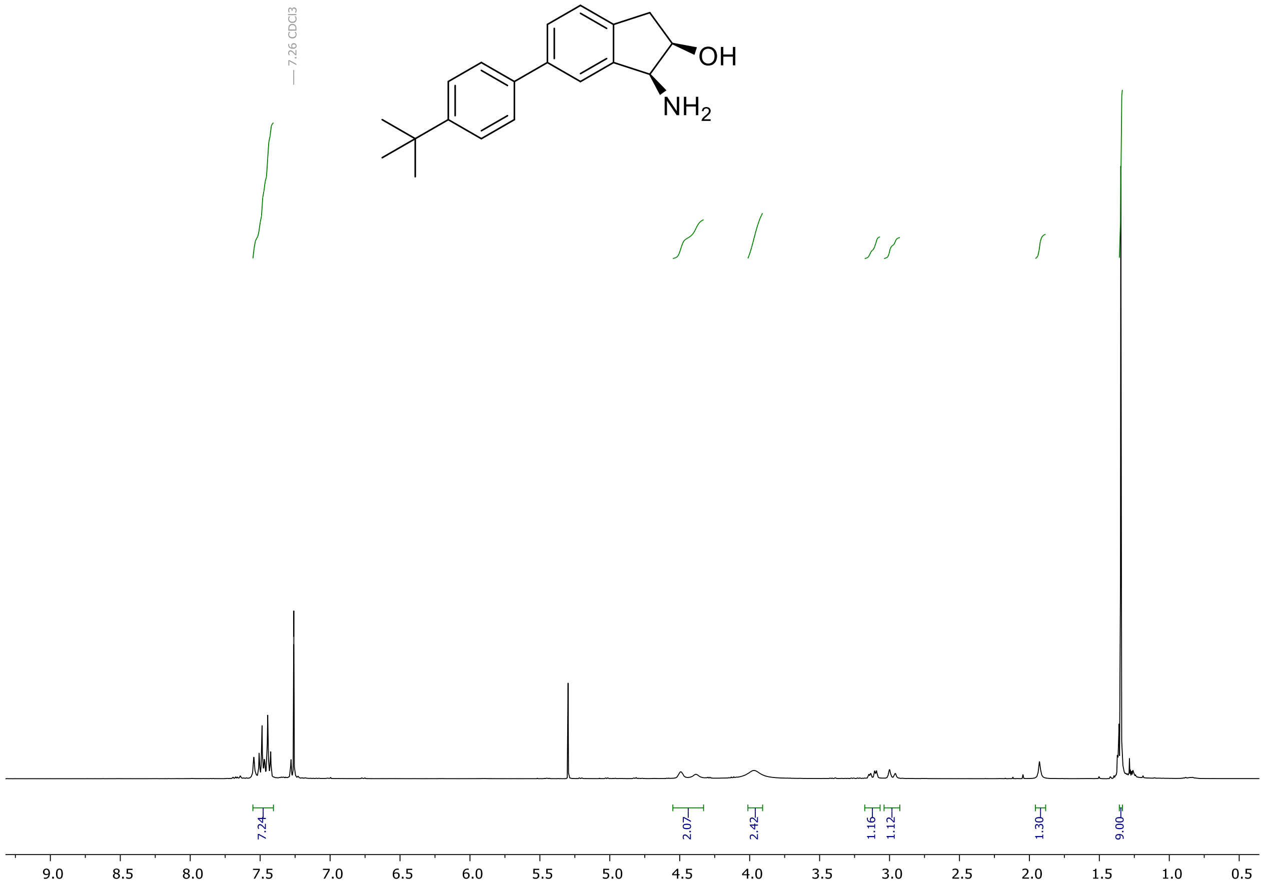
7.26 CDCl3
OH
NH2
7.24
2.07
2.42
1.16
1.12
1.30
9.00
9.0 8.5 8.0 7.5 7.0 6.5 6.0 5.5 5.0 4.5 4.0 3.5 3.0 2.5 2.0 1.5 1.0 0.5

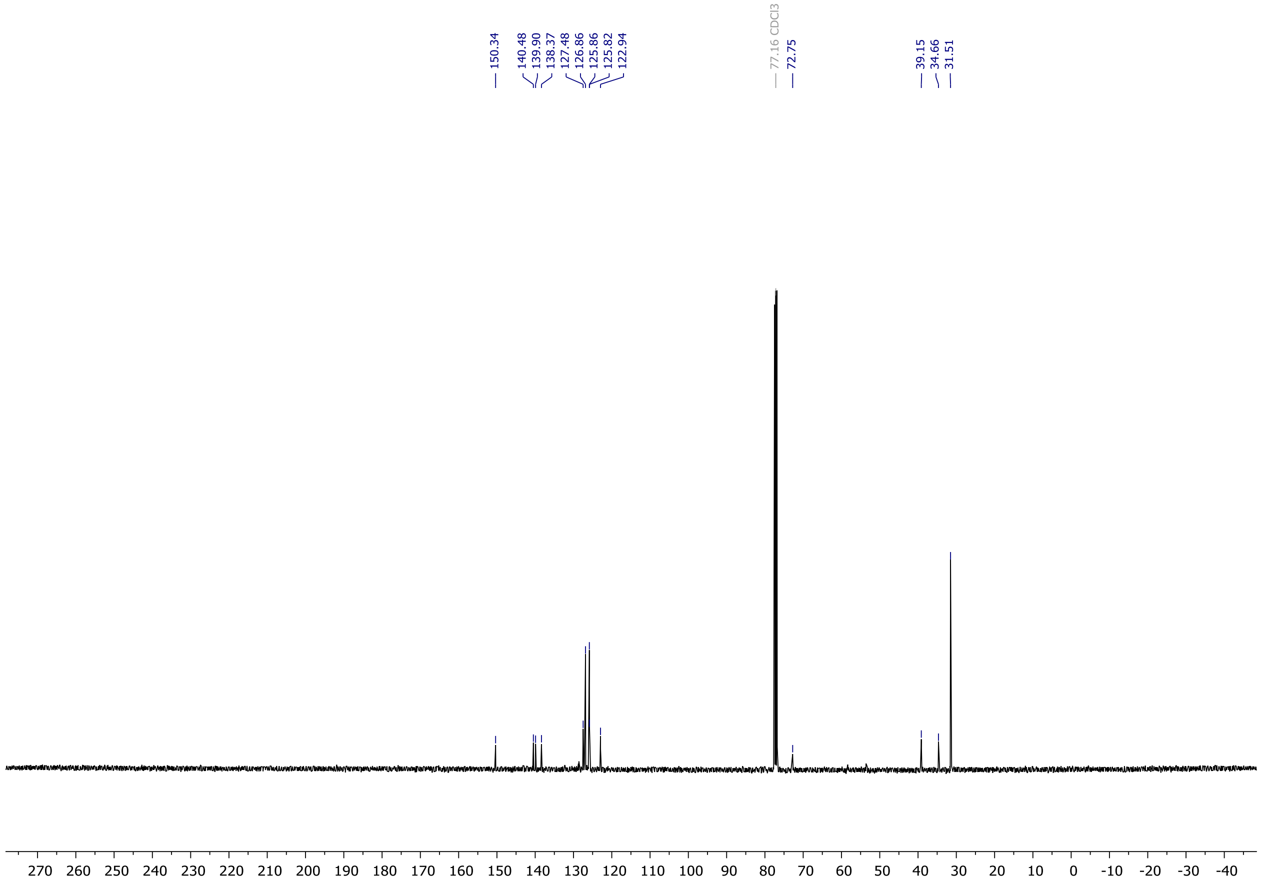
150.34
140.48
139.90
138.37
127.48
126.86
125.86
125.82
122.94
77.16 CDCl3
72.75
39.15
34.66
31.51
270 260 250 240 230 220 210 200 190 180 170 160 150 140 130 120 110 100 90 80 70 60 50 40 30 20 10 0 -10 -20 -30 -40

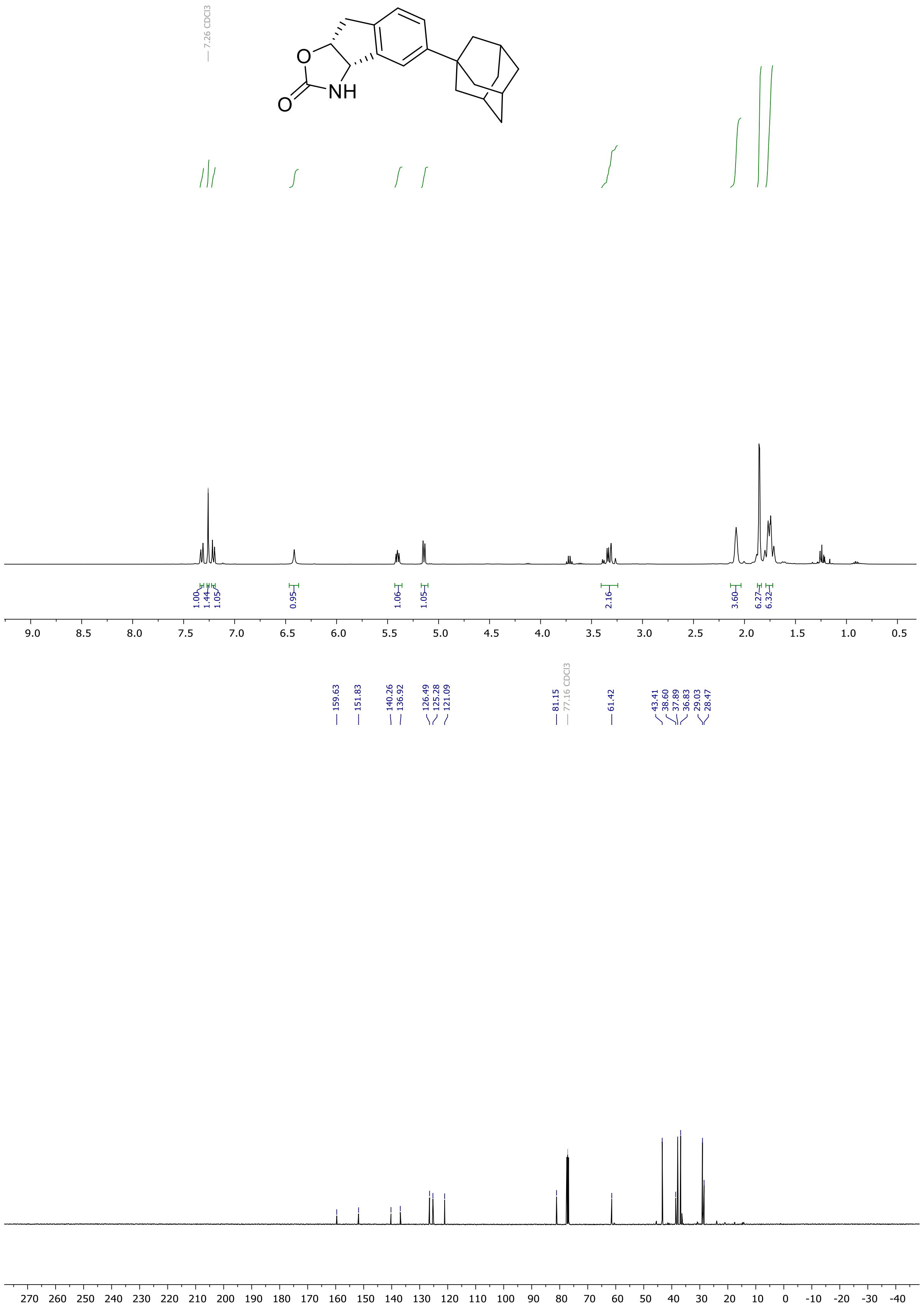
7.26 CDCl3
O
NH
O
1.00
1.44
1.05
0.95
1.06
1.05
2.16
3.60
6.27
6.32
9.0 8.5 8.0 7.5 7.0 6.5 6.0 5.5 5.0 4.5 4.0 3.5 3.0 2.5 2.0 1.5 1.0 0.5
159.63
151.83
140.26
136.92
126.49
125.28
121.09
81.15
77.16 CDCl3
61.42
43.41
38.60
37.89
36.83
29.03
28.47
270 260 250 240 230 220 210 200 190 180 170 160 150 140 130 120 110 100 90 80 70 60 50 40 30 20 10 0 -10 -20 -30 -40

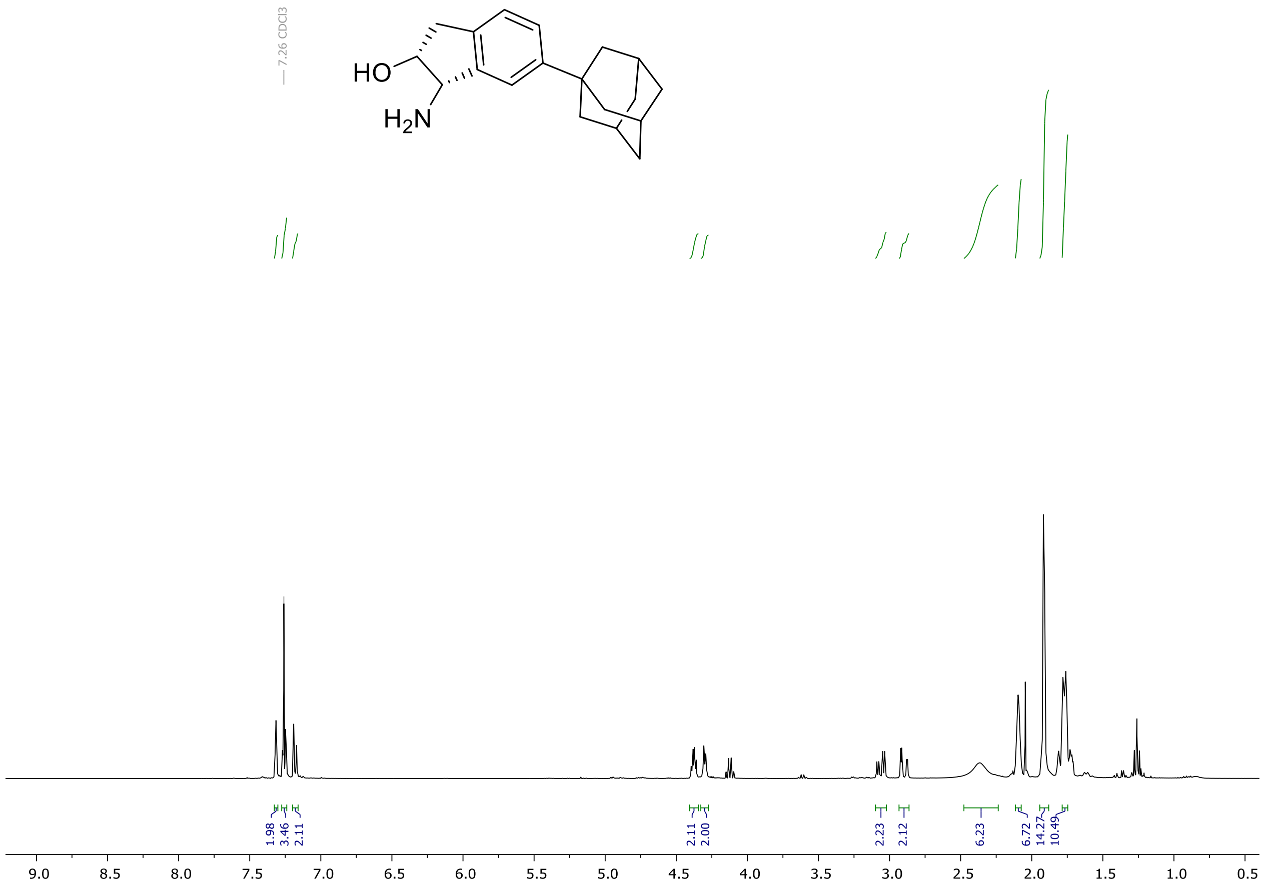
7.26 CDCl3
HO
H2N
1.98
3.46
2.11
2.11
2.00
2.23
2.12
6.23
6.72
14.27
10.49
9.0 8.5 8.0 7.5 7.0 6.5 6.0 5.5 5.0 4.5 4.0 3.5 3.0 2.5 2.0 1.5 1.0 0.5

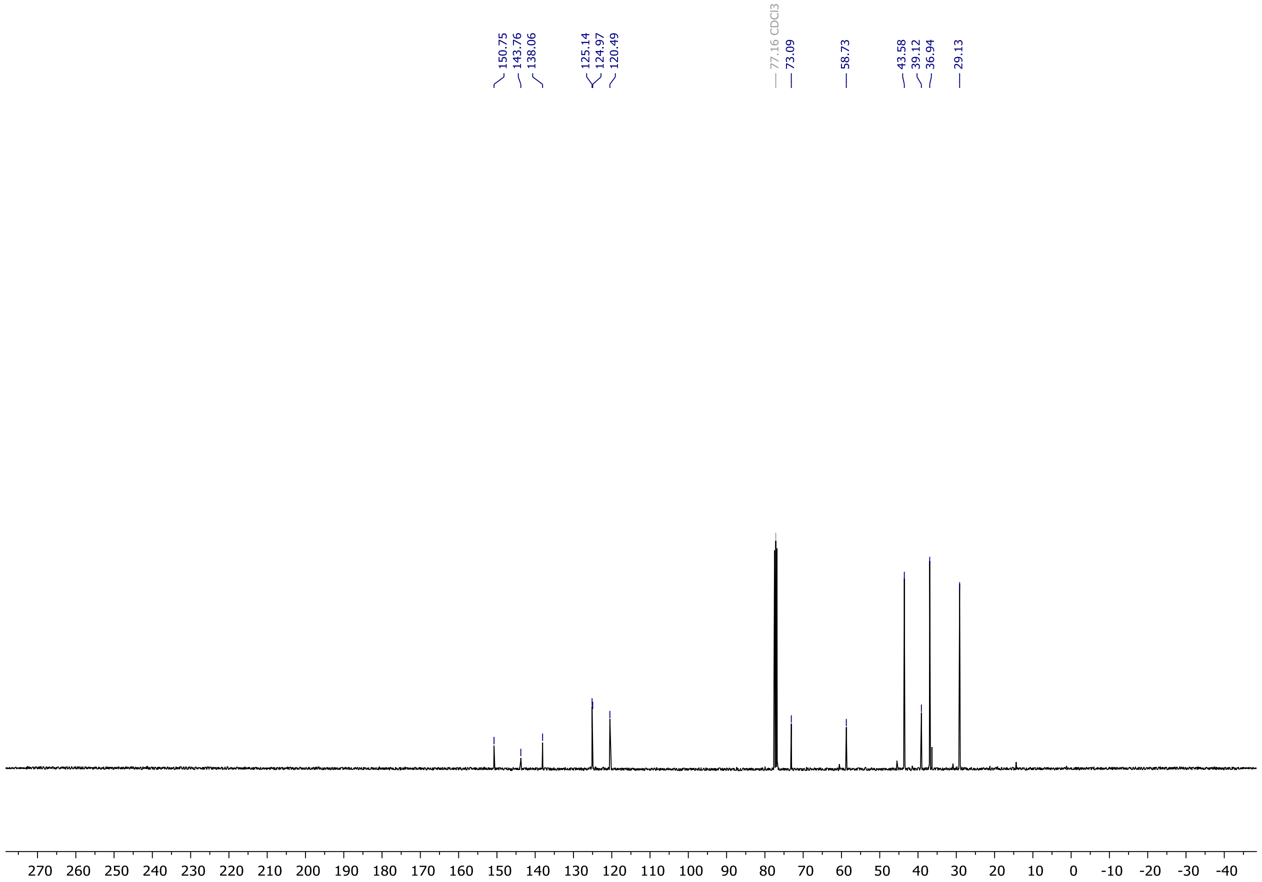
150.75
143.76
138.06
125.14
124.97
120.49
77.16 CDCl3
73.09
58.73
43.58
39.12
36.94
29.13
270 260 250 240 230 220 210 200 190 180 170 160 150 140 130 120 110 100 90 80 70 60 50 40 30 20 10 0 -10 -20 -30 -40

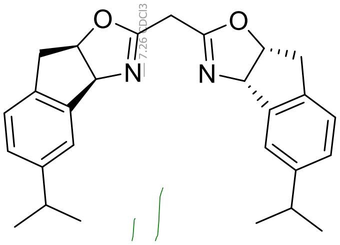

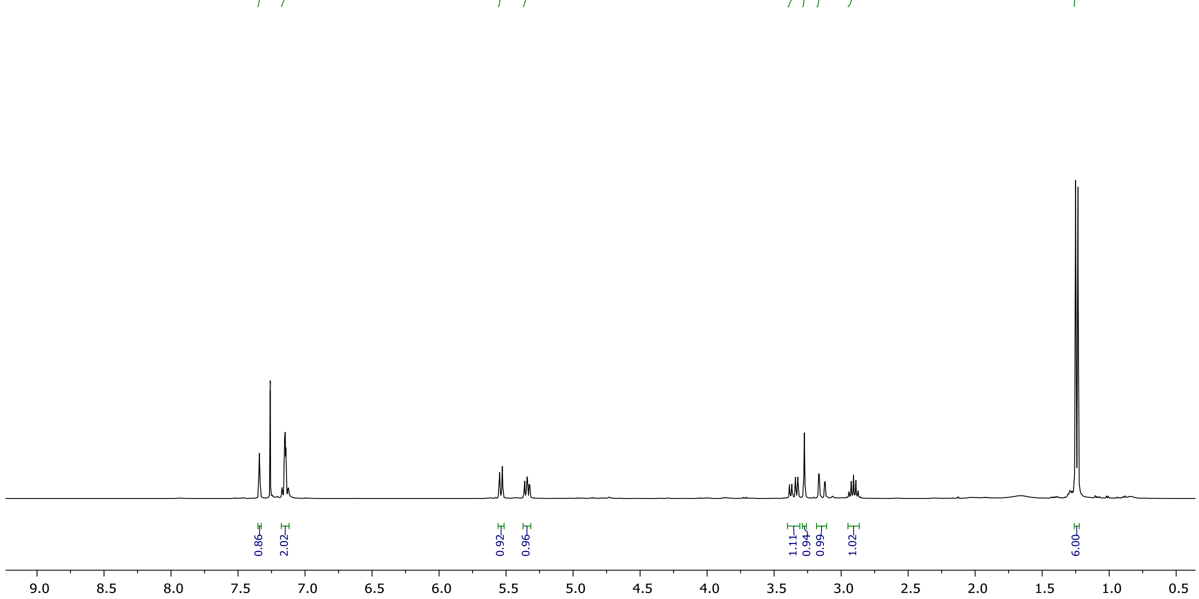
7.26 CDCl3
0.86
2.02
0.92
0.96
1.11
0.94
0.99
1.02
6.00
9.0 8.5 8.0 7.5 7.0 6.5 6.0 5.5 5.0 4.5 4.0 3.5 3.0 2.5 2.0 1.5 1.0 0.5

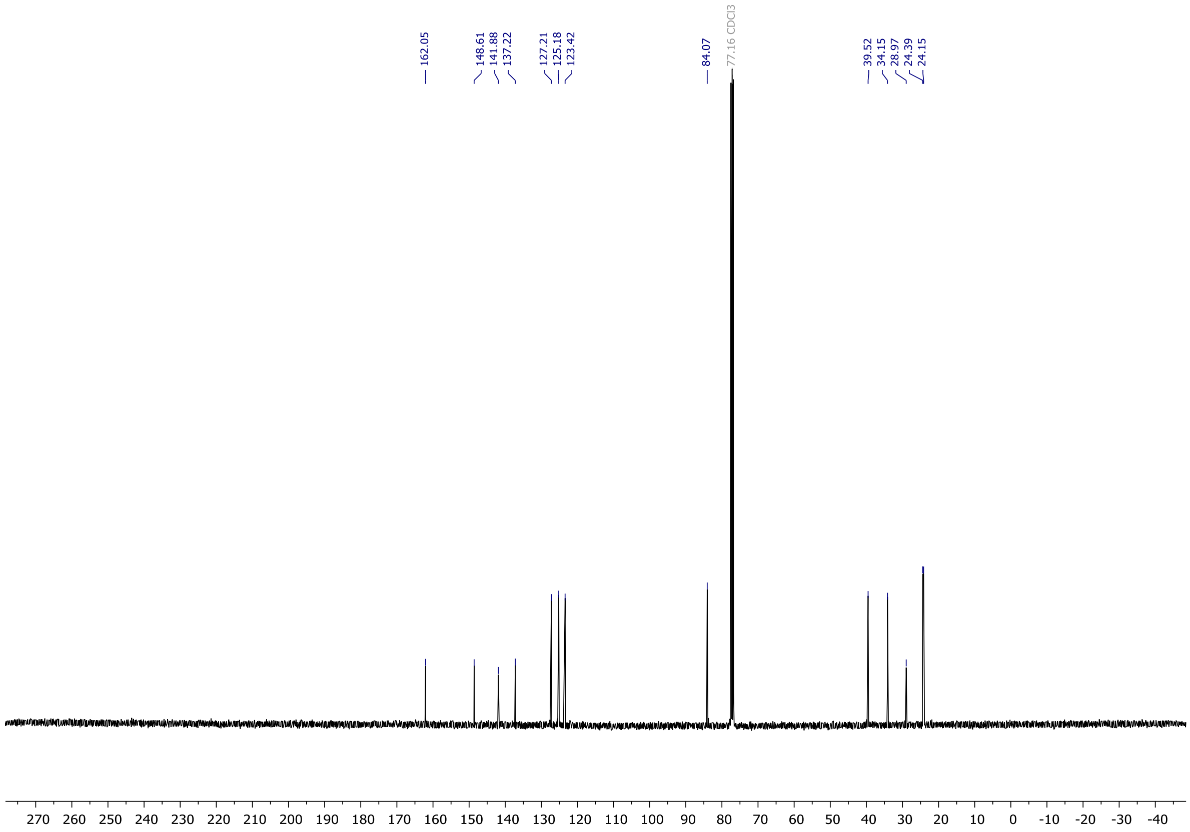
162.05
148.61
141.88
137.22
127.21
125.18
123.42
84.07
77.16 CDCl3
39.52
34.15
28.97
24.39
24.15
270 260 250 240 230 220 210 200 190 180 170 160 150 140 130 120 110 100 90 80 70 60 50 40 30 20 10 0 -10 -20 -30 -40

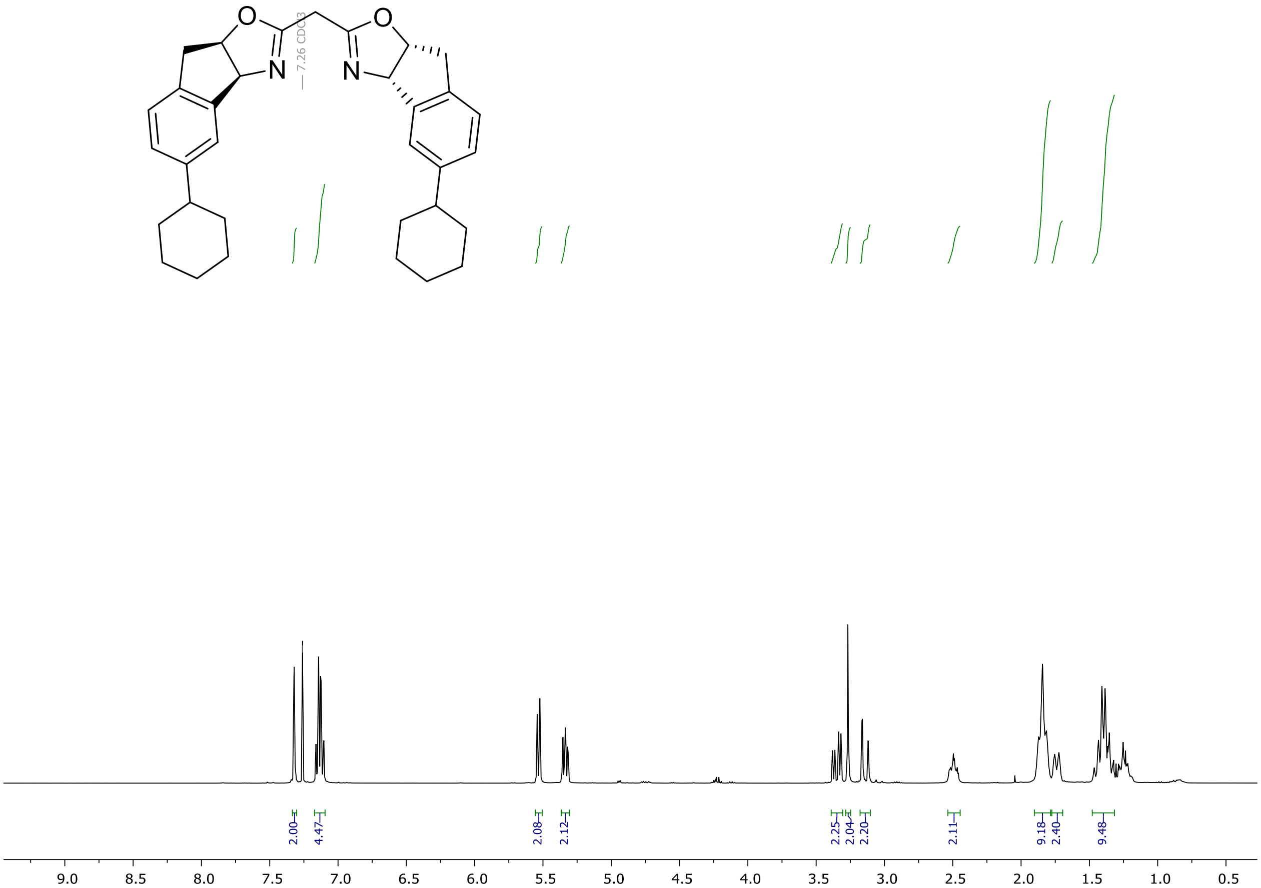
O
N
O
N
7.26 CDCl3
2.00
4.47
2.08
2.12
2.25
2.04
2.20
2.11
9.18
2.40
9.48
9.0 8.5 8.0 7.5 7.0 6.5 6.0 5.5 5.0 4.5 4.0 3.5 3.0 2.5 2.0 1.5 1.0 0.5

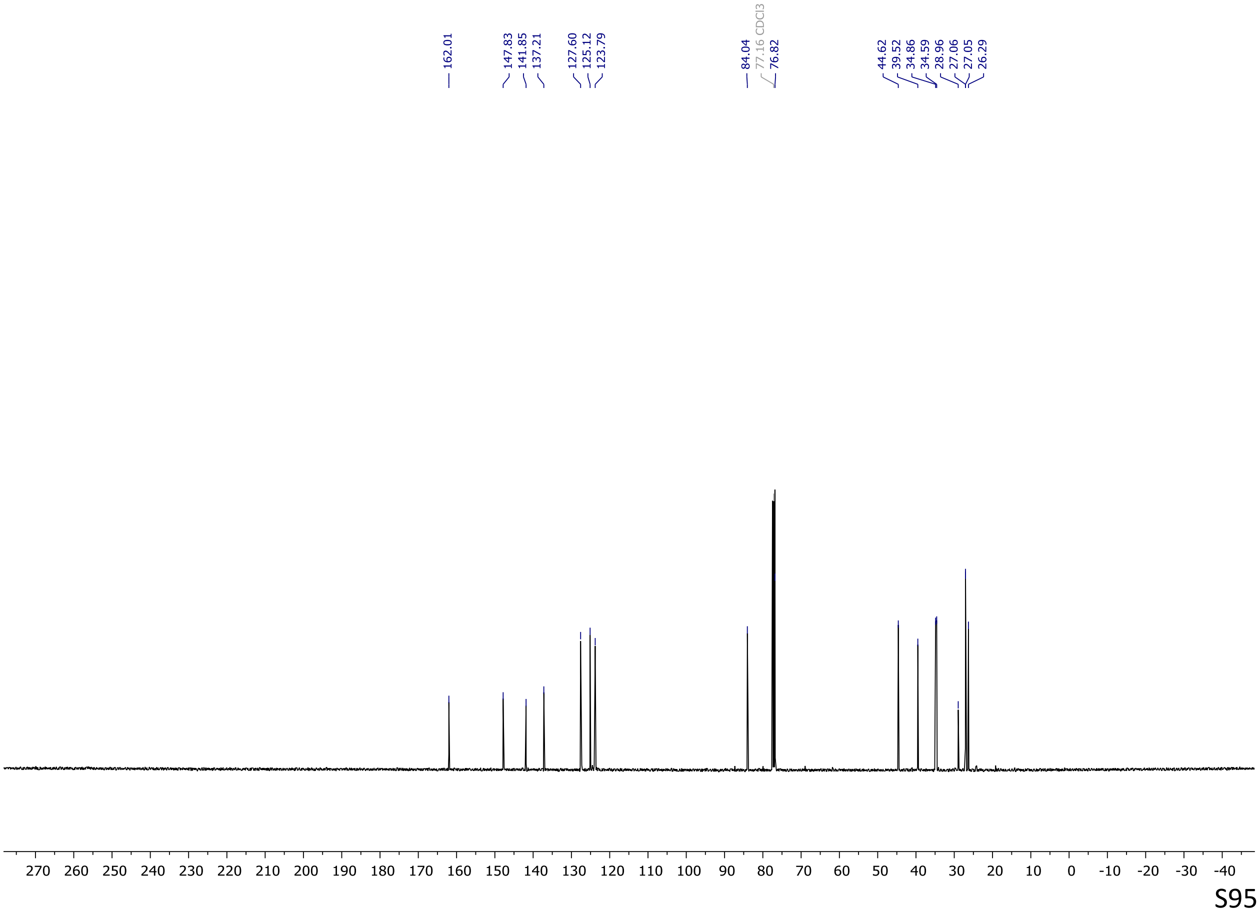
162.01
147.83
141.85
137.21
127.60
125.12
123.79
84.04
77.16 CDCl3
76.82
44.62
39.52
34.86
34.59
28.96
27.06
27.05
26.29
270 260 250 240 230 220 210 200 190 180 170 160 150 140 130 120 110 100 90 80 70 60 50 40 30 20 10 0 -10 -20 -30 -40

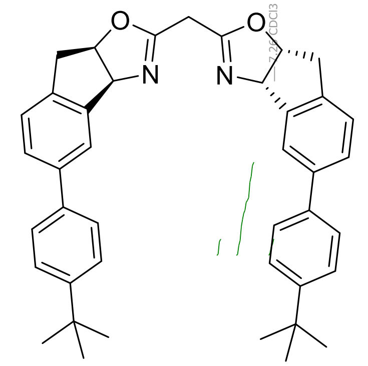

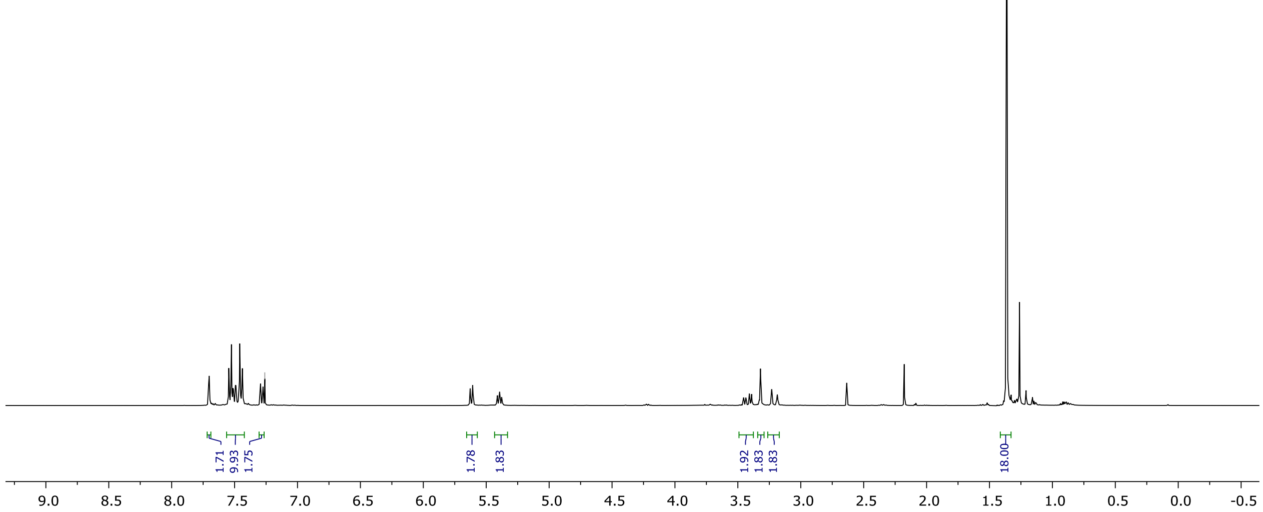
7.26 CDCl3
1.71
9.93
1.75
1.78
1.83
1.92
1.83
1.83
18.00
9.0 8.5 8.0 7.5 7.0 6.5 6.0 5.5 5.0 4.5 4.0 3.5 3.0 2.5 2.0 1.5 1.0 0.5 0.0 -0.5

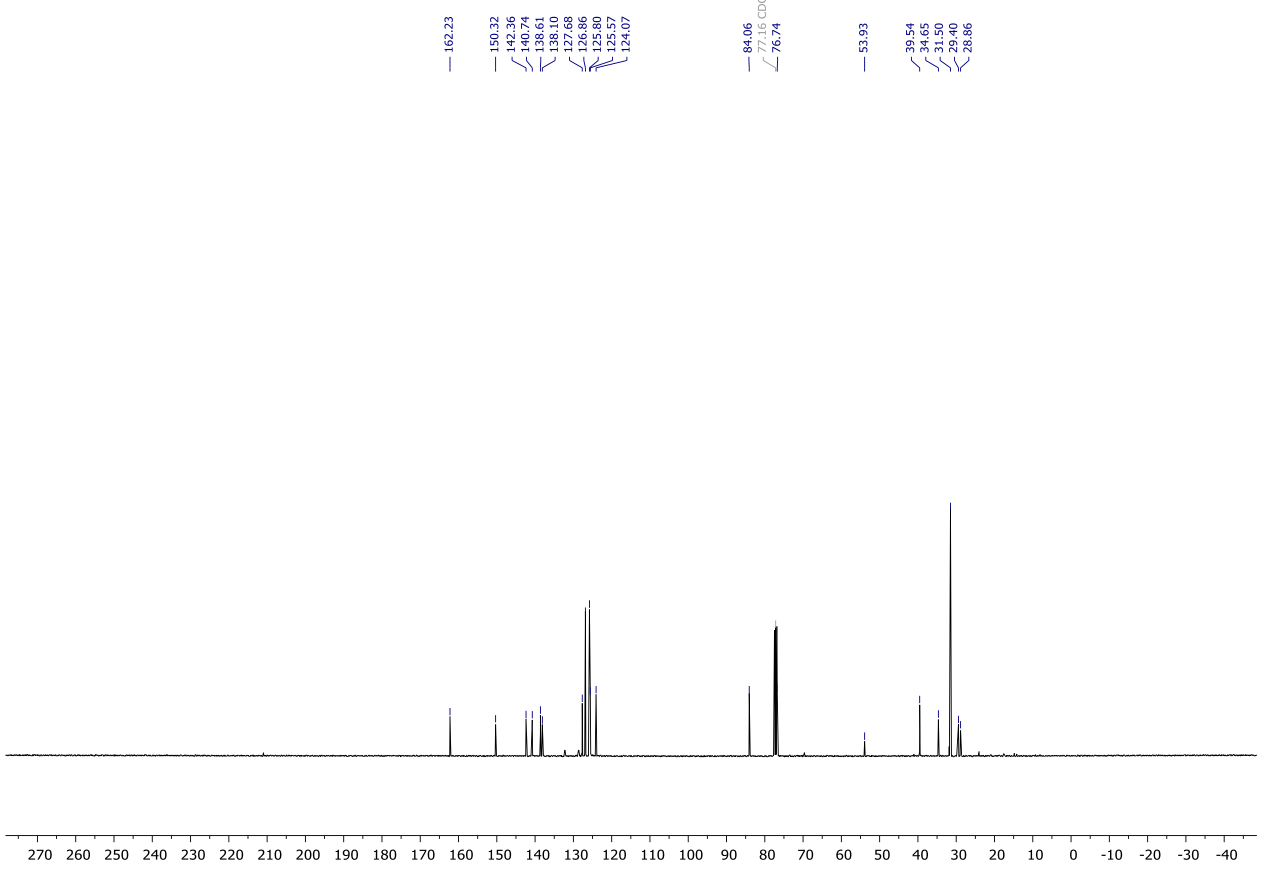
162.23
150.32
142.36
140.74
138.61
138.10
127.68
126.86
125.80
125.57
124.07
84.06
77.16 CDCl3
76.74
53.93
39.54
34.65
31.50
29.40
28.86
270 260 250 240 230 220 210 200 190 180 170 160 150 140 130 120 110 100 90 80 70 60 50 40 30 20 10 0 -10 -20 -30 -40

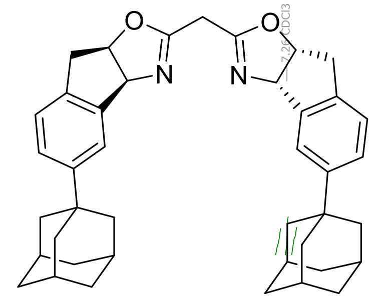
O
N
O
N

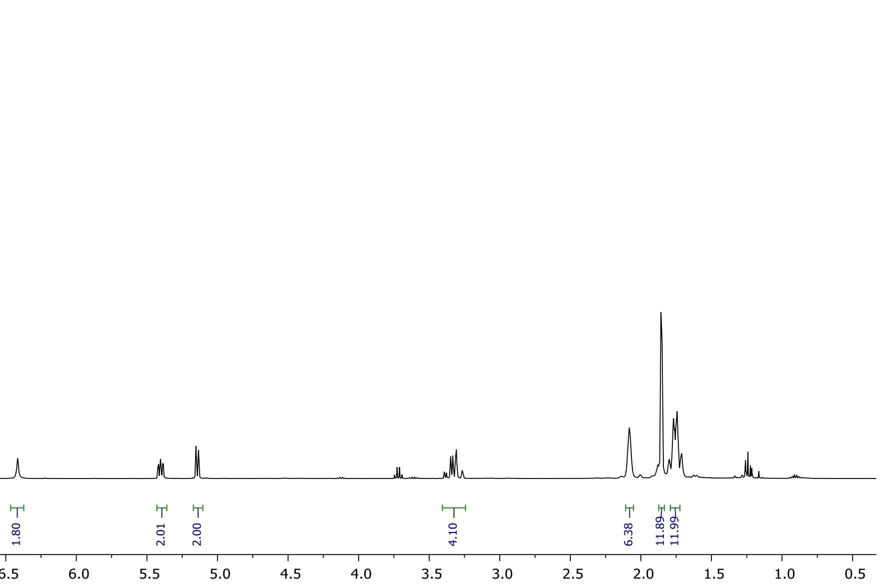
7.26 CDCl3
1.90
2.73
1.99
1.80
2.01
2.00
4.10
6.38
11.89
11.99
9.0 8.5 8.0 7.5 7.0 6.5 6.0 5.5 5.0 4.5 4.0 3.5 3.0 2.5 2.0 1.5 1.0 0.5

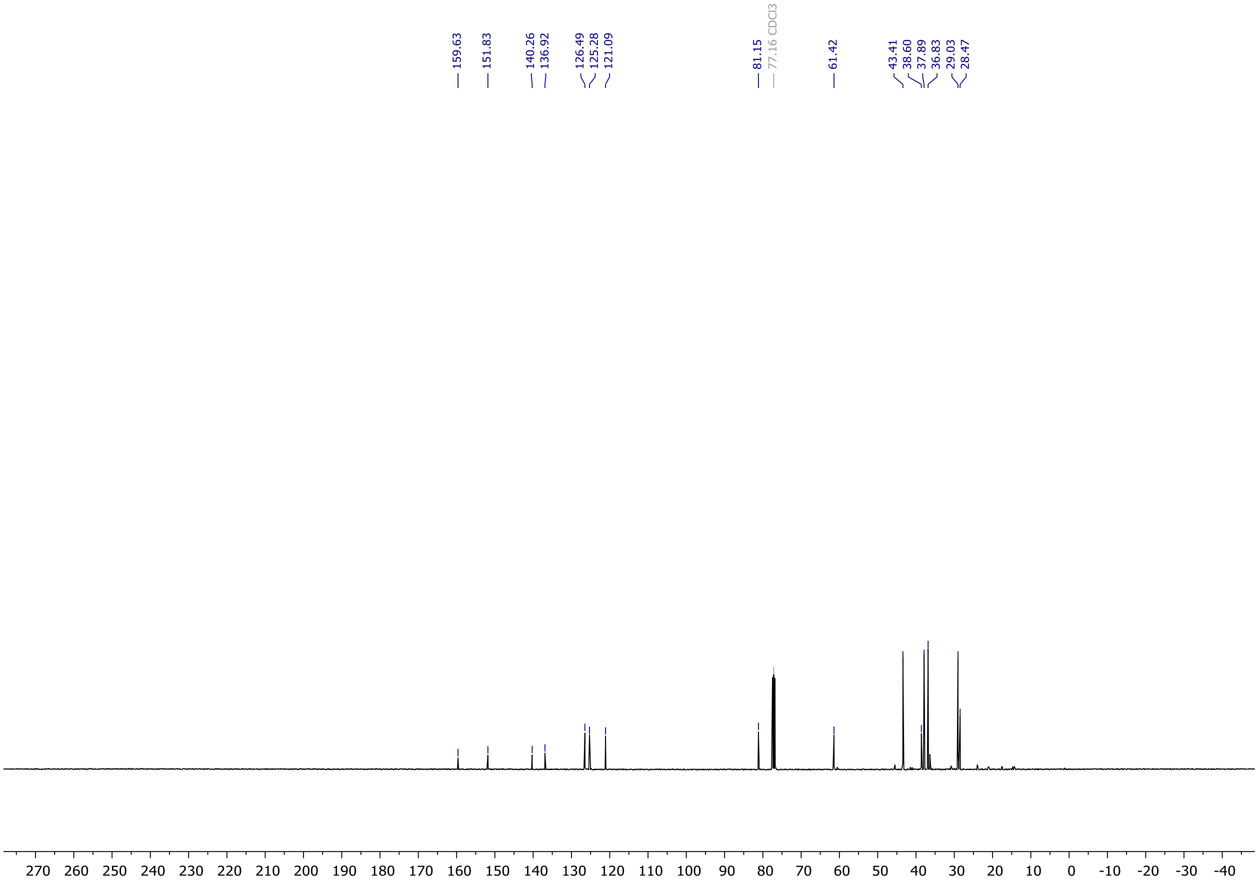
159.63
151.83
140.26
136.92
126.49
125.28
121.09
81.15
77.16 CDCl3
61.42
43.41
38.60
37.89
36.83
29.03
28.47
270 260 250 240 230 220 210 200 190 180 170 160 150 140 130 120 110 100 90 80 70 60 50 40 30 20 10 0 -10 -20 -30 -40

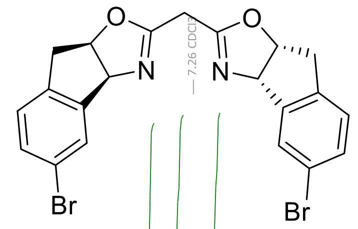
O
N
O
N
Br
Br

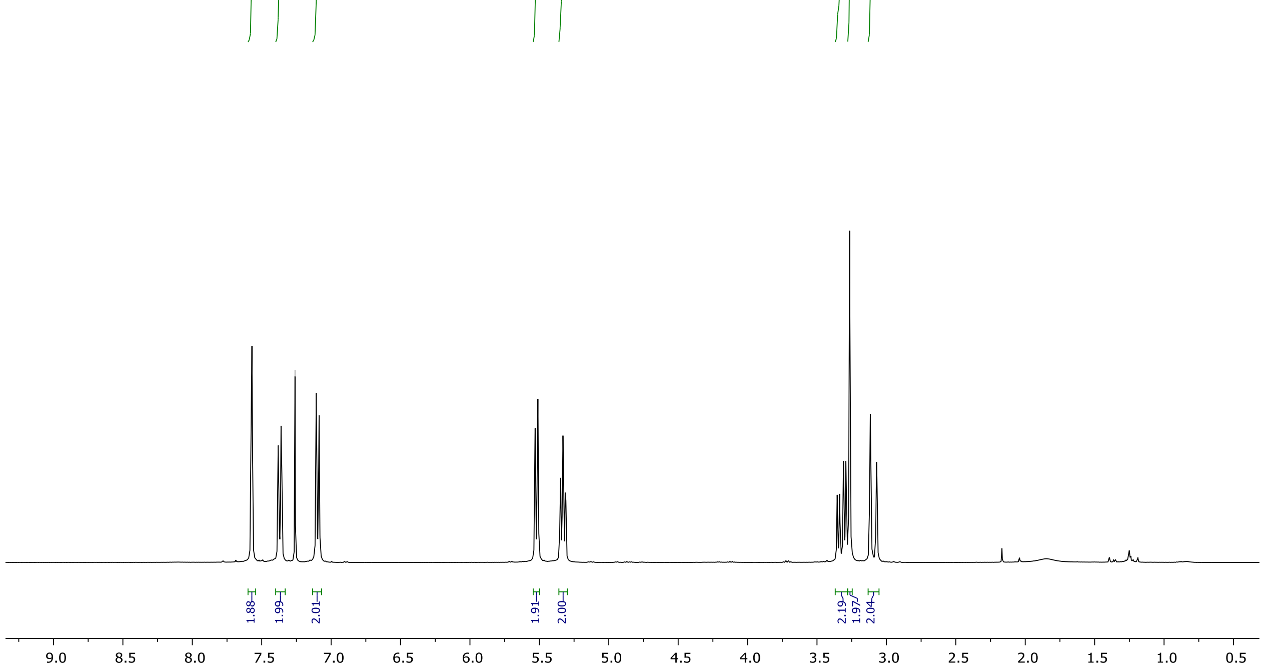
7.26 CDCl3
1.88
1.99
2.01
1.91
2.00
2.19
1.97
2.04
9.0 8.5 8.0 7.5 7.0 6.5 6.0 5.5 5.0 4.5 4.0 3.5 3.0 2.5 2.0 1.5 1.0 0.5

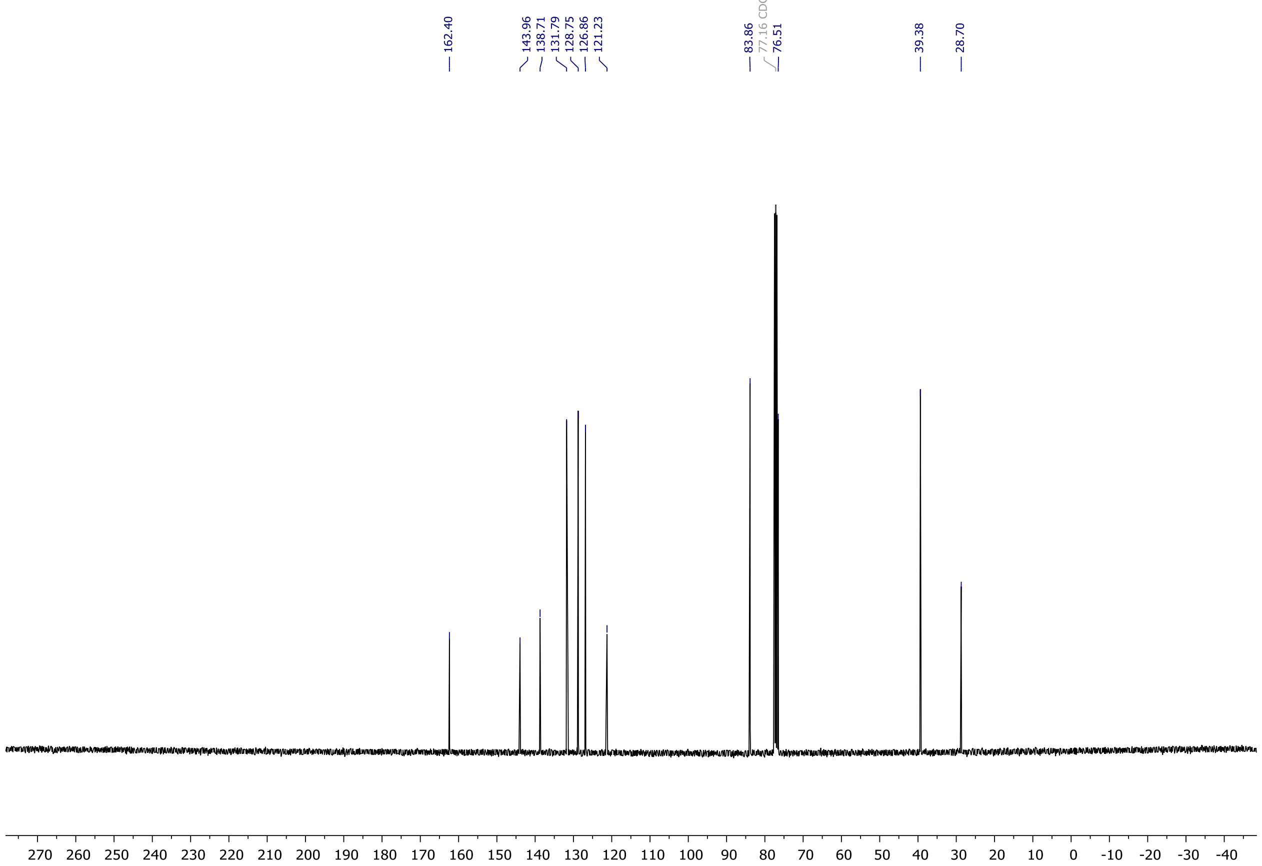
162.40
143.96
138.71
131.79
128.75
126.86
121.23
83.86
77.16 CDCl3
76.51
39.38
28.70
270 260 250 240 230 220 210 200 190 180 170 160 150 140 130 120 110 100 90 80 70 60 50 40 30 20 10 0 -10 -20 -30 -40

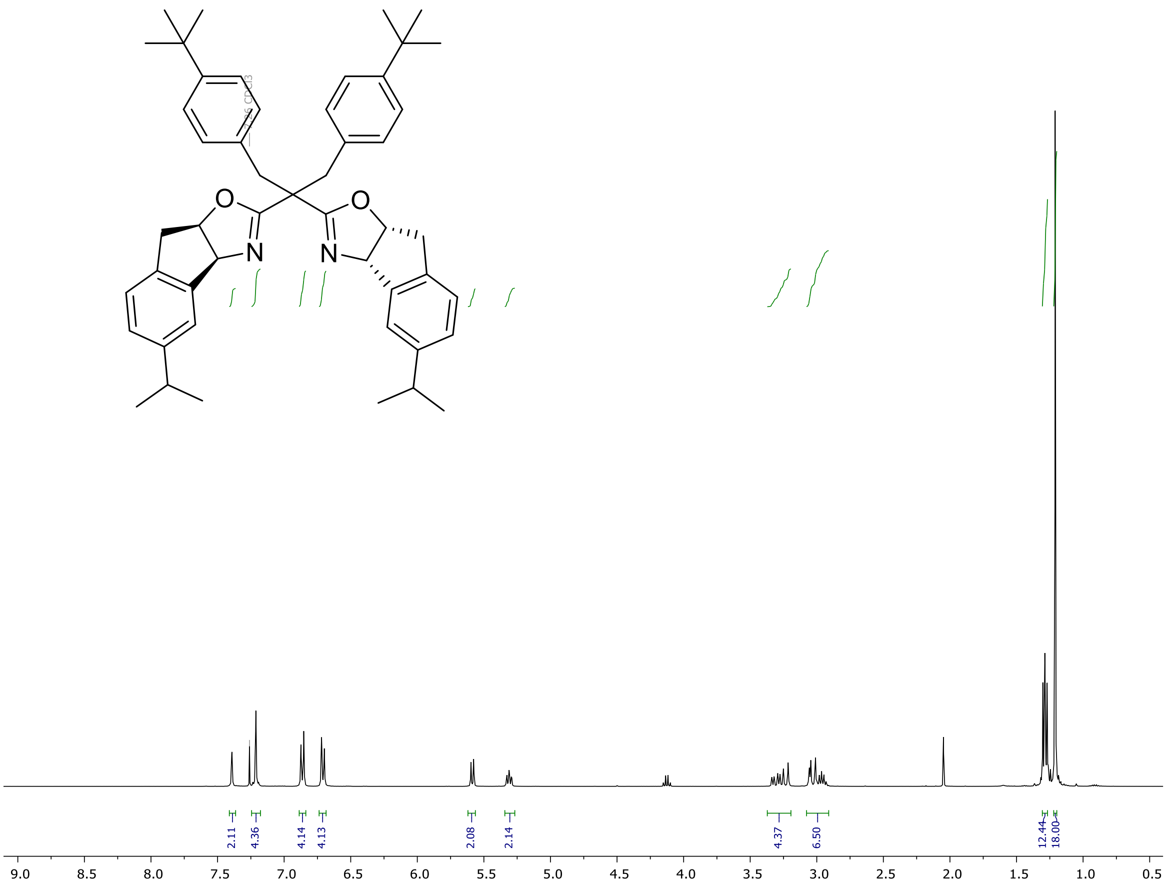
O
N
O
N
7.26 CDCl3
2.11
4.36
4.14
4.13
2.08
2.14
4.37
6.50
12.44
18.00
9.0 8.5 8.0 7.5 7.0 6.5 6.0 5.5 5.0 4.5 4.0 3.5 3.0 2.5 2.0 1.5 1.0 0.5

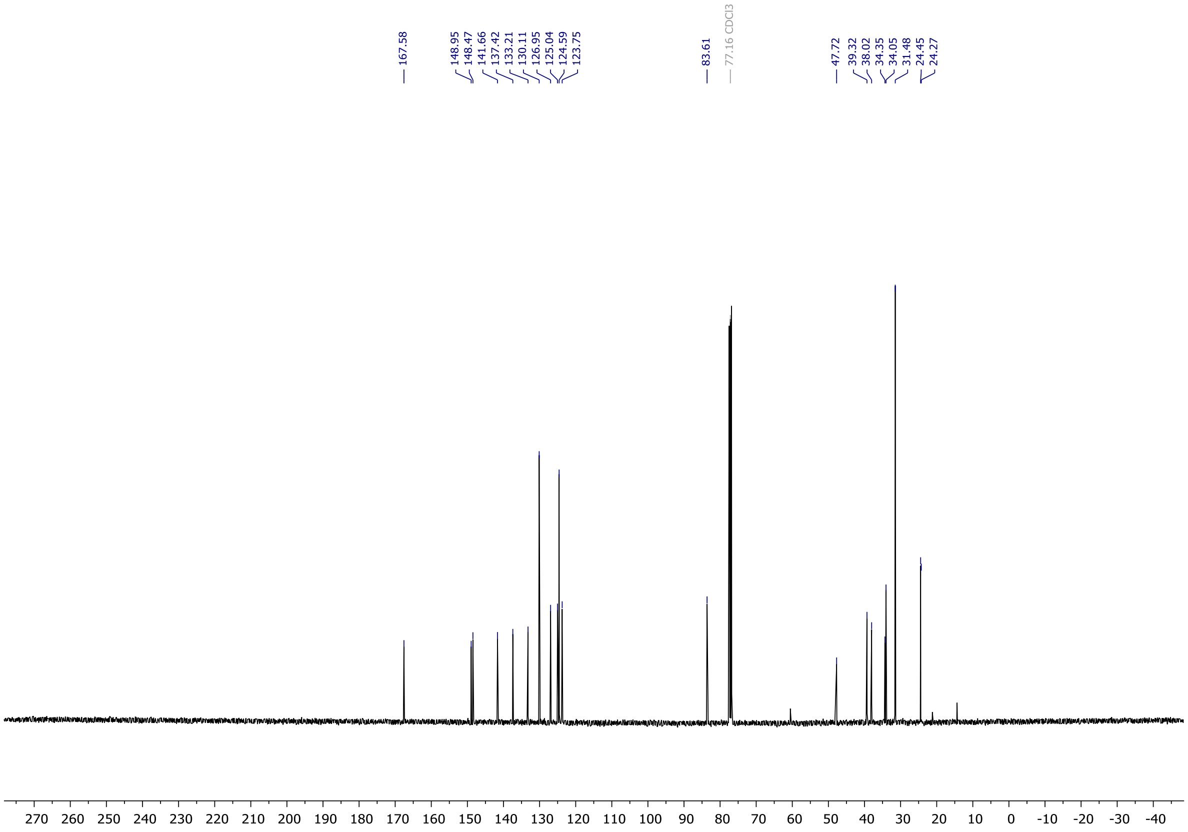
167.58
148.95
148.47
141.66
137.42
133.21
130.11
126.95
125.04
124.59
123.75
83.61
77.16 CDCl3
47.72
39.32
38.02
34.35
34.05
31.48
24.45
24.27
270 260 250 240 230 220 210 200 190 180 170 160 150 140 130 120 110 100 90 80 70 60 50 40 30 20 10 0 -10 -20 -30 -40

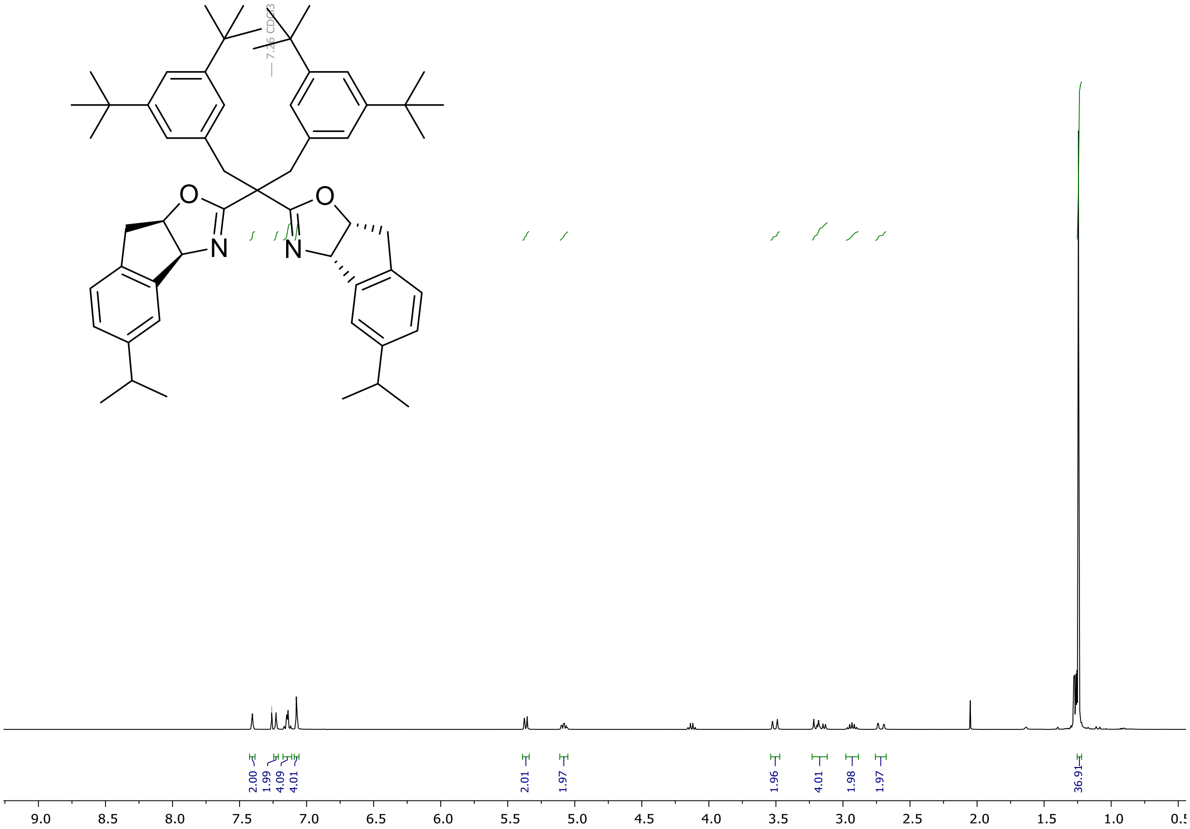

7.26 CDCl3
2.00
1.99
4.09
4.01
2.01
1.97
1.96
4.01
1.98
1.97
36.91
9.0 8.5 8.0 7.5 7.0 6.5 6.0 5.5 5.0 4.5 4.0 3.5 3.0 2.5 2.0 1.5 1.0 0.5

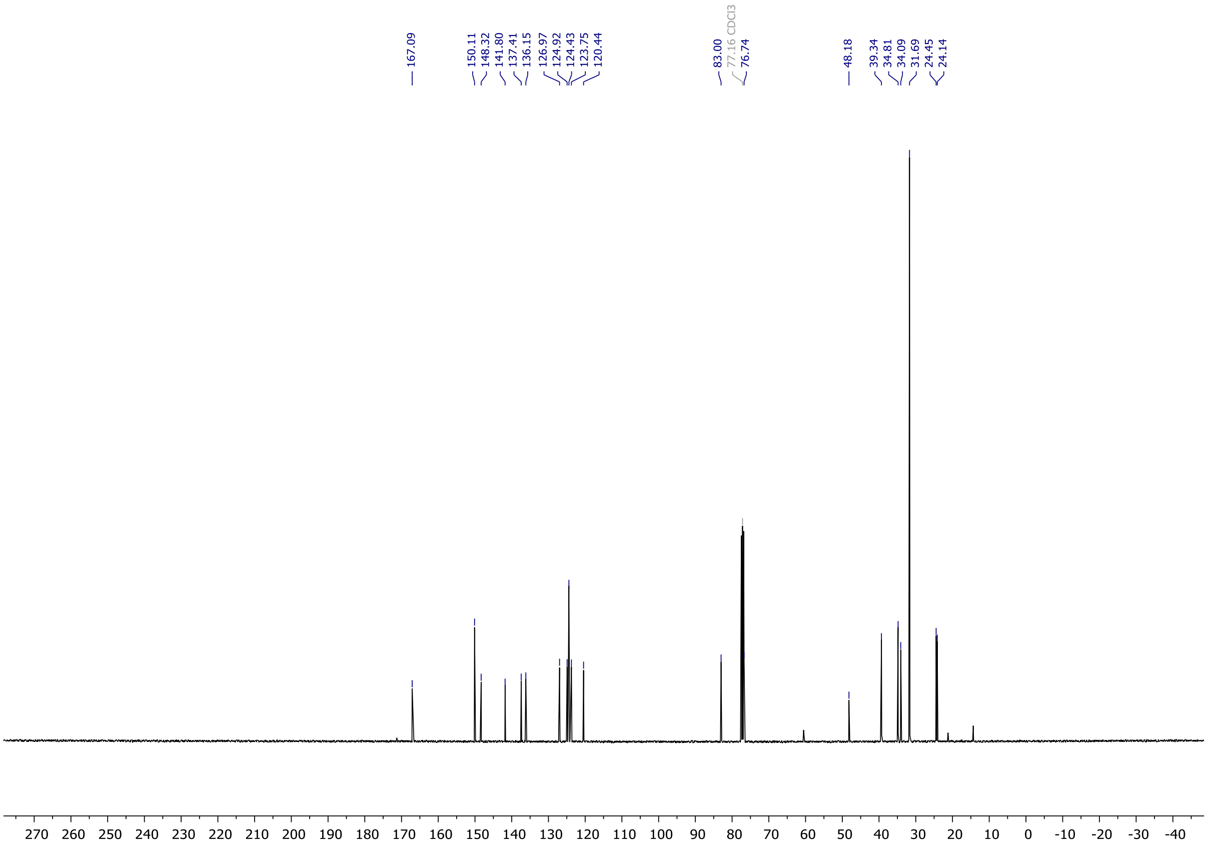

167.09
150.11
148.32
141.80
137.41
136.15
126.97
124.92
124.43
123.75
120.44
83.00
77.16 CDCl3
76.74
48.18
39.34
34.81
34.09
31.69
24.45
24.14
270 260 250 240 230 220 210 200 190 180 170 160 150 140 130 120 110 100 90 80 70 60 50 40 30 20 10 0 -10 -20 -30 -40

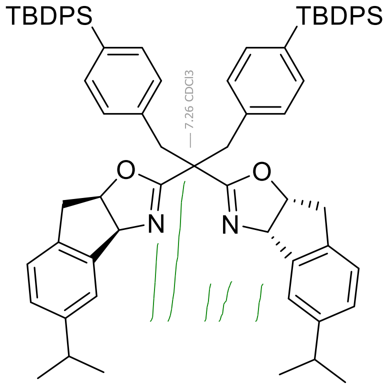
TBDPS
TBDPS
O
N
O
N

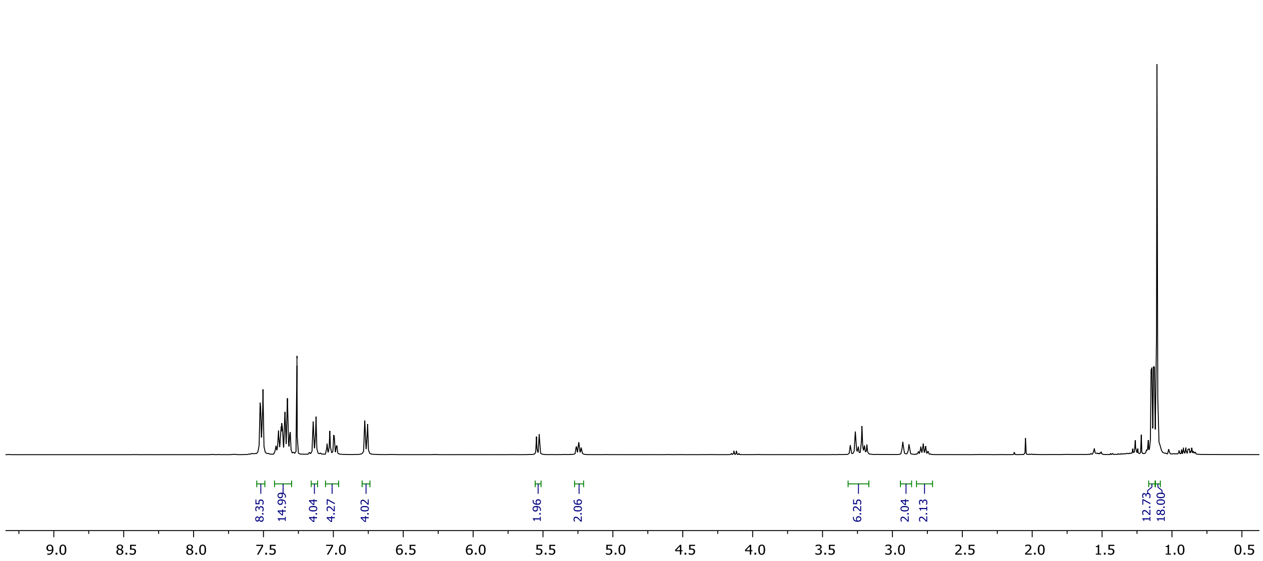
7.26 CDCl3
8.35
14.99
4.04
4.27
4.02
1.96
2.06
6.25
2.04
2.13
12.73
18.00
9.0 8.5 8.0 7.5 7.0 6.5 6.0 5.5 5.0 4.5 4.0 3.5 3.0 2.5 2.0 1.5 1.0 0.5

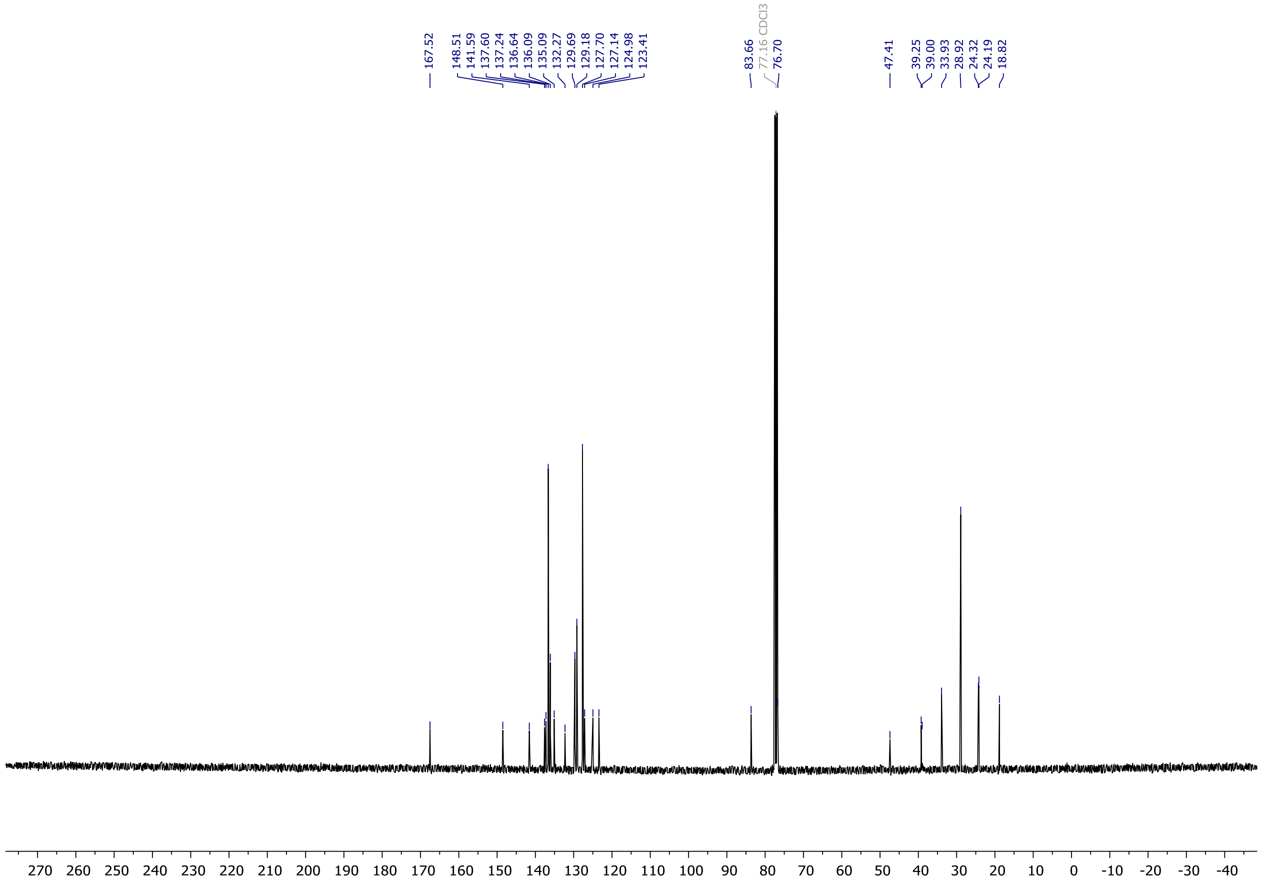
167.52
148.51
141.59
137.60
137.24
136.64
136.09
135.09
132.27
129.69
129.18
127.70
127.14
124.98
123.41
83.66
77.16 CDCl3
76.70
47.41
39.25
39.00
33.93
28.92
24.32
24.19
18.82
270 260 250 240 230 220 210 200 190 180 170 160 150 140 130 120 110 100 90 80 70 60 50 40 30 20 10 0 -10 -20 -30 -40

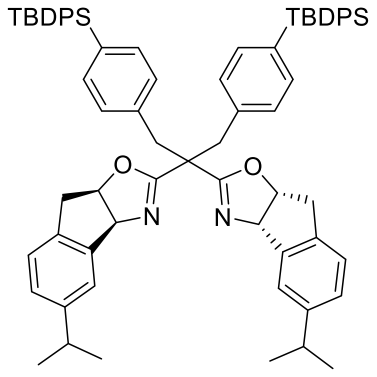
TBDPS
TBDPS
O
N
O
N

^{29}Si NMR

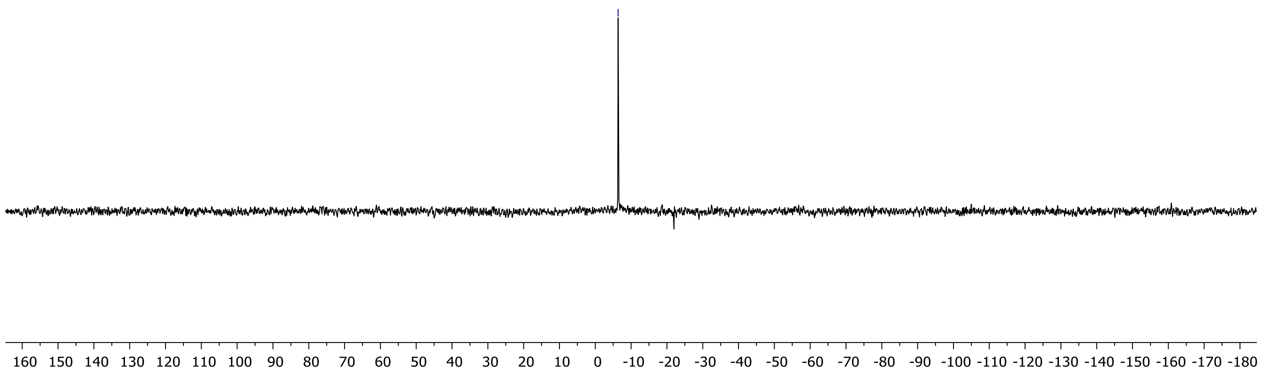
-6.38
160 150 140 130 120 110 100 90 80 70 60 50 40 30 20 10 0 -10 -20 -30 -40 -50 -60 -70 -80 -90 -100 -110 -120 -130 -140 -150 -160 -170 -180

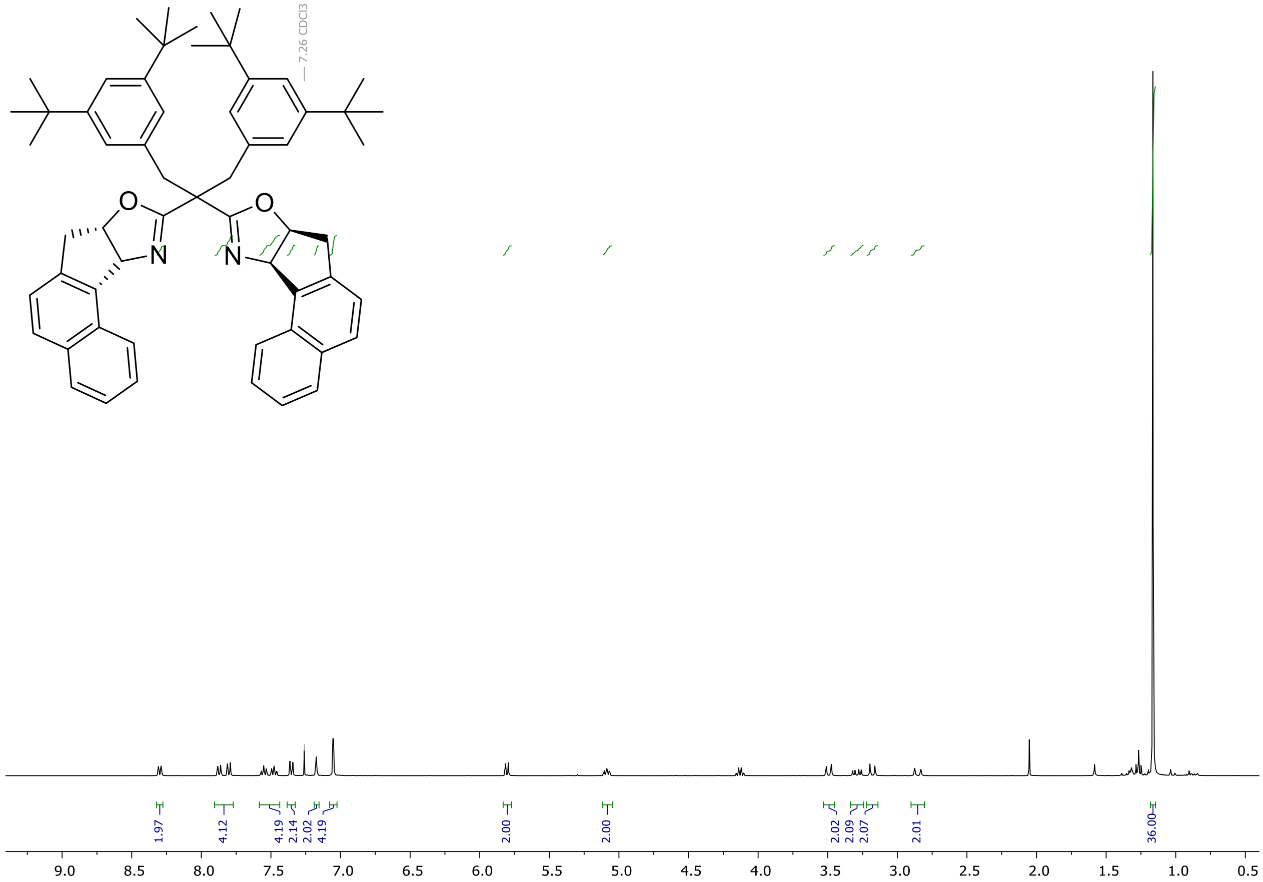

O
N
7.26 CDCl3
1.97
4.12
4.19
2.14
2.02
4.19
2.00
2.00
2.02
2.09
2.07
2.01
36.00
9.0 8.5 8.0 7.5 7.0 6.5 6.0 5.5 5.0 4.5 4.0 3.5 3.0 2.5 2.0 1.5 1.0 0.5

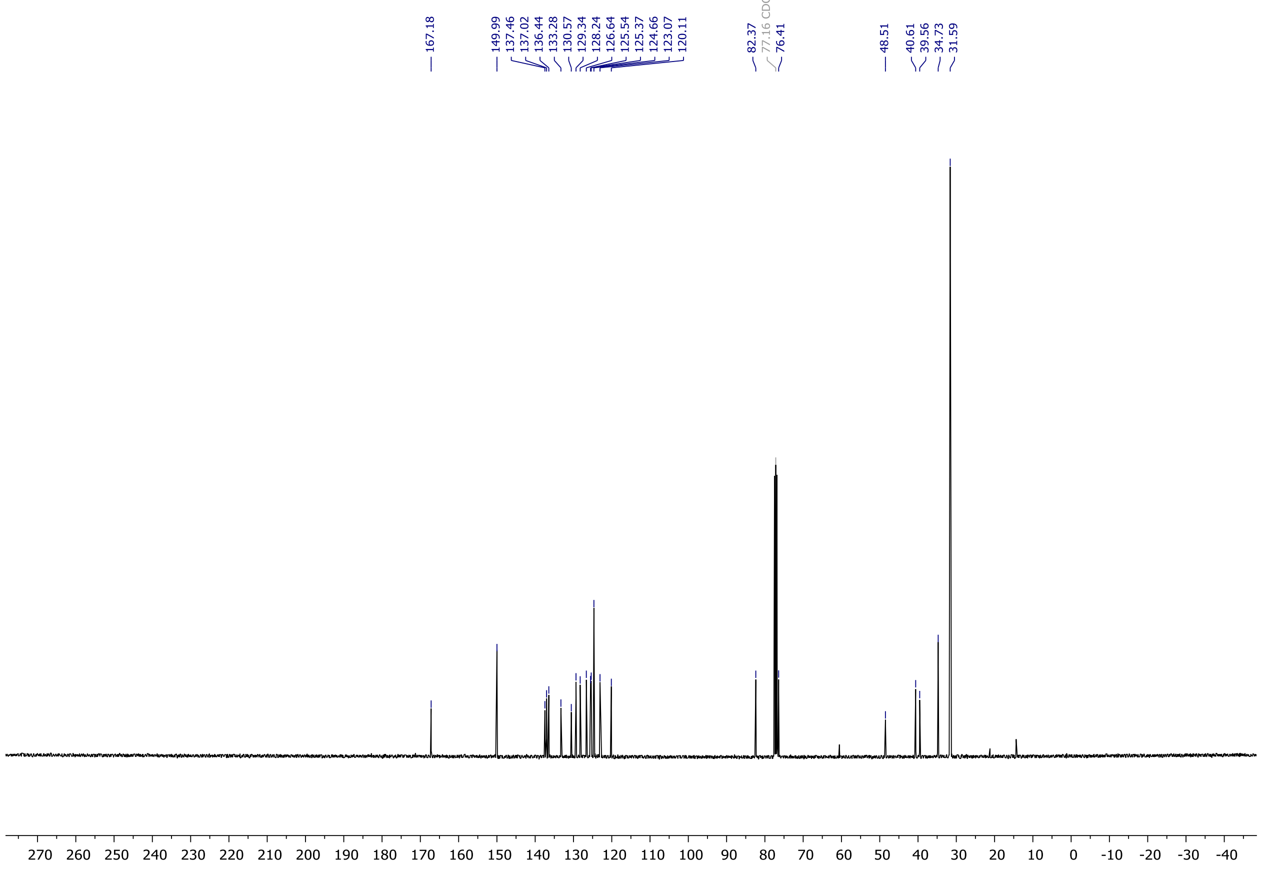

167.18
149.99
137.46
137.02
136.44
133.28
130.57
129.34
128.24
126.64
125.54
125.37
124.66
123.07
120.11
82.37
77.16 CDCl3
76.41
48.51
40.61
39.56
34.73
31.59
270 260 250 240 230 220 210 200 190 180 170 160 150 140 130 120 110 100 90 80 70 60 50 40 30 20 10 0 -10 -20 -30 -40

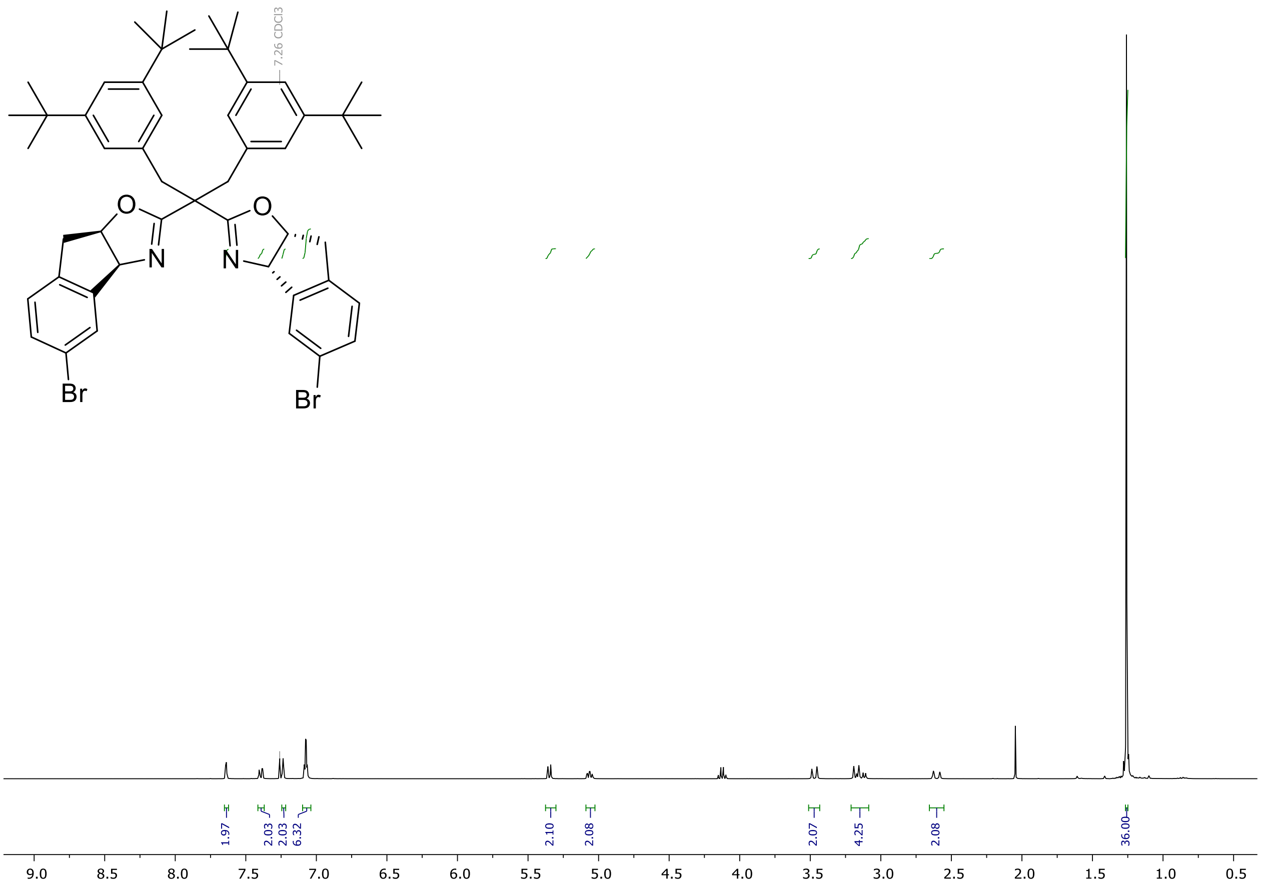
7.26 CDCl3
1.97
2.03
2.03
6.32
2.10
2.08
2.07
4.25
2.08
36.00
9.0 8.5 8.0 7.5 7.0 6.5 6.0 5.5 5.0 4.5 4.0 3.5 3.0 2.5 2.0 1.5 1.0 0.5
Br
Br
O
O
N
N

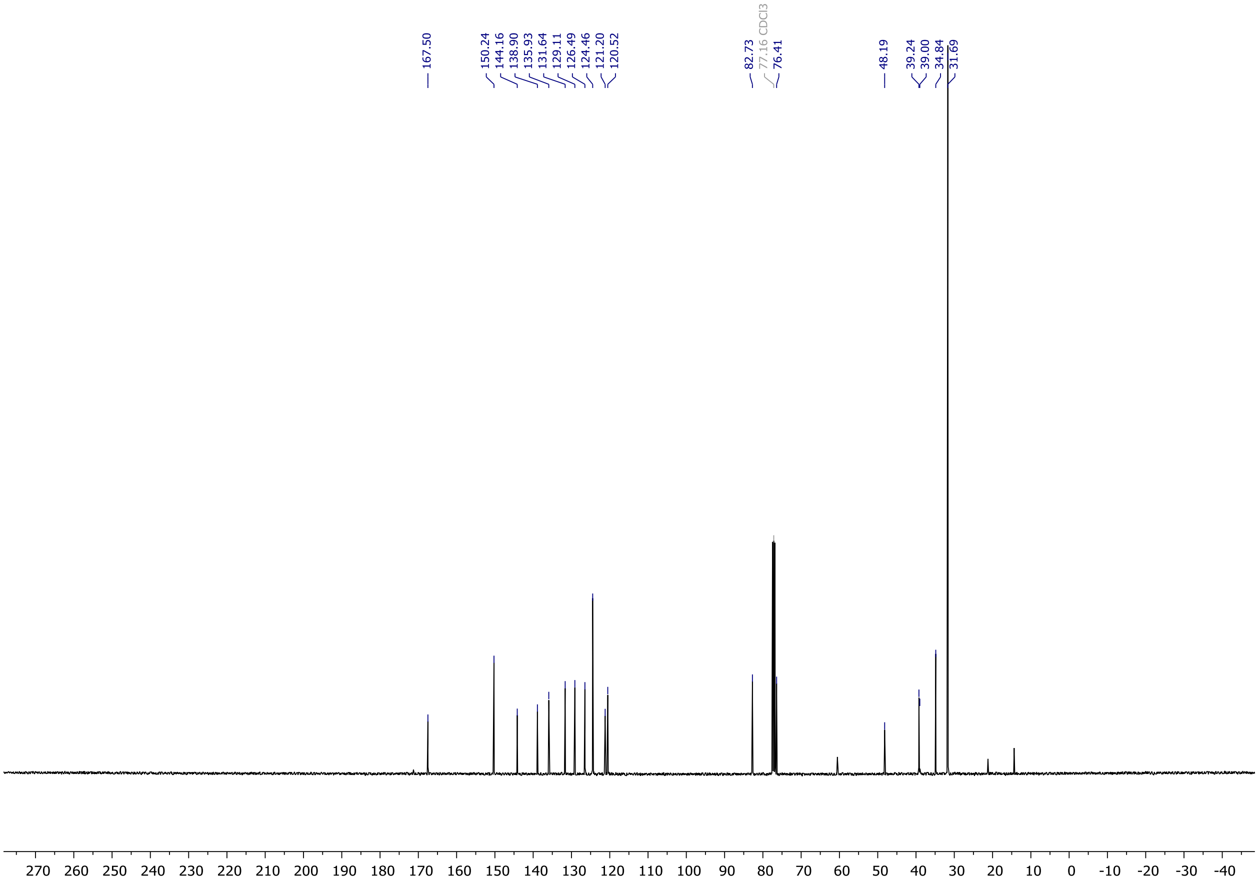
167.50
150.24
144.16
138.90
135.93
131.64
129.11
126.49
124.46
121.20
120.52
82.73
77.16 CDCl3
76.41
48.19
39.24
39.00
34.84
31.69
270 260 250 240 230 220 210 200 190 180 170 160 150 140 130 120 110 100 90 80 70 60 50 40 30 20 10 0 -10 -20 -30 -40

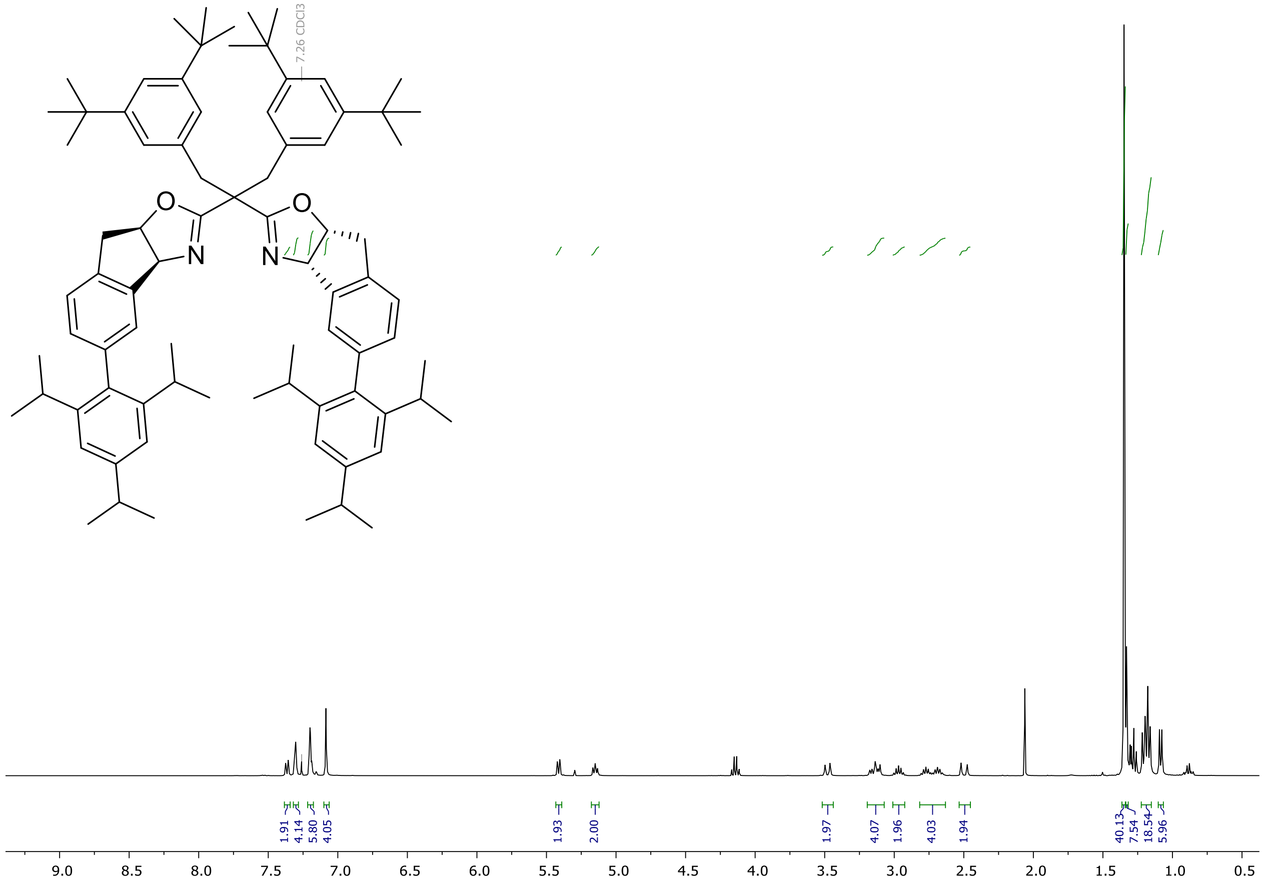
7.26 CDCl3
1.91
4.14
5.80
4.05
1.93
2.00
1.97
4.07
1.96
4.03
1.94
40.13
7.54
18.54
5.96
9.0 8.5 8.0 7.5 7.0 6.5 6.0 5.5 5.0 4.5 4.0 3.5 3.0 2.5 2.0 1.5 1.0 0.5

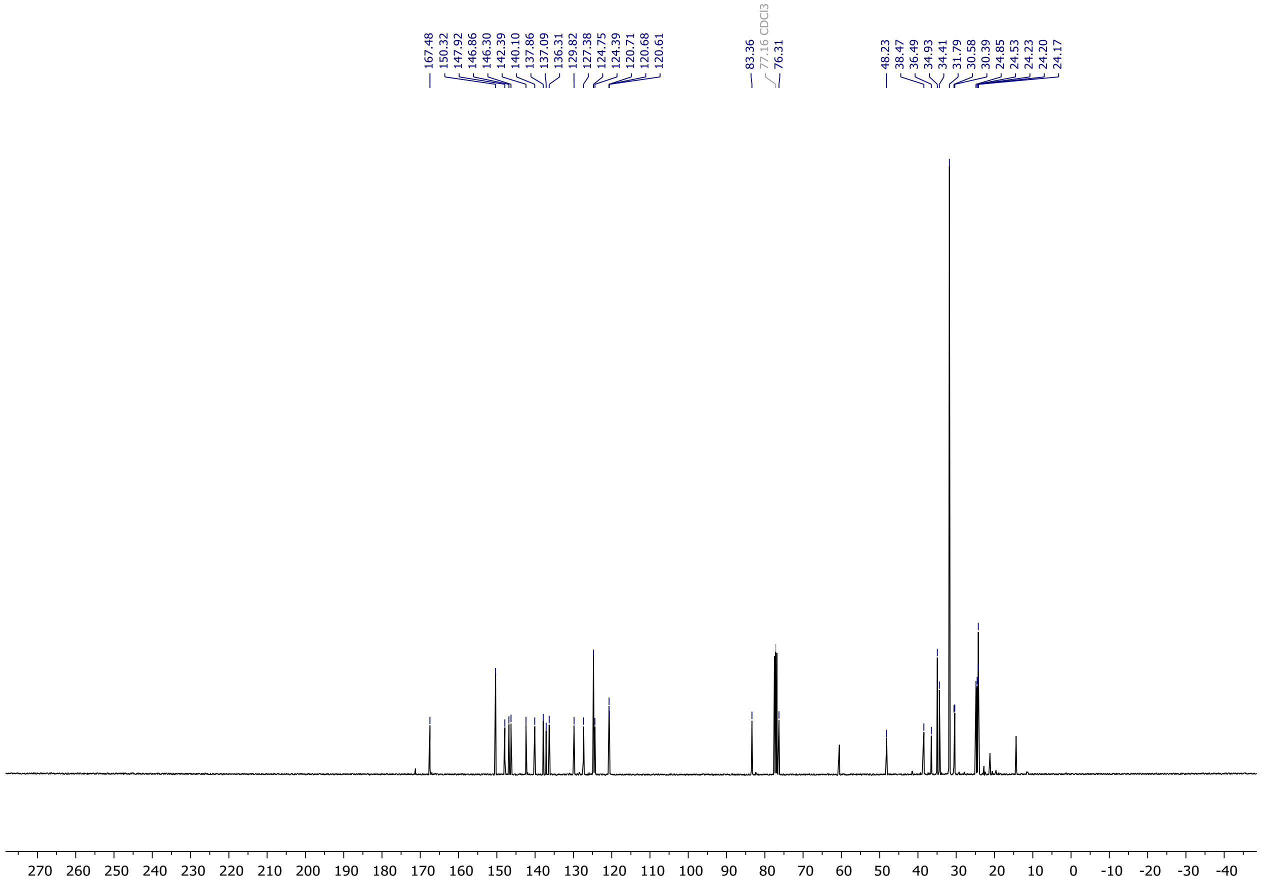
167.48
150.32
147.92
146.86
146.30
142.39
140.10
137.86
137.09
136.31
129.82
127.38
124.75
124.39
120.71
120.68
120.61
83.36
77.16 CDCl3
76.31
48.23
38.47
36.49
34.93
34.41
31.79
30.58
30.39
24.85
24.53
24.23
24.20
24.17
270 260 250 240 230 220 210 200 190 180 170 160 150 140 130 120 110 100 90 80 70 60 50 40 30 20 10 0 -10 -20 -30 -40

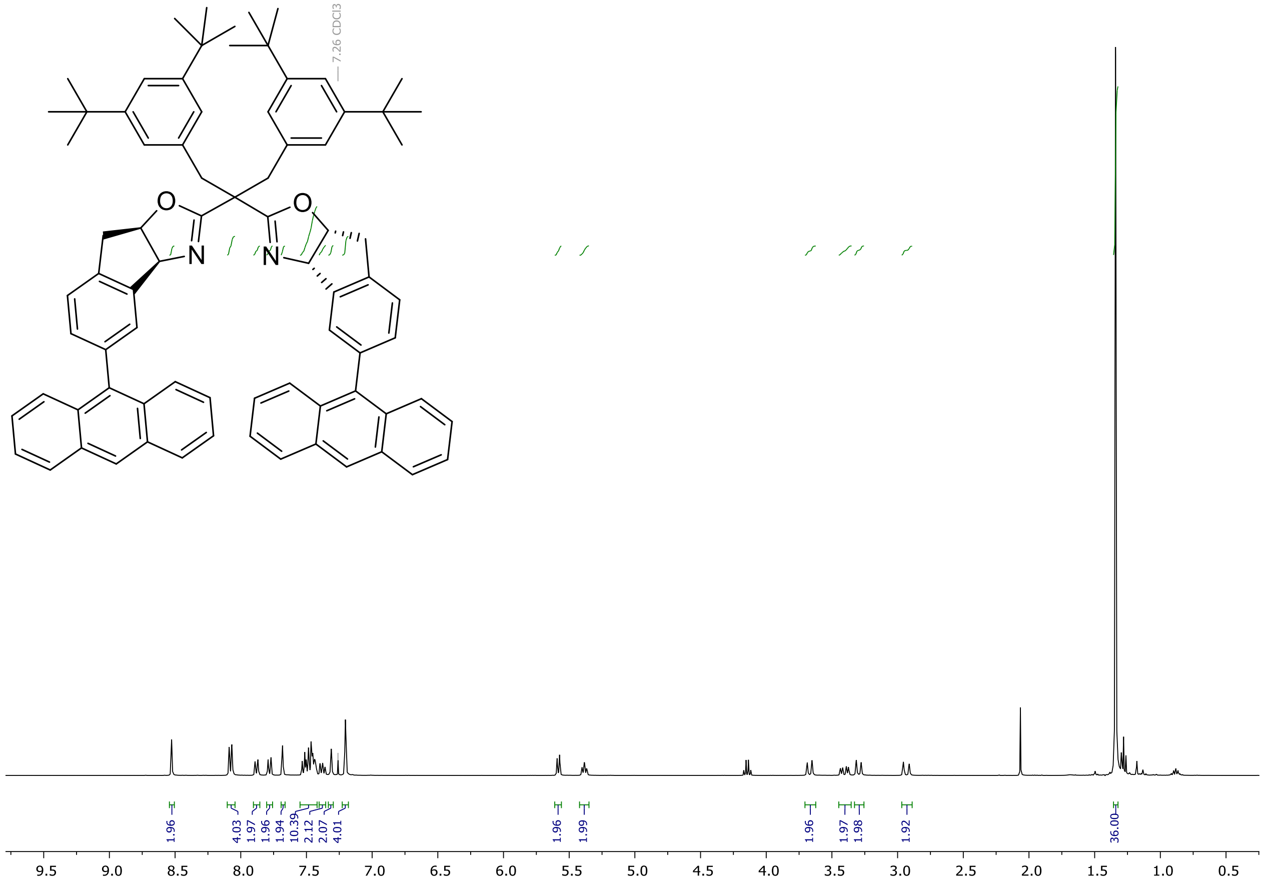
7.26 CDCl3
O
N
O
N
1.96
4.03
1.97
1.96
1.94
10.39
2.12
2.07
4.01
1.96
1.99
1.96
1.97
1.98
1.92
36.00
9.5 9.0 8.5 8.0 7.5 7.0 6.5 6.0 5.5 5.0 4.5 4.0 3.5 3.0 2.5 2.0 1.5 1.0 0.5

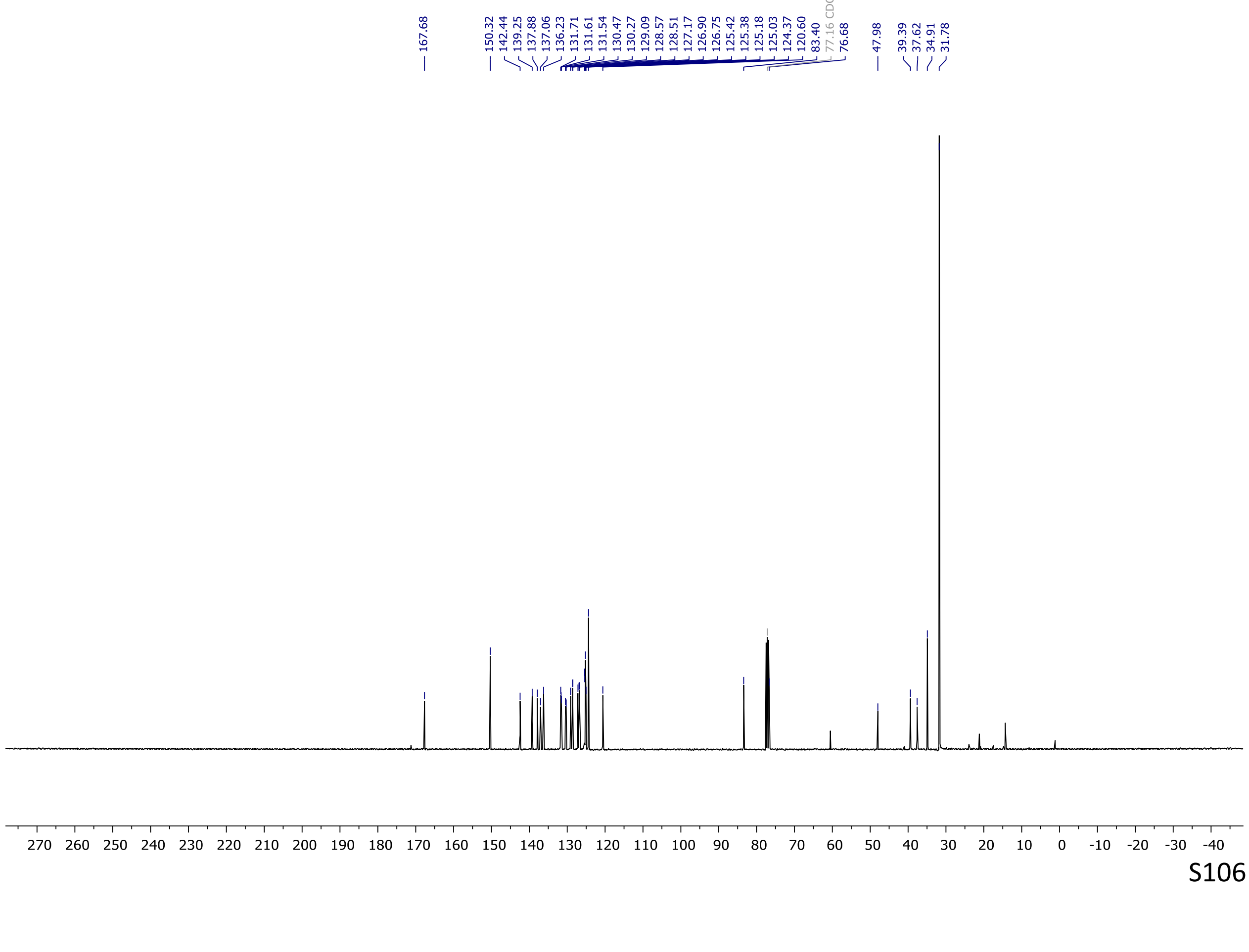
167.68
150.32
142.44
139.25
137.88
137.06
136.23
131.71
131.61
131.54
130.47
130.27
129.09
128.57
128.51
127.17
126.90
126.75
125.42
125.38
125.18
125.03
124.37
120.60
83.40
77.16 CDCl3
76.68
47.98
39.39
37.62
34.91
31.78
270 260 250 240 230 220 210 200 190 180 170 160 150 140 130 120 110 100 90 80 70 60 50 40 30 20 10 0 -10 -20 -30 -40

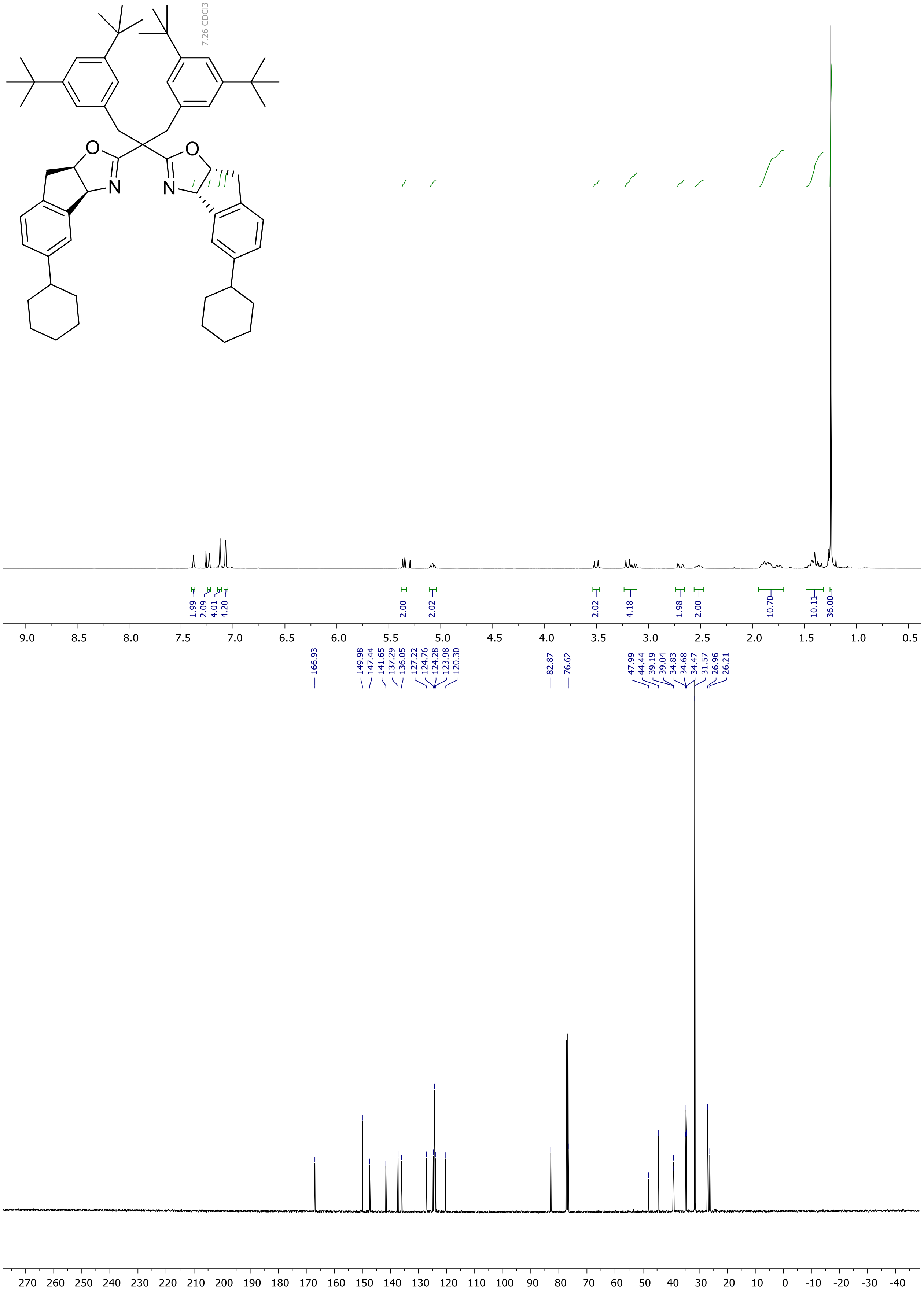
7.26 CDCl3
1.99
2.09
4.01
4.20
2.00
2.02
2.02
4.18
1.98
2.00
10.70
10.11
36.00
9.0 8.5 8.0 7.5 7.0 6.5 6.0 5.5 5.0 4.5 4.0 3.5 3.0 2.5 2.0 1.5 1.0 0.5
166.93
149.98
147.44
141.65
137.29
136.05
127.22
124.76
124.28
123.98
120.30
82.87
76.62
47.99
44.44
39.19
39.04
34.83
34.68
34.47
31.57
26.96
26.21
270 260 250 240 230 220 210 200 190 180 170 160 150 140 130 120 110 100 90 80 70 60 50 40 30 20 10 0 -10 -20 -30 -40

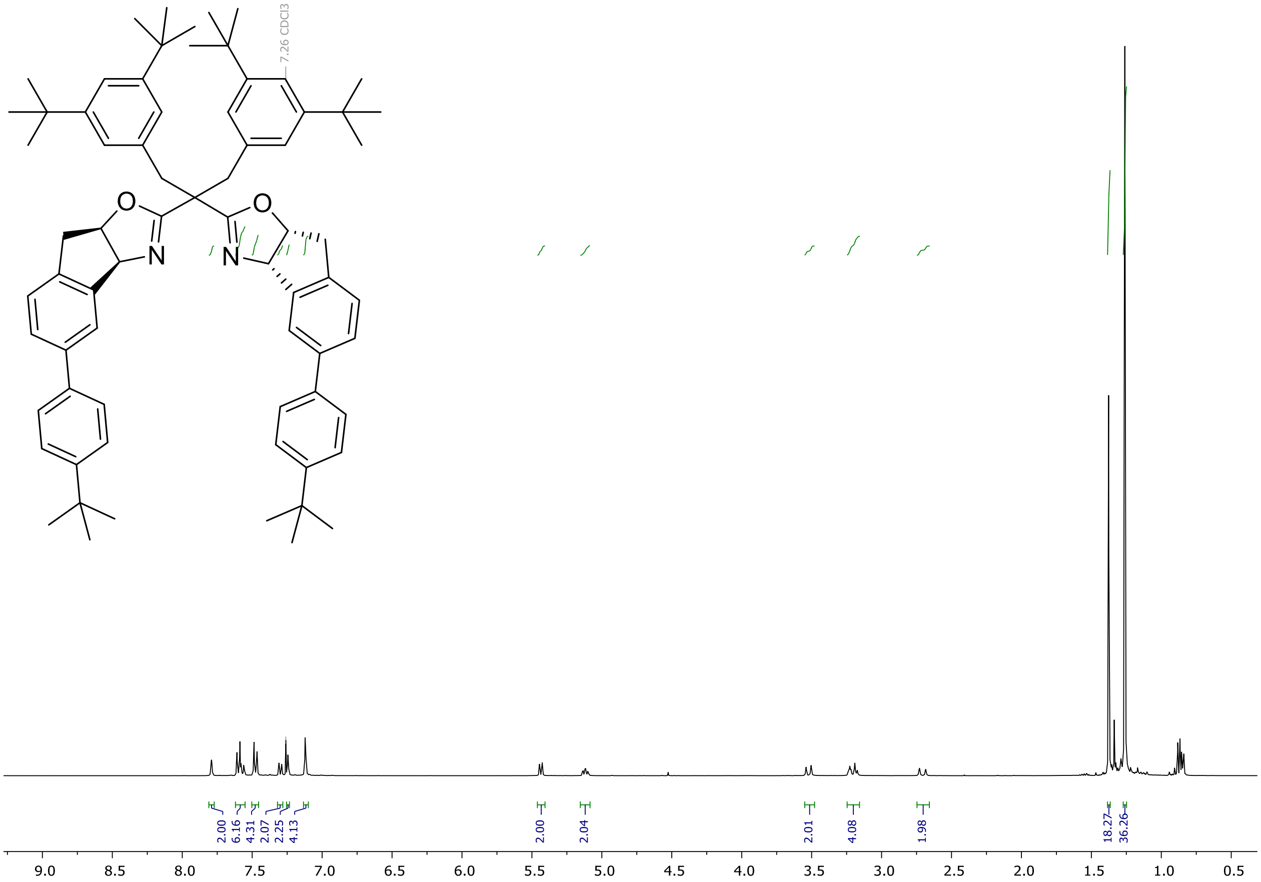

7.26 CDCl3
2.00
6.16
4.31
2.07
2.25
4.13
2.00
2.04
2.01
4.08
1.98
18.27
36.26
9.0
8.5
8.0
7.5
7.0
6.5
6.0
5.5
5.0
4.5
4.0
3.5
3.0
2.5
2.0
1.5
1.0
0.5

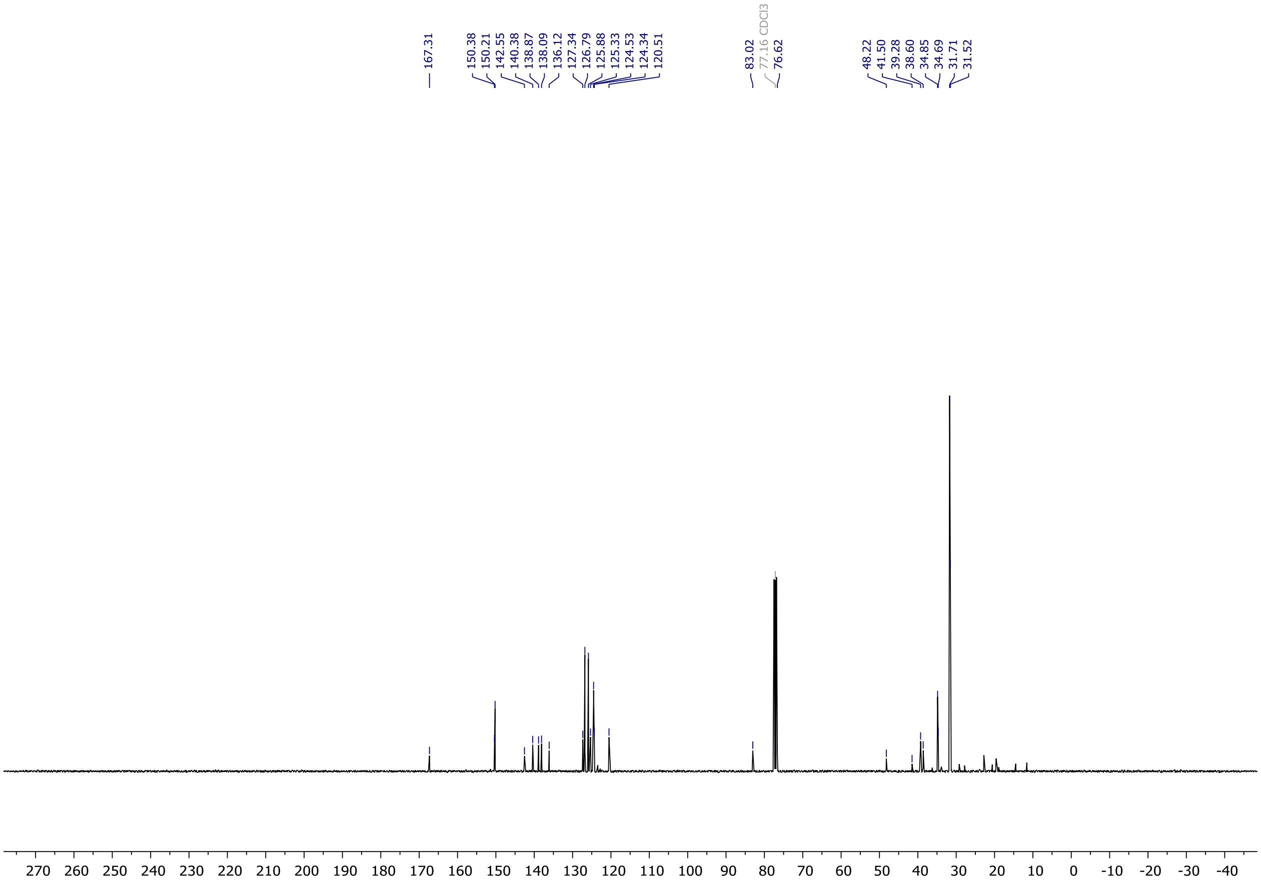

167.31
150.38
150.21
142.55
140.38
138.87
138.09
136.12
127.34
126.79
125.88
125.33
124.53
124.34
120.51
83.02
77.16 CDCl3
76.62
48.22
41.50
39.28
38.60
34.85
34.69
31.71
31.52
270 260 250 240 230 220 210 200 190 180 170 160 150 140 130 120 110 100 90 80 70 60 50 40 30 20 10 0 -10 -20 -30 -40

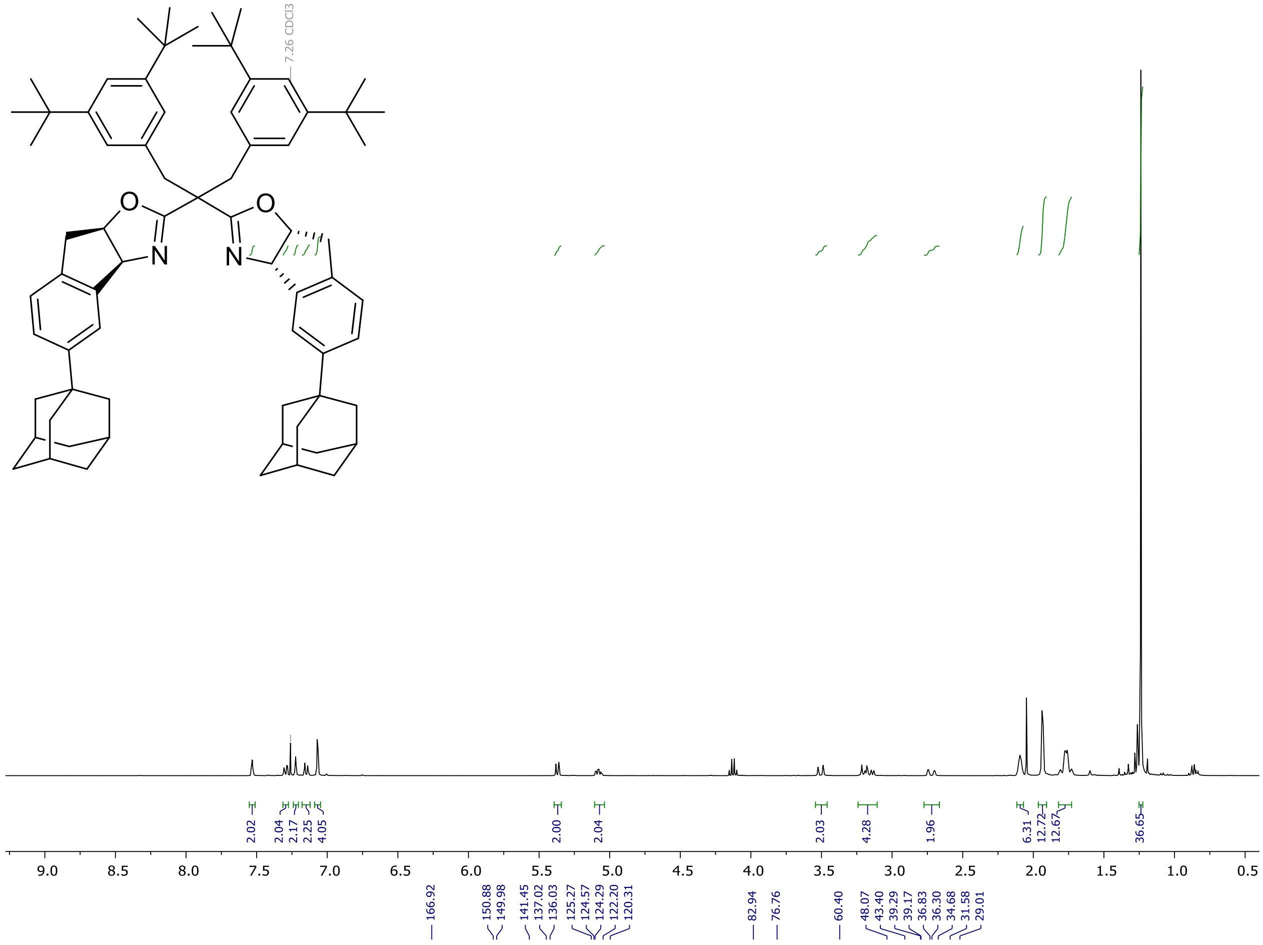
7.26 CDCl3
2.02
2.04
2.17
2.25
4.05
2.00
2.04
2.03
4.28
1.96
6.31
12.72
12.67
36.65
166.92
150.88
149.98
141.45
137.02
136.03
125.27
124.57
124.29
122.20
120.31
82.94
76.76
60.40
48.07
43.40
39.29
39.17
36.83
36.30
34.68
31.58
29.01

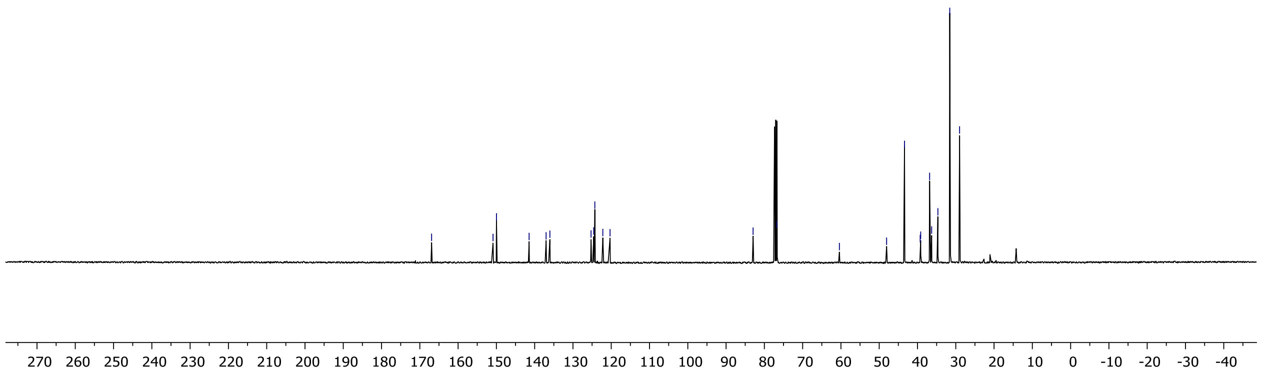

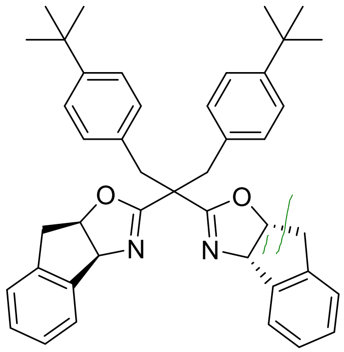

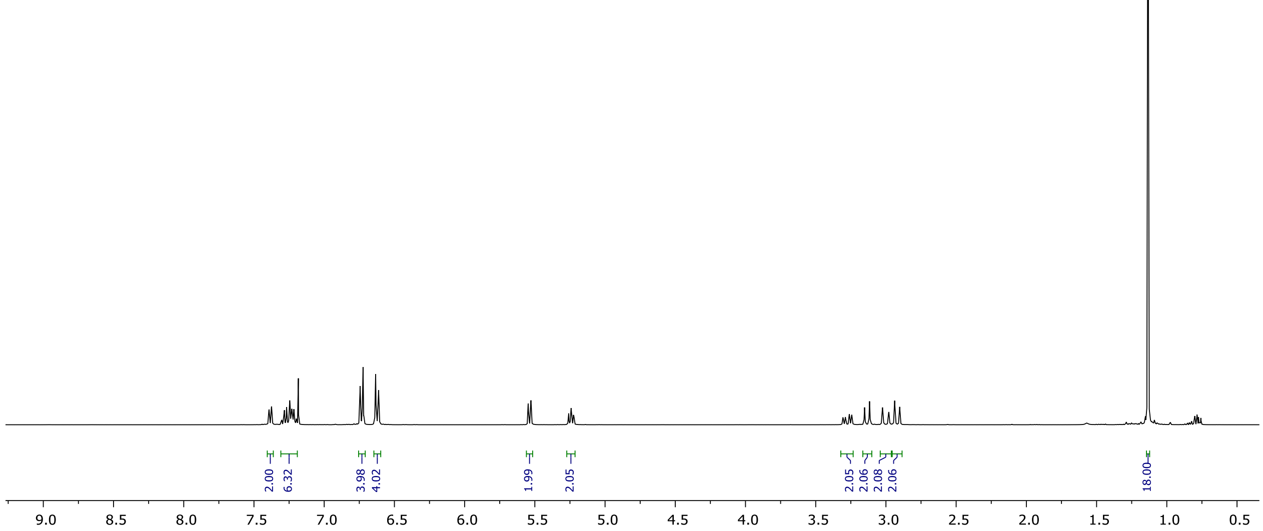
2.00
6.32
3.98
4.02
1.99
2.05
2.05
2.06
2.08
2.06
18.00
9.0 8.5 8.0 7.5 7.0 6.5 6.0 5.5 5.0 4.5 4.0 3.5 3.0 2.5 2.0 1.5 1.0 0.5

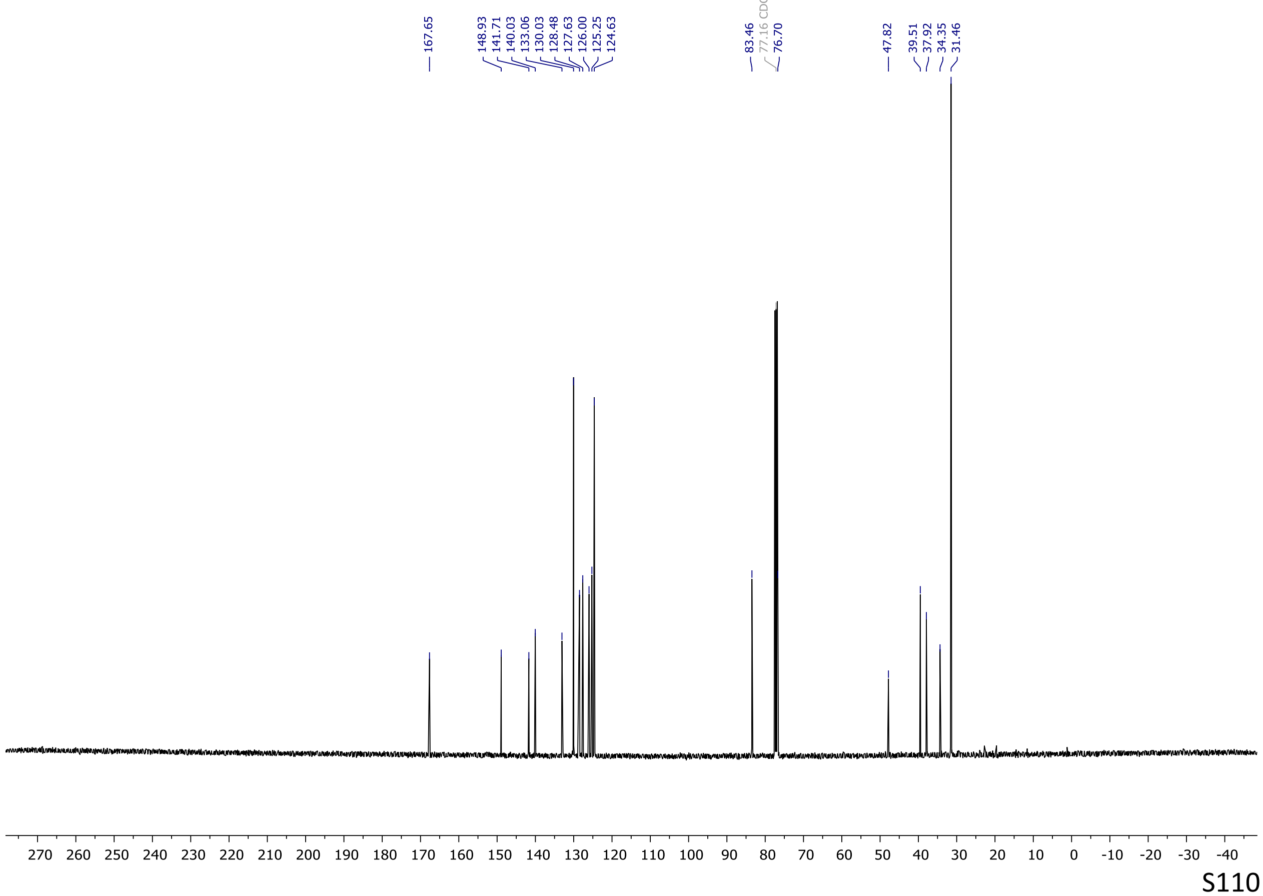
167.65
148.93
141.71
140.03
133.06
130.03
128.48
127.63
126.00
125.25
124.63
83.46
77.16 CDCl3
76.70
47.82
39.51
37.92
34.35
31.46
270 260 250 240 230 220 210 200 190 180 170 160 150 140 130 120 110 100 90 80 70 60 50 40 30 20 10 0 -10 -20 -30 -40

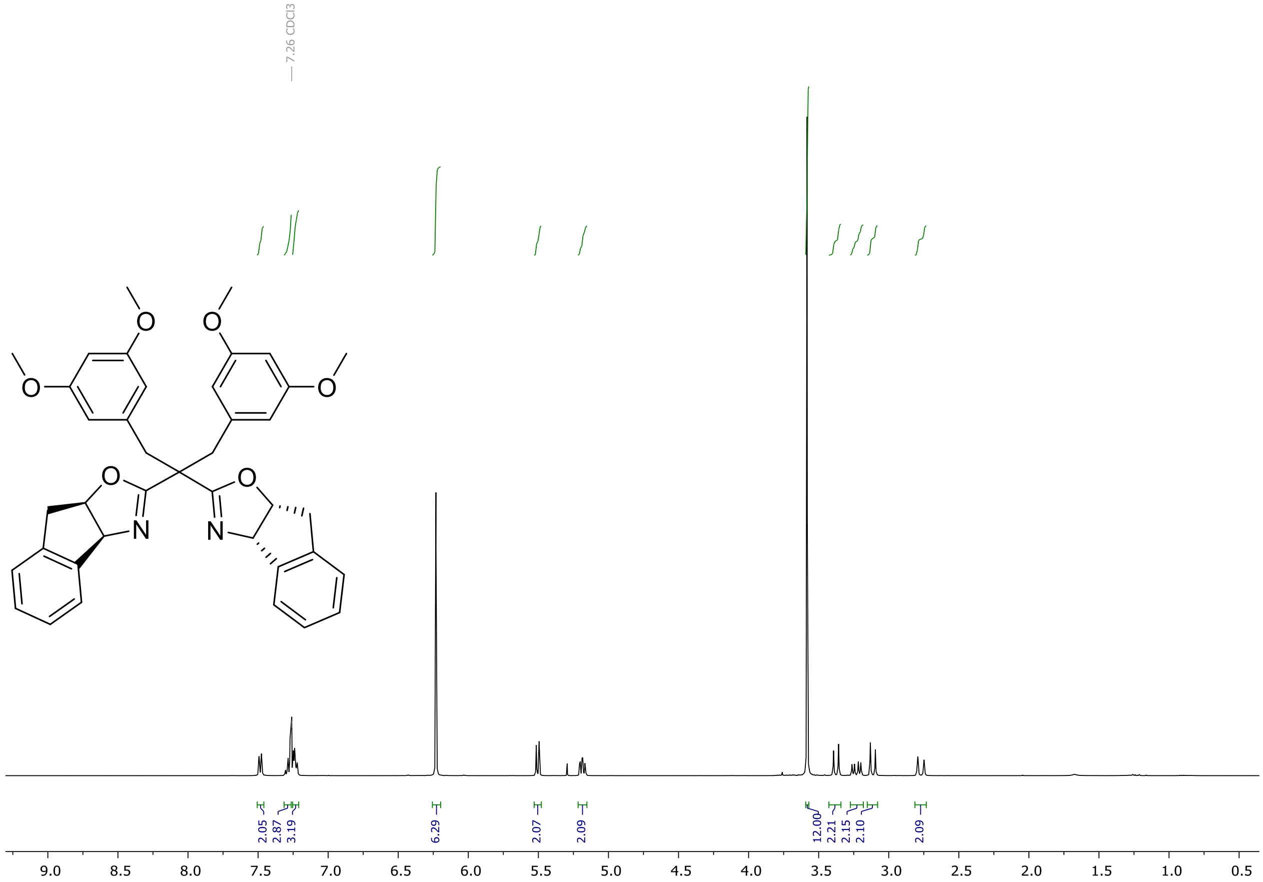
7.26 CDCl3
O
O
O
O
O
N
O
N
2.05
2.87
3.19
6.29
2.07
2.09
12.00
2.21
2.15
2.10
2.09
9.0 8.5 8.0 7.5 7.0 6.5 6.0 5.5 5.0 4.5 4.0 3.5 3.0 2.5 2.0 1.5 1.0 0.5
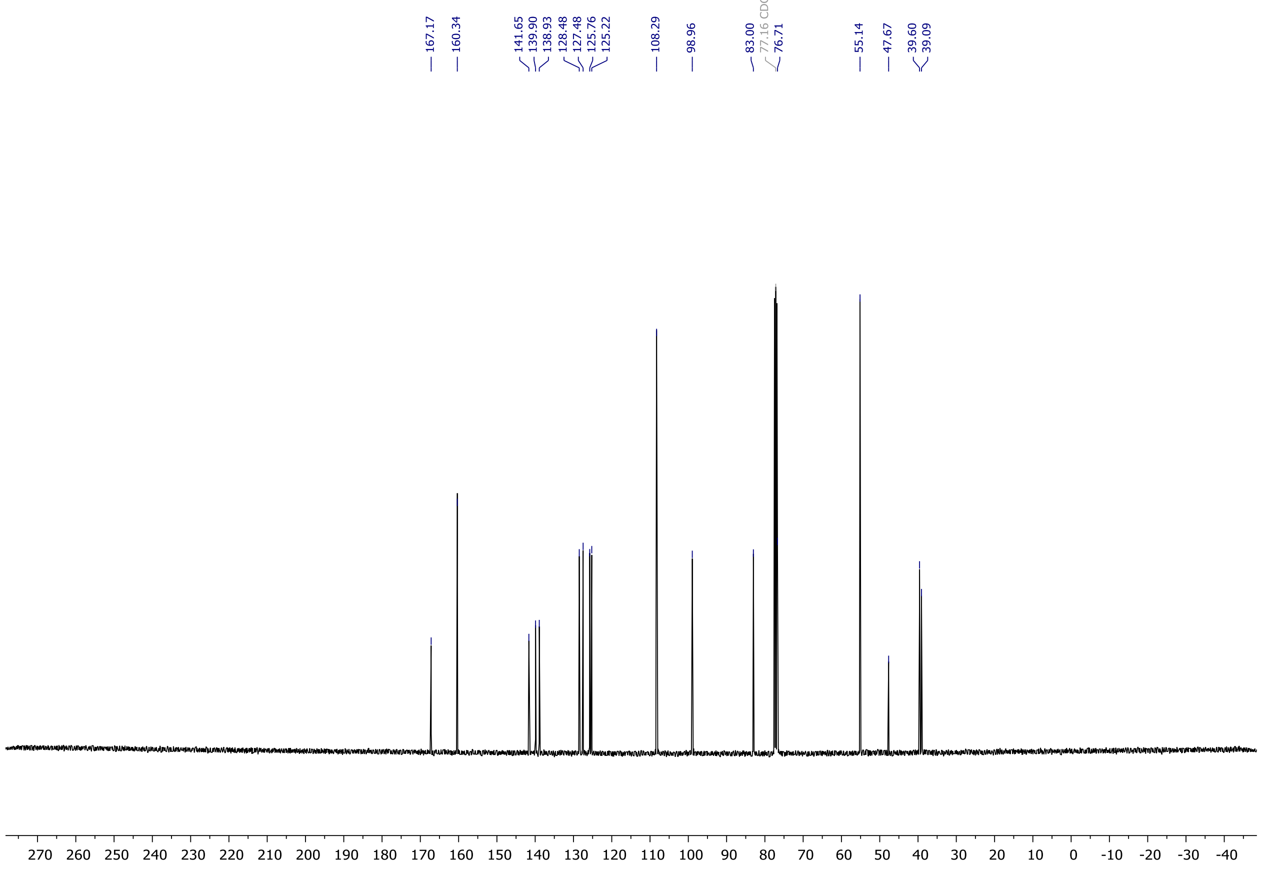
167.17
160.34
141.65
139.90
138.93
128.48
127.48
125.76
125.22
108.29
98.96
83.00
77.16 CDCl3
76.71
55.14
47.67
39.60
39.09
270 260 250 240 230 220 210 200 190 180 170 160 150 140 130 120 110 100 90 80 70 60 50 40 30 20 10 0 -10 -20 -30 -40

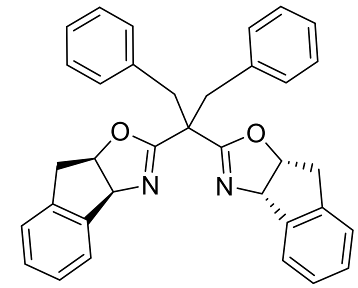

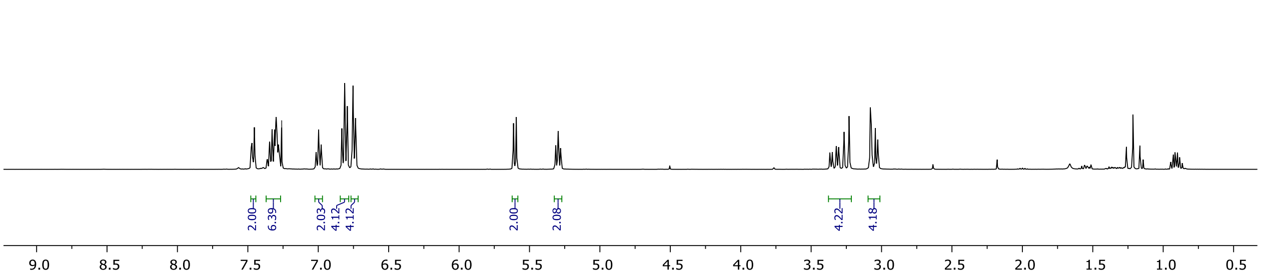

7.26 CDCl3
2.00
6.39
2.03
4.12
4.12
2.00
2.08
4.22
4.18
9.0 8.5 8.0 7.5 7.0 6.5 6.0 5.5 5.0 4.5 4.0 3.5 3.0 2.5 2.0 1.5 1.0 0.5

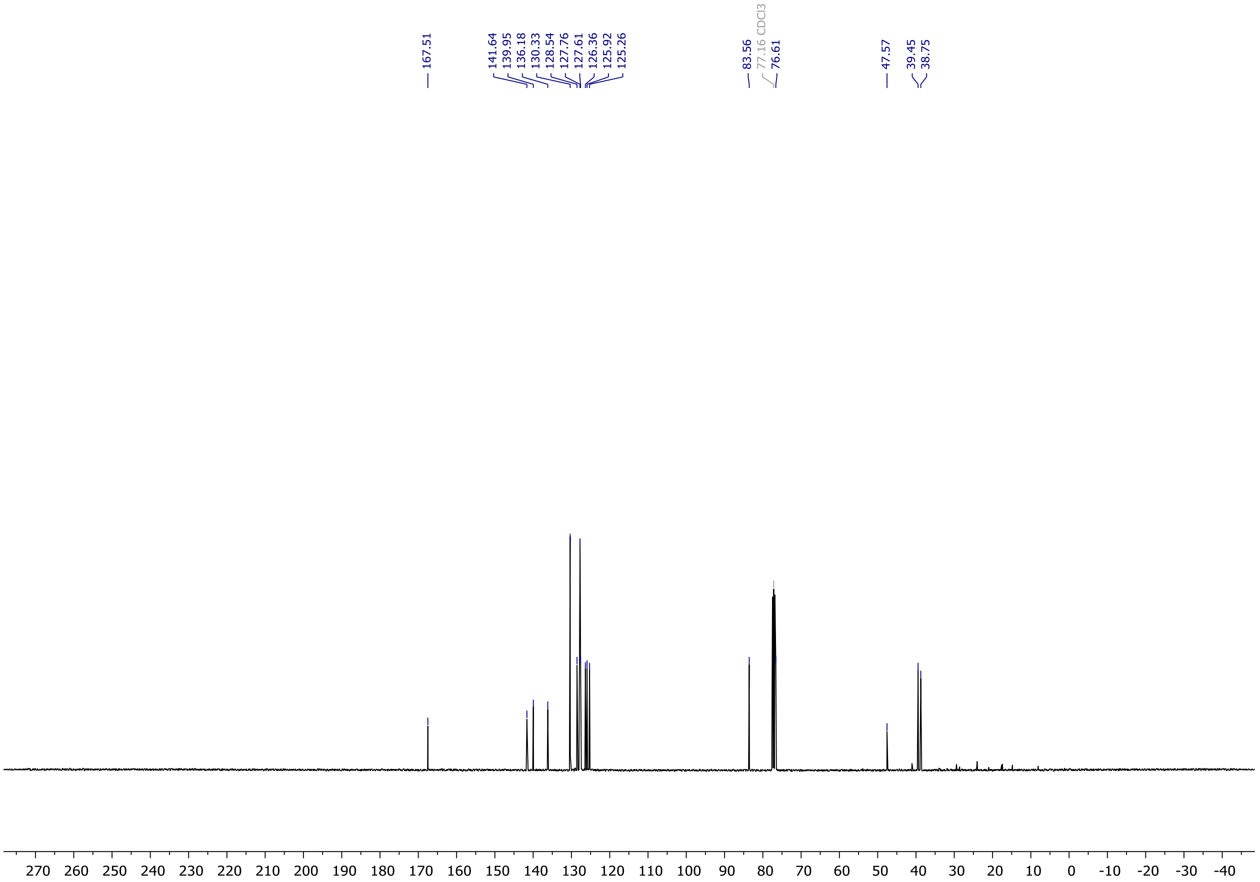

167.51
141.64
139.95
136.18
130.33
128.54
127.76
127.61
126.36
125.92
125.26
83.56
77.16 CDCl3
76.61
47.57
39.45
38.75
270 260 250 240 230 220 210 200 190 180 170 160 150 140 130 120 110 100 90 80 70 60 50 40 30 20 10 0 -10 -20 -30 -40

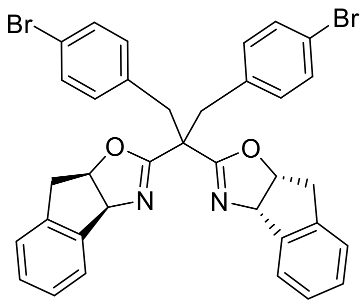

Br
Br
O
N
O
N

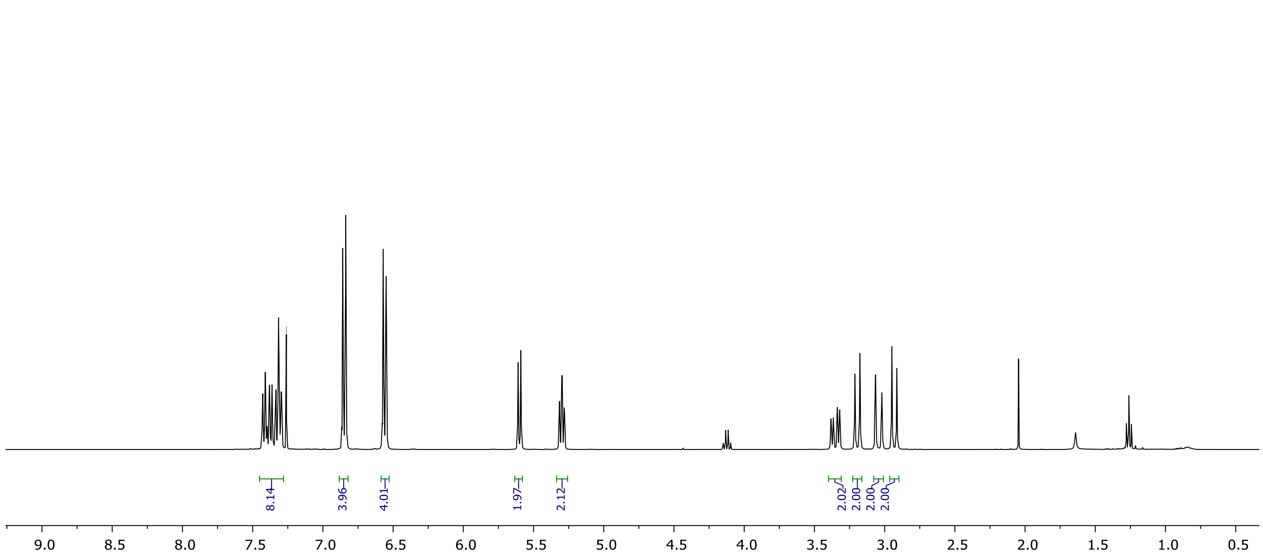

7.26 CDCl3
8.14
3.96
4.01
1.97
2.12
2.02
2.00
2.00
2.00
9.0 8.5 8.0 7.5 7.0 6.5 6.0 5.5 5.0 4.5 4.0 3.5 3.0 2.5 2.0 1.5 1.0 0.5

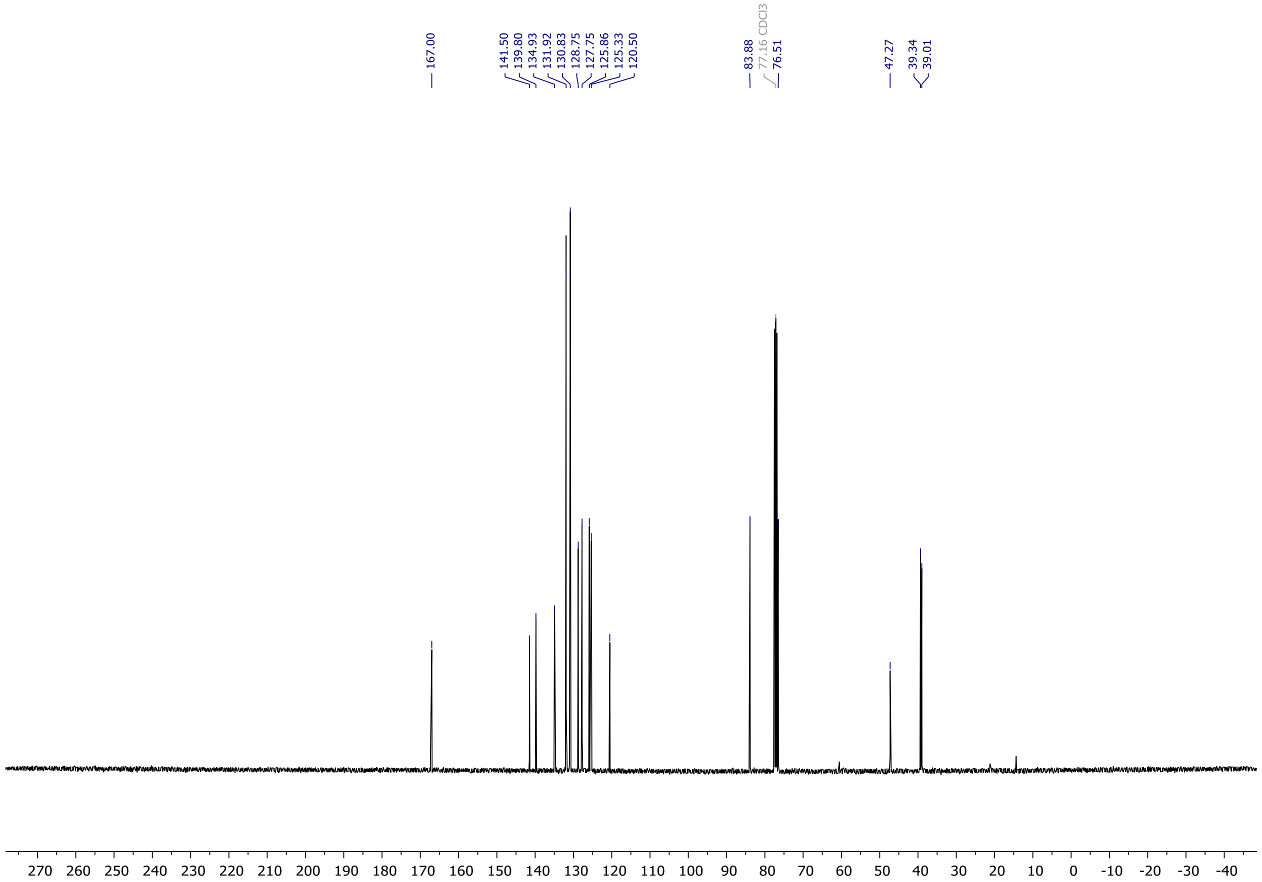

167.00
141.50
139.80
134.93
131.92
130.83
128.75
127.75
125.86
125.33
120.50
83.88
77.16 CDCl3
76.51
47.27
39.34
39.01
270 260 250 240 230 220 210 200 190 180 170 160 150 140 130 120 110 100 90 80 70 60 50 40 30 20 10 0 -10 -20 -30 -40

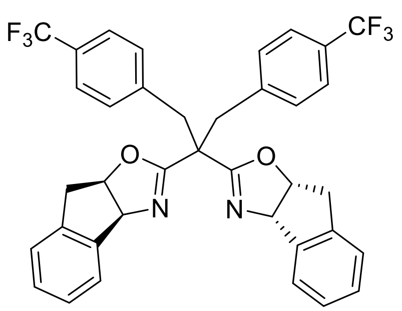

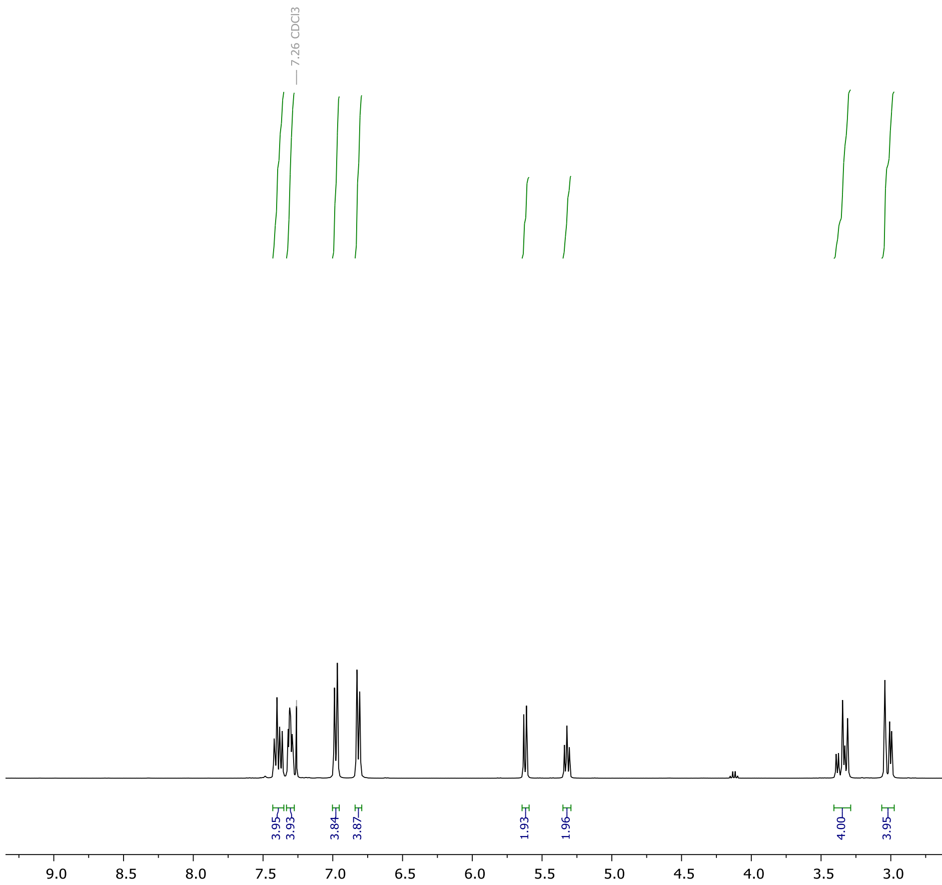

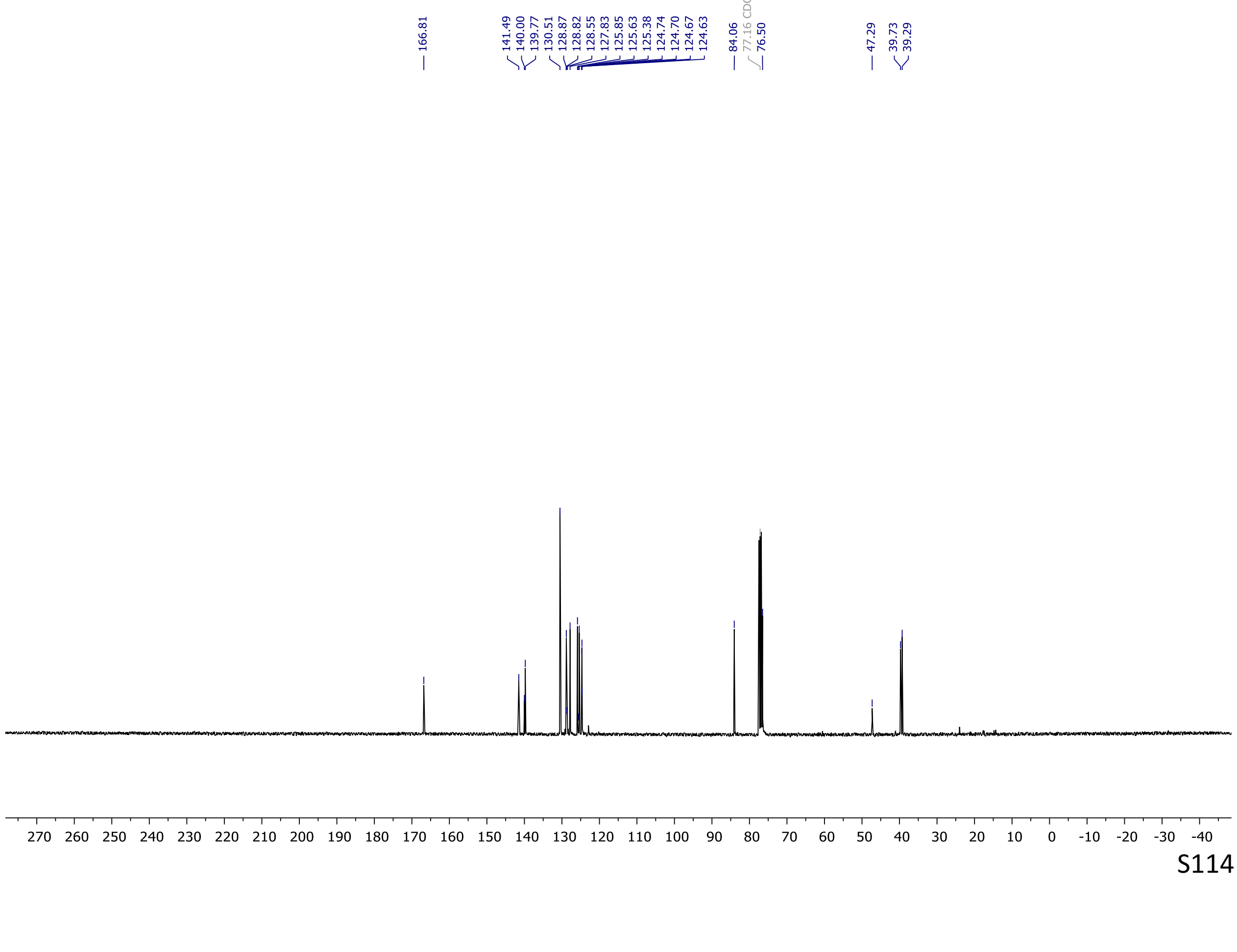

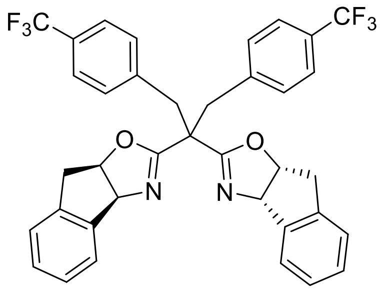

^{19}F NMR

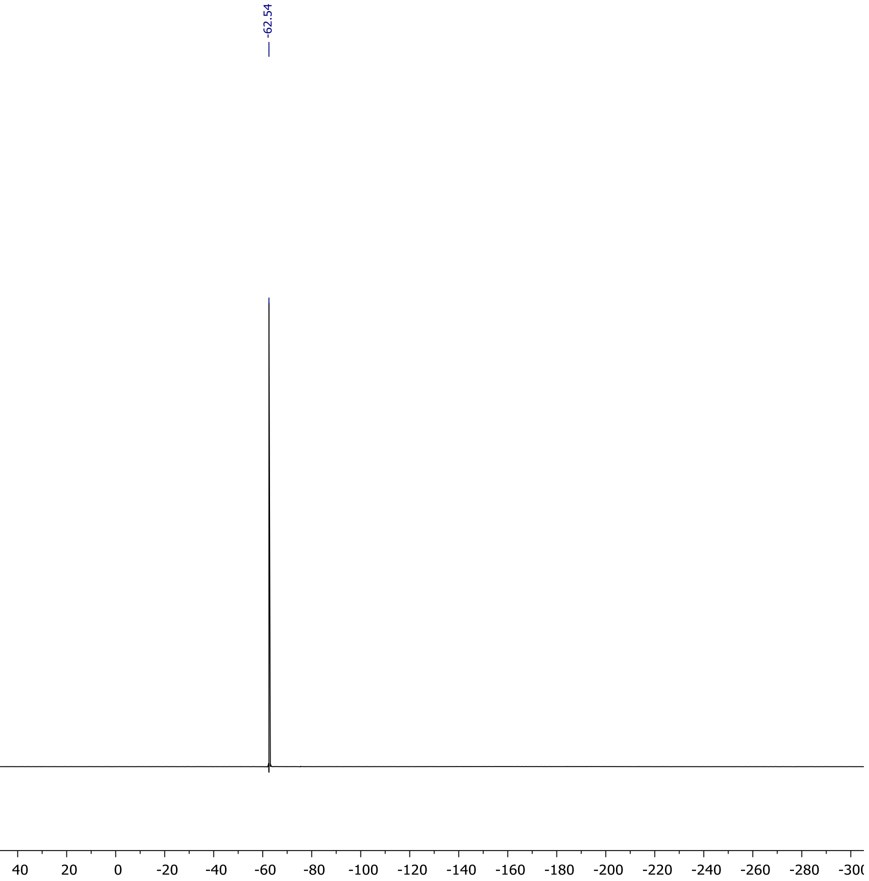

-62.54
200 180 160 140 120 100 80 60 40 20 0 -20 -40 -60 -80 -100 -120 -140 -160 -180 -200 -220 -240 -260 -280 -300

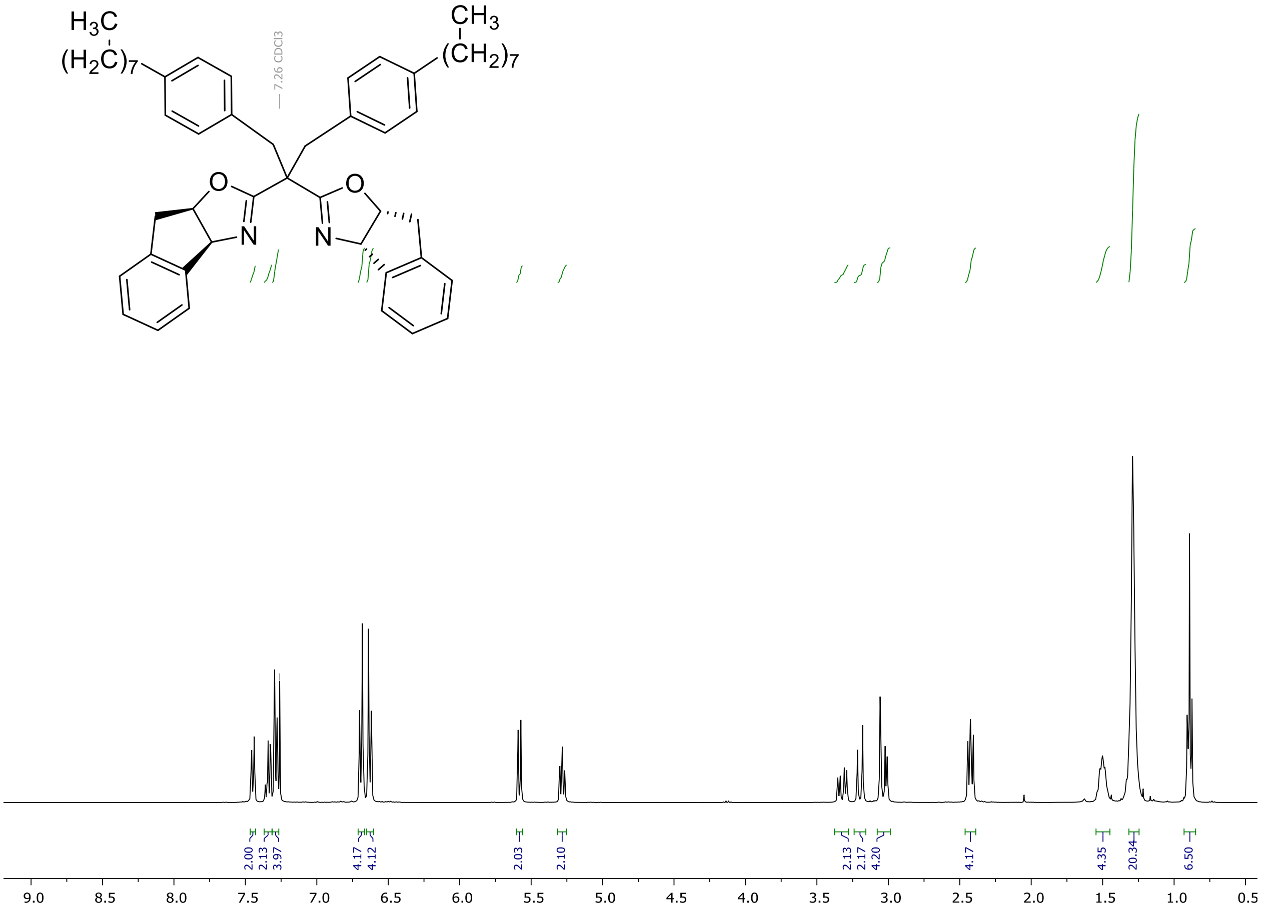

7.26 CDCl3
2.00
2.13
3.97
4.17
4.12
2.03
2.10
2.13
2.17
4.20
4.17
4.35
20.34
6.50
9.0 8.5 8.0 7.5 7.0 6.5 6.0 5.5 5.0 4.5 4.0 3.5 3.0 2.5 2.0 1.5 1.0 0.5

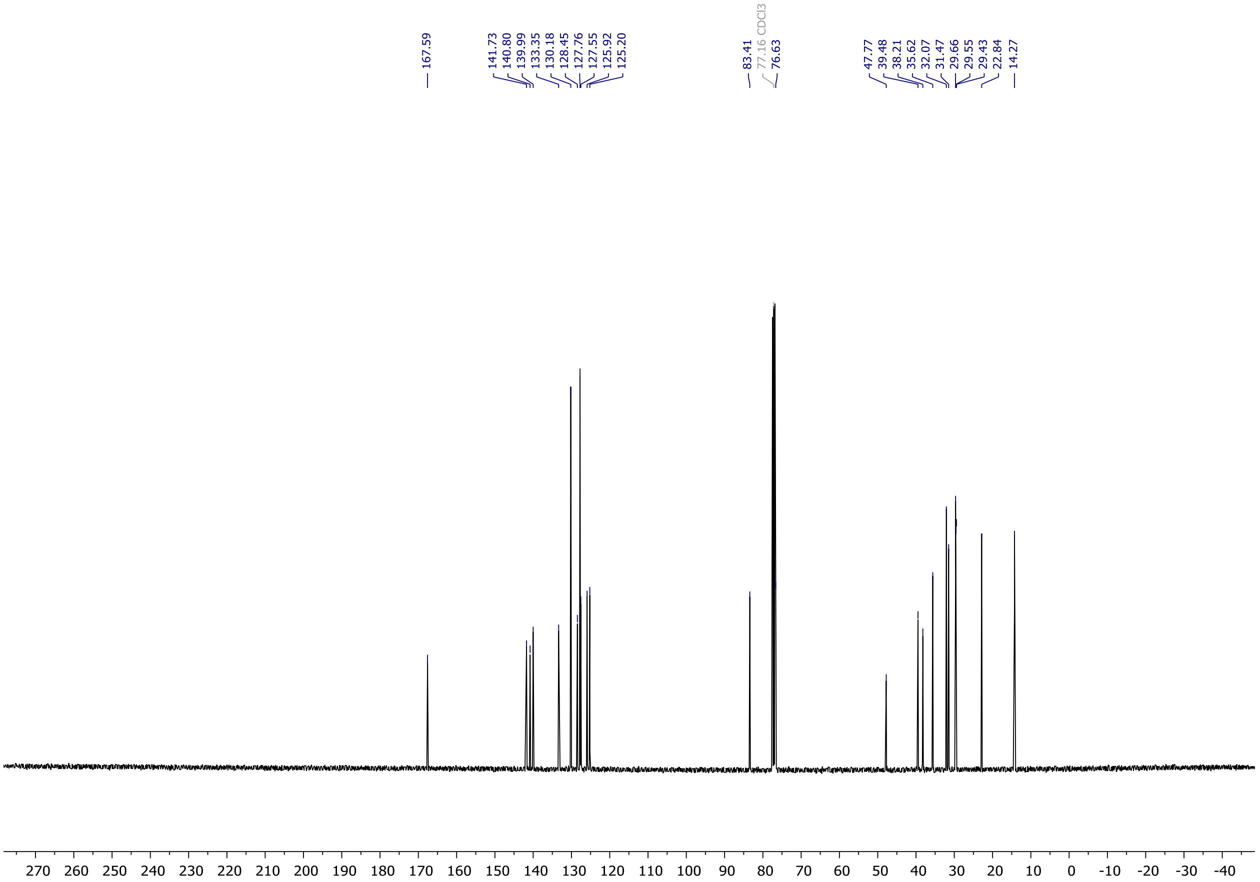

167.59
141.73
140.80
139.99
133.35
130.18
128.45
127.76
127.55
125.92
125.20
83.41
77.16 CDCl3
76.63
47.77
39.48
38.21
35.62
32.07
31.47
29.66
29.55
29.43
22.84
14.27
270 260 250 240 230 220 210 200 190 180 170 160 150 140 130 120 110 100 90 80 70 60 50 40 30 20 10 0 -10 -20 -30 -40

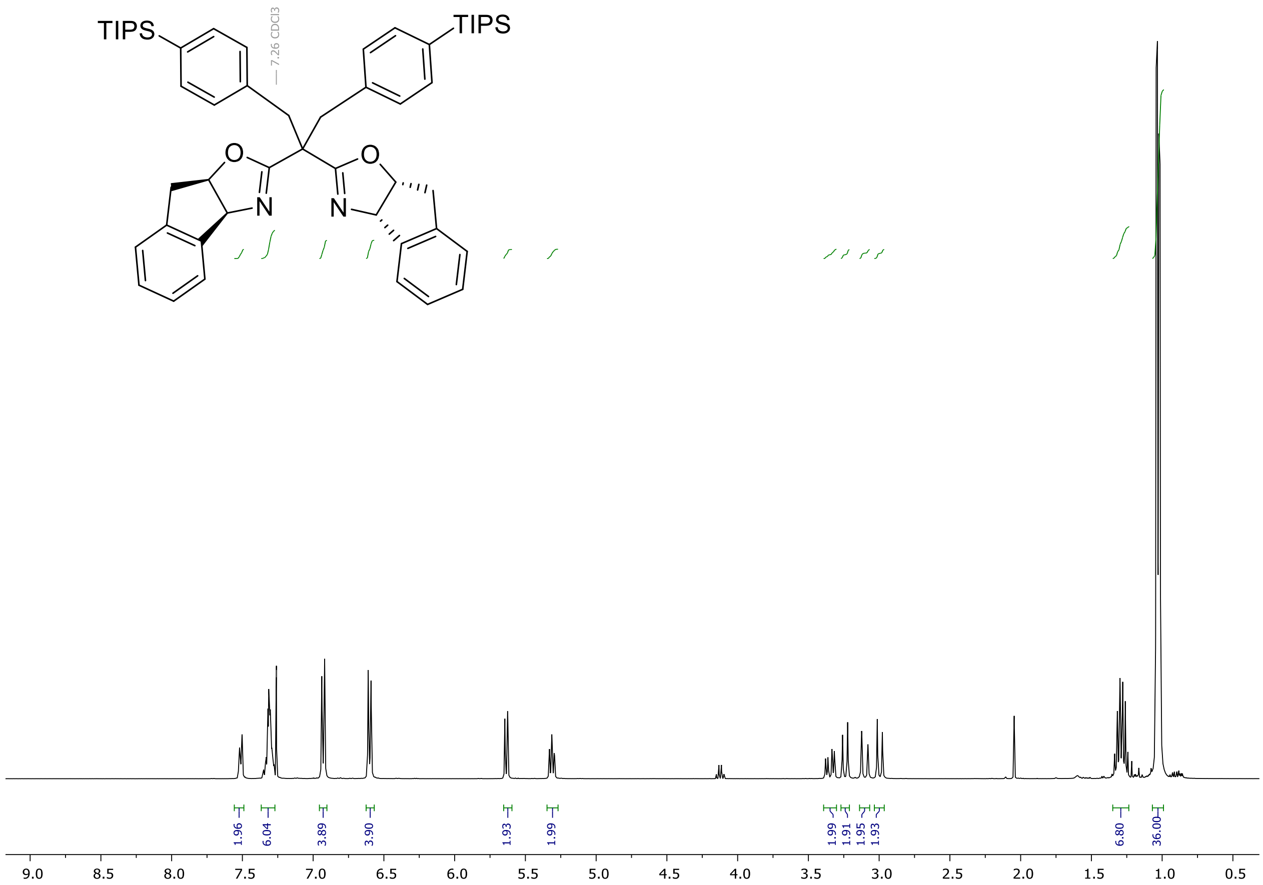

TIPS
TIPS
O
N
O
N
7.26 CDCl3
1.96
6.04
3.89
3.90
1.93
1.99
1.99
1.91
1.95
1.93
6.80
36.00
9.0 8.5 8.0 7.5 7.0 6.5 6.0 5.5 5.0 4.5 4.0 3.5 3.0 2.5 2.0 1.5 1.0 0.5

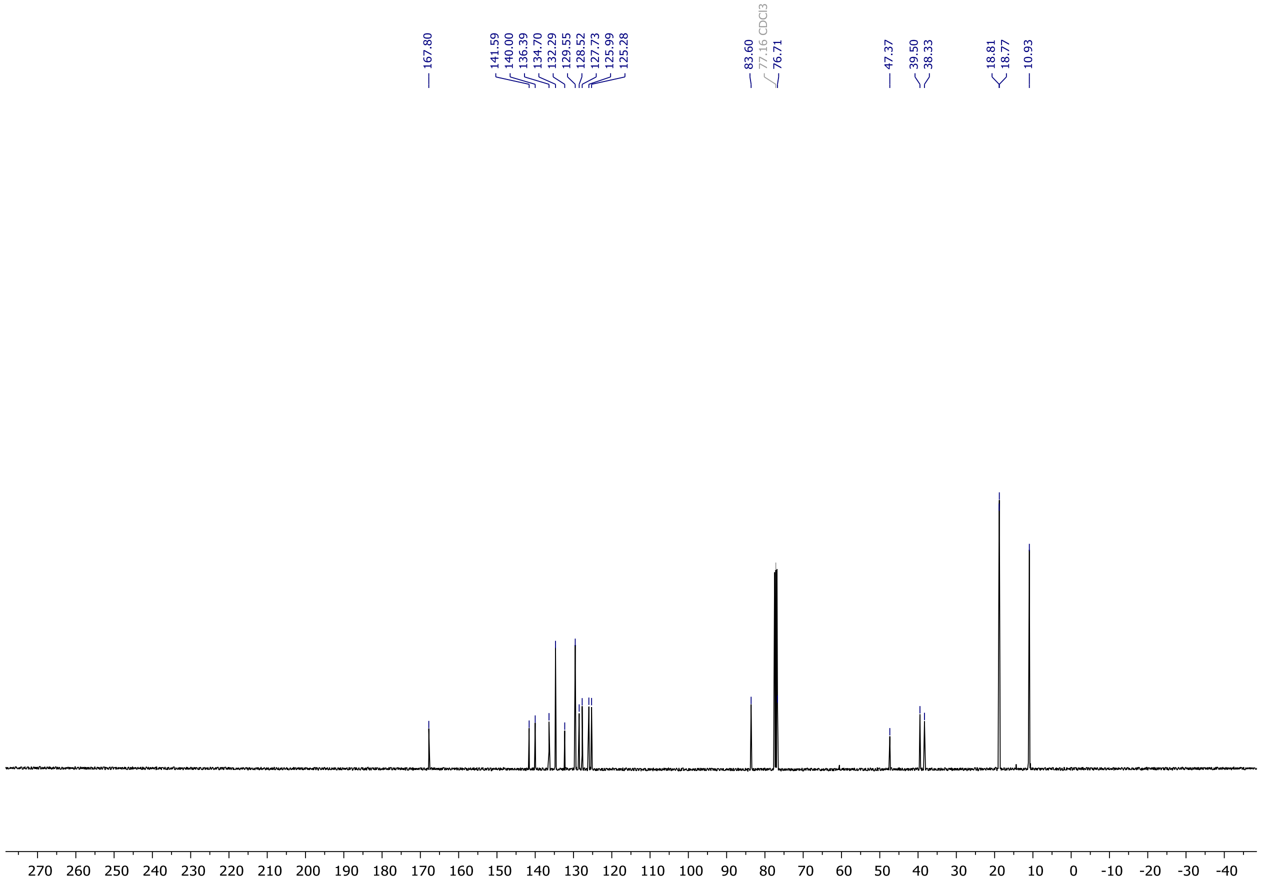

167.80
141.59
140.00
136.39
134.70
132.29
129.55
128.52
127.73
125.99
125.28
83.60
77.16 CDCl3
76.71
47.37
39.50
38.33
18.81
18.77
10.93
270 260 250 240 230 220 210 200 190 180 170 160 150 140 130 120 110 100 90 80 70 60 50 40 30 20 10 0 -10 -20 -30 -40

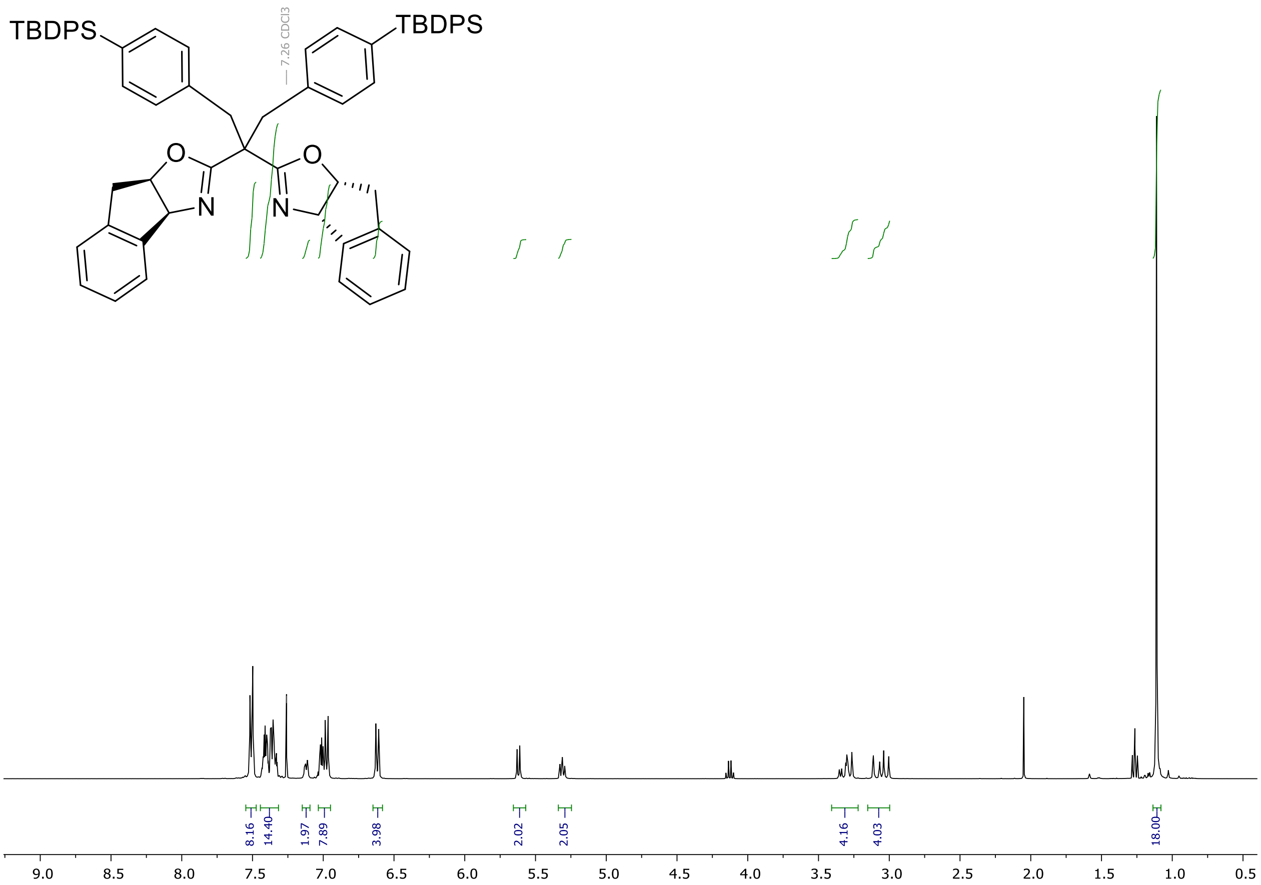
TBDPS
TBDPS
O
N
O
N
7.26 CDCl3
8.16
14.40
1.97
7.89
3.98
2.02
2.05
4.16
4.03
18.00
9.0 8.5 8.0 7.5 7.0 6.5 6.0 5.5 5.0 4.5 4.0 3.5 3.0 2.5 2.0 1.5 1.0 0.5

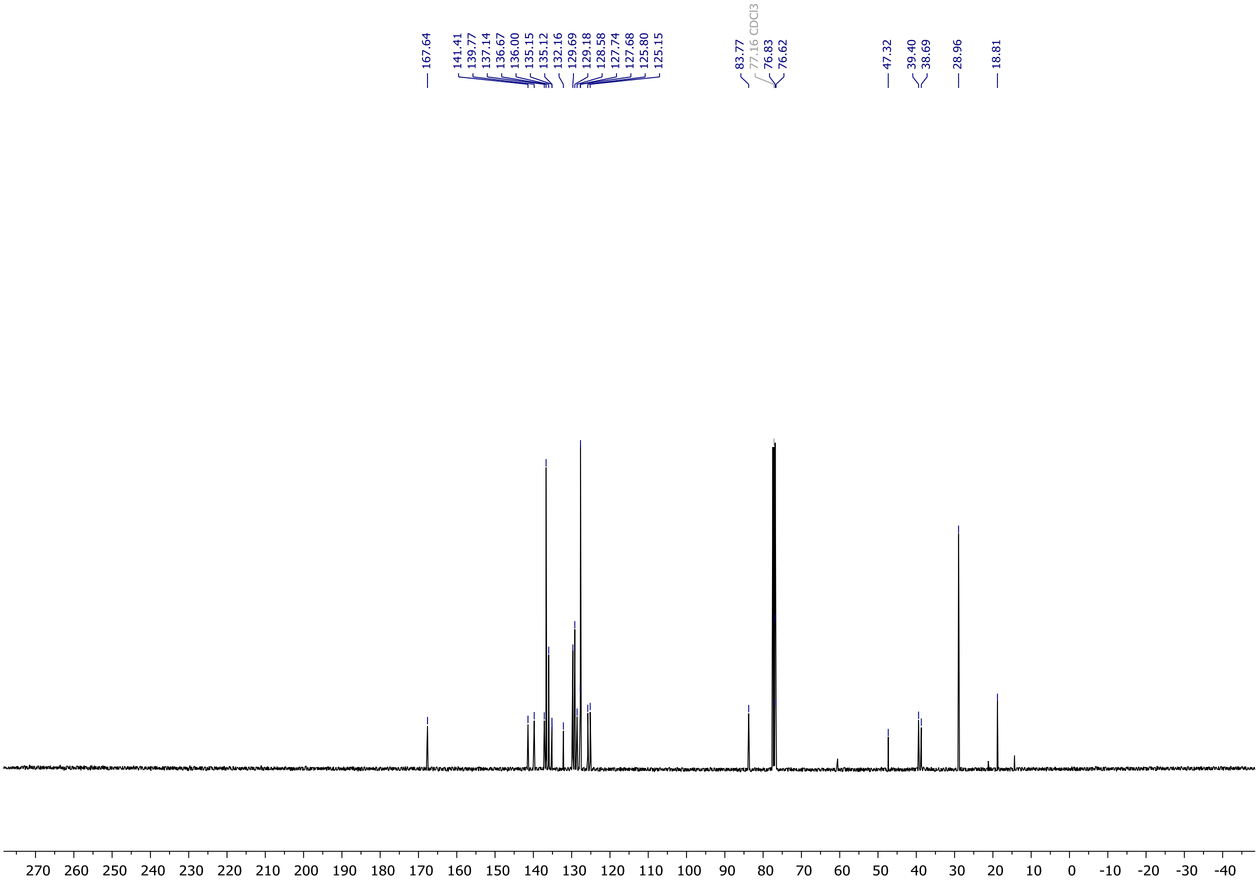
167.64
141.41
139.77
137.14
136.67
136.00
135.15
135.12
132.16
129.69
129.18
128.58
127.74
127.68
125.80
125.15
83.77
77.16 CDCl3
76.83
76.62
47.32
39.40
38.69
28.96
18.81
270 260 250 240 230 220 210 200 190 180 170 160 150 140 130 120 110 100 90 80 70 60 50 40 30 20 10 0 -10 -20 -30 -40

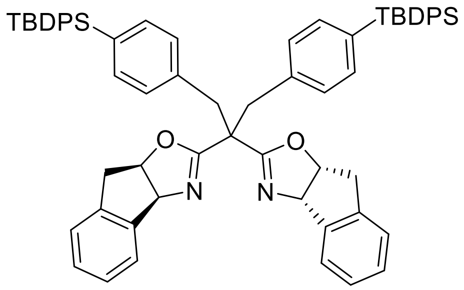
TBDPS
TBDPS
O
N
O
N

^{29}Si NMR

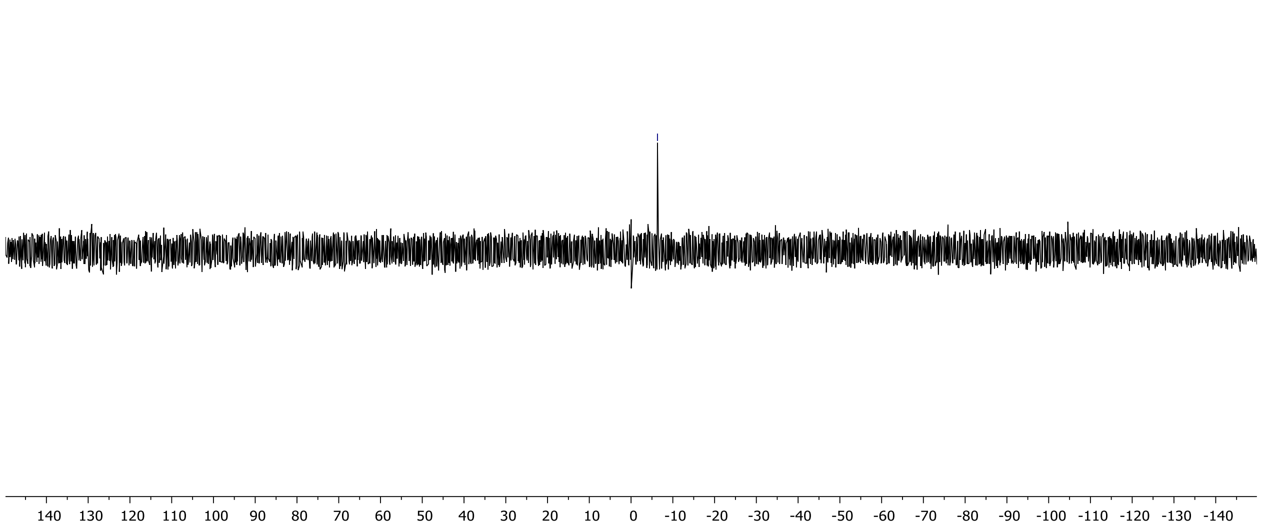
-6.32
140 130 120 110 100 90 80 70 60 50 40 30 20 10 0 -10 -20 -30 -40 -50 -60 -70 -80 -90 -100 -110 -120 -130 -140

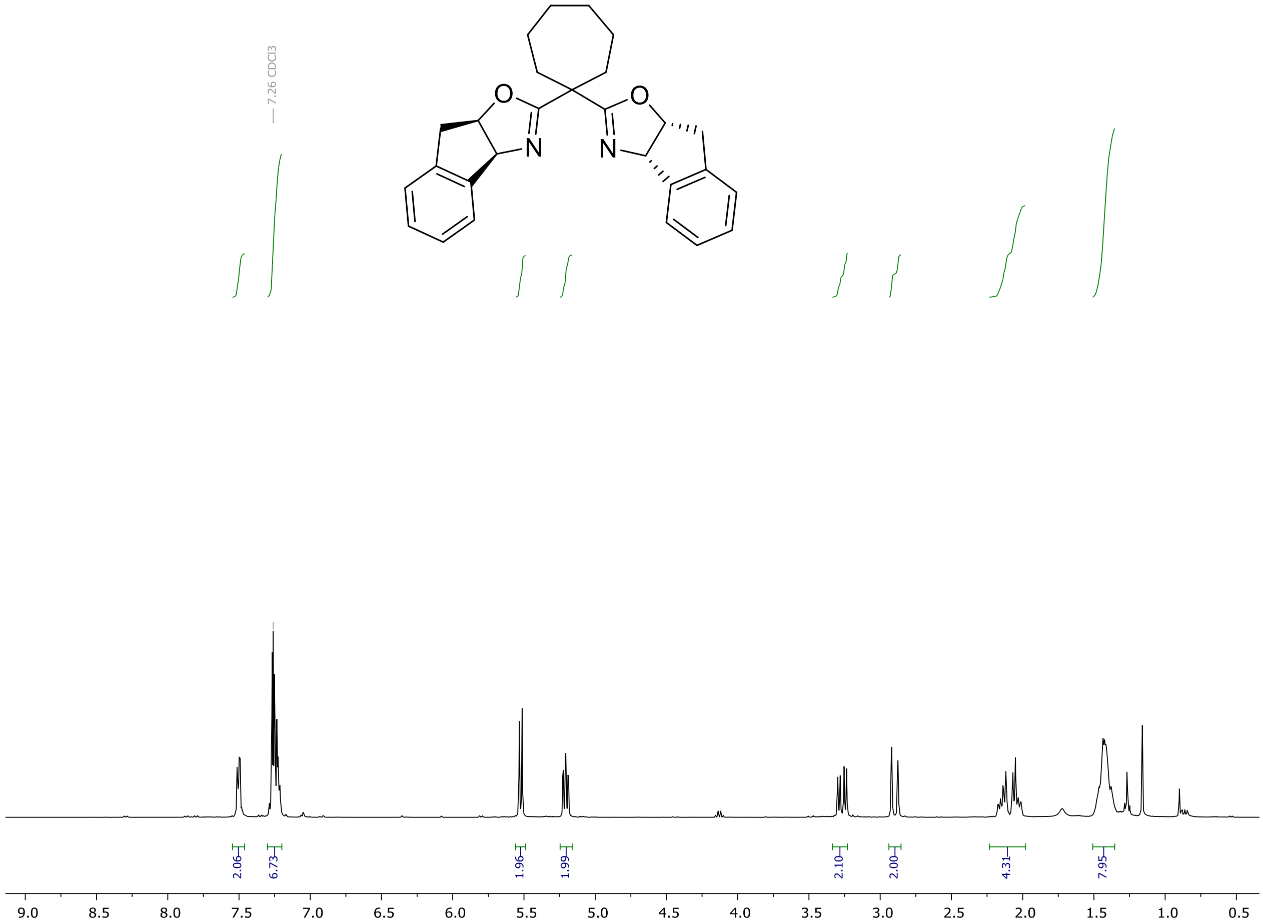

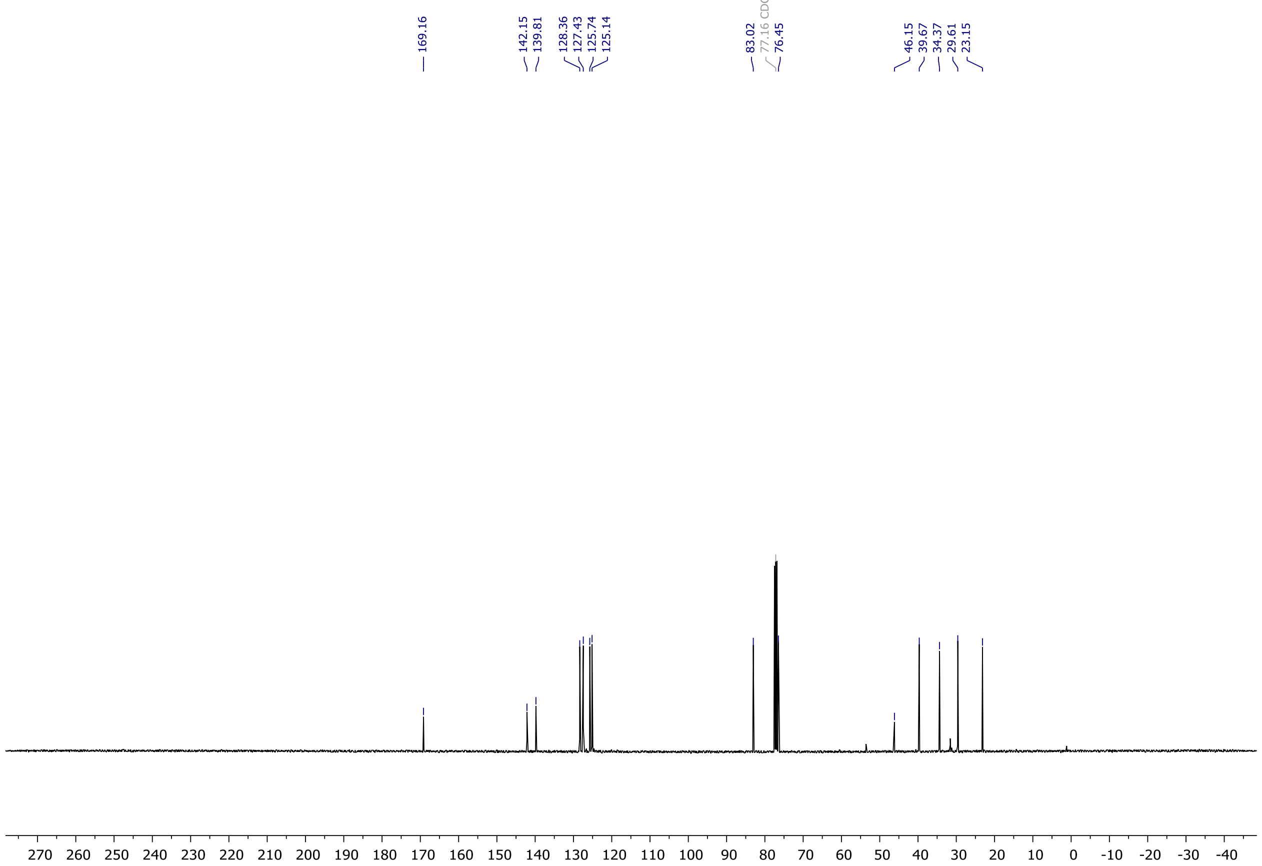

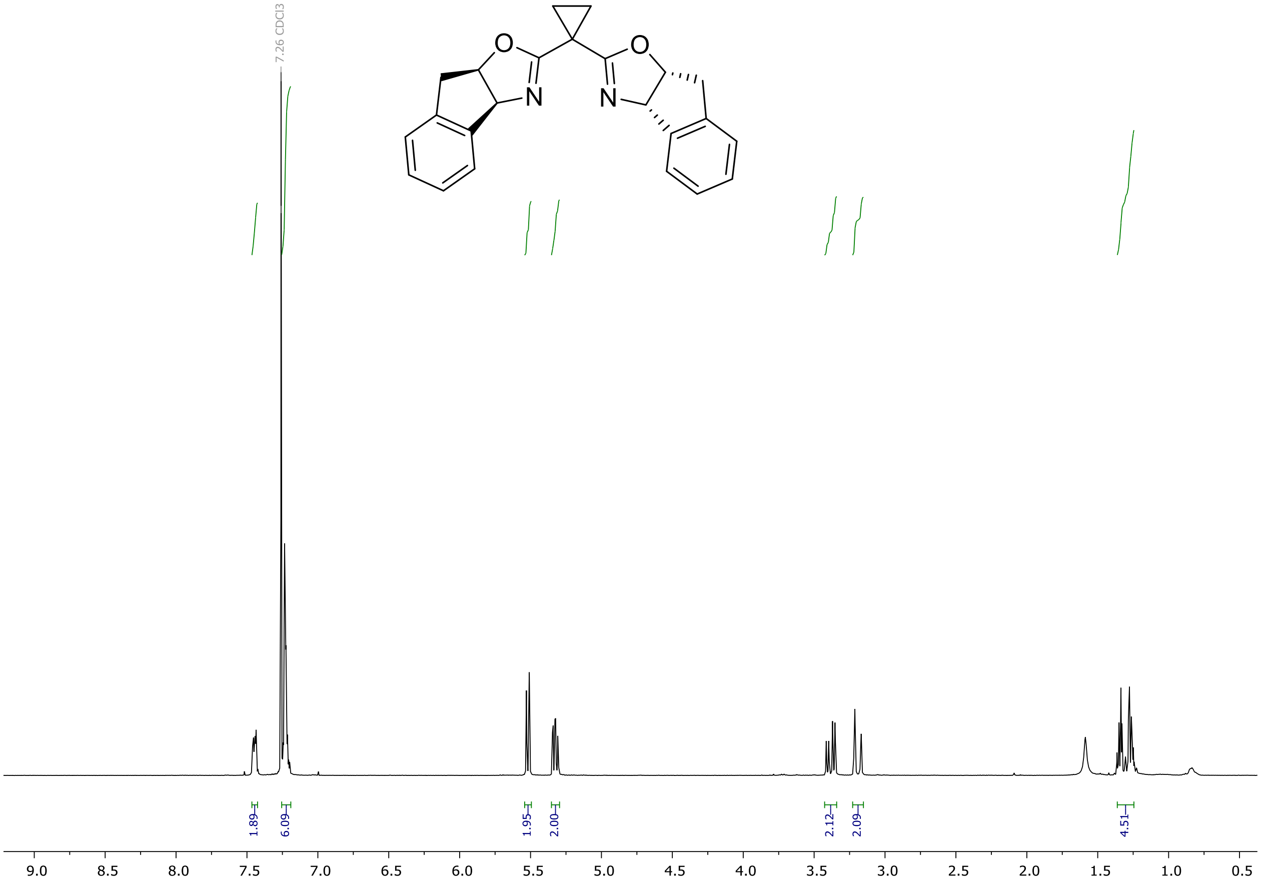
7.26 CDCl3
1.89
6.09
1.95
2.00
2.12
2.09
4.51
9.0 8.5 8.0 7.5 7.0 6.5 6.0 5.5 5.0 4.5 4.0 3.5 3.0 2.5 2.0 1.5 1.0 0.5

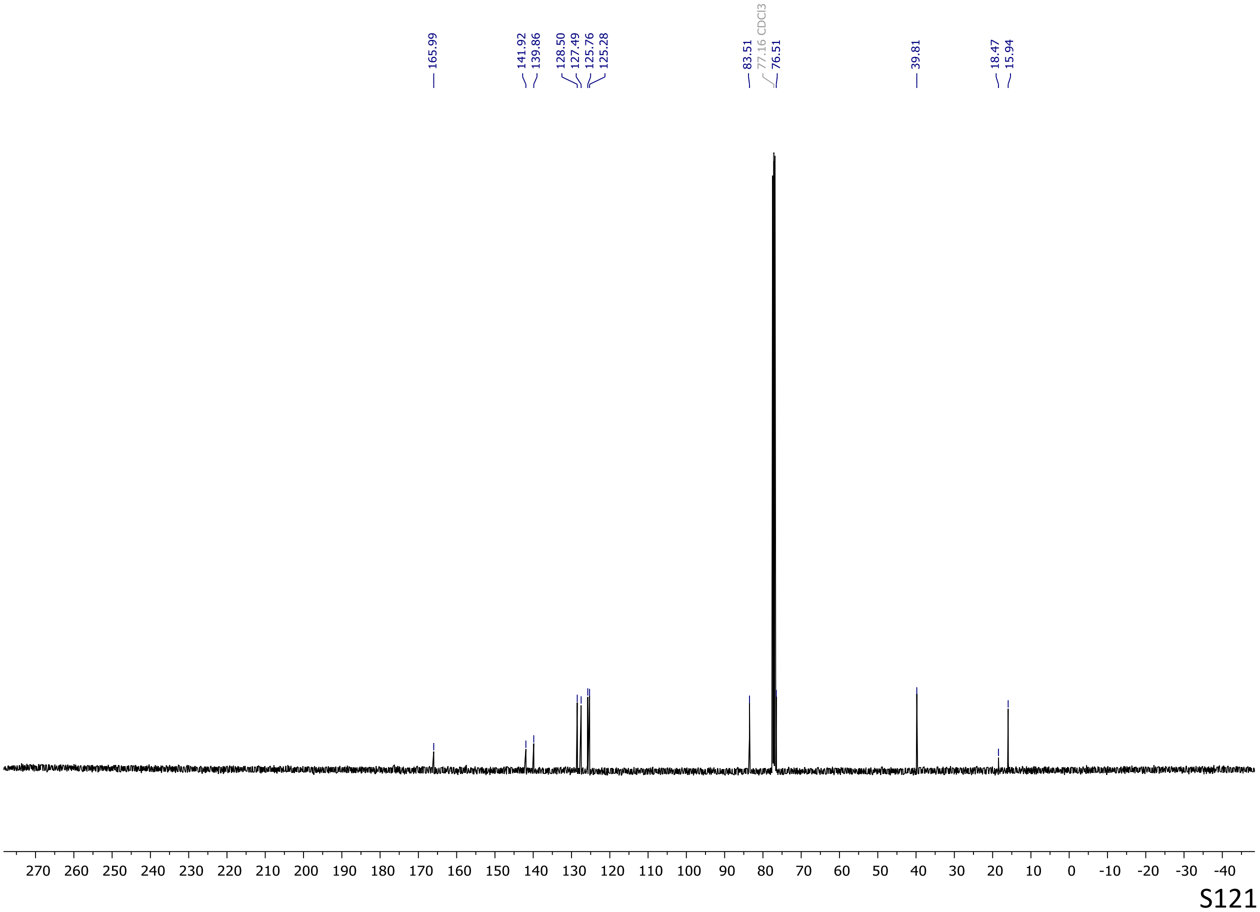
165.99
141.92
139.86
128.50
127.49
125.76
125.28
83.51
77.16 CDCl3
76.51
39.81
18.47
15.94
270 260 250 240 230 220 210 200 190 180 170 160 150 140 130 120 110 100 90 80 70 60 50 40 30 20 10 0 -10 -20 -30 -40

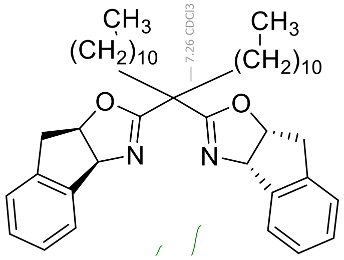

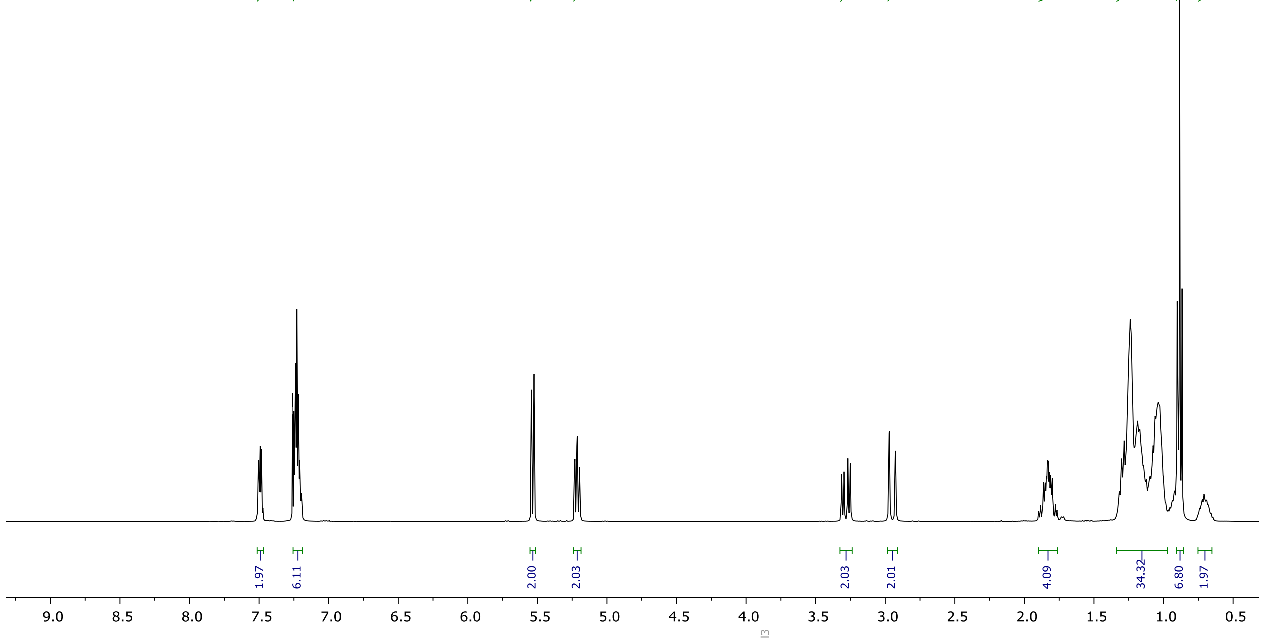

7.26 CDCl3
1.97
6.11
2.00
2.03
2.03
2.01
4.09
34.32
6.80
1.97
9.0 8.5 8.0 7.5 7.0 6.5 6.0 5.5 5.0 4.5 4.0 3.5 3.0 2.5 2.0 1.5 1.0 0.5

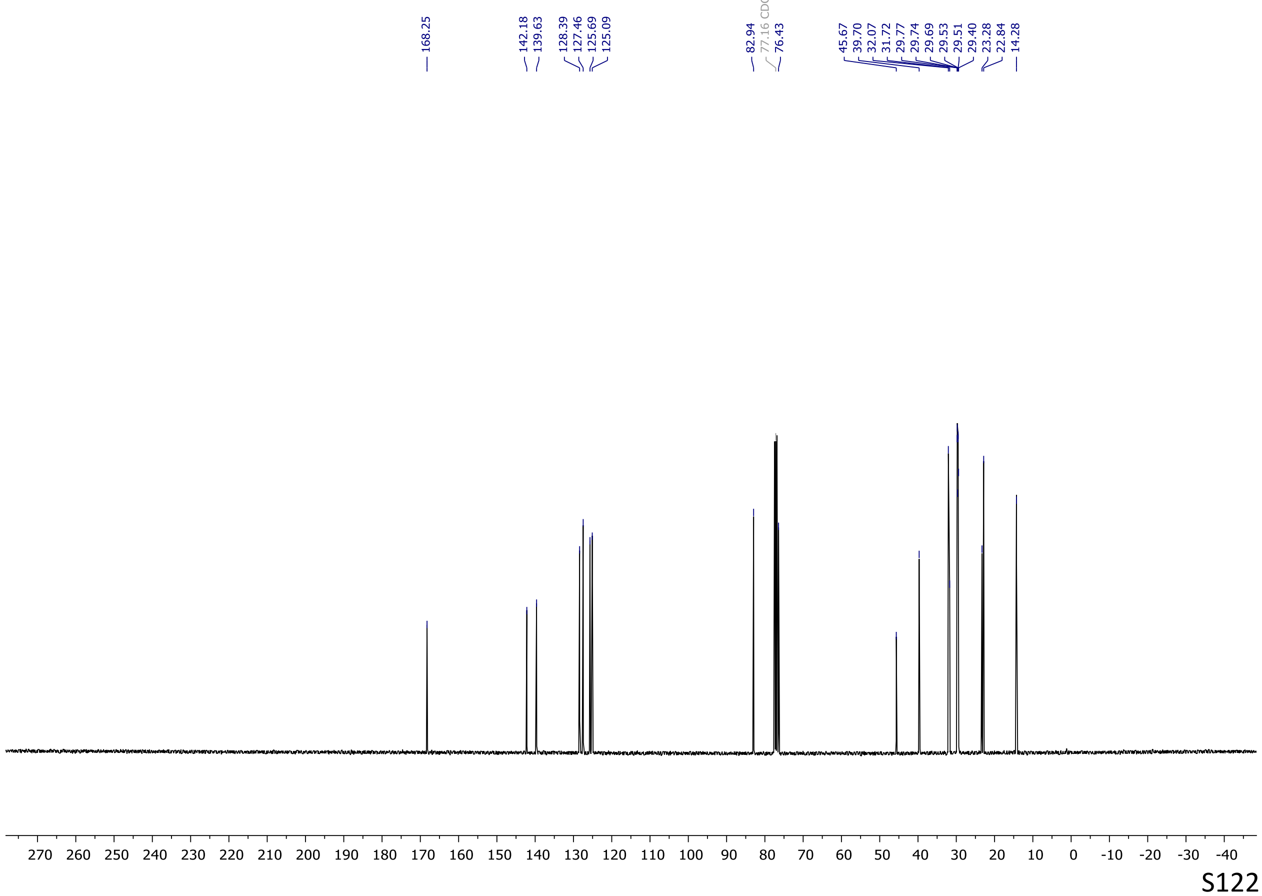

168.25
142.18
139.63
128.39
127.46
125.69
125.09
82.94
77.16 CDCl3
76.43
45.67
39.70
32.07
31.72
29.77
29.74
29.69
29.53
29.51
29.40
23.28
22.84
14.28
270 260 250 240 230 220 210 200 190 180 170 160 150 140 130 120 110 100 90 80 70 60 50 40 30 20 10 0 -10 -20 -30 -40

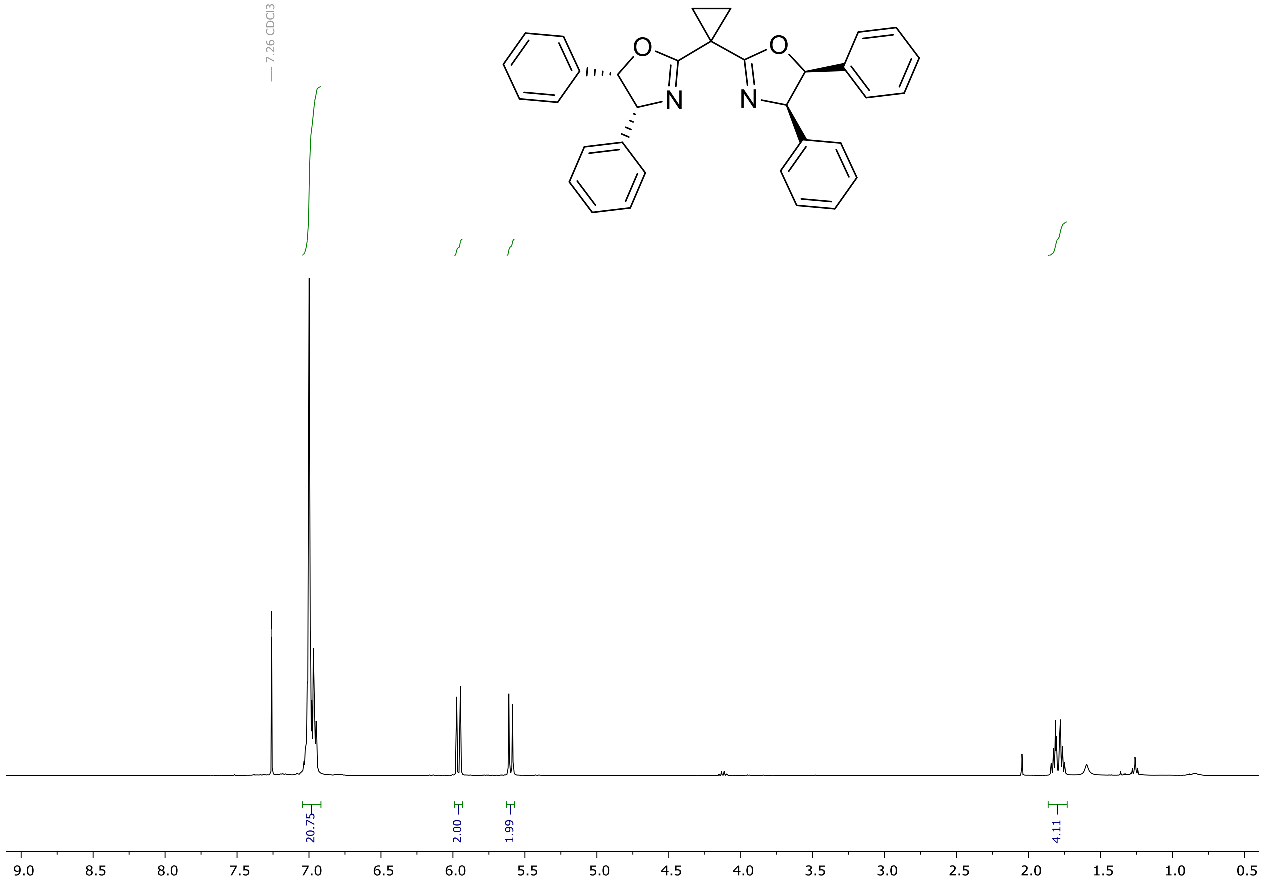
7.26 CDCl3
O
N
20.75
2.00
1.99
4.11
9.0 8.5 8.0 7.5 7.0 6.5 6.0 5.5 5.0 4.5 4.0 3.5 3.0 2.5 2.0 1.5 1.0 0.5

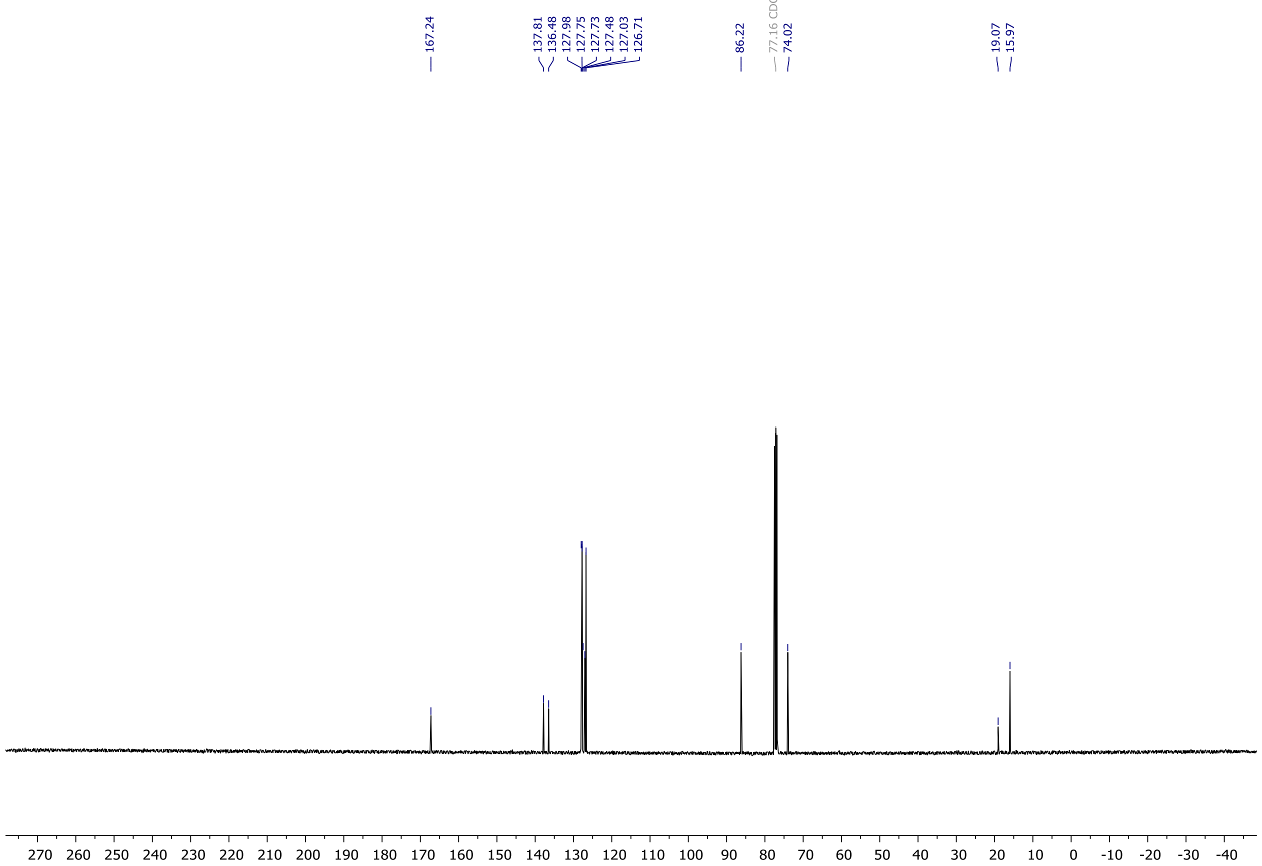
167.24
137.81
136.48
127.98
127.75
127.73
127.48
127.03
126.71
86.22
77.16 CDCl3
74.02
19.07
15.97
270 260 250 240 230 220 210 200 190 180 170 160 150 140 130 120 110 100 90 80 70 60 50 40 30 20 10 0 -10 -20 -30 -40

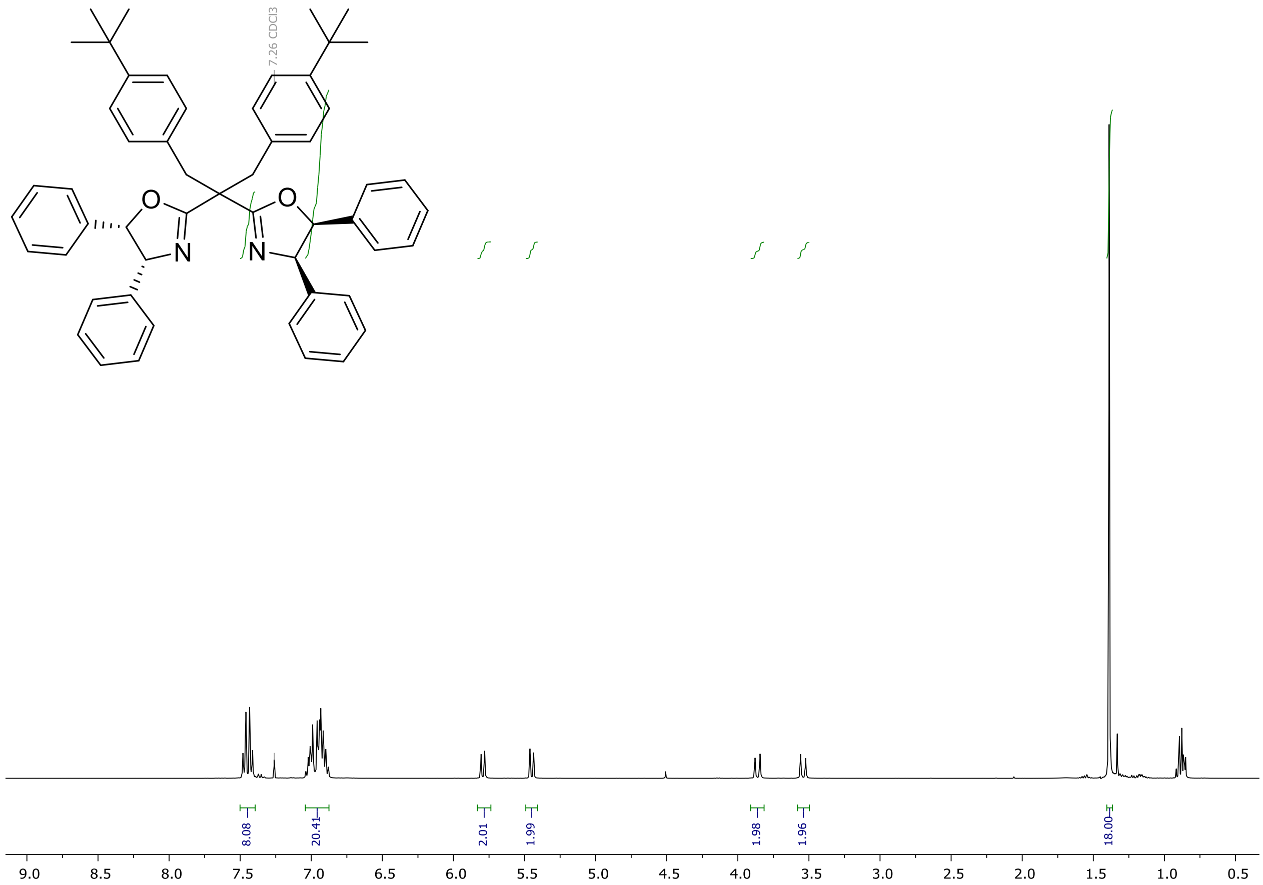
O
N
O
N
7.26 CDCl3
8.08
20.41
2.01
1.99
1.98
1.96
18.00
9.0 8.5 8.0 7.5 7.0 6.5 6.0 5.5 5.0 4.5 4.0 3.5 3.0 2.5 2.0 1.5 1.0 0.5

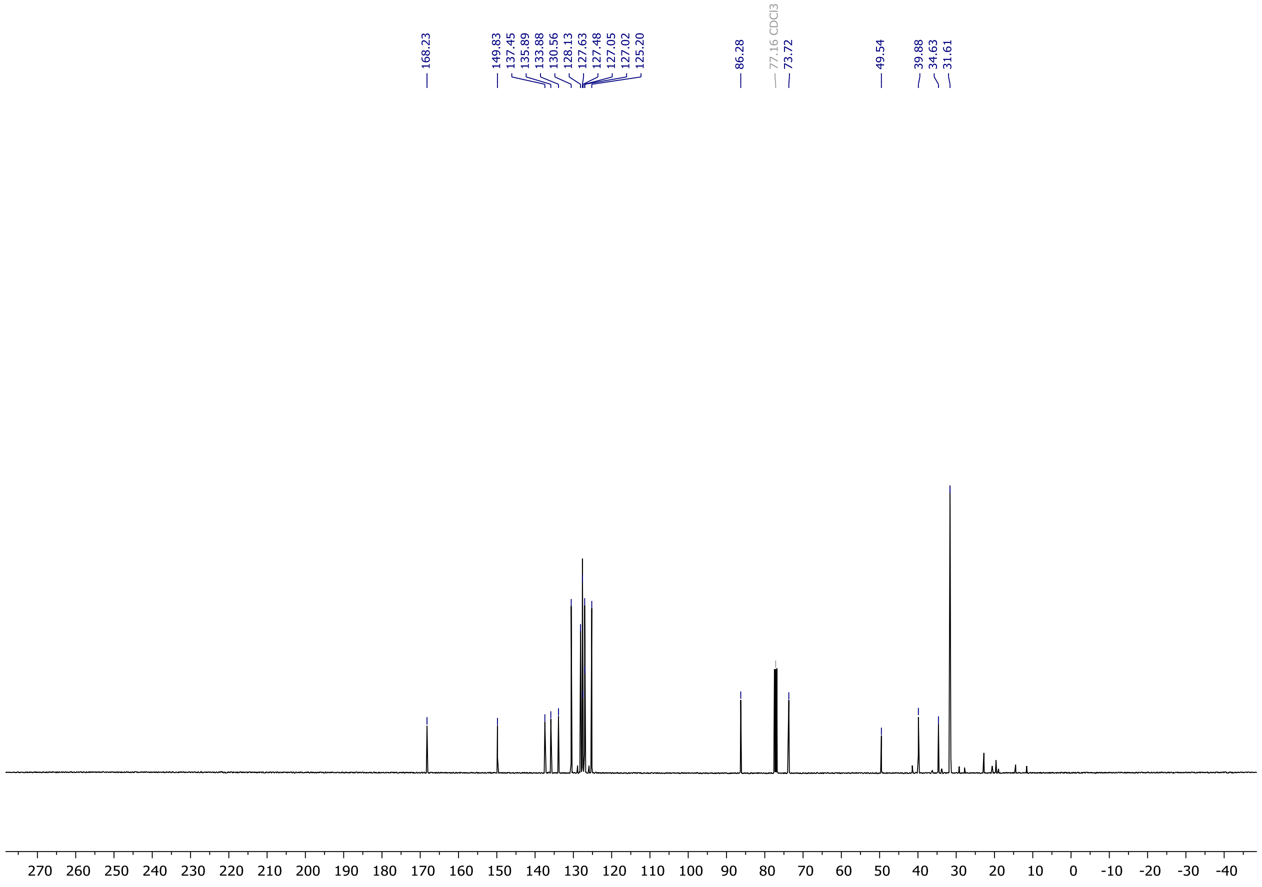
168.23
149.83
137.45
135.89
133.88
130.56
128.13
127.63
127.48
127.05
127.02
125.20
86.28
77.16 CDCl3
73.72
49.54
39.88
34.63
31.61
270 260 250 240 230 220 210 200 190 180 170 160 150 140 130 120 110 100 90 80 70 60 50 40 30 20 10 0 -10 -20 -30 -40

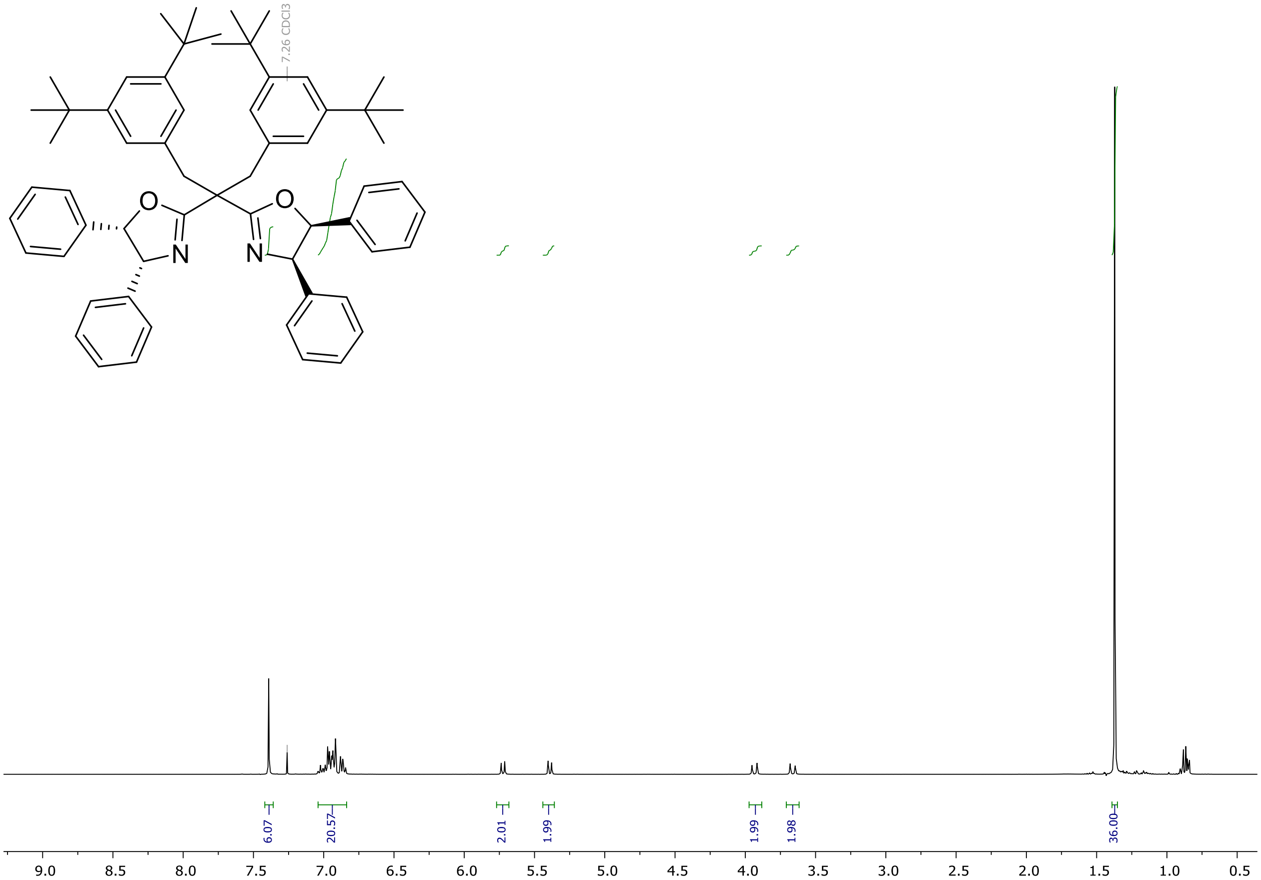

O
N
O
N
7.26 CDCl3
6.07
20.57
2.01
1.99
1.99
1.98
36.00
9.0 8.5 8.0 7.5 7.0 6.5 6.0 5.5 5.0 4.5 4.0 3.5 3.0 2.5 2.0 1.5 1.0 0.5

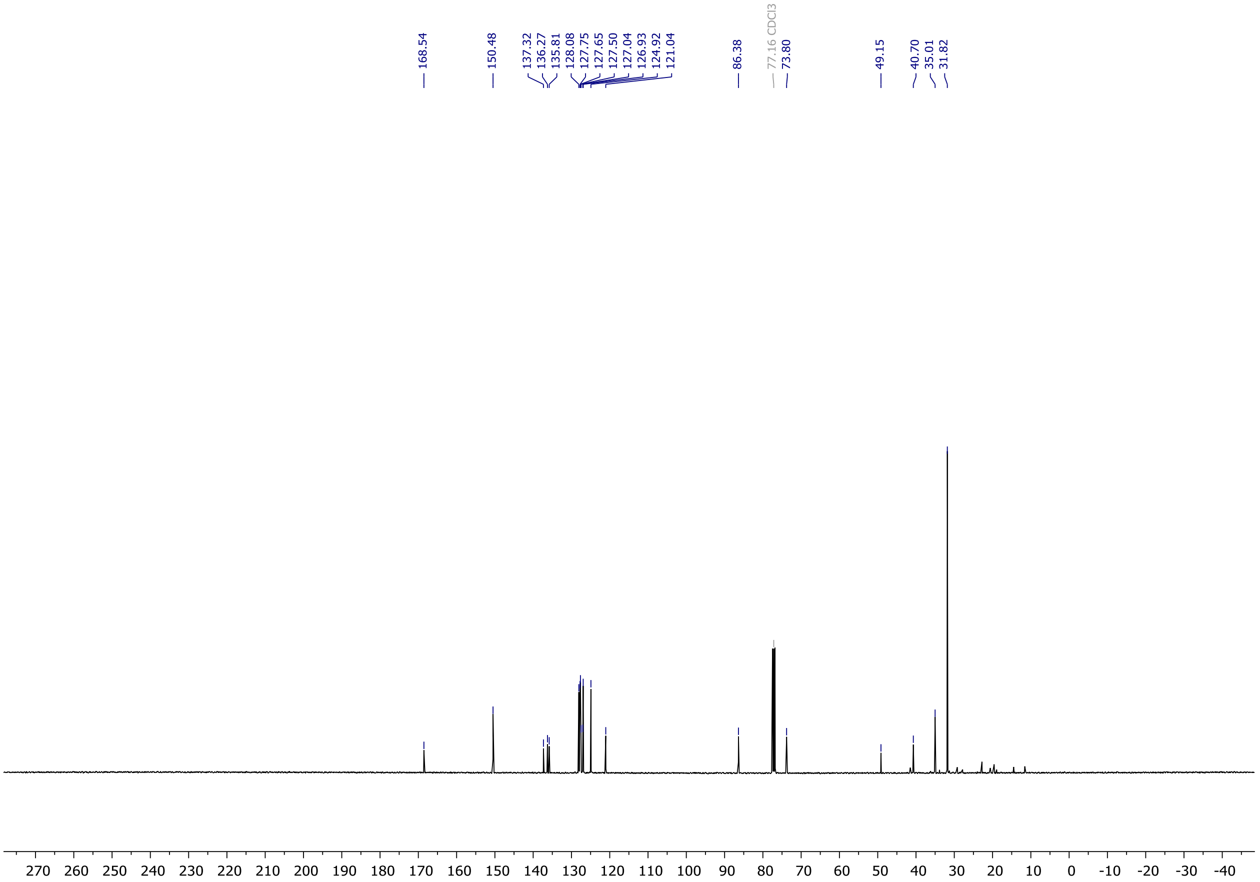

168.54
150.48
137.32
136.27
135.81
128.08
127.75
127.65
127.50
127.04
126.93
124.92
121.04
86.38
77.16 CDCl3
73.80
49.15
40.70
35.01
31.82
270 260 250 240 230 220 210 200 190 180 170 160 150 140 130 120 110 100 90 80 70 60 50 40 30 20 10 0 -10 -20 -30 -40

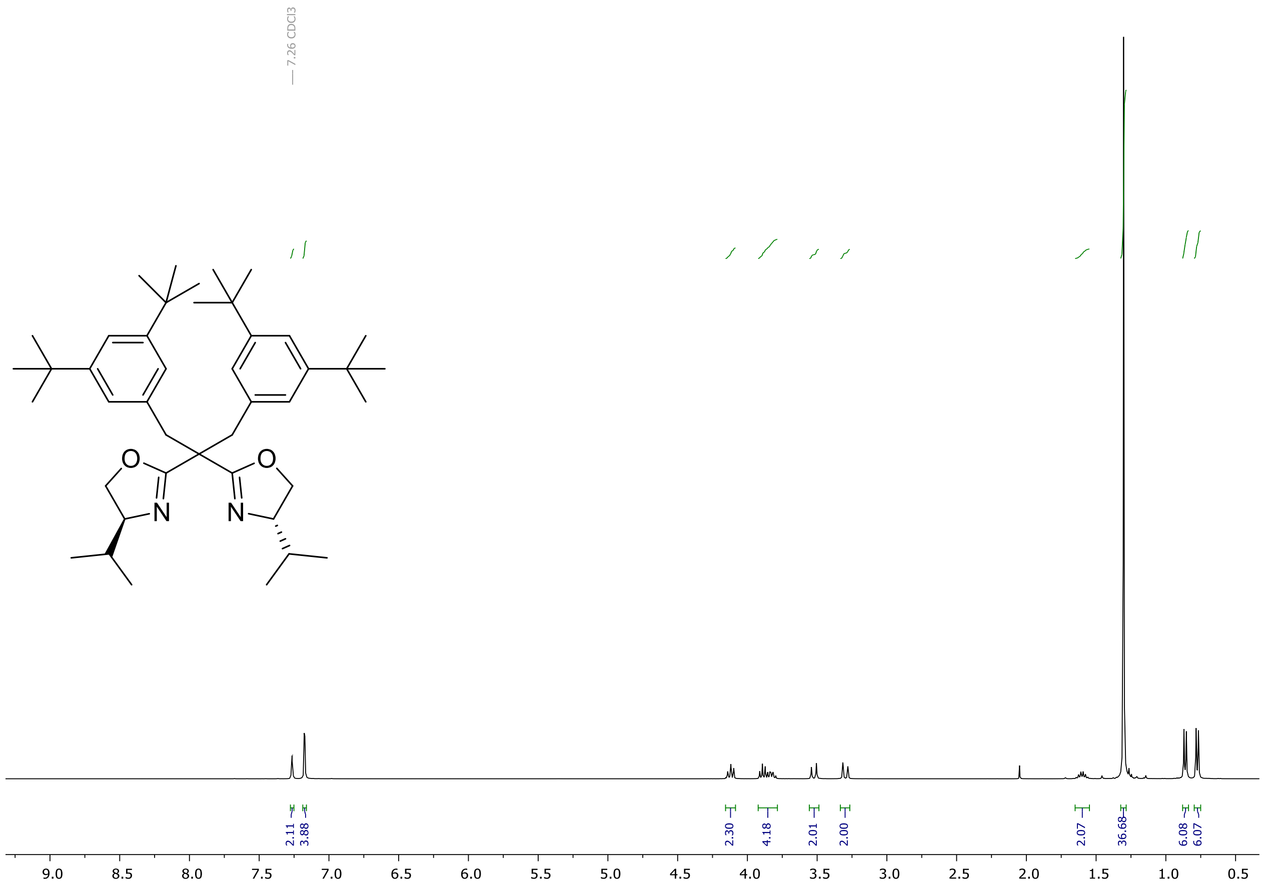
O
N
O
N
7.26 CDCl3
2.11
3.88
2.30
4.18
2.01
2.00
2.07
36.68
6.08
6.07
9.0 8.5 8.0 7.5 7.0 6.5 6.0 5.5 5.0 4.5 4.0 3.5 3.0 2.5 2.0 1.5 1.0 0.5

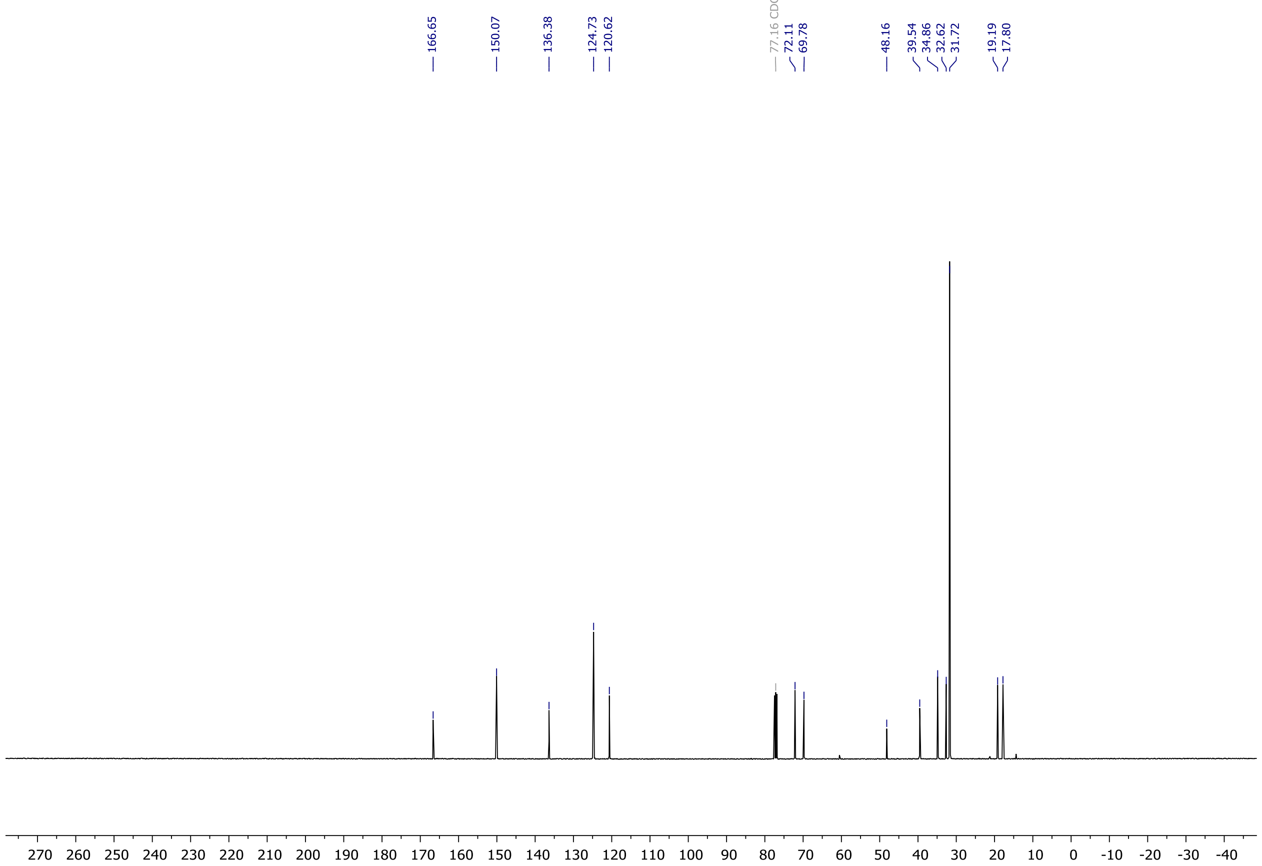
166.65
150.07
136.38
124.73
120.62
77.16 CDCl3
72.11
69.78
48.16
39.54
34.86
32.62
31.72
19.19
17.80
270 260 250 240 230 220 210 200 190 180 170 160 150 140 130 120 110 100 90 80 70 60 50 40 30 20 10 0 -10 -20 -30 -40

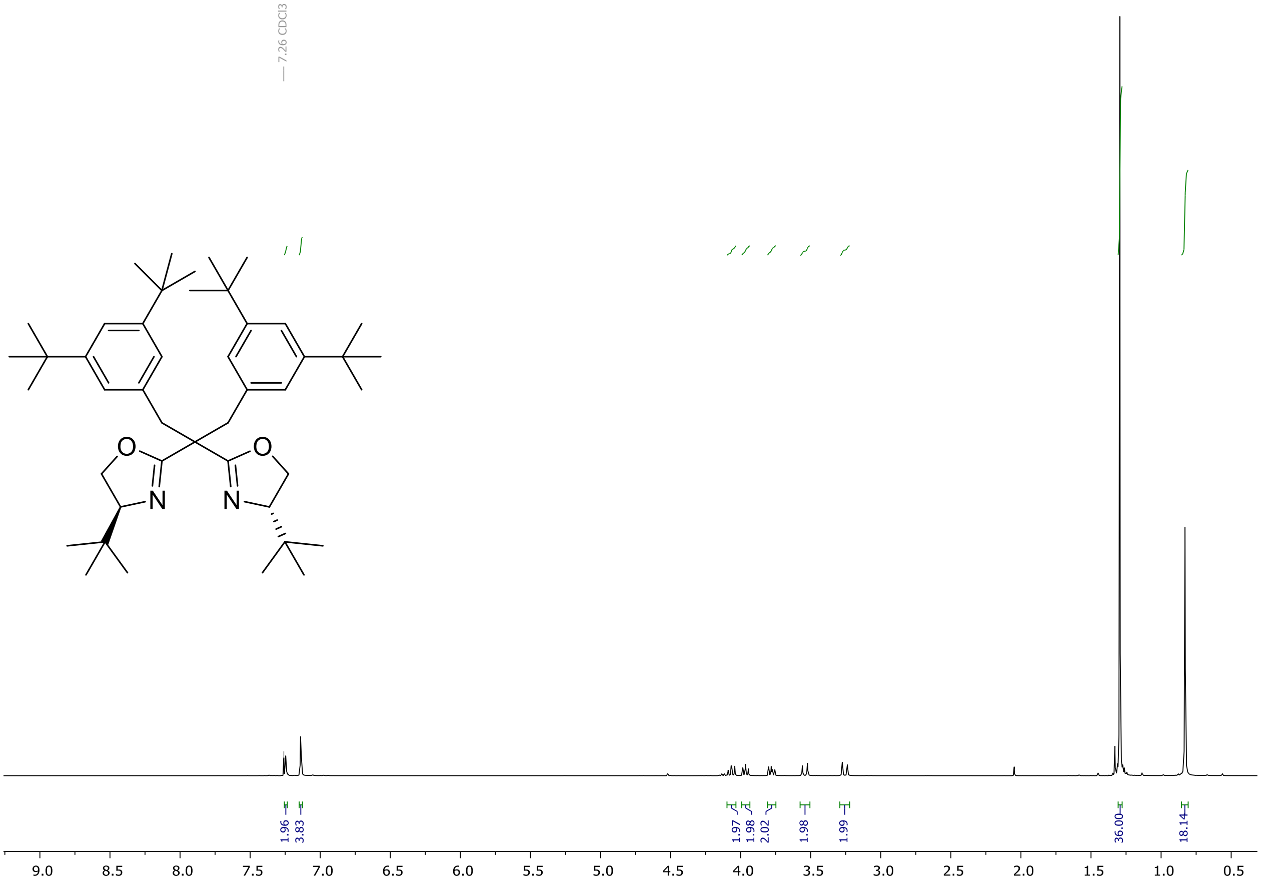

7.26 CDCl3
1.96
3.83
1.97
1.98
2.02
1.98
1.99
36.00
18.14
9.0 8.5 8.0 7.5 7.0 6.5 6.0 5.5 5.0 4.5 4.0 3.5 3.0 2.5 2.0 1.5 1.0 0.5

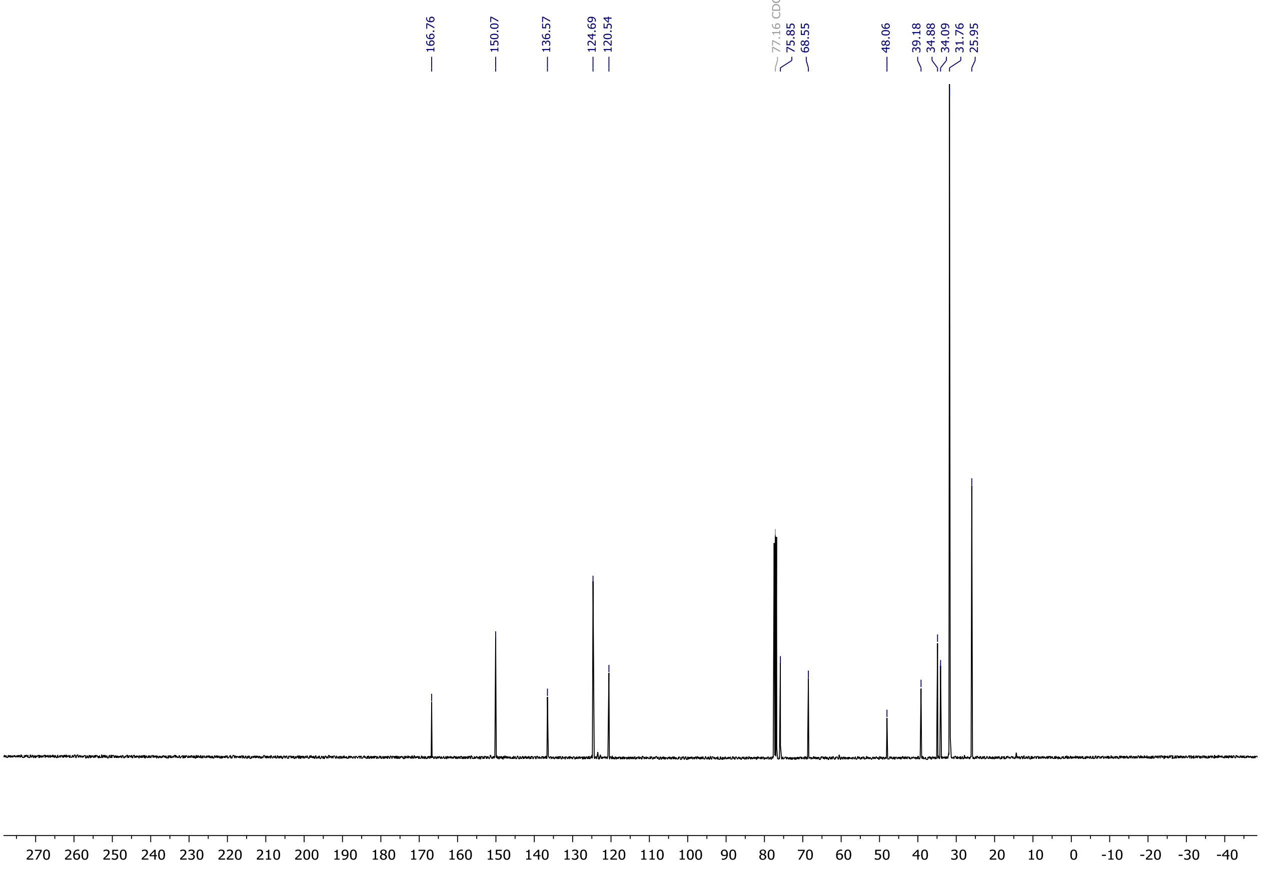

166.76
150.07
136.57
124.69
120.54
77.16 CDCl3
75.85
68.55
48.06
39.18
34.88
34.09
31.76
25.95
270 260 250 240 230 220 210 200 190 180 170 160 150 140 130 120 110 100 90 80 70 60 50 40 30 20 10 0 -10 -20 -30 -40

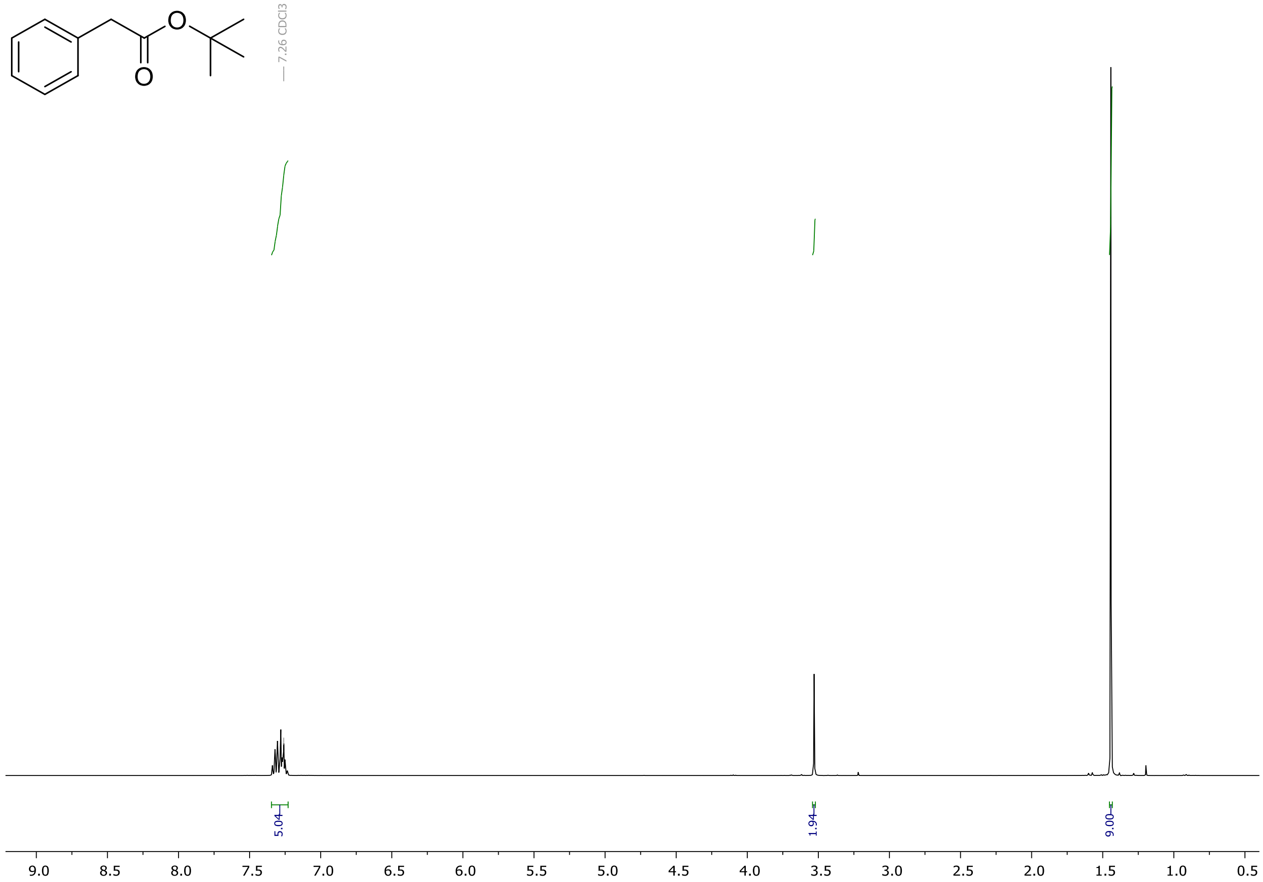
O
O
7.26 CDCl3
5.04
1.94
9.00
9.0 8.5 8.0 7.5 7.0 6.5 6.0 5.5 5.0 4.5 4.0 3.5 3.0 2.5 2.0 1.5 1.0 0.5

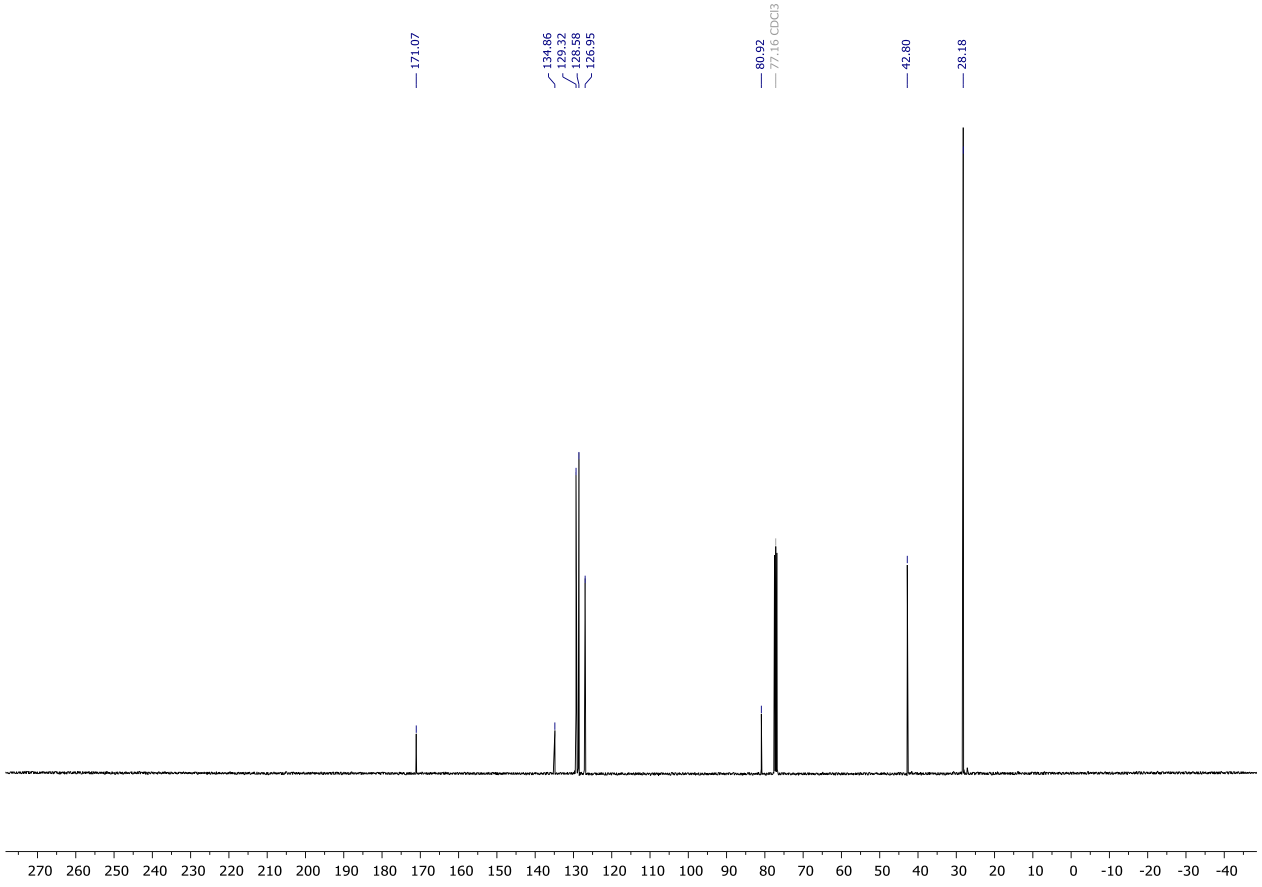
171.07
134.86
129.32
128.58
126.95
80.92
77.16 CDCl3
42.80
28.18
270 260 250 240 230 220 210 200 190 180 170 160 150 140 130 120 110 100 90 80 70 60 50 40 30 20 10 0 -10 -20 -30 -40

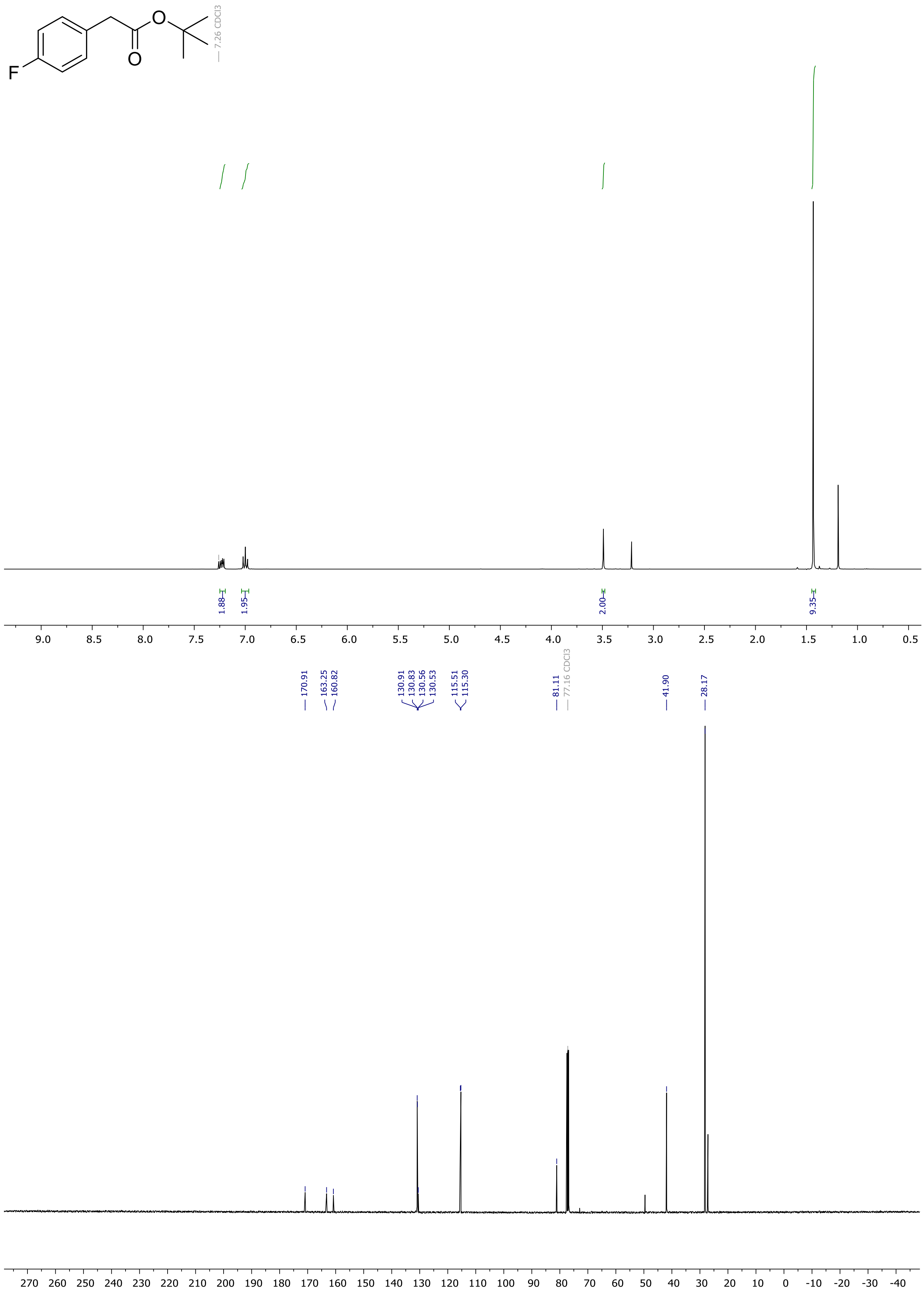
F
O
O
7.26 CDCl3
1.88
1.95
2.00
9.35
9.0 8.5 8.0 7.5 7.0 6.5 6.0 5.5 5.0 4.5 4.0 3.5 3.0 2.5 2.0 1.5 1.0 0.5
170.91
163.25
160.82
130.91
130.83
130.56
130.53
115.51
115.30
81.11
77.16 CDCl3
41.90
28.17
270 260 250 240 230 220 210 200 190 180 170 160 150 140 130 120 110 100 90 80 70 60 50 40 30 20 10 0 -10 -20 -30 -40

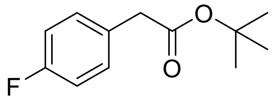

^{19}F NMR

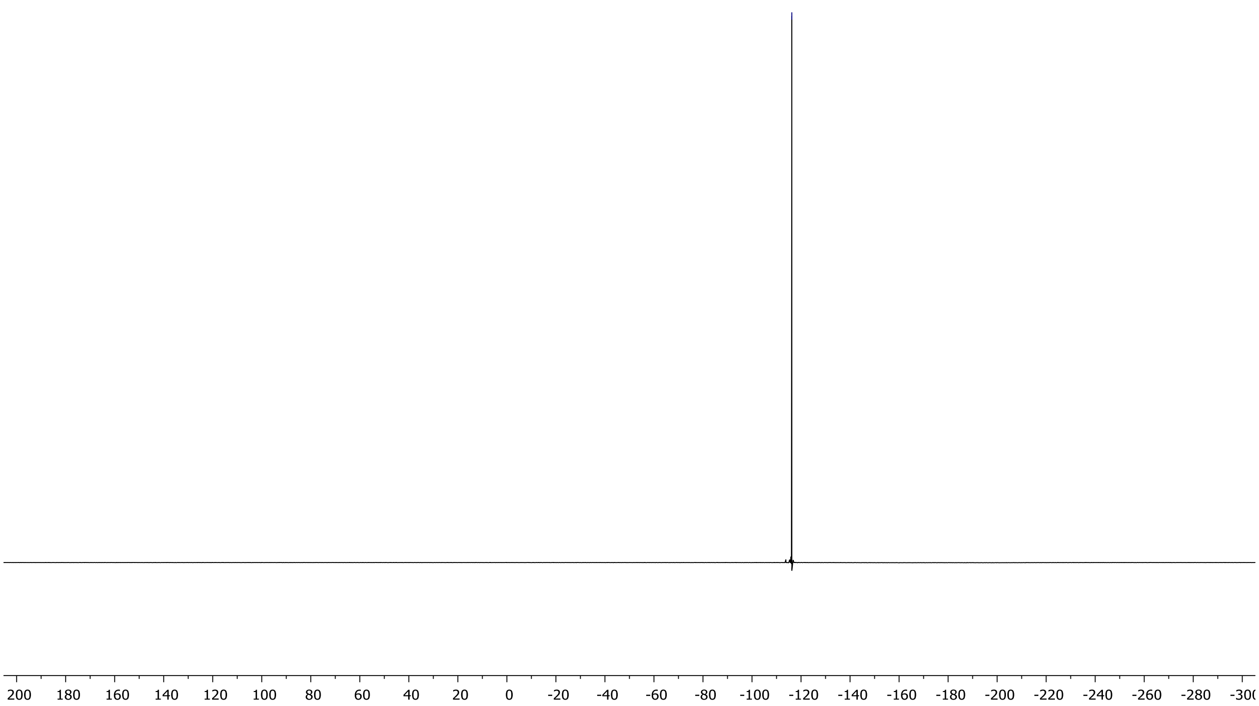

-116.25
200
180
160
140
120
100
80
60
40
20
0
-20
-40
-60
-80
-100
-120
-140
-160
-180
-200
-220
-240
-260
-280
-300

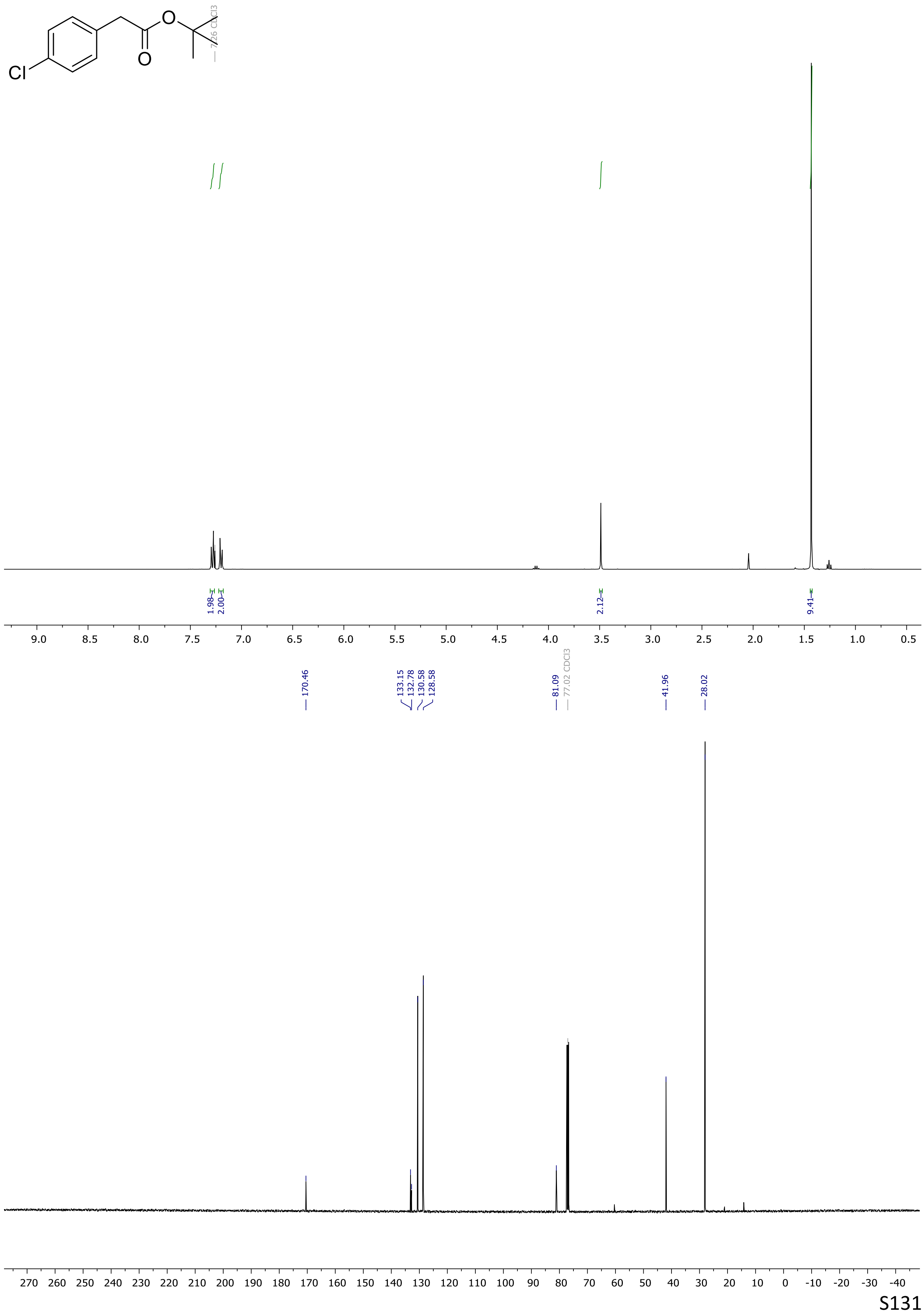
Cl
O
O
7.26 CDCl3
1.98
2.00
2.12
9.41
9.0
8.5
8.0
7.5
7.0
6.5
6.0
5.5
5.0
4.5
4.0
3.5
3.0
2.5
2.0
1.5
1.0
0.5
170.46
133.15
132.78
130.58
128.58
81.09
77.02 CDCl3
41.96
28.02
270 260 250 240 230 220 210 200 190 180 170 160 150 140 130 120 110 100 90 80 70 60 50 40 30 20 10 0 -10 -20 -30 -40

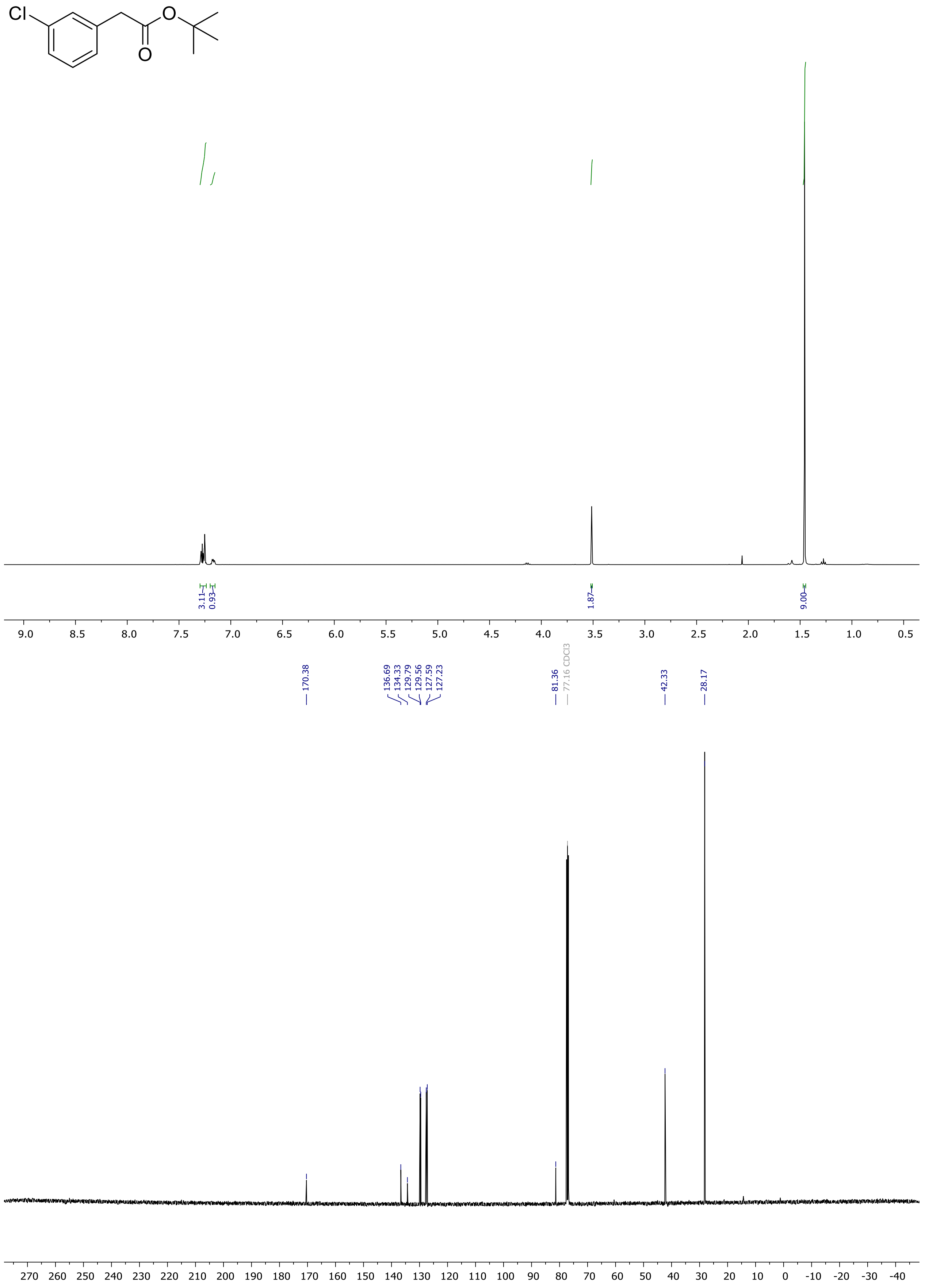
Cl
O
O
3.11
0.93
1.87
9.00
9.0 8.5 8.0 7.5 7.0 6.5 6.0 5.5 5.0 4.5 4.0 3.5 3.0 2.5 2.0 1.5 1.0 0.5
170.38
136.69
134.33
129.79
129.56
127.59
127.23
81.36
77.16 CDCl3
42.33
28.17
270 260 250 240 230 220 210 200 190 180 170 160 150 140 130 120 110 100 90 80 70 60 50 40 30 20 10 0 -10 -20 -30 -40

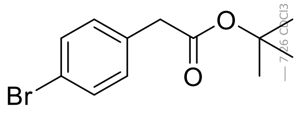
Br
O
O

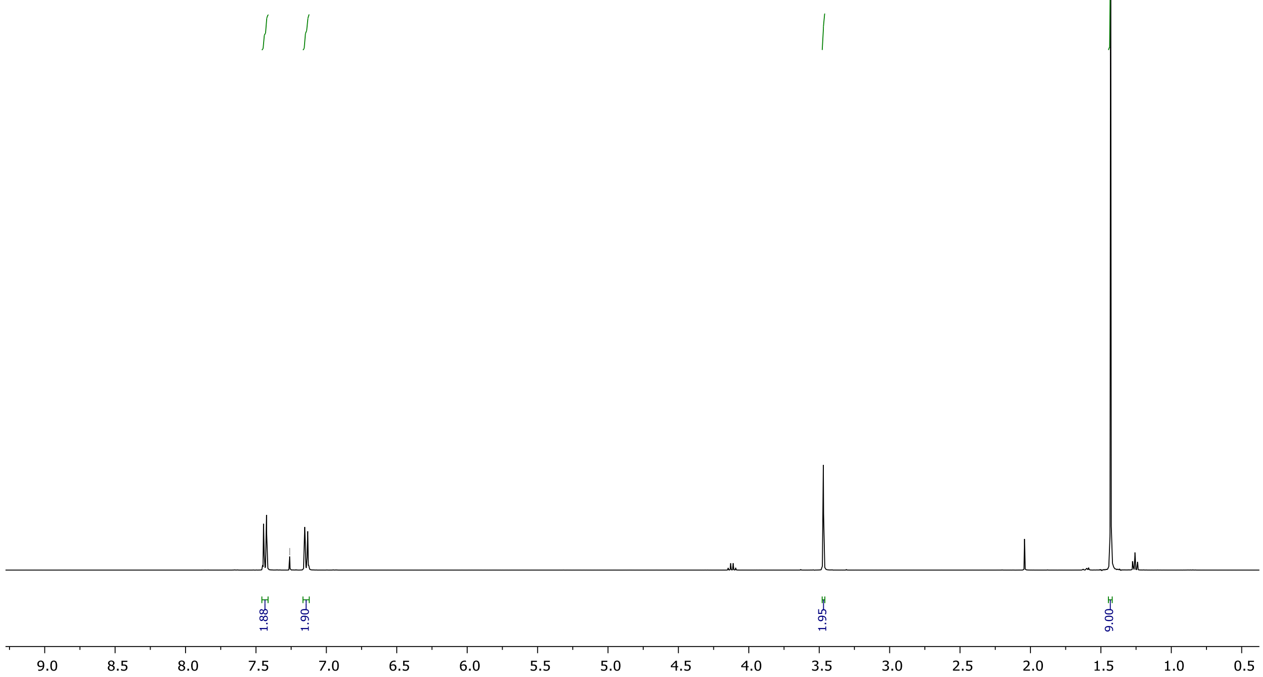
7.26 CDCl3
1.88
1.90
1.95
9.00
9.0
8.5
8.0
7.5
7.0
6.5
6.0
5.5
5.0
4.5
4.0
3.5
3.0
2.5
2.0
1.5
1.0
0.5

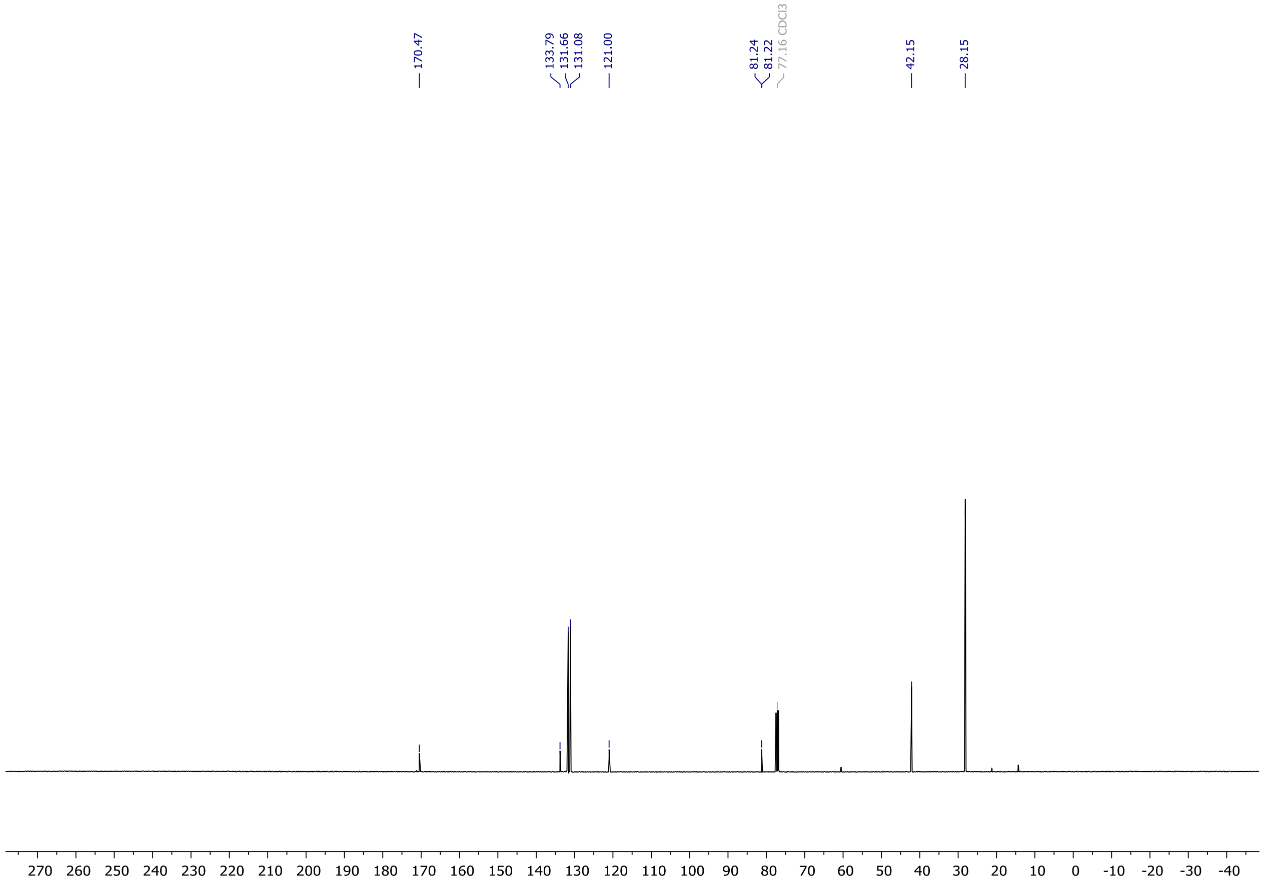
170.47
133.79
131.66
131.08
121.00
81.24
81.22
77.16 CDCl3
42.15
28.15
270 260 250 240 230 220 210 200 190 180 170 160 150 140 130 120 110 100 90 80 70 60 50 40 30 20 10 0 -10 -20 -30 -40

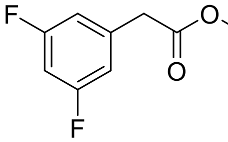
F
O
O
F

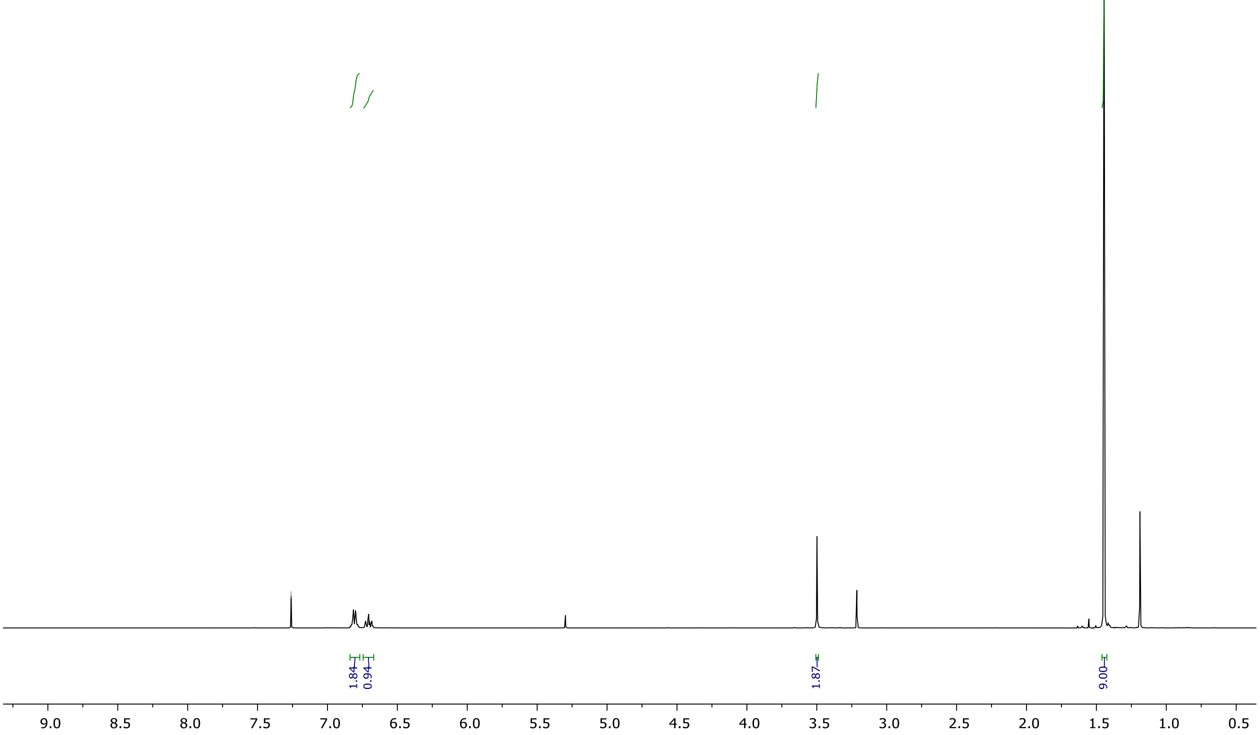
7.26 CDCl3
1.84
0.94
1.87
9.00
9.0 8.5 8.0 7.5 7.0 6.5 6.0 5.5 5.0 4.5 4.0 3.5 3.0 2.5 2.0 1.5 1.0 0.5

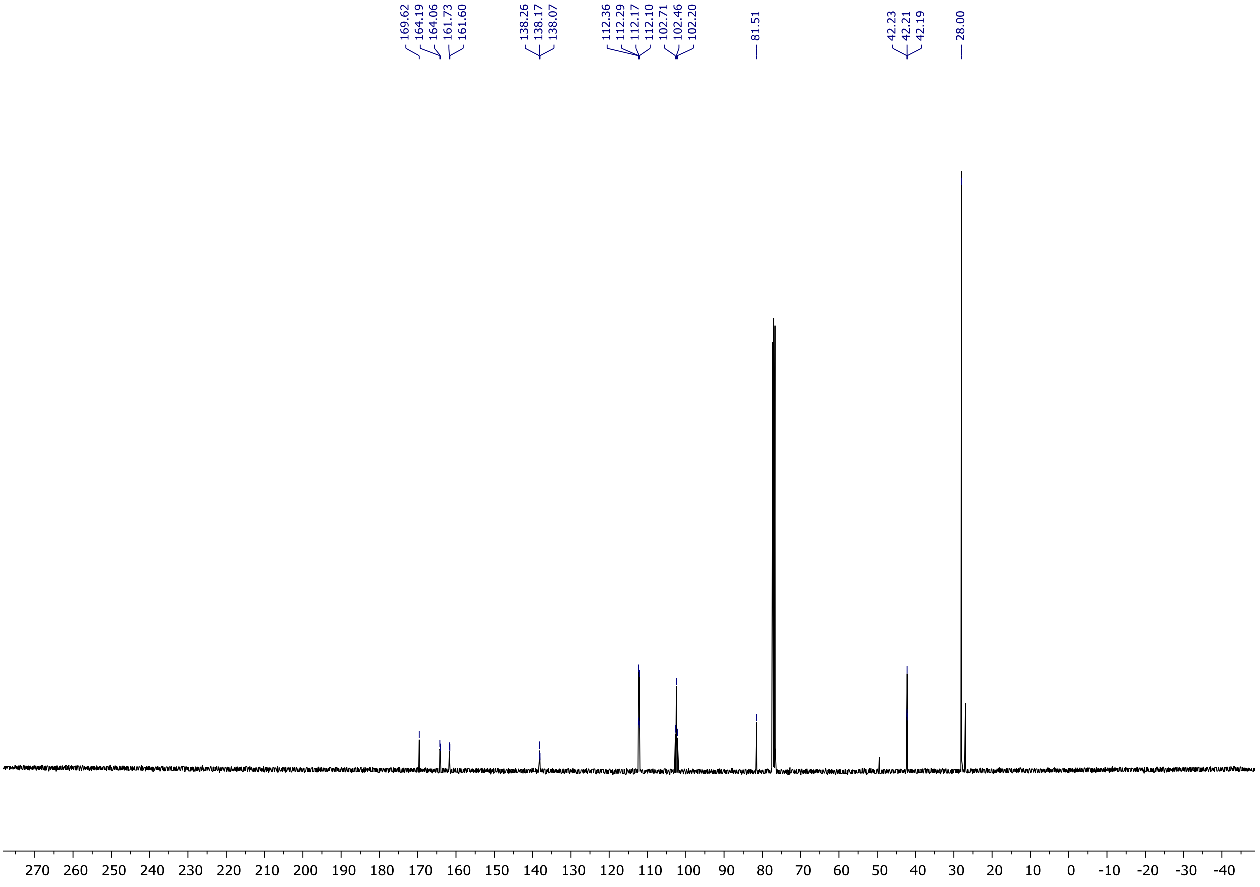
169.62
164.19
164.06
161.73
161.60
138.26
138.17
138.07
112.36
112.29
112.17
112.10
102.71
102.46
102.20
81.51
42.23
42.21
42.19
28.00
270 260 250 240 230 220 210 200 190 180 170 160 150 140 130 120 110 100 90 80 70 60 50 40 30 20 10 0 -10 -20 -30 -40

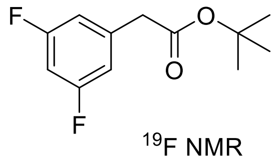
F
O
O
F

^{19}F NMR

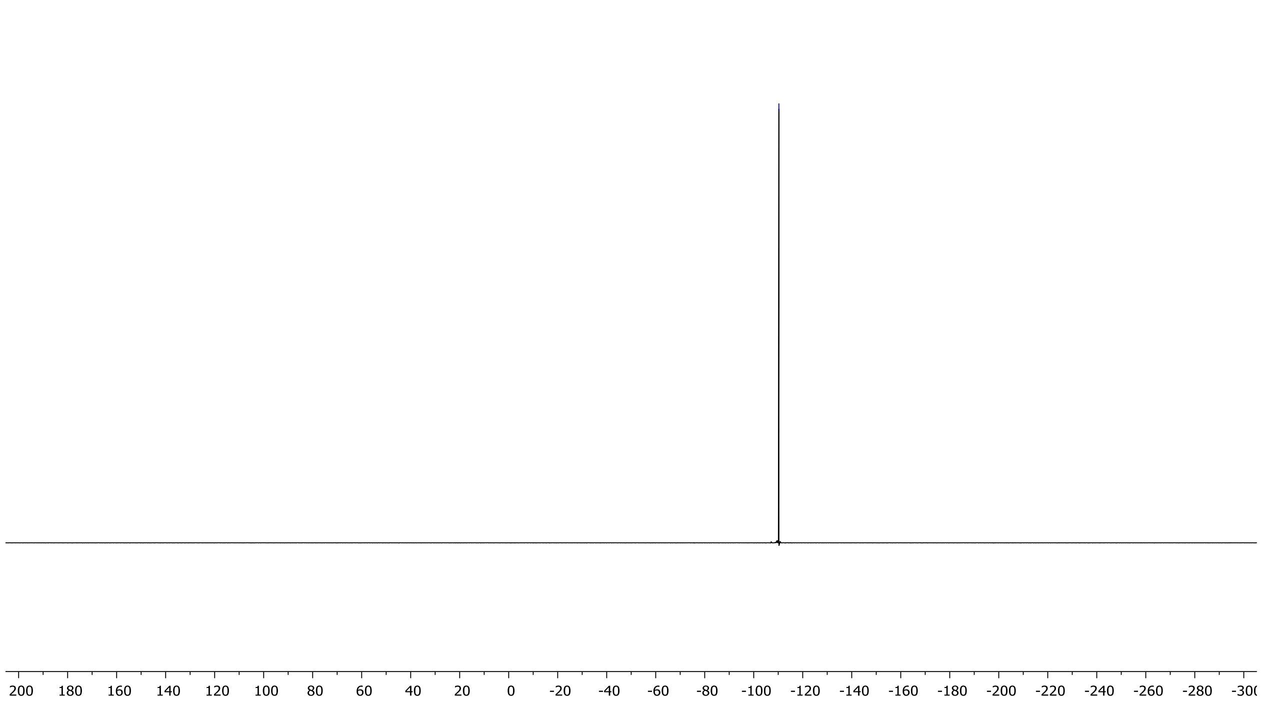
-110.27
200 180 160 140 120 100 80 60 40 20 0 -20 -40 -60 -80 -100 -120 -140 -160 -180 -200 -220 -240 -260 -280

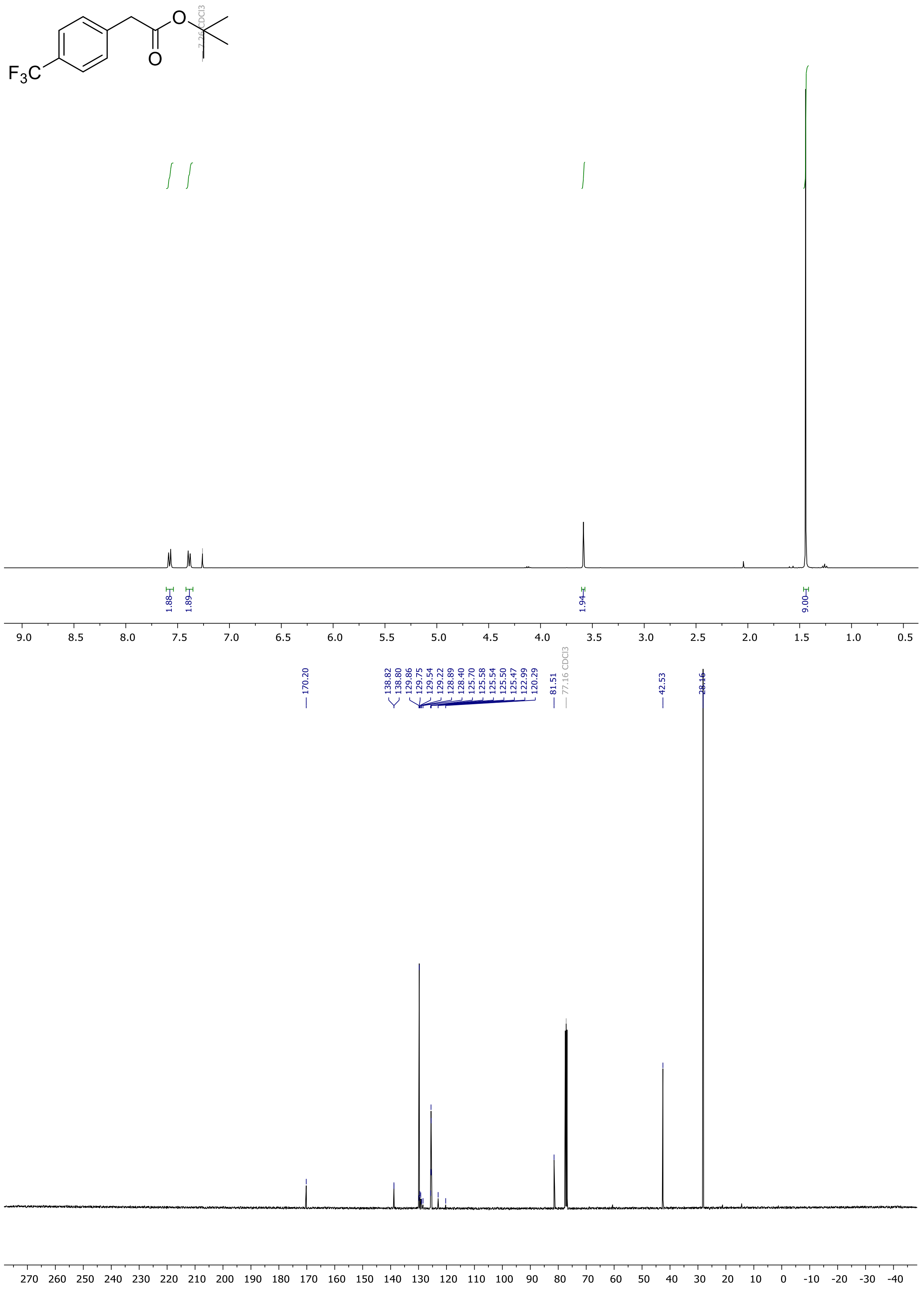
F3C
O
O
7.26 CDCl3
1.88
1.89
1.94
9.00
9.0
8.5
8.0
7.5
7.0
6.5
6.0
5.5
5.0
4.5
4.0
3.5
3.0
2.5
2.0
1.5
1.0
0.5
170.20
138.82
138.80
129.86
129.75
129.54
129.22
128.89
128.40
125.70
125.58
125.54
125.50
125.47
122.99
120.29
81.51
77.16 CDCl3
42.53
28.16
270 260 250 240 230 220 210 200 190 180 170 160 150 140 130 120 110 100 90 80 70 60 50 40 30 20 10 0 -10 -20 -30 -40

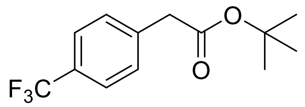

^{19}F NMR

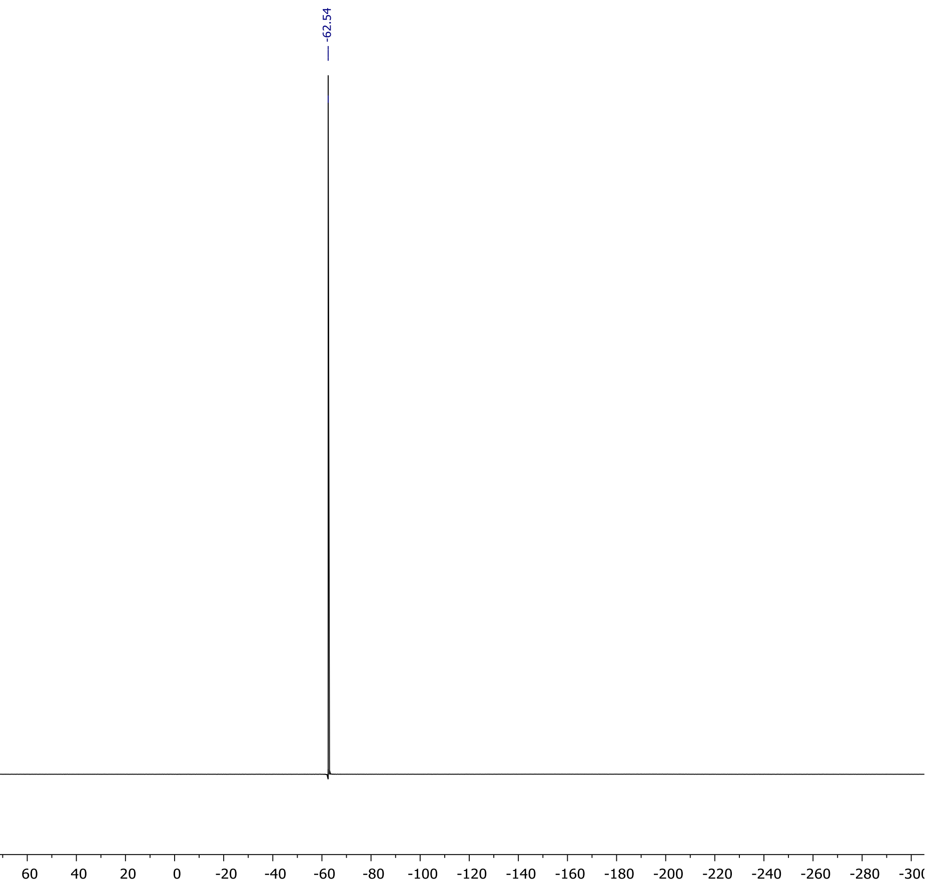
-62.54
200 180 160 140 120 100 80 60 40 20 0 -20 -40 -60 -80 -100 -120 -140 -160 -180 -200 -220 -240 -260 -280

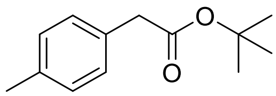

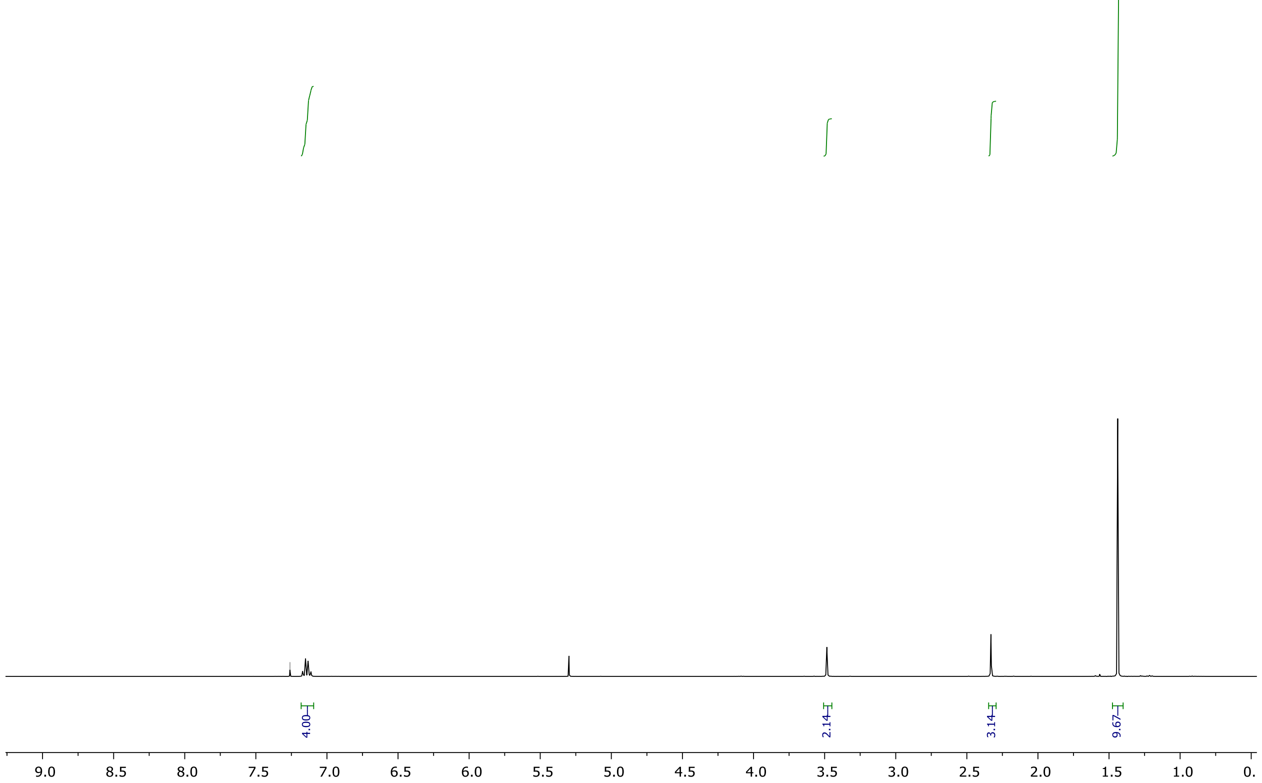
7.26 CDCl3
4.00
2.14
3.14
9.67
9.0 8.5 8.0 7.5 7.0 6.5 6.0 5.5 5.0 4.5 4.0 3.5 3.0 2.5 2.0 1.5 1.0 0.

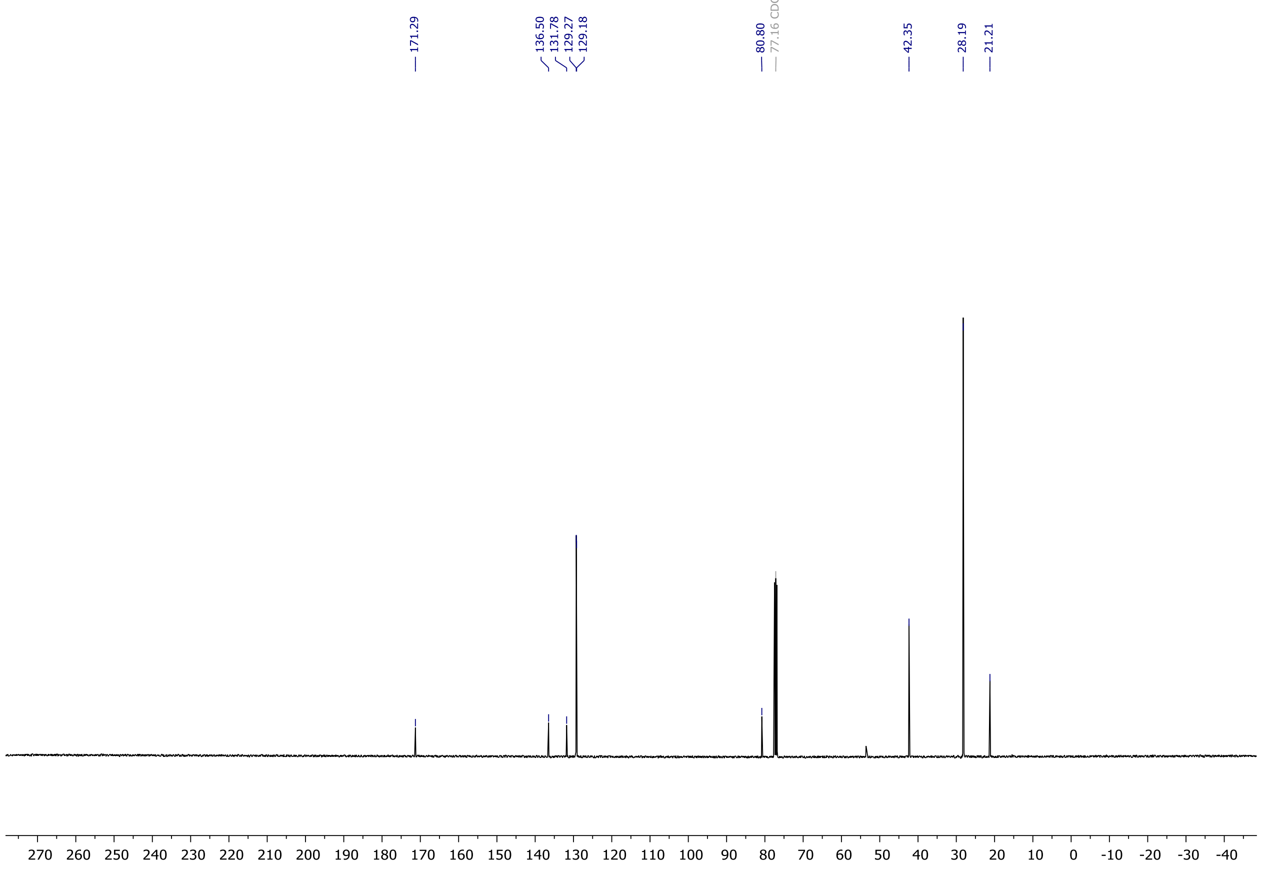
171.29
136.50
131.78
129.27
129.18
80.80
77.16 CDCl3
42.35
28.19
21.21
270 260 250 240 230 220 210 200 190 180 170 160 150 140 130 120 110 100 90 80 70 60 50 40 30 20 10 0 -10 -20 -30 -40

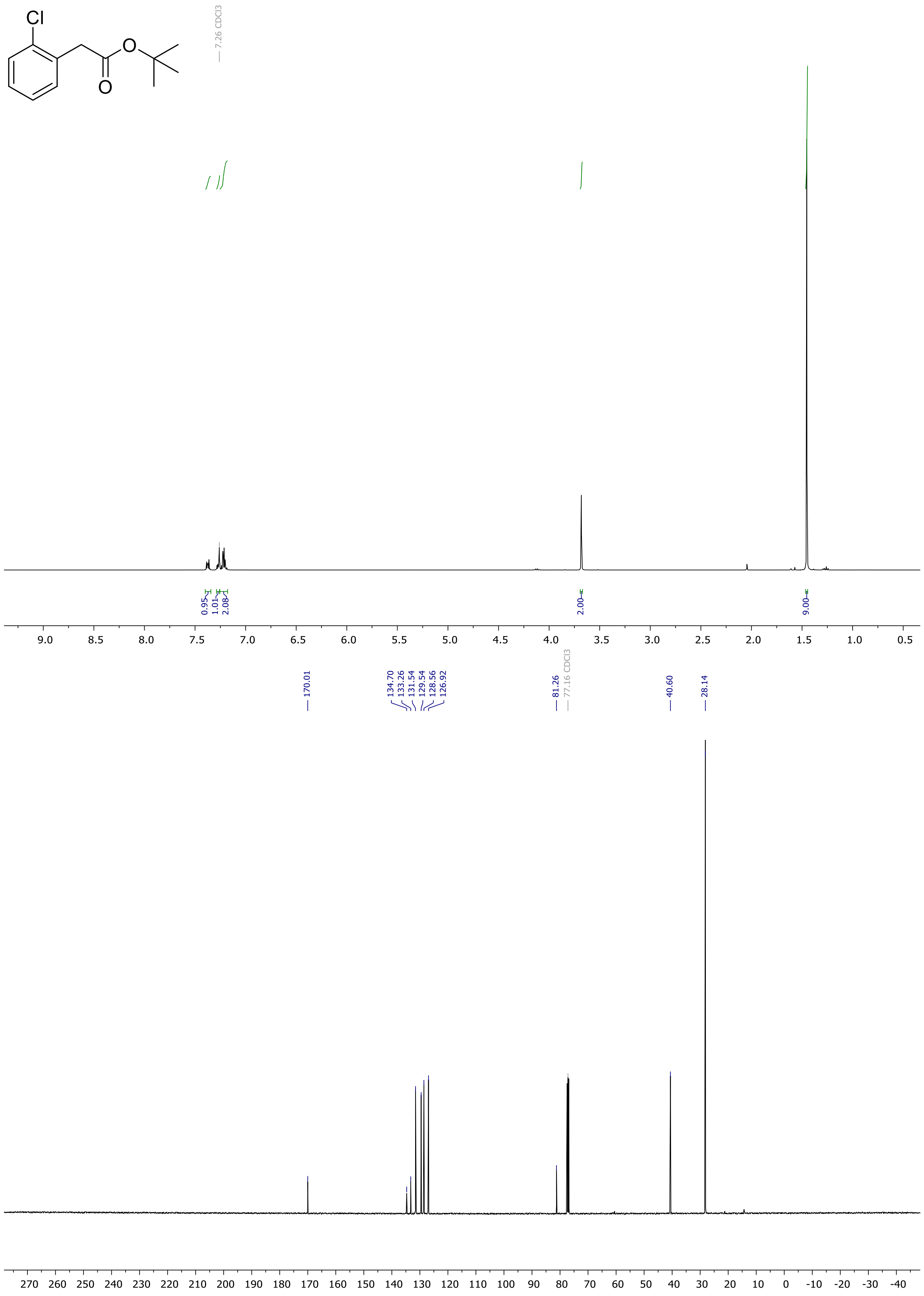

7.26 CDCl3
0.95
1.01
2.08
2.00
9.00
170.01
134.70
133.26
131.54
129.54
128.56
126.92
81.26
77.16 CDCl3
40.60
28.14

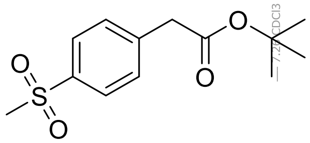
O
S
O
O
O

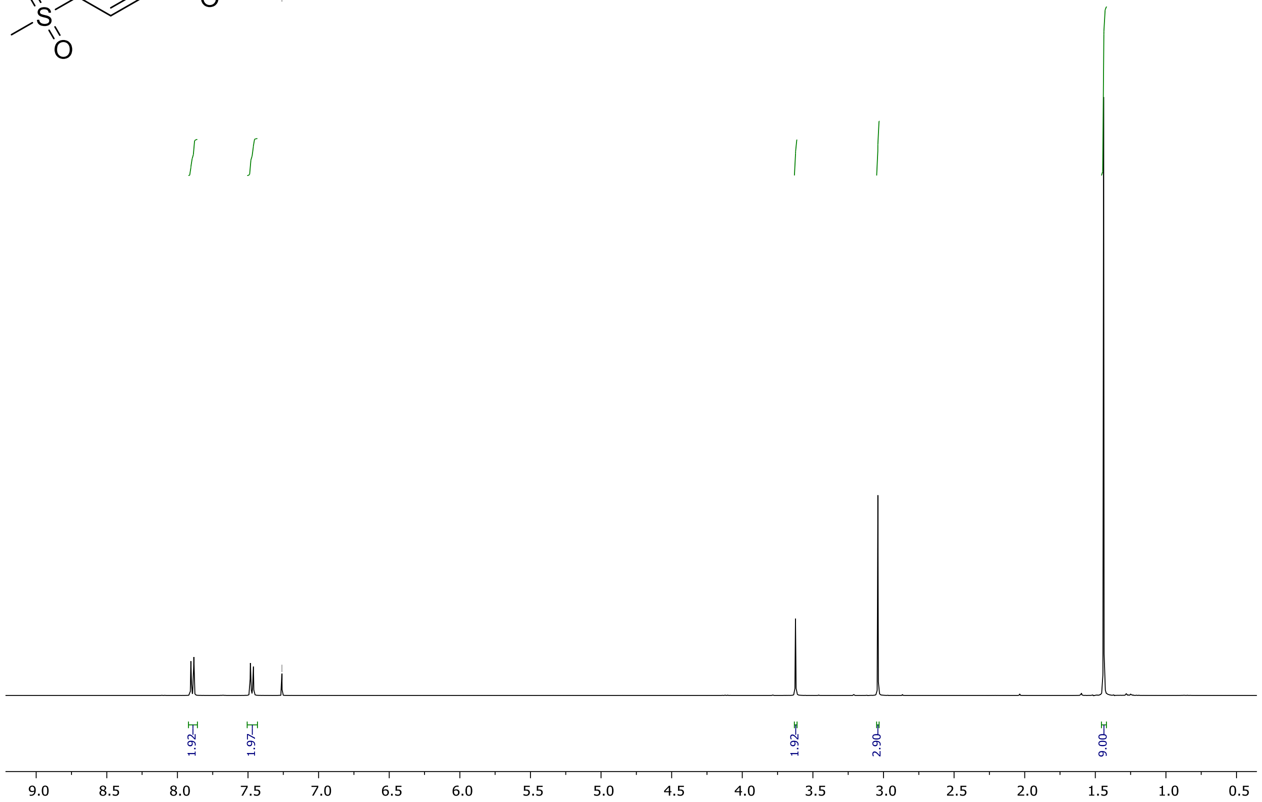
1.92
1.97
1.92
2.90
9.00
9.0 8.5 8.0 7.5 7.0 6.5 6.0 5.5 5.0 4.5 4.0 3.5 3.0 2.5 2.0 1.5 1.0 0.5

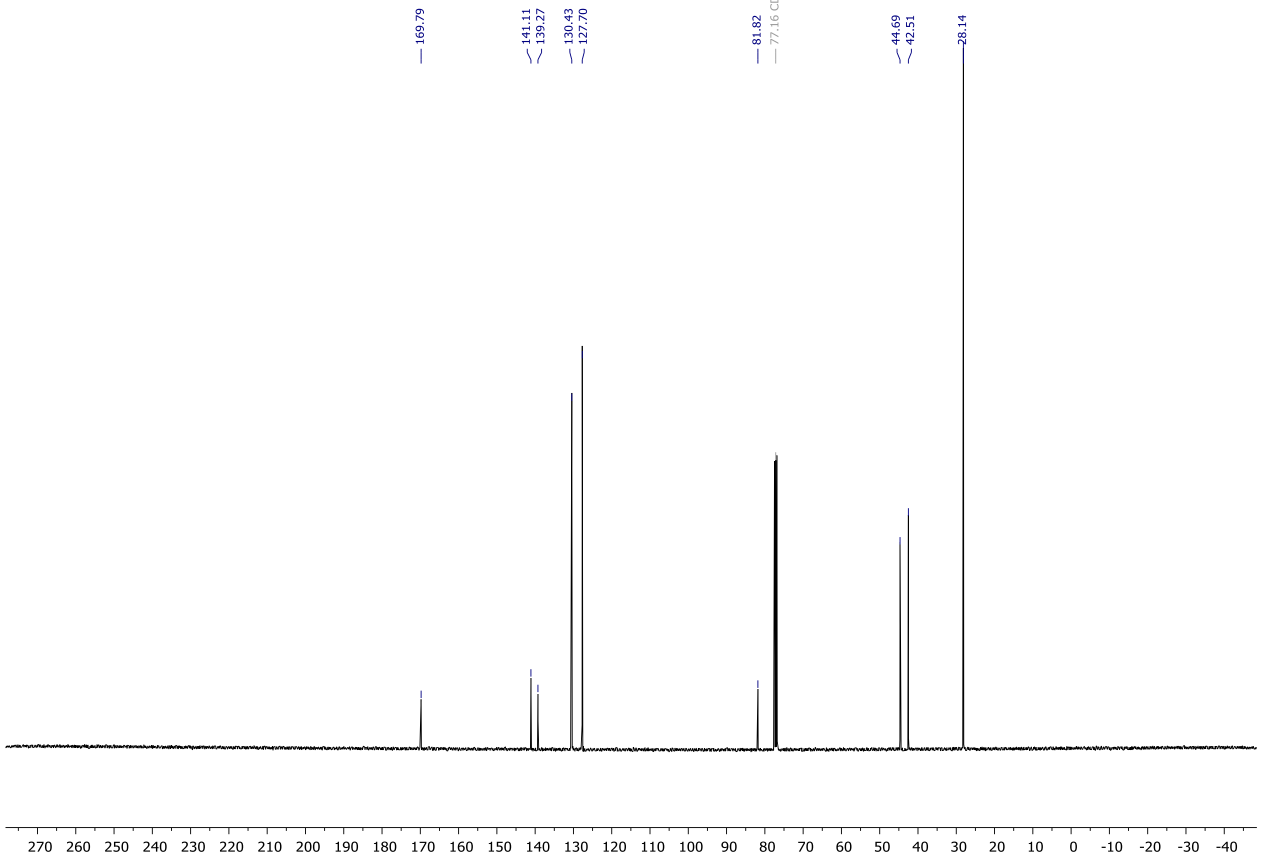
169.79
141.11
139.27
130.43
127.70
81.82
77.16 CDCl3
44.69
42.51
28.14
270 260 250 240 230 220 210 200 190 180 170 160 150 140 130 120 110 100 90 80 70 60 50 40 30 20 10 0 -10 -20 -30 -40

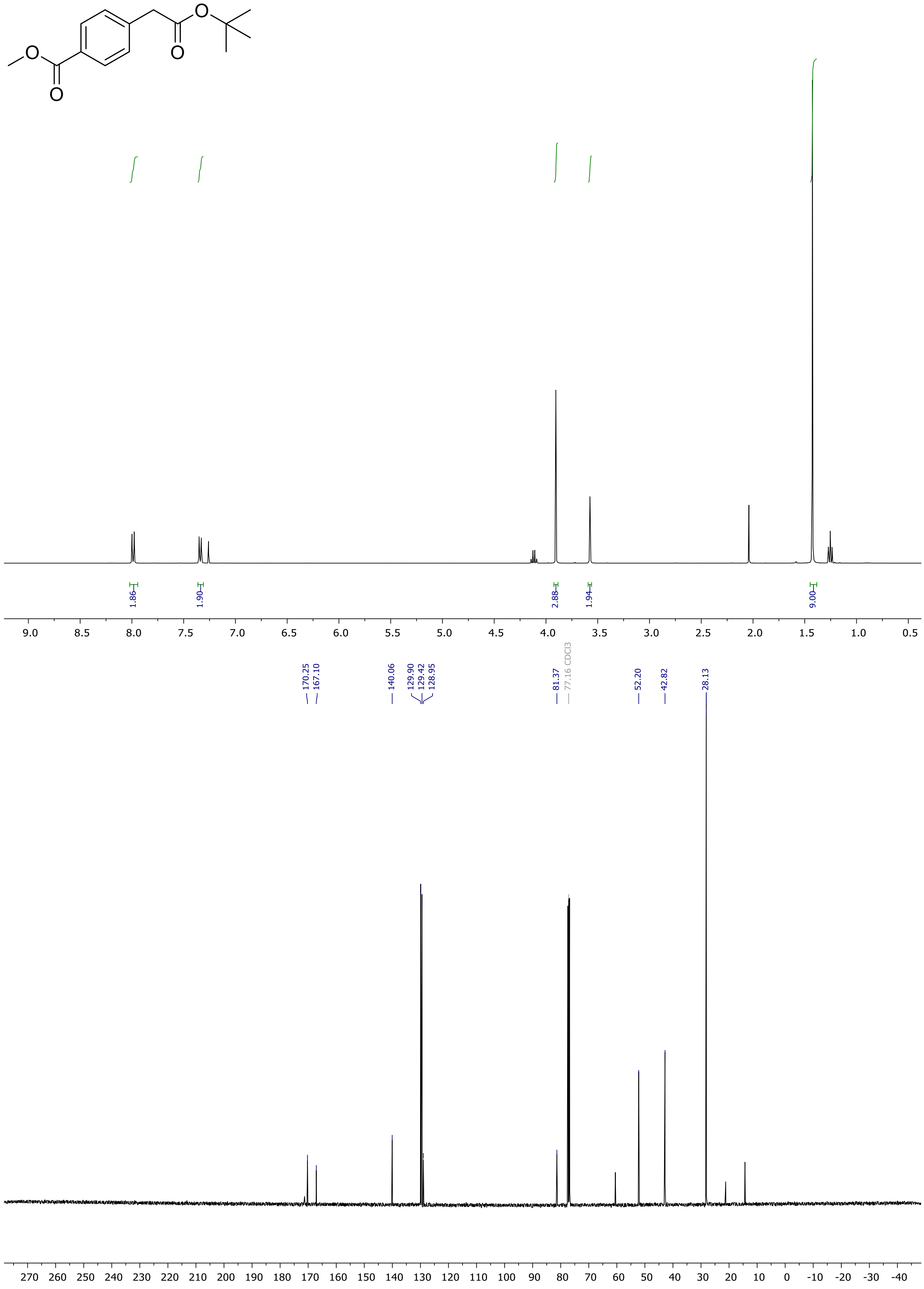

1.86
1.90
2.88
1.94
9.00
9.0 8.5 8.0 7.5 7.0 6.5 6.0 5.5 5.0 4.5 4.0 3.5 3.0 2.5 2.0 1.5 1.0 0.5
170.25
167.10
140.06
129.90
129.42
128.95
81.37
77.16 CDCl3
52.20
42.82
28.13
270 260 250 240 230 220 210 200 190 180 170 160 150 140 130 120 110 100 90 80 70 60 50 40 30 20 10 0 -10 -20 -30 -40

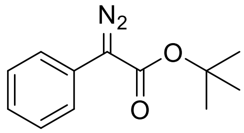

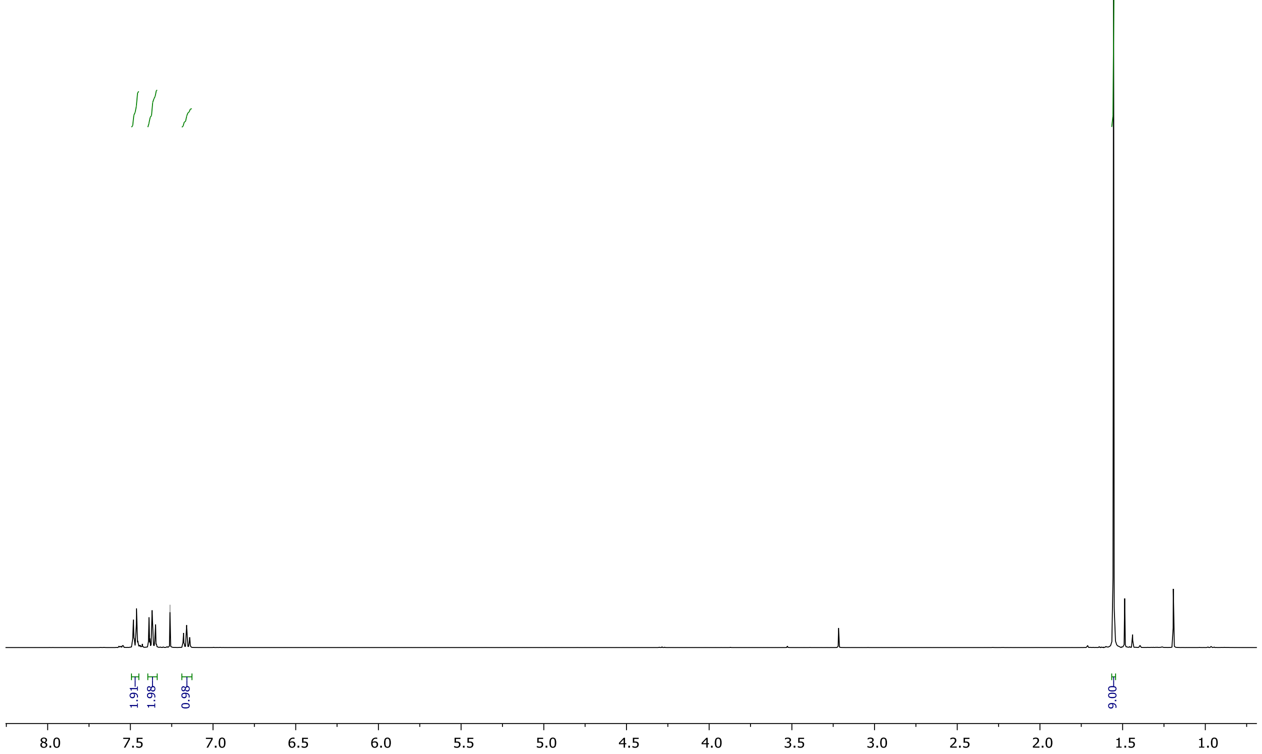
7.26 CDCl3
1.91
1.98
0.98
9.00
8.0
7.5
7.0
6.5
6.0
5.5
5.0
4.5
4.0
3.5
3.0
2.5
2.0
1.5
1.0

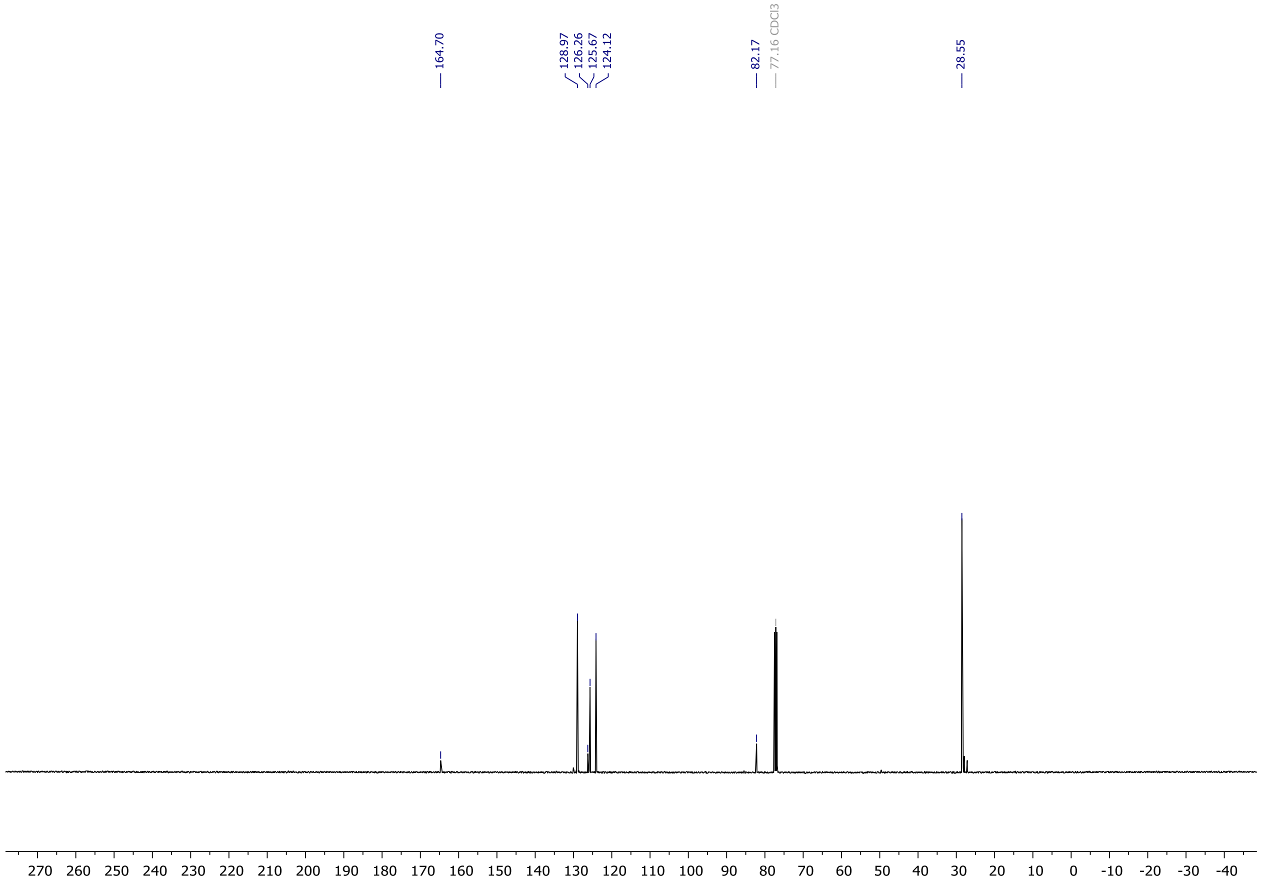
164.70
128.97
126.26
125.67
124.12
82.17
77.16 CDCl3
28.55
270 260 250 240 230 220 210 200 190 180 170 160 150 140 130 120 110 100 90 80 70 60 50 40 30 20 10 0 -10 -20 -30 -40

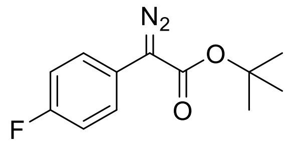
N2
O
O
F

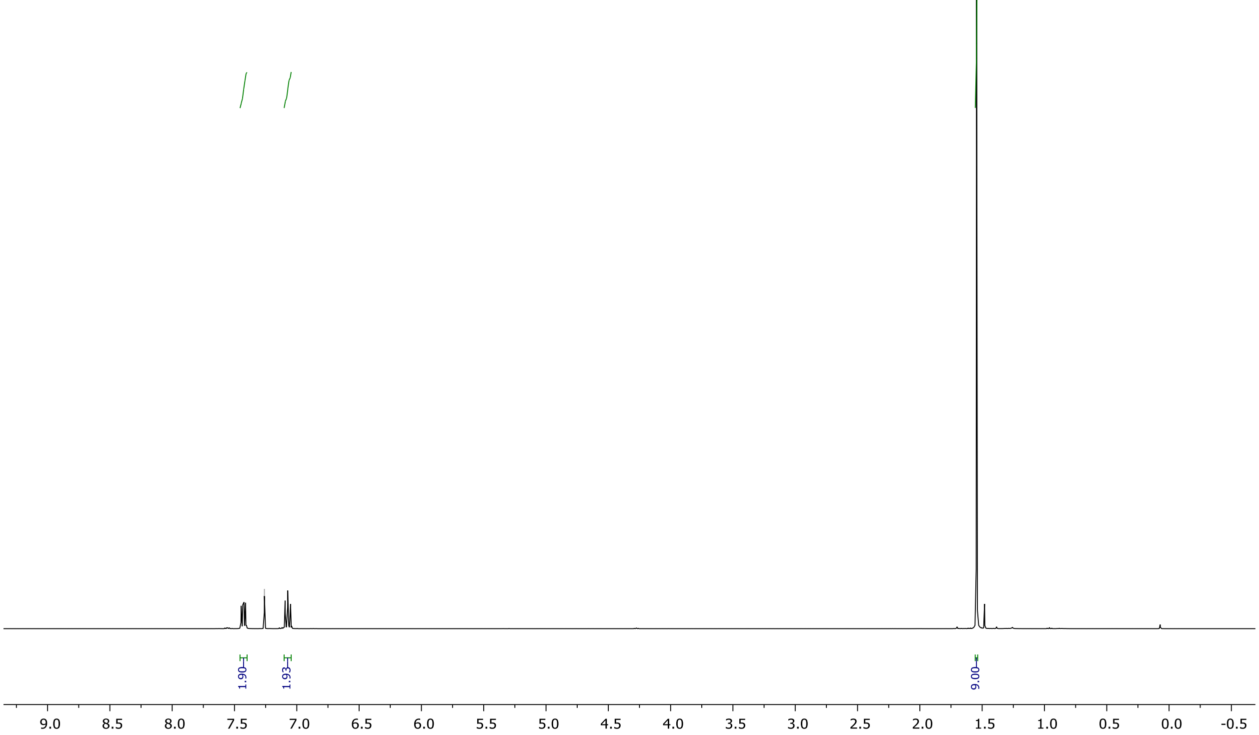
7.26 CDCl3
1.90
1.93
9.00
9.0 8.5 8.0 7.5 7.0 6.5 6.0 5.5 5.0 4.5 4.0 3.5 3.0 2.5 2.0 1.5 1.0 0.5 0.0 -0.5

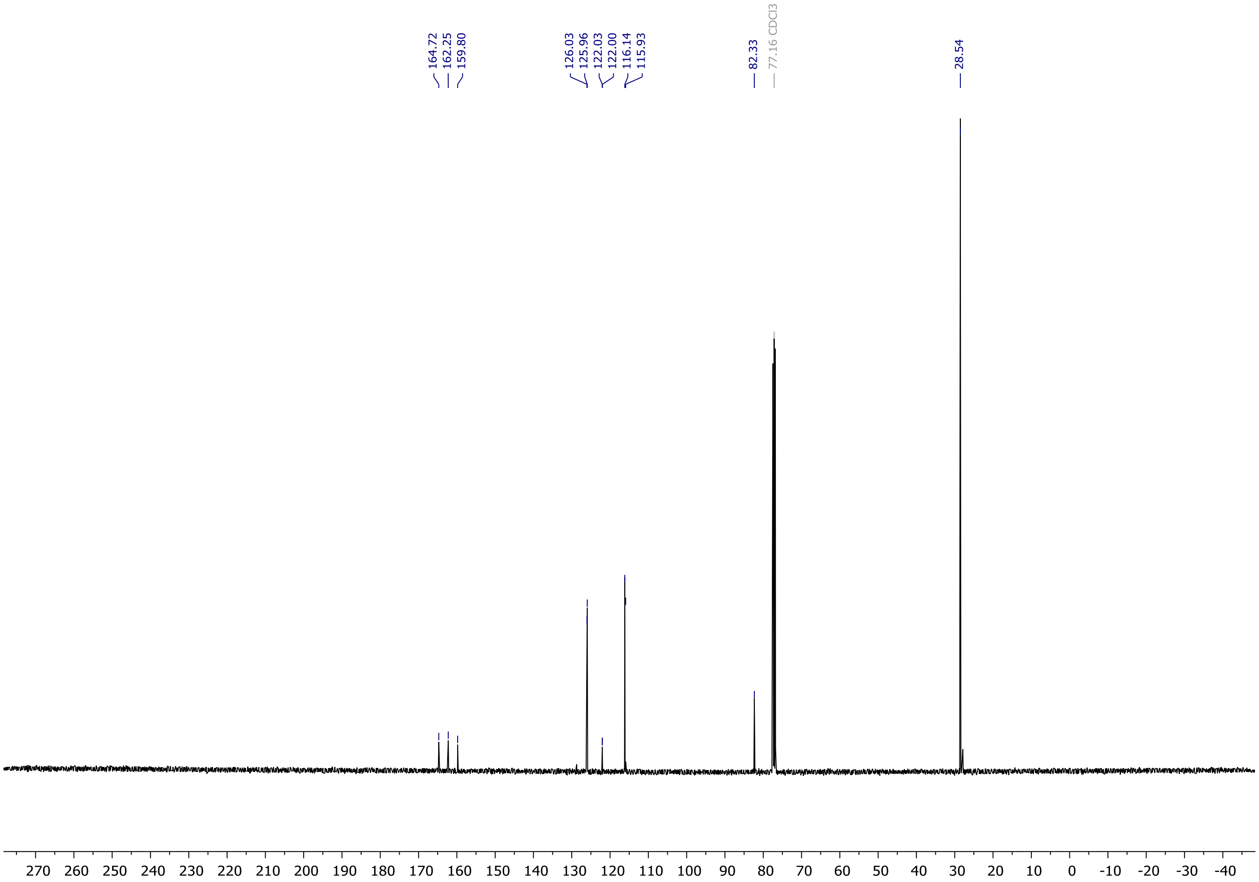
164.72
162.25
159.80
126.03
125.96
122.03
122.00
116.14
115.93
82.33
77.16 CDCl3
28.54
270 260 250 240 230 220 210 200 190 180 170 160 150 140 130 120 110 100 90 80 70 60 50 40 30 20 10 0 -10 -20 -30 -40

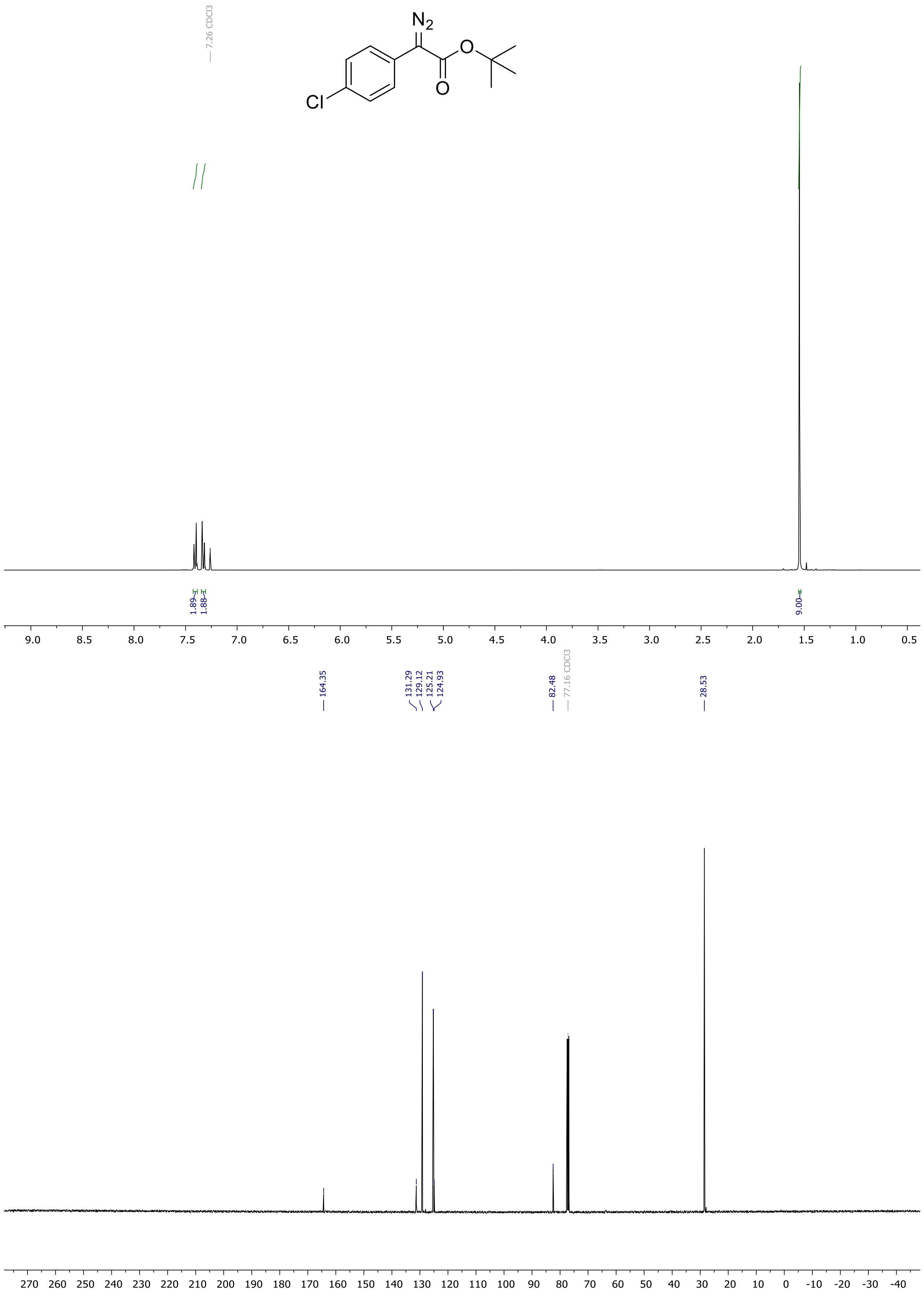

N2
O
O
Cl
7.26 CDCl3
1.89
1.88
9.00
9.0 8.5 8.0 7.5 7.0 6.5 6.0 5.5 5.0 4.5 4.0 3.5 3.0 2.5 2.0 1.5 1.0 0.5
164.35
131.29
129.12
125.21
124.93
82.48
77.16 CDCl3
28.53
270 260 250 240 230 220 210 200 190 180 170 160 150 140 130 120 110 100 90 80 70 60 50 40 30 20 10 0 -10 -20 -30 -40

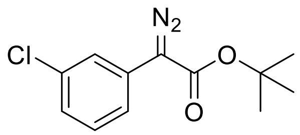

Cl
N_2
O
O

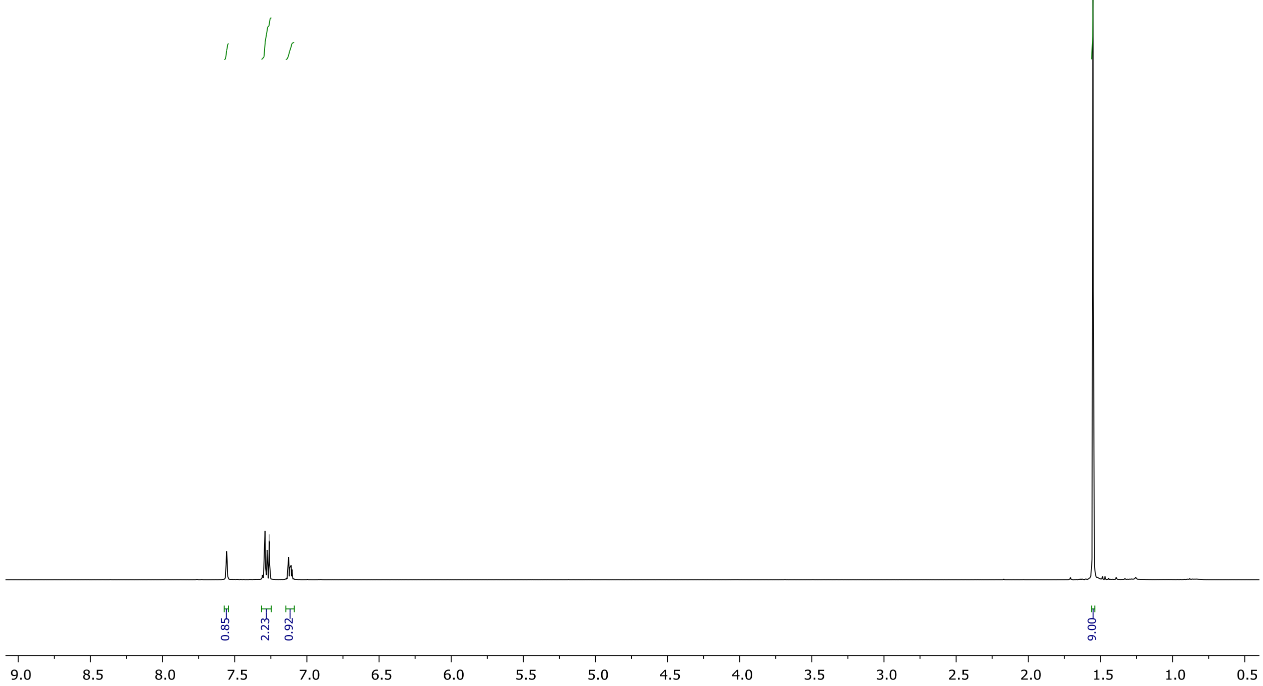

0.85
2.23
0.92
9.00
9.0 8.5 8.0 7.5 7.0 6.5 6.0 5.5 5.0 4.5 4.0 3.5 3.0 2.5 2.0 1.5 1.0 0.5

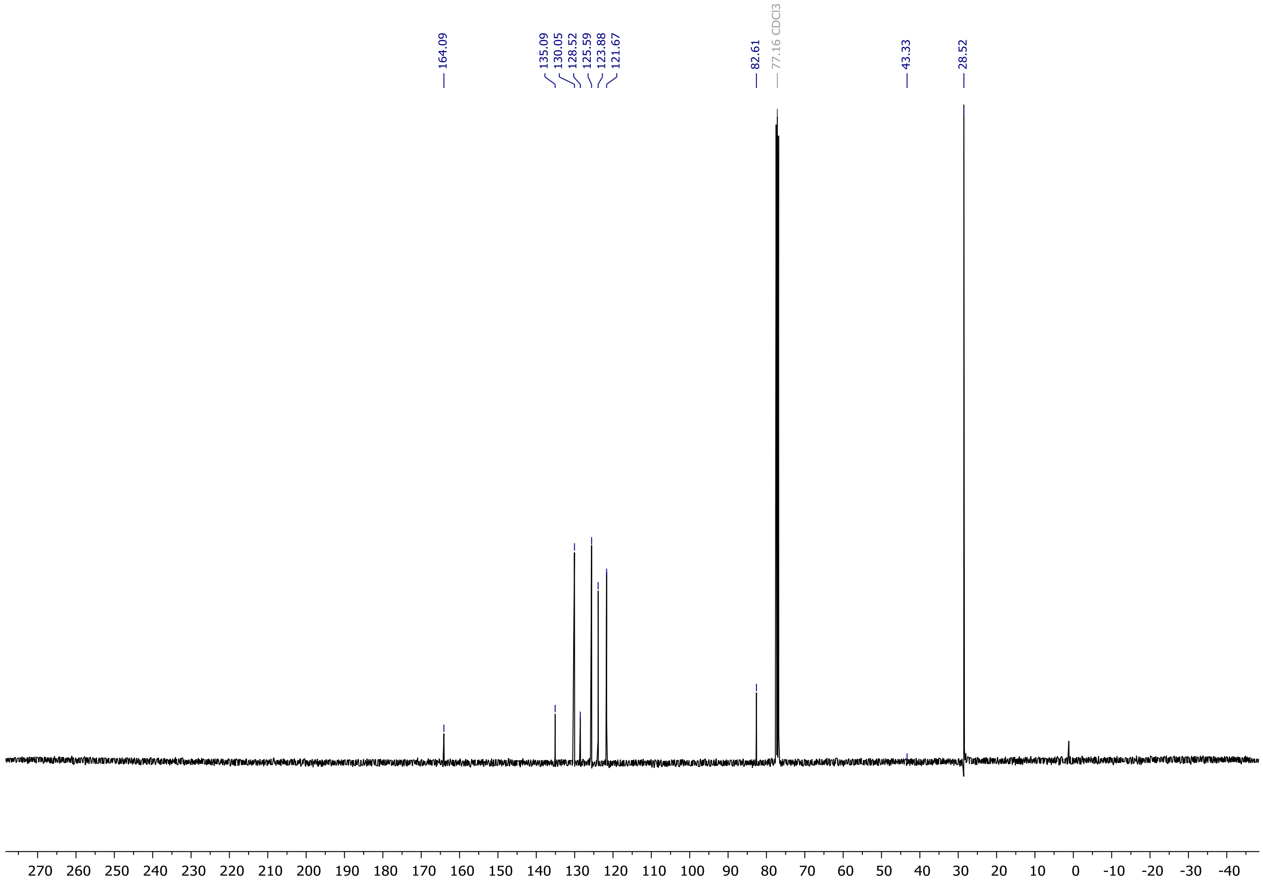

164.09
135.09
130.05
128.52
125.59
123.88
121.67
82.61
77.16 CDCl3
43.33
28.52
270 260 250 240 230 220 210 200 190 180 170 160 150 140 130 120 110 100 90 80 70 60 50 40 30 20 10 0 -10 -20 -30 -40

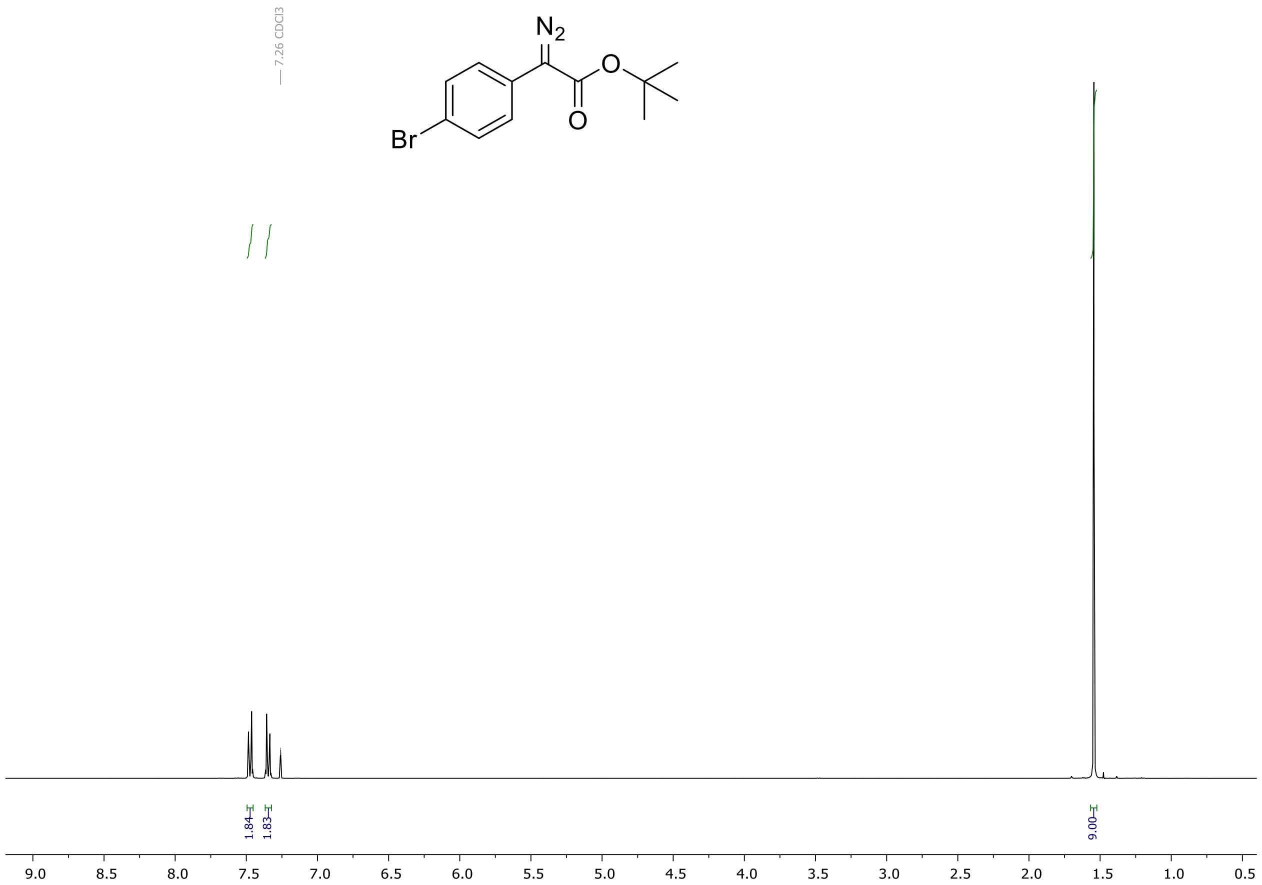
N2
O
O
Br
7.26 CDCl3
1.84
1.83
9.00
9.0 8.5 8.0 7.5 7.0 6.5 6.0 5.5 5.0 4.5 4.0 3.5 3.0 2.5 2.0 1.5 1.0 0.5

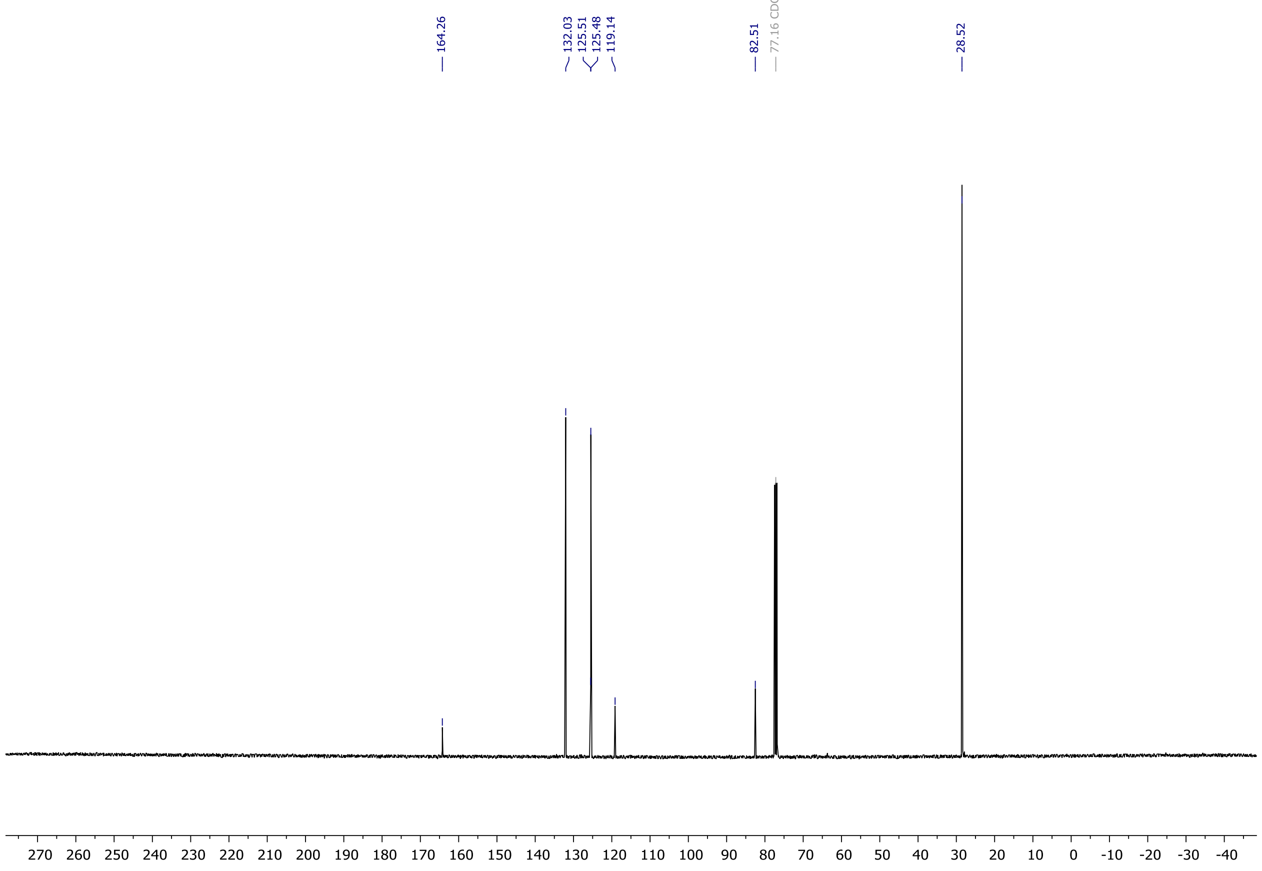
164.26
132.03
125.51
125.48
119.14
82.51
77.16 CDCl3
28.52
270 260 250 240 230 220 210 200 190 180 170 160 150 140 130 120 110 100 90 80 70 60 50 40 30 20 10 0 -10 -20 -30 -40

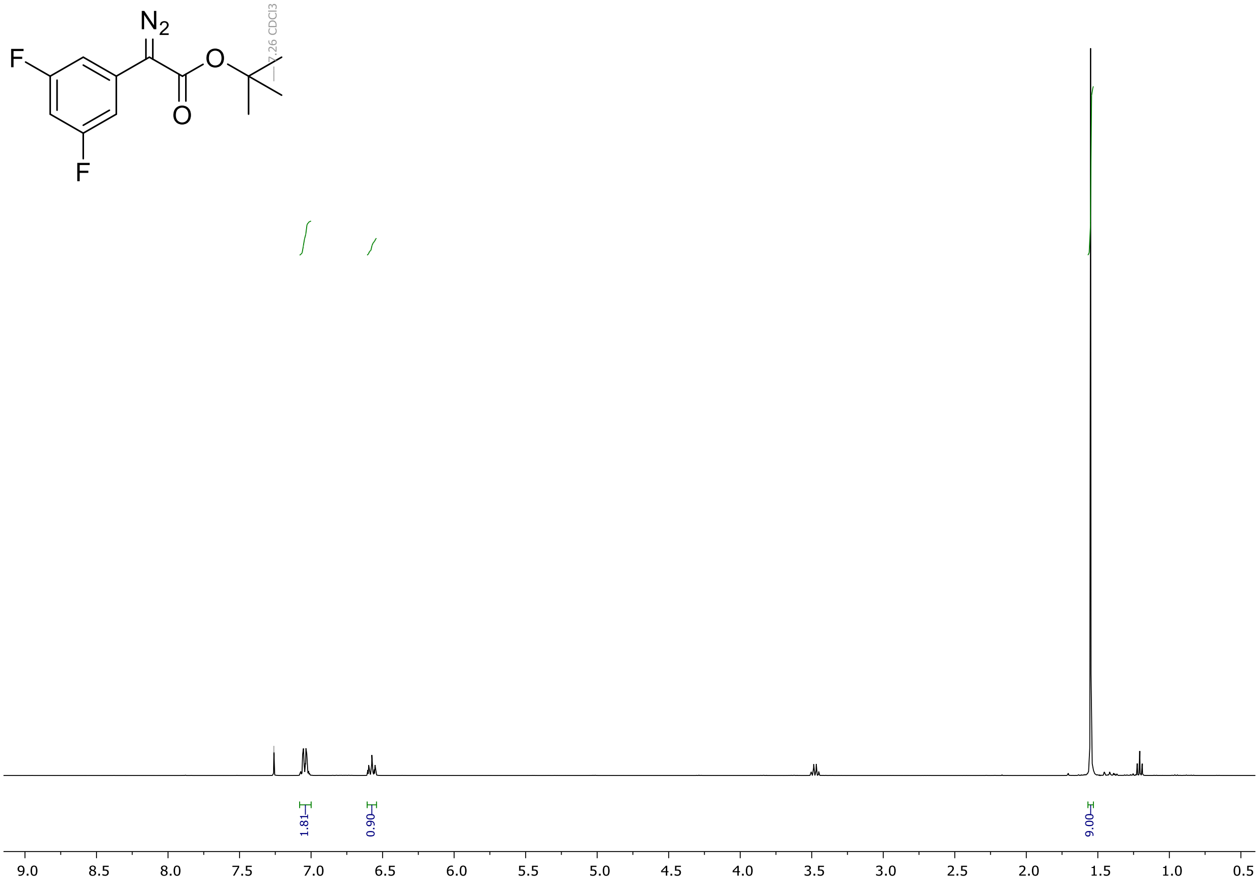
N2
F
F
O
O
7.26 CDCl3
1.81
0.90
9.00
9.0 8.5 8.0 7.5 7.0 6.5 6.0 5.5 5.0 4.5 4.0 3.5 3.0 2.5 2.0 1.5 1.0 0.5

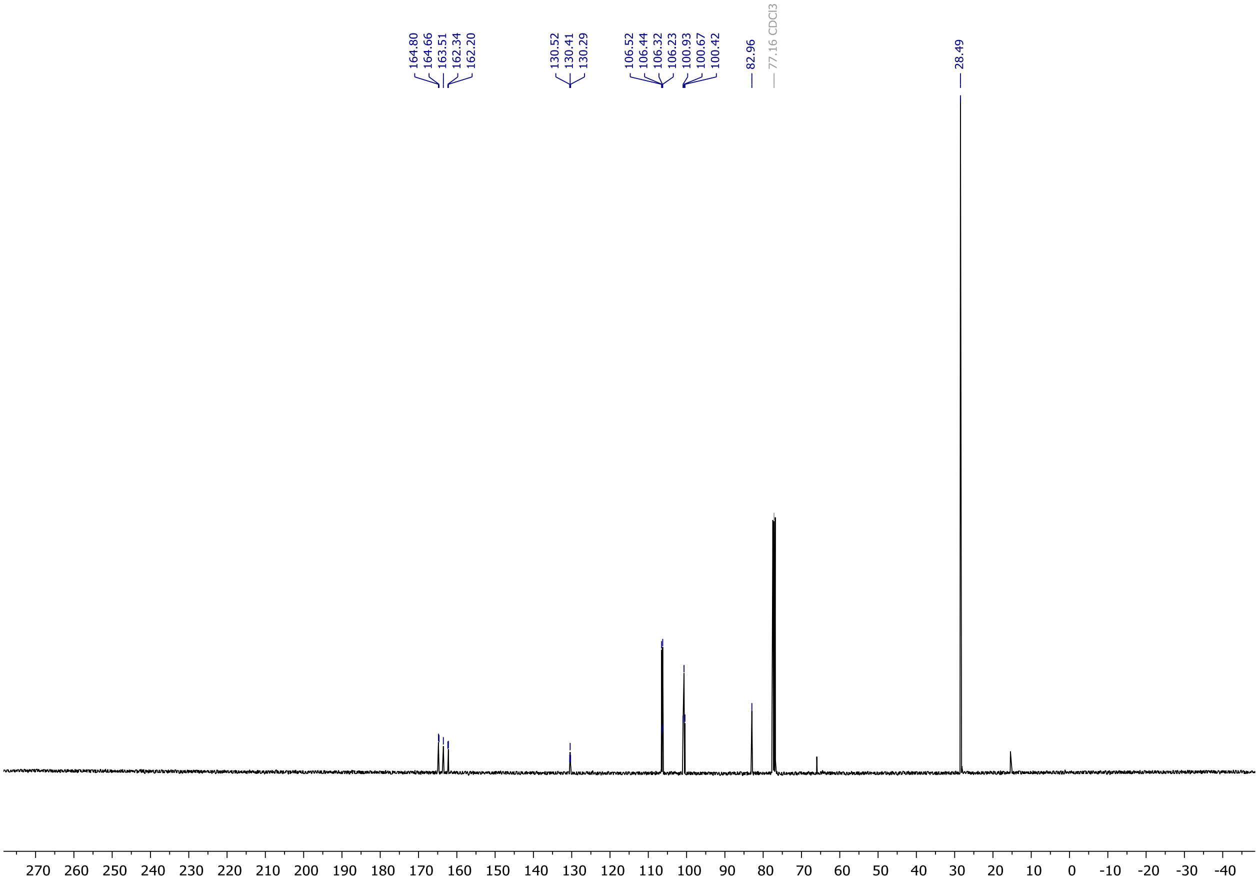
164.80
164.66
163.51
162.34
162.20
130.52
130.41
130.29
106.52
106.44
106.32
106.23
100.93
100.67
100.42
82.96
77.16 CDCl3
28.49
270 260 250 240 230 220 210 200 190 180 170 160 150 140 130 120 110 100 90 80 70 60 50 40 30 20 10 0 -10 -20 -30 -40

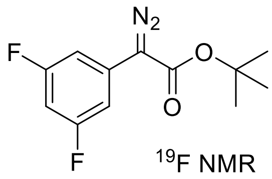

^{19}F NMR

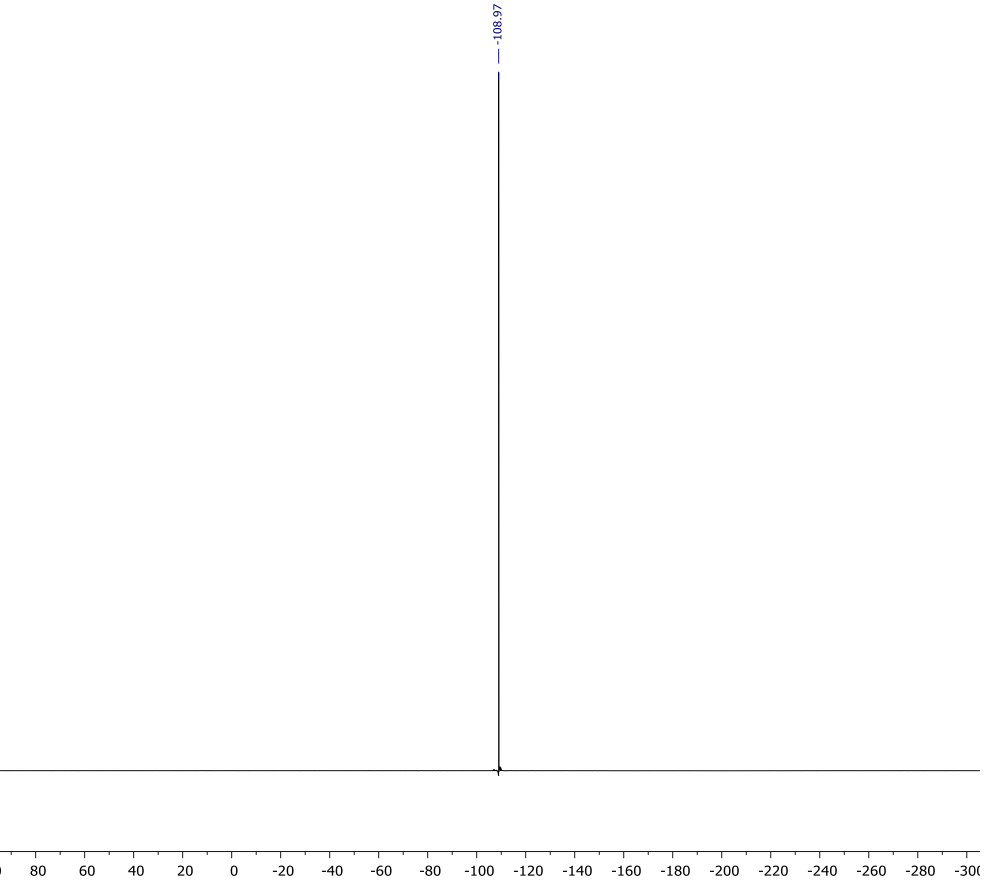
-108.97
200
180
160
140
120
100
80
60
40
20
0
-20
-40
-60
-80
-100
-120
-140
-160
-180
-200
-220
-240
-260
-280

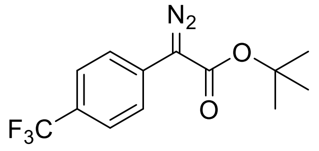
N2
O
O
F3C

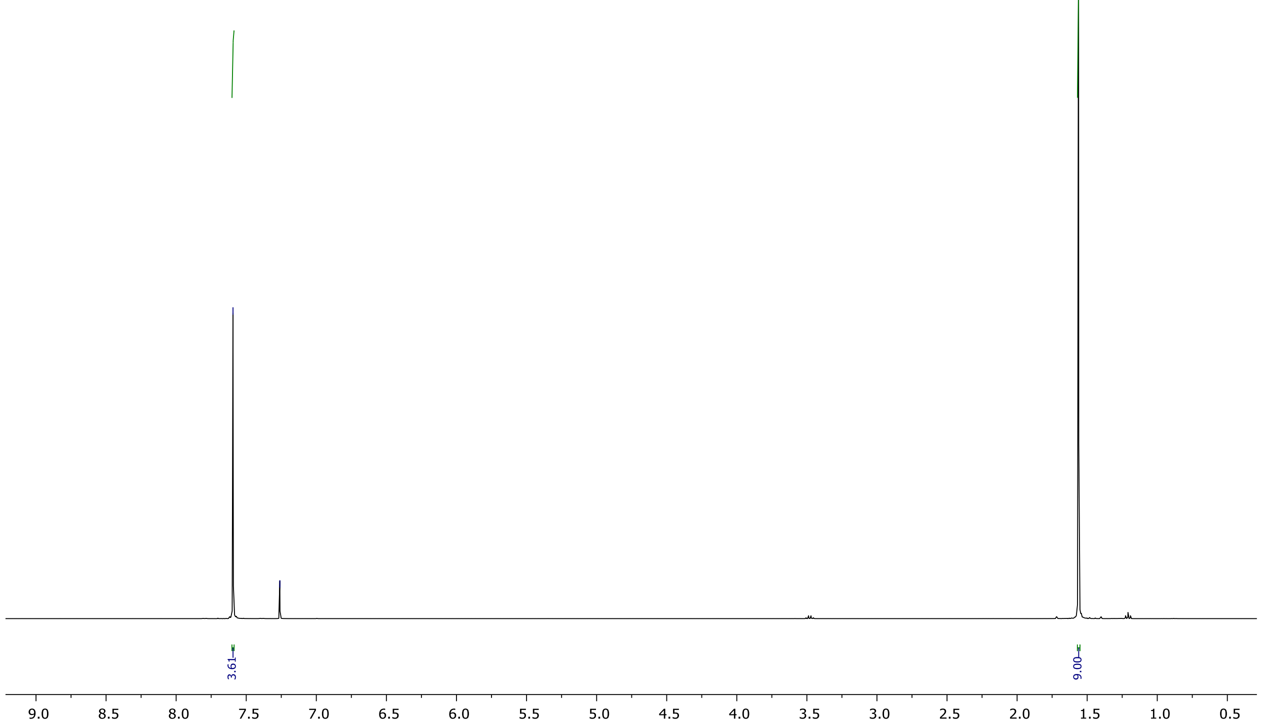
7.59
7.26 CDCl3
1.56
3.61
9.00
9.0 8.5 8.0 7.5 7.0 6.5 6.0 5.5 5.0 4.5 4.0 3.5 3.0 2.5 2.0 1.5 1.0 0.5

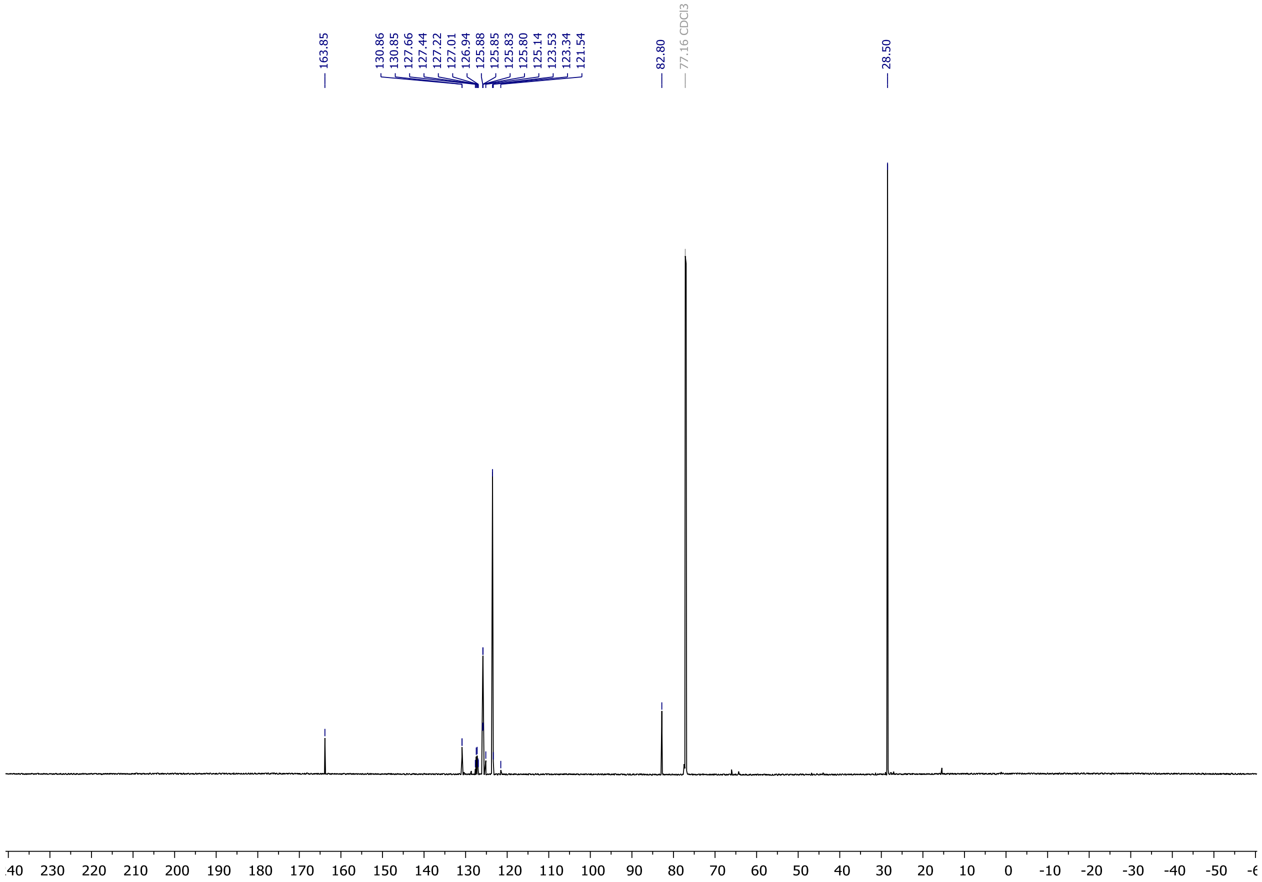
163.85
130.86
130.85
127.66
127.44
127.22
127.01
126.94
125.88
125.85
125.83
125.80
125.14
123.53
123.34
121.54
82.80
77.16 CDCl3
28.50
230 220 210 200 190 180 170 160 150 140 130 120 110 100 90 80 70 60 50 40 30 20 10 0 -10 -20 -30 -40 -50

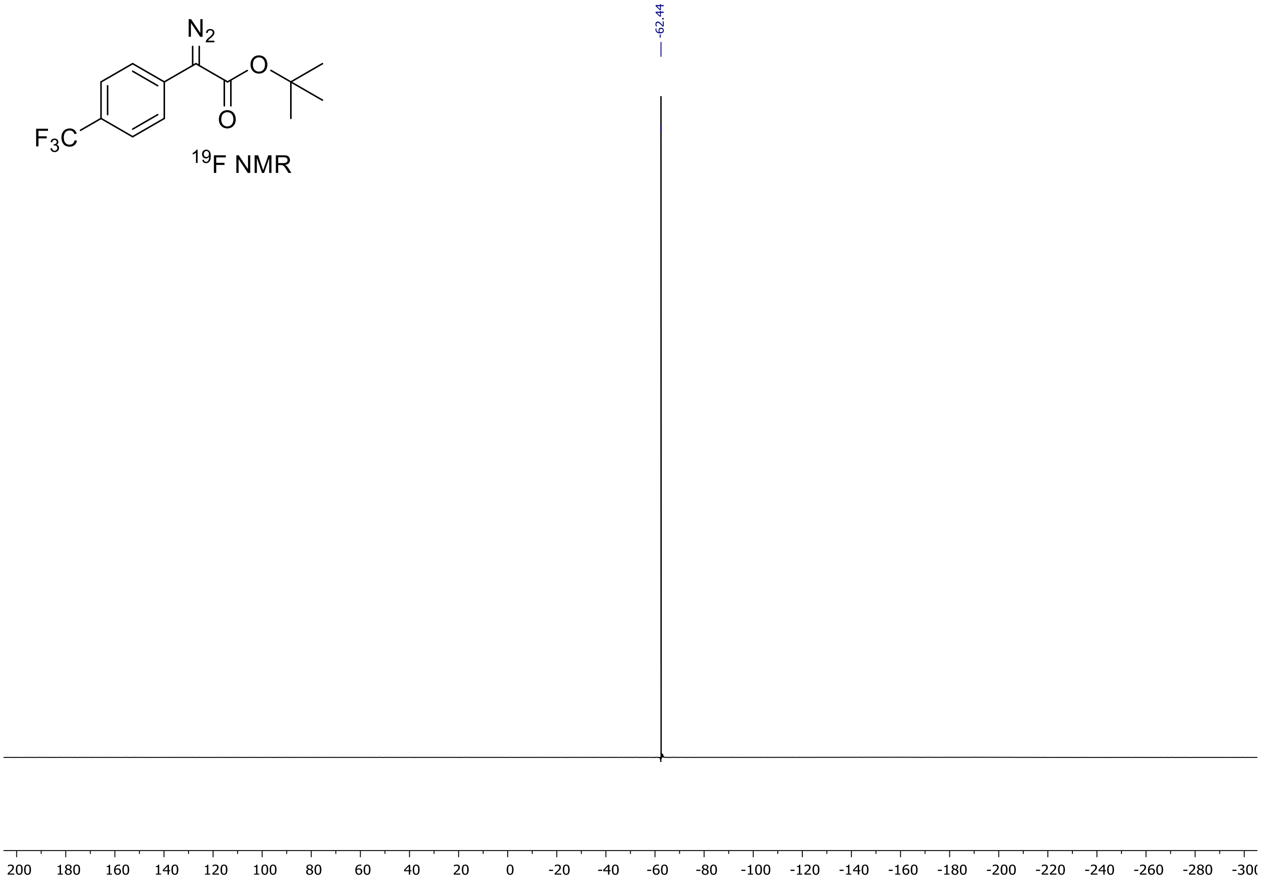
-62.44
^{19}F NMR
N_2
O
O
F_3C
200 180 160 140 120 100 80 60 40 20 0 -20 -40 -60 -80 -100 -120 -140 -160 -180 -200 -220 -240 -260 -280

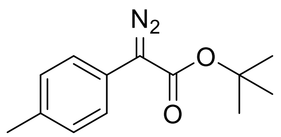

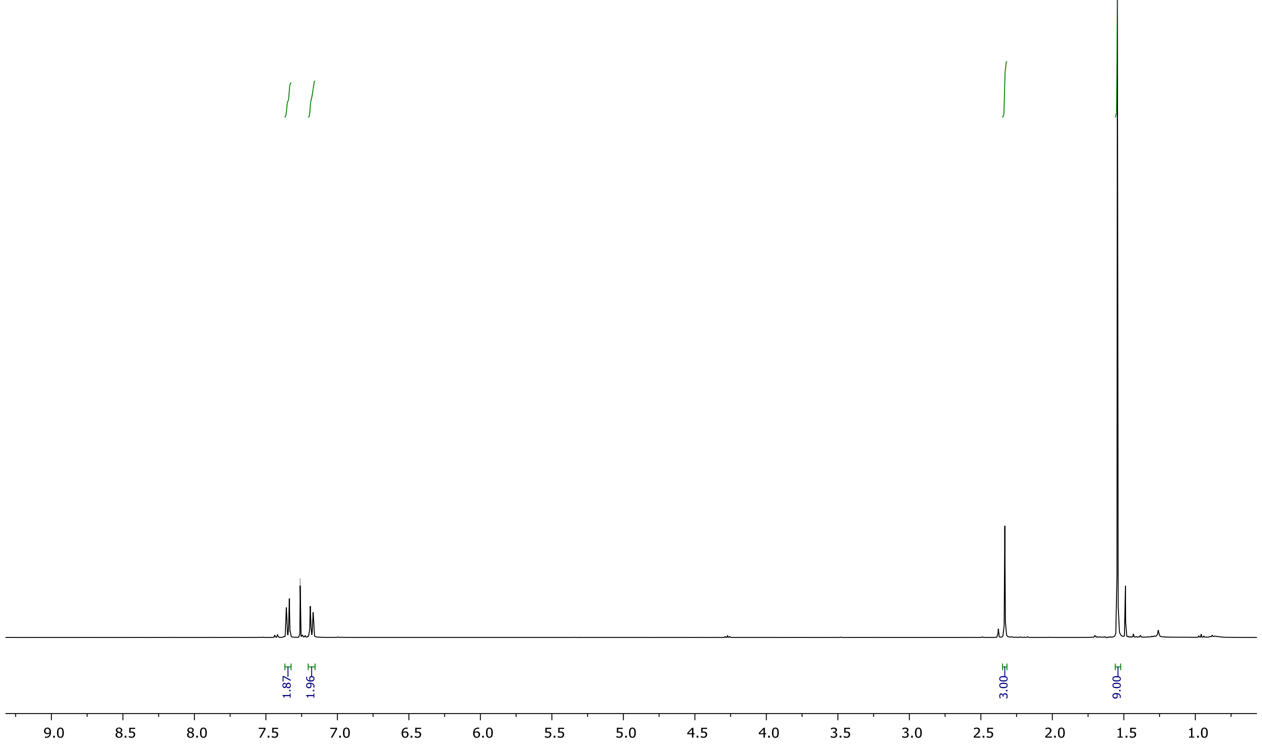
7.26 CDCl3
1.87
1.96
3.00
9.00
9.0 8.5 8.0 7.5 7.0 6.5 6.0 5.5 5.0 4.5 4.0 3.5 3.0 2.5 2.0 1.5 1.0

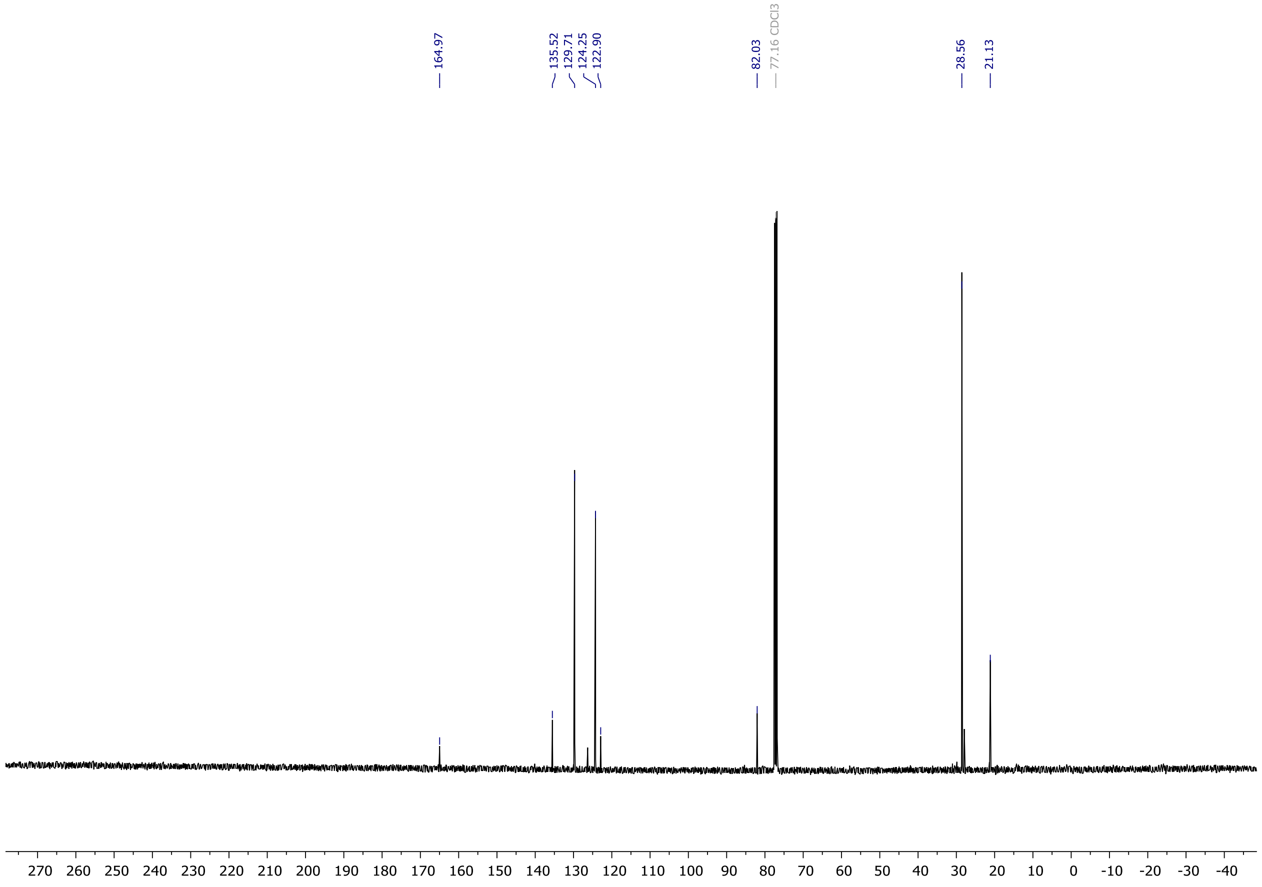
164.97
135.52
129.71
124.25
122.90
82.03
77.16 CDCl3
28.56
21.13
270 260 250 240 230 220 210 200 190 180 170 160 150 140 130 120 110 100 90 80 70 60 50 40 30 20 10 0 -10 -20 -30 -40

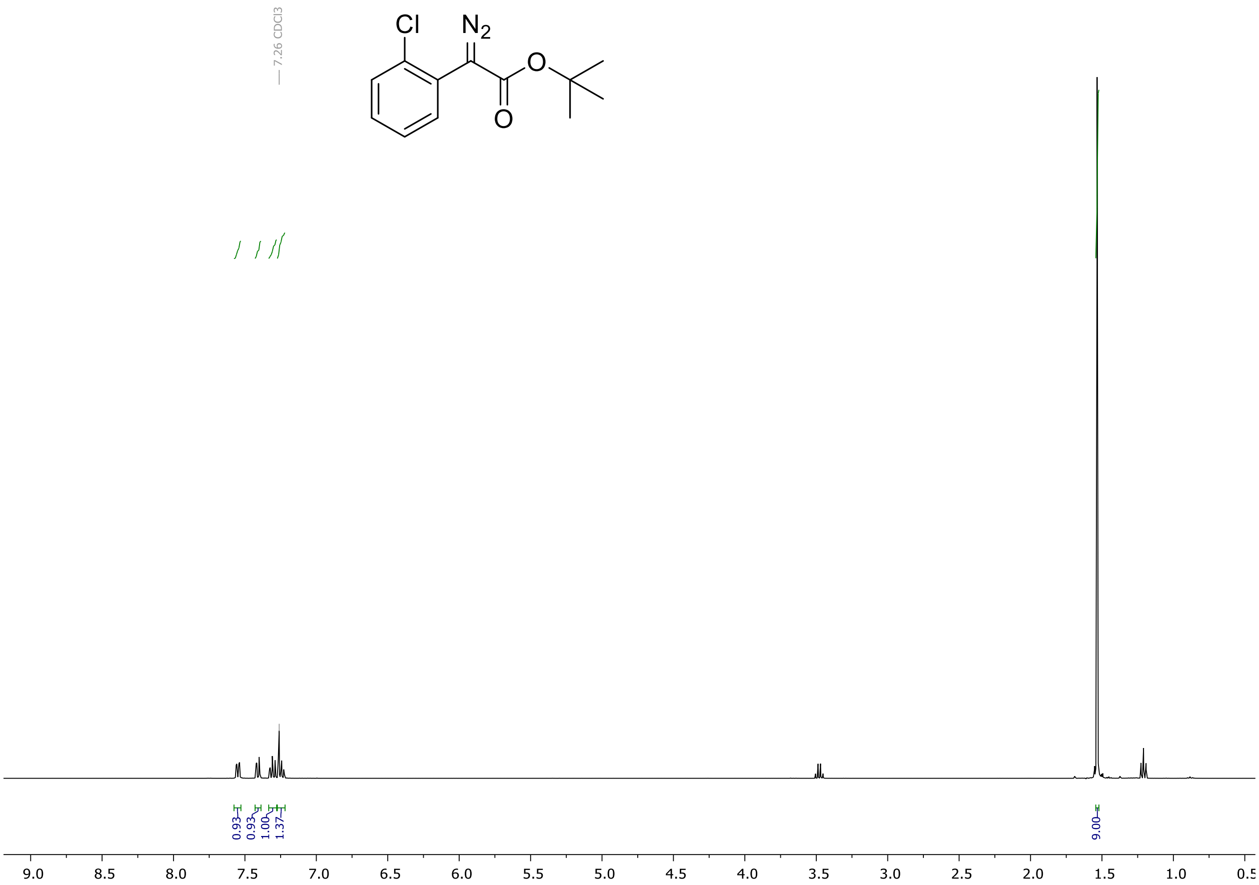
Cl
N2
O
O
7.26 CDCl3
0.93
0.93
1.00
1.37
9.00
9.0 8.5 8.0 7.5 7.0 6.5 6.0 5.5 5.0 4.5 4.0 3.5 3.0 2.5 2.0 1.5 1.0

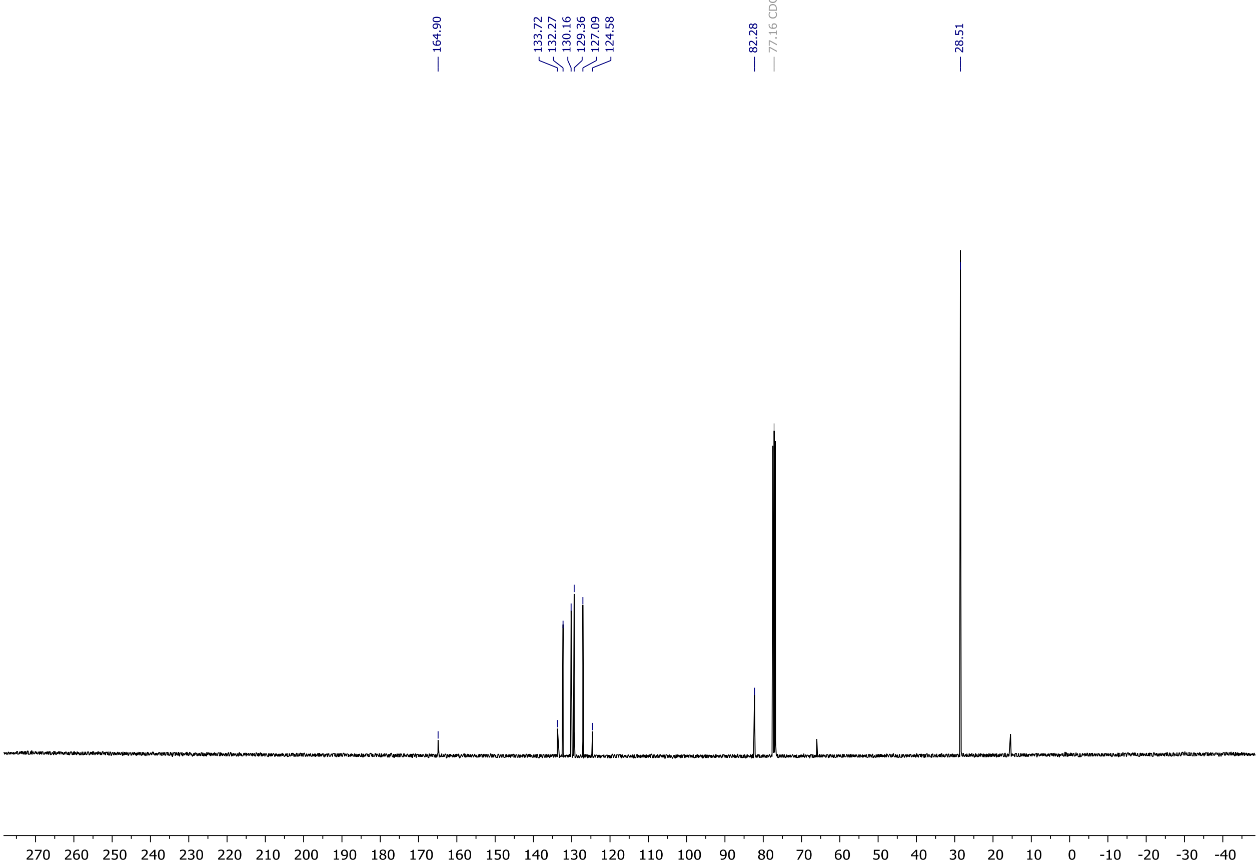
164.90
133.72
132.27
130.16
129.36
127.09
124.58
82.28
77.16 CDCl3
28.51
270 260 250 240 230 220 210 200 190 180 170 160 150 140 130 120 110 100 90 80 70 60 50 40 30 20 10 0 -10 -20 -30 -40

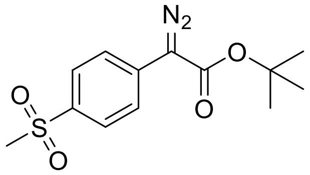

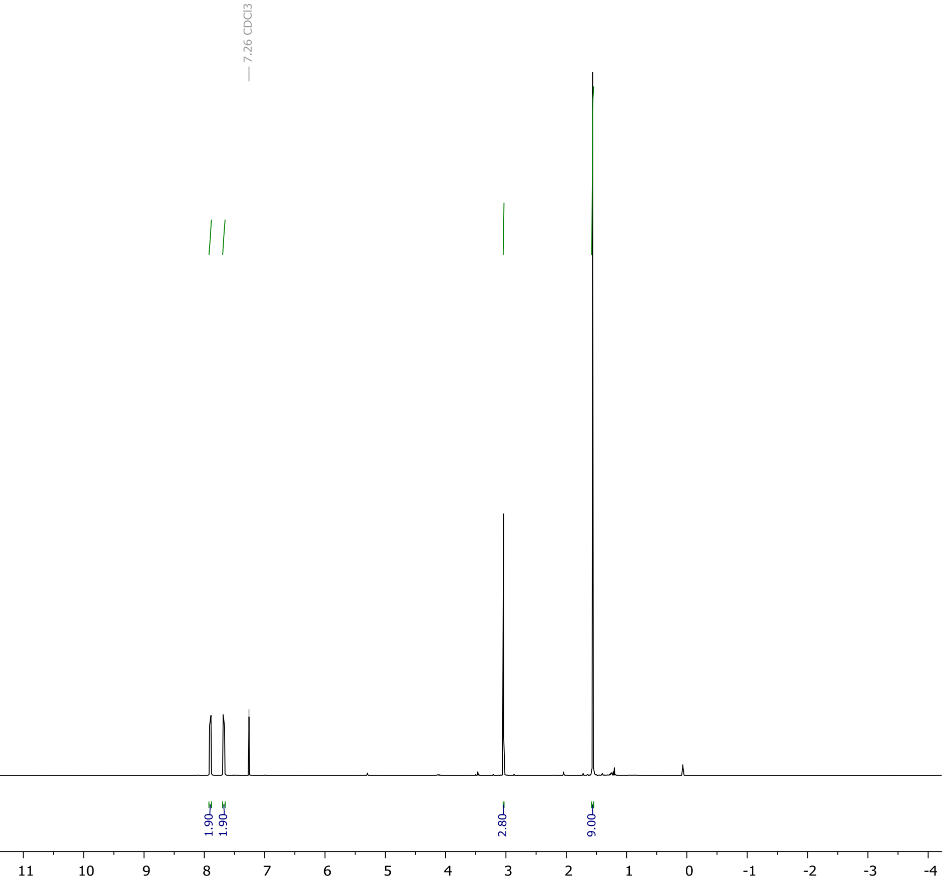
7.26 CDCl3
1.90
1.90
2.80
9.00
16 15 14 13 12 11 10 9 8 7 6 5 4 3 2 1 0 -1 -2 -3 -4

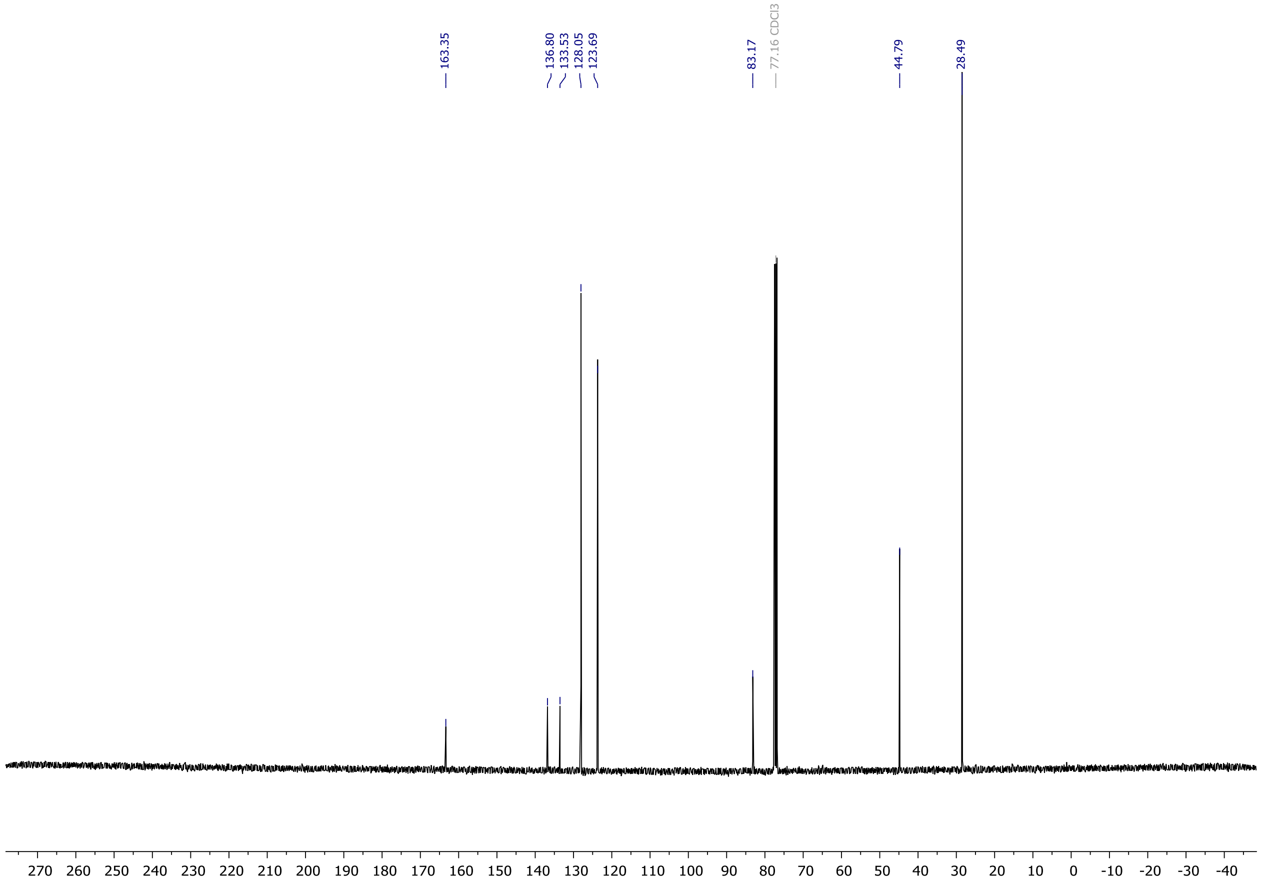
163.35
136.80
133.53
128.05
123.69
83.17
77.16 CDCl3
44.79
28.49
270 260 250 240 230 220 210 200 190 180 170 160 150 140 130 120 110 100 90 80 70 60 50 40 30 20 10 0 -10 -20 -30 -40

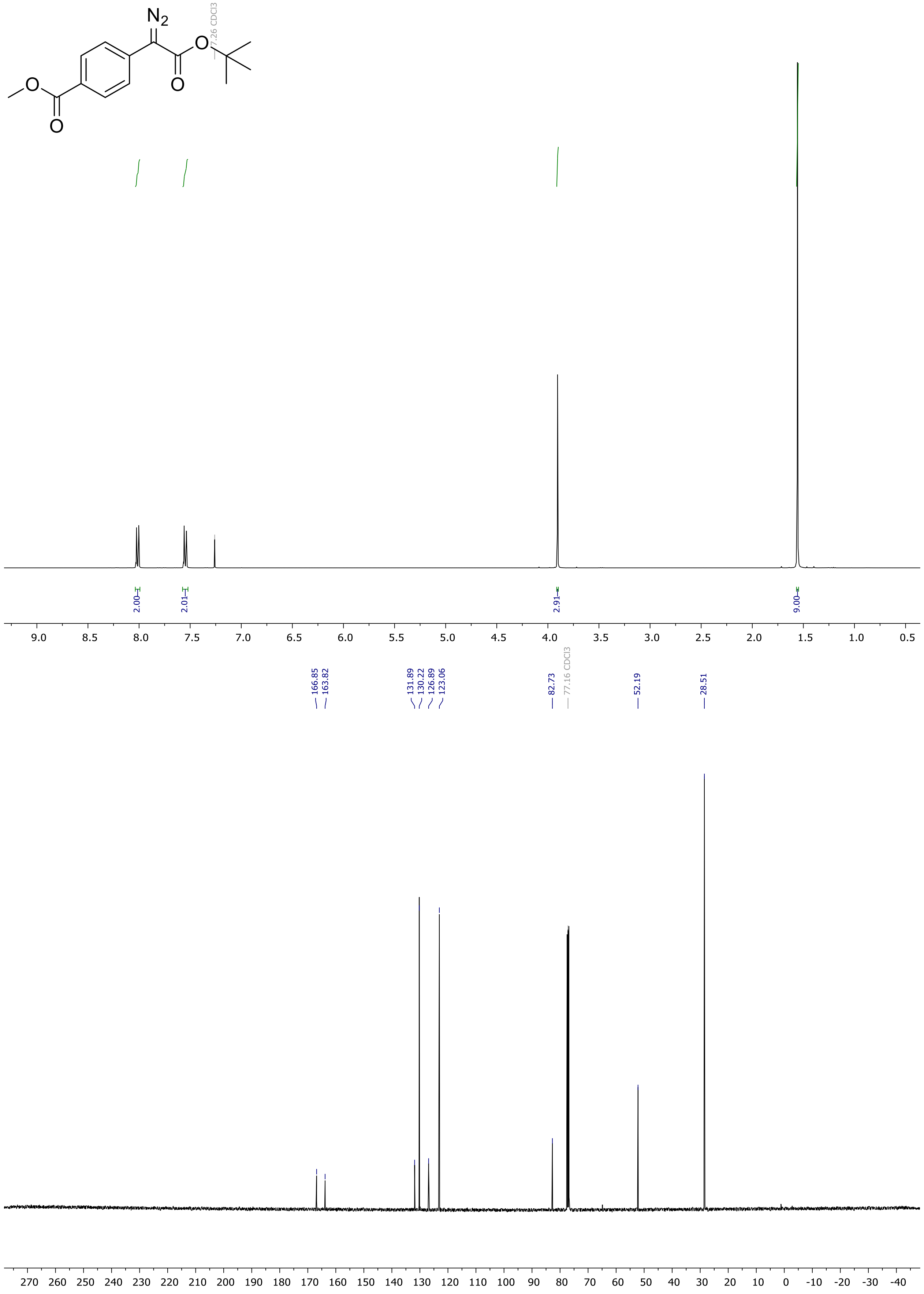
N2
O
O
O
O
7.26 CDCl3
2.00
2.01
2.91
9.00
9.0 8.5 8.0 7.5 7.0 6.5 6.0 5.5 5.0 4.5 4.0 3.5 3.0 2.5 2.0 1.5 1.0 0.5
166.85
163.82
131.89
130.22
126.89
123.06
82.73
77.16 CDCl3
52.19
28.51
270 260 250 240 230 220 210 200 190 180 170 160 150 140 130 120 110 100 90 80 70 60 50 40 30 20 10 0 -10 -20 -30 -40

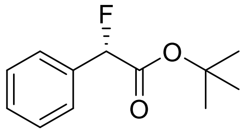

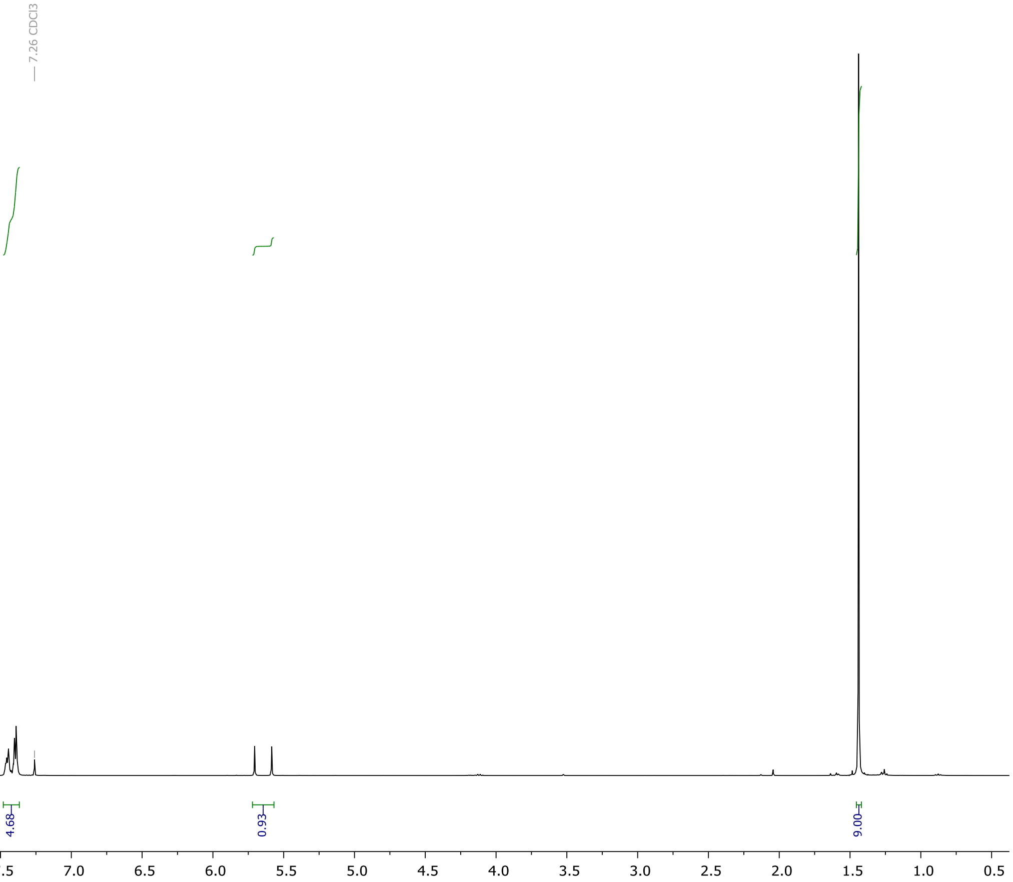

7.26 CDCl3
4.68
0.93
9.00
9.0 8.5 8.0 7.5 7.0 6.5 6.0 5.5 5.0 4.5 4.0 3.5 3.0 2.5 2.0 1.5 1.0 0.5

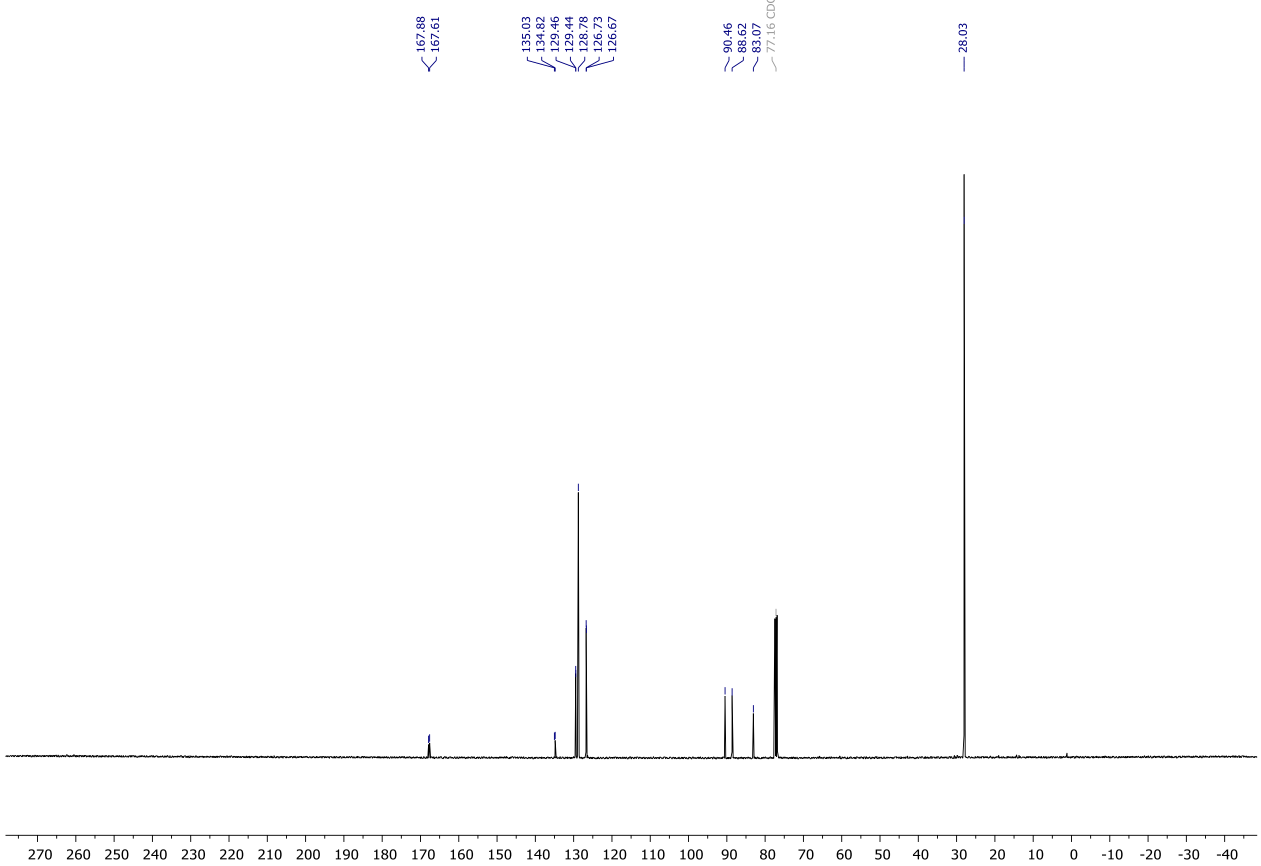

167.88
167.61
135.03
134.82
129.46
129.44
128.78
126.73
126.67
90.46
88.62
83.07
77.16 CDCl3
28.03
270 260 250 240 230 220 210 200 190 180 170 160 150 140 130 120 110 100 90 80 70 60 50 40 30 20 10 0 -10 -20 -30 -40

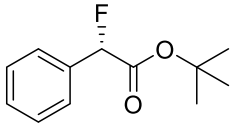

^{19}F NMR

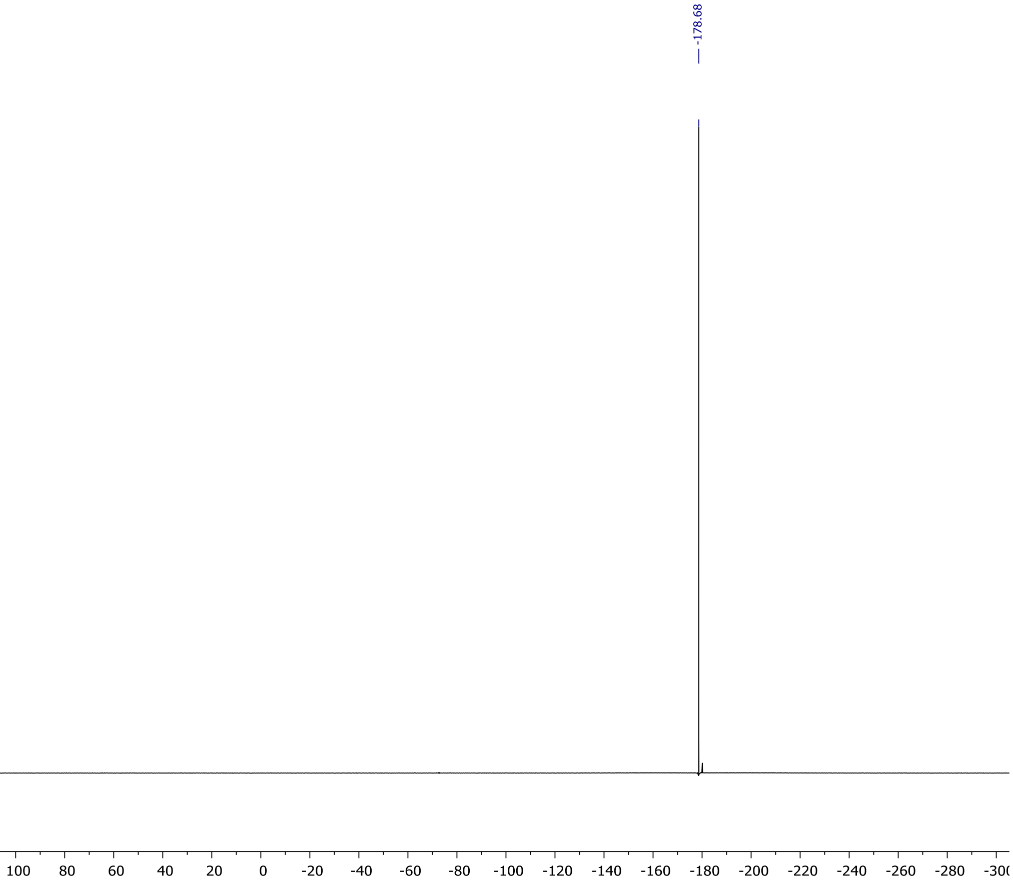

-178.68
200 180 160 140 120 100 80 60 40 20 0 -20 -40 -60 -80 -100 -120 -140 -160 -180 -200 -220 -240 -260 -280 -300

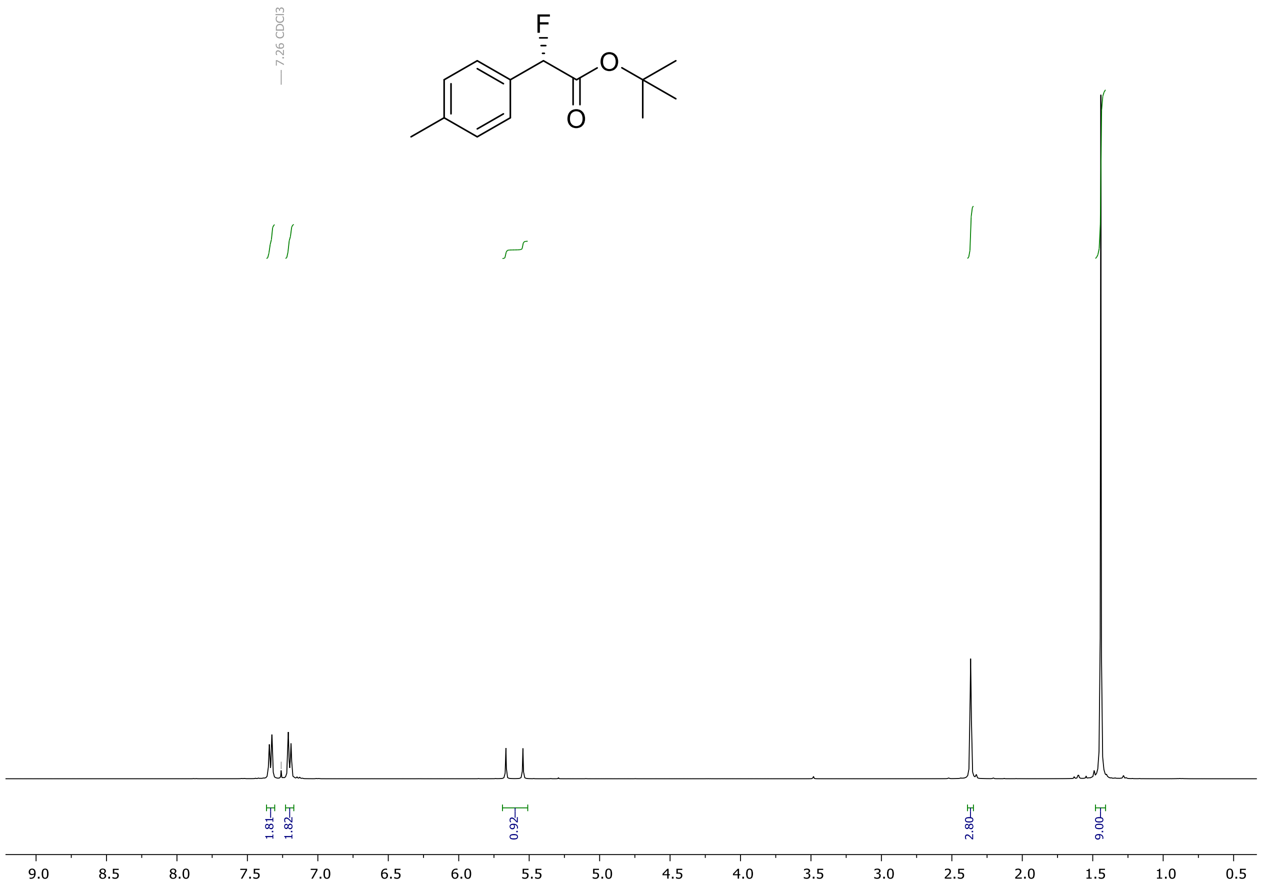

F
O
O
7.26 CDCl3
1.81
1.82
0.92
2.80
9.00
9.0 8.5 8.0 7.5 7.0 6.5 6.0 5.5 5.0 4.5 4.0 3.5 3.0 2.5 2.0 1.5 1.0 0.5

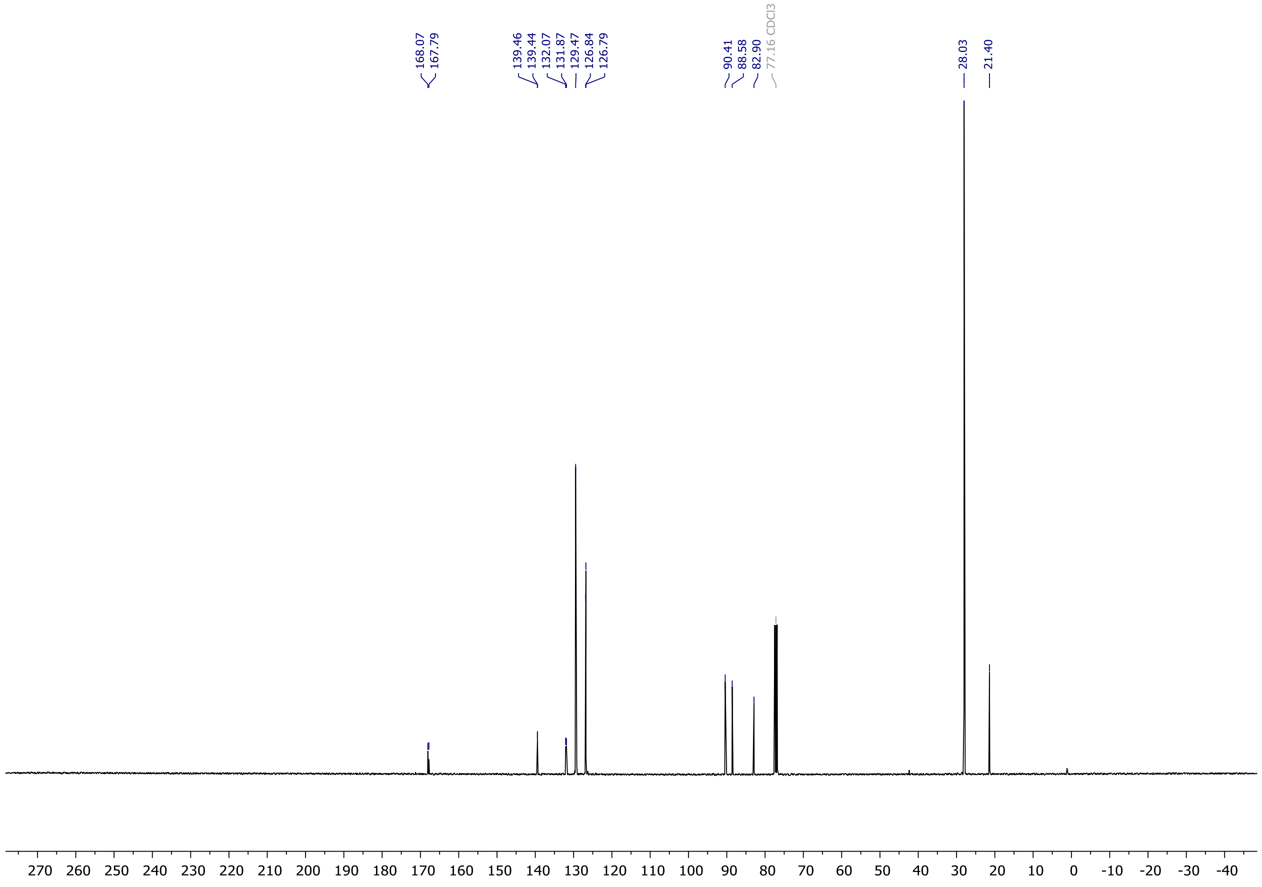

168.07
167.79
139.46
139.44
132.07
131.87
129.47
126.84
126.79
90.41
88.58
82.90
77.16 CDCl3
28.03
21.40
270 260 250 240 230 220 210 200 190 180 170 160 150 140 130 120 110 100 90 80 70 60 50 40 30 20 10 0 -10 -20 -30 -40

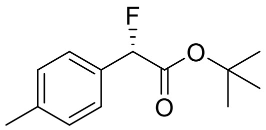

^{19}F NMR

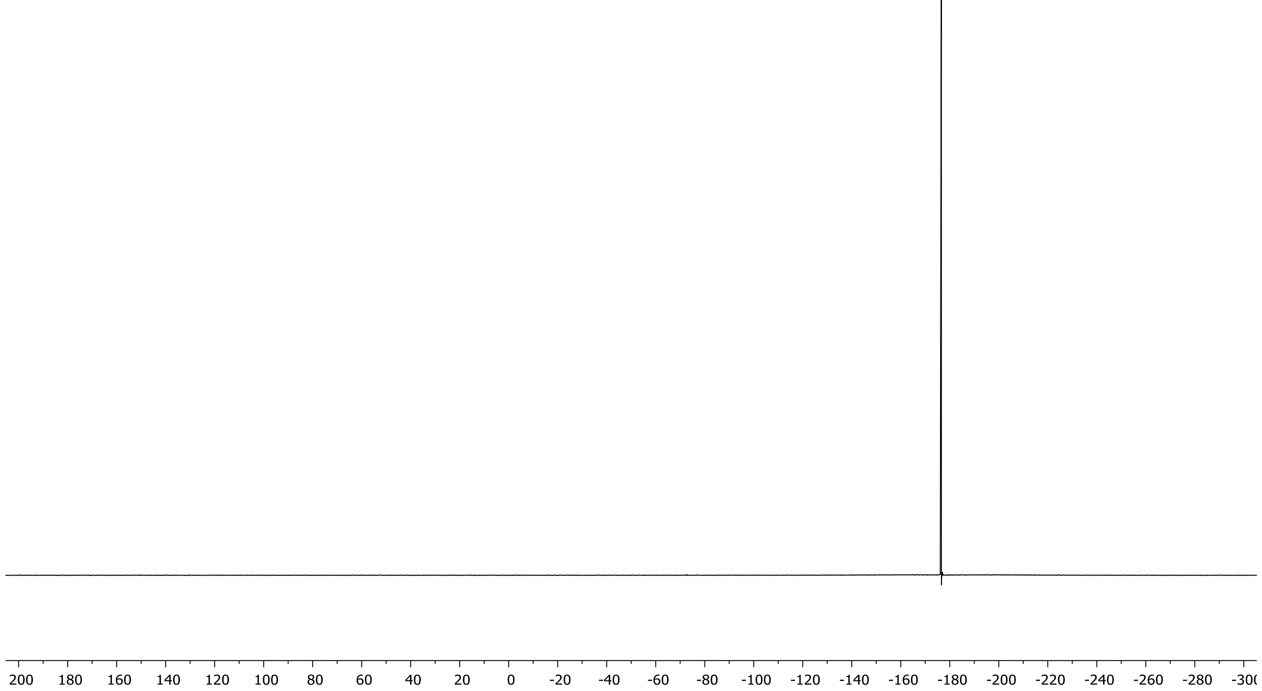

-176.58
200 180 160 140 120 100 80 60 40 20 0 -20 -40 -60 -80 -100 -120 -140 -160 -180 -200 -220 -240 -260 -280 -300

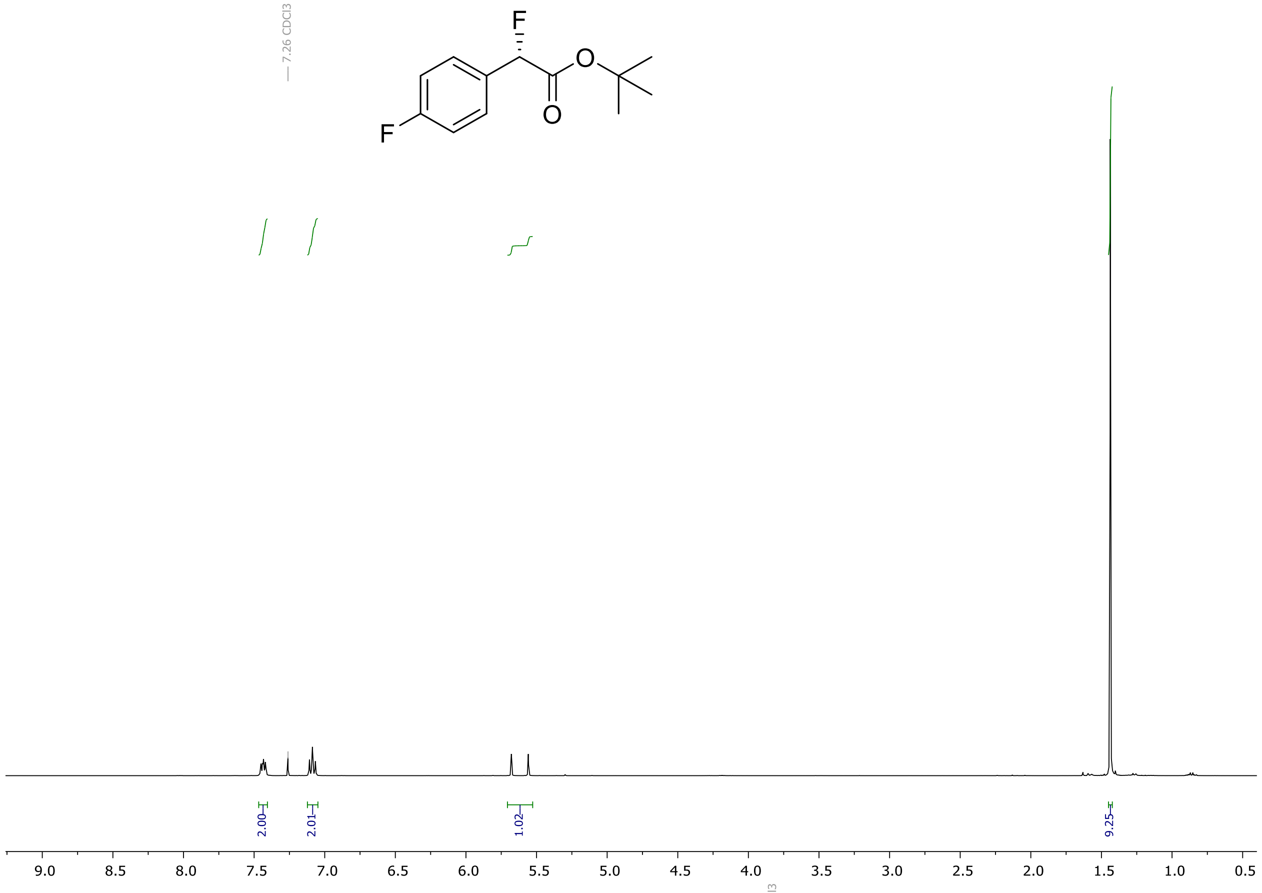
F
O
O
F
7.26 CDCl3
2.00
2.01
1.02
9.25
9.0 8.5 8.0 7.5 7.0 6.5 6.0 5.5 5.0 4.5 4.0 3.5 3.0 2.5 2.0 1.5 1.0 0.5

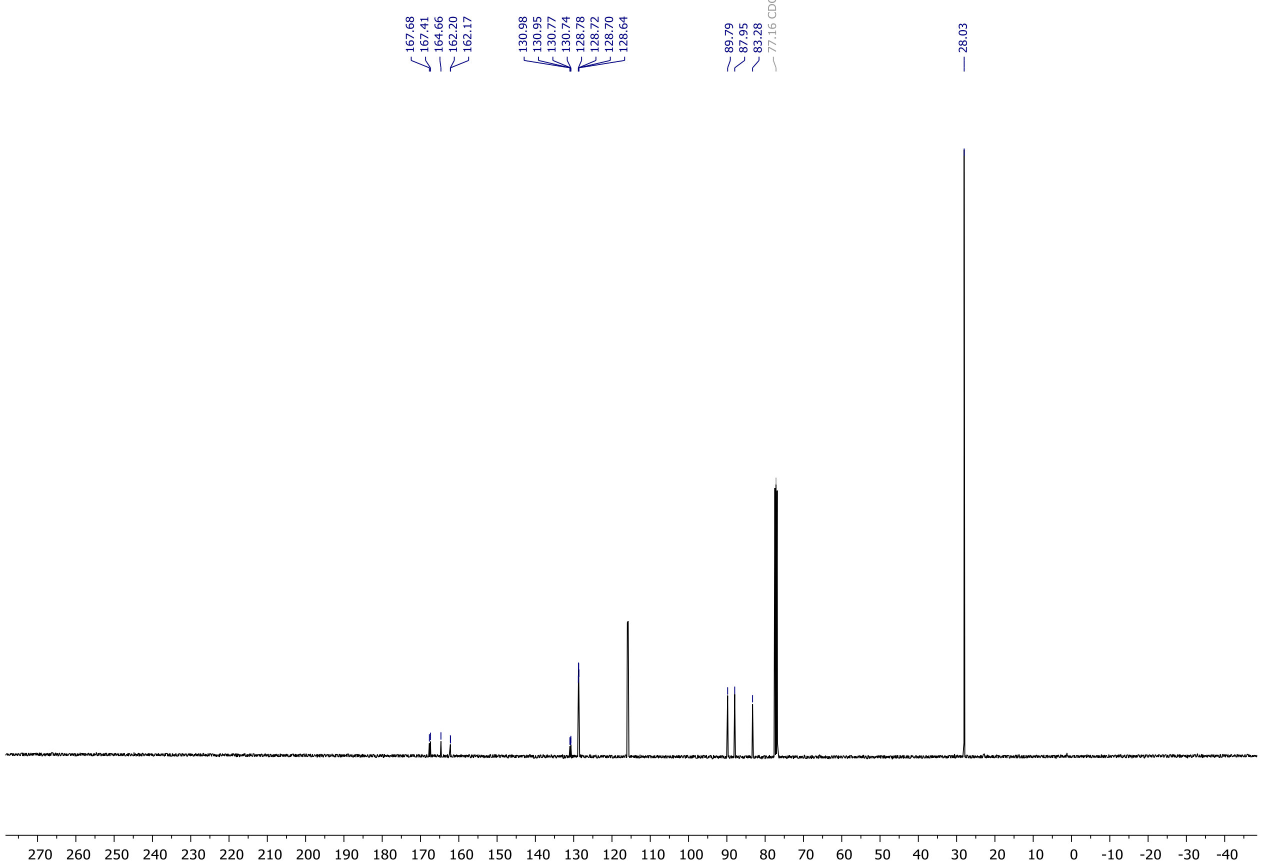
167.68
167.41
164.66
162.20
162.17
130.98
130.95
130.77
130.74
128.78
128.72
128.70
128.64
89.79
87.95
83.28
77.16 CDCl3
28.03
270 260 250 240 230 220 210 200 190 180 170 160 150 140 130 120 110 100 90 80 70 60 50 40 30 20 10 0 -10 -20 -30 -40

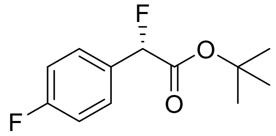

^{19}F NMR

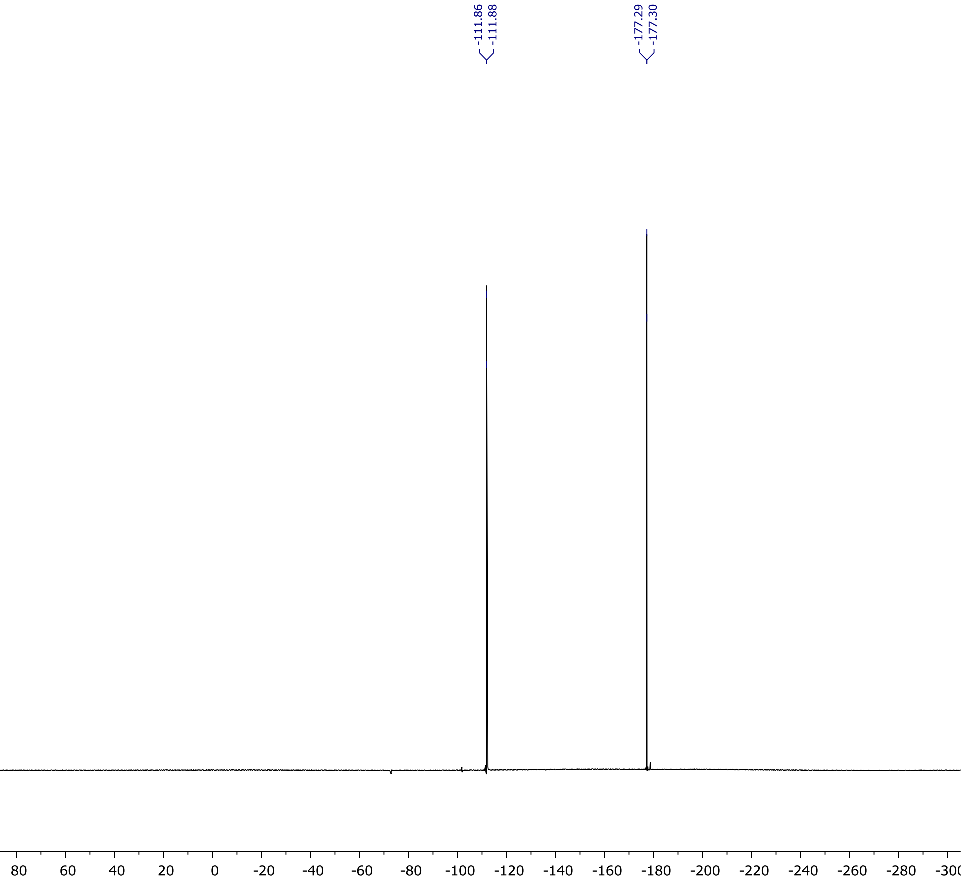

-111.86
-111.88
-177.29
-177.30
200 180 160 140 120 100 80 60 40 20 0 -20 -40 -60 -80 -100 -120 -140 -160 -180 -200 -220 -240 -260 -280

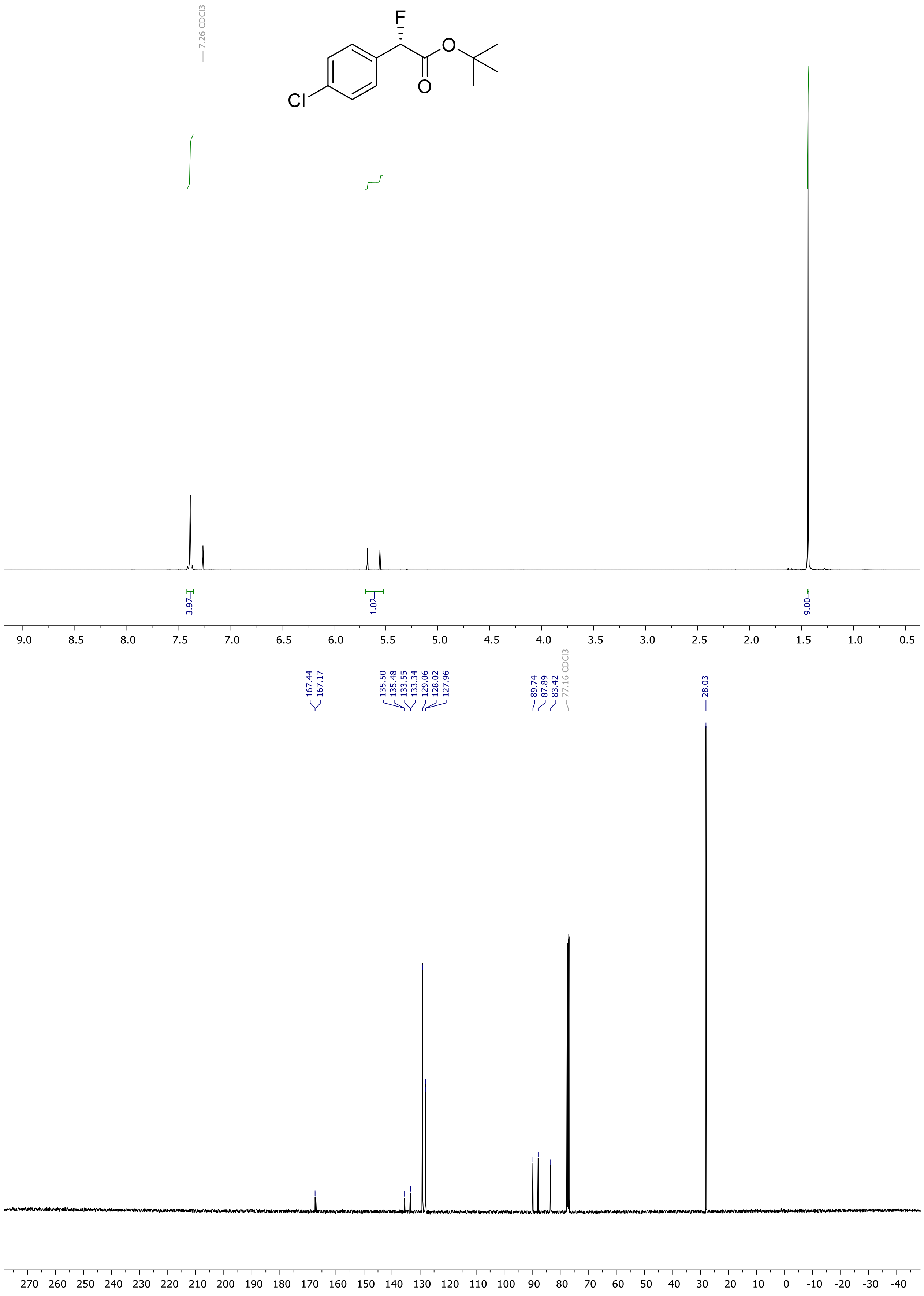
F
O
O
Cl
7.26 CDCl3
3.97
1.02
9.00
9.0 8.5 8.0 7.5 7.0 6.5 6.0 5.5 5.0 4.5 4.0 3.5 3.0 2.5 2.0 1.5 1.0 0.5
167.44
167.17
135.50
135.48
133.55
133.34
129.06
128.02
127.96
89.74
87.89
83.42
77.16 CDCl3
28.03
270 260 250 240 230 220 210 200 190 180 170 160 150 140 130 120 110 100 90 80 70 60 50 40 30 20 10 0 -10 -20 -30 -40

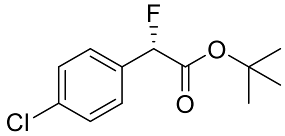

^{19}F NMR

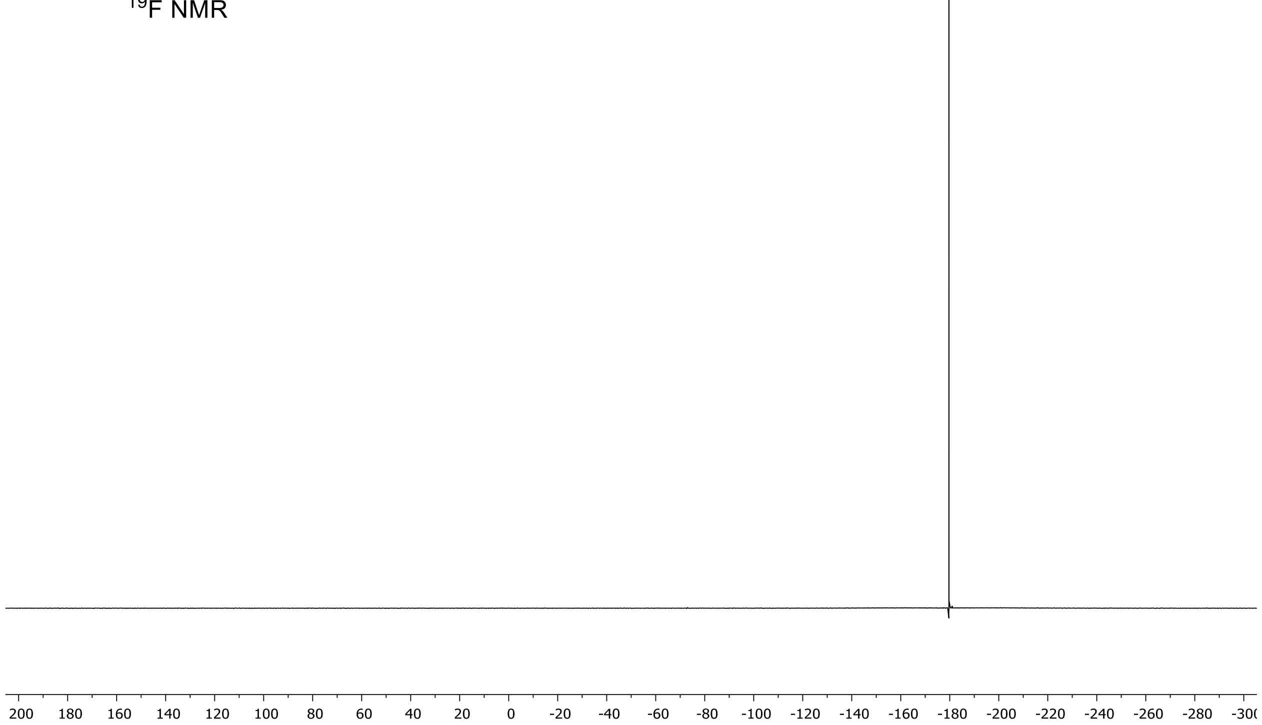

-179.66
200 180 160 140 120 100 80 60 40 20 0 -20 -40 -60 -80 -100 -120 -140 -160 -180 -200 -220 -240 -260 -280 -300

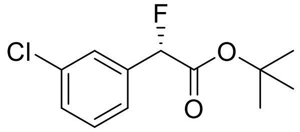

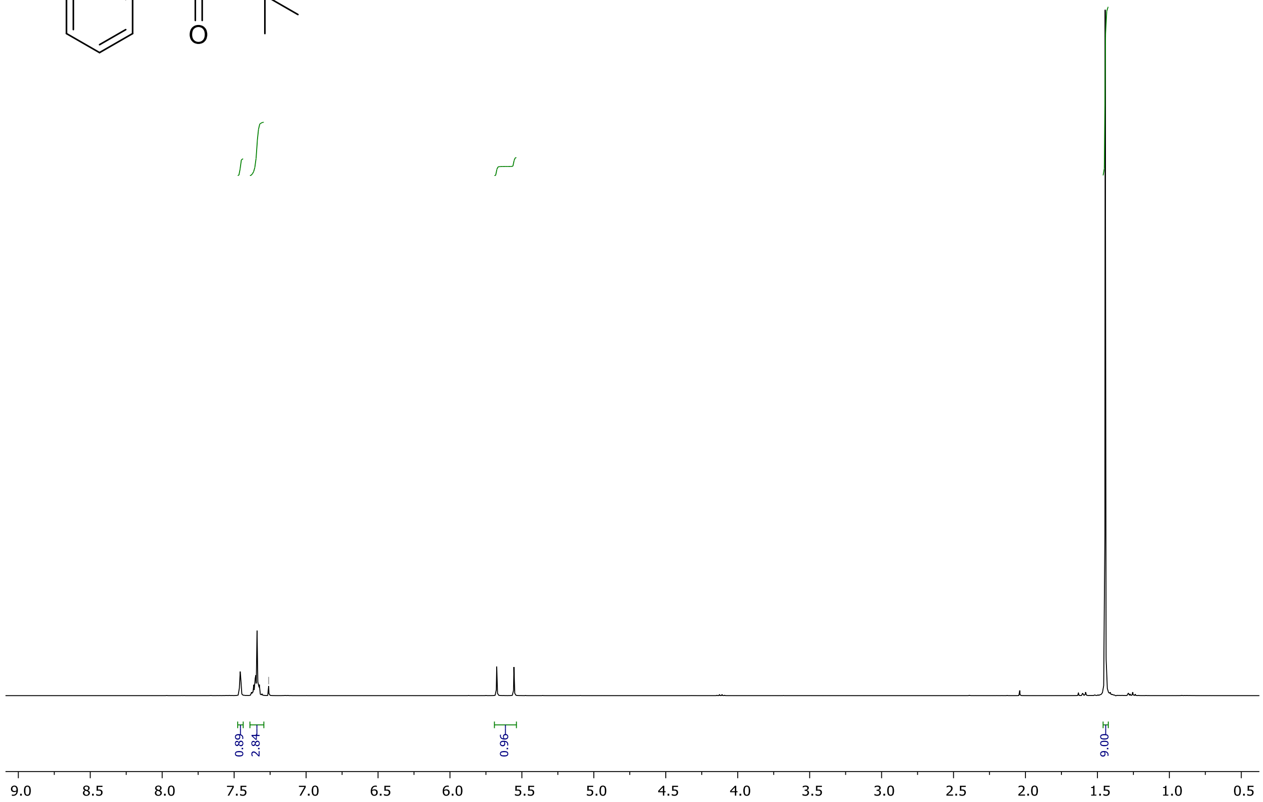
0.89
2.84
0.96
9.00
9.0 8.5 8.0 7.5 7.0 6.5 6.0 5.5 5.0 4.5 4.0 3.5 3.0 2.5 2.0 1.5 1.0 0.5

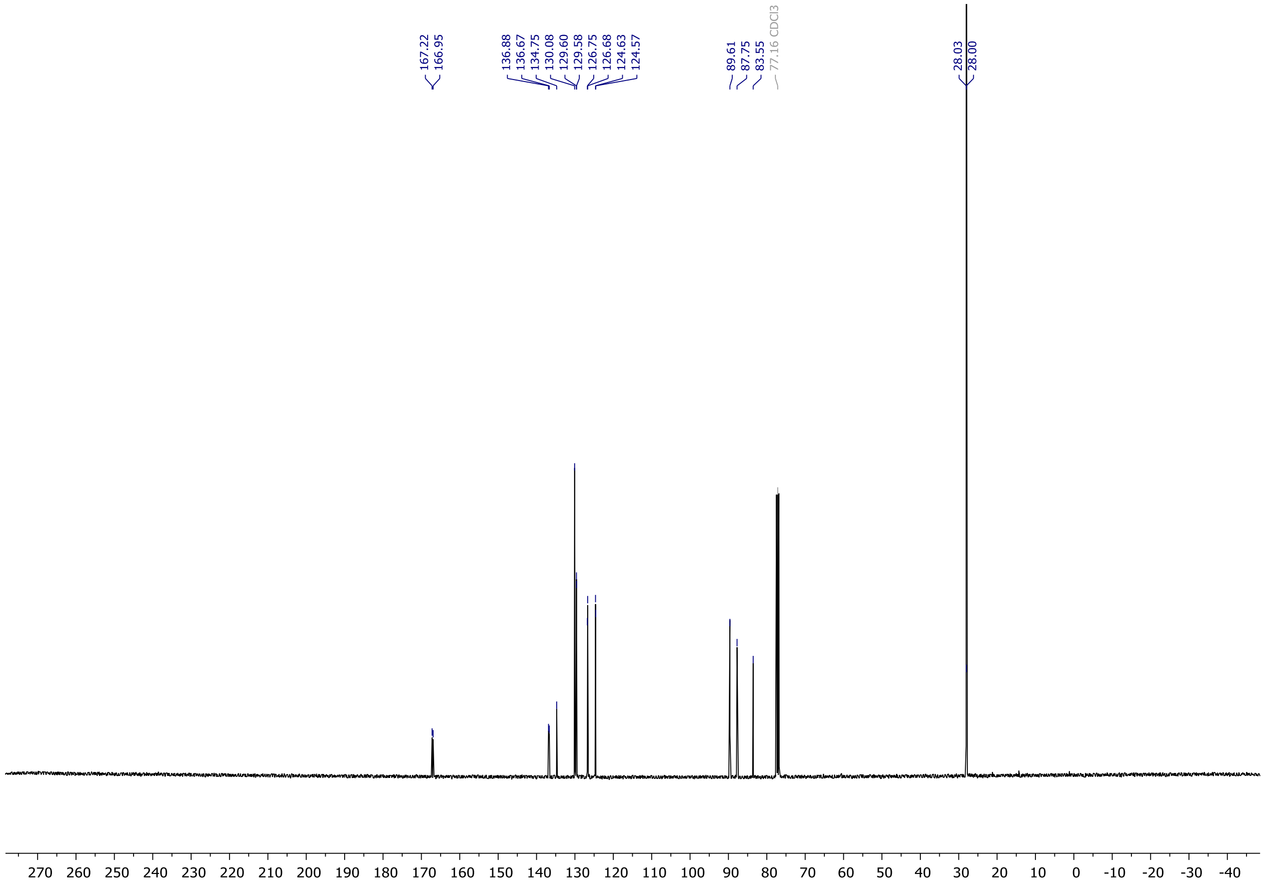
167.22
166.95
136.88
136.67
134.75
130.08
129.60
129.58
126.75
126.68
124.63
124.57
89.61
87.75
83.55
77.16 CDCl3
28.03
28.00
270 260 250 240 230 220 210 200 190 180 170 160 150 140 130 120 110 100 90 80 70 60 50 40 30 20 10 0 -10 -20 -30 -40

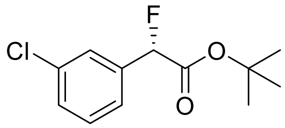

F
Cl
O
O

^{19}F NMR

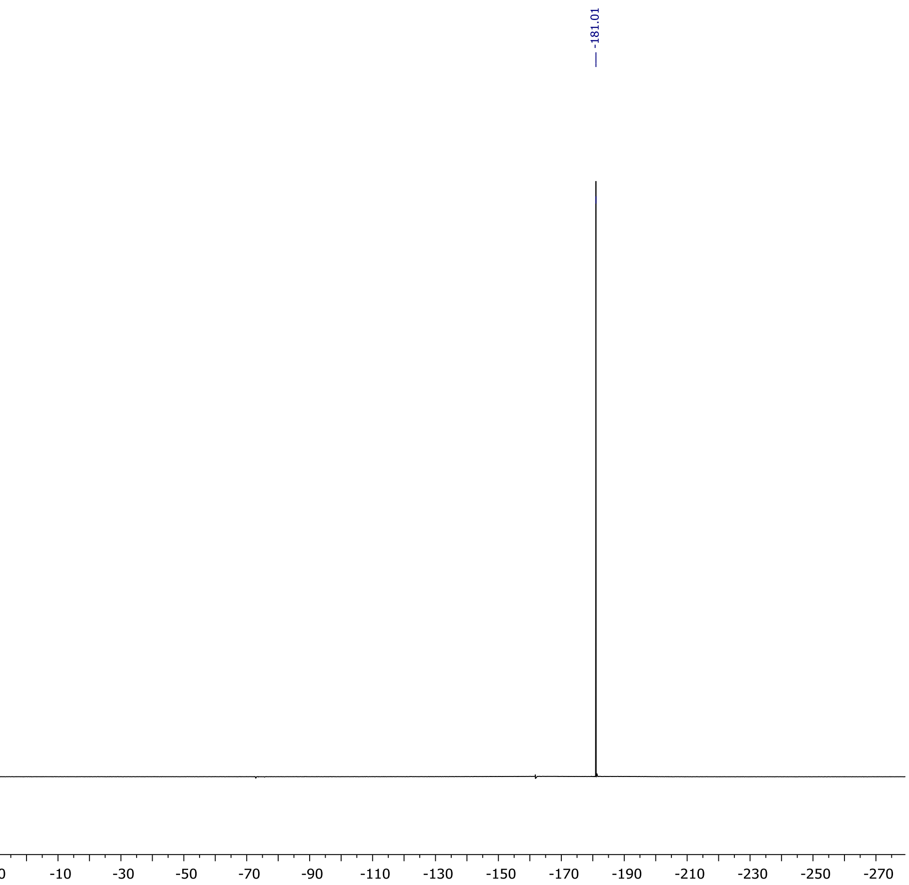

-181.01
110
90
70
50
30
10
-10
-30
-50
-70
-90
-110
-130
-150
-170
-190
-210
-230
-250
-270

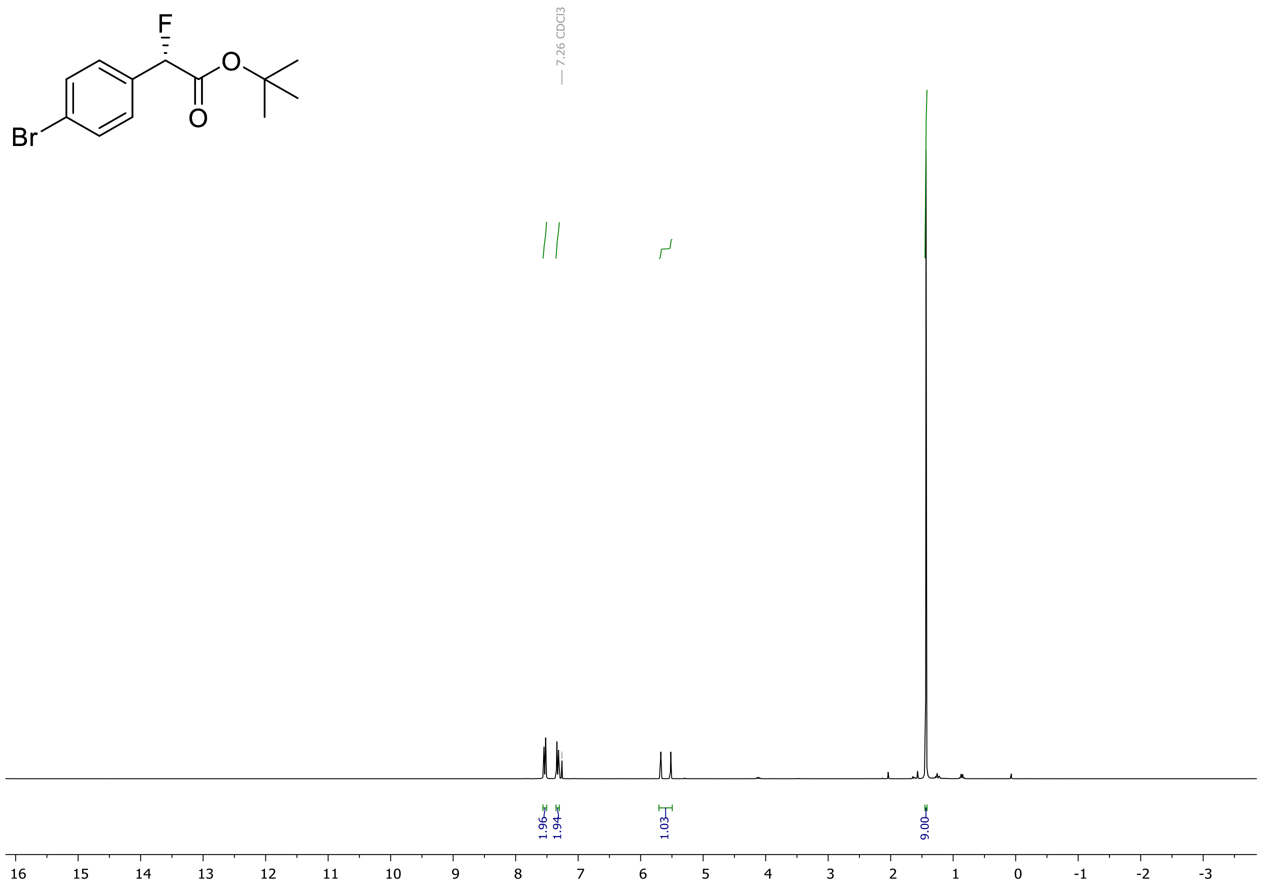
F
O
O
Br
7.26 CDCl3
1.96
1.94
1.03
9.00
16 15 14 13 12 11 10 9 8 7 6 5 4 3 2 1 0 -1 -2 -3

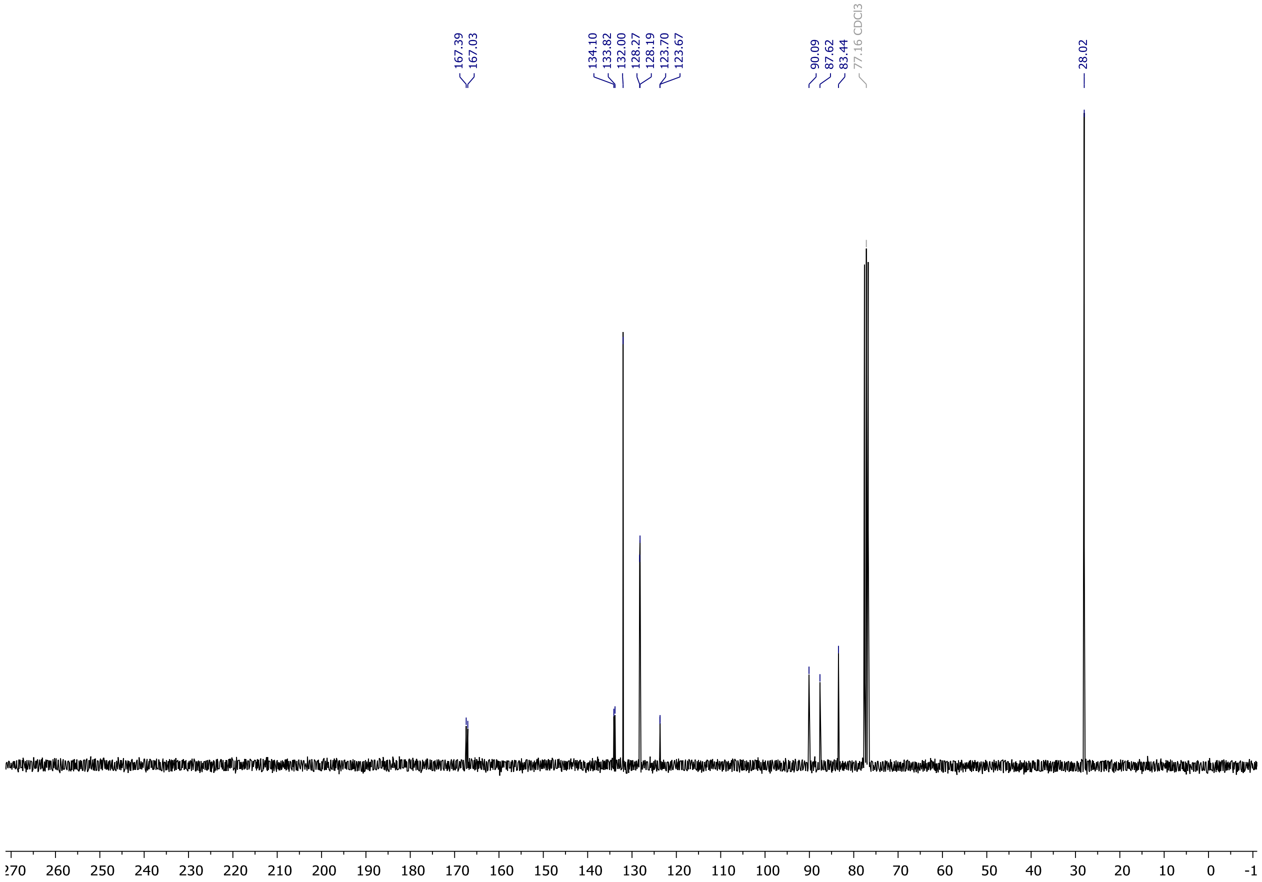
167.39
167.03
134.10
133.82
132.00
128.27
128.19
123.70
123.67
90.09
87.62
83.44
77.16 CDCl3
28.02
270 260 250 240 230 220 210 200 190 180 170 160 150 140 130 120 110 100 90 80 70 60 50 40 30 20 10 0 -1

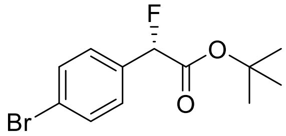

^{19}F NMR

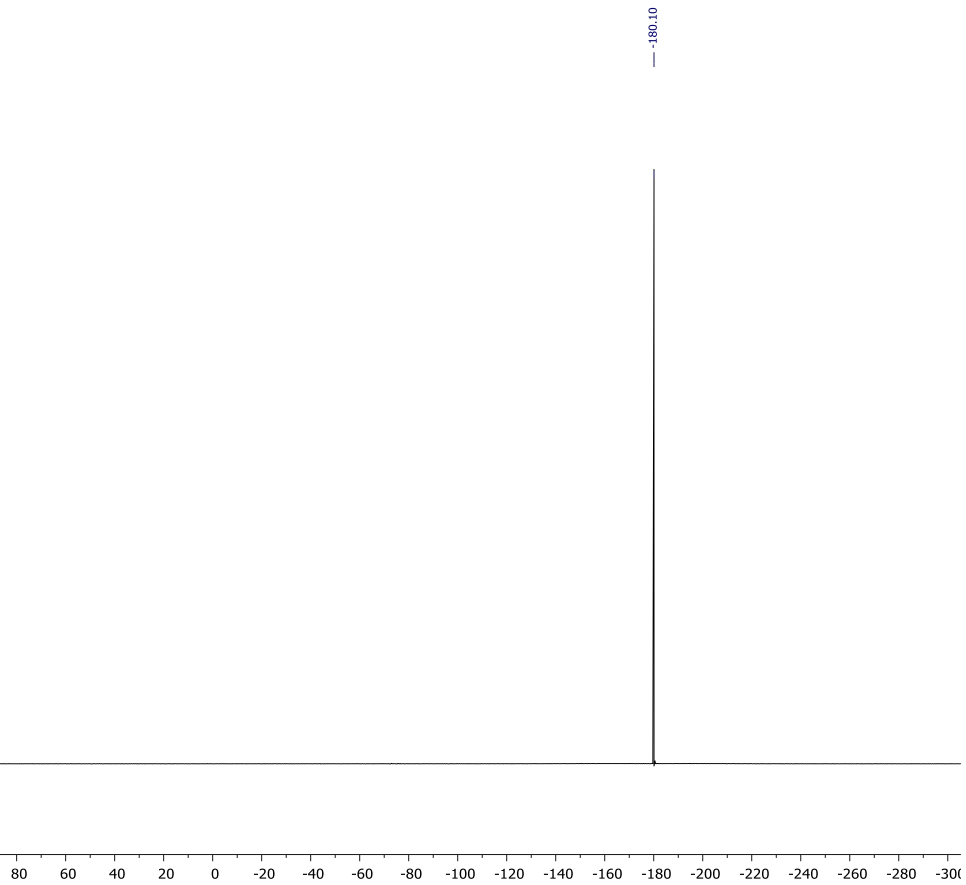
-180.10
200
180
160
140
120
100
80
60
40
20
0
-20
-40
-60
-80
-100
-120
-140
-160
-180
-200
-220
-240
-260
-280

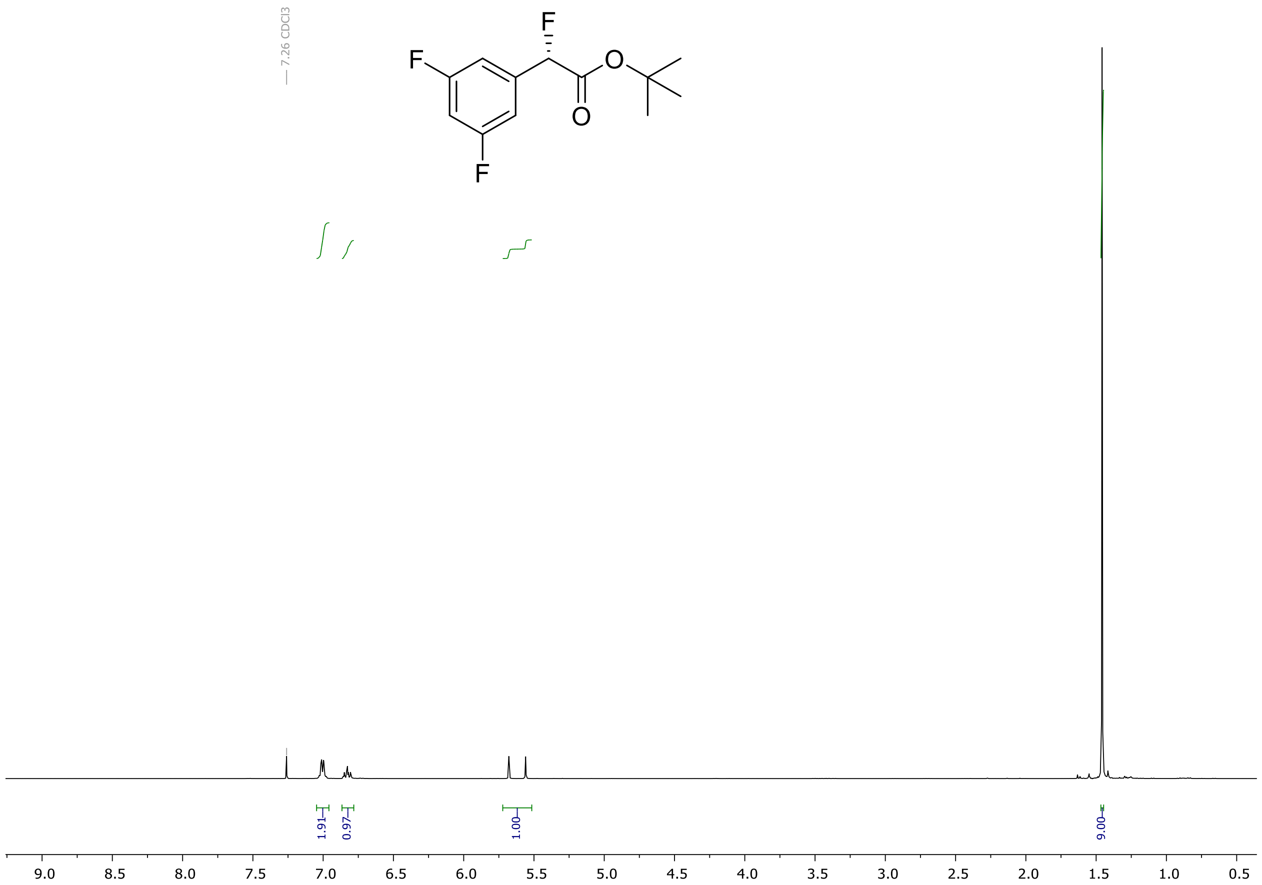
7.26 CDCl3
F
F
F
O
O
1.91
0.97
1.00
9.00
9.0 8.5 8.0 7.5 7.0 6.5 6.0 5.5 5.0 4.5 4.0 3.5 3.0 2.5 2.0 1.5 1.0 0.5

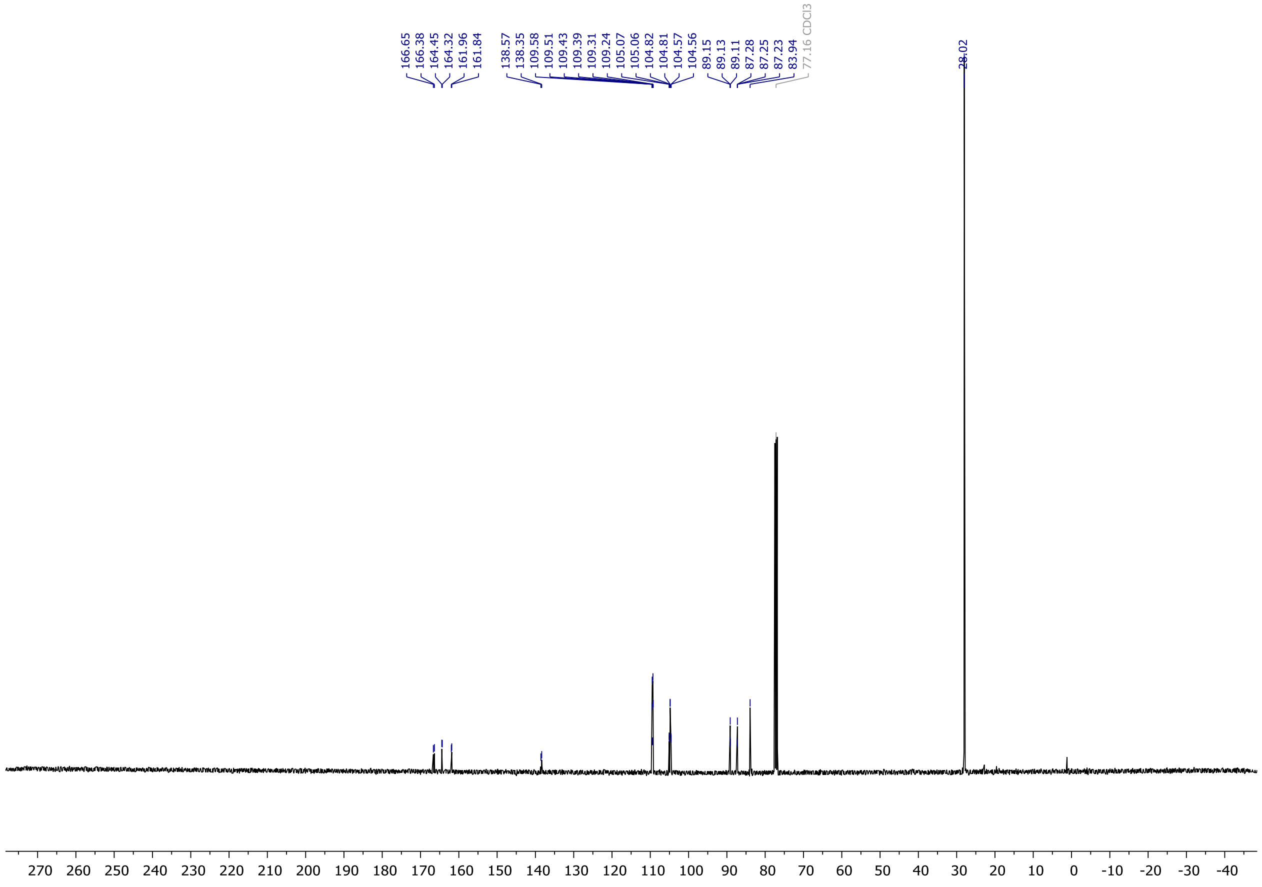
166.65
166.38
164.45
164.32
161.96
161.84
138.57
138.35
109.58
109.51
109.43
109.39
109.31
109.24
105.07
105.06
104.82
104.81
104.57
104.56
89.15
89.13
89.11
87.28
87.25
87.23
83.94
77.16 CDCl3
28.02
270 260 250 240 230 220 210 200 190 180 170 160 150 140 130 120 110 100 90 80 70 60 50 40 30 20 10 0 -10 -20 -30 -40

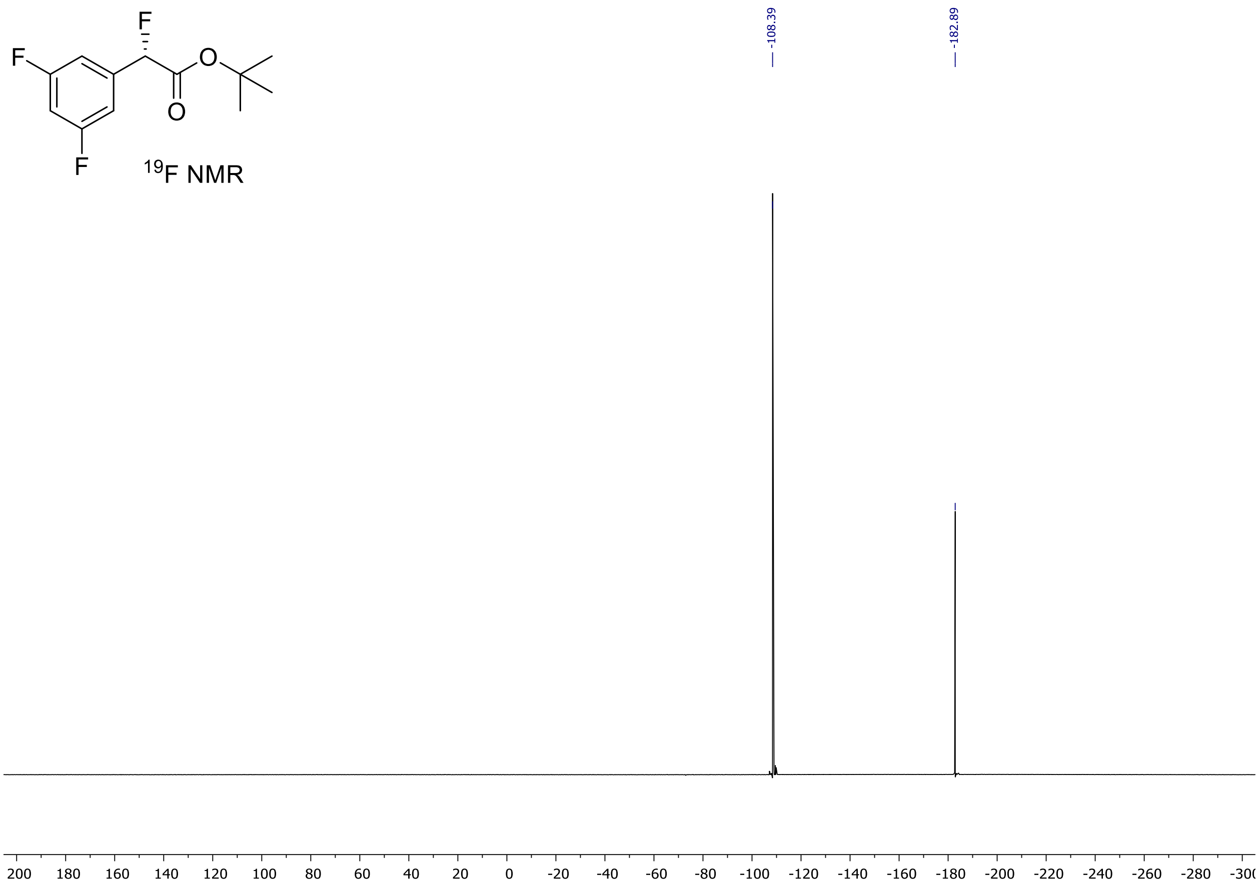

F
F
O
O
F
19F NMR
-108.39
-182.89
200 180 160 140 120 100 80 60 40 20 0 -20 -40 -60 -80 -100 -120 -140 -160 -180 -200 -220 -240 -260 -280 -300

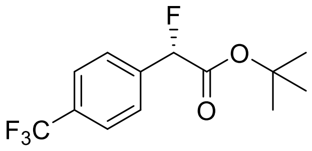
F
O
O
F3C

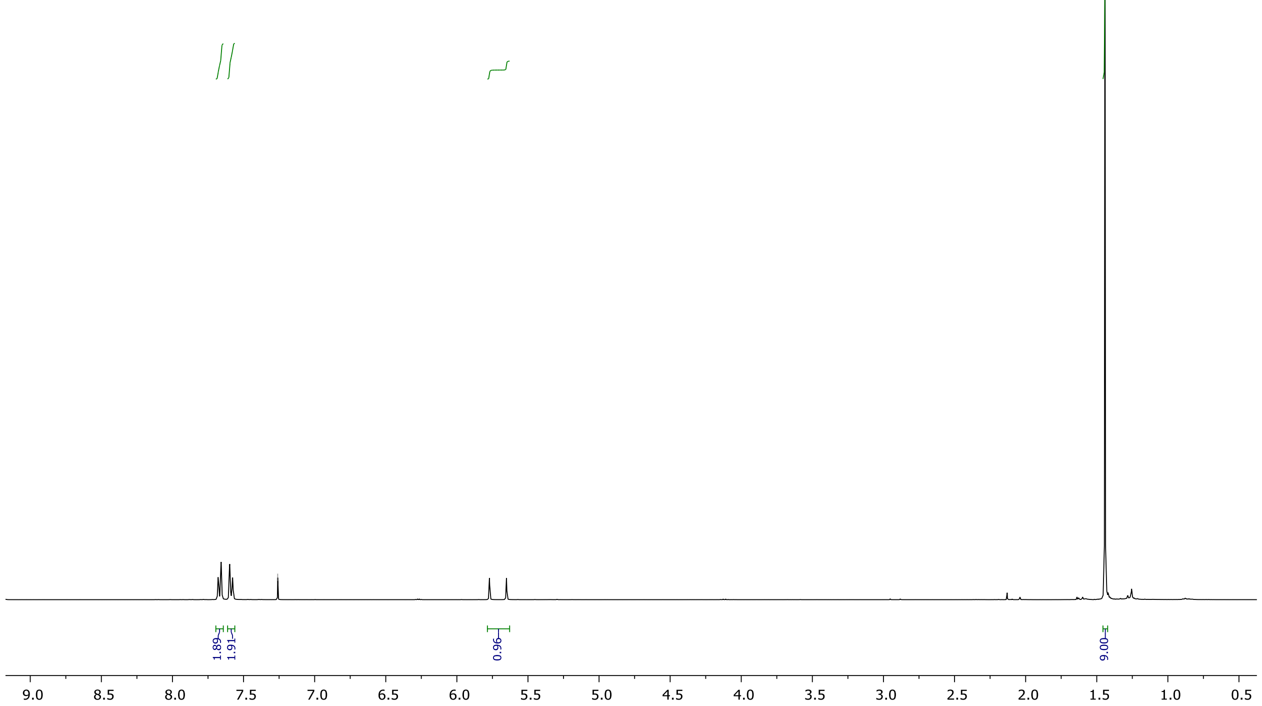
7.26 CDCl3
1.89
1.91
0.96
9.00
9.0 8.5 8.0 7.5 7.0 6.5 6.0 5.5 5.0 4.5 4.0 3.5 3.0 2.5 2.0 1.5 1.0 0.5

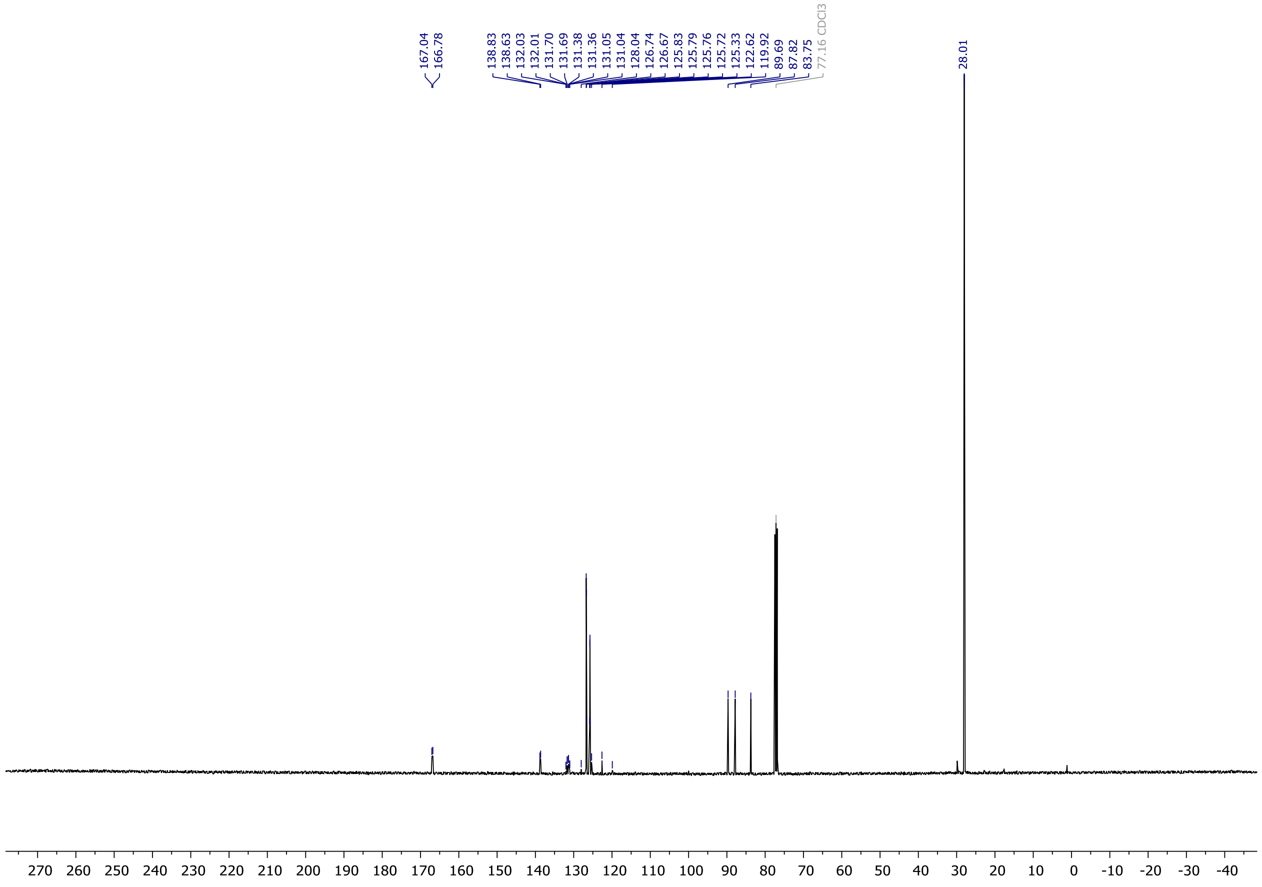
167.04
166.78
138.83
138.63
132.03
132.01
131.70
131.69
131.38
131.36
131.05
131.04
128.04
126.74
126.67
125.83
125.79
125.76
125.72
125.33
122.62
119.92
89.69
87.82
83.75
77.16 CDCl3
28.01
270 260 250 240 230 220 210 200 190 180 170 160 150 140 130 120 110 100 90 80 70 60 50 40 30 20 10 0 -10 -20 -30 -40

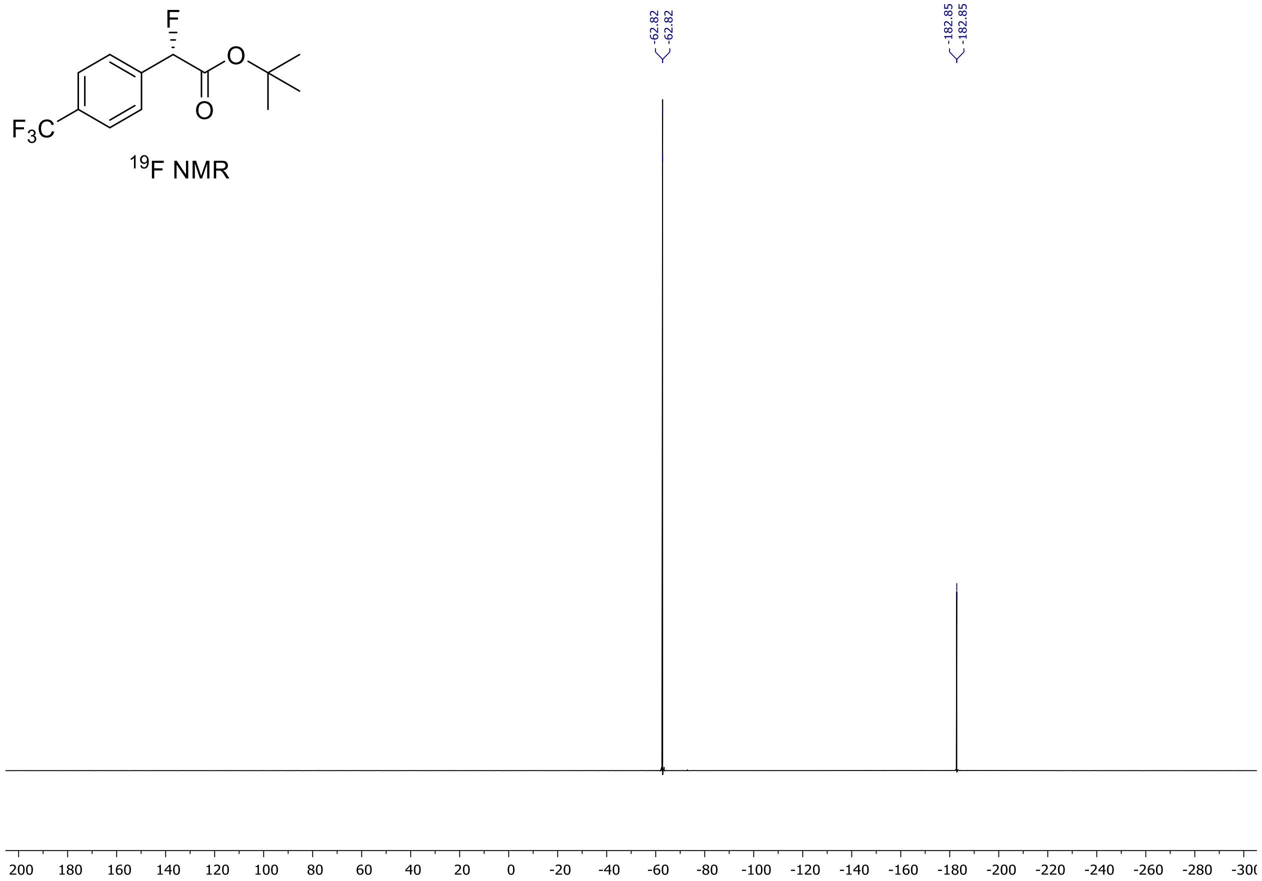
F
O
O
F3C
19F NMR
-62.82
-62.82
-182.85
-182.85
200 180 160 140 120 100 80 60 40 20 0 -20 -40 -60 -80 -100 -120 -140 -160 -180 -200 -220 -240 -260 -280 -300

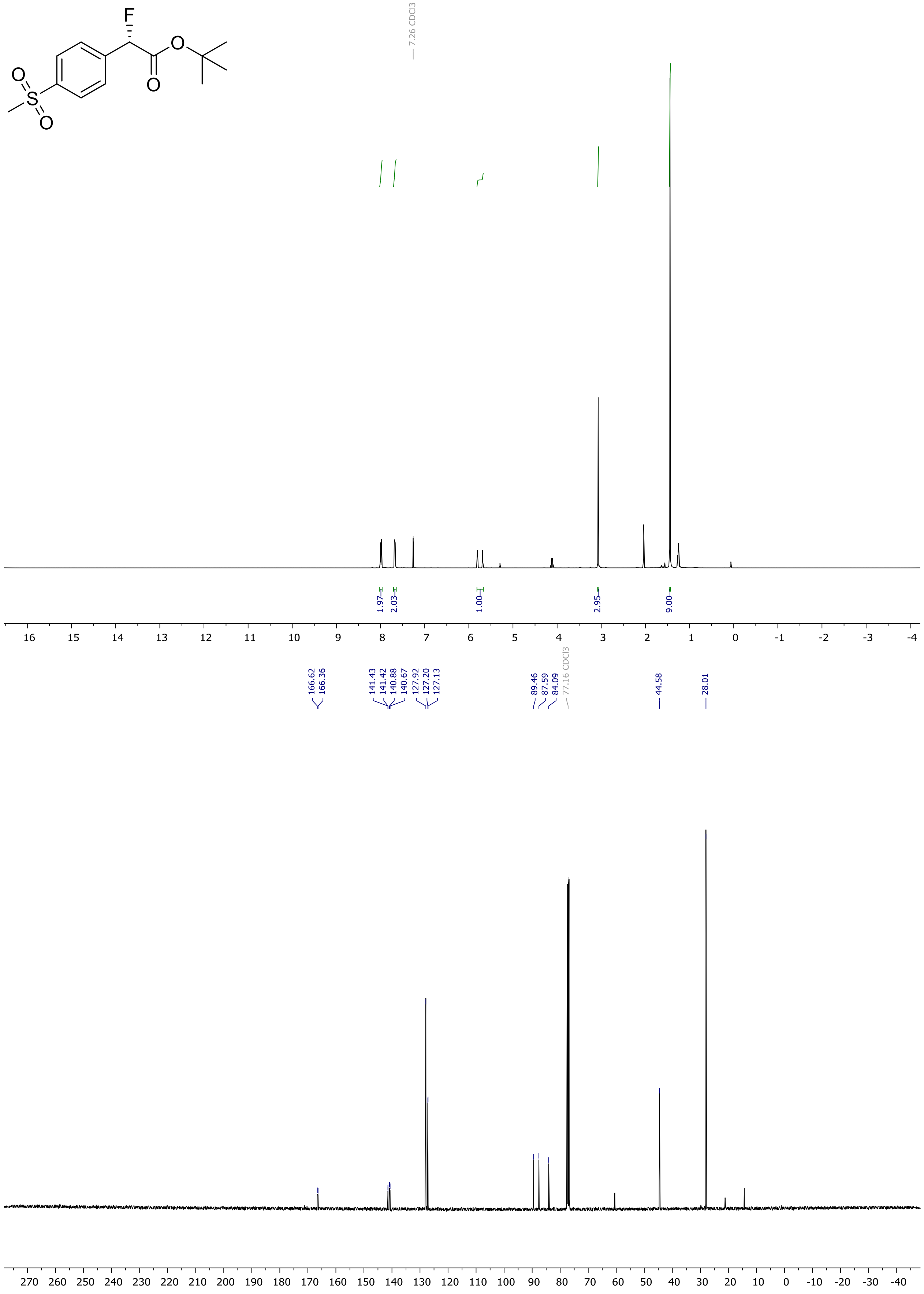
F
O
O
O
S
O
7.26 CDCl3
1.97
2.03
1.00
2.95
9.00
16 15 14 13 12 11 10 9 8 7 6 5 4 3 2 1 0 -1 -2 -3 -4
166.62
166.36
141.43
141.42
140.88
140.67
127.92
127.20
127.13
89.46
87.59
84.09
77.16 CDCl3
44.58
28.01
270 260 250 240 230 220 210 200 190 180 170 160 150 140 130 120 110 100 90 80 70 60 50 40 30 20 10 0 -10 -20 -30 -40

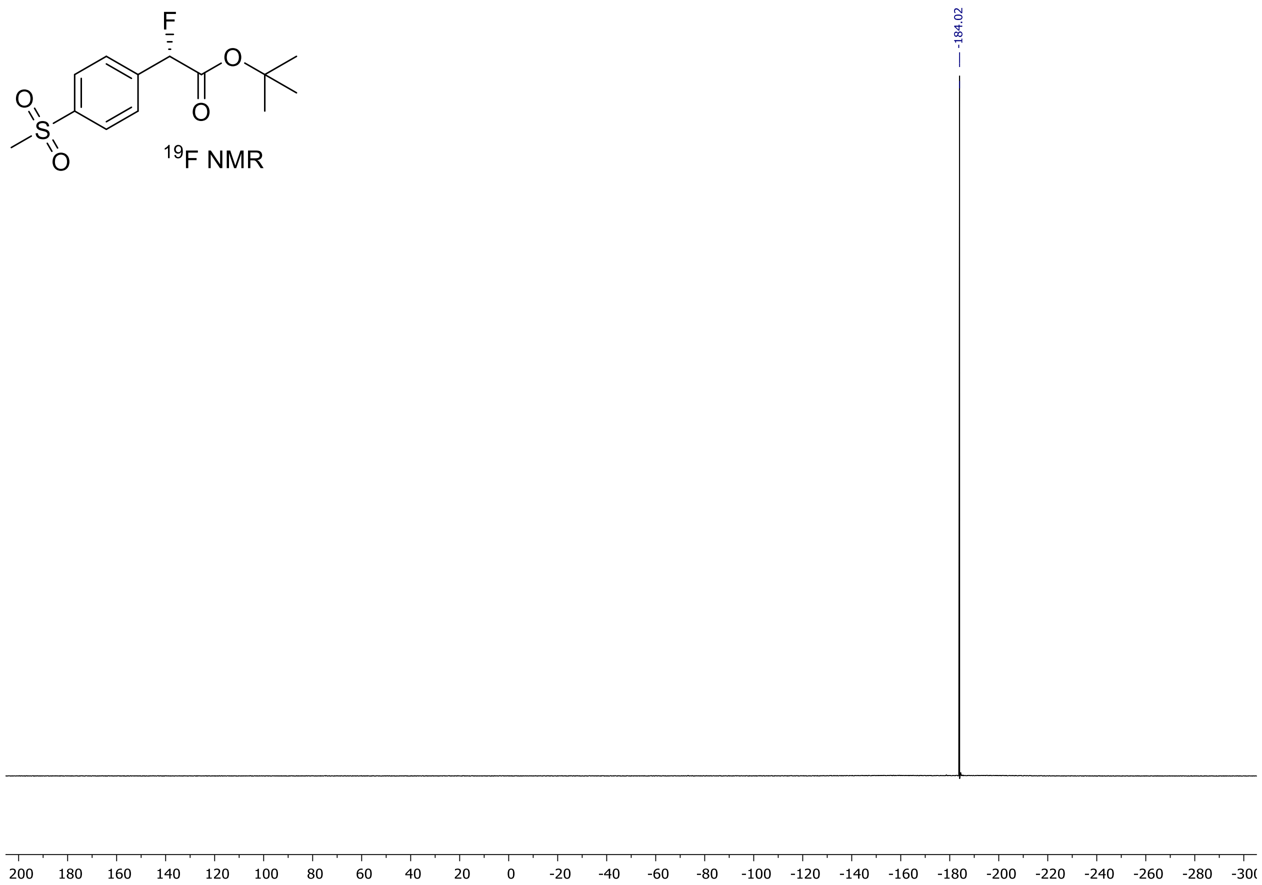

F
O
O
S
O
O
¹⁹F NMR
-184.02
200 180 160 140 120 100 80 60 40 20 0 -20 -40 -60 -80 -100 -120 -140 -160 -180 -200 -220 -240 -260 -280 -300

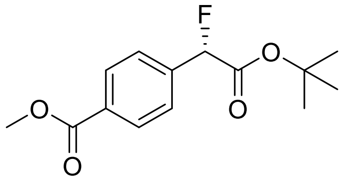
F
O
O
O
O

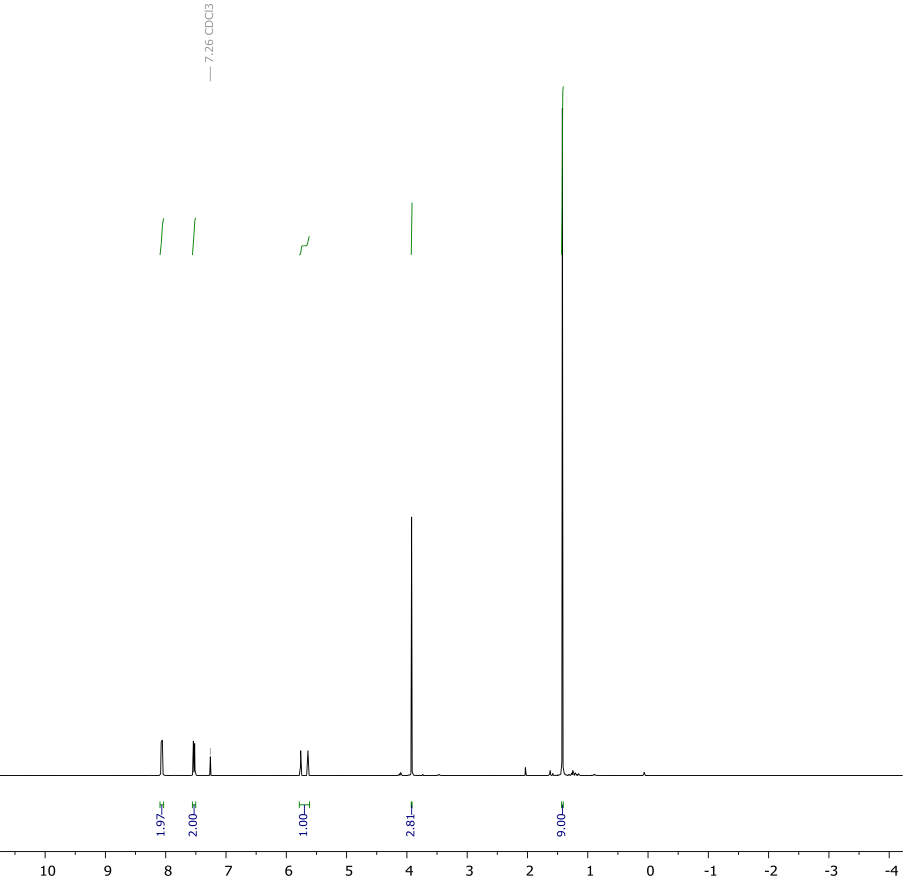
7.26 CDCl3
1.97
2.00
1.00
2.81
9.00
16 15 14 13 12 11 10 9 8 7 6 5 4 3 2 1 0 -1 -2 -3 -4

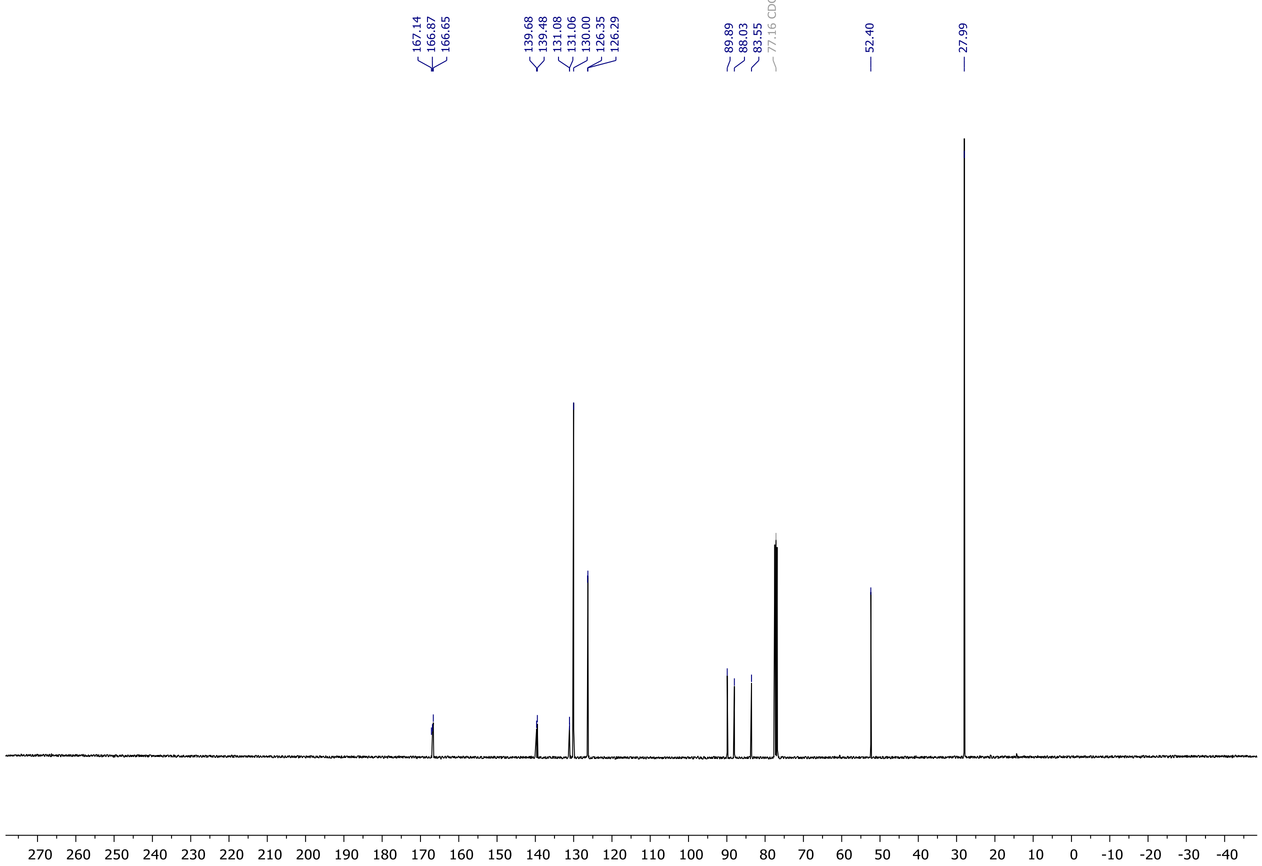
167.14
166.87
166.65
139.68
139.48
131.08
131.06
130.00
126.35
126.29
89.89
88.03
83.55
77.16 CDCl3
52.40
27.99
270 260 250 240 230 220 210 200 190 180 170 160 150 140 130 120 110 100 90 80 70 60 50 40 30 20 10 0 -10 -20 -30 -40

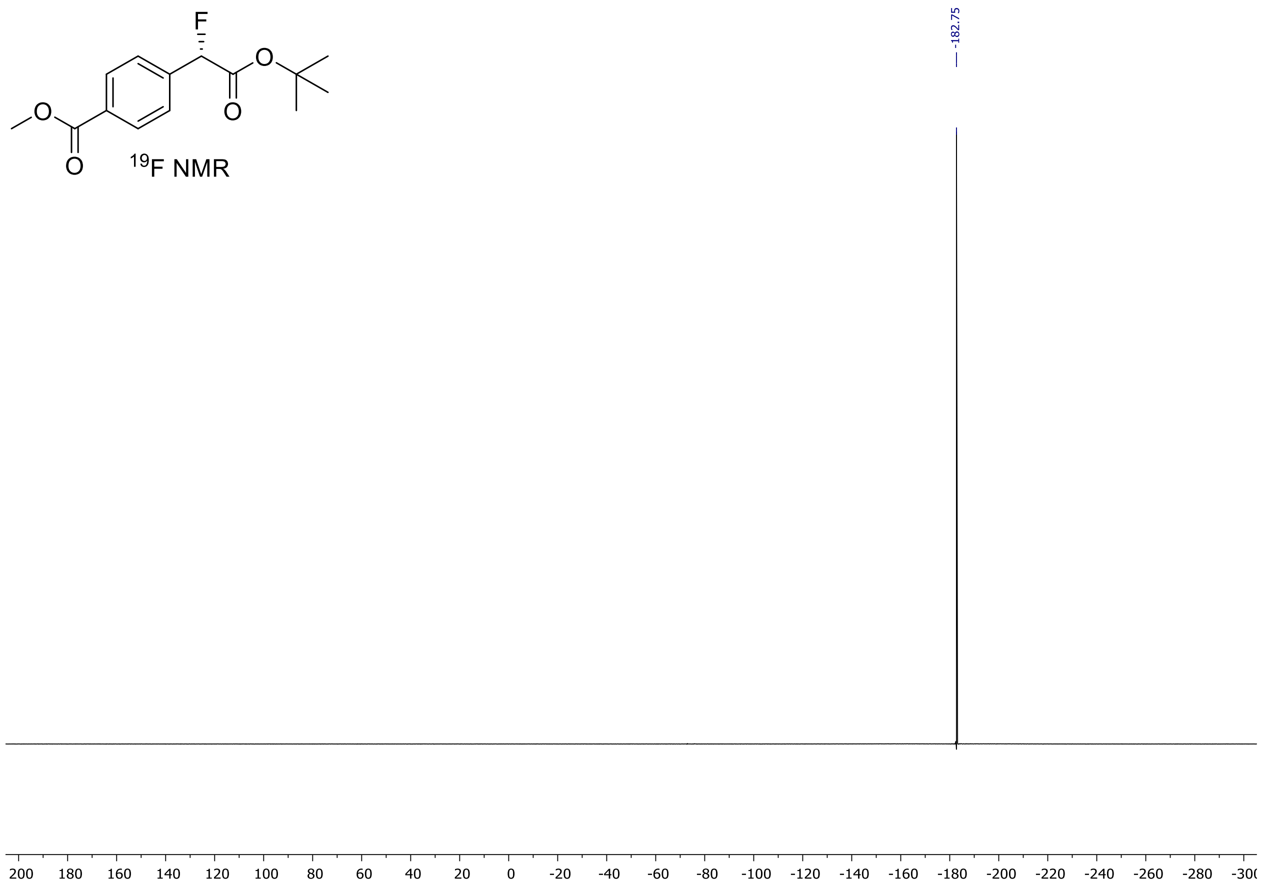

^{19}F NMR
-182.75
200
180
160
140
120
100
80
60
40
20
0
-20
-40
-60
-80
-100
-120
-140
-160
-180
-200
-220
-240
-260
-280
